# Author Correction: Dose escalation and expansion cohorts in patients with advanced breast cancer in a Phase I study of the CDK7-inhibitor samuraciclib

**DOI:** 10.1038/s41467-023-40561-x

**Published:** 2023-08-07

**Authors:** R. C. Coombes, Sacha Howell, Simon R. Lord, Laura Kenny, Janine Mansi, Zahi Mitri, Carlo Palmieri, Linnea I. Chap, Paul Richards, William Gradishar, Sagar Sardesai, Jason Melear, Joyce O’Shaughnessy, Patrick Ward, Pavani Chalasani, Tobias Arkenau, Richard D. Baird, Rinath Jeselsohn, Simak Ali, Glen Clack, Ashwani Bahl, Stuart McIntosh, Matthew G. Krebs

**Affiliations:** 1 Imperial College, South Kensington, London, UK; 2 Division of Cancer Sciences, Faculty of Biology, Medicine and Health, The University of Manchester and The Christie NHS Foundation Trust, Manchester Academic Health Science Centre, Manchester, UK; 3 Early Phase Clinical Trials Unit, Department of Oncology, University of Oxford, Oxford, UK; 4 Guy’s and St Thomas’ NHS Foundation Trust, London, UK; 5 OHSU Knight Cancer Institute, Portland, OR USA; 6 University of Liverpool, Liverpool, UK; 7 University of California, Los Angeles, CA USA; 8 Blue Ridge Cancer Center, Salem, VA USA; 9 Northwestern University, Chicago, IL USA; 10 US Oncology Research, OHC, Cincinnati, OH USA; 11 Baylor University Medical Center, Texas Oncology, Dallas, TX USA; 12 University of Arizona Cancer Center, Tucson, AZ USA; 13 Sarah Cannon Research Institute, London, UK; 14 Cancer Research UK Cambridge Centre, Cambridge, UK; 15 Dana-Farber Cancer Institute, Boston, MA USA; 16 Carrick Therapeutics, Dublin, Ireland

**Keywords:** Breast cancer, Drug development, Drug development

Correction to: *Nature Communications* 10.1038/s41467-023-40061-y, published online 24 July 2023

In this article the author name R. C. Coombes was incorrectly written as R. Charles Coombes. The original article has been corrected.

