# Supplementary material for: Dose escalation and expansion cohorts in patients with advanced breast cancer in a Phase I study of the CDK7-inhibitor samuraciclib
Source: Nat Commun. 2023 Jul 24;14:4444. doi: 10.1038/s41467-023-40061-y (PMC10366102; doi:10.1038/s41467-023-40061-y)
Supplement: Supplementary file 1 — Supplementary Information [file 41467_2023_40061_MOESM1_ESM.pdf]

SUPPLEMENTARY INFORMATION

METHODOLOGY

Module 1A:

In the Module 1A dose escalation cohort, patients initially received 1 oral dose of samuraciclib at Cycle 0, Day 1. In the absence of a dose-limiting toxicity (DLT) and following a washout period of at least 48 hours, patients could then enter Cycle 1 and receive samuraciclib daily. In the continued absence of a DLT, each patient could continue to receive samuraciclib daily for a minimum of 21 days (Cycle 1) and could continue to receive additional 21-day cycles of treatment until they were no longer gaining clinical benefit or another treatment discontinuation criterion had been met, at which time an end of treatment visit was conducted (within 3 days after the last samuraciclib dose). An end of study visit was conducted 28 to 35 days after the last dose of samuraciclib.

At least 3 and up to 6 evaluable patients were required for each dose cohort. At least 3 evaluable patients in a cohort had to complete at least one 21-day cycle of dosing before a Safety Review Committee (SRC), composed of study site investigators and Sponsor medical personnel, could review and initiate the next cohort and dose escalation. Within the initial cohorts there was a staggered interval, with subsequent patients not being dosed until the first patient within each cohort had completed 7 days of samuraciclib monotherapy.

Patient enrolment in the dose escalation cohort proceeded according to a Bayesian model rolling 6 design:

1. If no DLT was observed in a cohort of 3 to 6 evaluable patients, then dose escalation could occur.
2. If 1 patient experienced a DLT in a group of 3 or more evaluable patients, then the cohort was expanded to include 6 evaluable patients. If only 1 DLT was observed in this expanded cohort, then dose escalation occurred.

3. If at least 2 patients experienced a DLT in a cohort of up to 6 patients, irrespective of the number of patients enrolled, the dose was considered not tolerated, and recruitment to that cohort and dose escalation ceased.

The frequency of dosing, any washout period to assess PK, and dosing staggering intervals between patients could have been changed based upon emerging data as reviewed and agreed by the SRC.

Module 1A also included a breast cancer expansion cohort with sequential tumor biopsies to evaluate pharmacodynamic effects in tumor tissue. Between 6 and 12 additional patients were to be entered into this cohort, which was initiated upon definition of the minimally biologically active dose (MBAD). Paired biopsies were taken from patients dosed with samuraciclib at 240mg once daily (OD) at pre-study and Cycle 2 Day 1, and 360mg OD at pre-study and Cycle 2 Day 15. All biopsy samples were stained for 2 markers of inhibition of CDK7 activity. pCDK1/2/3 staining was evaluated using an antibody that recognized the T-loop phosphorylation site of both CDK1, CDK2, and CDK3 (abcam ab201008) and phosphorylated RNA polymerase II (pPolII) was evaluated using an antibody that recognized phosphorylated Ser 5 of the c-terminal domain repeat of RNA polymerase II. Serial sections of the biopsies were also stained to identify regions of tumor tissue and then stained for each marker. Stained sections were scanned at 20x using a Hamamtsu Nanozoomer 2.0 HT and then analyzed for the level of staining using Indica Labs HALO software (version 3.0) using a cytonuclear algorithm to identify strong (3+), moderate, (2+), weak (1+) and negative (0) for each marker. From this an H-score was calculated based on the percentage of tumor cells that fell into each staining category. Triplicate sections were analyzed for the 240mg OD biopsies, and an average was reported, but very little variability in H-score was observed between sections. Consequently, a single section was analyzed for the 360mg OD cohort.

The schedule of assessments for both parts of the study are provided in Supplementary Tables 1 and 2.

Module 1B-1:

Module 1B-1 was a cohort expansion to explore the safety, tolerability, PK and pharmacodynamic effects, and anti-tumor activity of samuraciclib when given as monotherapy to patients with advanced TNBC; participants were treated at a starting dose of 360 mg once daily (OD). The initial sample size was to be a maximum of 50 participants, of whom approximately 30 participants were to receive the CT7001 dosing regimen which was declared as the RP2D.

Module 1B-1 included an interim efficacy analysis when 15 patients in the RP2D Population were evaluable for efficacy assessments. The Module 1B-1 TNBC expansion phase was designed using a 2-stage design. The null hypothesis for the ORR was 10% and this was tested against an alternative hypothesis of ≥30%. If 0 or 1 responses were observed in the first 15 evaluable patients, no further patients were to be recruited, and the alternative hypothesis was to be rejected. Otherwise, recruitment was to continue until up to a total of 30 evaluable patients were recruited and if ≥6/30 (20%) responses were observed, across both stages of the study, the null hypothesis was to be rejected. At the timepoint for the interim efficacy assessment, the requirement of 2 or more participants achieving a Response Evaluation Criteria in Solid Tumors (RECIST) PR had not been met, with only 1 participant in the first 15 evaluable participants achieving a PR. However, 8 out of 15 evaluable participants achieved SD, with 4 participants being treated for at least 16 weeks, which in this aggressive tumor type is of significant clinical note. Following discussion with the SRC it was agreed that further evaluable participants would be recruited into this expansion study.

The schedule of assessments for this study is provided in Supplementary Table 3.

Module 2A:

Module 2A is an open-label, single-arm, ascending dose Phase 1b study intended to determine the future dosing regimen of samuraciclib and fulvestrant. The decision algorithm described below is based on

DLTs recorded in the first cycle. Patients were considered evaluable if they completed the first cycle or discontinued therapy in the first cycle due to a DLT. Patients in a cohort were replaced if they could not complete the first cycle unless this was due to a DLT.

Part A was planned to have two dose cohorts with up to 6 evaluable patients to be enrolled per cohort. In each cohort, the dose of fulvestrant was fixed at the standard dose of 500mg given at intervals of 28±2 days with an additional 500mg dose given 14±2 days after the first dose. Fulvestrant was administered as two consecutive slow intramuscular (IM) injections (1-2 minutes) of 250mg in 5 mL, one in each buttock (gluteal area). Pre-and peri-menopausal women also had to have commenced treatment with a luteinizing hormone-releasing hormone (LHRH) agonist at least 4 weeks prior to first dose of study medication. Patients continued to receive treatment in 28-day cycles until they no longer gained clinical benefit or they discontinued treatment.

Cohort 1 tested samuraciclib at 240mg OD as monotherapy, which is approximately 33% lower than the current maximum tolerated dose (MTD) of 360mg. If no DLT was recorded in Cycle 1 in the first 3 evaluable patients, Cohort 2 commenced enrolment at 360mg of samuraciclib OD. If 1/3 patients had a DLT in Cohort 1, a further 3 evaluable patients were assessed. If < 2/6 patients had a DLT, Cohort 2 started enrolment. If ≥ 2/3 or ≥ 2/6 patients in Cohort 1 experienced a DLT, dose escalation was stopped. In Cohort 2, if 0/3 or 1/3 evaluable patients experienced a DLT, the cohort was expanded to 6 patients. If ≥ 2/3 or ≥ 2/6 patients experienced a DLT, the Cohort 1 dosing regimen (samuraciclib at 240mg and fulvestrant at 500mg) was determined as the preliminary Phase 2 dosing regimen. If < 2/6 evaluable patients in Cohort 2 had a DLT and at least 3 patients had completed at least 2 cycles, this was considered the recommended Phase 2 regimen.

The SRC was convened to decide whether and when to open the second samuraciclib dosing cohort and which dosing regimen to advance to randomized Phase 2 testing. If the SRC considered safety and/or

tolerability data as borderline, additional patients in sets of 3 were to be enrolled in Part A for further assessment.

Module 2A was estimated to require approximately 12 patients who completed Cycle 1 to confirm the dose to progress the study. Following review by the SRC, additional patients were enrolled into Module 2A to yield 20 evaluable patients.

The schedule of assessments for this study is provided in Supplementary Table 4.

INCLUSION AND EXCLUSION CRITERIA

To be included in either Module 1A, Module 1B-1 or Module 2A, patients had to meet all the following criteria:

Inclusion Criteria

1. At least 18 years of age.
2. Eastern Cooperative Oncology Group (ECOG) performance status 0 or 1 with no deterioration over the previous 2 weeks.
3. Estimated life expectancy of greater than 12 weeks.
4. Ability to swallow and retain oral medication.
5. Women of childbearing potential had to practice effective contraception. This included:
    a. Abstinence if consistently employed as the patient's preferred and usual lifestyle.
    b. Sex only with a person of the same sex or with vasectomized partner.
    c. Medroxyprogesterone injections (e.g., Depo-Provera), levonorgestrel intrauterine system (e.g., Mirena), intrauterine device (IUD), or barrier method (e.g., condom, diaphragm) for the duration of the study and for 6 months after the last dose of samuraciclib.

    Note: Contraceptives that are prone to drug-drug interactions may not be effective because of a potential CYP3A4 interaction with samuraciclib.

6. Women not of childbearing potential were defined as women who were postmenopausal (defined as ≥50 years of age, amenorrhoeic for at least 12 months after cessation of all exogenous hormonal treatments, and with serum follicle stimulating hormone [FSH], luteinizing hormone [LH] and plasma estradiol levels in the testing laboratory's postmenopausal range). Women under 50 years old were considered postmenopausal if they had been amenorrhoeic for 12 months or more following cessation of exogenous hormonal treatments and with LH and FSH levels in the post-menopausal range for the institution, or women who had been amenorrhoeic for at least 12 months and were less than 50 years of age. Women who were surgically sterilized (defined as documented irreversible surgical sterilization by hysterectomy or by bilateral tubal ligation, oophorectomy, or salpingectomy) were also considered to be not of childbearing potential.
7. Sexually active male patients had to be willing to use condoms with all sexual partners for the duration of the study and for 3 months after the last dose of samuraciclib. If a female partner was a woman of childbearing potential who was not using effective contraception, the patient had to use a condom with spermicide during the study and for 6 months after the last dose of samuraciclib.
8. Provision of signed and dated, written informed consent before any study-specific procedures, sampling, or analyses, including access to archival tumor tissue.

Patients who met the following criteria could be included in the optional pharmacogenomics sub-studies:

1. Provision of signed and dated, written informed consent for the genetic research.

Patients who met the following criteria could be included in Module 1A:

1. Histological, radiological, or cytological confirmation of an advanced non-hematological malignancy not considered to be appropriate for further standard treatment.

2. For inclusion in the paired biopsy expansion cohort in breast cancer patients, patients had to have histological, radiological, or cytological confirmation of breast cancer not considered to be appropriate for further standard treatment.

Patients who met the following criteria could be included in Module 1B-1:

1. Histologically confirmed carcinoma of the breast not expressing oestrogen receptor (ER) and progesterone receptor (PgR) and negative for human epidermal growth factor receptor 2 (HER2).

    - Assessment of ER, PgR and HER2 in breast carcinoma tissue will be based on results from local pathology laboratories. Independent central review is not intended.

    - Negative assessment for ER, PgR and HER2 by local laboratories should be consistent with the criteria described in the most recent versions of the ASCO/CAP guidelines for testing of ER, PgR and HER2, respectively (Hammond et al, 2010; Wolff et al, 2018):

        - ER- and PgR-negativity is determined as <1% of tumour cells positive by IHC utilizing an IHC assay consistent with local standards.

        - HER2-negativity is determined as immunohistochemistry score 0/1+ or negative by in situ hybridization (FISH/CISH/SISH) defined as a HER2/CEP17 ratio <2 or for single probe assessment a HER2 copy number <4.

        - Determination of negative ER, PgR and HER2 status should be based on data from the most recent tumour biopsy. In case no tumour biopsy was

performed after initial resection of the primary tumour, the original tumour tissue serves as basis for assessment of ER/PgR/HER2 status.

2. Metastatic or locally advanced disease not amenable to resection or radiation therapy with curative intent with documented progressive disease on or within 6 months of most recent prior chemotherapy.

3. Disease must be measurable by RECIST, version 1. 1

4. Patients must have received at least one cytotoxic chemotherapy regimen for metastatic/locally advanced disease.

Patients who met the following criteria could be included in Module 2A:

1. Post-menopausal or if pre- or peri-menopausal then amenable to treatment with an LHRH agonist.
2. Histologically confirmed diagnosis of carcinoma of the breast with evidence of metastatic or locally advanced disease, not amenable to resection or radiation therapy with curative intent.
3. Documentation of ER-positive and/or PgR-positive tumor based on most recent tumor biopsy utilizing an assay consistent with local standards. ER- and PgR-positivity were defined as ≥1% positive stained cells[S1].
4. Documentation of HER2 negativity based on local testing on most recent tumor biopsy. HER2-negativity was defined as immunohistochemistry score 0/1+ or negative by *in situ* hybridization (FISH/CISH/SISH) defined as a HER2/CEP17 ratio <2 or for single probe assessment a HER2 copy number <4[S2]. If no tumor biopsy was performed after initial resection of the primary tumor, the original tumor tissue served as the basis for assessment of ER/PgR and HER2 status. Assessment of ER, PgR and HER2 status was based on results from local pathology laboratories.
5. Measurable disease as defined by RECIST version 1.1.
6. Patients had to have documented objective disease progression while on or within 6 months after

the end of the most recent therapy.

a. Patients must have received an aromatase inhibitor together with a CDK4/6 inhibitor in the same line of therapy for the treatment of locally advanced or metastatic disease or early breast cancer, if the disease-free interval, between initiation of adjuvant therapy and first line treatment of locally advanced or metastatic disease, was <12 months. In addition, the following prior therapies for locally advanced or metastatic disease were allowed: up to two lines of endocrine treatment (prior fulvestrant was not allowed), everolimus, and one line of prior chemotherapy for locally advanced or metastatic disease.

Exclusion Criteria

Patients who met any of the following criteria were excluded from the 3 modules:

1. Any other malignancy which had been active or treated within the past 3 years, with the exception of cervical intraepithelial neoplasia and non-melanoma skin cancer.
2. Any unresolved toxicity (except alopecia) from prior therapy of ≥Grade 2 according to Common Terminology Criteria for Adverse Events (CTCAE).
3. Spinal cord compression or brain metastases, unless asymptomatic, stable, and did not require steroids for at least 4 weeks before the first dose of study medication (if stable and requiring no intervention, the patient could be enrolled in the study).
4. Refractory nausea and vomiting, chronic gastrointestinal diseases or previous significant bowel resection with clinically significant sequelae that precluded adequate absorption of samuraciclib.
5. Uncontrolled seizures.
6. Active infection that required systemic antibiotic, antifungal, or antiviral medication within 14 days prior to the first dose of samuraciclib.
7. Severe or uncontrolled medical condition (e.g., severe chronic obstructive pulmonary disease, severe Parkinson’s disease, active inflammatory bowel disease) or psychiatric condition.
8. Active bleeding diatheses.

9. Renal transplant.
10. Known hepatitis B, hepatitis C, or human immunodeficiency virus (HIV) infection.
11. Breastfeeding or pregnancy.
12. Receipt of cytotoxic treatment for the malignancy within 28 days or ≤5 half-lives, whichever was shorter, before the first dose of study medication.
13. Receipt of non-cytotoxic treatment for the malignancy within 5 half-lives of the drug before the first dose of study medication.
14. Receipt of systemic corticosteroids (at a dose >10mg prednisone/day or equivalent) within 14 days before the first dose of study medication.
15. Receipt of any small-molecule investigational product within 28 days or ≤5 half-lives, whichever was shorter, before the first dose of study medication.
16. Receipt of any biological investigational product (e.g., immune checkpoint blockers, antibodies, nanoparticles) within 42 days before the first dose of study medication.
17. Receipt of St John's Wort within 21 days before the first dose of study medication. or of another concomitant medication, herbal supplement, or food that was a strong inhibitor or inducer of CYP3A4, CYP2C19, CYP2D6, or P-glycoprotein activity within 14 days before the first dose of samuraciclib.
18. Receipt of a blood transfusion (blood or blood products) within 14 days before the first dose of study medication.
19. Known hypersensitivity to samuraciclib or any excipient of the product.
20. Impaired hepatic or renal function, as demonstrated by any of the following laboratory values:
    a. Albumin <30 g/L.
    b. Aspartate aminotransferase (AST) or alanine aminotransferase (ALT) >2.5 × the upper limit of normal (ULN).
    c. >5.0 × ULN for patients with liver metastases.
    d. Total bilirubin >1.5 × ULN.

e. Serum creatinine >1.5 × ULN.

21. Liver function had deteriorated in a manner that would likely make the patient meet the AST, ALT, or bilirubin levels specified above at the time of the first dose of study medication.
22. Other evidence of impaired hepatic synthesis function.
23. Inadequate bone marrow reserve or organ function, as demonstrated by any of the following laboratory values:
    a. Absolute neutrophil count (ANC) <1.5 × 10^9/L.
    b. Platelet count <100 × 10^9/L.
    c. Hemoglobin <90 g/L.
24. Persistent (>4 weeks) severe pancytopenia due to previous therapy rather than to disease (ANC <0.5 × 10^9/L or platelets <50 × 10^9/L).
25. Cardiac dysfunction (defined as myocardial infarction within 6 months of study entry, New York Heart Association [NHYA] Class II/III/IV heart failure, unstable angina, unstable cardiac arrhythmias, or left ventricular ejection fraction <55%).
26. Mean resting QT interval corrected for heart rate by the Fridericia formula (QTcF) >470 msec obtained from 3 electrocardiograms (ECGs) obtained within 5 minutes of each other prior to the first dose.
27. Any clinically important abnormalities in rhythm, conduction, or morphology on resting ECG (e.g., complete left bundle branch block, third degree heart block). Controlled atrial fibrillation was permitted.
28. Any factor that increased the risk of QTc prolongation or arrhythmic events (e.g., heart failure, hypokalemia, congenital long QT syndrome, immediate family history of long QT syndrome or unexplained sudden death under 40 years of age).
29. In the opinion of the investigator, unlikely to comply with study procedures, restrictions, or requirements.
30. A history of hemolytic anemia or marrow aplasia.

31. Had received a live-virus vaccination within 28 days or less of planned treatment start (note: seasonal flu vaccines that did not contain live virus were permitted).

Patients who met the following criteria were excluded from the optional pharmacogenomics sub-studies:

1. Allogenic bone marrow transplant.
2. Non-leucocyte-depleted whole blood transfusion within 120 days of the date of the genetic sample collection.

Patients who met the following criteria were excluded from Module 1A:

1. International normalized ratio (INR) ≥1.5.

Patients who met the following criteria were excluded from Module 1B-1:

1. Patients must not have received more than three lines of cytotoxic chemotherapy for metastatic and/or locally advanced disease.

Patients who met the following criteria were excluded from Module 2A:

1. Prior therapy with fulvestrant.
2. More than 2 lines of endocrine treatment for locally advanced or metastatic disease.
3. Patients with liver metastasis could only be enrolled upon approval by the medical monitor.

STATISTICAL METHODS AND DEFINITIONS

Anti-tumor activity: Anti-tumor activity endpoints were analyzed in each module using the Evaluable for Response Population (defined as all patients who received at least 1 dose of samuraciclib, had a baseline RECIST assessment and had at least 1 other post-baseline RECIST assessment). Best objective response (BOR) was determined for each patient based on the best response recorded from the start of study treatment to the end of treatment. Objective response rate (ORR) was defined as the percentage of

patients who had at least one response of complete response (CR) or partial response (PR) prior to any evidence of progression. The percentage change in tumor size was derived at each tumor imaging assessment by the best percentage change from baseline in the sum of the diameters of target lesions. In Module 1A initial efficacy was evaluated using the disease control rate (DCR) defined as percentage of participants with a complete response (CR) or partial response (PR) or stabilization of disease at first on treatment RECIST assessment. In Modules 1B-1 and Module 2A the clinical benefit rate (CBR) was estimated by dividing the number of patients with CR, PR, or stable disease (SD) ≥24 weeks by the number of patients in the particular analysis population. Duration of response (DoR) was defined as the time from documentation of tumor response to disease progression. Progression-free survival (PFS) was defined as the time from start of treatment until the date of objective disease progression or death by any cause, whichever occurred first. Efficacy results were summarized descriptively and Kaplan-Meier plots provided where appropriate.

Biomarker analyses were performed using the Biomarker Population (defined as all patients who received at least 1 dose of samuraciclib and provided at least 1 biomarker sample). Summaries of change from baseline and percent change from baseline were also summarized by visit and cohort.

Safety analyses were performed in each module using the Safety Population (defined as all patients who received at least 1 dose of samuraciclib). These were summarized by Medical Dictionary for Regulatory Activities (MedDRA) system organ class (SOC), preferred term (PT), and CTCAE grade. Serious adverse events (SAEs) were analyzed and reported separately. Details of any deaths were listed. All laboratory results, ECOG performance status, ECG parameters, weight and vital sign measurements were listed individually by patient and summarized descriptively. For urinalysis parameters, any qualitative assessments were summarized using the number of patients with results of negative, trace, or positive. Abnormal physical examination findings were listed.

Samuraciclib pharmacokinetic (PK) analyses were performed using the PK Population (defined as all patients who received at least 1 dose of samuraciclib and who had at least 1 samuraciclib plasma concentration above the lower limit of quantification and no important AEs or protocol deviations or other event that could have impacted the PK analysis). PK parameters were derived using standard non-compartmental methods, with graphical presentations of PK data as appropriate. Samuraciclib plasma concentrations and derived PK parameters were summarized by dose level. Parameters following single and multiple dosing were summarized separately. Appropriate statistics were used to summarize plasma concentrations and PK parameters.

Genetic analysis of circulating tumor DNA (ctDNA) was performed using the TARGET molecular profiling platform[S3]. Samples were analyzed from 27 out of 31 patients, with the other 4 patients being non-evaluable. Where possible, ctDNA from 2 blood samples were analyzed, typically from screening and Cycle 3 Day 1 timepoints, and high confidence mutations in either sample were deemed to be indicative of a mutation in a given gene.

Statistical associations between time to progression and demographic data, prior therapies, location of metastatic disease and presence of a mutation in a gene were assessed using a Mann-Whitney U-test, and where a potential association was suspected, this was further assessed using a Kaplan-Meier analysis with a log-rank test used to determine significance.

DOSE-LIMITING TOXICITIES AND MAXIMUM TOLERATED DOSE

A DLT was defined as any toxicity not attributable to the disease or disease-related processes under investigation that occurred before the end of Cycle 1 and which included:

- Hematological toxicities:
  - Grade 4 neutropenia (ANC <500 cells/mm^3) lasting longer than 4 consecutive days
  - Grade 3 neutropenia (ANC ≥500 to <1000 cells/mm^3) of any duration accompanied by fever

≥38.5°C or systemic infection

  - Grade 3 thrombocytopenia (25,000 to <50,000 cells/mm^3) with bleeding
  - Any other confirmed hematological toxicity ≥CTCAE Grade 4 (a repeat may be required for confirmation of an isolated abnormality in the absence of clinical signs, symptoms or other abnormal investigations, i.e., a suspected spurious value).

- Non-hematological toxicity ≥CTCAE Grade 3 including:
  - Laboratory abnormalities (a repeat may be required for confirmation of an isolated abnormality in the absence of clinical signs, symptoms or other abnormal investigations, i.e., a suspected spurious value)
  - QTcF prolongation (>500 msec and/or + 60 ms)
  - Any other toxicity that was greater than that at baseline and was clinically significant or unacceptable or did not respond to supportive care
  - Any event, including significant dose reductions or omissions, judged to be a DLT by the SRC.

The definition of a DLT excluded:

- Alopecia of any grade.
- Inadequately treated Grade 3 nausea, vomiting, or diarrhea (all patients were to receive optimal anti-emetic or anti-diarrheal prophylaxis or treatment)
- Any toxicity clearly unrelated to CT7001 treatment, e.g., solely related to the disease or disease-related process under investigation
- Isolated laboratory changes of any grade without clinical sequelae or clinical significance.

Once the non-tolerated dose was defined, the MTD was confirmed as the previous dose level below the non-tolerated dose, or a dose between the non-tolerated dose and the last tolerated dose. Six evaluable participants were required to determine the MTD. The MTD was determined and agreed by the SRC and

with the sponsor. All investigator sites attended the SRC so that updates on toxicity and MTD could be communicated immediately. No more than 6 participants could be recruited at a non-tolerated dose level.

A summary of DLTs by cohort is provided in Supplementary Table 5.

EFFICACY SUBGROUP ANALYSIS

Two parameters were found to be associated with increased likelihood of benefit: *TP53* mutation status, and the absence of liver metastases (Supplementary Figure 4).

BIOMARKER METHODOLOGY

The following sample preparation method was used:

1) Cells were pelleted and resuspended in 2 mL 70% Ethanol and incubated on ice for 1 hour.
2) Cells were then pelleted and then washed and resuspended in ice-cold PBS.
3) Cells were then pelleted and resuspended in 200 µL of FACS buffer (PBS +0.5% BSA) containing primary antibody at a 1:100 dilution (see Supplementary table 12 for antibodies used).
4) Cells then incubated for 1 hour on ice.
5) Wash cells with 1 mL ice-cold PBS and re-suspend in 200 µL of FACS buffer (PBS +0.5% BSA) containing the secondary antibody (Goat anti-rabbit AF488, 1:1000 dilution).
6) Place the tubes on ice for 1 hour
7) Wash cells with 1 mL ice-cold PBS and re-suspend in 500 µL ice cold PBS. Samples were stored at 4°C prior to flow cytometry analysis.

Cells were analysed cells on a BD Fortessa X20, acquiring 100,000 events. Cells are gated by side scatter area (SSC-A) versus forward scatter area (FSC-A) to separate the three populations of cells: lymphocytes, monocytes and granulocytes (see Supplementary Figure 11). Data were exported and

analysed using FlowJo (Treestar) version 10.4.2 to report the median fluorescence intensity of staining in the cell populations.

BIOMARKER RESULTS

Supplementary Figure 5 shows a reduction of pPolII by approximately 30% across all dose levels tested in Module 1A after induction of lymphocytes by samuraciclib in patients who completed 21 days of treatment (i.e., Cycle 2 Day 1 timepoint). Later timepoints also showed inhibition (data on file), although not reaching statistical significance due to the low number of patients. pPolII was determined by a flow cytometry assay on samples of peripheral blood mononuclear cell (PBMC) preparations derived from blood. The data show box plots for each sampling timepoint normalized to the screening sample for that patient. Note that samples were taken pre-dose at all timepoints. Statistical comparison between pre-treatment samples (Cycle 1 Day 1) and subsequent timepoints were conducted using a 1-tailed Mann-Whitney test with Bonferroni correction.

H-scores for pre-dose and on-study biopsies and summary statistics for the observed H-score when stained for pCDK1/2/3 and pPolII are shown in Supplementary Table 11. Significance was calculated using Student's one-tailed paired t-test, where the alternative hypothesis was that on-study samples had reduced H-score.

SUPPLEMENTARY FIGURE 1: CONSORT DIAGRAMS

Module 1A

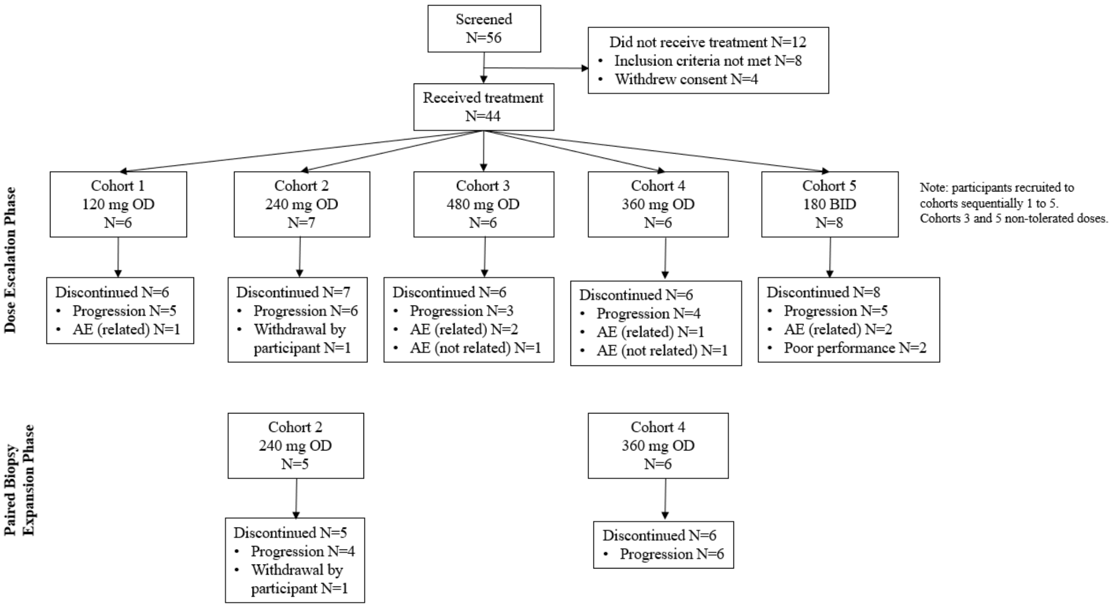

Module 1B-1

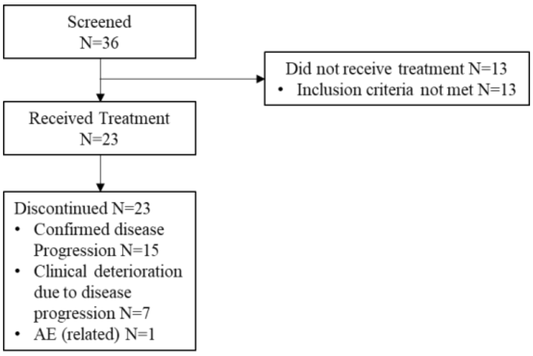

Module 2A

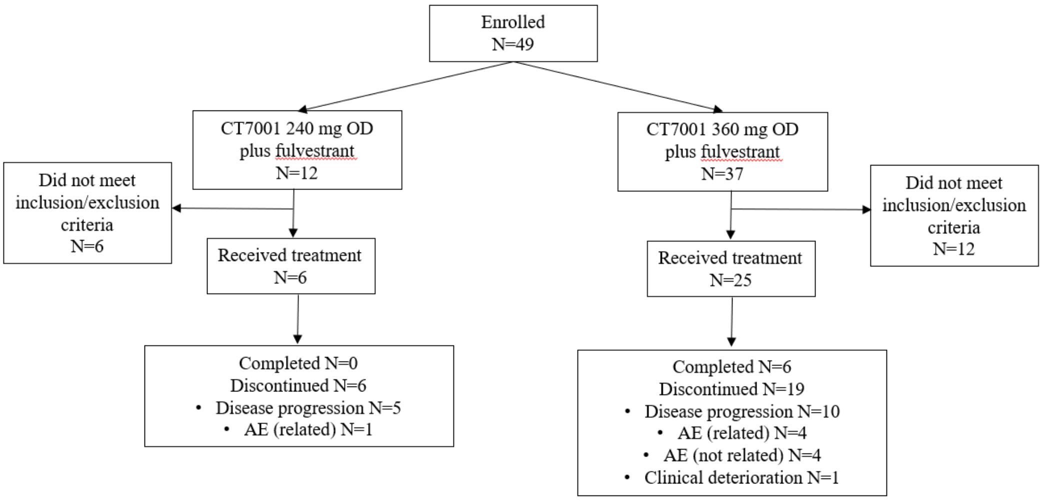

AE=adverse event; BID=twice daily; OD=once daily

SUPPLEMENTARY FIGURE 2: MECHANISM OF ACTION

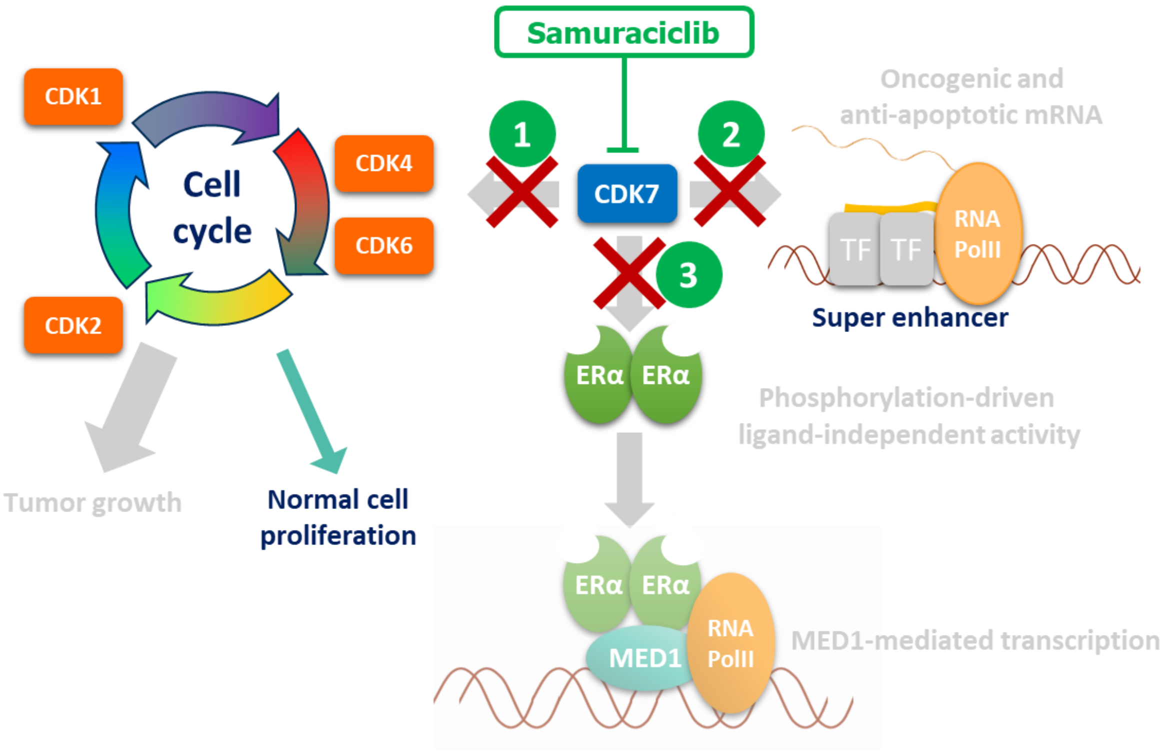

Samuraciclib blocks CDK7-mediated oncogenic effects on 1) the cell cycle through phosphorylation of other CDKs, 2) transcription of oncogenic and anti-apoptotic genes, and 3) signalling by and activation of hormone receptors.
CDK=cyclin-dependent kinase; ER=estrogen receptor; MED1=mediator subunit 1; mRNA=messenger ribonucleic acid; pPolII=phosphorylated RNA polymerase II; RNA=ribonucleic acid

SUPPLEMENTARY FIGURE 3: PLATELET LEVELS

(a)

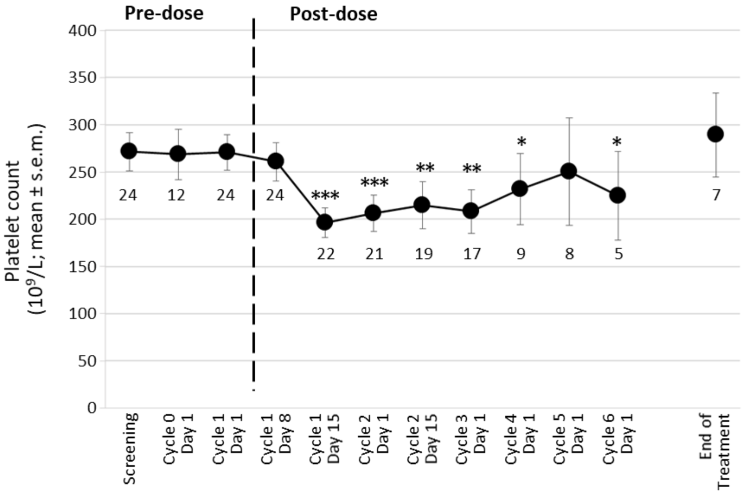

(b)

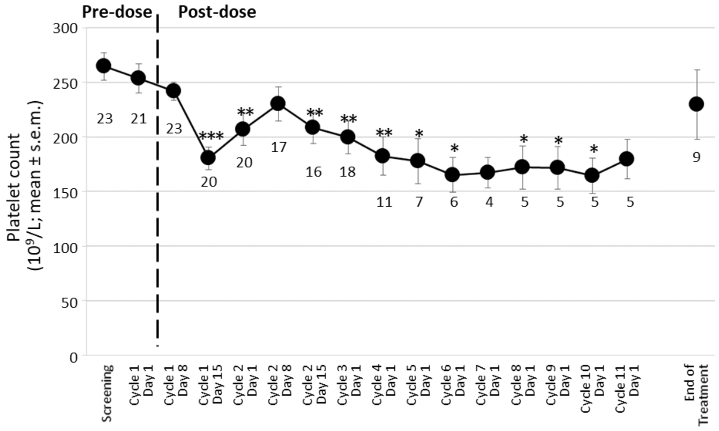

(c)

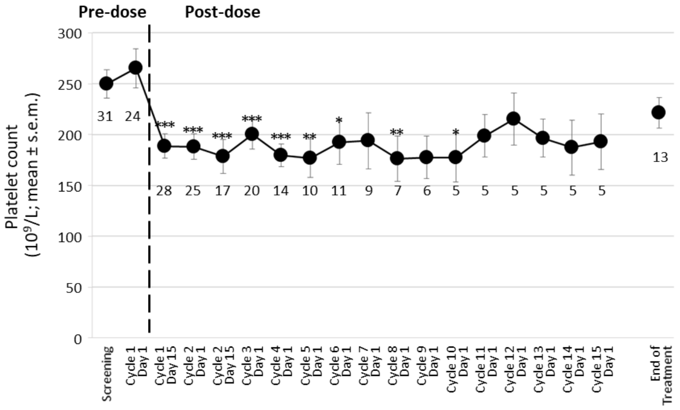

* p < 0.05
** p < 0.01
*** p < 0.001

Wilcoxon signed-rank test vs. screening sample
p-values are for a two-tailed test

Data labels show patient number

End of treatment data shown where sample was obtained ≥ 3 days after last recorded dose of samuraciclib (median 15 days, range 3 – 41 days)

a). Module 1A dosed with 240mg OD or 360mg OD samuraciclib, b). Module 1B-1 dosed with 360mg OD samuraciclib, c). Module 2A dosed with 240mg OD or 360mg OD samuraciclib in combination with fulvestrant,
For all panels: data are mean ± standard error of the mean (SEM) for patients sampled at the indicated timepoints and continuing on study. Patient numbers at each timepoint are shown on the data labels. Data were excluded where n<5. End of treatment is shown discontinuously as the data point summarizes information from the variable timepoints at which patients discontinued. Significance was assessed using a Wilcoxon two-tailed signed-rank test by comparison to the matched screening sample for each patient (where no symbol shown p>0.05).

SUPPLEMENTARY FIGURE 4: KAPLAN MEIER PLOTS OF PFS

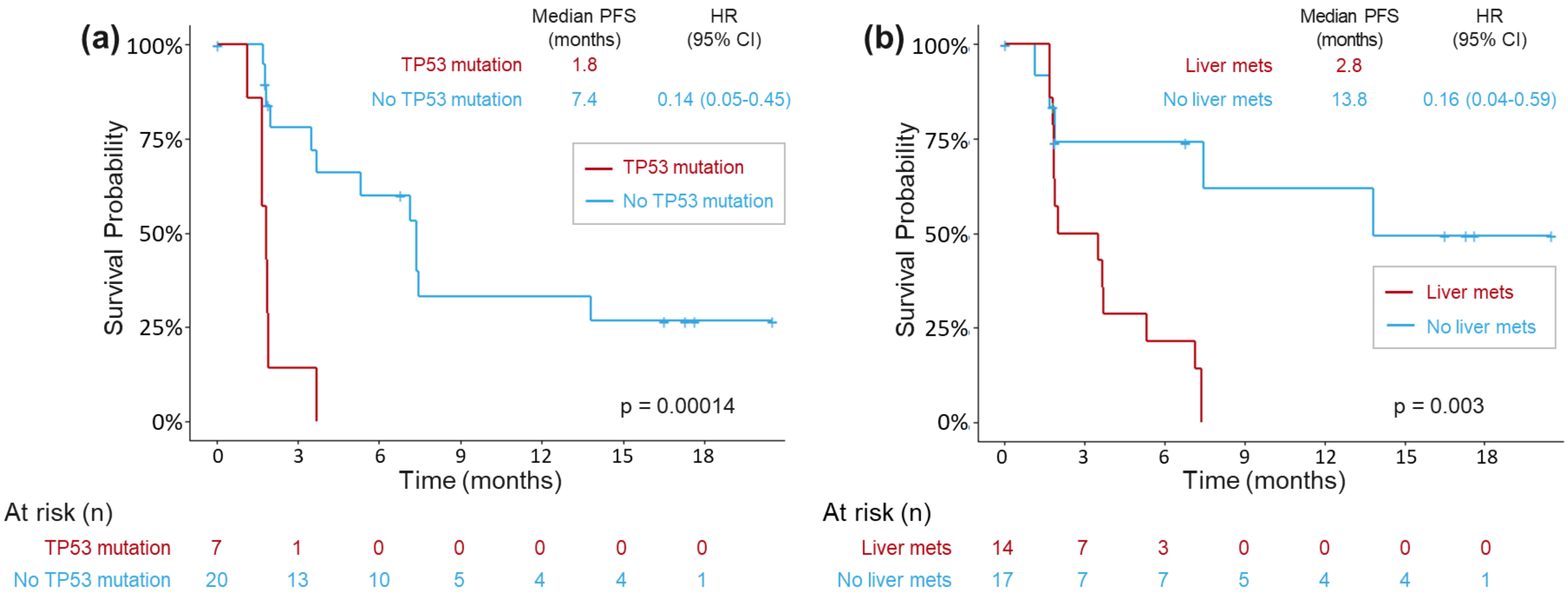

a). Progression-free survival by TP53 mutation status, b). Progression-free survival by presence or absence of liver metastases. Statistical significance was determined using a two-tailed signed log-rank test.
CI=confidence interval; HR=hazard ratio; PFS=progression-free survival

SUPPLEMENTARY FIGURE 5: pPolII LEVELS IN LYMPHOCYTES – MODULE 1A ALL PATIENTS WHO COMPLETED CYCLE 1

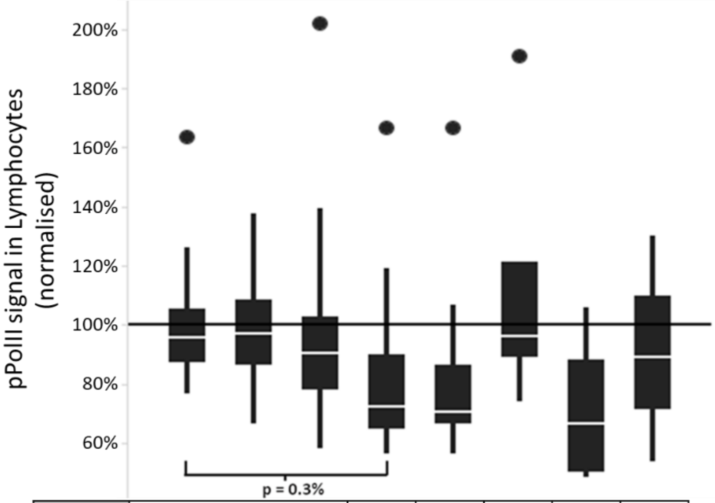

Cycle	1			2	3	4	5	6
Day	1	8	15	1	1	1	1	1
Patient n	25	23	17	18	8	4	4	3
Median	96%	97%	91%	72%	71%	96%	87%	89%

pPolII=phosphorylated RNA polymerase II
C1D1 data reflects the intra-patient variability.
Box plots show median, upper and lower quartiles and range; where data is greater than or less than 1.5 times the interquartile range relative to the upper or lower quartile, respectively, then it is shown as an outlier. Statistical comparison between pre-treatment samples (Cycle 1 Day 1) and subsequent timepoints were conducted using a 1-tailed Mann-Whitney test with Bonferroni correction.

SUPPLEMENTARY FIGURE 6: SINGLE DOSE PLASMA PHARMACOKINETICS (GEOMETRIC MEAN PLASMA PK CONCENTRATION PER TREATMENT GROUP – CYCLE 0 IN MODULE 1A, PK ANALYSIS POPULATION)

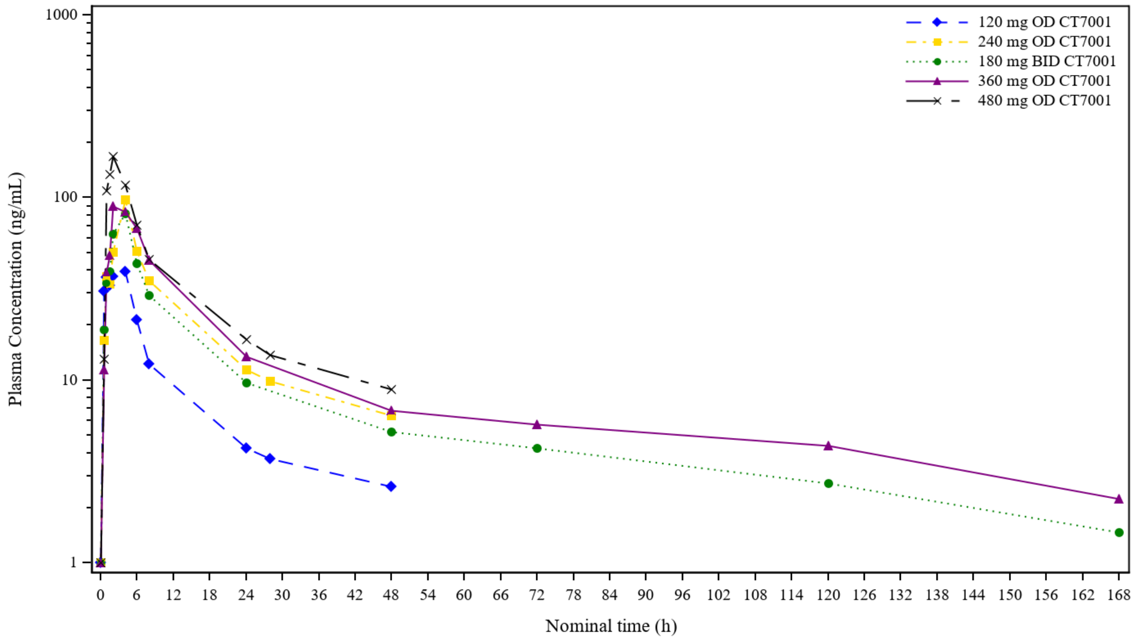

OD=once daily; BID=twice daily; LLOQ=lower limit of quantification; PK=pharmacokinetic.
Pre-dose timepoint where all concentrations are <LLOQ are plotted at the assay LLOQ.
Mean concentration only calculated where more than 2 participants had a quantifiable concentration at the timepoint.
N=33.

SUPPLEMENTARY FIGURE 7: MULTIPLE DOSE PLASMA PHARMACOKINETICS (MEAN PLASMA PK CONCENTRATIONS PER TREATMENT GROUP – CYCLE 2, DAY 1 IN MODULE 1A, PK ANALYSIS POPULATION)

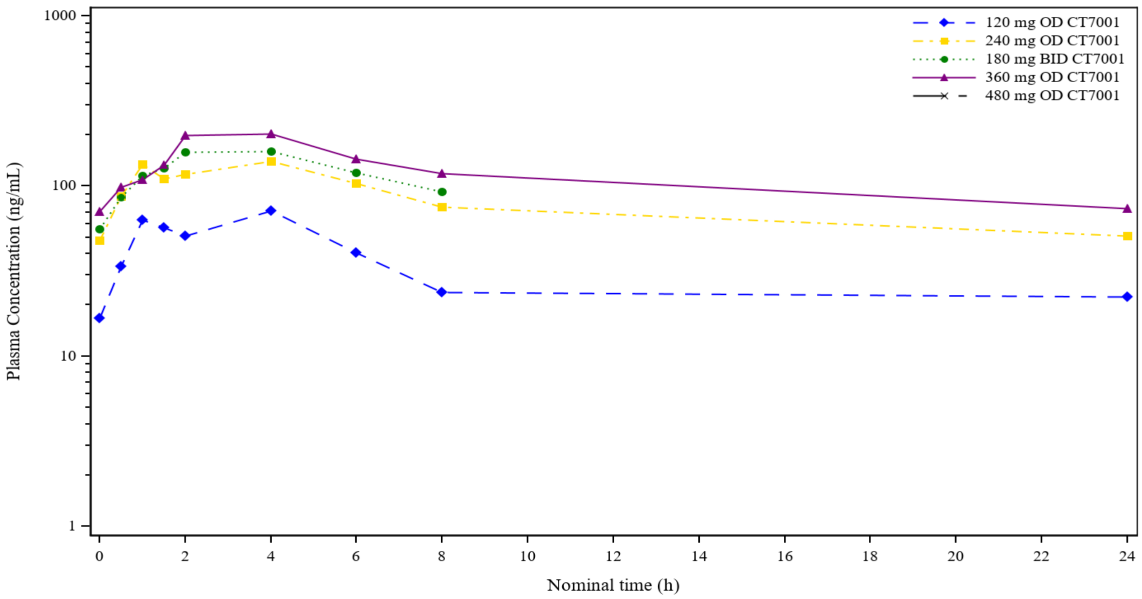

OD=once daily; BID=twice daily; LLOQ=lower limit of quantification; PK=pharmacokinetic
Pre-dose timepoint where all concentrations are <LLOQ are plotted at the assay LLOQ.
Mean concentration only calculated where more than 2 participants had a quantifiable concentration at the timepoint. The 480 mg OD CT7001 cohort is not presented since only 1 participant had quantifiable concentrations.
180 mg BID participants received a second dose at 12 hours.
N=26.

SUPPLEMENTARY FIGURE 8: MEAN TRHOUGH PLASMA PK CONCENTRATIONS PER TREATMENT GROUP (MODULE 1A, PK ANALYSIS POPULATION)

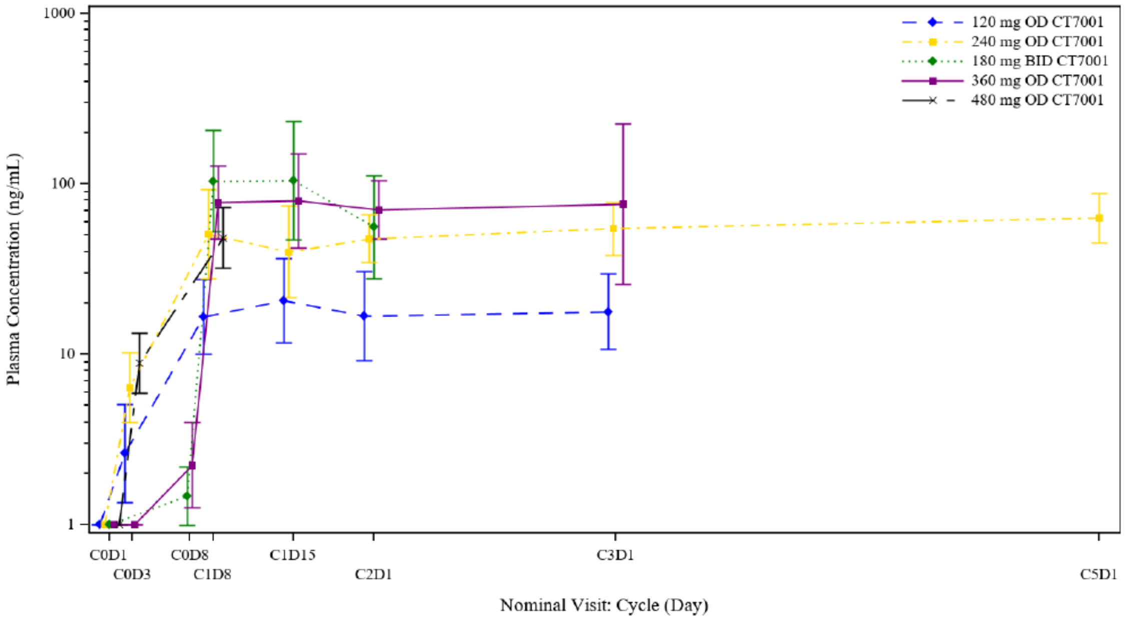

C=cycle; D=day; OD=once daily; BID=twice daily; LLOQ=lower limit of quantification; PK=pharmacokinetic.
Cycle 0 Day 1 pre-dose timepoint where all concentrations are <LLOQ are plotted at the assay LLOQ.
Error bars represent standard deviation range, either side of the geometric mean, calculated as geometric mean x/÷ geometric standard deviation. Mean concentration only calculated where more than 2 participants had a quantifiable concentration at the timepoint.
N=26.

SUPPLEMENTARY FIGURE 9: BOX PLOTS OF SAMURACICLIB TROUGH CONCENTRATIONS AFTER 360MG DAILY DOSING OF SAMURACICLIB ON C1D15 AND C2D1 FROM MODULE 1A AND MODULE 2B (COMBINATION WITH FULVESTRANT)

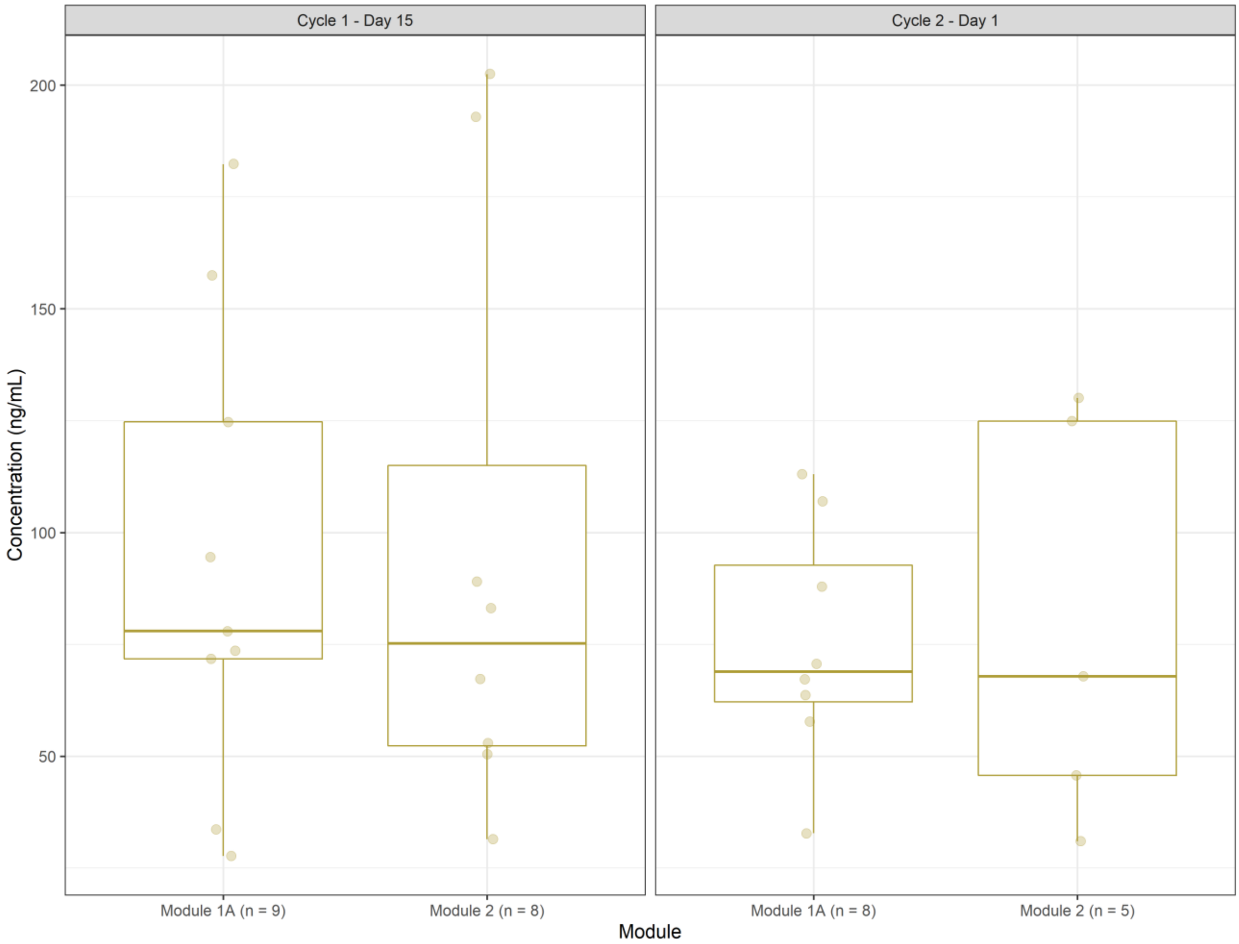

Boxplot: lower whisker is smallest observation greater than or equal to lower hinge - 1.5 * IQR, lower hinge is 25% quantile, middle is median, upper edge of notch is median + 1.58 * IQR/sqrt(n), upper hinge is 75% quantile, upper whisker is largest observation less than or equal to upper hinge + 1.5 * IQR. Points represent raw data.
Note: Cycle = 21 days in Module 1A and = 28 days in Module 2A

SUPPLEMENTARY FIGURE 10: BOX PLOTS OF FULVESTRANT TROUGH CONCENTRATIONS ON 500MG + LOADING DOSE REGIMEN OF FULVESTRANT AT C3D1 AND C5D1 IN MODULE 2A AND REPORTED TROUGH CONCENTRATIONS* FOR WESTERN PARTICIPANTS AS MONOTHERAPY

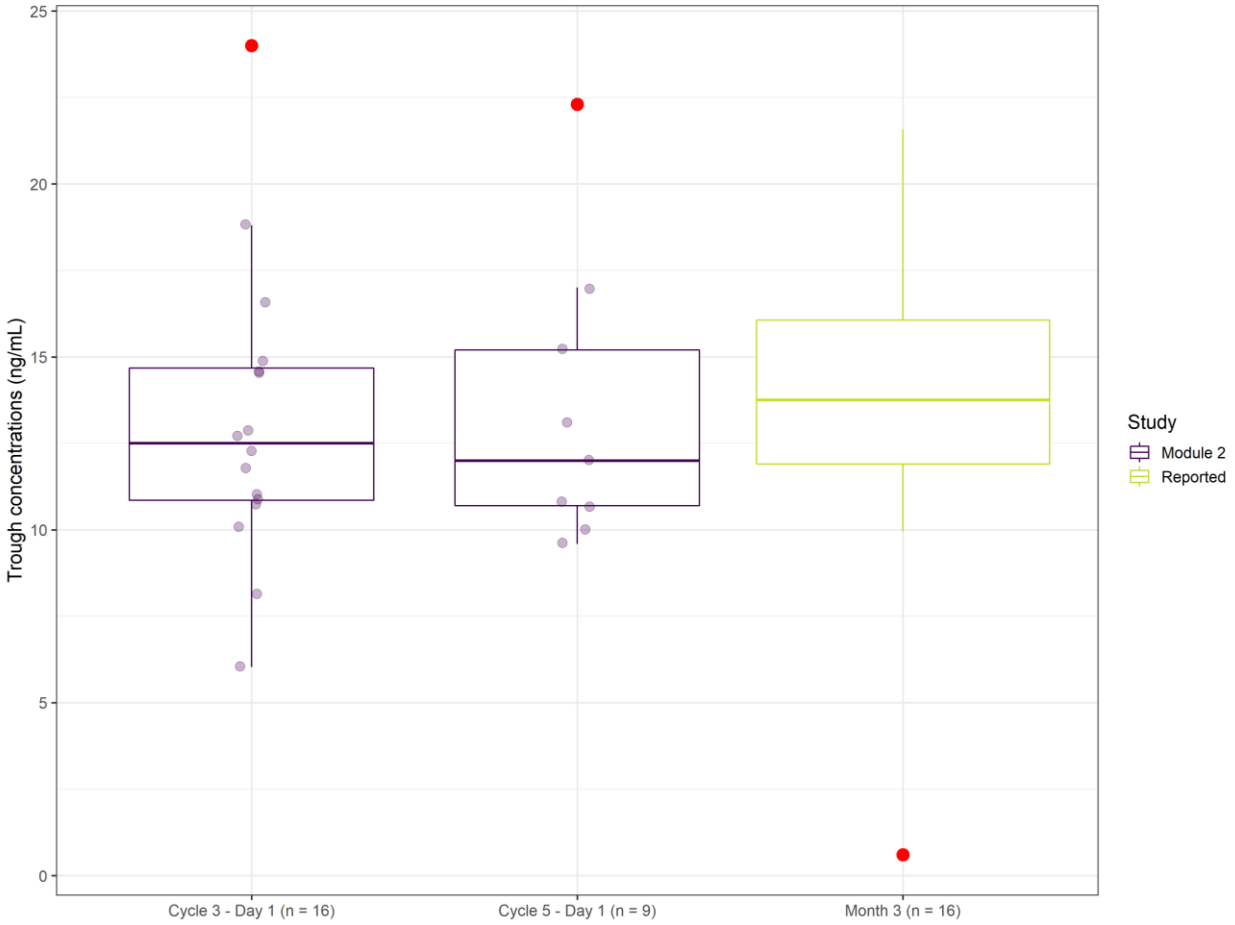

Boxplot: lower whisker is smallest observation greater than or equal to lower hinge - 1.5 * IQR, lower hinge is 25% quantile, middle is median, upper edge of notch is median + 1.58 * IQR / sqrt(n), upper hinge is 75% quantile, upper whisker is largest observation less than or equal to upper hinge + 1.5 * IQR. Points represent raw data, points highlighted in red are outliers

* Fulvestrant data from FINDER2 trials[S4]

SUPPLEMENTARY FIGURE 11: EXAMPLE FLOW CYTOMETRY GATING FOR LYMPHOCYTES FROM PATIENT WHITE BLOOD CELL PREPARATIONS

Lymphocytes were selected based on forward scatter (FSC) and side scatter (SSC). Typically lymphocytes represented 25-30% of the white blood cell preparations. Data in Supplementary Figure 5 are derived from the median fluorescence intensity of RNA pol II phosho-S5 for the lymphocyte gated population.

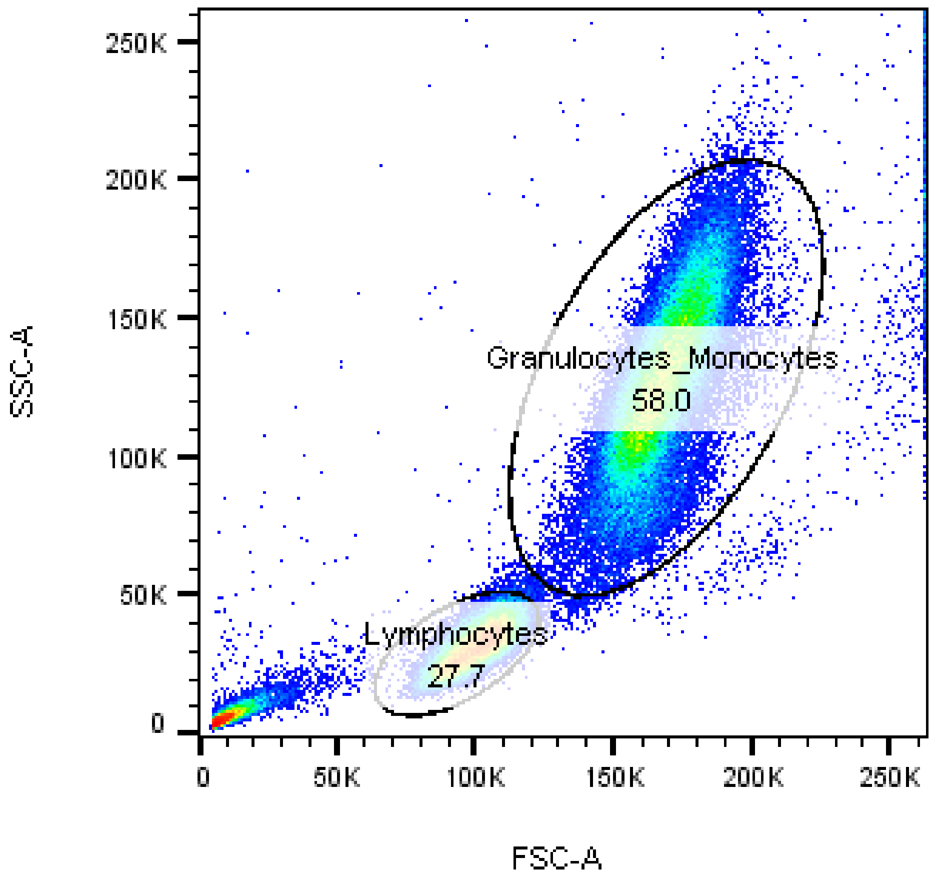

SUPPLEMENTARY TABLE 1: SCHEDULE OF EVENTS FOR MODULE 1A (DOSE ESCALATION)

Visit	Screen-ing	Cycle 0			Cycle 1 (21 days)			Cycle 2 (21 days)		Cycle 3 onward (21 days)	End of treat-ment	End of study
Timing of Visit	Day -28 to Day -1	Day 1[a] single dose	Day 2 washout	Day 3-6 PK	Day 1	Day 8<br>±1 day	Day 15<br>±1 day	Day 1<br>±1 day	Day 15<br>±3 days	Day 1<br>±3 days	Within 3 days after last dose	28 to 35 days after last dose
Informed consent	X											
Medical history[aa]	X											
Adverse events	X	X	X	X	X	X	X	X	X	X	X	X
Concomitant medications	X	X	X	X	X	X	X	X	X	X	X	X
Height[b]	X											
Weight	X	X			X	X	X	X	X	X	X	X
Physical examination	X	X			X	X	X	X	X	X	X	X
ECOG performance	X	X			X	X	X	X	X	X	X	X
Vital signs	X	X[c]	X		X[d]	X[e]	X[e]	X[d]		X[e]		
12-lead ECG	X	X[c]	X		X[d]	X[e]	X[e]	X[d]		X[e]		
Hematology (including reticulocyte count)	X	X[f]			X	X	X	X	X	X	X	
Serum chemistry and tumor markers	X	X[f]			X	X	X	X	X	X	X	
Coagulation (aPTT and INR)	X	X[f]			X	X	X	X	X	X	X	
Pregnancy test[g]<br>PK blood sample	X	X[f]<br>X[i]	X[i]	X	X[z]	X[j]	X[j]	X<br>X[k]		X<br>X[l]	X	
Blood sample for PBMCs biomarker	X	X[m]	X		X[n]	X[j]	X[j]	X[n]		X[p]	X	
Pharmacogenetics blood sample[q]		X[n]										
Plasma for ctDNA[q]	X[n]	X[n]						X[n]		X[n]	X	
Blood samples for exploratory research		X[n]						X[n]		X[n]		
Urinalysis	X	X[f]			X	X	X				X	
Fresh tumor biopsy[r]		X[s]						X[t]			X[u]	
Archival tissue (if available)	X											
Imaging (CT or MRI[v])	X									X[h]	X	
IMP dosing[w]		X			Dosing Period							
Overall survival/PFS[x]				X								

Abbreviations: aPTT=activated partial thromboplastin time; CT=computed tomography; ctDNA=circulating tumor deoxyribonucleic acid; ECG=electrocardiogram; ECOG=Eastern Cooperative Oncology Group; IMP=investigational medicinal product; INR=international normalized ratio; MRI=magnetic resonance imaging; PBMC=peripheral blood mononuclear cell; PFS=progression-free survival; PK=pharmacokinetic; SRC= Safety Review Committee.

Note: Each cycle=21 days. Cycle 3 onwards, Day 1 visits only

[a] All screening procedures must be completed and results available and reviewed before dosing on Cycle 0 Day 1.

[b] Height measured at Screening only.

[c] Supine blood pressure, pulse, temperature, respiratory rate, oxygen saturation measured pre-dose and at 0.5, 1, 2, 4, and 8 hours post-dose. ECGs measured pre-dose, 1, 2, 4, and 8 hours post-dose.

[d] Supine blood pressure, pulse, temperature, respiratory rate, oxygen saturation and ECGs measured pre-dose and 1,2, 4, and 8 hours post-dose.

[e] Measured pre-dose only.

[f] Laboratory tests did not need repeating if visit was within 3 days of Screening samples. All tests were collected pre-dose on all dosing days.

[g] For women of childbearing potential only. Urine or serum tests allowed.

[h] Scans taken at Cycles 3, 5, 7, etc. with a 7-day window permitted for the imaging.

[i] Collected pre-dose and at 0.5 (± 5 mins), 1, 1.5, 2, 4, 6, 8 (± 10 mins), 24 (± 60 mins), 48, and 72 (± 60 mins), 120 (± 24 hours if scheduled over a weekend) and 168 (± 60 mins) hours post-dose (which was to occur before the Cycle 1 Day 1 dose).
[j] Collected pre-dose on Day 8 and Day 15 (Cycle 1).
[k] Collected pre-dose and at 0.5, 1, 1.5, 2, 4, 6, 8, and 24 hours post-dose (before the Cycle 2 Day 2 dose)
[l] Collected pre-dose every other cycle. i.e., Day 1 of Cycle 3, 5 etc.
[m] Collected pre-dose, 24, and 48 hours post-dose (matching same time as 24- and 48-hour post-dose PK samples).
[n] Collected pre-dose.
[o] Deleted.
[p] Collected pre-dose on Day 1 from Cycle 3 and subsequent cycles.
[q] Only for participants who separately consented to genetic research.
[r] Only for participants who consented to collection of tumor biopsy samples (part of the main study consent form).
[s] Collected between Day -7 and pre-dose on Cycle 0 Day 1.
[t] Collected on Cycle 2 Day 1, the same day as PK sampling.
[u] Non-mandatory biopsy after confirmation of progression of disease (as soon as possible after disease progression confirmed).
[v] The imaging method (CT or MRI) used at Screening was to be used at each subsequent visit.
[w] Participants took their CT7001 dose in the clinic on the day when PK sampling was planned.
[x] Participants were followed up until disease progression. Data on survival was also checked.
[y] Collected between Day -7 and pre-dose on Cycle 1 Day 1.
[z] This was a Cycle 0 PK sample but noted in the table on Cycle 1 Day 1 for completeness.
[aa] Included information on prior anti-cancer treatments, alcohol consumption and tobacco use.
NB: The frequency of dosing, any washout period to assess PK, and dosing staggering interval between participants could change based upon emerging data as reviewed and agreed by the SRC without the requirement for a substantial amendment to the protocol. The frequency of PK sampling could be changed based on SRC review and could include up to 3 additional PK samples of 4 mL within any given cycle and taken by venipuncture, to better characterize the individual PK profile based upon emerging PK data.

SUPPLEMENTARY TABLE 2: SCHEDULE OF EVENTS FOR MODULE 1A (BIOPSY EXPANSION IN BREAST CANCER PATIENTS)

**Visit**	**Screening**	**Cycle 1 (21 days)**			**Cycle 2 (21 days)**		**Cycle 3 etc. (21 days)**	**End of Treatment**	**End of Study**
**Timing of Visit**	**Day -28 to Day -1[a]**	**D1**	**D8**	**D15**	**D1**	**D15**	**D1**	**Within 3 days after last CT7001 dose**	**28 to 35 days after last CT7001 dose**
			**±1 day**	**±1 day**	**±1 day**	**±3 days**	**±3 days**		
Informed consent	X								
Medical history[aa]	X								
Adverse events	X	X	X	X	X	X	X	X	X
Concomitant medications (including immunomodulators)	X	X	X	X	X	X	X	X	X
Height[b]	X								
Weight	X	X	X	X	X	X	X	X	X
Physical examination	X	X	X	X	X	X	X	X	X
ECOG performance status	X	X	X	X	X	X	X	X	X
Vital signs	X	X[d]	X[e]	X[e]	X[d]		X[e]		
Triplicate 12-lead ECGs	X	X[d]	X[e]	X[e]	X[d]		X[e]		
**Laboratory Samples - Mandatory**									
Hematology including reticulocyte count	X	X[f]	X	X	X	X	X	X	
Serum chemistry including blood borne tumor markers	X	X[f]	X	X	X	X	X	X	
Coagulation (aPTT and INR)	X	X[f]	X	X	X	X	X	X	
Pregnancy test[g]	X				X		X	X	
PK blood sample		X[j]	X[j]	X[j]	X[k]		X[l]		
Blood sample for PBMCs biomarker analysis (WBC)[n]	X	X			X		X	X	
Blood samples for exploratory research		X[n]			X[n]		X[n]		
Urinalysis	X	X	X	X				X	
Tumor biopsy expansion cohorts (mandatory paired biopsies)[t]	X[y]					X		X[u]	
Archival tissue sample (if available)	X								
**Laboratory Samples - Optional**									
Plasma preparation for ctDNA [n,q]	X	X			X		X	X	
Pharmacogenomics blood sample[n,q]		X							
Imaging (CT or MRI scan[v])	X						X[h]	X	
IMP administration[w]		**Dosing Period**							
Overall survival/ PFS[x]		X							

Abbreviations: aPTT=activated partial thromboplastin time; CT=computed tomography; ctDNA=circulating tumor deoxyribonucleic acid; ECG=electrocardiogram; ECOG=Eastern Cooperative Oncology Group; IMP=investigational medicinal product; INR=international normalized ratio; IXRS=interactive response system; MRI=magnetic resonance imaging; PBMC=peripheral blood mononuclear cell; PFS=progression-free survival; PK=pharmacokinetic; RNA=ribonucleic acid; SRC= Safety Review Committee; WBC=white blood cells.

Note: Each cycle=21 days. Cycle 3 onwards, Day 1 visits only.

[a] All screening procedures must be completed and results available and reviewed before dosing on Cycle 1 Day 1.

[b] Height measured at Screening only.

[d] Supine blood pressure, pulse, temperature, respiratory rate, oxygen saturation, and ECGs measured pre-dose and 1, 2, 4, and 8 hours post-dose.

[e] Measured pre-dose only.

[f] Laboratory tests did not need repeating if visit was within 3 days of Screening samples. All tests were collected pre-dose on all dosing days.

[g] For women of childbearing potential only. Urine or serum tests allowed.
[h] Scans taken at Cycles 3, 5, 7, etc. with a 7-day window permitted for the imaging.
[j] Collected pre-dose on Day 1, Day 8, and Day 15 (Cycle 1).
[k] Collected pre-dose and at 0.5, 1, 1.5, 2, 4, 6, 8, and 24 hours post-dose (before the Cycle 2 Day 2 dose).
[l] Collected pre-dose every other cycle. i.e., Day 1 of Cycle 3, 5 etc.
[n] Collected pre-dose.
[q] Only for participants who separately consented to genetic research.
[t] Tumor Biopsies: A biopsy of a readily accessible tumor lesion was obtained within 7 days before first dosing, on Day 15 ± 2 days of Cycle 2 and, if feasible, within 2 weeks after disease progression. First priority was given to formalin-fixed material for immunohistochemistry (including but not limited to CDK7, pPolII, c-Myc, pCDK1 and Ki67/Caspase). Where a second biopsy core was taken, it was used for RNA ChIP sequencing.
[u] Non-mandatory biopsy after confirmation of progression of disease (as soon as possible after disease progression confirmed).
[v] The imaging method (CT or MRI) used at Screening was to be used at each subsequent visit.
[w] CT7001 was dispensed by an IXRS system. Participants took their CT7001 dose in the clinic on the day when PK sampling was planned.
[x] Participants were followed up until disease progression. Data on survival was also checked.
[y] Collected between Day -7 and pre-dose on Cycle 1 Day 1.
[aa] Included information on prior anti-cancer treatments, alcohol consumption and tobacco use.
NB: The frequency of dosing, any washout period to assess PK, and dosing staggering interval between participants could change based upon emerging data as reviewed and agreed by the SRC without the requirement for a substantial amendment to the protocol. The frequency of PK sampling could be changed based on SRC review and could include up to 3 additional PK samples of 4 mL within any given cycle and taken by venipuncture, to better characterize the individual PK profile based upon emerging PK data.

SUPPLEMENTARY TABLE 3: SCHEDULE OF EVENTS

Mandatory Procedures

The investigator may schedule visits (unplanned visits) in addition to those listed on the schedule of activities to conduct evaluations or assessments required to protect the wellbeing of the patient.

**Protocol Activity**	**Screening**	**Active Treatment Phase [a] - One Cycle = 21 days**				**End of Treatment/ Withdrawal [c]**	**Post-Treatment Follow-Up [d]**
		**Cycles 1 and 2**			**Cycles ≥3 [v]**		
**Study Day**	**Within 28 days prior to treatment assignment unless specified otherwise**	**Day 1 [b]**	**Day 8**	**Day 15**	**Day 1**		
**Visit Window**		**±2 days**	**±2 days**	**±2 days**	**±3 days**	**Within 28 days**	
Informed Consent [e]	X						
Medical/Oncological History [f]	X						
Signs/Symptoms [g]		X	X	X	X	X	
Physical Examination/Vital Signs [h]	X	X	X	X	X	X	
ECOG Performance Status	X	X	X	X	X	X	
Laboratory Studies							
Haematology [i]	X	X	X	X	X	X	
Serum chemistry [j]	X	X	X	X	X	X	
Pregnancy test, serum oestradiol and FSH (if applicable) [k]	X						
Urinalysis [l]	X	X	X	X	X	X	
Triplicate 12-Lead ECGs [m]	X	X		X	X	X	
Tumour Assessments							
CT/MRI Scans and clinical evaluation of superficial disease [n]	X	Performed/repeated as described in footnote				X	X
Radionuclide Bone Scan, Whole Body [o]	X	Performed/repeated as described in footnote				X	X
Other Clinical Assessments							
Adverse Event Reporting [p]	X	Performed as described in footnote					
Concomitant Medications/Treatments	Recorded from 28 days prior to the start of study treatment up to 28 days after the last dose of study treatment						
Pharmacokinetics [q]		X	X[r]		X	X	
CYP2D6 Polymorphisms [s]		X					
IP Diary [t]		X			X		
Study Treatment							
CT7001 [u]		Daily Dosing					

a. **Active Treatment Phase**: Assessments should be performed prior to dosing on the visit day unless otherwise indicated. One cycle consists of 21 days. A cycle could be longer than 21 days if persistent toxicity delays initiation of the subsequent cycle. Day 1 of any cycle visit should coincide with the day the CT7001 treatment begins. If there are delays due to toxicity, then the start of the next cycle visit will be delayed until the patient has recovered and can begin study treatment again. The active treatment phase is ongoing as long as the patient is receiving CT7001.

b. **Serum Chemistry, Haematology, Physical Examination and ECG** not required if performed as part of screening within 7 days prior to treatment assignment.

c. **End of Treatment/Withdrawal**: Visit to be performed as soon as possible but no later than 4 weeks from the last dose of investigational products and prior to initiation of any new anticancer therapy. Obtain assessments if not completed during the previous 4 weeks on study (or within the previous 8 weeks or 12 weeks [as applicable] for disease assessments).

d. **Post Treatment Follow-up**: Patients who discontinue study treatment should be contacted 28 calendar days (±7 days) after discontinuation of study treatment) to assess if there have been any new adverse events and/or any change to any previously reported adverse events. Telephone contact is acceptable. Patients who discontinue active study treatment for any reason other than objective disease progression or death will continue to have tumour assessments performed every 8 weeks (±7days) for the first year, and then every 12 weeks (±7 days) in subsequent years (calculated from Cycle 1 Day 1) until documented progression, death or onset of new anticancer therapy, whichever occurs first.

e. **Informed Consent**: Informed consent must be obtained prior to any protocol required assessments being performed (with the exception of certain imaging assessments if meeting the criteria defined in Section 7.1 (Screening).

f. **Medical/Oncological History**: To include information on prior anticancer treatments, alcohol consumption and tobacco use.

g. **Baseline Signs and Symptoms** (tumour-related or otherwise) will be recorded at the Cycle1 Day1 visit prior to initiating treatment and then reported as adverse events during the trial if they worsen in severity or increase in frequency.

h. **Physical Examination/Vital Signs**: Includes an examination of all major body systems and breasts, height (at screening only), weight, supine blood pressure, pulse rate, respiratory rate and body temperature. May be performed by a physician, registered nurse or other qualified health care provider.

i. **Haematology** includes red blood cell count, haematocrit, mean cell volume, reticulocyte count (absolute particle count or relative particle count), white blood cell count with differential (absolute and percent counts of neutrophils, lymphocytes, monocytes, basophils, eosinophils) and platelet count. Additional haematology tests may be performed if clinically indicated.

j. **Serum Chemistry** includes HbA1c, ALT, AST, gamma glutamyl transferase (IU/L), alkaline phosphatase, bilirubin (total), creatine kinase, total protein, albumin, creatinine, urea nitrogen or urea, calcium (total), glucose, sodium, potassium, magnesium, chloride and phosphate. Additional serum chemistry tests may be performed as clinically indicated, including tumour markers (in accordance with local practice).

k. **Pregnancy Test** (serum or urine) at screening only for women of childbearing potential within 7 days of first dose of CT7001. (Tests may be repeated during the active treatment phase or later if so required by IRB/IECs or by local regulations.) Serum oestradiol and follicle stimulating hormone (FSH) levels are analysed at screening to confirm postmenopausal status of women <60 years old and not amenorrhoeic for at least 12 consecutive months with no alternative pathological or physiological cause.

l. **Urinalysis** includes visual examination and a dipstick test (including blood, glucose and protein). If either is abnormal, a microscopic examination should be performed as well.

m. **Triplicate ECGs** to be taken within 3-5 minutes of each other.

n. **CT or MRI Scans**: The same imaging method (CT or MRI) as used at Screening must be used at each subsequent disease assessment. Scans are performed every 8 weeks ((±7 days) for the first year and then every 12 weeks ((±7 days) from Cycle 1 Day 1 until disease progression, death, discontinuation from study participation (e.g., patient's request, lost to follow up) or start of subsequent cancer treatment, whichever occurs first.

   Clinical Evaluation of Superficial Disease should be performed during active treatment phase on Day 1 of every treatment Cycle (±2 days in first 2 Cycles and ±3 days in subsequent Cycles), at the end of treatment/withdrawal visit and during follow up at same intervals as described for CT or MRI scans.

o. **Radionuclide Bone Scans, Whole Body** should be performed at Screening. If at Screening bone lesions were identified, scans will be repeated during the active treatment phase and during follow-up visits every 16 weeks (± 1 week) from Cycle 1 Day 1, and at the time of confirmation of CR. If no bone lesions were identified at Screening, bone scans will only be performed when clinically indicated (i.e., patient describes new or worsening bone pain or has increasing alkaline phosphatase level or other signs and symptoms of new/progressing bone metastases) but are required at the time of confirmation of CR. New abnormalities found on subsequent bone scans must be confirmed by X-ray, CT scan with bone windows or MRI.

p. **Adverse Event Reporting**: Serious Adverse Events (SAEs) must be reported from the time the patient provides informed consent through and including 28 calendar days after the last administration of the study drug. SAEs occurring after the active reporting period has ended should be reported if the investigator becomes aware of them. All SAEs that the investigator believes have at least a reasonable possibility of being related to the study drugs are to be reported to the Sponsor. All AEs (serious and non-serious) should be recorded on the eCRF from the first dose of study treatment through last patient visit. It is expected that telephone contact with the patient will be made to assess SAEs and AEs 28 calendar days (±7 days) after the last administration of the study drug.

q. **Pharmacokinetics (PK)**: PK blood samples for through concentrations of CT7001 should be collected pre-dose on Day 1 of all Cycles, Day 8 of Cycle 1 and at the end of treatment/withdrawal visit.

r. **PK Blood Sample on Day 8** needs to be collected only in Cycle 1.

s. **CYP2D6 Polymorphisms**: A blood sample should be collected pre-dose on Day 1 of Cycle 1 for genotyping of CYP2D6 allelic variants and copy number change. As CYP2D6 is the main P450 enzyme involved in hepatic Phase I metabolism of CT7001, this is considered a mandatory study procedure.

t. **IP Diary**: Patients will be asked to complete a diary to document their investigational product intake.

u. **CT7001**: CT7001 will be dispensed by an IXRS system. Patients will be required to return all bottles of CT7001 as well as the completed patient diary on Day1 of each cycle for drug accountability.

v. Following 12 months of treatment, from Cycle 18 onwards, at the discretion of the investigator and patient, even numbered study visits may be omitted. Odd numbered study visits must be performed every 42 days +/- 3 days.

Optional Procedures

Protocol Activity		Cycle 1	Cycle 2		Cycles ≥3	Disease Progression
**Study Day**	**Within 28 days prior to treatment assignment unless specified otherwise**	**Day 1**	**Day 1**	**Day 15**	**Day 1**	
**Visit Window**		**-1 day**	**-1 day**	**±2 days**	**-3 days**	**+14 days**
Informed Consent [a]	X					
Blood Samples for ctDNA [b]	X	X	X		Pre-dose (every odd Cycle from C3)	X
Blood Samples for RNA Sequence Analysis [c]		X[c]	X			
Blood Samples for Exploratory Research [b]		X	X		Pre-dose (every odd Cycle from C3)	X
Blood Sample for Pharmacogenomics [d]		X				
Tumour Biopsies [e]	X			X		X
Archival Tumour Tissue [f]		X				

a. **Informed Consent**: All optional procedures require specific informed consent and this separately for: (1) ctDNA and WBC isolation (2) RNA-seq analysis, (3) exploratory research; (4) pharmacogenomics; (5) tumour biopsies; (6) collection of archival tumour tissue.

b. **Blood samples for ctDNA and for potential future exploratory research** should be collected at Screening (ctDNA only) and prior to dosing of CT7001 on Day 1 of Cycles 1, 2 and 3 and Day 1 in every subsequent odd cycle and, if feasible, within 14 days after documented disease progression.

c. **For RNA-Sequence analysis**, Predose samples should be taken on Day 1 of Cycles 1 and 2 with a second blood sample to be collected on Day 1 of Cycle 1 approximately 4 hours after dosing. Blood Samples for RNA-Sequence to be collected from patients ONLY who have consented to fresh tumour biopsies.

d. **A Blood Sample for Potential Future Pharmacogenomics Analysis** should be taken prior to dosing on Day 1 of Cycle 1. Due to its importance in CT7001 metabolism, analysis of CYP2D6 gene variations (polymorphisms and/or copy number change) is part of the mandatory procedures in this study.

e. **Tumour Biopsies**: A biopsy of a readily accessible tumour lesion should be obtained within 10 days before first dosing, on Day 15 ± 2 days of Cycle 2 and, if feasible, within two weeks after disease progression. First priority is given to formalin-fixed material for immunohistochemistry (including but not limited to CDK7, pPolII, c-Myc, pCDK1 and Ki67/Caspase). Where a second biopsy core is taken, it will be used for RNA ChIP Sequencing.

f. **Archival Tumour Tissue**: Even in case a fresh tumour biopsy can be obtained, an archival formalin-fixed paraffin-embedded tumour tissue sample may be requested.

SUPPLEMENTARY TABLE 4: SCHEDULE OF EVENTS FOR MODULE 2A

Protocol Activity	Screening [a]	Active Treatment Phase [b] - One Cycle = 28 days			End of Treatment/ Withdrawal [d, cc]	Post-Treatment Follow-Up [e]
		Cycles 1 and 2		Cycles ≥3 [dd]		
**Study Day**	**Within 28 days prior to allocation to study therapy unless specified otherwise**	**Day 1 [c]**	**Day 15**	**Day 1**		
**Visit Window**		**±2 days**	**±2 days**	**±7 days**	**Within 28 days**	
Informed Consent [f]	X					
Medical/Oncological History [g]	X					
Signs/Symptoms [h, cc]	X	Pre-dose	Pre-dose	Pre-dose	X	
Physical Examination/Vital Signs [i, cc]	X	Pre-dose	Pre-dose	Pre-dose	X	
ECOG Performance Status	X	Pre-dose	Pre-dose	Pre-dose	X	
Laboratory Studies						
Haematology [j]	X	Pre-dose	Pre-dose	Pre-dose	X	
Serum Chemistry [k]	X	Pre-dose	Pre-dose	Pre-dose	X	
Fasted Glucose [ee]		Pre-dose	Pre-dose	Pre-dose	Pre-dose	
Pregnancy Test, Serum Oestradiol and FSH (if applicable) [l]	X					
Urinalysis [m]	X	Pre-dose	Pre-dose	Pre-dose	X	
Triplicate 12-Lead ECGs [cc]	X	Pre-dose		Pre-dose	X	
Tumour Assessments						
CT/MRI Scans and Clinical Evaluation of Superficial Disease [n, cc]	X	Performed/repeated as described in footnote [n]			X	X
Radionuclide Bone Scan, Whole Body [o, cc]	X	Performed/repeated as described in footnote [o]			X	X
Other Clinical Assessments						
Adverse Event Reporting [p]	Recorded/reported from 28 days prior to the start of study treatment up to 28 days after the last dose of study treatment					
Concomitant Medications/Treatments						
Patient Feedback [q]					X	
Pharmacokinetics [r, cc]		Pre-dose [r]	Pre-dose [r] (C1 only)	Pre-dose (every other Cycle)	X [r]	
CYP2D6 Polymorphisms [s]		Pre-dose (C1 only)				
IP Diary [t, cc]		X	X	X		
Study Treatment						
Randomisation (Part B) [ff]		X				

Protocol Activity	Screening	Cycle 1	Cycle 1	Cycle 2	Cycles ≥3	End of Treatment/ Withdrawal	Post-Treatment Follow-Up
CT7001 (Parts A, B, C) or Placebo (Part B) [u, cc]		Daily Dosing					
Fulvestrant (Parts A, B and C) [v]		X	X (C1 only)	X			
		**Active Treatment Phase - One Cycle = 28 days**					
**Study Day**	**Within 28 days prior to allocation to study therapy unless specified otherwise**	**Day 1**	**Day 15**	**Day 1**	**Day 1**		
**Visit Window**		**Within 48 hours before first drug administration**	**Before drug administration**	**Before drug administration**	**Before drug administration**		
Optional Procedures and Assessments [w]							
Informed Consent [w]	X						
ctDNA [x, cc]	X	Pre-dose		Pre-dose	Pre-dose (every other Cycle)	X (within 2 weeks after disease progression)	
Pharmacogenomics [y]		Pre-dose					
Exploratory Research [z, cc]		Pre-dose		Pre-dose	Pre-dose (every other Cycle)	X (within 2 weeks after disease progression)	
Tumour Biopsy [aa, cc]	X (within 10 days prior to allocation to study therapy)			X (Cycle 2 Day 15 ± 7 days)		X (within 2 weeks after disease progression)	
Archival Tumour Material [bb]		To be collected at any time during active treatment phase					

a. **Screening:**

- Prior to any protocol required assessments being performed informed consent must be obtained from the patient.
- In case tumor assessments by CT or MRI were performed as part of routine procedures before the signing of the informed consent but within 28 days prior to allocation to study therapy, those assessments do not need to be repeated and can be used as baseline assessments as long as:
  - The tests were performed per the method requirements described in Section 8.1 and in footnote n.
  - Proper documentation is available in the patient's source notes that the radiographic procedures were performed as part of standard of care.
- Radionuclide bone scans performed as routine procedure within 12 weeks prior to allocation to study therapy are also accepted as baseline assessment if they meet the two requirements described above.

b. **Active Treatment Phase:**

- The active treatment phase is ongoing as long as the patient is receiving both study drugs (i.e., CT7001/placebo and fulvestrant).
- Assessments should be performed prior to dosing on the visit day unless otherwise indicated.
- A treatment cycle consists of 28 days but could be longer if persistent toxicity delays initiation of the subsequent cycle. In that case, Day 1 assessments of the subsequent cycle will be performed when study treatment is resumed, coinciding with the day the CT7001/placebo treatment begins.
- Day 1 procedures which were performed prior to knowing that the start of the cycle needs delay do not need to be repeated if:
  - Not required to determine whether toxicity has sufficiently resolved to resume study therapy.
  - Performed within 7 days prior to restart of study therapy.

- Fulvestrant injections will be given every 28 days (±7 days) except Cycle 1 during which it will be administered on Days 1 and 15 (±2 days). In case toxicity requires delay of the subsequent cycle, fulvestrant injections will be postponed accordingly.

c. **Serum chemistry, hematology, physical examination and ECG** are not required on Day 1 of Cycle 1 if performed as part of screening within 7 days prior to allocation to study therapy.

d. **End of Treatment/Withdrawal**: Visit and required tests to be performed as soon as possible but no later than 28 days from the last dose of investigational products on study and prior to initiation of any new anticancer therapy.
  - In the event study therapy was discontinued due to death or documented disease progression, the end of treatment visit represents the final visit.
  - Obtain tumor assessment by:
    - CT or MRI, as applicable, unless performed within the previous 8 weeks.
    - Bone scan unless performed within the previous 12 weeks.
  - Patients who continue on-study Fulvestrant monotherapy: The end of treatment schedule of events should be completed at the time of CT7001 discontinuation or prior to the administration of next dose of fulvestrant. The modified end of treatment visit will be completed at the discontinuation of Fulvestrant.
  - For patients who continue to receive clinical benefit despite the individual patient or the module meeting the primary endpoint, then at the discretion of the Investigator, with approval of the Sponsor, and in compliance with national regulations the Investigator may initiate completion of study participation and apply for Managed Access of Investigational Product.

e. **Post Treatment Follow-up**: Patients who discontinue study treatment for any reason other than objective disease progression or death will continue to have tumor assessments performed by CT or MRI, as applicable, and clinical evaluation in case of superficial disease every 8 weeks (±7days) for the first year, and then every 12 weeks (±7 days) in subsequent years (calculated from the date of cycle 1 day 1) until documented progression, death, onset of new anticancer therapy (Part A & C Only) or Module 2 LPLV, whichever occurs first. For patients enrolled into Part B, patients will continue to have tumor assessments and clinical evaluation of superficial disease until documented progression, death or Module 2 LPLV, whichever occurs first, irrespective of initiating a new anti-cancer therapy.
  - With regard to bone scans, please refer to footnote o.

f. **Informed Consent**: Informed consent must be obtained prior to any protocol required assessments being performed (with the exception of certain imaging assessments if meeting the criteria defined in Section 7.1 (Screening) and footnote a.

g. **Medical/Oncological History**: To include information on prior anticancer treatments, alcohol consumption and tobacco use.

h. **Signs and Symptoms** (tumor-related or otherwise) at baseline will be recorded at the Cycle1 Day1 visit prior to initiating treatment and then reported as adverse events during the trial if they worsen in severity or increase in frequency.

i. **Physical Examination/Vital Signs**: Includes an examination of all major body systems and breasts, height (at screening only), weight, supine blood pressure, pulse rate, respiratory rate and body temperature. May be performed by a physician, registered nurse or other qualified health care provider.

j. **Hematology** includes hemoglobin, WBC, absolute neutrophil count, platelet count and reticulocytes. Additional hematology tests may be performed if clinically indicated. For Module 2 B/C mean platelet volume will also be performed.

k. **Serum Chemistry** includes HbA1c, AST, ALT, alkaline phosphatase, creatine kinase, glucose, sodium, potassium, magnesium, total calcium, total bilirubin, blood urea nitrogen (BUN), serum creatinine and albumin. Additional serum chemistries tests may be performed as clinically indicated, including tumor markers (in accordance with local practice).

l. **Pregnancy Test** (serum) at screening, within 7 days prior to first dose of CT7001 or Placebo, only for women of childbearing potential. (Tests may be repeated during the active treatment phase or later if so required by IRB/IECs or by local regulations.)
  - Serum estradiol and follicle stimulating hormone (FSH) levels are analyzed at screening to confirm postmenopausal status of women <50 years old or <60 years old and not amenorrhoeic for at least 12 consecutive months with no alternative pathological or physiological cause.

m. **Urinalysis** includes visual examination and a dipstick test for blood, glucose and protein. If either is abnormal, a microscopic examination should be performed as well.

n. **CT or MRI Scans**: Please refer to Section 8.1 for instructions regarding use of CT scan or MRI for tumor assessment in chest versus abdomen, pelvis and other areas.
  - The same imaging method (CT or MRI) as used at Screening must be used at each subsequent disease assessment.
  - Scans are performed at screening (see also footnote a), every 8 weeks (±7 days) for the first year and then every 12 weeks (±7 days) in subsequent years, from Cycle 1 Day 1 until disease progression, death, discontinuation from study participation (e.g., patient's request, lost to follow up) or start of subsequent cancer treatment, whichever occurs first.
  - **Clinical Evaluation of Superficial Disease** should include photographs of all superficial tumor lesions and should be performed at same intervals as described for CT or MRI scans and timing of clinical evaluations should coincide with the scans (+/-7 days).

o. **Radionuclide Bone Scans, Whole Body** should be performed at Screening. See also footnote a.

- If at Screening bone lesions were identified, scans will be repeated during the active treatment phase and during follow-up visits every 16 weeks (± 7 days) from Cycle 1 Day 1, and at the time of confirmation of CR.
- If no bone lesions were identified at Screening, bone scans will only be performed when clinically indicated (i.e., patient describes new or worsening bone pain or has increasing alkaline phosphatase level or other signs and symptoms of new/progressing bone metastases) but are required at the time of confirmation of CR. New abnormalities found on subsequent bone scans must be confirmed by CT scan with bone windows or MRI.

p. **Adverse Event Reporting**: Serious Adverse Events (SAEs) must be reported from the time the patient provides informed consent through and including 28 calendar days after the last administration of the study drug. SAEs occurring after the active reporting period has ended should be reported if the investigator becomes aware of them. All SAEs that the investigator believes have at least a reasonable possibility of being related to the study drugs are to be reported to the Sponsor. All AEs (serious and non-serious) should be recorded on the eCRF from the first dose of study treatment through last patient visit. It is expected that telephone contact with the patient will be made to assess SAEs and AEs 28 calendar days (±7 days) after the last administration of the study drug.

q. **Patient Feedback**: All patients at the time of discontinuing from the study will be given the opportunity to complete a brief questionnaire to provide feedback relating to their trial participation. Completion of this questionnaire is optional. A sub-set of patients who express interest may be given the opportunity to participate in a face-to-face interview.

r. **Pharmacokinetics (PK)**:

- Blood samples for trough concentrations of CT7001/placebo will be collected before dosing on Day 1 and 15 of Cycles 1, Day 1 in Cycles 2 and 3, Day 1 in every subsequent odd Cycle and at the end of treatment visit.
- Blood samples for trough concentrations of fulvestrant will be collected before injection of fulvestrant on Days 1 and Day 15 of Cycle 1, Day 1 in Cycles 2 and 3, Day 1 in every subsequent odd Cycle and at the end of treatment visit.

s. **CYP2D6 Polymorphisms**: A blood sample should be collected pre-dose on Day 1 of Cycle 1 for genotyping of CYP2D6 allelic variants and copy number change. As CYP2D6 is the main P450 enzyme involved in hepatic Phase I metabolism of CT7001, this is considered a mandatory study procedure.

t. **IP Diary**: Part B - Patients will be asked to complete a diary to document their investigational product intake.

u. **CT7001 or Placebo**: CT7001/Placebo will be dispensed by an IXRS system. Patients will be required to return all bottles of CT7001/placebo as well as the completed patient diary on Day1 of each cycle for drug accountability.

v. **Fulvestrant**: Fulvestrant will be dispensed by an IXRS system. To be administered on-site as two consecutive slow intramuscular (IM) injections (1-2 minutes) of 250 mg in 5 mL, one in each buttock (gluteal area). Fulvestrant will be dosed at 500 mg given at intervals of 28 ± 2 days, with an additional 500 mg given 14 ± 2 days after the first dose.

w. **Optional Procedures and Assessments** require specific **Informed Consent**, and this separately for (1) ctDNA, WBC isolation, (2) retained sample for exploratory research, (3) tumor biopsies, (4) archival tumor material, and (5) retention of remaining material from the blood sample collected and prepared for genotyping of CYP and drug transporter genes and potential use for additional future pharmacogenomic research .

x. **ctDNA**: A blood sample for ctDNA should be collected at screening, pre-dose on Day 1 of Cycles 1, 2 and 3, Day 1 in every other subsequent odd Cycle and within 2 weeks after disease progression.

y. **Pharmacogenomics**: A blood sample for pharmacogenomic analysis should be collected pre-dose on Day 1 Cycle 1.

z. **Exploratory Research:** A blood sample for potential future exploratory research should be collected pre-dose on Day 1 of Cycles 1, 2 and 3, Day 1 in every other subsequent odd Cycle and within 2 weeks after disease progression.

aa. **Tumour Biopsy**: In patients with readily accessible lesions who have provided consent, a biopsy should be obtained within 10 days before allocation to study therapy and as much as feasible on Day 15 ± 7 days of Cycle 2 and within 2 weeks after disease progression. Priority is given to formalin-fixed material for immunohistochemistry (including but not limited to CDK7, pPolII, c-Myc, pCDK1 and Ki67/Caspase). In case the biopsy is of sufficient size, remaining material should be fresh-frozen for RNA signature and ChIP-Seq analyses.

bb. **Archival Tumour Material**: Even in case a fresh tumor biopsy can be obtained, an archival formalin-fixed paraffin-embedded tumor tissue sample will be requested (see also Section 8.5.4). It can be collected any time during the active treatment phase but ideally should be obtained during the first 2 cycles.

cc. **Fulvestrant Monotherapy**: These procedures may be omitted for patients who have Sponsor Approval to continue on-study receiving monotherapy Fulvestrant.

dd. Following 12 months of treatment, from Cycle 12 onwards, at the discretion of the investigator and patient, even numbered study visits may be omitted with the exception of fulvestrant administration. Odd numbered study visits must be performed every 56 days +/- 7 days. Where local regulations and procedures allow, Fulvestrant may be administered by a qualified individual at the patients home or by their primary care physician.

ee. **Fasted Glucose**: For patients entering the study with a medical history of diabetes, a fasted glucose test will be required pre-dose at C1D1 and C1D15 (mandatory) and at the remaining cycles at the discretion of the investigator.

ff. **Randomization (Part B):** Randomization may be performed up to 48 hours prior to the planned C1D1 IMP administration.

SUPPLEMENTARY TABLE 5: DOSE LIMITING TOXICITIES – MODULE 1A DOSE ESCALATION COHORT (SAFETY ANALYSIS POPULATION)

**System Organ Class**<br>**MedDRA preferred term**<br>**Number of patients (%)**	**Cohort 1 120mg OD N=6**	**Cohort 2 240mg OD N=7[b]**	**Cohort 4 360mg OD N=6**	**Cohort 3 480mg OD N=6**	**Cohort 5 180mg BID N=8**	**Total N=33**
Patients with any DLT	0	0	0	4 (66.7)	2 (25.0)	6 (18.2)
Vomiting	0	0	0	2 (33.3) [a]	0	2 (6.1)
Diarrhoea	0	0	0	1 (16.7) [a]	0	1 (3.0)
Gastroesophageal reflux disease	0	0	0	0	1 (12.5)	1 (3.0)
Nausea	0	0	0	1 (16.7) [a]	0	1 (3.0)
Oesophagitis	0	0	0	0	1 (12.5)[a]	1 (3.0)
Stomatitis	0	0	0	1 (16.7)	0	1 (3.0)
Thrombocytopenia	0	0	0	0	1 (12.5)[a]	1 (3.0)
Weight decreased	0	0	0	0	1 (12.5)	1 (3.0)
Decreased appetite	0	0	0	0	1 (12.5)	1 (3.0)

AE=adverse event; BID=twice daily; DLT=dose-limiting toxicity; MedDRA=Medical Dictionary for Regulatory Activities; OD=once daily.

Led to permanent discontinuation of study drug (note only 1 out of the 2 DLTs of vomiting led to permanent discontinuation of study drug)

[b] 1 patient died due to disease progression before completing 1 full cycle of dosing, so this patient was replaced resulting in 7 patients in this cohort.

A patient may have reported more than 1 type of AE.

Includes AEs with an onset date on or after the date of first dose and up to and including 28 days following the date of last dose of study medication.

SUPPLEMENTARY TABLE 6: SUMMARY OF TREATMENT-EMERGENT ADVERSE EVENTS REPORTED IN ≥10% OF ALL PARTICIPANTS (MODULE 1A – ALL PARTICIPANTS, SAFETY ANALYSIS POPULATION)

**MedDRA Preferred Term Number of participants (%)**	**Cohort 1 120 mg OD N=6**	**Cohort 2 240 mg OD N=12**	**Cohort 4 360 mg OD N=12**	**Cohort 3 480 mg OD N=6**	**Cohort 5 180 mg BID N=8**	**Total N=44**
Diarrhoea	6 (100.0)	8 (66.7)	12 (100.0)	6 (100.0)	6 (75.0)	38 (86.4)
Vomiting	4 (66.7)	9 (75.0)	10 (83.3)	6 (100.0)	7 (87.5)	36 (81.8)
Nausea	5 (83.3)	9 (75.0)	11 (91.7)	3 (50.0)	6 (75.0)	34 (77.3)
Fatigue	2 (33.3)	7 (58.3)	6 (50.0)	0	2 (25.0)	17 (38.6)
Abdominal pain	3 (50.0)	1 (8.3)	5 (41.7)	2 (33.3)	1 (12.5)	12 (27.3)
Anaemia	1 (16.7)	5 (41.7)	2 (16.7)	1 (16.7)	1 (12.5)	10 (22.7)
Decreased appetite	1 (16.7)	2 (16.7)	4 (33.3)	1 (16.7)	1 (12.5)	9 (20.5)
ALT increased	1 (16.7)	1 (8.3)	4 (33.3)	1 (16.7)	1 (12.5)	8 (18.2)
Cough	1 (16.7)	4 (33.3)	0	0	3 (37.5)	8 (18.2)
Upper respiratory tract infection	5 (83.3)	1 (8.3)	1 (8.3)	0	1 (12.5)	8 (18.2)
Constipation	0	3 (25.0)	4 (33.3)	0	0	7 (15.9)
AST increased	1 (16.7)	1 (8.3)	2 (16.7)	2 (33.3)	1 (12.5)	7 (15.9)
Dyspnoea	2 (33.3)	1 (8.3)	2 (16.7)	1 (16.7)	0	6 (13.6)
Back pain	2 (33.3)	2 (16.7)	2 (16.7)	0	0	6 (13.6)
Urinary tract infection	0	1 (8.3)	1 (8.3)	1 (16.7)	2 (25.0)	5 (11.4)

AE=adverse event; ALT=alanine aminotransferase; AST=aspartate aminotransferase; BID=twice daily; MedDRA=Medical Dictionary for Regulatory Activities; OD=once daily; PT=preferred term; SOC=system organ class

Within a system organ class, a participant may have reported more than 1 type of AE.

Participants with multiple AEs are counted once for each SOC/PT.

Includes AEs with an onset date on or after the date of first dose and up to and including 28 days following the date of last dose of study medication.

SUPPLEMENTARY TABLE 7: INCIDENCE OF CTCAE GRADE ≥3 EVENTS REPORTED IN MORE THAN 1 PARTICIPANT (MODULE 1A – ALL PARTICIPANTS, SAFETY ANALYSIS POPULATION)

**MedDRA Preferred Term Number of participants (%)**	**Cohort 1 120 mg OD N=6**	**Cohort 2 240 mg OD N=12**	**Cohort 4 360 mg OD N=12**	**Cohort 3 480 mg OD N=6**	**Cohort 5 180 mg BID N=8**	**Total N=44**
Participants with any Grade ≥3 TEAE	2 (33.3)	6 (50.0)	6 (50.0)	4 (66.7)	3 (37.5)	21 (47.7)
Diarrhoea	0	0	1 (8.3)	1 (16.7)	0	2 (4.5)
GGT increased	0	1 (8.3)	1 (8.3)	0	0	2 (4.5)
Dyspnoea	1 (16.7)	0	0	1 (16.7)	0	2 (4.5)
Pulmonary embolism	0	0	1 (8.3)	1 (16.7)	0	2 (4.5)
Anaemia	1 (16.7)	1 (8.3)	0	0	0	2 (4.5)
Thrombocytopenia	0	0	1 (8.3)	0	1 (12.5)	2 (4.5)
Malignant neoplasm progression	1 (16.7)	1 (8.3)	0	0	0	2 (4.5)

AE=adverse event; BID=twice daily; GGT=gamma-glutamyltransferase; MedDRA=Medical Dictionary for Regulatory Activities; OD=once daily; TEAE=treatment-emergent adverse event

A participant may have reported more than 1 type of AE.

Includes AEs with an onset date on or after the date of first dose and up to and including 28 days following the date of last dose of study medication.

SUPPLEMENTARY TABLE 8: SUMMARY OF DRUG-RELATED ADVERSE EVENTS REPORTED IN MORE THAN 1 PARTICIPANT (MODULE 1A – ALL PARTICIPANTS, SAFETY ANALYSIS POPULATION)

**MedDRA Preferred Term Number of participants (%)**	**Cohort 1 120 mg OD N=6**	**Cohort 2 240 mg OD N=12**	**Cohort 4 360 mg OD N=12**	**Cohort 3 480 mg OD N=6**	**Cohort 5 180 mg BID N=8**	**Total N=44**
Participants with any drug-related TEAE	6 (100.0)	12 (100.0)	12 (100.0)	6 (100.0)	8 (100.0)	44 (100.0)
Diarrhoea	6 (100.0)	7 (58.3)	11 (91.7)	6 (100.0)	6 (75.0)	36 (81.8)
Vomiting	4 (66.7)	9 (75.0)	9 (75.0)	6 (100.0)	7 (87.5)	35 (79.5)
Nausea	5 (83.3)	9 (75.0)	11 (91.7)	3 (50.0)	6 (75.0)	34 (77.3)
Fatigue	1 (16.7)	5 (41.7)	6 (50.0)	0	2 (25.0)	14 (31.8)
Abdominal pain	2 (33.3)	1 (8.3)	4 (33.3)	1 (16.7)	0	8 (18.2)
Decreased appetite	1 (16.7)	1 (8.3)	4 (33.3)	1 (16.7)	1 (12.5)	8 (18.2)
AST increased	0	1 (8.3)	1 (8.3)	1 (16.7)	1 (12.5)	4 (9.1)
Weight decreased	0	0	2 (16.7)	0	2 (25.0)	4 (9.1)
Gastroesophageal reflux disease	0	0	1 (8.3)	0	2 (25.0)	3 (6.8)
ALT increased	1 (16.7)	0	0	1 (16.7)	1 (12.5)	3 (6.8)
Blood creatinine increased	0	1 (8.3)	2 (16.7)	0	0	3 (6.8)
Anaemia	1 (16.7)	1 (8.3)	0	0	1 (12.5)	3 (6.8)
Thrombocytopenia	0	0	2 (16.7)	0	1 (12.5)	3 (6.8)
Abdominal pain upper	0	0	0	1 (16.7)	1 (12.5)	2 (4.5)
Dry mouth	0	1 (8.3)	0	0	1 (12.5)	2 (4.5)
Dyspepsia	0	1 (8.3)	1 (8.3)	0	0	2 (4.5)
Dysphagia	0	0	0	0	2 (25.0)	2 (4.5)
Oral pain	0	1 (8.3)	0	0	1 (12.5)	2 (4.5)
Mucosal inflammation	0	0	2 (16.7)	0	0	2 (4.5)
Dehydration	0	1 (8.3)	0	0	1 (12.5)	2 (4.5)

ALT=alanine aminotransferase; AST=aspartate aminotransferase; BID=twice daily; MedDRA=Medical Dictionary for Regulatory Activities; OD=once daily; TEAE=treatment-emergent adverse event.
Within a system organ class, a participant may have reported more than 1 type of adverse event.
Includes adverse events with an onset date on or after the date of first dose and up to and including 28 days following the date of last dose of study medication.

SUPPLEMENTARY TABLE 9: SUMMARY OF DRUG-RELATED ADVERSE EVENTS REPORTED IN MORE THAN 1 PARTICIPANT (MODULE 1B-1 – ALL PARTICIPANTS, SAFETY ANALYSIS POPULATION)

**MedDRA Preferred Term**<br>**Number of Participants**<br>**(%) [a]**	**Total**<br>**360 mg OD CT7001**<br>**N=23**
Participants with any drug-related TEAE	22 (95.7)
Nausea	22 (95.7)
Diarrhoea	21 (91.3)
Vomiting	12 (52.2)
Abdominal pain	4 (17.4)
Abdominal pain upper	2 (8.7)
Constipation	2 (8.7)
Stomatitis	2 (8.7)
Fatigue	9 (39.1)
Mucosal inflammation	2 (8.7)
Alopecia	2 (8.7)
Platelet count decreased	2 (8.7)
Decreased appetite	2 (8.7)
Flushing	2 (8.7)

PT=preferred term; MedDRA=Medical Dictionary for Regulatory Activities; SOC=system organ class; TEAE=treatment-emergent adverse event

[a] Participants with multiple causally-related AEs are counted once for each SOC/PT.

SUPPLEMENTARY TABLE 10: SUMMARY OF DRUG-RELATED ADVERSE EVENTS REPORTED IN MORE THAN 1 PARTICIPANT (MODULE 2A – ALL PARTICIPANTS, SAFETY ANALYSIS POPULATION)

**MedDRA Preferred Term<br>Number of patients (%)**	**240mg OD + fulvestrant (N=6)**	**360mg OD + fulvestrant (N=25)**	**Total (N=31)**
Participants with any drug-related TEAE	**6 (100.0)**	**24 (96.0)**	**30 (96.8)**
Diarrhoea	5 (83.3)	23 (92.0)	28 (90.3)
Nausea	6 (100)	19 (76.0)	25 (80.6)
Vomiting	6 (100)	17 (68.0)	23 (74.2)
Fatigue	2 (33.3)	9 (36.0)	11 (35.5)
Decreased appetite	2 (33.3)	7 (28.0)	9 (29.0)
Abdominal pain	1 (16.7)	6 (24.0)	7 (22.6)
Abdominal pain	1 (16.7)	3 (12.0)	4 (12.9)
Dysgeusia	0	4 (16.0)	4 (12.9)
Headache	2 (33.3)	2 (8.0)	4 (12.9)
AST increased	1 (16.7)	3 (12.0)	4 (12.9)
ALT increased	1 (16.7)	2 (8.0)	3 (9.7)
Dysphagia	0	3 (12.0)	3 (9.7)
Rash	0	3 (12.0)	3 (9.7)
Thrombocytopenia	0	3 (12.0)	3 (9.7)
Constipation	1 (16.7)	1 (4.0)	2 (6.5)
Dry mouth	0	2 (8.0)	2 (6.5)
Flatulence	1 (16.7)	1 (4.0)	2 (6.5)
Mouth ulceration	0	2 (8.0)	2 (6.5)
Stomatitis	1 (16.7)	1 (4.0)	2 (6.5)
Mucosal inflammation	0	2 (8.0)	2 (6.5)
Taste disorder	0	2 (8.0)	2 (6.5)
Weight decreased	0	2 (8.0)	2 (6.5)
Dehydration	0	2 (8.0)	2 (6.5)
Dry skin	1 (16.7)	1 (4.0)	2 (6.5)
Anaemia	0	2 (8.0)	2 (6.5)
Muscular weakness	2 (33.3)	0	2 (6.5)

AE=adverse event; ALT=alanine aminotransferase; AST=aspartate aminotransferase; MedDRA=Medical Dictionary for Regulatory Activities; OD=once daily; PT=preferred term; SOC=system organ class; TEAE=treatment emergent adverse event

Within a system organ class, a patient may have reported more than 1 type of AE.

Patients with multiple AEs are counted once for each SOC/PT.

Includes AEs with an onset date on or after the date of first dose and up to and including 28 days following the date of last dose of study medication.

SUPPLEMENTARY TABLE 11: H-SCORE FOR PATIENT BIOPSIES STAINED FOR pCDK1/2/3 AND pPolII IN MODULE 1A (PK ANALYSIS POPULATION)

Cancer classification	Biopsy location	Dose (mg OD)	pCDK1/2/3 H-Score		pPolII H-Score	
			Pre-dose	On study	Pre-dose	On study
Rhabdomyosarcoma	Axilla	240	148	64	223	167
HR+ breast cancer	Liver	240	234	151	251	232
HR+ breast cancer	Liver	240	125	90	243	225
HR+ breast cancer	Liver	360	89	128	271	262
HR+ breast cancer	Liver	360	91	113	273	281
HR+ breast cancer	Breast	360	178	112	286	271
Average 240mg OD patients			169	102	239	208
Average 360mg OD patients			119	118	277	271
Average all patients			144	110	258	240
SEM all patients			23	12	9	17
p-value (paired t-test, 1 sided)				8.8%		4.4%

HR+ = Hormone receptor positive; OD=once daily; SEM = standard error of the mean
Significance between pre-dose and on study results calculated using Student's one-tailed paired t-test.

SUPPLEMENTARY TABLE 12: ANTIBODIES USED

Antibody	Application	Supplier & Code	Description	Dilution/ Final concentration	Validation
RNA pol II pSer5	Immunohistochemistry (Fig 3); Flow cytometry (Supplementary Figure 5)	Abcam ab193467	Rabbit monoclonal [EPR19015] to RNA polymerase II CTD repeat YSPTSPS (phospho S5)	Immuno-histochemistry: 1 in 8000 0.02 µg/mL<br><br>Flow cytometry: 1 in 100 1.5 µg/mL	Immunohistochemistry: Positive nuclear staining in normal spleen samples. Significant reduction in staining in MCF7 cell line treated with samuraciclib.<br><br>Flow cytometry: Significant reduction in intensity of labelling in peripheral blood mononucleated cells treated *ex vivo* with samuraciclib.<br><br>Vendor: Single band observed on western blot corresponding to RNA polII.
pCDK1/2/3	Immunohistochemistry (Fig 3)	Abcam ab201008	Rabbit monoclonal [EPR19546] to CDK1 (phospho T161) + CDK2 (phospho T160) + CDK3 (phospho T160)	1 in 500 1.2 µg/mL	Immunohistochemistry: Positive nuclear staining in normal tonsil samples. Significant reduction in staining in MCF7 cell line treated with samuraciclib.<br><br>Vendor: Bands corresponding to phosphorylated forms of CDK1, CDK2 and CDK3 observed by western blot.
Goat anti-Rabbit IgG H&L AlexaFluor 488	Flow cytometry (Supplementary Figure 5)	Abcam ab150077	Polyclonal goat anti-rabbit IgG H&L conjugated to AlexaFluor 488	1 in 1000 2.0 µg/mL	Flow cytometry: No significant binding to peripheral blood mononucleated cells in absence of primary antibody.

SUPPLEMENTARY REFERENCES

1. Hammond, M. E. et al. American Society of Clinical Oncology/College of American Pathologists guideline recommendations for immunohistochemical testing of estrogen and progesterone receptors in breast cancer (unabridged version). *Arch. Pathol. Lab. Med.* **134**, e48-72 (2010). doi: 10.5858/134.7.e48.

2. Wolff, A. C. et al. Human epidermal growth factor receptor 2 testing in breast cancer: American Society of Clinical Oncology/College of American Pathologists Clinical Practice Guideline Focused Update. *J. Clin. Oncol.* **36**, 2105-2122 (2018). doi: 10.1200/JCO.2018.77.8738.

3. Rothwell, D. G. et al. Utility of ctDNA to support patient selection for early phase clinical trials: the TARGET study. *Nat. Med.* **25**, 738-743 (2019). doi: 10.1038/s41591-019-0380-z.

4. CEDR Faslodex Approval, 09 September 2010. https://www.accessdata.fda.gov/drugsatfda_docs/nda/2010/021344Orig1s012.pdf

CT7001_001 CORE PROTOCOL

PROTOCOL REDACTED TO REMOVE DETAILS OF OTHER MODULES NOT RELEVANT TO THIS MANUSCRIPT

CLINICAL TRIAL PROTOCOL

**Study Title:**	A Modular, Multipart, Multiarm, Open-label, Phase I/IIa Study to Evaluate the Safety and Tolerability of CT7001 Alone and in Combination with Anti-cancer Treatments in Patients with Advanced Malignancies
**Study Number:**	CT7001_001
**EudraCT Number:**	2017-00202620
**Study Phase:**	Phase I/IIa
**Test Product:**	CT7001
**Indication:**	Treatment of advanced or metastatic tumours, hormone-sensitive metastatic breast cancer (including triple-negative breast cancer), small cell lung cancer, castrate resistant prostate cancer, ovarian cancer and acute myeloid leukaemia
**Sponsor:**	Carrick Therapeutics<br>NovaUCD,<br>Belfield Innovation Park,<br>University College Dublin,<br>Belfield, Dublin 4, Ireland
**Medical Monitor:**	[redacted]<br>Emas Pharma Ltd<br>63-65 Knowl Piece,<br>Wilbury Way, Hitchin<br>Hertfordshire, SG4 0TY, UK<br>Telephone: [redacted]
**Date of Original Protocol:**	Version 2.0 , 29 September 2017
**Amendment**	Version 3.0 – not applicable<br>Version 4.0, 28 March 2018<br>Version 5.0, 10 July 2018<br>Version 6.0 25 September 2019<br>Version 7.0, 15 January 2019<br>Version 8.0, 06 March 2019<br>Version 9.0 26 March 2019

Confidentiality Statement

The information in this document is confidential and will not be disclosed to others without written authorisation from the Sponsor, except to the extent necessary to obtain informed consent from persons receiving the study drug or their legal guardians, or for discussions with Regulatory Authorities, Institutional Review Boards, Ethics Committees, or persons participating in the conduct of the study. Do not copy or distribute without written permission from the Sponsor.

SIGNATURE PAGE

[redacted]
Carrick Therapeutics, Senior Medical Director

[redacted]

Matthew Krebs
Chief Investigator

[redacted]
Lead Biostatistician, Phastar

This modular, Phase I/IIa study aims to investigate the optimal dose of CT7001 when used as monotherapy dose or in combination with other anti-cancer treatments.

The design consists of a core study protocol (**Volume 1**) and individual Modules. Each Module has the following main objectives:

- **Module 1 Part A and Part B (Volume 2)**: To evaluate the safety, tolerability, pharmacokinetics (PK) and pharmacodynamic (PDc) effects of CT7001 as monotherapy and to determine the minimally biologically active dose (MBAD) and maximum tolerated dose (MTD) in patients with advanced solid malignancies.
- **Module 1 Part B: (Volume 2)**: To refine the safety, tolerability, and PK and PDc profiles of CT7001 in patients with advanced solid malignancies, potentially including triple-negative breast cancer (TNBC), small cell lung cancer (SCLC), castrate resistant prostate cancer (CRPC), ovarian cancer patients, and other appropriate cancer indications.
- **Module 1 Part B-1 – TNBC Expansion (Volume 3):** To determine the recommended Phase 2 dose of CT7001 as monotherapy, further characterize safety, tolerability and blood concentrations of CT7001, and explore its anti-tumour activity in triple negative breast cancer.
- **Module 2 (Volume 4):** To evaluate the tolerability of the combination of CT7001 and fulvestrant in patients with advanced hormone receptor positive breast cancer (Part A) and compare the efficacy of the combination versus fulvestrant alone (Part B).
- **Module 3 (Volume 5):** To evaluate of the safety, tolerability, and antitumour activity of CT7001 as monotherapy in patients with acute myeloid leukaemia (AML).
- **Module 4 (Volume 6):** To explore the effect of food on the total and peak exposure of CT7001, when given as monotherapy to patients with advanced solid malignancies.
- **Module 5 (Volume 7):** To evaluate CT7001 in combination with anti-programmed death receptor 1 (anti-PD-1) (nivolumab or pembrolizumab) or anti-programmed death ligand 1 (anti-PD-L1) (atezolizumab or durvalumab) monoclonal antibodies (mAb) in patients with advanced solid malignancies.

TABLE OF CONTENTS

SIGNATURE PAGE **2**

LIST OF TABLES **10**

LIST OF FIGURES **10**

INVESTIGATOR SIGNATURE PAGE **11**

LIST OF ABBREVIATIONS **12**

SYNOPSIS OF FULL STUDY CT7001_001 .... **17**

V1 VOLUME 1 -CORE PROTOCOL **23**

V1.1 BACKGROUND **23**

V1.1.1 Introduction 23
V1.1.2 CT7001 23
V1.1.3 CDK7 23
V1.1.4 Overview of Nonclinical Data 23
V1.1.5 Current Clinical Data of CT7001 25

V1.2 CORE STUDY OBJECTIVES AND ENDPOINTS **30**

V1.2.1 Core Study Objectives 30
V1.2.2 Core Study Endpoints 31

V1.3 CORE PATIENT SELECTION **32**

V1.3.1 Core Inclusion Criteria 32
V1.3.2 Core Exclusion Criteria 34

V1.4 CORE STUDY DESIGN AND RATIONALE **36**

V1.4.1 Study Design 36
V1.4.2 Rationale for Study and Study Design 38

V1.5 STUDY TREATMENT **39**

V1.5.1 Drug Supply 39
V1.5.2 Dispensing 40
V1.5.3 Dosing Regimen of CT7001 40
V1.5.4 Drug Administration 40
V1.5.5 General Rules 40
V1.5.6 Medication Dosing Errors 41
V1.5.7 Intra-Subject Dose Modification 41
V1.5.8 Inter-Patient Dose Modification 44
V1.5.9 Compliance 44
V1.5.10 Drug Storage and Accountability 44
V1.5.11 Concomitant Medications 44
V1.5.12 Contraception 49

V1.6 STUDY PROCEDURES AND VARIABLES (ALL MODULES) **49**

V1.6.1 Data Collection 50

V1.6.2 Safety ... 50
V1.6.3 Core Study Pharmacokinetics ... 52
V1.6.4 Core Biomarker Analysis ... 52
V1.6.5 Exploratory Research ... 53
V1.6.6 Biological Sampling Procedures ... 54
V1.6.7 Anti-tumour Activity ... 55

V1.7 ADVERSE EVENT REPORTING ... 56

V1.7.1 Reporting Period ... 56
V1.7.2 Definition of Adverse Event ... 57
V1.7.3 Definition of Serious Adverse Events ... 57
V1.7.4 Hospitalization ... 59
V1.7.5 Severity Assessment ... 60
V1.7.6 Causality Assessment ... 61
V1.7.7 Exposure During Pregnancy ... 61
V1.7.8 Occupational Exposure ... 62
V1.7.9 Withdrawal Due to Adverse Events (See Also Section on Patient Withdrawal) ... 62
V1.7.10 Eliciting Adverse Event Information ... 63
V1.7.11 Reporting Requirements ... 63

V1.8 DATA ANALYSIS AND STATISTICAL METHODS ... 64

V1.8.1 Core Study Endpoints ... 64
V1.8.2 Determination of Sample Size ... 65
V1.8.3 Description of Analysis Data Sets ... 66
V1.8.4 Methods of Statistical Analysis ... 66

V1.9 ETHICS ... 71

V1.9.1 Institutional Review Board (IRB)/Independent Ethics Committee (IEC) ... 71
V1.9.2 Ethical Conduct of the Study ... 72
V1.9.3 Patient Information and Confidentiality ... 72
V1.9.4 Patient Consent ... 72
V1.9.5 Potential Risks and Benefits ... 73
V1.9.6 Patient Recruitment ... 75
V1.9.7 Reporting of Safety Issues and Serious Breaches of the Protocol or ICH GCP ... 75

V1.10 QUALITY CONTROL AND QUALITY ASSURANCE ... 75

V1.11 DATA HANDLING AND RECORD KEEPING ... 75

V1.11.1 Case Report Forms/Electronic Data Record ... 75
V1.11.2 Record Retention ... 76

V1.12 DEFINITION OF END OF TRIAL ... 76

V1.12.1 End of Trial in all Participating Countries ... 76
V1.12.2 End of Trial in a Member State of the European Union ... 76

V1.13 SPONSOR DISCONTINUATION CRITERIA ... 76

V1.14 ADMINISTRATIVE PROCEDURES AND CONSIDERATIONS ... 77

V1.14.1 Safety Review Committee ... 77
V1.14.2 Protocol Amendments ... 77

V1.14.3 Clinical Study Report 77
V1.14.4 Financing and Insurance 77

V1.15 PUBLICATION OF STUDY RESULTS 77

V1.15.1 Communication of Results by Carrick 77
V1.15.2 Publications by Investigators 78

V2 VOLUME 2- MODULE 1A AND 1B – MONOTHERAPY IN PATIENTS WITH ADVANCED SOLID MALIGNANCIES 80

LIST OF ABBREVIATIONS (IN ADDITION TO CORE) 81

V2.1 INTRODUCTION 82

V2.1.2 Module 1 Part A and B Cohort Expansions 85
V2.1.3 Rationale 85
V2.1.4 Module 1 Benefit/Risk and Ethical Assessment 88

V2.2 MODULE 1 OBJECTIVES 90

V2.3 PATIENT SELECTION AND RESTRICTIONS ON STUDY 91

V2.3.1 Additional Module 1 Inclusion Criteria 91
V2.3.2 Additional Module 1 Parts A and B Exclusion Criteria 92

V2.4 STUDY TREATMENT, CONDUCT AND WITHDRAWAL 92

V2.4.1 Treatment 92
V2.4.2 Module 1 Part A - Dose Escalation 92
V2.4.3 Module 1 Part A & B – Tumour Biopsy Cohort Expansion 95

V2.5 MODULE 1 PART A AND PART B: PLAN AND TIMING OF PROCEDURES 95

V2.5.1 Module 1 Plan 96
V2.5.2 Follow-up Period 97

V2.6 COLLECTION OF STUDY VARIABLES 97

V2.6.1 Recording of Data 97
V2.6.2 Screening 97
V2.6.3 Safety 98
V2.6.4 Pharmacokinetics 100
V2.6.5 Biomarkers 101
V2.6.6 Biological Sampling Procedures 104
V2.6.7 Anti-tumour Activity 108

V2.7 EVALUATION AND CALCULATION VARIABLES AND STATISTICAL METHODS 110

V2.7.1 Definition of Module 1 Study Endpoints 110
V2.7.2 Determination of Sample Size 113
V2.7.3 Calculation or Derivation of Module 1 Safety Variables 113
V2.7.4 Calculation or Derivation of Module 1 Pharmacokinetic Variables 113
V2.7.5 Calculation or Derivation of Module 1 Biomarker Variables 113
V2.7.6 Calculation or Derivation of Module 1 Exploratory Research Variables 113
V2.7.7 Calculation or Derivation of Module 1 Antitumour Activity Variables 113
V2.7.8 Description of Analysis Sets 113

V2.7.9 Methods of Statistical Analysis 113

V2.8 MODULE 1 PART A STUDY TIMETABLE 115

V3 VOLUME 3 – MODULE 1 PART B-1 (TNBC) - PHASE 1B EXPANSION STUDY OF CT7001 AS MONOTHERAPY IN PATIENTS WITH METASTATIC OR LOCALLY ADVANCED TRIPLE-NEGATIVE BREAST CANCER 121

V4 VOLUME 4 - MODULE 2 - EVALUATION OF CT7001 IN COMBINATION WITH FULVESTRANT IN PATIENTS WITH ADVANCED HORMONE RECEPTOR POSITIVE BREAST CANCER, WHO HAD PREVIOUS TREATMENT WITH AN AROMATASE INHIBITOR AND A CDK4/6 INHIBITOR 122

V5 VOLUME 5 – POTENTIAL MODULE 3 – TO EVALUATE CT7001 AS MONOTHERAPY IN PATIENTS WITH ACUTE MYELOID LEUKAEMIA 123

LIST OF ABBREVIATIONS (IN ADDITION TO CORE) 124

V5.1 INTRODUCTION 125

V5.2 STUDY MODULE DESIGN AND FLOWCHART 125

V5.2.1 Overall Module 3 Design 125
V5.2.2 Module 3 Part A 125
V5.2.3 Module 3 Part B 126
V5.2.4 Rationale 127
V5.2.5 Module 3 Study Timetable 127
V5.2.6 Module 3 Benefit/Risk and Ethical Assessment 127

V5.3 MODULE 3 OBJECTIVES 128

V5.4 MODULE 3 PATIENT SELECTION 129

V5.4.1 Module 3 Inclusion Criteria 129
V5.4.2 Inclusion/Exclusion Criteria 129
V5.4.3 Restrictions 129
V5.4.4 CT7001 129

V5.5 MODULE 3 PLAN AND TIMING OF PROCEDURES 129

V5.6 MODULE 3 COLLECTION OF STUDY VARIABLES 129

V5.6.1 Biomarkers 129

V5.7 MODULE 3 EVALUATION AND CALCULATION OF VARIABLES AND STATISTICAL METHODS 130

V5.7.1 Definition of Study Endpoints 131
V5.7.2 Determination of Sample size 132

V6 VOLUME 6 - MODULE 4 – TO EVALUATE FOOD EFFECT ON THE BIOAVAILABILITY OF CT7001 133

LIST OF ABBREVIATIONS (IN ADDITION TO CORE) 134

V6.1 INTRODUCTION 135

V6.2 STUDY MODULE DESIGN AND FLOWCHART 135

V6.2.1 Overall Module 4 Design 135
V6.2.2 Module 4 Objectives 135
V6.2.3 Rationale for Conducting a Food Effect Study 135
V6.2.4 Rationale for Choice of Dose of CT7001 135
V6.2.5 Module 4 Benefit/Risk and Ethical Assessment 136
V6.2.6 Overall Module 4 Benefit/Risk and Ethical Assessment 138
V6.2.7 Assessment of the Impact of Food on the PK of CT7001 138
V6.2.8 Evaluation of Calculation of Variables and Statistical Methods 139

V6.3 MODULE 4 PATIENT SELECTION 139

V6.3.1 Inclusion/Exclusion criteria 139
V6.3.2 Restrictions 140

V6.4 CT7001 TREATMENT OF PATIENTS 140

V6.4.1 Emerging Data 140

V6.5 MODULE 4 COLLECTION OF STUDY VARIABLES 143

V6.6 MODULE 4 STUDY TIMETABLES 144

V7 VOLUME 7 - POTENTIAL MODULE 5 - SAFETY AND TOLERABILITY AND ANTI-TUMOUR ACTIVITY OF CT7001 IN COMBINATION WITH MONOCLONAL ANTIBODIES IN PATIENTS WITH ADVANCED MALIGNANCIES 147

LIST OF ABBREVIATIONS (IN ADDITION TO CORE) 148

V7.1 INTRODUCTION 149

V7.2 STUDY MODULE DESIGN AND FLOWCHART 149

V7.2.1 Overall Module 5 Design 149
V7.2.2 Module 5 Part A 150
V7.2.3 Module 5 Part B 151
V7.2.4 Module 5 Rationale 152
V7.2.5 Module 5 Study Timetable 152
V7.2.6 Module 5 Benefit/Risk and Ethical Assessment 152

V7.3 MODULE 5 OBJECTIVES 155

V7.4 MODULE 5 PATIENT SELECTION 156

V7.4.1 Inclusion/Exclusion Criteria 156
V7.4.2 Restrictions 156

V7.5 MODULE 5 STUDY TREATMENT 156

V7.5.1 CT7001 156
V7.5.2 Nivolumab 156
V7.5.3 Pembrolizumab 156

V7.6 MODULE 5 PLAN AND TIMETABLE 156

V7.7 MODULE 5 COLLECTION OF STUDY VARIABLES 156

V7.7.1 Biomarkers 156

V7.8 MODULE 5 EVALUATION AND CALCULATION OF VARIABLES AND STATISTICAL METHODS 156

REFERENCES 157

APPENDIX A INTERPRETATION OF AE CAUSALITY 160

APPENDIX B MEDICATIONS, HERBAL SUPPLEMENTS AND FOODS THAT SIGNIFICANTLY INDUCE OR INHIBIT CYTOCHROME P450 3A4 OR P-GLYCOPROTEIN ACTIVITY 161

AB.1 Strong CYP3A4, CYP2C19 and/or CYP2D6 Inhibitors 161

AB.2 Strong CYP3A4, CYP2C19 and/or CYP2D6 Inducers 162

AB.2.1 Drugs whose clearance are dependent on CYP3A4 and have a narrow therapeutic index 162

AB.3 P-Glycoprotein (PGP) Inhibitors and Inducers 163

APPENDIX C ACTIONS REQUIRED IN CASES OF COMBINED INCREASE OF AMINOTRANSFERASE AND TOTAL BILIRUBIN – HY'S LAW 165

AC.1 Background 165

AC.1.1 Investigator Accountabilities 165
AC.1.2 Potential HY's Law (PHL) 165
AC.1.3 Hy's Law (HL) 165
AC.1.4 If PHL criteria are not met: 166
AC.1.5 If PHL criteria are met: 166

AC.2 Follow-up, Review and Assessment of PHL 166

AC.2.1 Cases 166
AC.2.2 Actions Required for Repeat Episodes of PHL 167

APPENDIX D DRUGS KNOWN TO PREDISPOSE TO TORSADE DE POINTES 168

APPENDIX E RESPONSE EVALUATION CRITERIA IN SOLID TUMOURS (RECIST) VERSION 1.1 170

APPENDIX F NEW YORK HEART ASSOCIATION (NYHA) FUNCTIONAL CLASSIFICATION 174

APPENDIX G FULVESTRANT SPC 175

APPENDIX H NIVOLUMAB SPC 188

APPENDIX I PEMBROLIZUMAB SPC 189

LIST OF TABLES

Table 1: Status of Modules 1A and 4 as of 26-Nov-2018 26
Table 2: CT7001 Excipients 39
Table 3: Predicted Efficacious Human Doses based on Scaling Factors from Mouse to Human 86
Table 4: C_{max} and AUC_{0-t} Values from 28-day Toxicology Studies 86
Table 5: Margins from the Proposed Human Doses to Doses used in the GLP Rat and Dog Studies based on Scaling Factors 87
Table 6: Blood Volume – Screening and Cycle 0 104
Table 7: Blood Volume – Cycle 1 105
Table 8: Blood Volume – Cycle 2 105
Table 9: Blood Volume – Cycle 3 and subsequent Cycles (Day 1 only) 106
Table 10: Blood Volume – End of Treatment Visit 106
Table 11: Blood Volumes Required 107
Table 12: Schedule of Events – Module 1 Part A (Dose Escalation): Each Cycle = 21 Days. Cycle 3 onwards, Day 1 visits only 115
Table 13: Schedule of Events - Module 1 Part B (Expansion): Each Cycle = 21 Days, Cycle 3 onwards, Day 1 visits only 118
Table 14: Schedule of Events – Module 4 144
Table 15: PK Blood Sample Collections 146

LIST OF FIGURES

Figure 1: CT7001 decreases pPolII levels in PBMC. 28
Figure 2: Average reticulocyte counts across first three dosing cohorts (120, 240 & 480 mg). 29
Figure 3: Overall Module 1 Design 83
Figure 4: Schematic of Module 4 Study Design 142

INVESTIGATOR SIGNATURE PAGE

CT7001_001 A Modular, Multipart, Multiarm, Open-label, Phase I/IIa Study to Evaluate the Safety and Tolerability of CT7001 Alone and in Combination with Anticancer Treatments in Patients with Advanced Malignancies

I have read the Protocol and agree to conduct the trial in compliance with the International Council for Harmonisation Guideline for Good Clinical Practice and applicable regulatory requirements and to inform all who assist me in the conduct of this trial of their responsibilities and obligations.

Signed: ____________________________ Date: _________________________

Print Name: ________________________

LIST OF ABBREVIATIONS

Abbreviation	Definition
AE	adverse event
ALT	alanine aminotransferase
AML	acute myeloid leukaemia
Anti-PD-1	anti-programmed death receptor 1
Anti-PD-L1	anti-programmed death ligand 1
AST	aspartate aminotransferase
ANC	absolute neutrophil count
ATP	adenosine triphosphate
AUC	area under the plasma concentration-time curve
AUC_{tau}	area under the plasma concentration-time curve in the dosing interval
AUC_{0-24}	area under the plasma concentration-time curve from Time 0 to 24 hours
AUC_{0-48}	area under the plasma concentration-time curve from Time 0 to 248 hours
AUC_{0-t}	area under the plasma concentration-time curve from Time 0 to the time of the last measurable concentration
$AUC_{0-\infty}$	area under the plasma concentration-time curve from Time 0 extrapolated to infinity
BOR	best objective response
C0D1	Day 1 of Cycle 0
C0D2	Day 2 of Cycle 0
CAK	CDK-activating kinase
CBR	clinical benefit response
CBRR	clinical benefit response rate
CDK	cyclin-dependent kinase
CL/F	apparent plasma clearance
CL_{ss}/F	apparent plasma clearance at steady state
C_{max}	maximum observed plasma concentration

Abbreviation	Definition
$C_{ss,max}$	maximum observed plasma concentration at steady state
$C_{ss,min}$	minimum observed plasma concentration at steady state
CNS	central nervous system
CR	complete response
CRPC	castrate-resistant prostate cancer
Crl:WI [Han]	Han Wistar rat (Charles River Wistar Hannover Rat)
CRM	continual reassessment method
CRO	contract research organisation
CSR	Clinical Study Report
CT	computed tomography
CTC	common toxicity criteria
CTCAE	Common Terminology Criteria for Adverse Events
CTD	C-terminal domain
ctDNA	circulating tumour deoxyribonucleic acid
CTLA-4	cytotoxic lymphocyte associated protein 4
CYP	cytochrome P450
DILI	drug induced liver injury
DLT	dose-limiting toxicity
DNA	deoxyribonucleic acid
DoR	durability of response
DRR	durable response rate
EC	Ethics Committee
ECG	electrocardiogram
ECOG	Eastern Cooperative Oncology Group
eCRF	electronic case report form
FDA	Food and Drug Administration
FTIH	first time in human
GCP	Good Clinical Practice

Abbreviation	Definition
G-CSF	granulocyte-colony stimulating factor
GI	gastrointestinal
GLP	Good Laboratory Practice
HED	human equivalent dose
HEK	human embryonic kidney
hERG	human-ether-a go-go-gene
HNSCC	head and neck squamous cell carcinoma
HR+	Hormone receptor positive
HV	healthy volunteers
Hy	Hy's Law
IB	Investigator's Brochure
IC_{50}	half maximal inhibitory concentration
ICF	informed consent form
ICH	International Council for Harmonisation
IMP	Investigational Medicinal Product
INR	international normalised ratio
IO	immuno-oncology
IP	investigational product
IUD	intrauterine device
IV	intravenous
LFT	liver function test
LH	luteinizing hormone
mAb	monoclonal antibody
MAD	multiple ascending dose
MBAD	minimally biological active dose
MedDRA	Medical Dictionary for Regulatory Activities
MFD	maximum feasible dose
MRI	magnetic resonance imaging

Abbreviation	Definition
MRT	mean residence time
MRT_{ss}	mean residence time at steady state
MTD	maximum tolerated dose
NE	not evaluable
NPD	nonprogressive disease
NSCLC	non small cell lung cancer
OD	once daily
ORR	objective response rate
OS	overall survival
PBMC	peripheral blood mononuclear cell
PD	progression of disease
PDc	pharmacodynamic
PD-1	programmed death receptor 1
PFS	progression-free survival
PG	pharmacogenetic
PGP	p-glycoprotein
PHL	Potential Hy's Law
PID	percentage intended dose
PK	pharmacokinetic
PolII	RNA polymerase II
PR	partial response
PV	pharmacovigilance
QTcF	QT interval corrected for heart rate by the Fridericia formula
RDI	relative dose intensity
RECIST	Response Evaluation Criteria in Solid Tumours
RNA	ribonucleic acid
SAD	single ascending dose
SAE	serious adverse event

Abbreviation	Definition
SAP	statistical analysis plan
SCLC	small cell lung cancer
SD	stable disease
SOC	System Organ Class
SoE	Schedule of Events
SPC	Summary of Product Characteristics
SRC	Safety Review Committee
STD 10	severely toxic dose in 10% of animals
$T_{½}$	apparent elimination half-life
TBL	total bilirubin
TEAE	treatment-emergent adverse event
TL	target lesion
T_{max}	time to maximum observed plasma concentration
TNBC	triple-negative breast cancer
ULN	upper limit of normal

SYNOPSIS OF FULL STUDY CT7001_001

**Sponsor**: Carrick Therapeutics	
**Study Title**: A Modular, Multipart, Multiarm, Open-label, Phase I/IIa Study to Evaluate the Safety and Tolerability of CT7001 Alone and in Combination with Anticancer Treatments in Patients with Advanced Malignancies	
Study Number: CT7001_001	**Study Phase:** Phase I/IIa
EudraCT Number: 2017-00202620	ClinTrials.Gov ID: NCT03363893

CORE STUDY OBJECTIVES

Primary Objectives:

- To investigate the safety and tolerability of CT7001 given alone or in combination with anti-cancer treatments

Secondary Objectives:

- To characterise the pharmacokinetics (PK) of CT7001, given alone or in combination with anti-cancer treatments, after a single dose and at steady state after multiple dosing.
- To assess the biological and antitumour activity of CT7001, given alone or in combination with anticancer treatments.

Exploratory Objectives:

- To investigate the presence and identity of metabolites of CT7001 and, if appropriate, characterise their PK.
- To explore the relationship between PK and safety, anti-tumour activity, and biological activity and the impact of patient characteristics on PK.
- To collect and store deoxyribonucleic acid (DNA) for future exploratory research into genes and genetic variation that may influence response to CT7001 (i.e., distribution, safety, tolerability, and efficacy).
- To collect and store predose plasma and serum samples and archival tumour tissue, if available, for potential future exploratory research into factors that may influence the development of agents to treat human disease or response to CT7001 (i.e., distribution, safety, tolerability. and efficacy).
- To investigate predictive markers and acquired resistance to CT7001 that may be observed in plasma circulating tumour DNA (ctDNA).
- To investigate predictive markers and acquired resistance to CT7001 that may be observed in tumour tissue.

Core Study Design: The modular study design allows investigation of the optimal dose of CT7001 when used as monotherapy dose or in combination other anticancer treatments. Module 1 Part A will be performed initially and includes intensive safety monitoring to ensure the safety of study patients. Part B will refine the safety, tolerability, PK and PDc profiles of CT7001 in subjects with advanced solid malignancies, potentially including TNBC, SCLC, CRPC, Ovarian cancer subjects and other appropriate cancer indications. Further modules will be added at a later date as protocol amendments.

The individual modules have the following main objectives:

- **Module 1 Part A**: To evaluate the safety, tolerability, PK and PDc effects of CT7001 as monotherapy and to determine the minimally biologically active dose (MBAD) and maximum tolerated dose (MTD) in patients with advanced solid malignancies.
- **Module 1 Part B**: To refine the safety, tolerability, and PK and PDc profiles of CT7001 in patients with advanced solid malignancies, potentially including TNBC, SCLC, CRPC, Ovarian cancer subjects and other appropriate cancer indications.
- **Module 1 Part B-1 (Cohort 1 – TNBC):** To determine the recommended Phase 2 dose of CT7001 as monotherapy, further characterize safety, tolerability and blood concentrations of CT7001, and explore its anti-tumour activity in triple negative breast cancer.
- **Module 2:** To evaluate the tolerability of the combination of CT7001 and fulvestrant in patients with advanced hormone receptor positive breast cancer (Part A) , compare the efficacy CT7001 combined with fulvestrant versus fulvestrant alone (Part B) and evaluate the efficacy of CT7001 combined with fulvestrant in patients whose disease had progressed on or after fulvestrant plus placebo in Part B of the study (Part C).
- **Module 3:** To evaluate the safety, tolerability, and antitumour activity of CT7001 as monotherapy in patients with acute myeloid leukaemia (AML).
- **Module 4:** To explore the effect of food on the total and peak exposure of CT7001, when given as monotherapy to patients with advanced solid malignancies.
- **Module 5:** To evaluate CT7001 in combination with anti-programmed death receptor 1 (anti-PD-1) (nivolumab or pembrolizumab) or anti-programmed death ligand 1 (anti-PD-L1) (atezolizumab or durvalumab) monoclonal antibodies (mAb) in patients with advanced solid malignancies.

The CT7001 starting dose and schedule of CT7001 in Module 1A has been selected using the pre-clinical data and the Food and Drug Administration (FDA) guidance for industry for selection of a starting dose in cancer patients. The starting dose in subsequent modules will be determined by the Safety Review Committee (SRC) on the basis of emerging preclinical and clinical data.

In each module, the frequency of dosing, any washout period to assess PK and dosing staggering interval between patients may change based upon emerging data as reviewed and agreed by the SRC without the requirement to submit a substantial amendment to the protocol. The frequency of PK sampling by venepuncture may also be modified based on SRC review, and may include up to three additional PK samples of 4ml within any given cycle to better characterise the PK profile based upon emerging PK data.

Only patients who provide written informed consent and meet all inclusion and no exclusion criteria will be enrolled into the respective study module.

In all treatment combinations (e.g. Module 2, CT7001 with fulvestrant), the dose of each approved standard of care combination agent will not exceed its approved dose.

Core Study Centres: To be defined separately for each module. Centres will be based in the United Kingdom, continental Europe and the USA.

**Core Number of Patients Planned (for all Modules):** The number of patients to be enrolled is dependent on the size and number of cohorts defined for each module. Subsequent to Module 1A protocol amendments will be required for each new module.
**Core Diagnosis and Main Eligibility Criteria (for all modules):** Patients at least 18 years of age with histological or cytological confirmation of an advanced malignancy.
**Test Product:** CT7001, oral<br>**Concomitant Study Drugs:** To be defined within the respective modules.
**Core Endpoints**<br>Safety and Tolerability<br>• Adverse Events (AEs).<br>• Clinical laboratory results (haematology including reticulocyte count, serum chemistry including tumour specific biomarkers, coagulation, urinalysis).<br>• Physical examination findings.<br>• Eastern Cooperative Oncology Group (ECOG) performance status.<br>• Electrocardiogram (ECG) parameters (heart rate, PR interval, QRS complex, QT interval, and QT interval corrected for heart rate by the Fridericia formula (QTcF).<br>• Weight.<br>• Vital signs (supine systolic and diastolic blood pressure and pulse, temperature, respiratory rate, oxygen saturation).<br>Efficacy<br>• Objective response rate<br>• Progression-free survival<br>PK and PDc<br>• PK Parameters for CT7001 (and main CT7001 metabolite, if applicable).<br>• Biological Activity Parameters (Biomarkers).<br>• Anti-tumour Activity.<br>Core Exploratory Endpoints<br>• Identification of CT7001 metabolites<br>• Biomarkers for *de novo* and acquired resistance to CT7001 and for efficacy.
**Main Analyses:**<br>Safety<br>Safety analyses will be performed in each study module using the Safety Population.<br>Safety data AEs, and treatment-emergent AEs [TEAEs]) from all cycles of treatment will be presented graphically, as is deemed appropriate for each module. These will be summarized by Medical Dictionary for Regulatory Activities (MedDRA) System Organ Class (SOC),

MedDRA preferred term (PT), and Common Terminology Criteria for Adverse Events (CTCAE) grade.

Serious Adverse Events (SAEs) will be analysed and reported separately.

Details of any deaths will be listed.

All laboratory results, ECOG performance status, ECG parameters, weight and vital sign measurements will be listed individually by patient and summarised descriptively in each module. Any qualitative urinalysis assessments will be summarised using the number of patients with results of negative, trace, or positive.

Abnormal physical examination findings will be listed.

CT7001 PK analyses will be performed using the PK Population. PK parameters will be derived using standard non-compartmental methods using R Statistical Package or Phoenix WinNonlin; graphical presentations of PK data will be used as is deemed appropriate.

CT7001 plasma concentrations and derived PK parameters will be summarised by dose level. Parameters following single and multiple dosing will be summarised separately. Appropriate statistics will be used to summarise plasma concentrations and PK parameters.

All plasma concentrations and PK parameters results will be listed individually by patient for any patient with a measurable plasma concentration and summarised descriptively for the PK population.

Biomarker analyses will be performed in each module using the Biomarker Population. Further details will be provided in the Laboratory Manual.

Analyses of Anti-Tumour Activity

Anti-tumour activity endpoints will be analysed using the Evaluable for Response Population and when applicable the Intent-To-Treat Population.

Tumour response will be determined using Response Evaluation Criteria in Solid Tumours (RECIST) version 1.1.

Objective response rate (ORR) is defined as the percentage of patients who have at least one response of complete response (CR) or partial response (PR) prior to any evidence of progression.

Duration of Response (DOR), defined as the time from documentation of tumour response to disease progression..

The Disease Control Rate (DCR) is defined as the percentage of patients with no documented objective disease progression for at least 4 months.

The percentage change in tumour size will be derived at each tumour imaging assessment by the best percentage change from Baseline in the sum of the diameters of target lesions (TL).

Progression-free Survival (PFS), defined as the time from start of treatment until the date of objective disease progression or death by any cause, whichever occurs first.

Efficacy results will be descriptively summarised and Kaplan-Meier plots for DOR and PFS will be provided as appropriate.

MODULE 1 – PHASE 1 STUDY OF CT7001 AS MONOTHERAPY

Module 1 Part A & B (Module 1A & Module 1B, Volume 2)

- First-in-human single and multiple ascending dose to investigate safety, tolerability, PK and PDc and to define the minimally biological active dose (MBAD) and maximum tolerated dose (MTD)

Module 1A includes breast cancer cohort with sequential tumour biopsies to evaluate PDc in tumour tissue. The breast cancer biopsy cohort will be initiated upon definition of MBAD and participation will require separate informed consent. In Part B, additional evaluable patients in a maximum of 4 cohorts will be treated at the MBAD established in Module 1 Part A in each specific disease group, which will include, but not be limited to (TNBC), SCLC, CRPC and ovarian cancer patients.

Module 1 Part B-1 (TNBC) (Module 1B,Volume 3)

Phase Ib expansion cohort in patients with advanced TNBC to further characterize the safety and tolerability of CT7001, define a recommended Phase 2 dose (RP2D) in patients with solid tumours and explore the efficacy of CT7001 in patients with metastatic or recurrent TNBC who had previously received a taxane and an anthracycline and at least one line of chemotherapy for metastatic or recurrent disease.

MODULE 2 – EVALUATION OF CT7001 IN COMBINATION WITH FULVESTRANT IN PATIENTS WITH ADVANCED HORMONE RECEPTOR POSITIVE BREAST CANCER, WHO HAD PREVIOUS TREATMENT WITH AN AROMATASE INHIBITOR AND A CDK4/6 INHIBITOR (Volume 4)

Module 2 Part A (Module 2A, Volume 4)

Single-arm evaluation of safety and tolerability of CT7001 in combination with fulvestrant to define the recommended dose for Phase II testing.

Module 2 Part B (Module 2B, Volume 4)

Randomised Phase II study of CT7001 in combination with fulvestrant versus fulvestrant alone for a preliminary comparison of safety and efficacy.

Module 2 Part C (Module 2C, Volume 4)

Single arm Phase 2a study of CT7001 in combination with fulvestrant in patients who had received fulvestrant and placebo (Arm B) in Part B and developed progressive disease.

MODULE 3 – TO EVALUATE CT7001 AS MONOTHERAPY IN PATIENTS WITH ACUTE MYELOID LEUKAEMIA (Volume 5)

Phase I/IIa study to define a safe and active dose of CT7001 in patients with relapsed or refractory acute myeloid leukaemia who had previously received at least two lines of therapy.

MODULE 4 – TO EVALUATE FOOD EFFECT ON THE BIOAVAILABILITY OF CT7001 (Volume 6)

Randomised, balanced, single-dose, two-treatment, two-period, two-sequence (fed versus fasting) crossover study to evaluate the effect of a high-fat, high-calorie meal on the total and peak blood exposure of CT7001 when given as monotherapy to patients with advanced solid malignancies.
**MODULE 5 – TO EVALUATE SAFETY,TOLERABILITY AND ANTI-TUMOUR ACTIVITY OF CT7001 IN COMBINATION WITH ANTI-PD-1 OR -PD-L1 MONOCLONAL ANTIBODIES IN PATIENTS WITH ADVANCED MALIGNANCIES (Volume 7)**
Single-arm evaluation of safety and tolerability of CT7001 in combination with an anti-PD-1 or anti-PD-L1 antibody in patients with metastatic or recurrent solid tumours to define a RP2D and explore the ani-tumour activity.

V1 VOLUME 1 -CORE PROTOCOL

V1.1 BACKGROUND

V1.1.1 Introduction

CT7001 is a small molecule, adenosine triphosphate (ATP) competitive, selective oral inhibitor of cyclin-dependent kinase 7 (CDK7). A first-in-human modular Phase 1/2 clinical study was initiated in November 2017 (EudraCT number: 2017-002026-20; ClinicalTrials.gov identifier: NCT03363893). The single- and multiple-ascending dose part of the Phase 1 study (Module 1A) is currently still open but has determined 360 mg as a safe and biologically active dose for further testing in Module 1B. A study of the effect of food on the bioavailability of CT7001 (called Module 4) is ongoing and is scheduled to complete before the end of 2018. Section V1.1.5 will review the status of and data from Module 1A and Module 4 as per 26-Nov-2018. Further modules are planned in which Module 3 will evaluate CT7001 in patients with acute myeloid leukaemia (AML) and Module 5 will investigate CT7001 in combination with anti-PD-1 or anti-PD-L1 monoclonal antibodies in patients with advanced solid malignancies. Additional modules may be added at a later date following approval of the relevant substantial protocol amendment.

V1.1.2 CT7001

CT7001 (previously also known as ICEC0942) is a small molecule, ATP competitive, selective oral inhibitor of CDK7 which potently inhibits all key biological effects of CDK7 in cancer (Patel et al, 2018).

V1.1.3 CDK7

CDK7 has three critical roles in cancer. These are enhanced transcriptional initiation of multiple oncogenes such as c-Myc and upregulation of anti-apoptotic genes such as MCL-1 via phosphorylation of the c-terminal domain of ribonucleic acid (RNA) Polymerase II (Chipumuro et al, 2014; Feaver et al, 1994; Fisher, 2005; Glover-Cutter et al, 2009; Kwiatkowski et al, 2014), rapid progression through the cell cycle via phosphorylation of other members of the CDK family (such as CDK2, 4 and 6) (Fisher, 2005; Fisher and Morgan, 1994; Schachter and Fisher, 2013; Schachter et al, 2013), and loss of sensitivity to hormonal therapy via phosphorylation of ERα (Chen et al, 2000). Please refer to the Investigator's Brochure (IB) for a comprehensive review.

V1.1.4 Overview of Nonclinical Data

The IB provides a comprehensive review and description of all relevant nonclinical study data of CT7001.

Pharmacokinetic studies showed good oral bioavailability in three non-human species (mouse, rat and dog), which predicted good bioavailability in humans. Plasma clearance was high in rats and dogs with a high volume of distribution, resulting in an apparent elimination half-life ($T_{½}$) of 4.8 hours in rats and 9.5 hours in dogs.

Cell growth inhibition studies showed broad activity of CT7001 against a wide range of tumour cell lines, including TNBC (Ainscow et al, 2018; Clark et al, 2017). Encouraging activity was

also observed in vivo, including in patient-derived xenograft models of TNBC (Bahl et al, 2017). Of note, in sensitive in vivo models CT7001 has shown a cytotoxic phenotype with tumour size reduction.

Western blot analysis showed that CT7001 inhibits the phosphorylation of RNA Pol II and Rb, in vitro and in vivo and this in tumour and in normal cells. This suggested that phosphorylated RNA polymerase II (pPolII) could be useful a PDc biomarker in early clinical development.

CT7001 was metabolically stable in human microsomes and hepatocytes in vitro. Intrinsic clearance of CT7001 was high in rat microsomes, intermediate in mouse and dog microsomes, and low in human liver microsomes. Metabolite profiling following incubation of CT7001 with mouse and human hepatocytes showed that the overall turnover of CT7001 was low, indicating the compound has high stability. No evidence of Phase II metabolism (e.g., glucuronidation) was observed.

In a cytochrome P450 (CYP) study using human microsomes, CT7001 strongly inhibited CYP3A4 and showed weak signals for 2D6 and 2C19, with mean half maximal inhibitory concentration (IC_{50}) of 5.7, 35.5, and 44.9 µM, respectively. Mean IC_{50} values for CYP1A2, 2C9, 2B6, 2C8 and 3A5 were greater than 50 µM. In a separate assay, the mean IC_{50} of CT7001 for CYP3A4 was 2.9 and 1.8 µM when using testosterone or midazolam as substrates, respectively.

Based on the in vitro data, CT7001 is unlikely to cause clinically significant hepatic drug interactions through inhibition of CYP1A2, 2C9, 2B6, 2C8 and 3A5. However, there is a potential for inhibition of intestinal and hepatic CYP3A4 which may be clinically significant for CYP3A4 substrates. CT7001 has shown weak potential to inhibit CYP2D6 and 2C19 which is unlikely to be clinically significant but should be considered when co-medicating with 2D6 and 2C19 substrates.

A phenotyping study investigating the metabolism of CT7001 by eight cytochrome P450 enzymes found that CYP mediated clearance of CT7001 mainly occurs via CYP2D6, followed by 3A4 and a small contribution by 2C19. This suggests that co-medications that modulate the activity of CYP 2D6, 3A4 and 2C19 may affect the exposure of CT7001.

The potential of CT7001 to inhibit transporter proteins has not been studied yet. Plasma protein binding is high (>~95%), with a similar unbound fraction in all 3 species tested (rat, dog and human).

Daily administration of 100 mg/kg/day CT7001 to Han Wistar rats for 7 days was well tolerated. Microscopic findings seen in the GI tract and testes suggest an effect of CT7001 on rapidly dividing cells, likely a result of the pharmacological action of the test article.

The daily administration of 15 or 60 mg/kg/day CT7001 to rats and 20 mg/kg/day CT7001 to beagle dogs for 7 days was also well tolerated with no unscheduled deaths or macroscopic or microscopic changes. However, food consumption was reduced over the dosing period; subsequent moistening of the food showed increased consumption. A significant decrease in reticulocyte numbers was observed. Erythroid enucleation is believed to be dependent on the activity of transcriptionally active CDKs, including CDK7 (Wölwer et al, 2015). Therefore, the reduction in reticulocytes was interpreted as a pharmacological effect of CT7001 and considered as another PDc biomarker of potential utility in early clinical development of CT7001.

The main effects of toxicological significance produced by CT7001 in both rats and dogs were manifest in tissues with rapidly dividing cells, namely bone marrow, lymphoid tissue and the gastro-intestinal tract. All effects partially or fully reversed within 4 weeks from cessation of dosing, and the effects produced are consistent with the pharmacological mode-of-action of the drug. CT7001 is not phototoxic.

CT7001 showed no inhibition of human ether-a-go-go-related gene (hERG) channel tail current in a test using the whole-cell patch-clamp technique (human embryonic kidney [HEK] cells transfected with hERG), and the CT7001 IC50 in the K^+ channel was determined to be greater than 5 μM.

Cardiovascular toxicity potential was also studied in vivo in dogs. Oral administration of 5, 15, and 20 mg/kg CT7001 had no effect on haemodynamic parameters, electrocardiogram (ECG) parameters, or body temperature in the dog, compared with control article (vehicle) administration.

Effects of CT7001 on the central nervous system (CNS [Irwin]) and respiratory systems were assessed in rats. No significant effects were observed in the test article groups.

CT7001 has low mutagenic potential. At concentrations up to the lower limit of toxicity, it induced no mutations in a five strains AMES Test. Low potential for genotoxicity was demonstrated in micronucleus tests with human peripheral blood lymphocytes.

V1.1.5 Current Clinical Data of CT7001

A first-in-human multi-module Phase I/II clinical study commenced in November 2017 (EudraCT number: 2017-002026-20; ClinicalTrials.gov identifier: NCT03363893). This is the only clinical study conducted to date.

The single- and multiple-ascending dose Phase 1a part of the study (Module 1, Part A) is close to completion. As of 26-Nov-2018, 32 patients have been dosed and five dose cohorts have completed enrolment (120, 240, 480 mg and a step-down dose level of 360 mg, all taken once daily (OD), and 180 mg given twice a day (BID), all in a fasted state). This module includes a separate cohort with paired BC biopsies pre-dose and on study, with an additional 6 patients dosed to date.

Module 4 is evaluating the effect of food on CT7001 bioavailability in cancer patients, with 8 patients dosed as of 26-Nov-2018. Two single doses of 120 mg are given for testing of food effect. Patients continue then on daily dosing, which originally was 240 mg and has changed recently to 360 mg after determination as recommended Phase 2 dose in Module 1A.

Table 1 provides a summary of the current enrolment and dosing status in Modules 1A and 4.

Table 1: Status of Modules 1A and 4 as of 26-Nov-2018

Cohorts	Dose	Recruited	Dosed	Ongoing
Cohort 1	120mg OD	8	6	0
Cohort 2	240mg OD	7	7*	0
Cohort 3	480mg OD	8	6	0
Cohort 4	360mg OD	6	6	1 (Cycle 7)
Cohort 5	180 mg BID	9	7	1 (Cycle 2)
Cohort with paired breast cancer biopsies	240 mg OD	9	5	1 (Cycle 5)
	360 mg OD	1	1	0
Food effect study	120 → 240 mg OD	9	8	8 (Cycle 3)
**TOTAL**		**57**	**46**	**11**

* Includes 1 replacement patient from Cohort 3 who had a starting dose of 240 mg

An expansion cohort of CT7001 at 360 mg OD in triple negative breast cancer (TNBC) is scheduled to start patient recruitment in December 2018 (Module 1, Part B). This is an open-label, uncontrolled Phase Ib study in patients with metastatic or locally advanced TNBC and documented disease progression who had previously received a taxane and an anthracycline for early or metastatic breast cancer and at least one line of chemotherapy for metastatic disease. The primary objective of the study will be to further characterize the safety of CT7001 and determine the definitive RP2D. A key secondary objective of the study is to explore the efficacy of CT7001 as monotherapy in this population. In case the observed objective response rate may exceed 30% in approximately 30 patients treated at the definitive RP2D, paired with good duration of response and acceptable safety, the study may expand to a Phase IIb study enrolling up to 130 patients..

The subsections below provide a synopsis of the clinical data as recorded in the database as of 26-Nov-2018. Please refer to the IB for detailed information. The data reported here and in the IB is to be viewed as preliminary as study CT7001_001 is ongoing, information continues to be rapidly evolving and the database has not been locked and thus most data has not been cleaned yet.

V1.1.5.1 Safety

As of 26-November-2018, safety data were available from 38 dosed patients, all receiving CT7001 as monotherapy in a fasted state.

In general, CT7001 has shown good safety and acceptable tolerability. Most frequently recorded adverse effects were diarrhoea, nausea and vomiting, which occurred in more than 70% of patients across dose levels, largely at Grade 1. There was no apparent relationship with dose or with blood concentrations of CT7001 (Cmax, AUC or trough levels).

Diarrhoea is an expected target-related adverse effect. Vomiting started usually a few hours after administration of CT7001. It cannot be excluded as this is related to Cmax. This prompted

the exploration of BID dosing in Cohort 5 which however, did not appear to reduce the incidence of vomiting or of nausea. A current working hypothesis is that the main cause of vomiting as well as nausea may be due to the local chemical irritation of the gastric mucosa; taking the drug after a meal may ameliorate these effects. Therefore, Module 4 (which is evaluating the impact of food on the bioavailability of CT7001) was brought forward in the development program. The first set of PK data is expected in Dec-2018. In the event of the PK data from the first or subsequent larger data sets will demonstrate that food has no clinically relevant effect on the bioavailability of CT7001, the study protocol includes a provision that the Data Monitoring Committee may then allow taking CT7001 after a meal. Until such point CT7001 must be taken in a fasted state, as per the instructions provided in the protocol (Section 6.4). The therapeutic or preventive use of common anti-emetics had a positive effect on vomiting and/or nausea in some patients but little effect in others.

Laboratory abnormalities were uncommon. Of note, only 1 event of neutropenia (Grade 1 at 240 mg OD) was recorded. There was 1 event of Grade 4 thrombocytopaenia (at 180mg BID), which was associated with minor nose bleeding and fully reversible upon discontinuation of CT7001.

Thirteen serious adverse events were reported, However, only two were attributed to CT7001 by the respective investigator. They both concerned thrombocytopenia in the same patient (M1A01C501). The case of Grade 4 thrombocytopenia was reported to regulatory authorities as serious unexpected adverse drug reaction (SUSAR). No death occurred which investigators attributed to study therapy.

Dose-limiting toxicities (DLTs) were only recorded at the non-tolerated dose of 480 mg OD (5 events) and on 180 mg given BID (1 event). These included 4 events which were defined as DLT in the study protocol. These were 1 case each of CTCAE Grade 3 diarrhoea, oral mucositis and vomiting at the non-tolerated dose of 480 mg OD, and 1 case of Grade 4 thrombocytopenia at 180 mg BID. The Safety Review Committee judged two additional events to amount to a DLT on clinical grounds. One event was Grade 2 nausea and the other Grade 1 vomiting, each recorded at 480 mg OD in the same patient.

All adverse effects which investigators attributed to CT7001 were reversible upon interruption or discontinuation of CT7001.

V1.1.5.2 Pharmacokinetics (PK)

Preliminary PK data based on uncleaned data and nominal sampling time are available from Study CT7001_001 for 120, 240, 360 and 480 mg OD doses and 180 mg BID dose (only single dose PK data available or 180 mg BID cohort). After oral administration CT7001 was rapidly absorbed with median time to maximum observed plasma concentration (T_{max}) ranging from 1.5 to 4 hours across the cohorts. The absorption phase for CT7001 is characterised by double peaking in some subjects, plasma concentrations then undergo a bi-phasic decline. In the earlier cohorts (doses of 120, 240 and 480 mg), plasma samples were only taken for 48 hours after administration of the single oral dose. The fitted geometric mean elimination half-life for these cohorts ranged from ~ 30 to 38 hours and while the fit had a high correlation coefficient the span of the fit was only over approximately one half-life. Consequently, the sampling regime was extended to 168 hours after single dose and 4 of the 6 subjects dosed 360 mg experienced the longer sampling regime and all the subjects dosed 180 mg BID experienced the longer sampling regimen. For these subjects the determined half-life ranged from 59,3 to 85.7 hours and 54.8 to 82.3 hours for the 360 mg and 180 mg doses respectively. This indicates that the

initial sampling period may underestimate CT7001 half-life. If half-life is underestimated other PK parameters that are dependent on half-life will be poorly estimated (e.g. $AUC_{0\text{-inf}}$, Cl/F, V_z/F, TPC). Based on visual observation of trough concentrations CT7001 steady-state was achieved with 8 to 15 days of daily dosing. Plasma exposure appeared to increase dose-proportionally after single and multiple dosing. The geometric mean accumulation ratio at steady-state ranged from 2.1 to 3.8 across the cohorts.

V1.1.5.3 Pharmacodynamics (PDc)

PD data is currently available from surrogate normal cells. Data from paired tumour biopsies is pending. In normal cells, two PDc effects of CT7001 were evaluated, a biochemical and a biomechanical effect of CDK7 inhibition.

Based on the rationale and non-clinical data described in Sections V1.1.4, V1.1.5, phosphorylated RNA polymerase II (pPolII) in peripheral blood mononuclear cell (PBMCs) was used as biochemical PDc biomarker. Figure 1 illustrates the significant reduction of pPolII by ~30% induced by CT7001 in PBMC across all dose levels tested in Module 1A for subjects who have completed 21 days of treatment (i.e., cycle 2 day 1 timepoint). Later timepoints also show inhibition, although not reaching statistical significance due to the low number of patients.

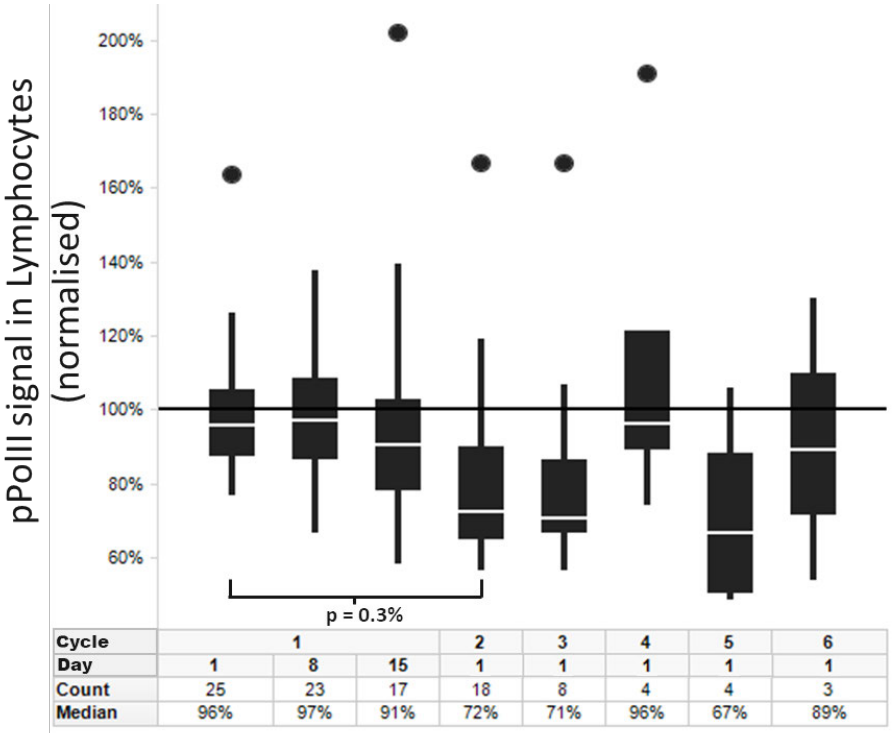

Figure 1: CT7001 decreases pPolII levels in PBMC.

pPolII was determined by a flow cytometry assay on patient samples of PBMC preparations derived from blood. The data show box plots for each sampling timepoint normalised to the screening sample for that subject. Note that samples were taken pre-dose at all timepoints. Statistical comparison between pre-treatment samples (Cycle 1 day 1) and subsequent timepoints were done using a 1-tailed Mann-Whitney test with Bonferonni correction.

Enumeration of reticulocyte counts was used as a biomechanical PDc marker, based on the mechanistic rationale and non-clinical data described in Section V1.1.4. Across all dose levels tested in Module 1A, CT7001 has produced a significant decline in reticulocyte counts (Figure 2). Subjects who came off treatment for unrelated reasons have shown a subsequent increase of reticulocyte counts by 25% (p = 0.08), demonstrating that the effect on reticulocytes is drug-induced and reversible. Of note, across dose levels and cycles anaemia has been uncommon to date (see IB). However, it is to be recognized that only few Module 1A patients have stayed on CT7001 for four months or longer.

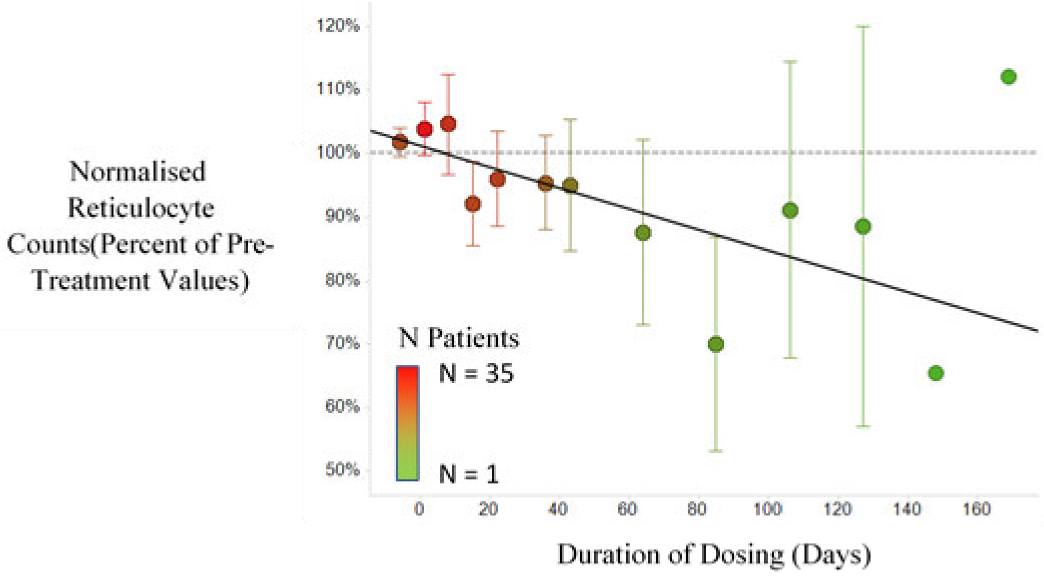

Figure 2: Average reticulocyte counts across first three dosing cohorts (120, 240 & 480 mg).

Reticulocyte counts for subjects across the three cohorts were monitored over time. Values on treatment were normalized to the average value observed for each patient from screening and pre-treatment cycle 0 samples. Data are mean ± standard error of the mean for between n=35 and n=1 samples (denoted by symbol color). Linear regression analysis (solid line) showed a significant decrease in normalized reticulocyte count over time on treatment (p=0.2).

V1.1.5.4 Minimum Biologically Active Dose (MBAD)

The study's SRC originally declared 240 mg OD as MBAD based on observed target toxicity (diarrhoea), target-mediated PDc effect on reticulocyte counts and preliminary signals of anti-tumour activity. This prompted the opening of a cohort with paired breast cancer biopsies at 240 mg OD. Later review of the data from cohort 1 at the starting dose of 120 mg OD revealed similar biological activity as observed for 240 mg OD, including PDc and anti-tumour effects. Accordingly, 120 mg OD represents the lowest dose tested to date in humans that has demonstrated biological activity.

V1.1.5.5 Non-Tolerated Dose

At 480 mg, 3/6 patients experienced a dose-limiting toxicity (1 case each of Grade 3 diarrhoea, mucositis or vomiting). Furthermore, 3 additional subjects experienced G1-2 vomiting with little effect of anti-emetic therapy. In one of these subjects, the SRC judged this to amount to a

DLT, on clinical grounds.As a result, the study's SRC defined 480 mg as non-tolerated dose when given OD in a fasted state.

V1.1.5.6 Maximum Tolerated Dose (MTD) and Preliminary Recommended Phase 2 Dose (RP2D)

Six subjects in Cohort 4 (360 mg OD in fasted state) have completed Cycle 1 without recording a DLT or other concerning safety or tolerability findings. Accordingly, a dose of 360 mg OD given in a fasted state has been determined as the MTD and the preliminary RP2D for further characterization and investigation of CT7001 as monotherapy. Accordingly, this is the dose which has been taken forward to Module 1B, the phase 1b expansion cohort in TNBC, and this is the target dose for use in combination with fulvestrant in Module 2.

V1.1.5.7 Anti-Tumour Effects

The latest current cut-off for efficacy evaluation was 24-September-2018, with 26 patients evaluable. Three patients had stable disease for ≥18 weeks, including 1 patient at 240 mg with castrate-refractory prostate cancer for 27 weeks and 2 patients with colorectal cancer at 120 mg for 18 weeks each (1 patient with a best change in lesion size of -21% on CT scan).

Five patients had stable disease for ≥12 weeks (3 ongoing). This included 1 patient at 240 mg with small-cell lung cancer for 15 weeks, 1 patient at 240 mg with oesophageal cancer for 12 weeks, 1 patient at 240 mg with HR-positive BC for 12+ weeks, 1 patient at 360 mg with castrate-refractory prostate cancer for 12+ weeks and 1 patient at 360 mg with chondrosarcoma for 12+ weeks.

Among the patients with stable disease, three had a decline in blood biomarkers, 2/2 patients with castrate-refractory prostate cancer (decline in PSA (prostate specific antigen) by 45% and 30%, respectively), and 1 patient with HR-positive BC (decline in cancer antigen CA 15-3 by 30%).

V1.1.5.8 Summary of Current Clinical Experience

The current clinical experience of CT7001 has shown good safety and PK behavior, positive PDc effects in surrogate normal cells and preliminary signs of anti-tumour effect; warranting further clinical investigation.

V1.2 CORE STUDY OBJECTIVES AND ENDPOINTS

V1.2.1 Core Study Objectives

Primary Objectives

- To investigate the safety and tolerability of CT7001 given alone or in combination with anti-cancer treatments.

Secondary Objectives

- To characterise the PK of CT7001, given alone or in combination with anti-cancer treatments, after a single dose and at steady state after multiple dosing.

- To assess the biological and anti-tumour activity of CT7001, given alone or in combination with anti-cancer treatments.

Exploratory Objectives

- To investigate the presence and identity of metabolites of CT7001 and, if appropriate, characterise their PK.

- To explore the relationship between PK and safety, antitumour activity, and biological activity and the impact of patient characteristics on PK.

- To collect and store DNA for future exploratory research into genes and genetic variation that may influence response to CT7001 (i.e., distribution, safety, tolerability, and efficacy).

- To collect and store predose plasma and serum samples and archival tumour tissue, if available, for potential future exploratory research into factors that may influence the development of agents to treat human disease or response to CT7001 (i.e., distribution, safety, tolerability. and efficacy).

- To investigate predictive markers and acquired resistance to CT7001 that may be observed in ctDNA.

- To investigate predictive markers and acquired resistance to CT7001 that may be observed in tumour tissue.

V1.2.2 Core Study Endpoints

Core Primary Endpoints

This section describes the study endpoints common to every module of the study. Endpoints specific to a module are described within the respective module-specific section of this protocol.

- AEs.

- Clinical laboratory results (haematology, serum chemistry, coagulation, urinalysis).

- Physical examination findings.

- ECOG performance status.

- ECG parameters (heart rate, PR interval, QRS complex, QT interval, and QTcF).

- Weight.

- Vital signs (supine systolic and diastolic blood pressure and pulse, temperature respiratory rate, oxygen saturation).

Core Secondary Endpoints

- PK Parameters for CT7001
- Biological Activity Parameters (Biomarkers)
- Anti-tumour Activity

Core Exploratory Endpoints

- PK Parameters for Major Metabolites:
  - PK Metabolite:CT7001 ratio
  - Metabolite identification.
- Predictive Markers and Acquired Resistance to CT7001:
  - Total and Phosphorylated CDK7
  - Myc oncogene expression.

V1.3 CORE PATIENT SELECTION

Each Investigator must keep a record (i.e., a patient screening log) of patients who are screened for every module of this study.

Patients must meet all of the following core study inclusion criteria and none of the exclusion criteria to enrol in this study. Additional module-specific inclusion/exclusion criteria may be defined within the relevant volumes of this protocol. Patients who do not meet the core eligibility criteria and all other criteria specific for the relevant module should not under any circumstances be enrolled in this study or receive investigational medicinal product (IMP). There can be no exceptions to this rule.

If a patient who does not meet the eligibility criteria is enrolled in error or started treatment, the Investigator will inform Emas Pharma Ltd. (the Contract Research Organisation [CRO]), Medical Monitor immediately, and the Emas Medical Monitor will ensure all such contracts are appropriately documented. If the patient has not started treatment, he/she will be withdrawn the study after completion of follow-up safety assessments.

If the patient has started on treatment in any particular module, the SRC will conduct an individual benefit/risk assessment, and if the SRC assesses that the patient is receiving clinical benefit, the Investigator may choose to continue the patient in the study. If the SRC assesses that the patient is not receiving clinical benefit, then the patient must be withdrawn from the study after completion of follow-up safety assessments.

V1.3.1 Core Inclusion Criteria

Patients who meet all of the following criteria may be included in the study (providing any module-specific criteria are also met):. **Check each Module for any specific/additional criteria.**

1. At least 18 years of age.
2. ECOG performance status 0 or 1 with no deterioration over the previous 2 weeks.
3. Estimated life expectancy of greater than 12 weeks.
4. Ability to swallow and retain oral medication.
5. Women of childbearing potential must practice effective contraception. This includes:
    - Abstinence if consistently employed as the patient's preferred and usual lifestyle ,
    - Sex only with person of the same sex or with vasectomised partner
    - Medroxyprogesterone injections [e.g., Depo-Provera], levonorgestrel intrauterine system [e.g., Mirena], intrauterine device (IUD), or barrier method [e.g., condom, diaphragm] for the duration of the study and for 6 months after the last dose of CT7001.
    - **Note:** Contraceptives that are prone to drug-drug interactions may not be effective because of a potential CYP3A4 interaction with CT7001. Furthermore, some hormonal contraceptives are contra-indicated in certain populations, please refer to the specific module contraception requirements.
6. Women not of childbearing potential is defined as women who are postmenopausal (defined as ≥50 years of age, amenorrhoeic for at least 12 months after cessation of all exogenous hormonal treatments, and have serum follicle-stimulating hormone, luteinizing hormone (LH) and plasma oestradiol levels in the testing laboratory's postmenopausal range). Women under 50 years old would be considered postmenopausal if they have been amenorrhoeic for 12 months or more following cessation of exogenous hormonal treatments and with LH and follicle stimulating hormone levels in the post-menopausal range for the institution or women who have been amenorrhoeic for at least 12 months and are less than 50 years of age. Women who are surgically sterilised (defined as documented irreversible surgical sterilisation by hysterectomy or by bilateral tubal ligation, oophorectomy, or salpingectomy).
7. Sexually active male patients must be willing to use condoms with all sexual partners for the duration of the study and for 3 months after the last dose of CT7001. If a female partner is a woman of childbearing potential who is not using effective contraception, the patient must use a condom with spermicide during the study and for 6 months after the last dose of CT7001.
8. Provision of signed and dated, written informed consent before any study-specific procedures, sampling, or analyses, including access to archival tumour tissue.

Host Genetics Research Study: Pharmacogenetics Samples (Optional)

Patients who meet all of the following criteria may be included in optional genetics substudies:

1. Provision of signed and dated, written informed consent for the genetic research.

V1.3.2 Core Exclusion Criteria

Patients who meet any of the following criteria will be excluded from the whole study:

1. Any other malignancy which has been active or treated within the past 3 years, with the exception of cervical intraepithelial neoplasia and non-melanoma skin cancer.
2. Any unresolved toxicity (except alopecia) from prior therapy of ≥ Grade 2 according to CTCAE.
3. Spinal cord compression or brain metastases, unless asymptomatic, stable, and not requiring steroids for at least 4 weeks before the first dose of IMP (if stable and requiring no intervention, the patient can be enrolled in the study).
4. Refractory nausea and vomiting, chronic GI diseases or previous significant bowel resection, with clinically significant sequelae that would preclude adequate absorption of CT7001.
5. Uncontrolled seizures.
6. Active infection requiring systemic antibiotic, antifungal, or antiviral medication.
7. Severe or uncontrolled medical condition (e.g., severe chronic obstructive pulmonary disease, severe Parkinson’s disease, active inflammatory bowel disease) or psychiatric condition.
8. Active bleeding diatheses.
9. Renal transplant.
10. Known hepatitis B, hepatitis C, or human immunodeficiency virus infection.
11. Breastfeeding or pregnancy.
12. Receipt of cytotoxic treatment for the malignancy within 28 days before the first dose of IMP.
13. Receipt of noncytotoxic treatment for the malignancy within 5 half-lives of the drug before the first dose of IMP.
14. Receipt of corticosteroids (at a dose > 10 mg prednisone/day or equivalent) within 14 days before the first dose of IMP.
15. Receipt of any small-molecule IMP within 28 days before the first dose of IMP.
16. Receipt of any biological IMP (e.g., immune checkpoint blockers, antibodies, nanoparticles) within 42 days before the first dose of IMP.
17. Receipt of St John’s Wort within 21 days before the first dose of IMP or of another concomitant medication, herbal supplement, or food that is a strong inhibitor or inducer of CYP3A4, CYP2C19, CYP2D6, or P-glycoprotein (PGP) activity within 14 days before the first dose of CT7001 (see APPENDIX B).

18. Receipt of a blood transfusion (blood or blood products) within 14 days before the first dose of IMP.

19. Known hypersensitivity to CT7001 or any excipient of the product.

20. Impaired hepatic or renal function as demonstrated by any of the following laboratory values:

    - Albumin < 30 g/L.
    - Aspartate aminotransferase (AST) or alanine aminotransferase (ALT) > 2.5 × the upper limit of normal (ULN).
    - > 5.0 × ULN for patients with liver metastases.
    - Total bilirubin > 1.5 × ULN.
    - Serum creatinine > 1.5 × ULN.
    - International normalised ratio (INR) ≥ 1.5.

21. Liver function deteriorating in a manner that would likely make the patient meet the AST, ALT, or bilirubin levels specified above in at the time of the first dose of IMP.

22. Other evidence of impaired hepatic synthesis function.

23. Inadequate bone marrow reserve or organ function as demonstrated by any of the following laboratory values:

    - Absolute neutrophil count (ANC) < 1.5 × 10^9/L.
    - Platelet count < 100 × 10^9/L.
    - Haemoglobin < 90 g/L.

24. Persistent (> 4 weeks) severe pancytopenia due to previous therapy rather than to disease (ANC < 0.5 × 10^9/L or platelets < 50 x 10^9/L).

25. Cardiac dysfunction (defined as myocardial infarction within 6 months of study entry, New York Heart Association Class II/III/IV heart failure, unstable angina, unstable cardiac arrhythmias, or left ventricular ejection fraction < 55%).

26. Mean resting QT interval corrected for heart rate by the Fridericia formula (QTcF) > 470 msec obtained from three ECGs obtained within 5 minutes of each other prior to the first dose

27. Any clinically important abnormalities in rhythm, conduction, or morphology on resting ECG (e.g., complete left bundle branch block, third degree heart block). Controlled atrial fibrillation (AF) is permitted.

28. Any factor that increases the risk of QTc prolongation or of arrhythmic events (e.g., heart failure, hypokalaemia, congenital long QT syndrome, immediate family history of long QT syndrome or unexplained sudden death under 40 years of age).

29. In the opinion of the Investigator, unlikely to comply with study procedures, restrictions, or requirements.

30. A history of haemolytic anaemia or marrow aplasia.

31. Has received a live-virus vaccination within 28 days or less of planned treatment start. Note: seasonal flu vaccines that do not contain live virus are permitted.

Host Genetics Research Study: Pharmacogenetics Samples (Optional)

Patients who meet any of the following criteria will be excluded from optional genetic substudies:

1. Allogenic bone marrow transplant.

2. Non-leucocyte-depleted whole blood transfusion within 120 days of the date of the genetic sample collection.

V1.4 CORE STUDY DESIGN AND RATIONALE

V1.4.1 Study Design

This is a modular, Phase I/IIa study aims to investigate the optimal dose of CT7001 when used as monotherapy dose or in combination with other anti-cancer treatments. (Volume 1)

The individual modules have the following main objectives:

- **Module 1 Part A**: To evaluate the safety, tolerability, PK and PDc effects of CT7001 as monotherapy and to determine the minimally biologically active dose (MBAD) and maximum tolerated dose (MTD) in patients with advanced solid malignancies. (Volume 2)
- **Module 1 Part B**: To refine the safety, tolerability, PK and PDc profiles of CT7001 in subjects with advanced solid malignancies, potentially including TNBC, SCLC, CRPC, Ovarian cancer subjects and other appropriate cancer indications (Volume 2)
- **Module 1 Part B-1 (Cohort 1 – TNBC):** To determine the recommended Phase 2 dose of CT7001 as monotherapy, further characterize safety, tolerability and blood concentrations of CT7001, and explore its anti-tumour activity in triple negative breast cancer (Volume 3)
- **Module 2:** To evaluate the tolerability of the combination of CT7001 and fulvestrant in patients with advanced hormone receptor positive breast cancer (Part A), compare the efficacy CT7001 combined with fulvestrant versus fulvestrant alone (Part B) and evaluate the efficacy of CT7001 combined with fulvestrant in patients whose disease had progressed on or after fulvestrant plus placebo in Part B of the study (Part C). (Volume 4)

- **Module 3:** To evaluate of the safety, tolerability, and antitumour activity of CT7001 as monotherapy in patients with acute myeloid leukaemia (AML). (Volume 5)
- **Module 4:** To explore the effect of food on the total and peak exposure of CT7001, when given as monotherapy to patients with advanced solid malignancies. (Volume 6)
- **Module 5:** To evaluate CT7001 in combination with anti-PD-1 (nivolumab or pembrolizumab) or anti-PD-L1 (atezolizumab or durvalumab) monoclonal antibodies (mAb) in patients with advanced solid malignancies. (Volume 7)

Key Definitions referred to in all Modules:

Maximum Tolerated Dose (MTD)

A dose will be considered non-tolerated and dose escalation will cease if two or more of up to six evaluable patients experience a DLT at a dose level. Once the non-tolerated dose is defined, the MTD will be confirmed at the previous dose level below the non-tolerated dose, or a dose between the nontolerated dose and the last tolerated dose may be investigated. Six evaluable patients are required to determine the MTD. The MTD will be determined and agreed by the SRC with the Sponsor (see SRC remit document). All investigator sites will attend the SRC such that updates on toxicity and MTD will be communicated immediately. No more than six patients can be recuited at a non-tolerated dose level.

Minimally Biologically Active Dose (MBAD)

The MBAD is defined as the smallest amount of drug needed to produce the desired or specified effect. This predicted minimal efficacious human dose is based on scaling factors from mouse to human.

Maximum Feasible Dose (MFD)

The maximum dose that is feasible to deliver to a patient at one administration, due to the tolerability associated with the number and or bulk of the capsules that need to be swallowed.

Study components that are core to the whole study (including all modules) are discussed in this section of the Protocol. Components specific to Module 1 Part A and Part B are discussed in Volume 2 and additional later Modules 1 Part B-1 Cohort 1 (TNBC), 2, 3, 4 and 5 are discussed further in Volume 3, Volume 4, Volume 5, Volume 6 and Volume 7, respectively. The risk benefit profiles of each module are discussed in the relevant sections. Each protocol amendment and/or module addition will be approved before starting each new module.

The initial dosing schedule, frequency or sequence of CT7001 in each module may subsequently be changed between cohorts in response to emerging safety, PK, and PDc findings in previous modules. If the dosing frequency/schedule is changed, the initial total daily dose, of the new dosing frequency/schedule, will not exceed the current maximum total daily dose that has been explored and found to be tolerable; this change can be made without the requirement of a substantial protocol amendment. Rationale will be described in each new module following the relevant protocol amendment. The CT7001 MBAD and MTD for individual modules may differ depending on the emerging safety profile for each treatment combination.

In all combinations, the dose of each combination agent investigated will not exceed its current recommended dose. For cohorts in which CT7001 is dosed in combination with cytotoxic chemotherapy, dosing will not continue once the cycles of chemotherapy have been completed.

V1.4.2 Rationale for Study and Study Design

The mechanism of action of CT7001 provides the potential for both monotherapy anti-tumour activity and combination with a number of anti-cancer treatments, resulting in either synergistic or additive activity, in patients with solid or haematological malignancies.

This study is modular in design, allowing evaluation of the safety, tolerability, PK and anti-tumour activity of CT7001 at increasing doses when administered alone and in combination with anti-cancer agents in patients with advanced malignancies. The modular study design allows an investigation of the optimal monotherapy dose and combination doses of CT7001 with other anti-cancer treatments, with intensive safety monitoring to ensure the safety of the patients.

Key aspects of the whole study, such as the starting dose of CT7001 in Module 1 and the dose escalation, stopping criteria, and cohort size of all study modules have been determined in accordance with established methodology for Phase I oncology studies (Ivy et al, 2010; ICH harmonised tripartite guideline: Nonclinical evaluation for anticancer pharmaceuticals S9, 2009; EMA guideline on strategies to identify and mitigate risks for first-in-human and early clinical trials with investigational medicinal products, 2017). Study criteria that are core to all modules are presented in this section of the protocol. Additional module-specific criteria are presented in the relevant later appendices as appropriate.

As part of the clinical drug development programme for CT7001, Carrick plans to include investigations into variations in PDc and exploratory biomarker profiles and their relationship to drug effect; these biomarkers may be derived from DNA, and/or metabolites. Specific biomarkers to be analysed are detailed within the respective study module section. There are many potential benefits of this exploratory research, including the possibility to identify patients most likely to benefit from treatment, explain outliers or non-responders, or explain adverse reactions related to drug exposure. This research may result in an understanding of the impact of variation between individuals and how this information can be utilised to bring better drugs to the clinic.

The results from this study will form the basis for decisions for future studies using CT7001.

V1.5 STUDY TREATMENT

V1.5.1 Drug Supply

The investigational product used in this trial is called CT7001, will be supplied by the sponsor.

V1.5.1.1 Pharmaceutical Properties

Chemical (IUPAC) name:

(3R,4R)-4-[[[7-(benzylamino)-3-isopropyl-pyrazolo[1,5-a]pyrimidin-5-yl]amino]methyl]piperidin-3-ol

Laboratory Code: CT7001, ICEC0942

Structural formula:

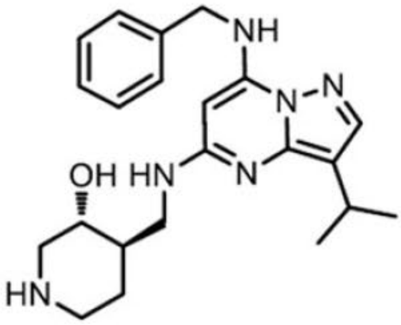

V1.5.1.2 Formulation, Packaging and Storage

CT7001 will be supplied as capsules containing 60 mg or 120mg equivalents of CT7001 free base and the excipients listed in.Table 2.

Table 2: CT7001 Excipients

Material	(%w/w)
CT7001 (drug substance)	30.00
Microcrystalline Cellulose (Avicel PH 102)	64.50
Sodium starch glycolate (Explotab®)	5.00
Magnesium Stearate (Hyqual®)	0.25
Silica Colloidal Anhydrous (Aerosil 200)	0.25
**Total**	100.00

The capsules at each strength are opaque white hydroxypropyl-methylcellulose capsule shells. Capsules with 60 mg are size 1 and capsules with 120 mg size 00, respectively.

The sponsor, through his delegate, will supply the oral drug formulation to sites in high-density polyethylene bottles containing 60 mg or 120 mg capsules. Bottles are secured with a child-resistant and tamper-evident closure.

All IMP will be kept in a secure place under appropriate storage conditions as specified on the IMP label.

V1.5.2 Dispensing

The patient number should be recorded on the bottle label in the spaces provided by site personnel at the time of assignment to patient. Site personnel must ensure that patients clearly understand the directions for self-medication.

Patients should be given a sufficient supply to last until their next study visit. Unused drug and/or empty bottles should be returned to the site at the next study visit.

Patients should be instructed to keep their medication in the bottles provided and not transfer it to any other container.

V1.5.3 Dosing Regimen of CT7001

Module 1A has determined 360 mg given once a day in a fasted state as preliminary RP2D regimen with appropriate safety and tolerability for further investigation. Accordingly, this is the CT7001 dosing regimen used at the start of Module 1B. As in Module 1A, a treatment cycle is defined operationally as 21 days.

For dosing regimens with OD dosing, each daily dose should be taken around a similar time of the day. Which time of the day is at the patient’s discretion.

For potential dosing regimens with BID dosing, the two doses should be taken 9-12 hours apart, each in a fasted state.

V1.5.4 Drug Administration

CT7001 has to be taken orally in a fasted state, with no food or any liquids other than water for 2 hours before and 1 hour after each CT7001 dose.

Module 4 is currently evaluating the effect of food on the bioavailability of CT7001 in cancer patients. In case no significant effect will be observed, the SRC may remove the fasting requirement.

Site personnel must ensure that patients clearly understand the directions for self-medication. Patients should be instructed to swallow CT7001 capsules whole and not to chew them prior to swallowing. No capsule should be ingested if it is broken, cracked, or otherwise not intact.

V1.5.5 General Rules

Patients who miss a day’s dose must be instructed NOT to ‘make it up’ the next day.

Patients who vomit any time after taking a dose must be instructed NOT to 'make it up' but rather resume treatment the next day as prescribed.

V1.5.6 Medication Dosing Errors

Medication dosing errors may mainly result from the administration of CT7001 at the wrong dosage strength. Such medication errors are to be captured on the IP administration electronic case report form (eCRF) and on the SAE form when appropriate. In the event of medication dosing error, the sponsor should be notified immediately.

V1.5.6.1 Overdose

There are no clinical data yet on overdose with CT7001 as this has not occurred to date. There is no definition of what constitutes an overdose. There is no known antidote.

Any patient who receives a higher dose than that intended in the module should be monitored closely, managed with appropriate supportive care, and followed-up expectantly. Such incidence should be recorded as an overdose and will be recorded in the eCRF as follows:

- An overdose with associated AEs will be recorded as an AE of the relevant diagnosis/symptoms on the AE eCRF page and in the overdose eCRF page.
- An overdose with no associated symptoms will be reported only on the overdose eCRF page.
- If an overdose occurs, the Investigator or other site personnel must notify the Emas Medical Monitor immediately but no later than by the end of the next business day of first awareness.

The Emas Medical Monitor will work with the Investigator to ensure that all relevant information is provided to the safety database: drug.safety@bionical-emas.com

For overdoses associated with an SAE, standard reporting timelines apply (see Section V1.3.11.1). Other overdoses will be reported within 28 days.

V1.5.7 Intra-Subject Dose Modification

Every effort should be made to administer study treatment on the planned dose and schedule. However, in the event of significant treatment-related toxicity, administration of CT7001 may need adjustment as described in the following sections.

Patients are to be instructed to notify Investigators at the first occurrence of any adverse sign or symptom. In the event of significant treatment-related toxicity, CT7001 dosing may be interrupted, delayed and/or reduced as described below. In the event of multiple toxicities, dose modification should be based on the worst toxicity observed.

No specific dose adjustments are recommended for Grade 1-2 treatment-related toxicity. However, investigators should always manage their patients according to their best medical judgment based on the particular clinical circumstances.

V1.5.7.1 Types of Dosing Modifications

Dosing may be interrupted (within a cycle) or delayed (at start of a next cycle) and the dose may be reduced.

Note to the following Sections V1.5.7.1.1, V1.5.7.1.2 and V1.5.7.1.3:

- MUST = mandatory
- SHOULD = not mandatory but highly recommended
- MAY = per the investigator's best clinical judgment

V1.5.7.1.1 Dosing Interruption or Delay

The first measure of dose modification is interruption (within a cycle) or delay (at start of a next cycle) of dosing.

Patients experiencing the following adverse events MUST have their treatment interrupted or delayed:

- Grade 3 neutropenia (ANC < 1.0 × 10^9/L) associated with a documented infection or fever ≥ 38.5°C.
- Grade 4 neutropenia (ANC < 0.5 x 10^9/L).
- Grade 4 thrombocytopenia (platelet count < 25 x 10^9/L).
- Grade 3 anemia (Hb < 80 g/L and transfusion indicated).
- Grade ≥ 3 diarrhoea, oral mucositis, vomiting or nausea if persistent despite optimal medical treatment.
- Grade ≥ 3 other non-haematological toxicity if persistent despite optimal medical treatment.
- Grade 3 average QTc prolongation (QTc ≥ 501 msec or > 60 msec change from baseline) corrected for heart rate by the Fridericia formula
- Grade 4 QTc abnormalities (torsades de pointes, polymorphic ventricular tachycardia, signs and/or symptoms of serious arrhythmia)

Appropriate follow up assessments should be performed and proper therapy and medical care, as clinically indicated, should be provided. If a treatment delay results from a decline in haematological parameters, the frequency of laboratory assessments should be increased as clinically appropriate.

V1.5.7.1.2 Restart of Treatment

Restart of treatment within a cycle or start of a next cycle SHOULD occur when the following parameters have been met:

- ANC ≥ 1.0 × 10^9/L, no fever and full resolution of a documented infection
- Platelet count ≥ 50 × 10^9/ L
- Hb ≥ 80 g/L

- Grade ≤ 1 diarrhoea, oral mucositis and vomiting
- Grade ≤ 2 nausea
- QTc < 481 msec corrected for heart rate by the Fridericia formula and potential contributing causes (e.g., electrolyte imbalance, concomitant medications known to prolong QTc) corrected

In case of Grade 4 QTc abnormalities, study therapy MUST be discontinued permanently.

In case recovery of the other toxicities takes more than 21 days, permanent discontinuation of CT7001 SHOULD be strongly considered. Treatment resumption for patients recovering from treatment-related toxicity after more than 21 days of treatment interruption or cycle delay but deemed to be deriving obvious clinical benefit per the investigator's best medical judgment is left at the investigator's discretion.

Depending on when a toxicity resolved, a treatment interruption may lead to the patient missing all subsequent planned doses within that same cycle. Doses omitted for toxicity are not to be replaced within the same cycle.

In the event of a treatment interruption or cycle delay for reasons other than treatment-related toxicity (e.g., non-cancer related surgery) lasting >2 weeks, treatment resumption will be decided in consultation with the sponsor.

V1.5.7.1.3 Dose Reductions

Following dose interruption or cycle delay the dose of CT7001 may need to be reduced when treatment is resumed.

As noted in Section V1.5.7.1.2, in case of Grade 4 QTc abnormalities study therapy MUST be discontinued permanently.

Prior to concluding that an episode of prolongation of the QTc interval is due to study drug, thorough consideration should be given to potential precipitating factors (e.g., change in patient clinical condition, effect of concurrent medication, electrolyte disturbance) and possible evaluation by specialist. If IMP causality cannot be ruled out, dose reduction as described below should be performed upon restart of therapy.

When treatment is resumed after sufficient resolution of the other toxicities listed in Section V1.5.7.1.1, dose reduction SHOULD be considered but is at the investigator's discretion. Factors to take into account include the time it took to recover from a given toxicity, clinical sequelae associated with a toxicity and overall risk-benefit assessment per the investigator's best clinical judgment.

In case the investigator considers a dose reduction indicated for other reasons, this needs prior discussion and agreement with the sponsor.

A maximum of two dose reductions of CT7001 will be allowed per patient. Patients requiring more than 2 dose reductions will be discontinued from the study and entered into the follow-up phase.

V1.5.8 Inter-Patient Dose Modification

Module 1A is still ongoing, with a subset of patients in Cohort 4 (360 mg) continuing to receive study therapy, Cohort 5 investigating CT7001 dosed at 180 mg twice a day and a cohort with paired breast cancer biopsies open for recruitment. In Module 4, which will investigate the effect of food on the bioavailability of CT7001, patients will receive daily dosing of 240 mg OD after completion of the single dose cross-over bioavailability part.

The SRC will meet regularly to review all available data on safety and anti-tumour effects but also all the further data emerging from Modules 1A and 4. In case inter-subject modification of the CT7001 dosing regimen appears indicated, this would prompt a major protocol amendment.

V1.5.9 Compliance

Patients will be required to return all bottles of CT7001 as well as their completed patient diary at the beginning of each cycle for drug accountability. Drug accountability will be performed on Day 1 of every cycle prior to dispensing drug supply for the next cycle. The number of remaining capsules will be documented and recorded.

V1.5.10 Drug Storage and Accountability

Storage conditions stated in the IB may be superseded by the label storage. Investigators and site staff are reminded to continuously monitor room storage temperatures and ensure that thermometers are working correctly as required for proper storage of the investigational product. Temperature excursions must be reported immediately to the sponsor and documented. Once a deviation is identified, the investigational product (CT7001) MUST be quarantined and not used until the sponsor provides documentation of permission to use the investigational product.

At the end of the trial or at the close-out of the site, any unused investigational product will be destroyed. If the destruction occurs at the trial site, the investigator must ensure that the materials are destroyed in compliance with applicable environmental regulations, institutional policy, and any special instructions provided by the sponsor. Destruction must be adequately documented. Alternatively, investigational product maybe shipped to a local depot for destruction.

To ensure adequate records, CT7001 capsules will be accounted for as instructed by the sponsor. Patients are required to return previously dispensed containers as well as their completed patient diary to the clinic at each visit for accountability purposes even if they will not be issued with new medication at that visit.

V1.5.11 Concomitant Medications

If medically reasonable and feasible, subjects taking regular medication (with the exception of strong inhibitors or inducers of CYP3A4, CYP2C19, CYP2D6, or PGP (see APPENDIX B) should be maintained on it throughout the study/module.

Patients must be instructed not to take any new medications (over-the-counter or other products) during the study without prior consultation with the investigator. Medications that are considered necessary for the subject's safety and well-being may be given at the discretion of the Investigator.

Any medications including herbal supplements, vitamins or medicines taken by the patient from 28 days prior to the start of study treatment and up to 28 days following the last dose of investigational product and the reason for their administration must be recorded on the eCRF.

Routine postoperative care, such as dressing changes, suture removal, drain removal, or venous access (central or peripheral), does not need to be recorded. Anaesthetics used for any surgical procedures performed during the patient's participation in the study can be recorded as "unspecified anaesthesia" on the concomitant treatment records; it is not necessary to list the specific anaesthetics.

Appropriate palliative and supportive care for cancer-related symptoms will be offered to all patients in this study.

V1.5.11.1 Prohibited Medications

The following treatments are prohibited throughout the duration of the active treatment phase:

- **Anti-cancer agents**: No additional investigational or commercial anti-cancer agents such as chemotherapy, immunotherapy, targeted therapy, biological response modifiers or endocrine therapy will be permitted during the active treatment phase. In general, any drugs containing "for the treatment of 'breast' cancer"" or other indications specific to additional cohorts, on the product insert are not permitted on study.
    - Patients may receive bisphosphonates or denosumab for the treatment of bone metastases during participation in the study.
    - Patients with prostate cancer or pre/peri menopausal breast cancer may receive goserelin.
- **No investigational product other than CT7001**.
- **Blood transfusions** are not allowed within 14 days before the first IMP dose.
- **Live virus or bacterial vaccines** (e.g., yellow fever, measles, influenza, rubella, mumps, typhoid, mycobacterium tuberculosis [BCG], Yersinia pestis [EV] vaccines).
    - An increased risk of infection by the administration of these vaccines has been observed with conventional chemotherapy and the effects with CT7001 are unknown.
        - Live vaccines must not be administered until 3 months after the last dose of IMP.
        - If the live vaccine induces B-cell depletion then they should not be administered until 6 months after the last dose of IMP (Rubin et al, 2013).
    - Administration of the live flu vaccine is allowed if given at least 28 days prior to start of screening.
    - The administration of killed vaccines (e.g., cholera, bubonic plague, non-live influenza, polio, hepatitis A, and rabies vaccine) is allowed.

V1.5.11.2 Medications Not Recommended

The following treatments are not recommended throughout the duration of the active treatment phase. Alternative therapies should be considered whenever possible. If the investigators deemed usage of the following treatments necessary, consultation and agreement with the sponsor is required prior to initiation of treatment.

- Medications, herbal supplements, and foods that are strong inducers or inhibitors of CYP3A4, CYP2C19, CYP2D6 or PGP (see Appendix B for a listing) should be avoided from 14 days before the first dose of CT7001 until 28 days after the last dose of CT7001.
    - **Note:** St. John's Wort should be avoided from 21 days before the first dose of CT7001.
    - Aprepitant (Emend®) is a substrate, moderate inhibitor and inducer of CYP3A4 and an inducer of CYP2C19. Therefore, aprepitant should not be used in this study for the treatment of nausea and vomiting.
    - If the Investigator feels that concomitant administration of such medications, herbal supplements or foods is necessary based upon medical judgment, such products may be administered with caution following discussion between the Investigator and the Carrick Therapeutics Physician or CRO medical monitor.
        - CT7001 is an investigational drug for which no in vivo data on drug interactions are currently available.
        - Patients taking concomitant medications whose disposition is dependent upon CYP3A4 and which have a narrow therapeutic index should be closely monitored for signs of changed tolerability.
        - CT7001 shows a weak potential to inhibit CYP2D6 and 2C19, which should be considered when co-prescribing 2D6 and 2C19 substrates.
- CT7001 exhibits pH-dependent solubility. As such there is a risk that agents that increase gastric pH (such as PPIs, H2 antagonists) may affect the bioavailability of CT7001 and should be avoided in the study if possible. However, if clinically required, they may be prescribed. Such patients should be monitored for signs of changed CT7001 activity.
- Chronic immunosuppressive therapies should be avoided, including systemic corticosteroids.
    - Steroids given for physiological replacement, as anti-emetics or inhaled as well as short course of oral or topical steroids given for allergic reactions or asthma flares are allowed.
- Drugs known to predispose to Torsades de Pointes should be avoided during the active treatment phase. Refer to Appendix D for a list of such drugs.
- The use of any natural or herbal products or other 'folk remedies' should be discouraged.

V1.5.11.3 Permitted and/or Recommended Treatments

The following treatments are permitted throughout the duration of the active treatment phase, with all medications and treatments to be recorded in the eCRF:

- Continuation of therapies for pre-existing medical conditions.
    - This includes bisphosphonates and/or receptor activator of nuclear factor kappa-B ligand (RANKL) inhibitors for the treatment of osteoporosis or management of existing bone metastases, provided patients have been receiving them at a stable dose for at least 2 weeks prior to randomization.
        - Please note that the need to initiate, or increase the dose of, these therapies during the study will be considered as indicative of disease progression unless disease progression can be fully ruled out and the exact alternative reason for the use of these therapies is clearly documented in the subject's source documentation.
- Treatments of medical and/or surgical complications.
- All study patients should be offered **best supportive care** as per standard institutional practice and/or most recent guidelines by organizations such as the American Society of Clinical Oncology (ASCO), the National Comprehensive Cancer Network (NCCN) in the US and/or the European Society of Medical Oncology (ESMO).
    - **Anti-emetic medication** should be given for nausea (N) and/or vomiting (V) as deemed indicated by the investigator.
        - Low grade N/V were the most common adverse effects observed to date in Module 1A.
        - Having a light meal finishing ≥2 hours before administration of CT7001 appeared to reduce the risk and/or severity of N/V.
        - The first dose of CT7001 should be given without prophylactic anti-emetic medication as approximately a third of patients in Module 1A did not require anti-emetic medication. Secondary anti-emetic prophylaxis is allowed if deemed clinically indicated by the investigator.
        - In case of treatment-emergent N/V, the first choice of anti-emetic medication should be oral metoclopramide dosed as per institutional practice.
        - In case control of N/V by metoclopramide is insufficient, a serotonin (5-HT3) antagonist given orally should be considered as next choice. This includes ondansetron, granisetron or dolasetron, each to be dosed as per institutional practice and/or ASCO/NCCN/ESMO guidelines.
        - Some patients in Module 1A received an oral proton-pump inhibitor in addition to an anti-emetic and this appeared to reduce N/V in a subset of patients.

- Aprepitant (Emend®) is a substrate, moderate inhibitor and inducer of CYP3A4 and an inducer of CYP2C19. Therefore, aprepitant should not be used in this study for the treatment of nausea and vomiting.

- **Haematopoietic growth factors**:
  - Primary prophylactic use of granulocyte colony stimulating factor (G-CSF) or granulocyte macrophage colony stimulating factor (GM-CSF) is not permitted but non-pegylated G-CSF may be used to treat treatment-emergent neutropenia when clinically indicated as per standard institutional practice and/or most recent ASCO/NCCN/ESMO guidelines.
    - If neutropenic complications occur in a cycle, secondary prophylaxis may be given at the discretion of the investigator, but only if dose reduction or delay are not considered to be a reasonable alternative.
  - Erythropoietin may be used at the investigator's discretion for the supportive treatment of anaemia.
- **Red blood cell transfusions** may be given as clinically indicated for the treatment of anaemia but should be clearly noted as concurrent medications.
- **Diarrhoea**: In the event of diarrhoea, supportive measures should be initiated promptly. These include the following:
  - At the first sign of loose stools, the patient should initiate anti-diarrheal therapy (e.g., loperamide) and notify the investigator/site for further instructions and appropriate follow-up.
  - Patients should also be encouraged to drink plenty of fluids (e.g., 8 to 10 glasses of clear liquids per day).
  - Site personnel should assess response within 24 hours.
  - If diarrhoea does not resolve with anti-diarrheal therapy within 24 hours to at least Grade 1, CT7001 should be suspended until diarrhoea is resolved to at least Grade 1.
  - In case of Grade ≥3 diarrhoea, CT7001 should be interrupted (with a cyclve) or delayed (at start of next cycle). See also Sections V1.5.7.1.1 and V1.5.7.1.2.
  - In severe cases of diarrhoea, the measuring of neutrophil counts and body temperature and treatment with antidiarrheal agents should be considered.
    - Antidiarrheal agents should be deferred if blood or mucus is present in the stool or if diarrhoea is accompanied by fever. In these circumstances, appropriate diagnostic microbiologic specimens should be obtained to exclude an infectious aetiology.

        - If diarrhoea is severe (requiring intravenous (IV) rehydration) and/or associated with fever or severe neutropenia, broad-spectrum antibiotics such as fluoroquinolones should be prescribed.

        - Patients with severe diarrhoea or any grade of diarrohea associated with severe nausea or vomiting should be carefully monitored and given intravenous fluid (IV hydration) and electrolyte replacement.

    - **Medication and other measures for pain control** should follow standard institutional practice and/or ASCO/NCCN/ESMO guidelines.
    - **Other Medications,** in accordance with local standard of care, will be permitted unless specified otherwise (see prohibited medicines and medicines not recommended).

V1.5.12 Contraception

Women of childbearing potential (for definition of no childbearing potential see Section V1.3.1, Inclusion Criterion #6) must practice effective contraception. This includes:

- Abstinence if consistently employed

- Sex only with person of the same sex or with vasectomised partner

- Medroxyprogesterone injections (e.g., Depo-Provera), levonorgestrel intrauterine system (e.g., Mirena), intrauterine device (IUD), or barrier method (e.g., condom, diaphragm) for the duration of the study and for 6 months after the last dose of CT7001.

- **Note** that contraceptives that are prone to drug-drug interactions may not be effective because of a potential CYP3A4 interaction with CT7001. Furthermore, some hormonal contraceptives are contra-indicated in certain populations, please refer to the specific module contraception requirements.

Sexually active male subjects must be willing to use condoms with all sexual partners for the duration of the study and for 3 months after the last dose of CT7001. If a female partner is a woman of childbearing potential who is not using effective contraception (as defined above), the subject must use a condom with spermicide during the study and for 6 months after the last dose of CT7001.

V1.6 STUDY PROCEDURES AND VARIABLES (ALL MODULES)

Web Based Data Capture will be used for data collection and query handling. The Investigator will ensure that data are recorded on the eCRFs as specified in the Protocol and in accordance with the instructions provided.

The Investigator ensures the accuracy, completeness, and timeliness of the data recorded and for the provision of answers to data queries according to the Clinical Study Agreement. The Investigator will sign the completed eCRFs. A copy of the completed eCRFs will be archived at the study site.

For details of data and study management see the Study Manual.

V1.6.1 Data Collection

Following patient consent, each patient will undergo a screening during a period of up to 28 days in each module. Tumour assessments and other clinical data obtained as standard of care before informed consent may be used for the study provided the assessments fall within the protocol-specified period before the first IMP dose.

Screen Failures

The reason for screen failure will be collected by the Investigator using a screen failure log.

Demographics

Demographic data and other characteristics will be recorded for each module and will include, gender, race, date of birth, weight, height, alcohol consumption, and smoking history as a minimum.

Medical History

A standard medical, medication, and surgical history will be obtained for each patient with review of the selection criteria with them.

V1.6.2 Safety

Adverse Events

See Core Protocol Section V1.7.

Physical Examination

A complete physical examination will be performed in each module at the time points specified in the respective Schedules of Events and as clinically indicated and results entered in the eCRF.

ECOG Performance Status

Performance status will be assessed in each module as follows:

0 = Fully active, able to carry out all predisease activities without restriction.

1 = Restricted in physically strenuous activity but ambulatory and able to carry out work of a light or sedentary nature e.g., light housework, office work.

2 = Ambulatory and capable of self-care but unable to carry out any work activities; up and about more than 50% of waking hours.

3 = Capable of only limited self-care, confined to bed or chair more than 50% of waking hours.

4 = Completely disabled, cannot carry on self-care, totally confined to bed or chair.

The status score will be entered in the eCRF.

Vital Signs

Systolic and diastolic blood pressure and pulse will be measured in each module after the patient has been resting semisupine for at least 10 minutes. Temperature, respiratory rate, and oxygen saturation will also be measured. Vital sign measurements will be entered in the eCRF.

Height and Weight

Patient height and weight will be measured and entered in the eCRF.

12-Lead Electrocardiogram (ECG)

Triplicate 12-lead ECG will be performed 3 to 5 minutes apart after the patient has been resting semisupine for at least 10 minutes. Heart rate, RR interval, PR interval, QRS complex, QT interval, and QTcF will be entered in the eCRF. The timing of ECG assessments may be altered depending on the emerging PK and safety profile. Additional ECG assessments may be added if indicated by the emerging data.

Laboratory Safety Assessment

Samples for the following tests will be collected and tested and results entered in the eCRF:

- Haematology: haemoglobin, platelet count, reticulocyte count (absolute particle count or relative particle count), haematocrit, mean cell volume and red blood cell count, white blood cell count with differential (absolute or percent counts of neutrophils, lymphocytes, monocytes, basophils, eosinophils).
- Serum chemistry: albumin, alkaline phosphatase, ALT, AST, bilirubin (total), calcium (total), creatinine, glucose, magnesium, phosphate, potassium, sodium, urea nitrogen or urea, C-reactive protein, chloride, creatine Kinase (IU/L), Gamma Glutamyl Transferase (IU/L), Total Protein and tumour specific biomarkers.
- Coagulation: INR, activated partial thromboplastin time.
- Pregnancy test (Urine or Serum).
- Urinalysis: blood, glucose, protein.

The timing of blood samples within each module may be altered depending on the emerging PK and safety profiles. Additional sampling times may be added if indicated by the emerging data.

Laboratory values that meet the criteria for ≥ CTCAE Grade 3 or have changed significantly from Baseline and are considered to be of clinical concern will be repeated within 7 days and followed-up as appropriate. If a decrease in lymphocytes of ≥ CTCAE Grade 2 occurs, an assessment of lymphocyte populations will be performed.

In the event of an AST or ALT value ≥ 3 × ULN or total bilirubin ≥ 2 × ULN, refer to APPENDIX C for further instructions.

V1.6.3 Core Study Pharmacokinetics

Collection of Pharmacokinetic Samples

Instructions for the collection and processing of PK samples are provided in a separate Laboratory Manual. Collection time of previous dose, time of dose on the sample day and sample time will be entered in the eCRF.

If a patient misses any CT7001 dose in any module within 3 days before PK sampling is due contact the Carrick Physician or Emas Medical Monitor to determine change to the timing of the PK samples.

Determination of Drug Concentration in PK Samples

The concentration of CT7001 and any metabolites, if applicable, will be quantified by liquid chromatography with tandem mass spectrometry.

V1.6.4 Core Biomarker Analysis

Peripheral Blood for PDc Biomarker Analysis

Instructions for the collection processing of blood for biomarker analysis are provided in a separate Laboratory Manual for each study module. Collection times will be entered in the eCRF. The peripheral blood mononuclear cell (PBMC) sample will be collected at the same time as the respective overlapping PK sample timepoints. Specific biomarkers for investigation in each module are discussed in the respective module sections or Appendices of this Protocol.

Serial Tumour Samples

For patients with accessible lesions and who have provided consent, collection of fresh serial tumour biopsies is either mandatory or optional depending on the Module. Collection times will be entered into the eCRF. If possible biopsies should be taken the same day as a PK sample visit, if applicable to the Module.

The timing of these samples may be changed on the basis of emerging PK or PDc data available during the trial. An accessible lesion is defined as a tumour lesion that is amenable to repeat biopsy, unless clinically contraindicated or the patient has withdrawn consent. Failure to obtain sufficient tumour sample after making best efforts to biopsy the tumour will not be considered a protocol deviation.

Specific biomarkers for investigation in each module are discussed in the respective module sections of this report. Collection and processing instructions are provided in a separate Laboratory Manual. Collection times will be entered in the eCRF. A time-window for obtaining the on-treatment tumour biopsy (e.g. between 2 and 5 hours after a morning dose) will be specified in the lab manual. The timing of biopsies may be adjusted during the study, depending on emerging data, in order to ensure appropriate evaluation of the PDc effect of CT7001.

Archival Tumour Samples

If available, a formalin-fixed tumour tissue sample embedded in a paraffin block may be requested for each patient within a study module. Even if baseline biopsy samples can also be collected, retrieval of the archival diagnostic tumour material is still highly encouraged to

provide data on how the tumour has evolved since diagnosis. Archival samples from either primary or metastatic tumours will be accepted, but tissue from the primary tumour is preferred.

Tissue from the most recent biopsy is preferred for a patient who has archival tissue samples from multiple time points.

Tumour tissue blocks are preferred, but freshly prepared unstained slides (minimum 10, preferably 20) with 4 micron sections from the archival tumour block are accepted if tumour blocks cannot be submitted.

Collection and processing instructions are provided in a separate Laboratory Manual for each study module. Analyses will include genotyping of the tumour for all patients where archival tissue material is available. Collection times will be entered in the eCRF. **Tumour biopsies may be postponed** if the signs or symptoms of persistent or prolonged bleeding events are observed. Such events include; bleeding events requiring significant clinical intervention, INR or a PTT (partial thromboplastin time) > ULN (, platelets <50 x 10^9/L, urine dipstick "+++" for blood, or any other significant clinical concern as determined by the Investigator. Anti-platelet therapy should be stopped 7 days prior to the date of any planned fresh tumour biopsy and restarted afterwards.

V1.6.5 Exploratory Research

Exploratory Blood-borne Biomarkers

For patients in each module a blood sample will be collected and collection times entered in the eCRF. Instructions for the collection and processing of blood samples for biomarker analysis using plasma and serum are provided in a separate Laboratory Manual.

Pharmacogenetics and Circulating Tumour DNA

For patients in each module who have consented separately to genetic research, a blood sample will be collected and collection times entered in the eCRF. Collection and processing instructions are provided in a separate Laboratory Manual. In addition, plasma samples derived from the PBMC samples (above) will be processed for isolation of circulating tumour DNA (ctDNA).

For genetic research, the processes adopted for the coding and storage of samples will be more stringent to maintain patient confidentiality. As an added precaution, irrespective of the type of sample, the DNA sample will be assigned a unique number replacing the information on the sample tube. Thereafter, the DNA sample will be identifiable by the unique DNA number only.

The DNA number will be used to identify the sample and corresponding data at the contract laboratory. The link between the patient enrolment code and the DNA number will be maintained and stored in a secure environment with restricted access. The link will be used to identify the relevant DNA samples for analysis, facilitate correlation of genotypic results with clinical data, allow regulatory audit, and trace samples for destruction in the case of a patient who has requested disposal or destruction of samples not yet analysed.

No personal details identifying the individual will be available to any person (Carrick employee or contract laboratory staff) working with the DNA.

V1.6.6 Biological Sampling Procedures

Study sampling procedures are specific to each module, and are therefore described in the respective module-specific sections/Appendices of this Protocol.

Handling, Storage, and Destruction of Biological Samples

Instructions for the handling, storage, and destruction of biological samples obtained in this study are provided in a separate Laboratory Manual.

Labelling and Shipment of Biohazard Samples

Instructions for the labelling and shipment of biohazard samples in this study are provided in a separate Laboratory Manual.

Chain of Custody for Biological Samples

A full chain of custody is maintained for all samples used in this study throughout the sample lifecycle.

For each module, the Principal Investigator at each study centre keeps full traceability of collected biological samples from the patients while in storage at the centre until shipment or disposal (where appropriate) and keeps documentation of receipt of arrival.

The sample receiver keeps full traceability of the samples while in storage and during use until used or disposed of or until further shipment and keeps documentation of receipt of arrival.

Emas on behalf of Carrick Therapeutics keeps oversight of the entire life cycle through internal procedures, monitoring of study sites and auditing of external laboratory providers.

Samples retained for further use will be stored in a registered a biobank system during the entire life cycle.

Withdrawal of Informed Consent for Donated Biological Samples

If a patient withdraws consent to the use of voluntarily donated biological samples, then the samples will be disposed of/destroyed, and the action documented. If samples are already analysed, Carrick is not obliged to destroy the results of this research.

As collection of these biological samples is a voluntary part of the study then the patient may continue in the study.

The Principal Investigator:

- Ensures Carrick is notified immediately of the patient withdrawal of informed consent to the use of donated biological samples.
- Ensures that biological samples from that patient, if stored at the study site, are immediately identified, disposed of/destroyed, and the action documented.

- Ensures the laboratory(ies) holding the samples is/are informed about the withdrawn consent immediately and that samples are disposed of/destroyed, the action documented and the signed document returned to the study site.

- Ensures that the patient and Carrick are informed about the sample disposal.

Carrick /Emas ensures the central laboratory(ies) holding the samples is/are informed about the withdrawn consent immediately and that samples are disposed of/destroyed, the action documented and the document returned to the study site.

V1.6.7 Anti-tumour Activity

CT or magnetic resonance imaging (MRI) scans will be performed on each patient in a study module. Baseline images should encompass all areas of known predilection for metastases in the disease under evaluation and should additionally investigate areas that may be involved based on signs and symptoms of individual patients. Baseline assessments will be performed no more than 28 days before the first dose of CT7001 in a module and should be performed as close as possible to the start of study treatment within that module.

The imaging method used for the patient at Screening will be used at each subsequent visit.

Any other sites at which new disease is suspected should also be appropriately imaged. If an unscheduled assessment is performed and the patient has not progressed, every attempt should be made to perform subsequent assessments at the scheduled visits while the patient remains on study treatment.

Tumours will be measured and measurements entered into the eCRF.

Tumour Assessment

- Tumour response be will be assessed at each visit on the basis of current disease status compared with that at Baseline and previous visits and entered into the eCRF. At each tumour assessment visit in each module patients will be assigned a tumour response as below:

- CR = Complete response.

- PR = Partial response.

- SD = Stable disease.

- PD = Progression of disease.

If a tumour assessment cannot be evaluated, then a response of not evaluable (NE) will be assigned unless there is evidence of progression in which case the response will be assigned as PD.

Anti-tumour activity will be assessed locally by the Investigator. The Sponsor reserves the right to request an anonymised independent review of subject scan data if required.

V1.7 ADVERSE EVENT REPORTING

All observed adverse events (AEs) regardless of suspected causal relationship to the investigational product will be reported as described in the following sections. For all AEs, the investigator must pursue and obtain information adequate both to determine the outcome of the AE and to assess whether it meets the criteria for classification as a SAE requiring immediate notification to the sponsor or its designated representative ('the sponsor').

For all AEs, sufficient information should be obtained by the investigator to determine the causality of the AE. The investigator is required to assess causality.

Follow-up by the investigator may be required until the event or its sequelae resolve or stabilize at a level acceptable to the investigator, and the sponsor concurs with that assessment.

As part of ongoing safety reviews conducted by the sponsor, any non-serious AE that is determined by the sponsor to be serious will be reported by the sponsor as an SAE. To assist in the determination of case seriousness further information may be requested from the investigator to provide clarity and understanding of the event in the context of the clinical study.

All AEs spontaneously reported by the patient, reported in response to an open question from the study personnel (e.g., 'Have you had any health problems since the previous visit/you were last asked?'), or revealed by observation will be recorded in the eCRF.

When recording AEs, the diagnosis is preferred (when possible) to a list of signs and symptoms. However, if a diagnosis is known and there are other signs or symptoms that are not generally part of the diagnosis, the diagnosis and each sign or symptom should be recorded separately.

If no diagnosis, disease or syndrome can be recognized, signs or symptoms should be described in the study patient's own words (verbatim) unless, in the opinion of the investigator, clarification of the patient's verbatim language is deemed necessary.

The AE term will subsequently be coded using MedDRA.

The AE term, date of AE onset, date of AE resolution (if applicable), seriousness, severity, causality, action taken for the AE, and outcome will be recorded in the eCRF.

Medical conditions that exist before signing the informed consent form will be recorded as part of medical history.

V1.7.1 Reporting Period

AEs (serious and non-serious) should be recorded on the eCRF from the time the patient has taken at least one dose of investigational product through the patient's last visit. If a patient begins a new anti-cancer therapy, the AE reporting period for non-serious AEs ends at the time the new treatment is started.

For SAEs, the active reporting period to the sponsor begins from the time the patient provides informed consent, which is obtained prior to the patient's participation in the study, i.e., prior to undergoing any study-related procedure and/or receiving the investigational product, through the end of study visit (28-35 calendar days after the last administration of the investigational product). SAEs occurring to a patient after the active reporting period has ended

should be reported to the sponsor if the investigator becomes aware of them; at a minimum, all SAEs that the investigator believes have at least a reasonable possibility of being related to the investigational product are to be reported to the sponsor.

Death must be reported as an SAE if it occurs during the SAE reporting period after the last dose of investigational product, irrespective of any intervening treatment.

V1.7.2 Definition of Adverse Event

For purpose of this study, an AE is any untoward medical occurrence in a patient who participates in this study. The event need not necessarily have a causal relationship with the investigational product or procedure. Examples of AEs include but are not limited to:

- Clinically significant symptoms and signs (including abnormal laboratory findings)
- Changes in physical examination findings
- Hypersensitivity
- Drug abuse
- Drug dependency

Additionally, they may include the signs or symptoms resulting from

- Drug overdose
- Drug withdrawal
- Drug misuse
- Drug interactions
- Exposure during pregnancy
- Exposure via breast feeding
- Medication error
- Occupational exposure.

Worsening of signs and symptoms of the malignancy under study should be reported as AEs in the appropriate section of the eCRF. Disease progression assessed by measurement of malignant lesions on radiographs or other methods should not be reported as AEs.

V1.7.3 Definition of Serious Adverse Events

A SAE is any untoward medical occurrence at any dose that:

- Results in death
- Is life-threatening (immediate risk of death)
- Requires inpatient hospitalization or prolongation of existing hospitalization
- Results in persistent or significant disability/incapacity (substantial disruption of the ability to conduct normal life functions)

- Results in congenital anomaly/birth defect
- Is an important medical event that may jeopardise the patient or may require medical intervention to prevent one of the outcomes listed above

Medical and scientific judgment should be exercised in determining whether an event is an important medical event. An important medical event may not be immediately life-threatening and/or result in death or hospitalization. However, if it is determined that the event may jeopardize the patient or may require intervention to prevent one of the other AE outcomes, the important medical event should be reported as serious. Examples of such events are intensive treatment in an emergency room or at home for allergic bronchospasm; blood dyscrasias or convulsions that do not result in hospitalization; or development of drug dependency or drug abuse.

Progression of the malignancy under study (including signs and symptoms of progression) should not be reported as an SAE unless the outcome is fatal within the safety reporting period. If the malignancy has a fatal outcome during the study or within the safety reporting period, then the event leading to death must be recorded as an AE and as an SAE with CTCAE Grade 5.

Hospitalization due to signs and symptoms of disease progression should not be reported as an SAE.

When reporting a SAE, the following questions should be considered and included in the description if applicable:

- Is it of common occurrence in the population under study?
- Was it "treatment-emergent"?
- Did it respond to de-challenge?
- Did it recur on re-challenge?
- Were there concomitant medications?
- Were pertinent laboratory and/or other tests done?
- Was there an obvious alternative cause?

V1.7.3.1 Protocol-Specified Serious Adverse Events

There are no protocol-specified SAEs in this study. All SAEs will be reported by the investigator as described in previous sections and will be handled as SAEs in the safety database.

V1.7.3.2 Potential Cases of Drug-Induced Liver Injury (Hy's Law Cases)

Abnormal values in aspartate aminotransferase (AST) and/or alanine aminotransferase (ALT) levels concurrent with abnormal elevations in total bilirubin level that meet the criteria outlined below in the absence of other causes of liver injury are considered potential cases of drug-induced liver injury (potential Hy's Law cases) and should always be considered important medical events. Please refer to APPENDIX C for further details of actions required in cases of Hy's Law.

The threshold of laboratory abnormalities for a potential case of drug-induced liver injury depends on the patient's individual baseline values and underlying conditions. Patients who present with the following laboratory abnormalities should be evaluated further to definitively determine the aetiology of the abnormal laboratory values:

- Patients with AST or ALT and total bilirubin baseline values within the normal range who subsequently present with AST or ALT values ≥3 times the upper limit of normal (X ULN) concurrent with a total bilirubin value ≥2 X ULN with no evidence of haemolysis and an alkaline phosphatase value ≤2 X ULN or not available.
- For patients with pre-existing ALT or AST or total bilirubin values above the upper limit of normal, the following threshold values should be used in the definition mentioned above:
    - For pre-existing AST **or** ALT baseline values above the normal range: AST or ALT values ≥2times the baseline values and ≥3 X ULN or ≥8 X ULN (whichever is smaller)

      concurrent with
    - For patients with pre-existing values of total bilirubin above the normal range: Total bilirubin level increased from baseline by an amount of at least one time the upper limit of normal **or** if the value reaches ≥3 times the upper limit of normal (whichever is smaller).

The patient should return to the investigational site and be evaluated as soon as possible, preferably within 48 hours from awareness of the abnormal results. This evaluation should include laboratory tests, detailed history and physical assessment, and the possibility of hepatic neoplasia (primary or secondary) should be considered.

In addition to repeating measurements of AST and ALT, laboratory tests should include albumin, creatine kinase, total bilirubin, direct and indirect bilirubin, gamma-glutamyl transferase, prothrombin time (PT)/international normalized ratio (INR), and alkaline phosphatase. A detailed history, including relevant information, such as review of ethanol, acetaminophen, recreational drug and supplement consumption, family history, occupational exposure, sexual history, travel history, history of contact with a jaundiced person, surgery, blood transfusion, history of liver or allergic disease, and work exposure, should be collected. Testing for acute hepatitis A, B, or C infection and liver imaging (e.g., biliary tract) may be warranted. All cases confirmed on repeat testing as meeting the laboratory criteria defined above, with no other cause for liver function test (LFT) abnormalities identified at the time should be considered potential Hy's Law cases irrespective of availability of all the results of the investigations performed to determine aetiology of the abnormal LFTs. Such potential Hy's Law cases should be reported as SAEs.

V1.7.4 Hospitalization

Hospitalization is defined as any initial admission (even less than 24 hours) in a hospital or equivalent healthcare facility or any prolongation of an existing admission. Admission also includes transfer within the hospital to an acute/intensive care unit (e.g., from the psychiatric wing to a medical floor, or medical floor to a coronary care unit). An emergency room visit

does not necessarily constitute a hospitalization; however, the event leading to the emergency room visit should be assessed for medical importance.

Hospitalization does not include the following:

- Rehabilitation facilities
- Hospice facilities
- Respite care (e.g., caregiver relief)
- Skilled nursing facilities
- Nursing homes
- Same day surgeries (as outpatient/same day/ambulatory procedures).

Hospitalization or prolongation of hospitalization in the absence of a precipitating, clinical AE is not in itself an SAE. Examples include:

- Admission for treatment of a pre-existing condition not associated with the development of a new AE or with a worsening of the pre-existing condition (e.g., for work-up of persistent pre-treatment lab abnormality)
- Social admission (e.g., patient has no place to sleep)
- Administrative admission (e.g., for yearly physical examination)
- Protocol-specified admission during a study (e.g., for a procedure required by the study protocol)
- Optional admission not associated with a precipitating clinical AE (e.g., for elective cosmetic surgery)
- Hospitalization for observation without a medical AE
- Pre-planned treatments or surgical procedures.
    - These should be noted in the baseline documentation for the entire protocol and/or for the individual patient.
- Admission exclusively for the administration of blood products.

Diagnostic and therapeutic non-invasive and invasive procedures, such as surgery, should not be reported as AEs. However, the medical condition for which the procedure was performed should be reported if it meets the definition of an AE. For example, an acute appendicitis that begins during the AE reporting period should be reported as the AE, and the resulting appendectomy should be recorded as treatment of the AE.

V1.7.5 Severity Assessment

As required on the AE eCRFs, the investigator will report adverse events using concise medical terminology (verbatim) as well as collect on the eCRF the appropriate Common Terminology Criteria for Adverse Events (CTCAE) (version 5.0, publication date: November 27, 2017;

https://ctep.cancer.gov/protocolDevelopment/electronic_applications/docs/CTCAE_v5_Quick_Reference_8.5x11.pdf) and will use the following definitions of severity to describe the maximum intensity of the adverse event.

Grade	Clinical Description of Severity
1	MILD; asymptomatic or mild symptoms; clinical or diagnostic observations only; intervention not indicated.
2	MODERATE; minimal, local or non-invasive intervention indicated; limiting age-appropriate activities of daily living (ADL).
3	SEVERE or medically significant but not immediately life-threatening; hospitalization or prolongation of hospitalization indicated; disabling; limiting self-care ADL.
4	LIFE-THREATENING consequences; urgent intervention indicated
5	DEATH

Note the distinction between the severity and the seriousness of an AE. A severe event is not necessarily an SAE. For example, a headache may be severe (interferes significantly with the patient's usual function) but would not be classified as serious unless it met one of the criteria for SAEs listed above.

V1.7.6 Causality Assessment

The investigator’s assessment of causality must be provided for all AEs (serious and non-serious). The investigator must record the causal relationship in the eCRF, as appropriate, and report such an assessment in accordance with the serious adverse reporting requirements if applicable.

An investigator’s causality assessment is the determination of whether there exists a reasonable possibility that the investigational product caused or contributed to an AE. Generally, the facts (evidence) or arguments to suggest a causal relationship should be provided. If the investigator does not know whether or not the investigational product caused the event, then the event will be handled as “related to investigational product” for reporting purposes. If the investigator's causality assessment is "unknown but not related to investigational product", this should be clearly documented on study records.

In addition, if the investigator determines an SAE is associated with study procedures, the investigator must record this causal relationship in the source documents and eCRF, as appropriate, and report such an assessment in accordance with the SAE reporting requirements.

V1.7.7 Exposure During Pregnancy

An exposure during pregnancy occurs if:

- A female becomes, or is found to be, pregnant either while receiving or having been exposed (e.g., because of treatment or environmental exposure) to the investigational product; or the female becomes, or is found to be pregnant after discontinuing and/or being exposed to the investigational product
    - An example of environmental exposure would be a case involving direct contact with the investigational product in a pregnant woman (e.g., a nurse reports that she is pregnant and has been exposed to CT7001).

A male has either received or been exposed (e.g. because of environmental exposure) to the investigational product prior to or around the time of conception and/or is exposed during his partner's pregnancy.

If a study patient or their partner becomes or is found to be pregnant during the study patient's treatment with the investigational product, the investigator must submit this information to the sponsor, regardless of whether an SAE has occurred. This must be done irrespective of whether an AE has occurred and within 24 hours of awareness of the exposure. The information submitted should include the anticipated date of delivery. Follow-up is conducted to obtain general information on the pregnancy and its outcome for all reports with an unknown outcome. The investigator will follow the pregnancy until completion (or until pregnancy termination) and notify the sponsor of the outcome as a follow up to the initial report. In the case of a live birth, the structural integrity of the neonate can be assessed at the time of birth. In the event of a termination, the reason(s) for termination should be specified and, if clinically possible, the structural integrity of the terminated foetus should be assessed by gross visual inspection (unless pre-procedure test findings are conclusive for a congenital anomaly and the findings are reported). If the outcome of the pregnancy meets the criteria for an SAE (i.e., ectopic pregnancy, spontaneous abortion, intrauterine foetal demise, neonatal death, or congenital anomaly [in a live born baby, a terminated foetus, an intrauterine foetal demise, or a neonatal death]), the investigator should follow the procedures for reporting SAEs.

V1.7.8 Occupational Exposure

An occupational exposure occurs when during the performance of job duties, a person (whether a healthcare professional or otherwise) gets in unplanned direct contact with the investigational product, which may or may not lead to the occurrence of an adverse event. An occupational exposure is reported to the sponsor within 24hours of the investigator's awareness, using the SAE Report form, regardless of whether there is an associated AE/SAE. Since the information does not pertain to a patient enrolled in the study, the information is not reported on an eCRF. However, a copy of the completed SAE Report form is maintained in the investigator site file.

V1.7.9 Withdrawal Due to Adverse Events (See Also Section on Patient Withdrawal)

Withdrawal due to AEs should be distinguished from withdrawal due to other causes, according to the definition of AE noted earlier, and recorded on the appropriate AE eCRF page. When a patient withdraws because of an SAE, the SAE must be reported in accordance with the reporting requirements defined below.

V1.7.10 Eliciting Adverse Event Information

The Investigator is to report all directly observed AEs and all AEs spontaneously reported by the study patient. In addition, each study patient will be questioned about AEs.

V1.7.11 Reporting Requirements

Each AE is to be assessed to determine if it meets the criteria for SAEs. If an SAE occurs, expedited reporting will follow local and international regulations, as appropriate.

V1.7.11.1 Serious Adverse Event Reporting Requirements

All SAEs will be reported from informed consent until the End of Study visit. An SAE that occurs after the End of Study visit and comes to the attention of the Investigator must be reported only if there is (in the opinion of the investigator) a reasonable causal relationship with the study drug.

SAEs must be reported to the sponsor's representative (Emas) within 24 hours of becoming aware of the event. If the SAE is fatal or life-threatening, notification to Emas must be made immediately, irrespective of the extent of available AE information.

- This is achieved by completing the SAE Report form and sending it to Emas Pharma by email or fax with the Emas Medical monitor in copy:

**SAE CONTACT DETAILS:**	Emas Pharma Ltd<br>Fax: +44 (0)1462 600456<br>Email: drug.safety@bionical-emas.com
Emas Medical Monitor	Lisa White<br>Email: lisa.white@bionical-emas.com
Emas Medical Monitor (back -up)	Nayana Ghodki<br>Email: nayana.ghodki@bionical-emas.com

The Emas pharmacovigilance (PV) department, in close association with the Medical Monitor, will report every SAE to the regulatory authorities within the legally required timeframe.

After review of an SAE report by the EMAS PV department and/or the Medical Monitor, additional information may be requested (e.g., clinic or hospital records or procedure reports) to complete the report. If at the time the Investigator initially reports an SAE the event has not resolved, the Investigator must provide a follow-up report to the EMAS PV department as soon as it resolves (or upon receipt of significant information if the event is still ongoing).

The 24 hours time window for reporting also applies to additional new information (follow-up) on previously forwarded SAE reports as well as to the initial and follow-up reporting of

exposure during pregnancy, exposure via breastfeeding and occupational exposure cases. In the event the investigator does not become aware of the occurrence of an SAE immediately (e.g., if an outpatient study patient initially seeks treatment elsewhere), the investigator is to report the event within 24hours after learning of it and document the time of his/her first awareness of the AE.

For all SAEs, the investigator is obligated to pursue and provide information to Emas in accordance with the reporting timeframe specified above. In addition, an investigator may be requested by Emas and/or the sponsor to obtain specific additional follow-up information in an expedited fashion. This information collected for SAEs is more detailed than that captured on the AE eCRF. In general, this will include a description of the AE in sufficient detail to allow for a complete medical assessment of the case and independent determination of possible causality. Information on other possible causes of the event, such as concomitant medications, vaccines and/or illnesses must be provided. In the case of a patient's death, a summary of available autopsy findings must be submitted as soon as possible to the sponsor or its designated representative.

V1.7.11.2 Non-Serious Adverse Event Reporting Requirements

All AEs will be reported on the AE page(s) of the eCRF. It should be noted that the form for collection of SAE information is not the same as the AE eCRF. Where the same data are collected, the forms must be completed in a consistent manner. For example, the same AE term should be used on both forms. AEs should be reported using concise medical terminology on the eCRFs as well as on the form for collection of SAE information.

V1.7.11.3 Sponsor's Reporting Requirements to Regulatory Authorities

Adverse event reporting, including suspected unexpected serious adverse reactions (SUSARs), will be carried out in accordance with applicable local regulations.

V1.8 DATA ANALYSIS AND STATISTICAL METHODS

The core endpoints and main statistical methods are outlined in the core protocol. Any specific methods pertaining to each module will be described in each volume and a detailed statistical analysis plan (SAP) will be written and finalized. Any deviation from the CSP will be described in the SAP and reported in the CSR.

Interim and final data cut-off dates are defined for each module, where applicable, within the respective sections of this Protocol. All data summaries and analyses will be performed according to the SAP for the respective module and a CSR written describing the results.

V1.8.1 Core Study Endpoints

Core Primary Endpoints

This section describes the study endpoints common to every module of the study. Endpoints specific to a module are described within the respective module-specific section of this Protocol.

- AEs
- Clinical laboratory results (haematology, serum chemistry, coagulation, urinalysis)
- Physical examination findings
- ECOG performance status
- ECG parameters (heart rate, PR interval, QRS complex, QT interval, and QTcF)
- Weight
- Vital signs (supine systolic and diastolic blood pressure and pulse, temperature respiratory rate, oxygen saturation)

Core Secondary Endpoints

- PK Parameters for CT7001
- Biological Activity Parameters (Biomarkers)
- Anti-tumour Activity

Core Exploratory Endpoints

- PK Parameters for Major Metabolites:
    - PK Metabolite:CT7001 ratio
    - Metabolite identification
- Predictive Markers and Acquired Resistance to CT7001
    - Total and Phosphorylated CDK7
    - Myc oncogene expression

V1.8.2 Determination of Sample Size

The sample size of cohorts for each study module will be based on the requirement for adequate safety, PK, and PDc data balanced with exposure of as few subjects as possible to the IP and study procedures. The cohorts may be expanded by additional evaluable subjects as specified within the respective modules, and at doses at or above the established minimally biological active dose.

V1.8.2.3 Calculation or Derivation of Safety Variables

Details will be provided in the SAP.

V1.8.2.4 Calculation or Derivation of Pharmacokinetic Variables

Details will be provided in the SAP.

V1.8.2.5 Calculation or Derivation of Biomarker Variables

Details will be provided in the SAP.

V1.8.2.6 Calculation or Derivation of Exploratory Research Variables

Details will be provided in the SAP.

V1.8.2.7 Calculation or Derivation of Anti-Tumour Acitivity Variables

Details will be provided in the SAP.

V1.8.3 Description of Analysis Data Sets

The following populations will be used for analysis in all study modules:

- Safety Population: All subjects who received at least 1 dose of CT7001.
- PK Population: All subjects who received at least 1 dose of CT7001 and who has at least 1 CT7001 plasma concentration above the lower limit of quantification and no important AEs or protocol deviations or other event that may impact PK analysis.
- Biomarker Population: All subjects who received at least 1 dose of CT7001 and provided at least 1 biomarker sample.
- Evaluable for Response Population: All subjects who received at least 1 dose of CT7001 and had measurable disease at Baseline.

In all analysis populations, subjects will be analysed according to the dose actually received.

V1.8.4 Methods of Statistical Analysis

Statistical methods common to all modules are presented here with module-specific information presented within the relevant later sections of this Protocol. Data will be presented separately for each cohort of subjects within a study module.

With the exception of Part B of Module 2 comparing the safety, tolerability and anti-tumour activity of CT7001 in combination fulvestrant against fulvestrant alone, no formal statistical analysis will be carried out.

The data will be summarised using standard summary statistics.

Subject Disposition

The number and percentage of subjects enrolled in the study module, completing the study module, and discontinuing the study module will be presented in a tabular format. Reasons for discontinuation will also be summarised.

Important protocol deviations will be listed by subject.

A summary of analysis populations will be summarised in a tabular format.

Demographic and Baseline Characteristics

Demographics and baseline characteristics (including medical history and baseline disease characteristics) medications will be summarised descriptively for the Safety Population in each study module.

Prior and Concomitant Medications

Prior and concomitant medications will be coded using the most current WHO Drug Dictionary and summarised by anatomical therapeutic chemical level 3 and preferred term.

Exposure

Total exposure (date of last dose minus date of first dose+1) and total time on study (date of discontinuation minus date of first dose+1) will be summarised descriptively for each module.

In addition, the number and percentage of subjects with at least 1 dose interruption/dose delay and at least 1 dose reduction will be presented separately for the initial period of evaluability defined in each module and for any time in the study after this initial period.

Relative dose intensity (RDI; defined as the percentage of actual dose intensity delivered relative to the intended dose intensity through treatment discontinuation) and percentage intended dose (PID; defined as the percentage of the actual dose delivered relative to the intended dose through progression) will be derived and summarised descriptively in each module. In addition, the number and percentage of subjects will be summarised categorically for RDI and PID. The entire intended treatment period will be used in the derivation of RDI and PID in each module.

Safety Analyses

Safety analyses for all study modules will be performed using the Safety Population. Safety data from all cycles of treatment within a module will be combined in the presentation of safety data.

Graphical presentations of safety data will be presented for each module if deemed appropriate.

These presentations may include, but are not limited to, presentation of parameters against time or serum CT7001 concentration or shift plots. Appropriate scatter plots will also be considered to investigate trends in parameters compared to Baseline.

Adverse Events

AEs will be listed individually by subject, cohort, and CT7001 dose (as appropriate) within a module. For subjects who have a dose modification, all AEs (regardless of relationship to CT7001) will be assigned to the initial CT7001 dose within a module.

AE summary tables will include only TEAEs, defined as that AEs that occur from Cycle 0 Day 1/Cycle 1 Day 1 of the study module to 28 days after last dose in a module..

The number of subjects experiencing each TEAE will be summarised by MedDRA SOC, MedDRA preferred term, and CTCAE grade. The number and percentage of subjects with TEAEs in different categories (e.g., causally related, CTCAE Grade ≥3, etc.) will also be summarised.

SAEs will be analysed separately.

Details of any deaths will be listed.

Clinical Laboratory Tests

For all laboratory parameters included in the CTCAE, the CTCAE grade will be calculated.

All laboratory results will be listed individually by subject and summarised descriptively for each module. For urinalysis parameters, any qualitative assessments will be summarised using the number of subjects with results of negative, trace, or positive.

Physical Examinations

Abnormal findings will be listed.

ECOG Performance Status

ECOG performance status will be listed individually by subject and summarised descriptively for each module.

ECG Parameters

ECG parameters will be listed individually by subject and summarised descriptively for each module. QTc will be calculated using Fridericia correction formulae. This method shows the best rate correction and significantly improved prediction of 30-day and 1-year mortality. In general, Bazett's correction overcorrects at elevated heart rates and under corrects at heart rates below 60 bpm and hence is not an ideal correction. Fridericia's correction is more accurate than Bazett's correction in subjects with such altered heart rates (ICH E4).

Weight and Vital Signs

Weight and vital sign measurements will be listed individually by subject and summarised descriptively.

Pharmacokinetic Analyses

PK analyses will be performed for each module using the PK Population. The actual sampling times for each module will be used and PK parameters will be derived using standard non-compartmental methods using R Statistical Package or Phoenix WinNonlin.

Graphical presentations of PK data will be presented for each module as is deemed appropriate

For each module, plasma concentrations of CT7001 obtained will be summarised by nominal sample time. Plasma concentrations and derived PK parameters will be summarised by dose level. Parameters following single and multiple dosing will be summarised separately. Plasma

concentrations at each time point and PK parameters will be summarised using appropriate summary statistics.

For each module, all plasma concentrations and PK parameters results will be listed individually by subject for any subject with a measurable plasma concentration and summarised descriptively for the PK population.

Biomarker Analyses

Biomarker analyses will be performed in each module using the Biomarker Population.

Laboratory results in each module for which there are at least 6 subjects who have data will be summarised by visit and cohort using descriptive statistics. Summaries of change from Baseline and percent change from Baseline will also be summarised by visit and cohort.

Anti-tumour Activity Analyses

Antitumour activity endpoints will be analysed in each module using the Evaluable for Response Population.

Anti-tumour activity will be assessed locally by the Investigator. The Sponsor reserves the right to request an anonymised independent review of subject scan data if required.

Tumour Response

At each tumour assessment visit in each module subjects will be assigned a visit response of CR, PR, SDor PD depending on the status of their disease compared with baseline and previous visit assessments.

In the absence of progression, tumour response (CR, PR, SD) will be calculated in comparison to the baseline tumour measurements obtained before starting treatment.

If a subject has had a tumour assessment which cannot be evaluated, then the subject will be assigned a visit response of NE unless there is evidence of progression in which case the response will be assigned as PD.

Best Objective Response

Best objective response (BOR) will be determined for each subject in a module based on the best response recorded from the start of study treatment to the end of treatment, including any assessments for confirmation after the end of treatment in that module.

BOR will be summarised for the number and percentage of subjects in each category of response (CR, PR, SD, PD, NE).

Objective Response Rate

Objective response rate is defined as the percentage of subjects who have at least one response of CR or PR prior to any evidence of progression.

Clinical Benefit Rate (*where applicable*)

The Clinical Benefit Rate (CBR) is defined as the percentage of subjects with a confirmed reduction in tumour burden, CR or PR, and stabilisation of disease for at least 24 weeks.

Durable Response Rate

The Durable Response Rate (DRR) is defined as the percentage of subjects who have a confirmed response (CR or PR) with a duration of at least 3 months.

Duration of response will be defined as the time from the date of first documented response until date of documented progression or death in the absence of disease progression, the end of response should coincide with the date of progression or death from any cause used for the PFS endpoint. The time of the initial response will be defined as the latest of the dates contributing towards the first visit response of PR or CR.

If a subject does not progress following a response, then their duration of response will use the PFS censoring time.

Durability of Response

Durability of Response (DoR) is defined as the time from documentation of tumour response to disease progression.

Nonprogressive Disease

Non-progressive disease (NPD) will be assessed in each module at specified timepoints after the start of therapy. NPD is defined as the proportion of all subjects dosed that have a visit response of SD, PR or CR at the specified timepoint. Therefore, earlier visit responses of CR, PR that become PD or NE responses at the specified timepoint do not constitute NPD. A time window of 1 week around the specified timepoint will be applied and it is recommended that any visits occurring within this window after dosing are acceptable; however, if an earlier visit is defined as PD then the visit response at the specified timepoint would also be defined as PD.

If the response at the specified timepoint is missing or NE but the next evaluable response is SD or better, then the subject will be defined as having NPD at the specified time.

Percentage Change in Tumour Size (*where applicable)*

Percentage change in tumour size will be determined for subjects in a module with measurable disease at baseline and is derived at each visit by the percentage change from baseline in the sum of the diameters of TLs.

The best percentage change in tumour size is defined as the value representing the largest decrease (or smallest increase) from Baseline in tumour size. Percentage change in tumour size will be derived at each visit by the best percentage change from Baseline in the sum of the diameters of TLs.

Progression-free Survival

Progression-free survival (PFS) is defined as the time from start of treatment until the date of objective disease progression or death (by any cause in the absence of progression) regardless of whether the subject withdraws from therapy or receives another anti-cancer therapy prior to

progression. Patients who have not progressed or died at the time of analysis will be censored at the time of the latest date of assessment from their last evaluable RECIST assessment.

However, if the subject progresses or dies after two or more missed visits, the subject will be censored at the time of the latest evaluable RECIST assessment. If the subject has no evaluable visits or does not have baseline data, they will be censored at 0 days unless they die within two visits of baseline.

The PFS time for each module will always be derived based on scan/assessment dates not visit dates.

PFS will be descriptively summarised (n, median, quartiles, proportion progression-free at regular monthly intervals after the first CT7001 dose), and Kaplan-Meier plots will be provided as appropriate.

Overall Survival

Overall Survival (OS) is defined as the time from the date of Cycle 0 Day 1 of a module until death from any cause. Any subject not known to have died at the time of analysis will be censored on the basis of the last recorded date on which the subject was known to be alive.

OS will only be descriptively summarised (n, deaths, median, quartiles), and Kaplan-Meier plots will be provided as appropriate.

Pharmacogenomics and Exploratory Research

The results of exploratory biomarker research may be pooled with biomarker data from other studies with the study drug to generate hypotheses to be tested in future studies.

The plasma concentration data for CT7001 will be analysed using a population PK approach, which may include investigating the influence of covariates on PK.

A population PDc approach may be used to investigate the relationship between dose, PK and selected primary, secondary and/or exploratory endpoints.

Results will be reported separately from the CSR. These data may also be combined with similar data from other studies and explored using population PK and/or PK-PDc methods and will be reported separately from the Study Report. Metabolite ID may be performed on pooled samples and will be reported separately.

V1.9 ETHICS

V1.9.1 Institutional Review Board (IRB)/Independent Ethics Committee (IEC)

It is the responsibility of the investigator to have prospective approval of the study protocol, protocol amendments, patient information sheets, informed consent documents, and other relevant documents, e.g., recruitment advertisements, if applicable, from the IRB/IEC. The sponsor or its delegate will supply relevant material for the Investigator to submit to the

IRB/IEC for review and approval or may submit the required documents on behalf of the investigator, as per local standard practice, guidelines and regulations.

All correspondence with the IRB/IEC should be retained in the Investigator File. Copies of IRB/IEC approvals should be forwarded to Carrick or its delegate. The only circumstance in which an amendment may be initiated prior to IRB/IEC approval is where the change is necessary to eliminate apparent immediate hazards to the patients. In that event, the investigator must notify the IRB/IEC and Carrick or its delegate in writing immediately after the implementation.

The IRB/IEC will be provided with reports at the interval required (not to exceed 1 year) and a report after the completion or discontinuation of the Investigator's participation in the study.

V1.9.2 Ethical Conduct of the Study

The study will be conducted in accordance with legal and regulatory requirements, as well as the general principles set forth in the International Ethical Guidelines for Biomedical Research Involving Human Patients (Council for International Organizations of Medical Sciences 2002), Guidelines for Good Clinical Practice (ICH 1996), and the Declaration of Helsinki (World Medical Association 1996 & 2008). In addition, the study will be conducted in accordance with the protocol, the International Council for Harmonisation (ICH) guideline on harmonization guideline for Good Clinical Practice (GCP), and applicable local regulatory and Data Protection requirements and laws.

V1.9.3 Patient Information and Confidentiality

All parties will ensure protection of the personal data of study patients and will not include the names of study patients on any sponsor forms, reports, publications, or in any other disclosures, except where required by laws. A patient's name, address, birth date and other identifiable data will be replaced by a numerical code consisting of a numbering system provided by Carrick or its delegate to de-identify the study patient. In case of data transfer, Carrick will maintain high standards of confidentiality and protection of the study patients' personal data.

V1.9.4 Patient Consent

The informed consent document must be in compliance with ICH GCP, local regulatory requirements, data protection and legal requirements. The informed consent form(s) used during the informed consent process must be reviewed by the sponsor, approved by the IRB/IEC and available for inspection. The investigator must ensure that each study patient is fully informed about the nature and objectives of the study and possible risks associated with participation. The patients will be informed that participation is voluntary and that they can withdraw from the study at any time.

The investigator, or a person designated by the investigator, will obtain written informed consent from each patient before any study-specific activity is performed. The investigator will retain the original of each patient's signed consent document(s). The patient will be provided with a copy of the signed informed consent form(s).

A copy of the informed consent form (ICF) will be given to the patient, and the original ICF will be maintained with the patients' study records.

Separate consent will be obtained for participation in the treatment study and related mandatory procedures, and for optional procedures including but not limited to biomarker analyses, pharmacogenetic analyses and tumour biopsies.

Patients can withdraw their consent at any time, in which case the investigator must notify Carrick or its delegate in writing.

V1.9.5 Potential Risks and Benefits

V1.9.5.1 Potential Risks

The nonclinical and emerging clinical safety profile of CT7001 have not identified risks that would preclude investigation in the advanced cancer setting. The currently available clinical safety and tolerability data has to be considered as preliminary.

At 120 mg, 240 mg OD and 360mg OD CT7001 has been generally well tolerated. Adverse drug reactions of note were G1-2 nausea, vomiting and diarrhoea. At 480 mg OD, 3/6 subjects experienced a DLT (G3 diarrhoea, oral mucositis and vomiting). At 180mg BD, 1/7 patients experienced a DLT (G4 thrombocytopaenia). 360 mg OD has been determined as maximum tolerated dose and preliminary recommended Phase 2 dose.

Laboratory AEs have been rare and mild. The recorded laboratory abnormalities include increase in liver transaminases, prolongation of QTc, 1st degree AV block and anemia. Of note, a decline in white blood cells, neutrophils and platelets is not anticipated. Accordingly, fever or infection as a clinical complication of severe neutropenia or bleeding as a result of thrombocytopenia are not expected.

Reticulocyte decrease is expected based on the biomechanical effect of CDK7 inhibition on enucleation of erythroblasts and current clinical data. The current data suggest that the effect on reticulocytes is fully reversible upon discontinuation of CT7001. Of note, anemia has not been a common laboratory AE to date. However, it is to be recognized that only few patients in the Phase 1a study have remained on CT7001 for four months or longer.

All modules include mandatory procedures for safety monitoring. Various sections of the study protocol and appendices provide instructions and/or guidance to mitigate the risk of severe treatment-emergent toxicity and in case adverse effects may occur for their prompt and proper medical management.

Nonclinical data from hERG testing and in vivo safety pharmacology studies suggest a low potential of CT7001 for clinically significant prolongation of QTc, cardiovascular, respiratory or central nervous system toxicity (see IB).

Clinical drug interaction data of CT7001 are currently not available. In vitro cytochrome P450 (CYP) studies suggest that CT7001 is unlikely to cause clinically significant hepatic drug interactions through inhibition of CYP1A2, 2C9, 2B6, 2C8 and 3A5. Co-medication with drugs or food that modulate 2D6 or 2C19 and particularly CYP3A4 may affect the exposure of CT7001, and there is a potential for CT7001 to inhibit intestinal and hepatic CYP3A4 which may be clinically significant for CYP3A4 substrates. The potential of CT7001 to inhibit transporter proteins has not yet been studied. The risk of clinically significant drug interaction is mitigated by provisions in the study protocol (Sections V1.3.2, V1.5.11 and APPENDIX B) not to take certain drugs or food or doing so with caution.

CT7001 exhibits pH-dependent solubility. Medication which increases gastric pH (such as PPIs, H2 antagonists) may reduce the bioavailability of CT7001 and should be avoided unless clinically required. This is described in Section V1.5.11.2.

Preliminary in vitro studies have shown low potential of CT7001 for mutagenicity and genotoxicity. Nonetheless, patients should be informed of the potential risk of reproductive toxicity and the study protocol requires women of childbearing potential to agree to use adequate contraception during the study and for 6 months after the final dose of CT7001 (Section V1.3.1). Patients also must have a negative pregnancy test prior to enrolment. It is currently unknown whether CT7001 is excretion in human breast milk. Therefore, women who are breastfeeding are excluded from the study (Section V1.3.2).

V1.9.5.2 Potential Benefits

CT7001 is considered to have a positive benefit-risk profile for patients with advanced cancer.

CDKs are critical regulators of cell cycle progression and RNA transcription. The cell cycle CDKs 4, 6, 2, and 1 are core components of the cell cycle machinery and govern transition between cell cycle phases. The transcriptional CDKs, including CDKs 7, 9 and 12, phosphorylate the CTD of RNA polymerase II and regulate transcriptional initiation, elongation and processing. Compounds in clinical trial include the selective CDK4/6 and CDK7 inhibitors, as well as less selective agents that target CDKs 1 and 2 and the transcriptional CDKs.

CDK7 presents an opportunity to target proliferation and global transcription simultaneously as it is the CDK activating kinase that controls multiple checkpoint progressions through the cell cycle as well as stabilising the RNA polymerase II-based transcriptional apparatus. CDK7 acts bi-functionally as a CDK-activating kinase (CAK) controlling proliferation and as a transcriptional kinase phosphorylating the P-CTD-RNA PolII, thereby driving efficient transcriptional processes. CDK7 has recently emerged as an attractive gene control target in cancers driven by transcriptional dependencies and regulated by superenhancers (Kwiatkowski et al, 2014; Chipumuro et al, 2014; Christensen et al, 2014; Wang et al, 2015).

V1.9.5.3 Overall Risk/Benefit Assessment

The preclinical and emerging safety profile has not identified any risks that would preclude investigation of CT7001 in the advanced cancer setting. Based on the identified and potential risks associated with treatment, this clinical study protocol incorporates mandatory safety monitoring procedures and additional guidance documents to assist with early diagnosis and rapid management of potential drug-related symptoms.

No reproductive toxicology or teratogenic studies have been conducted with CT7001 to date, and it is unknown whether the drug is excreted in human milk. Therefore, women of childbearing potential and men should agree to use adequate contraception prior to study entry and for the duration of study participation and women who are breast feeding are excluded from the study. Both women and men should be fully informed of the lack of reproductive toxicity testing, and women must have a negative pregnancy test prior to enrolment.

V1.9.6 Patient Recruitment

Investigator databases may be used to aid patient recruitment. In case advertisements are used they must have received prior approval by IRB/IEC.

V1.9.7 Reporting of Safety Issues and Serious Breaches of the Protocol or ICH GCP

In the event of any prohibition or restriction imposed (i.e., clinical hold) by an applicable competent authority in any area of the world, or if the investigator is aware of any new information which might influence the evaluation of the benefits and risks of the investigational product, Carrick or its delegate should be informed immediately. In addition, the investigator will inform Carrick immediately of any urgent safety measures taken by the investigator to protect the study patients against any immediate hazard, and of any serious breaches of this protocol or of ICH GCP that the investigator becomes aware of.

V1.10 QUALITY CONTROL AND QUALITY ASSURANCE

During study conduct, Carrick or its agents will conduct periodic monitoring visits to ensure that the study protocol and Good Clinical Practices (GCPs) are being followed. The monitors may review source documents to confirm that the data recorded on CRFs is accurate. The investigator and institution will allow Carrick monitors or its agents as well as appropriate regulatory authorities direct access to source documents to perform this verification. The study site may be patient to review by an Institutional Review Board (IRB) or Independent Ethics Committee (IEC) and/or to quality assurance audits performed by Carrick or companies working with or on behalf of Carrick and/or to inspection by appropriate regulatory authorities. It is important that the investigator(s) and their relevant personnel are available during the monitoring visits and possible audits or inspections and that sufficient time is devoted to the process.

V1.11 DATA HANDLING AND RECORD KEEPING

V1.11.1 Case Report Forms/Electronic Data Record

As used in this protocol, the term CRF should be understood to refer to either an electronic data record, a paper form or both, depending on the data collection method used in this study.

A CRF is required and should be completed for each included patient. The completed original CRFs are the sole property of Carrick and should not be made available in any form to third parties, except for authorized representatives of Carrick or appropriate regulatory authorities, without written permission from Carrick.

The investigator has ultimate responsibility for the collection and reporting of all clinical, safety and laboratory data entered on the CRFs and any other data collection forms including source documents and ensuring that they are accurate, authentic / original, attributable, complete, consistent, legible, timely (contemporaneous), enduring and available when required.

The CRFs must be signed by the investigator or by an authorized staff member to attest that the data contained on the CRFs is true. Any corrections to entries made in the CRFs, source

documents must be dated, initialled and explained (if necessary) and should not obscure the original entry.

In most cases, the source documents are the hospital's or the physician's patient chart. In these cases, data collected on the CRFs must match the data in those charts. In some cases, the CRF, or part of the CRF, may also serve as source documents. In these cases, a document should be available at the investigator’s site as well as at Carrick and clearly identify those data that will be recorded in the CRF, and for which the CRF will stand as the source document.

V1.11.2 Record Retention

To enable evaluations and/or audits from regulatory authorities or Carrick or its designated agents the investigator agrees to keep records, including the identity of all participating patients (sufficient information to link records, e.g., CRFs and hospital records), all original signed informed consent documents, copies of all CRFs, safety reporting forms, source documents and detailed records of treatment disposition, and adequate documentation of relevant correspondence (e.g., letters, meeting minutes, telephone calls reports). The records should be retained by the investigator as long as is required by International Council for Harmonisation (ICH) guidelines, local regulations, or as specified in the Clinical Study Agreement (CSA), whichever is longer. If the investigator becomes unable for any reason to continue to retain study records for the required period (e.g., retirement, relocation), Carrick should be prospectively notified. The study records must be transferred to a designee acceptable to Carrick, such as another investigator, another institution, or to an independent third party arranged by Carrick. Investigator records must be kept for a minimum of 15 years after completion or discontinuation of the study or for longer if required by applicable local regulations. The investigator must obtain Carrick's written permission before disposing of any records, even if retention requirements have been met.

V1.12 DEFINITION OF END OF TRIAL

V1.12.1 End of Trial in all Participating Countries

End of Trial in all participating countries is defined as Last Patient Last Visit.

V1.12.2 End of Trial in a Member State of the European Union

End of Trial in a Member State of the European Union is defined as the time at which it is deemed that sufficient patients have been recruited and completed the study as stated in the regulatory application (i.e., Clinical Trial Application (CTA)) and ethics application in the Member State. Poor recruitment (recruiting less than the anticipated number in the CTA) by a Member State is not a reason for premature termination but is considered a normal conclusion to the study in that Member State.

V1.13 SPONSOR DISCONTINUATION CRITERIA

Premature termination of this study may occur because of a regulatory authority decision, change in opinion of the IRB/IEC, drug safety problems, or at the discretion of Carrick. In addition, Carrick retains the right to discontinue development of CT7001 at any time. If a study is prematurely terminated or discontinued, Carrick will promptly notify the investigator. After

notification, the investigator must contact all participating patients and the hospital pharmacy (if applicable) within a week of notification. As directed by Carrick, all study materials must be collected and all CRFs completed to the greatest extent possible.

V1.14 ADMINISTRATIVE PROCEDURES AND CONSIDERATIONS

V1.14.1 Safety Review Committee

The study will use a Safety Review Committee (SRC). The SRC membership and governance is outlined in a separate charter. The SRC will consist of Principal Investigator (or delegate) as chair, investigators from a representative subset of study sites, and medical and other scientific personnel from the sponsor and/or its delegate. Additional external patient matter expert consultants may get invited on an ad hoc basis, as appropriate. The SRC Remit document will define membership and decision process.

The SRC will be responsible for ongoing monitoring of the safety data as well as other data from patients, but also all data emerging, particularly from Module 1A and Module 4 (food effects on bioavailability of CT7001 in cancer patients). The SRC will also be responsible to determine whether inter-patient modification of the CT7001 dosing regimen appears indicated and which specific regimen to evaluate in a next cohort of patients. This assessment and decision will consider the totality of available data.

V1.14.2 Protocol Amendments

Any substantive change in the study requires a protocol amendment. All protocol amendments must be reviewed and agreed to by Carrick and the Principal Investigator(s) and approved by applicable regulatory authorities and IRB/IECs before implementation.

V1.14.3 Clinical Study Report

A final clinical study report (CSR) will be prepared in accordance with ICH guidelines on structure and contents of CSRs and any applicable regulatory and legal requirements and the completed CSR will be submitted to all relevant authorities within required time. Considering the multi-module nature of the current study, separate CSRs may be prepared and submitted for each completed study module.

V1.14.4 Financing and Insurance

Financing and insurance will be addressed in a separate clinical trial agreement.

V1.15 PUBLICATION OF STUDY RESULTS

Publication of study results is discussed in the Clinical Study Agreement.

V1.15.1 Communication of Results by Carrick

Carrick fulfils its commitment to publicly disclose clinical trial results through posting the results of this study on eudract.ema.europa.eu/ (EudraCT) and www.clinicaltrials.gov (ClinicalTrials.gov). Carrick posts the results of all studies that it has registered on EudraCT and/or ClinicalTrials.gov regardless of the reason for registration.

At EudraCT, Carrick posts the results ≤ 12 months after the end of the trial.

For posting of results at ClinicalTrials.gov, the timing depends on the status of the Carrick product:

- For studies involving a Carrick product whose drug development is discontinued before approval, Carrick posts the results within one year of discontinuation of the program (if there are no plans for out-licensing) or within two years (if out-licensing plans have not completed).
- For studies involving products that are not yet approved in any country, Carrick posts the results of completed studies within 30 days of US regulatory approval or one year after the first ex-US regulatory approval of the product (if only submitted for approval ex-US).
- For studies involving products applicable under the US Food and Drug Administration Amendments Act of 2007 (FDAAA), i.e., FDA approved products, Carrick posts results within one year of the primary outcome completion date (PCD).
    - Primary Completion Date is defined as the date that the final patient was examined or received an intervention for the purposes of final collection of data for the primary outcome, whether the clinical trial concluded according to the pre-specified protocol or was terminated.
- For studies involving products approved in any country, but not FDA approved, Carrick posts results one year from last patient, last visit (LPLV).

V1.15.2 Publications by Investigators

Carrick has no objection to publication by Investigators of any information collected or generated by Investigators, whether or not the results are favourable to the Investigational Product. However, to ensure against inadvertent disclosure of Confidential Information or unprotected Inventions, Investigators will provide Carrick an opportunity to review any proposed publication or other type of disclosure before it is submitted or otherwise disclosed.

Investigators will provide manuscripts, abstracts, or the full text of any other intended disclosure (poster presentation, invited speaker or guest lecturer presentation, etc) to Carrick at least 30 days before they are submitted for publication or otherwise disclosed. If any patent action is required to protect intellectual property rights, Investigators agree to delay the disclosure for a period not to exceed an additional 60 days.

Investigators will, on request, remove any previously undisclosed Confidential Information (other than the Study results themselves) before disclosure.

If the Study is part of a multi-centre study, Investigators agree that the first publication is to be a joint publication covering all centres. However, if a joint manuscript has not been submitted for publication within 12 months of completion or termination of the Study at all participating sites, Investigators are free to publish separately, patient to the other requirements of this Section.

For all publications relating to the study, Institutions will comply with recognized ethical standards concerning publications and authorship, including Section II “Ethical Considerations

in the Conduct and Reporting of Research" of the Uniform Requirements for Manuscripts Submitted to Biomedical Journals, http://www.icmje.org/index.html#authorship established by the International Committee of Medical Journal Editors.

Publication of study results is also provided for in the Clinical Study Agreement between Carrick and the Institution. In this section entitled Publications by Investigators, the defined terms shall have the meanings given to them in the Clinical Study Agreement.

V2 VOLUME 2- MODULE 1A [redacted] – MONOTHERAPY IN PATIENTS WITH ADVANCED SOLID MALIGNANCIES

STUDY CT7001_001 VOLUME 2, MODULE 1 PART A [redacted]

**Title of Core Study CT7001_001:**	A Modular, Multipart, Multiarm, Open-label, Phase I/IIa Study to Evaluate the Safety and Tolerability of CT7001 Alone and in Combination with Anti-cancer Treatments in Patients with Advanced Malignancies
**Title of Volume 2, Module 1 Part A:**	Phase I study of CT7001 as monotherapy
**Study Number:**	CT7001_001
**EudraCT Number:**	2017-002026-20
**ClinTrials.gov ID:**	NCT03363893
**Study Phase:**	Phase I
**Test Product:**	CT7001
**Indication in Volume 2, Module 1 Part A & Part B:**	Module 1A will evaluate the safety and tolerability of CT7001 will explore the safety, tolerability, PK, PDc effects and anti-tumour activity of CT7001 when given as monotherapy to patients with advanced solid malignancies. [redacted]
**Sponsor:**	Carrick Therapeutics<br>NovaUCD,<br>Belfield Innovation Park,<br>University College Dublin, Belfield,<br>Dublin 4, Ireland
**Medical Monitor:**	Dr Lisa White<br>Emas Pharma Ltd<br>63-65 Knowl Piece,Wilbury Way,<br>Hitchin, Hertfordshire, SG4 0TY, UK<br>Telephone: +44 (0) 1462 424400<br>E-mail: lisa.white@bionical-emas.com
**Date of Original Protocol:**	Version 2.0, 29 September 2017

Confidentiality Statement

The information in this document is confidential and will not be disclosed to others without written authorisation from the Sponsor, except to the extent necessary to obtain informed consent from persons receiving the study drug or their legal guardians, or for discussions with Regulatory Authorities, Institutional Review Boards, Ethics Committees, or persons participating in the conduct of the study. Do not copy or distribute without written permission from the Sponsor.

LIST OF ABBREVIATIONS (IN ADDITION TO CORE)

There are no abbreviations in this module in addition to those already defined previously.

V2.1 INTRODUCTION

Module 1 of this FTIH multiarm, multipart Phase I/IIa study to evaluate the safety and tolerability of CT7001 will explore the safety, tolerability, PK, PDc effects and anti-tumour activity of CT7001 when given as monotherapy to patients with advanced solid malignancies. The study design allows an investigation of the optimal dose and schedule of CT7001, with intensive safety monitoring to ensure the safety of the patients.

The module is composed of two parts, A and B. Part A will investigate the safety and tolerability of CT7001 with the aim of identifying both the MBAD and MTD dose of CT7001 There will be cohort expansions in Part A once the MBAD is defined which will collect paired biopsy data from breast cancer subjects. [redacted]

The results from this module may form the basis for decisions for further study modules and future studiesThe results from this module form the basis for decisions for further study modules and future studies.

Overall Study Module Design

Module 1 will evaluate the safety and tolerability of CT7001 as monotherapy to provide dose(s) and schedules(s) for subsequent study modules, each evaluating the safety and tolerability of a specific combination agent. Module 1 will also provide preliminary CT7001 efficacy readouts. The option to start further modules will be the decision of the SRC, based on emerging preclinical data and, safety and tolerability information from the initial module.

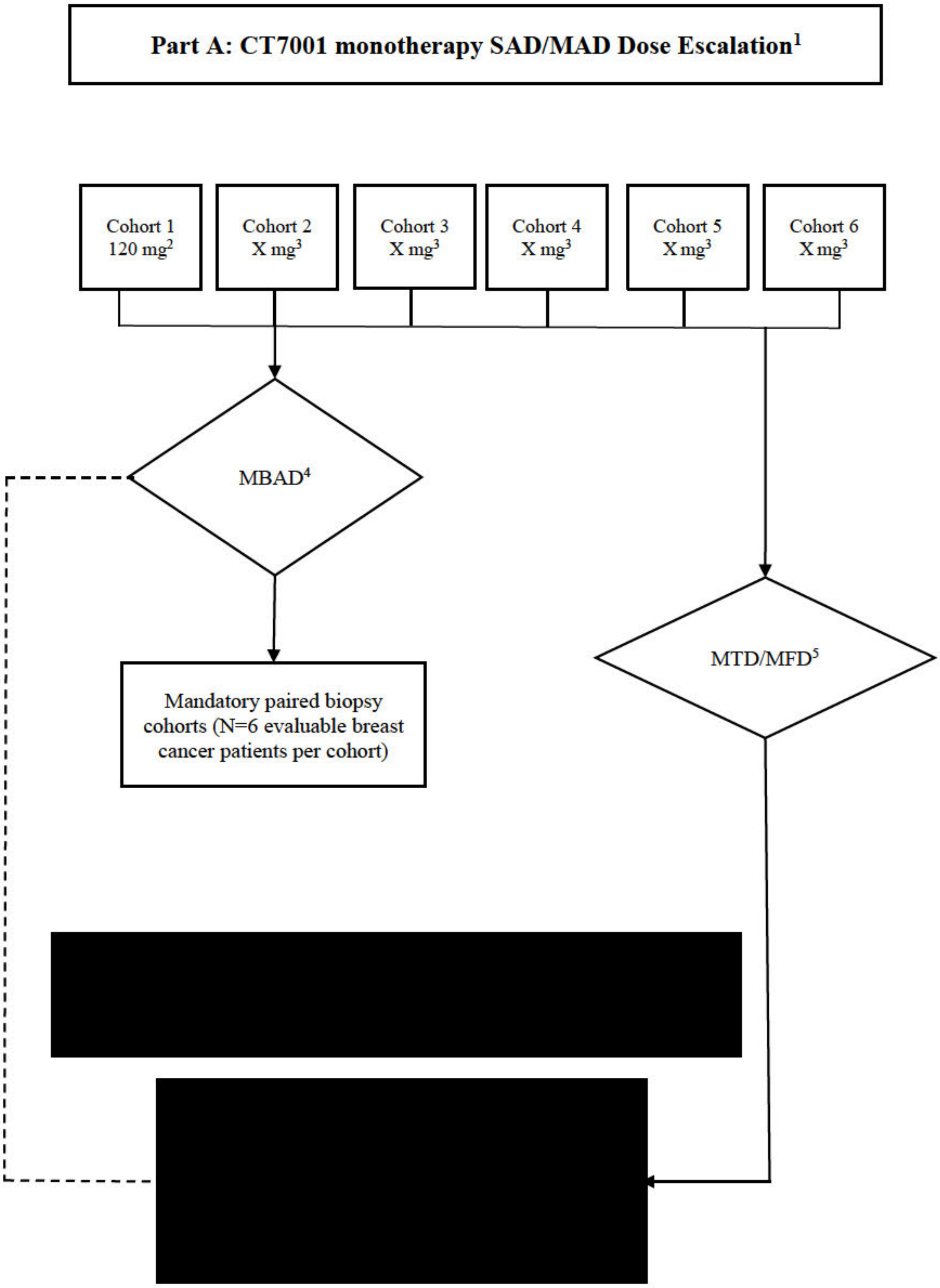

Figure 3: Overall Module 1 Design

[1] Subjects with advanced solid tumours and no established therapy available may participate in Part A: rolling 6 single ascending dose /multiple ascending dose (SAD/MAD) dose escalation design.

[2] Start dose of 120 mg CT7001 once daily; the specific determinations of the starting dose and the approach for dose escalation (including methodology for defining the most appropriate dose increment at each potential escalation step) were co-informed by the mechanism of action and all relevant preclinical data of CT7001 (the anti-cancer medicinal product under investigation), including toxicokinetics in rodents and dogs, minimum dose/exposure required for pharmacological anti-tumour effect, and type and reversibility of main organ toxicities.

[3] Upon completion of a given dosing cohort, the dose increment for the next escalation step will be determined and decided by the SRC, based data from the preceding dosing cohort (e.g., type, grade and duration of toxicity; PK; PDc; clinical anti-tumour effect), as well as all available data from at least three evaluable subjects dosed at that point. In addition, the study will employ the so-called continuous reassessment method (CRM) to aid the SRC in making the best-informed decision with regard to dose increments (see also protocol Section V2.4.2). Depending on those data, dose increments might be as high as 100%, but also no more than 30%.

[4] In this example, MBAD would be achieved in Cohort 2; this then would be the earliest possible trigger for mandated sequential biopsy cohorts, and also the expansion cohorts (part B). The decision to trigger these cohorts will be made by the SRC, dependent upon all available data, including safety, tolerability, PK, PDc and anti-tumour effect.

[5] Dose escalations will occur until either MTD or MFD is achieved; in this example, MTD would be achieved in Cohort 5. MFD would occur when subjects could no longer take the amount of CT7001 capsules in a given cohort.

The SRC will decide when to commence the Part A cohort expansions in breast cancer subjects and whether to commence Part B of Module 1 if deemed to be necessary once the MBAD has been identified from dose escalation in Part A. The MTD will be notified immediately to all sites along with guidance on subject management, where relevant. [redacted]

V2.1.1 Module 1 Part A

Part A of Module 1 will commence by enrolling patients with advanced solid tumours into a monotherapy dose escalation arm. Eligible participants will be enrolled in sequential cohorts treated with CT7001 given as an oral capsule dose while being monitored for safety and DLTs.

Part A will also include a sequential tumour biopsy expansion cohort in breast cancer patients, for evaluation of PK/PDc and tumour responses once MBAD is defined and agreed by the SRC.

The dose escalation, cohort size and the stopping criteria of this part of Module 1 are based upon accepted methodologies for Phase I oncology studies.

The starting dose of CT7001 has been chosen based upon data from the GLP toxicology studies and using the FDA guidance for industry (e.g. ICH S9) for starting dose selection for a cytotoxic agent in cancer patients.

Dose increments will be guided by safety data observed during Cycle 1, as well as on-going assessment of safety beyond Cycle 1 in earlier cohorts, plus PK and PDc data as available. Every new dose cohort will be evaluated for the occurrence of a DLT during Cycle 0 or Cycle 1 of treatment. Initially, patients will receive a single dose of CT7001 on Day 1 of Cycle 0 (C0D1) followed by a 2-day washout period (C0D2), before receiving cycles of 21 continuous days dosing of CT7001 capsules.

The frequency of dosing, any washout period to assess PK and dosing staggering interval between patients may change based upon emerging data as reviewed and agreed by the SRC without the requirement to submit a substantial amendment to the protocol. The frequency of

PK sampling may also be modified based on SRC review, and may include up to three additional PK samples of 4 ml each within any given cycle and taken by venepuncture, to better characterise the individual PK profile based upon emerging PK data.

V2.1.2 Module 1 Part A [redacted] Cohort Expansions

Once an MBAD dose of CT7001 has been identified from Part A of the module, the SRC may decide to commence sequential tumour biopsy cohort expansions in breast cancer patients (n=6 evaluable patients). Evaluable patients will have two evaluable samples from 2 different time points, for example, a baseline sample at study entry and a further sample while on treatment.

[redacted]

V2.1.3 Rationale

Rationale for Starting Dose

The starting dose of CT7001 has been selected using the ICH S9 guidance for starting dose selection for a new drug in cancer patients, the goal of which is to identify a dose that is expected to have pharmacologic effects and is reasonably safe to use. The choice of this dose has been justified using all available non-clinical data.

In the human tumour xenograft mouse models of anti-tumour activity, 30 and 50 mg/kg/day showed moderate efficacy, whilst 100 mg/kg/day showed robust efficacy. The table below shows the predicted efficacious human doses based on scaling factors from mouse to human (Table 3). These data suggest that 120 mg would be expected to be the lowest human dose expected to have pharmacologic effects.

Table 3: Predicted Efficacious Human Doses based on Scaling Factors from Mouse to Human

**Human doses versus those showing moderate and robust anti-tumour activity in human tumour xenograft mouse models**			
**Human dose (mg/60 kg)**	**Human dose (mg/kg)**	**Relative to Mouse Moderate PD and Efficacy (i.e. 30 mg/kg Dose, HED = 2.4 mg/kg)**	**Relative to Mouse Robust PD and Efficacy (i.e. 100 mg/kg Dose, HED = 8 mg/kg)**
60	1	0.42	0.12
120	2	0.83	0.25
240	4	1.67	0.50
480	8	3.33	1.0
960	16	6.67	2.0

HED = human equivalent dose

In the 28-day GLP compliant Toxicology studies, top-doses were 120 mg/kg/day and 20 mg/kg/day in rats and dogs, respectively. These doses produced similar total exposure in male dogs, female dogs and female rats, whilst C_{max} in male rats was lower (Table 4). Despite comparable exposure, the toxicity in rats given 120 mg/kg was greater in severity than in dogs given 20 mg/kg/day. Therefore, the rat is more sensitive and has been used to calculate the initial dose to be used in the first clinical trial with CT7001.

Table 4: C_{max} and AUC_{0-t} Values from 28-day Toxicology Studies

**28d Tox Study (species)**	**CT7001 Oral Dose (mg/kg/day)**	**Cmax (ng.h/mL)**		**AUC0-t (ng h/mL)**	
		**Male**	**Female**	**Male**	**Female**
**Rat**	60	234	415	2250	3190
**Rat**	120	445	696	4540	5320
**Dog**	20	777	1220	3880	5010

As described below, daily oral gavage administration of 15, 60, or 120 mg/kg/day CT7001 to Han Wistar (Crl:WI [Han]) rats for up to 4 weeks resulted in deaths and severe clinical observations in animals administered 120 mg/kg/day. Doses of 60 and 120 mg/kg/day produced histological changes in a range of tissues, however, the effects produced by 60 mg/kg were much less severe than those produced by 120 mg/kg/day.

As discussed in Section V1.1.4, the pre-clinical PK of CT7001 is associated with a large volume at steady state (V_{ss}) and high clearance but good bioavailability and so robustly predicting human exposure after oral dosing is challenging. Emphasis has therefore been given to scaling of dose, as per Note 2 in the ICH S9 guideline, rather than modelling plasma kinetics (Table 5).

Table 5: Margins from the Proposed Human Doses to Doses used in the GLP Rat and Dog Studies based on Scaling Factors

Human doses in comparison to those in rats and dogs achieved with the top doses used in Toxicology studies				
**Human dose (mg per 60 kg)**	**Human dose (mg/kg)**	**Relative to Rat middle dose tested and HNSTD (i.e. 60 mg/kg Dose, HED = 10 mg/kg)**	**Relative to Rat highest dose tested (i.e. 120 mg/kg Dose, HED = 19 mg/kg)**	**Relative to Dog highest dose tested and HNSTD (i.e. 20 mg/kg Dose, HED = 11 mg/kg)**
60	1	0.10	0.05	0.09
120	2	0.20	0.11	0.18
240	4	0.40	0.21	0.36
480	8	0.80	0.42	0.73
960	16	1.60	0.84	1.45

When using rodent toxicology studies to guide selection of start doses for small molecule anti-cancer drugs, guideline ICH S9 suggests setting a start dose of 1/10 of the severely toxic dose in 10% of the animals (STD 10). Despite 120 mg/kg/day being greater than the STD 10, as more than 10% of the rats showed severe toxicity, Carrick has used this dose to derive the first dose to be used in the Phase I clinical trial of CT7001 in cancer patients. This is justified for the following reasons:

- 60 mg/kg/day in rats is below the STD 10 because none of the animals showed severe toxicity.
- Although doses of both 60 mg/kg/day and 120 mg/kg/day in rats produced histological changes in a range of tissues, the effects produced by 60 mg/kg were much less severe than those produced by 120 mg/kg/day.
- Effects observed in the rat were either fully reversible or demonstrating resolution following 28 days after cessation of dosing.
- All doses explored in dogs were well tolerated with no unscheduled deaths and the highest non-severely toxic dose (HNSTD) is considered to be 20 mg/kg/day, which is a dose that results in comparable exposure to rats given 120 mg/kg.
- The preclinical toxicity of CT7001 is consistent with its expected cytostatic mode-of-action, primarily showing effects in the bone marrow and the GI tract.
- The toxicity target organs are common to most if not all, cytostatic and cytotoxic agents, and are therefore frequently encountered and adequately managed in clinical oncology; as a result, they are monitorable in the clinic and do not present a unique safety risk to patients.
- 120 mg would be expected to be the lowest human dose to have anti-tumour pharmacologic effects at 0.83 the dose producing moderate anti-tumour activity in mouse models.

A dose of 120 mg/kg/day equates to a human equivalent dose (HED) of 2 mg/kg, which for a 60 kg person results in a total dose 120 mg. The proposed starting dose in humans of 120 mg provides a 10-fold and 5-fold margin to those in rats and dogs given the top-doses in the

toxicology studies, respectively, demonstrating an adequate margin of dose; and a 5-fold margin to the 60 mg/kg dose in rats which was below the STD 10.

Rationale for Conducting Module 1

This first-in-human module of the multiarm, multipart, Phase I/Iia study to evaluate the safety and tolerability of CT7001, alone and in combination with anti-cancer treatments, in patients with advanced malignancies will explore the safety, tolerability, PK and PDc effects, and antitumour activity of CT7001 when given as monotherapy to patients with advanced solid malignancies. The study design allows an investigation of the optimal dose and schedule of CT7001 with intensive safety monitoring to ensure the safety of the patients.

The results from this study module may form the basis for decisions for further study modules and future studies.

Rationale for Study Module 1 Design

This study module is designed to evaluate the safety and tolerability of CT7001 at increasing doses, in patients with advanced malignancies. However, the study will also characterise the PK of CT7001 and explore potential biological activity by assessing PDc and exploratory biomarkers and anti-tumour activity. Part A, the dose escalation part of the module, will therefore be performed in an unselected population of patients with the option to cohort expand in specific patient groups to further explore specific aspects of efficacy, biomarker activity, PK parameters, or safety and tolerability of the drug combinations in Part B.

V2.1.4 Module 1 Benefit/Risk and Ethical Assessment

Potential Benefits

CT7001, in this module is considered to have a positive benefit-risk profile for patients with advanced cancer.

CDKs are critical regulators of cell cycle progression and RNA transcription. The cell cycle CDKs 4, 6, 2, and 1 are core components of the cell cycle machinery and govern transition between cell cycle phases. The transcriptional CDKs, including CDKs 7, 9 and 12, phosphorylate the CTD of RNA polymerase II and regulate transcriptional initiation, elongation and processing. Compounds in clinical trial include the selective CDK4/6 and CDK7 inhibitors, as well as less selective agents that target CDKs 1 and 2 and the transcriptional CDKs.

CDK7 presents an opportunity to target proliferation and global transcription simultaneously as it is the CDK activating kinase that controls multiple checkpoint progressions through the cell cycle as well as stabilising the RNA polymerase II-based transcriptional apparatus. CDK7 acts bi-functionally as a CAK controlling proliferation and as a transcriptional kinase phosphorylating the P-CTD-RNA PolII, thereby driving efficient transcriptional processes. CDK7 has recently emerged as an attractive gene control target in cancers driven by transcriptional dependencies and regulated by superenhancers (Kwiatkowski et al, 2014; Chipumuro et al, 2014; Christensen et al, 2014; Wang et al, 2015).

TNBC represents a heterogeneous subgroup of breast cancer with substantial genotypic and phenotypic diversity. TNBC subjects commonly exhibit poor prognosis and high relapse rates at early stages after conventional treatments. Currently, there is a lack of biomarkers and

targeted therapies for the management of TNBC. During tumour development and progression, alterations in cellular behaviour are frequently linked with kinase expression and activity. Recent studies have shown that high CDK7 expression is a promising biomarker of poor prognosis in TNBC and that targeting CDK7 may be a useful therapeutic strategy for TNBC (Li et al, 2016).

SCLC is an aggressive disease with high mortality, and has been found to be sensitive to transcription-targeting drugs, in particular to CDK7 inhibition (Christensen et al, 2014).

Despite the recent progress in development of new drugs to treat metastatic castrate-resistant prostate cancer (mCRPC) this disease continues to be incurable, illustrating the need for novel therapeutic strategies (Nuhn et al, 2019)

The development of an active treatment for platinum resistant ovarian cancer is a significant current area of unmet need. Previously unknown CDK7 target signatures have been characterized in both epithelial ovarian cancer (EOC) primary cells and ovarian cancer cell lines. CDK7 was seen to control cell proliferation and pharmacological inhibition selectively repressed EOC cell proliferation, therefore offering a potential development strategy for CT7001 (Frankavilla et al, 2017).

Refer to the IB for further information.

Potential Risks

The nonclinical and emerging clinical safety profile of CT7001 from Module 1 Part A have not identified risks that would preclude investigation in the advanced cancer setting. The currently available clinical safety and tolerability data has to be considered as preliminary.

At 120 mg, 240 mg OD and 360mg OD CT7001 has been generally well tolerated. Adverse drug reactions of note were G1-2 nausea, vomiting and diarrhoea. At 480 mg OD, 3/6 subjects experienced a DLT (G3 diarrhoea, oral mucositis and vomiting). At 180mg BD, 1/7 patients experienced a DLT (G4 thrombocytopaenia). 360 mg OD has been determined as maximum tolerated dose and preliminary recommended Phase 2 dose.

Laboratory AEs have been rare and mild. The recorded laboratory abnormalities include increase in liver transaminases, prolongation of QTc, 1st degree AV block and anemia. Of note, a decline in white blood cells, neutrophils and platelets is not anticipated. Accordingly, fever or infection as a clinical complication of severe neutropenia or bleeding as a result of thrombocytopenia are not expected.

Reticulocyte decrease is expected based on the biomechanical effect of CDK7 inhibition on enucleation of erythroblasts and current clinical data. The current data suggest that the effect on reticulocytes is fully reversible upon discontinuation of CT7001. Of note, anemia has not been a common laboratory AE to date. However, it is to be recognized that only few patients in the Phase 1a study have remained on CT7001 for four months or longer.

All modules include mandatory procedures for safety monitoring. Various sections of the study protocol and appendices provide instructions and/or guidance to mitigate the risk of severe treatment-emergent toxicity and in case adverse effects may occur for their prompt and proper medical management.

Nonclinical data from hERG testing and in vivo safety pharmacology studies suggest a low potential of CT7001 for clinically significant prolongation of QTc, cardiovascular, respiratory or central nervous system toxicity (see IB).

Clinical drug interaction data of CT7001 are currently not available. In vitro cytochrome P450 (CYP) studies suggest that CT7001 is unlikely to cause clinically significant hepatic drug interactions through inhibition of CYP1A2, 2C9, 2B6, 2C8 and 3A5. Co-medication with drugs or food that modulate 2D6 or 2C19 and particularly CYP3A4 may affect the exposure of CT7001, and there is a potential for CT7001 to inhibit intestinal and hepatic CYP3A4 which may be clinically significant for CYP3A4 substrates. The potential of CT7001 to inhibit transporter proteins has not yet been studied. The risk of clinically significant drug interaction is mitigated by provisions in the study protocol (Sections V1.3.2, V1.5.11 and APPENDIX B) not to take certain drugs or food or doing so with caution.

CT7001 exhibits pH-dependent solubility. Medication which increases gastric pH (such as PPIs, H2 antagonists) may reduce the bioavailability of CT7001 and should be avoided unless clinically required. This is described in Section V1.5.11.2).

Preliminary in vitro studies have shown low potential of CT7001 for mutagenicity and genotoxicity. Nonetheless, patients should be informed of the potential risk of reproductive toxicity and the study protocol requires women of childbearing potential to agree to use adequate contraception during the study and for 6 months after the final dose of CT7001 (Section V1.3.1). Patients also must have a negative pregnancy test prior to enrolment. It is currently unknown whether CT7001 is excretion in human breast milk. Therefore, women who are breastfeeding are excluded from the study (Section V.1.3.2).

Overall Benefit/Risk and Ethical Assessment

This study module is a first-in-human Phase I/IIa dose-escalation study with a CDK7 inhibitor. The study design aims to minimise potential risks and, although the potential benefits in patients are unknown at this time, non-clinical data demonstrate evidence of antitumour activity. Thus, the benefit/risk assessment for this module appears acceptable based on the lack of effective alternative treatments, the limited life expectancy due to malignant disease, and the strength of the scientific hypothesis under evaluation.

V2.2 MODULE 1 OBJECTIVES

Module 1 Part A Objectives

The core study objectives were presented earlier in Section V1.2.1. Additional objectives specific to Module 1 are presented below.

Additional primary objectives

- To select the CT7001 dose(s) and schedule(s) for further clinical evaluation in patients with advanced solid malignancies.

Additional secondary objectives

There are no specific Module 1A secondary objectives in addition to those stated for the Core Protocol (Section V1.2.1)

Additional exploratory objectives

There are no Module 1A exploratory objectives in addition to the Core Study Objectives presented earlier in Section V1.2.1.

[redacted]

V2.3 PATIENT SELECTION AND RESTRICTIONS ON STUDY

In addition to the eligibility criteria in the Core Protocol (Section V1.3), the criteria in the following sections will also be applied to patients enrolled in Module 1.

V2.3.1 Additional Module 1 Inclusion Criteria

Inclusion Criteria Common for Part A

Histological, radiological or cytological confirmation of an advanced non-haematological malignancy not considered to be appropriate for further standard treatment.

Inclusion Criteria for Part A Breast Cancer Cohort Expansions Only

A least one tumour suitable for biopsy.

[redacted]

V2.3.2 Additional Module 1 Parts A [redacted] Exclusion Criteria

Additional Exclusion Critera

For Module 1, there are no restrictions additional to those for the Core Protocol.

V2.4 STUDY TREATMENT, CONDUCT AND WITHDRAWAL

V2.4.1 Treatment

In this module 1A [redacted], patients will receive CT7001 as monotherapy.

V2.4.2 Module 1 Part A - Dose Escalation

Part A - Starting Dose and Dose Escalation Scheme

Administration

In Module 1, patients will receive CT7001 capsules as a single dose on Cycle 0 Day 1 with a a minimum of a 48 hour washout period (refer to Table 12, Section V2.8). CT7001 dosing will begin at 120 mg (Section V2.1.3). The dose will start as a once daily dosing regimen on Cycle 1 Day 1 (C1D1).

A cycle of study treatment (after Cycle 0) will be a minimum of 21 days.

The frequency of dosing in Part A of this module may change based on emerging PK data as reviewed and agreed by the SRC. If the dosing frequency is changed, the initial total daily dose, of the new dosing frequency, will not exceed the current maximum total daily dose that has been explored and found to be tolerable; this change can be made without the requirement of a substantial protocol amendment. Dose increments will be guided by safety data observed during Cycle 1, as well as on-going assessment of safety beyond Cycle 1 in earlier cohorts, plus PK and PDc data as available. Every new dose cohort will be evaluated for the occurrence of a DLT during Cycle 0 and Cycle 1 of treatment. Patients will receive a single dose of CT7001 on Day 1 of Cycle 0 (C0D1) followed by a washout period (initially 2 days) on Day 2 of Cycle 0 (C0D2), before receiving cycles of 21 continuous days dosing of CT7001 capsules in Cycle 1. The SRC will also review the wash-out period and may adjust it based on emerging PK data.

Initially, each patient must fast from food and liquids other than water for 2 hours before and 1 hour after each CT7001 dose. This requirement may be changed after review if fed-fasted PK data become available that show that exposure is not changed by a clinically relevant amount in the fed state compared to the fasted state.

Patients will be enrolled to ensure a minimum of 3 and a maximum of 6 evaluable patients per dose cohort, in a rolling 6 design. Dose escalation and de-escalation will proceed as follow:

- If no dose-limiting toxicity (DLT) is observed (see definition below) in a cohort of three to six evaluable patients (Section V2.7.2), then dose escalation may occur. Dose increases will be permitted after review of data from a minimum of three evaluable patients has been performed.

- If one patient experiences a DLT in a group of three or more evaluable patients, then the cohort will be expanded to include six evaluable patients. If only one DLT is observed in the complete cohort of six evaluable patients, then dose escalation may occur.

- If at least two patients experience a DLT in a group of up to six patients, irrespective of the number of patients enrolled, the dose will be considered not tolerated, and recruitment to the cohort and dose escalation will cease.

- A lower intermediary dose (de-escalation) may be considered to better define the MTD (see definition below).

The dose escalation scheme will not exceed doubling of the dose, in principle. Dose, frequency and schedule in subsequent cohorts may increase or decrease in response to safety, tolerability, pharmacokinetic and emerging non-clinical data.

As the primary purpose of Phase I clinical trials of anti-cancer agents is to estimate the MTD of the agent, in principle dose escalation will continue until either MTD or MFD has been defined. Accepted methodology for Phase I oncology studies being that the highest dose or exposure tested in the non-clinical studies does not limit the dose-escalation or highest dose investigated in patients with advanced cancer.

After each dose level during the dose escalation phase of the study, a SRC, including representation from all recruiting sites, will evaluate the safety and tolerability and PK of all patients being dosed with CT7001 to decide the next dose and/or schedule. The dose increase may be restricted to a maximum of 50% if significant (as defined by the SRC) drug related toxicity is observed in any cohort. Furthermore, if MTD is exceeded the information is rapidly communicated to all sites and no further dosing at that level, or higher, will take place.

Module 1 Part A dose cohorts may be expanded by up to 12 additional evaluable patients at doses at or above the MBAD.

Within initial dose cohorts there will be a staggering interval. The first patient will be dosed in a new cohort and subsequent patients may not be dosed until the first patient has completed 7 days of monotherapy or as advised by the SRC. Intermittent dosing schedules (e.g., 14 days of once-daily continuous dosing followed by a 7-day rest period) may also be evaluated if indicated by emerging data. Evaluation of the intermittent schedule will start at the MBAD from the daily continuous schedule. Dose escalation in the intermittent schedule will proceed using the same dose escalation design as described for the continuous dosing schedule, until the MTD is reached.

As a supportive tool, the CRM originally proposed by O'Quigley et al, (1990) will be used after the completion of each dose cohort to aid the decision by the SRC on the CT7001 dose level for the subsequent cohort and ensure that toxicities associated with longer term dosing are incorporated into the decision making process. The MTD will be defined using a target toxicity level of 25%. Prior toxicity probabilities will be elicited before the study starts to form the prior skeleton. An empiric dose toxicity model will be chosen and a normal prior distribution selected for the single model parameter (simulations will guide on the exact values of this distribution). The CRM procedure will employ the modified approach of handling partial follow-up data (TITE-CRM) as dose decisions may need to be made without the complete

follow-up data and so follow-up data are weighted by length of follow-up as given by Cheung and Chappell (2000).

The dose for subsequent cohorts or a decision to stop recruitment to Part A will be agreed by the SRC after review of the data from each cohort (Section V1.14.1).

Once the MBAD is determined and agreed by the SRC there will be tumour biopsy cohort expansions in breast cancer patients (n=6 evaluable patients with paired biopsies) within Part A of Module 1.

Definition of Dose-limiting Toxicity

A DLT is defined as any toxicity not attributable to the disease or disease-related processes under investigation, which occurs before the end of Cycle 1 and which includes:

- Haematological toxicities:
    - Grade 4 neutropenia (ANC < 500 cells/mm^3) lasting longer than 4 consecutive days
    - Grade 3 neutropenia (ANC ≥500 to <1000 cells/mm^3) of any duration accompanied by fever ≥ 38.5°C or systemic infection
    - Grade 3 thrombocytopenia (25,000 to <50,000/mm^3) with bleeding
    - Any other confirmed haematological toxicity ≥ CTCAE Grade 4 (a repeat may be required for confirmation of an isolated abnormality in the absence of clinical signs, symptoms or other abnormal investigations, i.e., a suspected spurious value).
- Non-haematological toxicity ≥ CTCAE Grade 3 including:
    - Laboratory abnormalities (a repeat may be required for confirmation of an isolated abnormality in the absence of clinical signs, symptoms or other abnormal investigations, i.e., a suspected spurious value)
    - QTcF prolongation (> 500 msec and /or + 60 ms)
    - Any other toxicity that is greater than that at Baseline and is clinically significant or unacceptable or does not respond to supportive care
    - Any event, including significant dose reductions or omissions, judged to be a DLT by the SRC.

The definition of a DLT excludes:

- Alopecia of any grade.
- Inadequately treated Grade 3 nausea, vomiting, or diarrhoea (all patients should receive optimal antiemetic or antidiarrhoeal prophylaxis or treatment).

- Any toxicity clearly unrelated to CT7001 treatment, e.g., solely related to the disease or disease-related process under investigation.
- Isolated laboratory changes of any grade without clinical sequelae or clinical significance.

Definition of an Evaluable Patient

For decisions on dose escalation, an evaluable patient is defined as a patient that has received CT7001 and has either completed minimum safety evaluation requirements and has received at least 75% of the specified dose during the first 21-day cycle or experienced a DLT during the Cycle 0 or Cycle 1.

V2.4.3 Module 1 Part A[redacted] – Tumour Biopsy Cohort Expansion

Once an MBAD dose of CT7001 has been identified from Part A of the module, the SRC may decide to commence sequential tumour biopsy cohort expansions in breast cancer patients (n=6 evaluable patients). Evaluable patients will have two evaluable samples from two different time points, for example, a baseline sample at study entry and a further sample while on treatment.

[redacted]

[redacted]

V2.5 MODULE 1 PART A [redacted]: PLAN AND TIMING OF PROCEDURES

Up to five hospitals in the United Kingdom will participate in this study module.

The number of patients to be enrolled in the module 1 is dependent on the size and number of dose escalation cohorts (three to six patients each [redacted].

Additional module duration is dependent on the number of cycles of CT7001 treatment in each Part, which will be continued until the subject is no longer gaining clinical benefit or another treatment discontinuation criterion has been met. Subjects who meet all inclusion and no exclusion criteria and provide written informed consent will be enrolled.

Each treated patient will participate in Module 1 Part A for at least 2 to 3 months, including, as a minimum, Screening (Day -28 to Day -1), 2-day Cycle 0, 21-day Cycle 1, End of Treatment (within 3 days after the last CT7001 dose) and End of Study (28 to 35 days after the last CT7001 dose). This is the same for the Part A tumour biopsy cohort expansions in breast cancer patients with the exception of Cycle 0.

Study variables will be collected throughout the entire treatment plan and at the end of the study (defined as 28 to 35 days after the last CT7001 dose). The Schedule of Events (SoE) for Module 1 Part A is provided in Table 12.

V2.5.1 Module 1 Plan

Module 1 Part A Treatment Period

In Part A of Module 1, CT7001 will be administered to patients with advanced solid malignancies at a starting dose of 120 mg (Section V2.1.3) and will be escalated to reach the MTD as defined by DLTs. Patients will initially receive one oral dose of CT7001 on Day 1 of the 6-day Cycle 0 period. Dosing will start on Cycle 1 Day 1.

The frequency of dosing, any washout period to assess PK and dosing staggering interval between patients may change based upon emerging data as reviewed and agreed by the SRCwithout the requirement to submit a substantial amendment to the protocol. The frequency of PK sampling may also be modified based on SRC review, and may include up to 3 additional PK samples of 4ml each within any given cycle and taken by venepuncture, to better characterise the individual PK profile based upon emerging PK data.

At least three and up to six evaluable patients will be required for each dose cohort. Patient enrolment will proceed according to a rolling 6 design (Section V2.4.2). In the absence of DLT, each patient will receive CT7001 for a minimum of 21 days (Cycle 1) and may continue to receive additional 21-day cycles of treatment until the patient is no longer gaining clinical benefit or another treatment discontinuation criterion has been met, at which time an End of Treatment visit will be conducted (within three days after the last CT7001 dose). An End of Study visit will be conducted 28 to 35 days after the last dose of CT7001.

At least three evaluable patients in a cohort must have completed at least one cycle of 21 days of dosing before the SRC can review and initiate the next patient cohort and dose escalation. Within initial cohorts there will be a staggering interval. The first patient will be dosed in a new cohort and subsequent patients may not be dosed until the first patient has completed 7 days of CT7001 monotherapy. On clinic days on which PK samples are scheduled to be taken, the dosing should be delayed until arrival at the clinic and patients should not take their dose until instructed to do so by site personnel.

For once a day dosing doses should be taken approximately 24 hours apart at the same timepoint each day.

Dose escalation will occur as described in Section V2.4.2.

Module 1, Part A, Tumour Biopsy Cohort Expansion in Breast Cancer Patients (Part A) [redacted]

Serial tumour biopsy cohort expansions of at least six and up to 12 additional evaluable patients may occur at doses at or above the MBAD (although enrolment to complete the dose escalation cohorts will take priority over the expansions). Serial tumour biopsies will be mandatory for the patients enrolled in these cohort expansions at the following time points:

1. At Baseline (- 7days)

2. On Day 1 of Cycle 2

3. At End of Treatment, collected as soon as possible after confirmation of PD.

At the time of each tumour biopsy, blood samples for assessment of PK parameters and biomarkers will also be collected.

[redacted]

V2.5.2 Follow-up Period

Patients will continue receiving treatment until they are no longer gaining clinical benefit or the patient withdraws consent. For Module 1A an End of Treatment visit will be performed within 3 days after the last CT7001 dose, and End of Study visit will be performed 28 to 35 days after the last CT7001 dose. [redacted]

Long-term follow up via a telephone call to the General Practitioner (GP) will be used to collect PFS data and OS data.

V2.6 COLLECTION OF STUDY VARIABLES

V2.6.1 Recording of Data

The recording of data collected in Module 1A will be conducted as discussed in Core Protocol Section V1.6 earlier.

V2.6.2 Screening

Each subject will undergo screening during a period of up to 28 days before C0 Day 1 of Part A, [redacted]. Tumour assessments and other clinical data obtained as standard of care before informed consent may be used for the study provided the assessments fall within the protocol-specified period before the first IMP dose.

All screening procedures (Part A SoE, Table 12) must be completed and results reviewed and available before dosing on Cycle 0 Day 1 (Part A) [redacted].

Demographics and medical history data will be obtained and recorded as described in Section V1.6.1 of the Core Protocol.

Subjects who fail screening may be re-screened only on approval of the SRC and Sponsor. Any subject re-screened will need to provide new informed consent and will be allocated a new subject number.

V2.6.3 Safety

Part A

Safety and tolerability will be assessed throughout the study by recording of AEs and concomitant medications, clinical laboratory evaluations, physical examinations, ECOG performance status, weight, and vital signs at Screening, Day 1 of Cycle 0 (C0D1), Days 1, 8 and 15 of Cycle 1 (C1), Days 1 and 15 of Cycle 2 (C2), and Day 1 of each subsequent cycle, if required (refer to SoE, Table 12). These parameters will also be assessed at the end of treatment (within 3 days of the last CT7001 dose) and at the End of Study (28 to 35 days after the last CT7001 dose). Triplicate 12-lead ECG measurements will also be taken at Screening, Cycle 0 days, Day 1 and 15 of C1 and C2, and Day 1 of subsequent cycles. Adverse events and concomitant medications will also be recorded on Cycle 0 days.

[redacted]

Adverse Events

Adverse events will be recorded in accordance with the Core Protocol Section V1.7.

Physical Examination

A complete physical examination will be performed on all patients at the time points specified in the Schedules of Events, and as clinically indicated. The results will be entered in the eCRF.

ECOG Performance Status

Performance status will be assessed as specified in the SoE for Module 1 Part A (Table 12) and Part B (Table 13) as follows:

0 = Fully active, able to carry out all predisease activities without restriction.

1 = Restricted in physically strenuous activity but ambulatory and able to carry out work of a light or sedentary nature e.g., light housework, office work.

2 = Ambulatory and capable of self-care but unable to carry out any work activities; up and about more than 50% of waking hours.

3 = Capable of only limited self-care, confined to bed or chair more than 50% of waking hours.

4 = Completely disabled, cannot carry on self-care, totally confined to bed or chair.

The status score will be entered in the eCRF.

Vital Signs

Systolic and diastolic blood pressure and pulse will be measured as specified in the SoE for Module 1, Part A (Table 12) [redacted]. Measurements will be taken after the patient has been resting semi-supine for at least 10 minutes. Temperature, respiratory rate, and oxygen saturation will also be measured. Vital sign measurements will be entered in the eCRF.

Height and Weight

Height and weight will be measured where specified in the relevant SoE and entered in the eCRF.

12-Lead ECG

When specified in the SoE, triplicate 12-lead ECG will be performed 3 to 5 minutes apart after the patient has been resting semisupine for at least 10 minutes. Heart rate, RR interval, PR interval, QRS complex, QT interval, and QTcF will be and entered in the eCRF. The timing of ECG assessments maybe altered depending on the emerging PK and safety profile. Additional ECG assessments may be added if indicated by the emerging data.

Laboratory Safety Assessment

Part A

Haematology (including reticulocyte count), serum chemistry (including tumour specific biomarkers) and coagulation samples will be assessed at Screening, C 0 Day 1 (if screening samples were taken more than 3 days before the start of C0), Days 1, 8 and 15 of C1, Days 1 and 15 of C2, Day 1 of subsequent cycles, and at the End of Treatment visit.

Urine or serum pregnancy will be assessed in women of childbearing potential only at Screening, C0 Day 1 (if screening measurements taken more than 3 days before the start of C0), Day 1 of Cycles 2, 3, 4 etc. (if applicable), and at the End of Treatment visit.

Urinalysis will be performed at Screening, C0 Day 1 (if screening samples were taken more than 3 days before the start of C0), Days 1, 8 and 15 of C1, Days 1 and 15 of Cycle 2, Day 1 of subsequent cycles and at the End of Treatment visit.

[redacted]

[redacted]

Sample Collection

Samples for the following tests will be collected and tested where specified on the relevant SoE:

- Haematology: haemoglobin, platelet count, reticulocyte count (absolute particle count or relative particle count), haematocrit, mean cell volume and red blood cell count (erythrocyte count), white blood cell count with differential (absolute or percent counts of neutrophils, lymphocytes, monocytes, basophils, eosinophils).

- Serum chemistry: albumin, alkaline phosphatase, ALT, AST, bilirubin (total), calcium (total), creatinine, glucose, magnesium, phosphate, potassium, sodium, urea nitrogen or urea, C-reactive protein, chloride, creatine kinase (IU/L), Gamma Glutamyl Transferase (IU/L), Total Protein, specific tumour biomarkers.

- Pregnancy Test (Urine or Serum).

- Urinalysis: blood, glucose, protein.

All results will be entered in the eCRF.

The timing of blood samples specified in the relevant SoE may be altered depending on the emerging PK and safety profiles. Additional sampling times may be added if indicated by the emerging data.

Laboratory values that meet the criteria for at least CTCAE Grade 3 or have changed significantly from Baseline and are considered to be of clinical concern will be repeated within 7 days and followed-up as appropriate. If a decrease in lymphocytes of at least CTCAE Grade 2 occurs, an assessment of lymphocyte populations will be performed.

In the event of an AST or ALT value at least 3 × ULN or total bilirubin at least 2 × ULN, refer to APPENDIX C for further instructions.

V2.6.4 Pharmacokinetics

Collection of Pharmacokinetic Samples

Part A

Blood samples for PK analysis of CT7001 and metabolites, if applicable, will be collected at multiple time points from predose to a specified time postdose before the C1 Day 1 dose and at multiple time points from predose to 24 hours before the C2 Day 2 dose as well as trough samples predose at Day 1 at Cycles 3, 5 etc. (Part A SoE, Table 12).

Any washout period to assess PK and dosing staggering interval between patients may change based upon emerging data as reviewed and agreed by the SRC without the requirement to submit a substantial amendment to the protocol. The frequency of PK sampling may also be modified based on SRC review, and may include up to 3 additional PK samples of 4ml each

within any given cycle and taken by venepuncture, to better characterise the individual PK profile based upon emerging PK data.

Pharmacokinetic sample collection and processing instructions are provided in a separate Laboratory Manual. Collection and dosing times will be entered in the eCRF.

If a patient misses any CT7001 dose within 3 days before PK sampling, the Carrick Physician or Emas Medical Monitor will be contacted to determine change to the timing of the PK samples.

[redacted]

Determination of Drug Concentration in Pharmacokinetic Samples

Concentration of CT7001 and metabolites, if applicable, in PK samples will be quantified by liquid chromatography with tandem mass spectrometry.

V2.6.5 Biomarkers

Peripheral Blood for Pharmacodynamic Biomarker Analysis

Part A

Blood samples for PBMC will be used for biomarker analyses and will be collected at screening, predose at Cycle 0 Day 1, and 24 and 48 hours post dose from Cycle 0 Day1. They will also be collected pre-dose at Cycle 1 Day 8 and Day 15.

For all subsequent Cycles at Day 1 a pre-dose sample will be taken including an End of Treatment sample.

[redacted]

Plasma for Circulating Tumour DNA

Part A

Where consent for genetic analysis has been given, plasma derived from the PBMC blood sample will be processed for the analysis of ctDNA. Note that this does not require an additional blood sample to be taken.

Plasma samples for ctDNA prepared at screening and Cycle 0 Day 1 for Part A.For both parts, samples will be prepared pre-dose on Day 1 of subsequent cycles starting from Cycle 2. In addition, a sample will be prepared at the End of Treatment visit.

[redacted]

Exploratory Research Samples (Part A only)

Blood samples for exploratory research will be taken at Cycle 0 Day 1. Samples will be taken pre-dose on Day 1 of subsequent cycles starting from Cycle 2.

Sample Collection

Sample collection and processing instructions are provided in a separate Laboratory Manual. Collection times will be entered in the eCRF.

Samples will be analysed for predictive biomarkers, changes in genetic alterations, and potential mechanisms of resistance to CT7001 treatment as well as for CDK7 pathway biomarkers that may include but are not limited to phospho-Pol II, Ki67 and Cleaved Caspase.

Pharmacogenetics (Part A only)

A blood sample for pharmacogenetic (PG) analysis, a blood sample will be collected at screening.

Any samples related to PG and genetic analyses will require a separate consent from patients.

Serial Tumour Samples - Optional

For patients with accessible lesions and who have provided consent, collection of fresh serial tumour biopsies is encouraged at the visits specified on the relevant SoE, and collection times will be entered into the eCRF. Timing of these samples may be changed on the basis of emerging PK or PDc data available during the trial. An accessible lesion is defined as a tumour lesion that is amenable to repeat biopsy, unless clinically contraindicated or the patient has withdrawn consent. Failure to obtain sufficient tumour sample after making best efforts to biopsy the tumour will not be considered a protocol deviation.

These samples will be analysed for CDK7 pathway biomarkers that may include but are not limited to phospho-Pol II, Ki67 and Cleaved Caspase.

Collection and processing instructions are provided in a separate Laboratory Manual. Collection times will be entered in the eCRF.

If the patient consents to collection of tumour biopsy samples, these will be collected on an optional basis between up to -7 days pre the first dose. A non-mandatory biopsy will be taken at the End of Treatment visit as soon as possible after confirmation of progression of the disease.

Part A

If the subject consents to collection of tumour biopsy samples, these will be collected on an optional basis between up to -7 days pre the first dose. A non-mandatory biopsy will be taken

at the End of Treatment visit as soon as possible after confirmation of progression of the disease.

[redacted]

Archival Tumour Samples (Part A Only)

A formalin-fixed tumour tissue sample embedded in a paraffin block, if available, may be requested for each patient. Even if baseline biopsy samples can also be collected, retrieval of the archival diagnostic tumour material is still highly encouraged to provide data on how the tumour has evolved since diagnosis. Archival samples from either primary or metastatic tumour will be accepted, but tissue from the primary tumour is preferred. Tissue from the most recent biopsy is preferred for a patient who has archival tissue samples from multiple time points.

Tumour tissue blocks are preferred, but freshly prepared unstained slides (minimum 10, preferably 20) with 4 micron sections from the archival tumour block are accepted if tumour blocks cannot be submitted.

Collection and processing instructions are provided in a separate Laboratory Manual Analyses will include genotyping of the tumour for all patients where archival tissue material is available. Collection times will be entered in the eCRF.

Serial Tumour Samples – Mandatory, Tumour Biopsy Cohort Expansion

Part A

For subjects with accessible breast cancer lesions and who have provided consent, collection of fresh serial tumour biopsies will be collected as specified in the SoE (Table 12) and collection times will be entered into the eCRF. Timing of these samples may be changed on the basis of emerging PK or PDc data available during the trial. An accessible lesion is defined as a tumour lesion that is amenable to repeat biopsy, unless clinically contraindicated or the subject has withdrawn consent. Failure to obtain sufficient tumour sample after making best efforts to biopsy the tumour will be considered a protocol deviation.

[redacted]

Collection and processing instructions are provided in a separate Laboratory Manual. Collection times will be entered in the eCRF.

V2.6.6 Biological Sampling Procedures

Volume of Blood

Part A

The total volume of blood to be collected from patients in Part A in accordance with the SoE (Table 12) is summarised in Table 6 to Table 10.

The number of samples taken, as well as the volume required for each analysis, is dependent on the number of cycles of treatment and may be changed during the study as new data on CT7001 become available. However, the total volume, including screening, Cycle 0 and Cycle 1 and an End of Treatment visit will be approximately, 254 mL. Cycle 2 will be approximately 86mL and each subsequent cycle from Cycle 3 onwards will be approximately 39 mL.

Any washout period to assess PK and dosing staggering interval between patients may change based upon emerging data as reviewed and agreed by the SRCwithout the requirement to submit a substantial amendment to the protocol. The frequency of PK sampling may also be modified based on SRC review, and may include up to three additional PK samples of 4ml each within any given cycle and taken by venepuncture, to better characterise the individual PK profile based upon emerging PK data.

Table 6: Blood Volume – Screening and Cycle 0

Sample Type	Sample Volume (mL)[a]	Number of Samples[f]	Total Volume (mL)
Haematology[b] including reticulocyte count	5	2	10
Serum chemistry[b] including tumour specific biomarkers	5	2	10
Coagulation[b]: INR and aPTT	5	2	10
PK sample (predose-168h)[c]	4	13	52
PG sample[d]	4	1	4
PBMC biomarker analysis[e]	10	5	50
Exploratory research sample	10	1	10
**TOTAL**	**43mL**	**26**	**146mL**

Abbreviations: PG = pharmacogenetic; PK = pharmacokinetic; PBMC = peripheral blood mononuclear cell.

[a] These volumes are intended as a guide and indicate estimated maximums (some sites may utilise smaller tube volumes additional safety blood), samples may be required when clinically indicated.

[b] All samples taken at screening and Cycle 0 Day 1 unless Cycle 0 Day 1 visit within 3 days of the screening laboratory tests.

[c] Samples taken over Cycle 0 Days (Note that the Cycle 0 168 hour postdose sample will fall as pre-dose on Day 1 of Cycle 1)

[d] Only for patients who have separately consented for genetic research.

[e] Screening, pre-dose Cycle 0 Day 1, 6h and 24h Cycle 0 Day 1, 48hs post dose Cycle 0 Day 2

[f] Up to 3 additional PK samples of 4ml may be included within and given cycle and taken by venepuncture to better categorise the individual PK profile based upon emerging PK data.

Table 7: Blood Volume – Cycle 1

Sample Type	Sample Volume (mL)[a]	Number of Samples[c]	Total Volume (mL)
Haematology including reticulocyte count	5	3	15
Serum chemistry including tumour specific biomarkers	5	3	15
Coagulation: INR and aPTT	5	3	15
PK sample (predose)[b]	4	2	8
PBMC biomarker analysis [b]	10	3	30
**TOTAL**	**29mL**	**14**	**83mL**

Abbreviations: PG = pharmacogenetics; PK = pharmacokinetic; PBMC = peripheral blood mononuclear cell.

[a] These volumes are intended as a guide and indicate estimated maximums (some sites may utilise smaller tube volumes additional safety blood), samples may be required when clinically indicated.

[b] Predose Day 8 and 15.

[c] Up to 3 additional PK samples of 4ml may be included within and given cycle and taken by venepuncture to better categorise the individual PK profile based upon emerging PK data.

Table 8: Blood Volume – Cycle 2

Sample Type	Sample Volume (mL)[a]	Number of Samples[f]	Total Volume (mL)
Haematology[b]	5	2	10
Serum chemistry[b]	5	2	10
Coagulation: INR and aPTT[b]	5	2	10
PK – trough samples (predose-24h)[c]	4	9	36
PBMC biomarker analysis [d]	10	1	10
Exploratory research sample[e]	10	1	10
**TOTAL**	**39mL**	**17**	**86mL**

Abbreviations: PG = pharmacogenetic; PK = pharmacokinetic; PBMC = peripheral blood mononuclear cell.

[a] These volumes are intended as a guide and indicate estimated maximums (some sites may utilise smaller tube volumes additional safety blood), samples may be required when clinically indicated.

[c] Cycle 2, Day 1 Predose, 0.5, 1, 1.5, 2, 4, 6, 8 and 24 hour post dose.

[d] Days 1 predose[e] Day 1 of Cycle 2 predose.

[f] Up to 3 additional PK samples of 4ml may be included within and given cycle and taken by venepuncture to better categorise the individual PK profile based upon emerging PK data.

Table 9: Blood Volume – Cycle 3 and subsequent Cycles (Day 1 only)

Sample Type	Sample Volume (mL)[a]	Number of Samples[c]	Total Volume (mL)
Haematology	5	1	5
Serum chemistry	5	1	5
Coagulation: INR and aPTT	5	1	5
PK – trough sample[b]	4	1	4
PBMC biomarker analysis [b]	10	1	10
Exploratory research sample[b]	10	1	10
**TOTAL**	**39mL**	**6**	**39mL**

Abbreviations: PG = pharmacogenetic; PK = pharmacokinetic; PBMC = peripheral blood mononuclear cell.

[a] These volumes are intended as a guide and indicate estimated maximums (some sites may utilise smaller tube volumes additional safety blood), samples may be required when clinically indicated.

[b] Predose.

[c] Up to 3 additional PK samples of 4ml may be included within and given cycle and taken by venepuncture to better categorise the individual PK profile based upon emerging PK data.

Table 10: Blood Volume – End of Treatment Visit

Sample Type	Sample Volume (mL)[a]	Number of Samples	Total Volume (mL)
Haematology	5	1	5
Serum chemistry	5	1	5
Coagulation	5	1	5
PBMC biomarker analysis	10	1	10
**TOTAL**	**25mL**	**4**	**25mL**

Abbreviations: PK = pharmacokinetic; PBMC = peripheral blood mononuclear cell.

[a] These volumes are intended as a guide and indicate estimated maximums (some sites may utilise smaller tube volumes additional safety blood), samples may be required when clinically indicated.

[redacted]

[redacted]

Handling, Storage, and Destruction of Biological Samples, labelling of samples and sample chain of custody is as described in the Core Protocol Section V1.6.6. Instructions are provided in a separate Laboratory Manual.

The procedure that will be following in cases where a patient withdraws their informed consent for the use of voluntarily donated biological samples is defined in Core Protocol Section V1.6.6.

V2.6.7 Anti-tumour Activity

Part A

Antitumour activity will be assessed by CT or MRI scans at Screening, Cycle 3 Day 1 and every 2 cycles (Cycle 5 7 etc.) End of Treatment according to RECIST version 1.1 (Appendix E) (Eisenhauer et al, 2009).

The imaging method used at Screening will be used at each subsequent visit.

Anti-tumour activity will be monitored according to the relevant SoE (Table 12) using procedures as described in Core Protocol Section V1.6.7.

Anti-tumour activity will be assessed locally by the Investigator. The Sponsor reserves the right to request an anonymised independent review of subject scan data if required.

[redacted]

[redacted]

[redacted]

V2.7 EVALUATION AND CALCULATION VARIABLES AND STATISTICAL METHODS

A detailed SAP will be written and finalized before database snapshot for the interim analysis Any deviation from the SAP will be described in the CSR.

Specific to Module 1 Part A, there will be an interim data cut-off after the last visit of the last patient, and after the last visit of the last patient in the last cohort, including any extension cohort(s).

[redacted]

V2.7.1 Definition of Module 1 Study Endpoints

The overall study endpoints were described earlier in Core Protocol Section V1.2.2. Endpoints in addition to these that are specific to Module 1 only are listed below.

Module 1 Primary Endpoints (All Common to Parts A [redacted]

Module 1 primary safety and tolerability endpoints are as stated in the Core Protocol (Section V1.2.2).

Module 1 Secondary Endpoints

PK Parameters for CT7001 will be collected in Module 1, as stated for the Core Protocol (Section V1.2.2).

Specific PK parameters for Module 1, Part A:

Cycle 0 (single dose)

- Plasma concentrations.
- C_{max}: maximum observed plasma concentration.
- C_{24}: plasma concentration at 24 hours.
- T_{max}: time to maximum observed plasma concentration.
- $T_{½}$: apparent terminal half-life.
- λ_z: terminal rate constant.
- AUC_{0-24}: area under the plasma concentration-time curve from Time 0 to 24 hours.
- AUC_{0-48}: area under the plasma concentration-time curve from Time 0 to 48 hours.
- AUC_{0-t}: area under the plasma concentration-time curve from Time 0 to the time of the last measurable concentration.
- $AUC_{0-\infty}$: area under the plasma concentration-time curve from Time 0 extrapolated to infinity.
- CL/F: apparent plasma clearance.
- V_z/F: apparent volume of distribution.
- MRT: mean residence time.

Cycle 2 Day 1

- Plasma Concentrations.
- $C_{ss,max}$: C_{max} at steady state.
- $C_{ss,min}$: minimum observed plasma concentration at steady state.

- $T_{ss,max}$: T_{max} at steady state.
- AUC_{tau}: area under the plasma concentration-time curve in the dosing interval.
- CL_{ss}/F: CL/F at steady state.
- MRT_{ss}: MRT at steady state.

Cycle 0, Cycle 1* and Cycle 2 Day 1

- Dose proportionality.
- TCP: temporal change parameter.
- R_{ac}: accumulation ratio.
- Dose-normalised $C_{max.}$
- Dose-normalised AUCs.
- C_{min}:C_{max} ratio.
- Trough concentrations.
- Time to steady-state.

  * Cycle 1 trough and steady state only

Cycle 3,5,7 (multiple dose)

- Plasma concentrations.
- Trough concentrations.

Exploratory Endpoints

PK Parameters for Major Metabolites

- Metabolite identification.
- Metabolite:CT7001 ratio.

The data will be reported outside the CSR.

A population PDc approach may be used to investigate the relationship between dose, PK and selected primary, secondary and/or exploratory endpoints. This may include investigating the influence of covariates on PK.

The PK results will be reported separately from the CSR. These data may also be combined with similar data from other studies and explored using population PK and/or PK-PD methods and will be reported separately from the Study Report. Met-ID may be performed on pooled samples and will be reported separately.

Predictive Markers and Acquired Resistance to CT7001

- Total and Phosphorylated CDK7.
- Myc oncogene expression.

V2.7.2 Determination of Sample Size

The primary objectives of the dose-escalation phase (Part A) of Module 1 is to investigate the safety and tolerability of CT7001 to enable determination of both the MBAD and MTD of CT7001 and of the dose(s) and schedule(s) for evaluation in later modules.

The sample size of cohorts of three to six patients was based on the requirement for adequate safety, PK, and PDc data balanced with exposure of as few patients as possible to the IMP and study procedures. These cohorts may be expanded by up to 12 additional evaluable patients at doses at or above the minimally biological active dose. The total number of patients in Module 1 Part A will depend on this potential expansion of cohorts and the number of dose escalations conducted.

V2.7.3 Calculation or Derivation of Module 1 Safety Variables

Details will be provided in the SAP.

V2.7.4 Calculation or Derivation of Module 1 Pharmacokinetic Variables

Details will be provided in the SAP.

V2.7.5 Calculation or Derivation of Module 1 Biomarker Variables

Details will be provided in the SAP.

V2.7.6 Calculation or Derivation of Module 1 Exploratory Research Variables

Details will be provided in the SAP.

V2.7.7 Calculation or Derivation of Module 1 Antitumour Activity Variables

Details will be provided in the SAP.

V2.7.8 Description of Analysis Sets

Details will be provided in the SAP.

V2.7.9 Methods of Statistical Analysis

Details will be provided in the SAP.

Pharmacokinetic Analyses

Module 1 PK analyses will be performed according to the SAP.

Biomarker Analyses

Module 1 biomarker analyses will be performed using the Biomarker Population. Details on the method and software will be provided in the laboratory manual.

Laboratory results from for which there are at least 6 patients have data will be summarised by visit and cohort using descriptive statistics. Summaries of change from Baseline and percent change from Baseline will also be summarised by visit and cohort.

Anti-tumour Activity Analyses

Module 1 antitumour activity endpoints will be analysed using the Evaluable for Response Population, as described in the SAP.

Pharmacogenetics and Exploratory Research

This will be conducted in accordance with the Core Protocol, Section V1.8.4.

V2.8 MODULE 1 PART A STUDY TIMETABLE

Table 12: Schedule of Events – Module 1 Part A (Dose Escalation): Each Cycle = 21 Days. Cycle 3 onwards, Day 1 visits only

Visit	Screening	Cycle 0			Cycle 1 (21 days)			Cycle 2 (21 days)		Cycle 3 etc. (21 days)	End of Treatment	End of Study
**Timing of Visit**	**Day -28 to Day -1**	**Day 1[a] single dose**	**Day 2 wash out**	**Day 3-6 PK**	**Day 1**	**Day 8 ±1 day**	**Day 15 ±1 day**	**Day 1 ±1 day**	**Day 15 ±3 days**	**Day 1 ±3 days**	**Within 3 days after last CT7001 dose**	**28 to 35 days after last CT7001 dose**
Informed consent	X											
Medical history[aa]	X											
Adverse events	X	X	X	X	X	X	X	X	X	X	X	X
Concomitant medications (including immunomodulators)	X	X	X	X	X	X	X	X	X	X	X	X
Height[b]	X											
Weight	X	X			X	X	X	X	X	X	X	X
Physical examination	X	X			X	X	X	X	X	X	X	X
ECOG performance status	X	X			X	X	X	X	X	X	X	X
Vital signs	X	X[c]	X		X[d]	X[e]	X[e]	X[d]		X[e]		
Triplicate 12-lead ECGs	X	X[c]	X		X[d]	X[e]	X[e]	X[d]		X[e]		
**Blood/ urine samples**												
Haematology including reticulocyte count	X	X[f]			X	X	X	X	X	X	X	
Serum chemistry including blood bourne tumour markers	X	X[f]			X	X	X	X	X	X	X	
Coagulation (aPTT and INR)	X	X[f]			X	X	X	X	X	X	X	
Pregnancy Test[g]	X	X[f]						X		X	X	
PK blood sample		X[i]	X[i]	X	X[z]	X[j]	X[j]	X[k]		X[l]		
Blood sample for PBMCs biomarker analysis	X	X[m]	X		X[n]	X[j]	X[j]	X[n]		X[p]	X	
Pharmacogenetics blood sample (optional)[q]		X[n]										
Plasma preparation for ctDNA (optional)[q]	X[n]	X[n]						X[n]		X[n]	X	

Table 12: Schedule of Events – Module 1 Part A (Dose Escalation): Each Cycle = 21 Days. Cycle 3 onwards, Day 1 visits only

Visit	Screening	Cycle 0			Cycle 1 (21 days)			Cycle 2 (21 days)		Cycle 3 etc. (21 days)	End of Treatment	End of Study
**Timing of Visit**	Day -28 to Day -1	Day 1[a] single dose	Day 2 wash out	Day 3-6 PK	Day 1	Day 8 ±1 day	Day 15 ±1 day	Day 1 ±1 day	Day 15 ±3 days	Day 1 ±3 days	Within 3 days after last CT7001 dose	28 to 35 days after last CT7001 dose
Blood Samples for Exploratory research		X[n]						X[n]		X[n]		
Urinalysis	X	X[f]			X	X	X				X	
Fresh Tumour biopsy (optional[r])		X[s]						X[t]			X[u]	
Archival Tissue Sample (if available)	X											
Imaging (CT or MRI scan[v])	X									X[h]	X	
IMP administration[w]		X			**Dosing Period**							
Overall survival/PFS[x]				X								
**Breast Cancer Patients Only once MBAD defined: SEPARATE CONSENT REQUIRED (No Cycle 0 will be required unless specified by the SRC, all other schedule of events remain the same with the exception of those below**												
Tumour Biopsy Expansion Cohort (mandatory paired biopsies)		Not applicable			X[y]			X[t]			X	
Plasma preparation for ctDNA (optional)[q]	X[n]	Not applicable			X[n]			X[n]		X[n]	X	
Blood Samples for Exploratory research		Not applicable			X[n]			X[n]		X[n]		
Pharmacogenetics blood sample (optional)[q]		Not applicable			X[n]							

Abbreviations: CT = computed tomography; ECG = electrocardiogram, ECOG = Eastern Cooperative Oncology Group; IMP = investigational medicinal product; MRI = magnetic resonance imaging; PBMC = Peripheral Blood Mononuclear Cells; PG = pharmacogenetic; PK = pharmacokinetic.

[a] All screening procedures must be completed and results available and reviewed before dosing on Cycle 0 Day 1.

[b] Height measured at Screening only

[c] Supine blood pressure and pulse, temperature, respiratory rate, oxygen saturation will be measured predose and at 0.5, 1, 2, 4, and 8 hours postdose. ECGs will be measured predose, 1, 2,4 and 8 hours post dose.

[d] Supine blood pressure and pulse, temperature, respiratory rate, oxygen saturation and ECGs will be measured predose and 1,2,4 and 8 hours post dose

[e] Measure predose only

[f] Laboratory tests do not need to be repeated if visit is within 3 days of Screening samples and are to be collected predose for all dosing days.

[g] For women of childbearing potential only. Urine or serum test allowed.

[h] Scans taken at Cycles 3, 5, 7, etc. a-7 day window is permitted for the imaging.

[i] Collected predose and at 0.5 (+/- 5min), 1, 1.5, 2, 4, 6, 8 (+/-10min), 24 (+/-60min), ,48, 72 (+/-60min), 120 (+/-24h if scheduled over a weekend) and 168 (+/-60min) hours postdose (which occurs before the Cycle 1 Day 1 dose).

[j] Collected predose on Day 8 and Day 15 (Cycle 1)

[k] Collected predose and at 0.5, 1, 1.5, 2, 4, 6, 8 and 24 postdose (before the Cycle 2 Day 2 dose)

[l] Collected predose every other cycle. i.e. Day 1 of Cycle 3, 5 etc.

[m] Collected predose, 24 and 48 hour post dose (matching same time as 24 and 48h post dose PK samples)
[n] Collected predose
[o] Deleted
[p] Collected predose on Day 1 from Cycle 3 and subsequent Cycles.
[q] Only for patients who have separately consented to genetic research.
[r] Only for patients who have consented to collection tumour biopsy samples (part of the main study consent form)
[s] Collected between Day -7 and predose on Cycle 0 Day 1.
[t] Collected on Cycle 2 Day 1, the same day as PK sampling.
[u] Nonmandatory biopsy after confirmation of progression of disease (as soon as possible after disease progression confirmed)
[v] The imaging method (CT or MRI) used at Screening will be used at each subsequent visit.
[w] Patients will take their CT7001 dose in the clinic on day when PK sampling is planned.
[x] Patients are followed up until disease progression. Data on survival will also be checked.
[y] Collected between Day -7 and predose on Cycle 1 Day 1.
[z] This is a Cycle 0 PK sample but noted in table on Cycle 1 Day 1 for completeness
[aa] To include information on prior anticancer treatments, alcohol consumption and tobacco use.

NB: The frequency of dosing, any washout period to assess PK and dosing staggering interval between patients may change based upon emerging data as reviewed and agreed by the SRC without the requirement for a substantial amendment to the protocol The frequency of PK sampling may be changed based on SRC review, and may include up to 3 additional PK samples of 4ml within any given cycle and taken by venepuncture, to better characterise the individual PK profile based upon emerging PK dat

[redacted]

[redacted]

[redacted]

[redacted]

[redacted]

[redacted]

[redacted]

[redacted]

[redacted]

[redacted]

[redacted]

[redacted]

[redacted]

[redacted]

[redacted]

[redacted]

[redacted]

[redacted]

[redacted]

[redacted]

[redacted]

[redacted]

[redacted]

[redacted]

[redacted]

[redacted]

[redacted]

[redacted]

[redacted]

[redacted]

[redacted]

[redacted]

[redacted]

[redacted]

[redacted]

[redacted]

[redacted]

[redacted]

REFERENCES

Ainscow E *et al*. CT7001: An orally bioavailable CDK7 inhibitor is a potential therapy for breast, small-cell lung and haematological cancers. *American Association of Cancer Research Annual Meeting*, 2018; http://www.abstractsonline.com/pp8/#!/4562/presentation/6442

Bahl A *et al*. Activity of CT7001 an orally bio-available cyclin-dependent kinase 7 selective inhibitor in models of triple negative breast cancer. *San Antonio Breast Cancer Conference*, 2017; https://www.abstracts2view.com/sabcs17/view.php?nu=SABCS17L_109&terms

Chen D et al, Activation of estrogen receptor alpha by S118 phosphorylation involves a ligand-dependent interaction with TFIIH and participation of CDK7. Mol Cell, 2000; 6:127–37.

Chen DS, and Mellman I. Oncology meets immunology: the cancer-immunity cycle. *Immunity*. 2013;39:1-10.

Cheson BD, Bennett, JM, Kopecky, KJ, Büchner T, Willman CL, et al. Revised recommendations of the International Working Group for diagnosis, standardization of response criteria, treatment outcomes, and reporting standards for therapeutic trials in acute myeloid leukemia. *J Clin Oncol.* 2003; 21:4642.

Cheung YK, Chappell R. Sequential Designs for Phase I Clinical Trials with Late-Onset Toxicities. *Biometrics*. 2000 Dec. 56 (4): 1177–1182.

Chipumuro E, Marco E, Christensen CL, Kwiatkowski N, Zhang T, et al. CDK7 inhibition suppresses super-enhancer-linked oncogenic transcription in MYCN-driven cancer. *Cell.* 2014 Nov 20;159(5):1126-39. doi: 10.1016/j.cell.2014.10.024. Epub 2014 Nov 6.

Choi YJ, Hu S, Ke N, Ren Y, Lopez J, et al. C. First-in-class CDK7 inhibitor, induces robust apoptosis in acute myeloid leukemia and demonstrates durable *in vivo* efficacy. In: *Haematologica.* 2016 Jun 1 (101): 211-211. Via Giuseppe Belli 4, 27100 Pavia, Italy: Ferrata Storti Foundation.

Christensen CL, Kwiatkowski N, Abraham BJ, Carretero J, Al-Shahrour F, et al. Targeting Transcriptional Addictions in Small Cell Lung Cancer with a Covalent CDK7 Inhibitor. *Cancer Cell*. 2014: 26 (6); 909–922.

Clark K et al. CT7001, a novel orally bio-available CDK7 inhibitor, is highly active in in-vitro and in-vivo models of AML. American Society of Hematology, 2017; http://www.bloodjournal.org/content/130/Suppl_1/2645

Dukelow T, Kishan D, Khasraw M, and Murphy CG. CDK4/6 inhibitors in breast cancer. *Anticancer Drugs*. 2015 Sep;26(8):797-806.

Eisenhauer EA, Therasse P, Bogaerts J, Schwartz LH, Sargent D, et al. New response evaluation criteria in solid tumours: revised RECIST guideline (version 1.1). *Eur J Cancer.* 2009;45:228-47.

Farkona S, Diamandis EP, and Blasutig IM. Cancer immunotherapy: the beginning of the end of cancer? *BMC Med*. 2016 May 5;14:73.

Feaver WJ et al. Relationship of CDK-activating kinase and RNA polymerase II CTD kinase TFIIH/TFIIK. Cell, 1994; 79:1103–1109.

Fisher RP, Morgan DO. A novel cyclin associates with MO15/CDK7 to form the CDK-activating kinase. Cell, 1994; 78:713–724.

Fisher RP. Secrets of a double agent: CDK7 in cell-cycle control and transcription. J Cell Sci, 2005; 118:5171–80.

Glover-Cutter K et al. TFIIH-associated Cdk7 kinase functions in phosphorylation of C-terminal domain Ser7 residues, promoter-proximal pausing, and termination by RNA polymerase II. Molecular and cellular biology, 2009; 29:5455-5464

Homet Moreno B, and Ribas A. Anti-programmed cell death protein-1/ligand-1 therapy in different cancers. *Br J Cancer*. 2015 Apr 28;112(9):1421-7.

Hu S, Ke N, Ren Y, Lopez J, Miljovska S, et al. Selective CDK7 inhibitors suppress super enhancer-genes, induce massive apoptosis in acute myeloid leukemia and demonstrate durable *in vivo* efficacy [abstract]. In: Proceedings of the 107th Annual Meeting of the American Association for Cancer Research; 2016 Apr 16-20; New Orleans, LA. Philadelphia (PA): AACR; *Cancer Res.* 2016;76(14 Suppl):Abstract nr 4820.

Hübel J, and Hieronymus T. HGF/Met-Signaling Contributes to Immune Regulation by Modulating Tolerogenic and Motogenic Properties of Dendritic Cells. *Biomedicines*. 2015, 3(1), 138-148.

Ivy SP, Siu LL, Garrett-Mayer E, and Rubinstein L. Approaches to Phase 1 Clinical Trial Design Focused on Safety, Efficiency, and Selected Patient Populations: A Report from the Clinical Trial Design Task Force of the National Cancer Institute Investigational Drug Steering Committee. *Clin Cancer Res.* 2010 Mar 15;16(6):1726-36. doi: 10.1158/1078-0432.CCR-09-1961. Epub 2010 Mar 9.

Kwiatkowski N, Zhang T, Rahl PB, Abraham BJ, Reddy J, et al. Targeting transcription regulation in cancer with a covalent CDK7 inhibitor. *Nature.* 2014;511:616-20.

Mellman I, Coukos G, and Dranoff G. Cancer immunotherapy comes of age. *Nature.* 2011;480:480-9.

Murray IA, Patterson AD, and Perdew GH. Aryl hydrocarbon receptor ligands in cancer: friend and foe. *Nat Rev Cancer*. 2014 Dec;14(12):801-14;

Nuhn *et al*. Update on Systemic Prostate Cancer Therapies: Management of Metastatic Castration-resistant Prostate Cancer in the Era of Precision Oncology. European Urology 2019, Volume 75, Issue 1, Pages 88–99

O'Quigley, J, Pepe, M, and Fisher, L. Continual reassessment method: a practical design for Phase 1 clinical trials in cancer. *Biometrics* 1990; 33-48.

Osborne CK, Pippen J, Jones SE, Parker LM, Ellis M, et al. Double-blind, randomized trial comparing the efficacy and tolerability of fulvestrant versus anastrozole in postmenopausal women with advanced breast cancer progressing on prior endocrine therapy: results of a North American trial. *Journal of Clinical Oncology.* 2002; 20(16):3386–3395.

Patel H *et al*. ICEC0942, an Orally Bioavailable Selective Inhibitor of CDK7 for Cancer Treatment. *Mol Cancer Ther.* 2018 Jun;17(6):1156-1166.

Postow MA, Callahan MK, Barker CA, Yamada Y, Yuan J, et al. Immunologic correlates of the abscopal effect in a patient with melanoma. *N Engl J Med.* 2012; 366(10):925-31.

Rubin LG, Levin MJ, Ljungman P, et al. 2013 IDSA Clinical Practice Guideline for Vaccination of the Immunocompromised Host. Clinical Infectious Diseases 2014;58(3):e44–100.

Schachter MM, Fisher RP. The CDK-activating kinase Cdk7: taking yes for an answer. Cell Cycle, 2013; 12:3239–40

Schachter MM et al. A Cdk7-Cdk4 T-loop phosphorylation cascade promotes G1 progression. Mol Cell, 2013; 50:250–260

Seymour L; Ivy SP, Sargent D, Spriggs S, Baker L, et al. The Design of Phase II Clinial trials Testing Cancer Therapeutics: Consensus Recommendations from the Clinical Trial Design Task Force of the National Cancer Institute Investigational Drug Steering Committee. *Clin Cancer Res.* 2010 March 15; 16(6):1764-1769.

Song IS, Lee DY, Shin MH, Kim H, Ahn YG, et al. Pharmacogenetics meets metabolomics: Discovery of tryptophan as a new endogenous OCT2 substrate related to metformin disposition. *PLoS One*. 2012; 7(5): e36637. Published online 2012 May 8.

Verma S, Bartlett CH, Schnell P, DeMichele AM, Loi S, et al. Palbociclib in Combination With Fulvestrant in Women With Hormone Receptor-Positive/HER2-Negative Advanced Metastatic Breast Cancer: Detailed Safety Analysis From a Multicenter, Randomized, Placebo-Controlled, Phase III Study (PALOMA-3). *Oncologist.* 2016 Oct;21(10):1165-1175.

Wang Y, Zhang T, Kwiatkowski N, Abraham BJ, Lee TI, et al. CDK7-dependent transcriptional addiction in triple-negative breast cancer. *Cell.* 2015 Sep 24;163(1):174-86. doi: 10.1016/j.cell.2015.08.063.

Wölwer CB *et al.* A Chemical Screening Approach to Identify Novel Key Mediators of Erythroid Enucleation. *PLoS One*, 2015, Nov 16; 10(11):e0142655.

APPENDIX A INTERPRETATION OF AE CAUSALITY

Causality: For each AE and SAE, an assessment must be made of the possibility that it was caused by the IMP(s). All cases judged by the Investigator or the Carrick Physician as having a "reasonable possibility of a causal relationship between the events and the IMPs" qualify as adverse drug reactions.

The relationship of each AE to IMPs, will be assessed as No or Yes, in answer to the question "Is there a reasonable possibility of a causal relationship between the event and the IMPs?"

- **No.** The time course between the administration of IMP and the occurrence or worsening of the AE rules out a causal relationship and another cause (concomitant drugs, therapies, complications, etc.) is suspected.

- **Yes.** The time course between the administration of IMP and the occurrence or worsening of the AE is consistent with a causal relationship and no other cause (concomitant drugs, therapies, complications, etc.) can be identified.

The following factors should also be considered:

- The temporal sequence from IMP administration, the event should occur after the IMP is given. The length of time from investigational product exposure to event should be evaluated in the clinical context of the event.

- Underlying, concomitant and intercurrent diseases. Each report should be evaluated in the context of the natural history and course of the disease being treated and any other disease the patient may have.

- Concomitant medication, the other medications the patient is taking or the treatment the patient receives, should be examined to determine whether any of them might be recognised to cause the event in question.

- The known response pattern for this class of IMP. Clinical and/or preclinical data may indicate whether a particular response is likely to be a class effect.

- Exposure to physical and/or mental stresses, and/or the exposure to stress, which might induce adverse changes in the recipient and provide a logical and better explanation for the event.

The pharmacology and PK of the IMP. The known pharmacologic properties (e.g. absorption, distribution, metabolism, and excretion) of the IMP should be considered.

APPENDIX B MEDICATIONS, HERBAL SUPPLEMENTS AND FOODS THAT SIGNIFICANTLY INDUCE OR INHIBIT CYTOCHROME P450 3A4 OR P-GLYCOPROTEIN ACTIVITY

AB.1 STRONG CYP3A4, CYP2C19 AND/OR CYP2D6 INHIBITORS

The drugs in Table AC-1 are known to strongly inhibit CYP3A4, CYP2C19 and/or CYP2D6 and should be avoided while administering CT7001 (FDA Drug Development and Drug Interactions: Table of Substrates, Inhibitors and Inducers, and the Flockhart Table, Indiana University School of Medicine). The list is not exhaustive, and a similar restriction will apply to other agents that are known to strongly modulate CYP3A4, CYP2C19 and/or CYP2D6. Medical judgement is required but **please be vigilant for signs and/or changes in tolerability particularly with 3A4 substrates.**

Please contact the Emas Medical Monitor with any queries you have on this issue.

Table AC-1: Strong CYP3A4, CYP2C19 and/or CYP2D6 Inhibitors

CYP3A4 Inhibitor	CYP2C19 Inhibitor	CYP2D6 Inhibitor
Clarithromycin,	Fluconazole	Bupropion
Telithromycin,	Fluoxetine	Cinacalcet
Troleandomycin	Fluvoxamine	Fluoxetine
Indinavir	Ticlopidine	Paroxetine
lopinavir	Voriconzole	Quinidine
Nelfinavir		Terbinafine
Ritonavir		
Saquinavir		
Tipranavir		
Telaprevir		
Itraconazole		
Ketoconazole		
Posaconazole		
Voriconazole		
Suboxone		
Nefadozone		
Boceprivir		
Conivaptan		
Cobicistat		
Danoprevir		

Table AC-1: Strong CYP3A4, CYP2C19 and/or CYP2D6 Inhibitors

CYP3A4 Inhibitor	CYP2C19 Inhibitor	CYP2D6 Inhibitor
Elvitegravir		
Grapefruit juice		
Paritaprevir		
Idelalisib		
Diltiazem		
Nelfinavir		

AB.2 STRONG CYP3A4, CYP2C19 AND/OR CYP2D6 INDUCERS

The drugs in Table AC-2 are known to strongly induce CYP3A4, CYP2C19 and/or CYP2D6 and should be avoided while administering CT7001 (FDA Drug Development and Drug Interactions: Table of Substrates, Inhibitors and Inducers, and the Flockhart Table, Indiana University School of Medicine). The list is not exhaustive, and a similar restriction will apply to other agents that are known to strongly induce CYP3A4, CYP2C19 and/or CYP2D6. Medical judgement is required.

Please contact the Emas Medical Monitor with any queries you have on this issue.

Table AC-2: Strong CYP3A4, CYP2C19 and/or CYP2D6 Inducers

CYP3A4 Inducer	CYP2C19 Inducer	CYP2D6 Inducer
Carbamazepine	Aprepitant	None known
Enzalutamide	Carbamazepine	
Mitotane	Enzalutamide	
Phenytoin	Rifampin	
Rifampin	Ritonavir	
St. John's wort	Nevirapine	
Phenobarbital	Phentobarbital	
Rifabutin	St John's Wort	
Nevirapine		
Troglitazone		

AB.2.1 Drugs whose clearance are dependent on CYP3A4 and have a narrow therapeutic index

There are currently no data confirming that there is a PK interaction between CT7001 and other drugs. However, *in vitro* data suggests CT7001 has the potential to cause drug interactions at the intestinal and hepatic level through CYP3A4.

CT7001 shows a weak potential to inhibit CYP2D6 and 2C19 which is unlikely to be clinically significant but should be considered when co-prescribing sensitive 2D6 and 2C19 substrates and substrates with narrow therapeutic index (e.g. S-mephenytoin). The potential for CT7001 to inhibit transporter systems is currently unknown.

If CT7001 is co-administered with CYP3A substrates with narrow therapeutic indices, including but not limited to alfentanil, cisapride, cyclosporine, ergot derivatives, fentanyl, pimozide, quinidine, sirolimus, tacrolimus, atorvastatin, lovastatin and simvastatin the patient should be closely monitored for signs of changed tolerability as a result of increased exposure of the concomitant medication. This list is not intended to be exhaustive, and similar precautions should be applied to other agents that are known to depend on CYP3A4 for metabolism.

Medical judgement is required. Please contact the Emas medical monitor with any queries you have on this issue.

AB.3 P-GLYCOPROTEIN (PGP) INHIBITORS AND INDUCERS

The drugs in Table AC-3 are known to strongly inhibit or induce P-Glycoprotein (PGP) and should be avoided while administering CT7001 (FDA Drug Development and Drug Interactions: Table of Substrates, Inhibitors and Inducers, and the Flockhart Table, Indiana University School of Medicine). The list is not exhaustive, and a similar restriction will apply to other agents that are known to strongly induce PGP. Medical judgement is required.

Please contact the Emas Medical Monitor with any queries you have on this issue.

Table AC-3: P-Glycoprotein (PGP) Inhibitors and Inducers

PGP Inhibitors	PGP Inducers
Amiodarone	Avasimibe
Carvedilol	Carbamazepine
Clarithromycin	Phenytoin
Dronedarone	Rifampin
Itraconazole	Ritonavir
Lapatinib	St. John's Wort
Lovinavir	Tipranavir
Ritonavir	
Propafenone	
Quinidine	
Ranolazine	
Ritonavir	
Saquinavir	
Telaprivir	
Tipranavir	

Table AC-3: P-Glycoprotein (PGP) Inhibitors and Inducers

PGP Inhibitors	PGP Inducers
Verapamil	

APPENDIX C ACTIONS REQUIRED IN CASES OF COMBINED INCREASE OF AMINOTRANSFERASE AND TOTAL BILIRUBIN – HY'S LAW

AC.1 BACKGROUND

Hy's law is a rule that states that a patient is at high risk of a fatal drug-induced liver injury if given a medication that causes hepatocellular injury (not cholestatic injury) with jaundice. The law is based on observations by Hy Zimmerman, a major scholar of drug-induced liver injury.

AC.1.1 Investigator Accountabilities

Each Investigator, or delegate, will regularly review the patient's laboratory data for increases in liver biochemistry parameters pertaining to cases of Hy's Law (HL). It is the Investigators responsibility to assess whether a patient meets Potential Hy's Law (PHL) criteria at any point during the CT7001_001 Study.

The Investigator is responsible for recording data related PHL/HL cases and for reporting AEs and SAEs as per the processes outlined in Section V1.7.3.2 of the CT7001_001 Clinical Study Protocol.

The assessment of PHL cases may be carried out in conjunction with representatives from Carrick.

Definitions

AC.1.2 Potential HY's Law (PHL)

- Aspartate Aminotransferase (AST) or Alanine Aminotransferase (ALT) $\geq 3 \times$ ULN and Total Bilirubin (TBL) $\geq 2 \times$ ULN at any point during the study irrespective of an increase in Alkaline Phosphatase (ALP).

AC.1.3 Hy's Law (HL)

- AST or ALT $\geq 3 \times$ ULN and TBL $\geq 2 \times$ ULN, where no other reason, other than the IMP, can be found to explain the combination of increases, e.g., elevated ALP indicating cholestasis, viral hepatitis, another drug.

PHL and HL elevations do not have to occur at the same time or within a specified time frame.

Identification of Potential Hy's Law Cases

Laboratory data from patients participating in the CT7001_001 study should be assessed to identify occurrence of the following criteria:

- Alanine aminotransferase $\geq 3 \times$ ULN
- Aspartate aminotransferase $\geq 3 \times$ ULN
- Total bilirubin $\geq 2 \times$ ULN

The Investigator, or delegate, will assess follow-up laboratory reports to determine if PHL/HL criteria are met. The Investigator, or delegate, will enter the laboratory data into the CT7001_001 clinical study database to facilitate review by the SRC and / or Sponsor as necessary.

PHL Follow-up Review and Assessment

AC.1.4 If PHL criteria are not met:

If the patient does not meet PHL criteria the Investigator will conduct follow-up on subsequent laboratory results as per the Clinical Study Protocol.

AC.1.5 If PHL criteria are met:

If the patient meets PHL criteria the Investigator will immediately notify the CT7001_001 Study Team Physician and the Emas Drug Safety Physician

The Investigator, in conjunction with the CT7001_001 Study Team Physician and the Emas Drug Safety Physician will decide on treatment options to manage cases PHL, to include:

- Ongoing assessment of LFTs and associated clinical symptoms until values return to normal ranges (as assessed by the Investigator).

If the PHL case meets serious criteria, report the event as an SAE using standard reporting procedures outlined in the CT7001_001 Clinical Study Protocol (assessment of seriousness will be performed in conjunction with the Study Team Physician.

AC.2 FOLLOW-UP, REVIEW AND ASSESSMENT OF PHL

AC.2.1 Cases

- No later than 3 weeks after the first LFT abnormality was detected, the Investigator, in conjunction with the CT7001_001 Study Team Physician will assess the case and decide the case is a drug induced liver injury (DILI) caused by the IMP. The Emas Drug Safety Physician may also be involved in this review.

Following the review and assessment, the Investigator will follow the instructions below.

- If there is an agreed alternative explanation for the ALT or AST and TBL elevations, a determination of whether the alternative explanation is an AE will be made and subsequently whether the AE meets the criteria for a SAE as defined in the Core Protocol Section V1.7.3.2.

- If the alternative explanation is not an AE, record the alternative explanation on the appropriate eCRF.

If the alternative explanation is an AE/SAE, record the AE /SAE in the eCRF accordingly, and follow standard reporting processes outlines in the CT7001_001 Clinical Study Protocol. If it is agreed that there is no explanation that would explain the ALT or AST and TBL elevations other than the IMP:

- Report an SAE (report term ‘Hy’s Law’) according to the standard reporting processes outlined in the CT7001_001 Clinical Study Protocol.

- The 'Medically Important' serious criterion should be used if no other serious criteria apply.
- As there is no alternative explanation for the HL case, a causality assessment of 'related' should be assigned.

AC.2.2 Actions Required for Repeat Episodes of PHL

When a patient meets PHL criteria on more than one instance, investigator should determine the cause for the previous occurrence of PHL criteria being met and answer the following question:

- Was the alternative cause for the previous occurrence of PHL criteria being metchronic or progressing malignant disease?
- If **'No'**: follow the reporting process described in this Appendix.
- If **'Yes'**: determine if there has been a significant change in the patient's condition compared with when PHL criteria were previously met.
- If there is no significant change no action is required.

  If there is a significant change, then the reporting process described in this Appendix should be followed (a 'significant' change in the patient's condition refers to a clinically relevant change in any of the individual liver biochemistry parameters (ALT, AST or total bilirubin) in isolation or in combination, or a clinically relevant change in associated symptoms. The determination of whether there has been a significant change will be at the discretion of the Investigator; this may be in consultation with the CT7001_001 Study Team Physician.

References

FDA Guidance for Industry (issued July 2009) 'Drug-induced liver injury: Premarketing clinical evaluation':

http://www.fda.gov/downloads/Drugs/GuidanceComplianceRegulatoryInformation/Guidances/UCM174090.pdf

APPENDIX D DRUGS KNOWN TO PREDISPOSE TO TORSADE DE POINTES

Generic Name	Brand Name(s)
Amiodarone	Cordarone®, Pacerone®
Arsenic trioxide	Trisenox®
Astemizole	Hismanal®
Azithromycin	Zithromax®
Bepridil	Vascor®
Chloroquine	Aralen®
Chlorpromazine	Thorazine®
Cisapride	Propulsid®
Citalopram	Celexa®
Clarithromycin	Biaxin®
Disopyramide	Norpace®
Dofetilide	Tikosyn®
Domperidone	Motilium®
Droperidol	Inapsine®
Erythromycin	Erythrocin®, E.E.S. ®
Flecainide	Tambocor®
Halofantrine	Halfan®
Haloperidol	Haldol®
Ibutilide	Corvert®
Levomethadyl	Orlaam®
Mesoridazine	Serentil®
Methadone	Dolophine®, Methadose®
Moxifloxacin	Avelox®
Ondansetron*	Zofran®
Pentamidine	Pentam®, NebuPent®
Pimozide	Orap®

Generic Name	Brand Name(s)
Probucol	Lorelco®
Procainamide	Pronestyl®, Procan®
Quinidine	Cardioquin®, Quinaglute®
Sotalol	Betapace®
Sparfloxacin	Zagam®
Terfenadine	Seldane®
Thioridazine	Mellaril®
Vandetanib	Caprelsa®

* when administered intravenously at high dose (32 mg)

Adapted from the University of Arizona Cancer Center for Education and Research on Therapeutics: "Torsades List: Drugs with a Risk of Torsades de Pointes," drugs that are generally accepted by the QTdrugs.org Advisory Board to carry a risk of Torsades de Pointes on the University of Arizona CERT website: http://www.crediblemeds.org/.

This list is not meant to be considered all inclusive. See website for current list.

APPENDIX E RESPONSE EVALUATION CRITERIA IN SOLID TUMOURS (RECIST) VERSION 1.1

Adapted from E.A. Eisenhauer, et al: New response evaluation criteria in solid tumours: Revised RECIST guideline (version 1.1). European Journal of Cancer 45 (2009) 228–247.

CATEGORIZING LESIONS AT BASELINE

Measurable Lesions

Lesions that can be accurately measured in at least one dimension.

- Lesions with longest diameter twice the slice thickness and at least 10 mm or greater when assessed by CT or MRI (slice thickness 5-8 mm).
- Lesions with longest diameter at least 20 mm when assessed by Chest X-ray.
- Superficial lesions with longest diameter 10 mm or greater when assessed by caliper.
- Malignant lymph nodes with the short axis 15 mm or greater when assessed by CT.

NOTE: The shortest axis is used as the diameter for malignant lymph nodes, longest axis for all other measurable lesions.

Non-Measurable Disease

Non-measurable disease includes lesions too small to be considered measurable (including nodes with short axis between 10 and 14.9 mm) and truly non-measurable disease such as pleural or pericardial effusions, ascites, inflammatory breast disease, leptomeningeal disease, lymphangitic involvement of skin or lung, clinical lesions that cannot be accurately measured with calipers, abdominal masses identified by physical examination that are not measurable by reproducible imaging techniques.

- Bone disease: Bone disease is non-measurable with the exception of soft tissue components that can be evaluated by CT or MRI and meet the definition of measurability at baseline.
- Previous local treatment: A previously irradiated lesion (or lesion patiented to other local treatment) is non-measurable unless it has progressed since completion of treatment.

Normal Sites

- Cystic lesions: Simple cysts should not be considered as malignant lesions and should not be recorded either as target or non-target disease. Cystic lesions thought to represent cystic metastases can be measurable lesions if they meet the specific definition above. If non-cystic lesions are also present, these are preferred as target lesions.
- Normal nodes: Nodes with short axis <10 mm are considered normal and should not be recorded or followed either as measurable or non-measurable disease.

RECORDING TUMOUR ASSESSMENTS

All sites of disease must be assessed at baseline. Baseline assessments should be done as close as possible prior to study start. For an adequate baseline assessment, all required scans must be done within 28 days prior to treatment and all disease must be documented appropriately. If baseline assessment is inadequate, subsequent statuses generally should be indeterminate.

Target Lesions

All measurable lesions up to a maximum of 2 lesions per organ, 5 lesions in total, representative of all involved organs, should be identified as target lesions at baseline. Target lesions should be selected on the basis of size (longest lesions) and suitability for accurate repeated measurements. Record the longest diameter for each lesion, except in the case of pathological lymph nodes for which the short axis should be recorded. The sum of the diameters (longest for non-nodal lesions, short axis for nodal lesions) for all target lesions at baseline will be the basis for comparison to assessments performed on study.

- If two target lesions coalesce the measurement of the coalesced mass is used. If a large target lesion splits, the sum of the parts is used.
- Measurements for target lesions that become small should continue to be recorded. If a target lesion becomes too small to measure, 0 mm should be recorded if the lesion is considered to have disappeared; otherwise a default value of 5 mm should be recorded.

NOTE: When nodal lesions decrease to <10 mm (normal), the actual measurement should still be recorded.

Non-Target Disease

All non-measurable disease is non-target. All measurable lesions not identified as target lesions are also included as non-target disease. Measurements are not required but rather assessments will be expressed as ABSENT, INDETERMINATE, PRESENT/NOT INCREASED, INCREASED. Multiple non-target lesions in one organ may be recorded as a single item on the case report form (e.g. ‘multiple enlarged pelvic lymph nodes’ or ‘multiple liver metastases’).

OBJECTIVE RESPONSE STATUS AT EACH EVALUATION

Disease sites must be assessed using the same technique as baseline, including consistent administration of contrast and timing of scanning. If a change needs to be made the case must be discussed with the radiologist to determine if substitution is possible. If not, subsequent objective statuses are indeterminate.

Target Disease

- Complete Response (CR): Complete disappearance of all target lesions with the exception of nodal disease. All target nodes must decrease to normal size (short axis < 10 mm). All target lesions must be assessed.
- Partial Response (PR): Greater than or equal to 30% decrease under baseline of the sum of diameters of all target measurable lesions. The short diameter is used in the sum for target nodes, while the longest diameter is used in the sum for all other target lesions. All target lesions must be assessed.

- Stable Disease (SD): Does not qualify for CR, PR or Progression. All target lesions must be assessed. Stable can follow PR only in the rare case that the sum increases by less than 20% from the nadir, but enough that a previously documented 30% decrease no longer holds.
- Progression of Disease (PD): 20% increase in the sum of diameters of target measurable lesions above the smallest sum observed (over baseline if no decrease in the sum is observed during therapy), with a minimum absolute increase of 5 mm.
- Indeterminate. Progression has not been documented, and:
  - One or more target measurable lesions have not been assessed.
  - Or assessment methods used were inconsistent with those used at baseline.
  - Or one or more target lesions cannot be measured accurately (e.g., poorly visible unless due to being too small to measure).
  - Or one or more target lesions were excised or irradiated and have not reappeared or increased.

Non-Target Disease

- CR: Disappearance of all non-target lesions and normalization of tumour marker levels. All lymph nodes must be 'normal' in size (<10 mm short axis).
- Non-CR/Non-PD: Persistence of any non-target lesions and/or tumour marker level above the normal limits.
- PD: Unequivocal progression of pre-existing lesions. Generally, the overall tumour burden must increase sufficiently to merit discontinuation of therapy. In the presence of SD or PR in target disease, progression due to unequivocal increase in non-target disease should be rare.

Indeterminate: Progression has not been determined and one or more non-target sites were not assessed or assessment methods were inconsistent with those used at baseline.

New Lesions

The appearance of any new unequivocal malignant lesion indicates PD. If a new lesion is equivocal, for example due to its small size, continued assessment will clarify the etiology. If repeat assessments confirm the lesion, then progression should be recorded on the date of the initial assessment. A lesion identified in an area not previously scanned will be considered a new lesion.

Supplemental Investigations

- If CR determination depends on a residual lesion that decreased in size but did not disappear completely, it is recommended the residual lesion be investigated with biopsy or fine needle aspirate. If no disease is identified, objective status is CR.
- If progression determination depends on a lesion with an increase possibly due to necrosis, the lesion may be investigated with biopsy or fine needle aspirate to clarify status.

Patientive Progression

Patients requiring discontinuation of treatment without objective evidence of disease progression should not be reported as PD on tumour assessment eCRFs. This should be indicated on the end of treatment eCRF as off treatment due to Global Deterioration of Health Status. Every effort should be made to document objective progression even after discontinuation of treatment.

Appendix 3 Table 1. Objective Response Status at Each Evaluation

Target Lesions	Non-Target Disease	New Lesions	Objective Status
CR	CR	No	CR
CR	Non-CR/Non-PD, Indeterminate or Missing	No	PR
PR	Non-CR/Non-PD, Indeterminate or Missing	No	PR
SD	Non-CR/Non-PD, Indeterminate or Missing	No	SD
Indeterminate or Missing	Non-PD	No	Indeterminate
PD	Any	Yes or No	PD
Any	PD	Yes or No	PD
Any	Any	Yes	PD

APPENDIX F NEW YORK HEART ASSOCIATION (NYHA) FUNCTIONAL CLASSIFICATION

NYHA Functional Classification

**NYHA Class**	**Patients with Cardiac Disease (Description of HF Related Symptoms)**
Class I (Mild)	Patients with cardiac disease but without resulting in limitation of physical activity. Ordinary physical activity does not cause undue fatigue, palpitation (rapid or pounding heart beat), dyspnea (shortness of breath), or anginal pain (chest pain).
Class II (Mild)	Patients with cardiac disease resulting in slight limitation of physical activity. They are comfortable at rest. Ordinary physical activity results in fatigue, palpitation, dyspnea, or anginal pain
Class III (Moderate)	Patients with cardiac disease resulting in marked limitation of physical activity. They are comfortable at rest. Less than ordinary activity causes fatigue, palpitation, dyspnea, or anginal pain.
Class IV (Severe)	Patients with cardiac disease resulting in the inability to carry on any physical activity without discomfort. Symptoms of heart failure or the anginal syndrome may be present even at rest. If any physical activity is undertaken, discomfort is increased.

The Criteria Committee of the New York Heart Association. *Nomenclature and Criteria for Diagnosis of Diseases of the Heart and Great Vessels*. 9th ed. Boston, Mass: Little, Brown & Co; 1994:253-256.

APPENDIX G FULVESTRANT SPC

The SPC for Fulvestrant 250 mg Solution for Injection (EU/1/03/269/001) is presented on the following pages.

1. NAME OF THE MEDICINAL PRODUCT

Faslodex 250 mg solution for injection.

2. QUALITATIVE AND QUANTITATIVE COMPOSITION

One pre-filled syringe contains 250 mg fulvestrant in 5 ml solution.

Excipients with known effect
For the full list of excipients, see section 6.1.

3. PHARMACEUTICAL FORM

Solution for injection.

Clear, colourless to yellow, viscous solution.

4. CLINICAL PARTICULARS

4.1 Therapeutic indications

Faslodex is indicated for the treatment of postmenopausal women with estrogen receptor positive, locally advanced or metastatic breast cancer for disease relapse on or after adjuvant anti-estrogen therapy, or disease progression on therapy with an anti-estrogen.

4.2 Posology and method of administration

Posology
Adult females (including Elderly)
The recommended dose is 500 mg at intervals of one month, with an additional 500 mg dose given two weeks after the initial dose.

Special populations
Renal impairment
No dose adjustments are recommended for patients with mild to moderate renal impairment (creatinine clearance ≥30 ml/min). Safety and efficacy have not been evaluated in patients with severe renal impairment (creatinine clearance <30 ml/min), and, therefore, caution is recommended in these patients (see section 4.4).

Hepatic impairment
No dose adjustments are recommended for patients with mild to moderate hepatic impairment. However, as fulvestrant exposure may be increased, Faslodex should be used with caution in these patients. There are no data in patients with severe hepatic impairment (see sections 4.3, 4.4 and 5.2).

Paediatric population
The safety and efficacy of Faslodex in children from birth to 18 years of age have not been established. Currently available data are described in sections 5.1 and 5.2, but no recommendation on a posology can be made.

Method of administration
Faslodex should be administered as two consecutive 5 ml injections by slow intramuscular injection (1-2 minutes/injection), one in each buttock (gluteal area).

Caution should be taken if injecting Faslodex at the dorsogluteal site due to the proximity of the

underlying sciatic nerve.

For detailed instructions for administration, see section 6.6.

4.3 Contraindications

Hypersensitivity to the active substance or to any of the excipients listed in section 6.1.
Pregnancy and lactation (see section 4.6).
Severe hepatic impairment (see sections 4.4 and 5.2).

4.4 Special warnings and precautions for use

Faslodex should be used with caution in patients with mild to moderate hepatic impairment (see sections 4.2, 4.3 and 5.2).

Faslodex should be used with caution in patients with severe renal impairment (creatinine clearance less than 30 ml/min).

Due to the intramuscular route of administration, Faslodex should be used with caution if treating patients with bleeding diatheses, thrombocytopenia or those taking anticoagulant treatment.

Thromboembolic events are commonly observed in women with advanced breast cancer and have been observed in clinical trials with Faslodex (see section 4.8). This should be taken into consideration when prescribing Faslodex to patients at risk.

Injection site related events including sciatica, neuralgia, neuropathic pain, and peripheral neuropathy have been reported with Faslodex injection. Caution should be taken while administering Faslodex at the dorsogluteal injection site due to the proximity of the underlying sciatic nerve (see sections 4.2 and 4.8).

There are no long-term data on the effect of fulvestrant on bone. Due to the mechanism of action of fulvestrant, there is a potential risk of osteoporosis.

Interference with estradiol antibody assays
Due to the structural similarity of fulvestrant and estradiol, fulvestrant may interfere with antibody based-estradiol assays and may result in falsely increased levels of estradiol.

Paediatric population
Faslodex is not recommended for use in children and adolescents as safety and efficacy have not been established in this group of patients (see section 5.1).

4.5 Interaction with other medicinal products and other forms of interaction

A clinical interaction study with midazolam (substrate of CYP3A4) demonstrated that fulvestrant does not inhibit CYP3A4. Clinical interaction studies with rifampicin (inducer of CYP3A4) and ketoconazole (inhibitor of CYP3A4) showed no clinically relevant change in fulvestrant clearance. Dose adjustment is therefore not necessary in patients who are receiving fulvestrant and CYP3A4 inhibitors or inducers concomitantly.

4.6 Fertility, pregnancy and lactation

Women of childbearing potential
Patients of child-bearing potential should be advised to use effective contraception while on treatment.

Pregnancy
Faslodex is contraindicated in pregnancy (see section 4.3). Fulvestrant has been shown to cross the placenta after single intramuscular doses in rat and rabbit. Studies in animals have shown reproductive

toxicity including an increased incidence of foetal abnormalities and deaths (see section 5.3). If pregnancy occurs while taking Faslodex, the patient must be informed of the potential hazard to the foetus and potential risk for loss of pregnancy.

Breast-feeding
Breast-feeding must be discontinued during treatment with Faslodex. Fulvestrant is excreted in milk in lactating rats. It is not known whether fulvestrant is excreted in human milk. Considering the potential for serious adverse reactions due to fulvestrant in breast-fed infants, use during lactation is contraindicated (see section 4.3).

Fertility
The effects of Faslodex on fertility in humans has not been studied.

4.7 Effects on ability to drive and use machines

Faslodex has no or negligible influence on the ability to drive or use machines. However, since asthenia has been reported very commonly with Faslodex, caution should be observed by those patients who experience this adverse reaction when driving or operating machinery.

4.8 Undesirable effects

This section provides information based on all adverse reactions from clinical trials, post-marketing studies or spontaneous reports. The most frequently reported adverse reactions are injection site reactions, asthenia, nausea, and increased hepatic enzymes (ALT, AST, ALP).

The following frequency categories for adverse drug reactions (ADRs) were calculated based on the Faslodex 500 mg treatment group in pooled safety analyses of the CONFIRM (Study D6997C00002), FINDER 1 (Study D6997C00004), FINDER 2 (Study D6997C00006), and NEWEST (Study D6997C00003) studies that compared Faslodex 500 mg with Faslodex 250 mg. The frequencies in the following table were based on all reported adverse drug reactions, regardless of the investigator assessment of causality.

Adverse reactions listed below are classified according to frequency and System Organ Class (SOC). Frequency groupings are defined according to the following convention: Very common (≥1/10), Common (≥1/100 to <1/10), Uncommon (≥1/1,000 to <1/100). Within each frequency grouping adverse reactions are reported in order of decreasing seriousness.

Table 1 Adverse Drug Reactions

**Adverse reactions by system organ class and frequency**		
Infections and infestations	Common	Urinary tract infections
Blood and lymphatic system disorders	Uncommon	Reduced platelet count
Immune system disorders	Common	Hypersensitivity reactions
Metabolism and nutrition disorders	Common	Anorexia[a]
Nervous system disorders	Common	Headache
Vascular disorders	Common	Venous thromboembolism[a], hot flushes
Gastrointestinal disorders	Very common	Nausea
	Common	Vomiting, diarrhoea
Hepatobiliary disorders	Very common	Increased hepatic enzymes (ALT, AST, ALP)[a]
	Common	Elevated bilirubin[a]
	Uncommon	Hepatic failure[c], hepatitis, elevated gamma-GT
Skin and subcutaneous tissue disorders	Common	Rash
Musculoskeletal and connective tissue	Common	Back pain[a]

disorders		
Reproductive system and breast disorders	Uncommon	Vaginal moniliasis, leukorrhea, vaginal haemorrhage
General disorders and administration site conditions	Very common	Asthenia[a], injection site reactions[b]
	Uncommon	Injection site haemorrhage, injection site haematoma, sciatica, neuralgia[c], neuropathy peripheral

[a] Includes adverse drug reactions for which the exact contribution of Faslodex cannot be assessed due to the underlying disease.

[b] The term injection site reactions does not include the terms injection site haemorrhage, injection site haematoma, sciatica, neuralgia and neuropathy peripheral.

[c] The event was not observed in major clinical studies (CONFIRM, FINDER 1, FINDER 2, NEWEST). The frequency has been calculated using the upper limit of the 95% confidence interval for the point estimate. This is calculated as 3/560 (where 560 is the number of patients in the major clinical studies), which equates to a frequency category of 'uncommon'.

Reporting of suspected adverse reactions
Reporting suspected adverse reactions after authorisation of the medicinal product is important. It allows continued monitoring of the benefit/risk balance of the medicinal product. Healthcare professionals are asked to report any suspected adverse reactions via the national reporting system listed in Appendix V.

4.9 Overdose

There is no human experience of overdose. Animal studies suggest that no effects other than those related directly or indirectly to anti-estrogenic activity were evident with higher doses of fulvestrant (see section 5.3). If overdose occurs, symptomatic supportive treatment is recommended.

5. PHARMACOLOGICAL PROPERTIES

5.1 Pharmacodynamic properties

Pharmacotherapeutic group: Endocrine therapy, Anti-estrogens, ATC code: L02BA03

Mechanism of action and pharmacodynamic effects
Fulvestrant is a competitive estrogen receptor (ER) antagonist with an affinity comparable to estradiol. Fulvestrant blocks the trophic actions of estrogens without any partial agonist (estrogen-like) activity. The mechanism of action is associated with down-regulation of estrogen receptor protein levels. Clinical trials in postmenopausal women with primary breast cancer have shown that fulvestrant significantly down-regulates ER protein in ER positive tumours compared with placebo. There was also a significant decrease in progesterone receptor expression consistent with a lack of intrinsic estrogen agonist effects. It has also been shown that fulvestrant 500 mg downregulates ER and the proliferation marker Ki67, to a greater degree than fulvestrant 250 mg in breast tumours in postmenopausal neoadjuvant setting.

Clinical efficacy and safety in advanced breast cancer
A phase III clinical trial was completed in 736 postmenopausal women with advanced breast cancer who had disease recurrence on or after adjuvant endocrine therapy or progression following endocrine therapy for advanced disease. The study included 423 patients whose disease had recurred or progressed during anti-estrogen therapy (AE subgroup) and 313 patients whose disease had recurred or progressed during aromatase inhibitor therapy (AI subgroup). This trial compared the efficacy and safety of Faslodex 500 mg (n=362) with Faslodex 250 mg (n=374). Progression-free survival (PFS) was the primary endpoint; key secondary efficacy endpoints included objective response rate (ORR), clinical benefit rate (CBR) and overall survival (OS). Efficacy results for the CONFIRM study are summarized in Table 2.

Table 2 Summary of results of the primary efficacy endpoint (PFS) and key secondary efficacy endpoints in the CONFIRM study

Variable	Type of estimate; treatment comparison	Faslodex 500 mg (N=362)	Faslodex 250 mg (N=374)	Comparison between groups (Faslodex 500 mg/Faslodex 250 mg)		
				Hazard ratio	95% CI	p-value
**PFS**	**K-M median in months; hazard ratio**					
**All Patients**		6.5	5.5	0.80	0.68, 0.94	0.006
**-AE subgroup (n=423)**		8.6	5.8	0.76	0.62, 0.94	0.013
**-AI subgroup (n=313)[a]**		5.4	4.1	0.85	0.67, 1.08	0.195
**OS[b]**	**K-M median in months; hazard ratio**					
**All Patients**		26.4	22.3	0.81	0.69, 0.96	0.016[c]
**-AE subgroup (n=423)**		30.6	23.9	0.79	0.63, 0.99	0.038[c]
**-AI subgroup (n=313)[a]**		24.1	20.8	0.86	0.67, 1.11	0.241[c]
**Variable**	**Type of estimate; treatment comparison**	**Faslodex 500 mg (N=362)**	**Faslodex 250 mg (N=374)**	**Comparison between groups (Faslodex 500 mg/Faslodex 250 mg)**		
				**Absolute difference in %**	**95% CI**	
**ORR[d]**	**% of patients with OR; absolute difference in %**					
**All Patients**		13.8	14.6	-0.8	-5.8, 6.3	
**-AE subgroup (n=296)**		18.1	19.1	-1.0	-8.2, 9.3	
**-AI subgroup (n=205)[a]**		7.3	8.3	-1.0	-5.5, 9.8	
**CBR[e]**	**% of patients with CB; absolute difference in %**					
**All Patients**		45.6	39.6	6.0	-1.1, 13.3	
**-AE subgroup (n=423)**		52.4	45.1	7.3	-2.2, 16.6	
**-AI subgroup (n=313)[a]**		36.2	32.3	3.9	-6.1, 15.2	

a Faslodex is indicated in patients whose disease had recurred or progressed on an anti-estrogen therapy. The results in the AI subgroup are inconclusive.
b OS is presented for the final survival analyses at 75% maturity.
c Nominal p-value with no adjustments made for multiplicity between the initial overall survival analyses at 50% maturity and the updated survival analyses at 75% maturity.
d ORR was assessed in patients who were evaluable for response at baseline (i.e. those with measurable disease at baseline: 240 patients in the Faslodex 500 mg group and 261 patients in the Faslodex 250 mg group).
e Patients with a best objective response of complete response, partial response or stable disease ≥24 weeks.

PFS:Progression-free survival; ORR:Objective response rate; OR:Objective response; CBR:Clinical benefit rate; CB:Clinical benefit; OS:Overall survival; K-M:Kaplan-Meier; CI:Confidence interval; AI:Aromatase inhibitor; AE:Anti-estrogen.

Two phase-III clinical trials were completed in a total of 851 postmenopausal women with advanced breast cancer who had disease recurrence on or after adjuvant endocrine therapy or progression following endocrine therapy for advanced disease. 77% of the study population had estrogen receptor positive breast cancer. These trials compared the safety and efficacy of monthly administration of

Faslodex 250 mg versus the daily administration of 1 mg anastrozole (aromatase inhibitor). Overall, Faslodex at the 250 mg monthly dose was at least as effective as anastrozole in terms of progression-free survival, objective response, and time to death. There were no statistically significant differences in any of these endpoints between the two treatment groups. Progression-free survival was the primary endpoint. Combined analysis of both trials showed that 83% of patients who received Faslodex progressed, compared with 85% of patients who received anastrozole. Combined analysis of both trials showed the hazard ratio of Faslodex 250 mg to anastrozole for progression-free survival was 0.95 (95% CI 0.82 to 1.10). The objective response rate for Faslodex 250 mg was 19.2% compared with 16.5% for anastrozole. The median time to death was 27.4 months for patients treated with Faslodex and 27.6 months for patients treated with anastrozole. The hazard ratio of Faslodex 250 mg to anastrozole for time to death was 1.01 (95% CI 0.86 to 1.19).

Effects on the postmenopausal endometrium
Preclinical data do not suggest a stimulatory effect of fulvestrant on the postmenopausal endometrium (see section 5.3). A 2-week study in healthy postmenopausal volunteers treated with 20 μg per day ethinylestradiol showed that pre-treatment with Faslodex 250 mg resulted in significantly reduced stimulation of the postmenopausal endometrium, compared to pre-treatment with placebo, as judged by ultrasound measurement of endometrium thickness.

Neoadjuvant treatment for up to 16 weeks in breast cancer patients treated with either Faslodex 500 mg or Faslodex 250 mg did not result in clinically significant changes in endometrial thickness, indicating a lack of agonist effect. There is no evidence of adverse endometrial effects in the breast cancer patients studied. No data are available regarding endometrial morphology.

In two short-term studies (1 and 12 weeks) in premenopausal patients with benign gynaecologic disease, no significant differences in endometrial thickness were observed by ultrasound measurement between fulvestrant and placebo groups.

Effects on bone
There are no long-term data on the effect of fulvestrant on bone. Neoadjuvant treatment for up to 16 weeks in breast cancer patients with either Faslodex 500 mg or Faslodex 250 mg did not result in clinically significant changes in serum bone-turnover markers.

Paediatric population
Faslodex is not indicated for use in children. The European Medicines Agency has waived the obligation to submit the results of studies with Faslodex in all subsets of the paediatric population in breast cancer (see section 4.2 for information on paediatric use).

An open-label phase II study investigated the safety, efficacy and pharmacokinetics of fulvestrant in 30 girls aged 1 to 8 years with Progressive Precocious Puberty associated with McCune Albright Syndrome (MAS). The paediatric patients received 4 mg/kg monthly intramuscular dose of fulvestrant. This 12-month study investigated a range of MAS endpoints and showed a reduction in the frequency of vaginal bleeding and a reduction in the rate of bone age advancement. The steady-state trough concentrations of fulvestrant in children in this study were consistent with that in adults (see section 5.2). There were no new safety concerns arising from this small study, but 5-year data are yet not available.

5.2 Pharmacokinetic properties

Absorption
After administration of Faslodex long-acting intramuscular injection, fulvestrant is slowly absorbed and maximum plasma concentrations (C_{max}) are reached after about 5 days. Administration of Faslodex 500 mg regimen achieves exposure levels at, or close to, steady state within the first month of dosing (mean [CV]: AUC 475 [33.4%] ng.days/ml, C_{max} 25.1 [35.3%] ng/ml, C_{min} 16.3 [25.9%] ng/ml, respectively). At steady state, fulvestrant plasma concentrations are maintained within a relatively narrow range with up to an approximately 3-fold difference between maximum and

trough concentrations. After intramuscular administration, the exposure is approximately dose-proportional in the dose range 50 to 500 mg.

Distribution
Fulvestrant is subject to extensive and rapid distribution. The large apparent volume of distribution at steady state (Vd_{ss}) of approximately 3 to 5 l/kg suggests that distribution is largely extravascular. Fulvestrant is highly (99%) bound to plasma proteins. Very low density lipoprotein (VLDL), low density lipoprotein (LDL), and high density lipoprotein (HDL) fractions are the major binding components. No interaction studies were conducted on competitive protein binding. The role of sex hormone-binding globulin (SHBG) has not been determined.

Biotransformation
The metabolism of fulvestrant has not been fully evaluated, but involves combinations of a number of possible biotransformation pathways analogous to those of endogenous steroids. Identified metabolites (includes 17-ketone, sulphone, 3-sulphate, 3- and 17-glucuronide metabolites) are either less active or exhibit similar activity to fulvestrant in anti-estrogen models. Studies using human liver preparations and recombinant human enzymes indicate that CYP3A4 is the only P450 isoenzyme involved in the oxidation of fulvestrant; however, non-P450 routes appear to be more predominant *in vivo*. *In vitro* data suggest that fulvestrant does not inhibit CYP450 isoenzymes.

Elimination
Fulvestrant is eliminated mainly in metabolised form. The major route of excretion is via the faeces, with less than 1% being excreted in the urine. Fulvestrant has a high clearance, 11±1.7 ml/min/kg, suggesting a high hepatic extraction ratio. The terminal half-life ($t_{1/2}$) after intramuscular administration is governed by the absorption rate and was estimated to be 50 days.

Special populations
In a population pharmacokinetic analysis of data from phase III studies, no difference in fulvestrant's pharmacokinetic profile was detected with regard to age (range 33 to 89 years), weight (40-127 kg) or race.

Renal impairment
Mild to moderate impairment of renal function did not influence the pharmacokinetics of fulvestrant to any clinically relevant extent.

Hepatic impairment
The pharmacokinetics of fulvestrant has been evaluated in a single-dose clinical trial conducted in subjects with mild to moderate hepatic impairment (Child-Pugh class A and B). A high dose of a shorter duration intramuscular injection formulation was used. There was up to about 2.5-fold increase in AUC in subjects with hepatic impairment compared to healthy subjects. In patients administered Faslodex, an increase in exposure of this magnitude is expected to be well tolerated. Subjects with severe hepatic impairment (Child-Pugh class C) were not evaluated.

Paediatric population
The pharmacokinetics of fulvestrant has been evaluated in a clinical trial conducted in 30 girls with Progressive Precocious Puberty associated with McCune Albright Syndrome (see section 5.1). The paediatric patients were aged 1 to 8 years and received 4 mg/kg monthly intramuscular dose of fulvestrant. The geometric mean (standard deviation) steady state trough concentration (Cmin,ss) and AUCss was 4.2 (0.9) ng/mL and 3680 (1020) ng*hr/mL, respectively. Although the data collected were limited, the steady-state trough concentrations of fulvestrant in children appear to be consistent with those in adults.

5.3 Preclinical safety data

The acute toxicity of fulvestrant is low.

Faslodex and other formulations of fulvestrant were well tolerated in animal species used in multiple dose studies. Local reactions, including myositis and granulomata at the injection site were attributed to the vehicle but the severity of myositis in rabbits increased with fulvestrant, compared to the saline control. In toxicity studies with multiple intramuscular doses of fulvestrant in rats and dogs, the anti-estrogenic activity of fulvestrant was responsible for most of the effects seen, particularly in the female reproductive system, but also in other organs sensitive to hormones in both sexes. Arteritis involving a range of different tissues was seen in some dogs after chronic (12 months) dosing.

In dog studies following oral and intravenous administration, effects on the cardiovascular system (slight elevations of the S-T segment of the ECG [oral], and sinus arrest in one dog [intravenous]) were seen. These occurred at exposure levels higher than in patients (C_{max} >15 times) and are likely to be of limited significance for human safety at the clinical dose.

Fulvestrant showed no genotoxic potential.

Fulvestrant showed effects upon reproduction and embryo/foetal development consistent with its anti-estrogenic activity, at doses similar to the clinical dose. In rats, a reversible reduction in female fertility and embryonic survival, dystocia and an increased incidence of foetal abnormalities including tarsal flexure were observed. Rabbits given fulvestrant failed to maintain pregnancy. Increases in placental weight and post-implantation loss of foetuses were seen. There was an increased incidence of foetal variations in rabbits (backwards displacement of the pelvic girdle and 27 pre-sacral vertebrae).

A two-year oncogenicity study in rats (intramuscular administration of Faslodex) showed increased incidence of ovarian benign granulosa cell tumours in female rats at the high dose, 10 mg/rat/15 days and an increased incidence of testicular Leydig cell tumours in males. In a two-year mouse oncogenicity study (daily oral administration) there was an increased incidence of ovarian sex cord stromal tumours (both benign and malignant) at doses of 150 and 500 mg/kg/day. At the no-effect level for these findings, systemic exposure levels (AUC) were, in rats, approximately 1.5–fold the expected human exposure levels in females and 0.8-fold in males, and in mice, approximately 0.8-fold the expected human exposure levels in both males and females. Induction of such tumours is consistent with pharmacology-related endocrine feedback alterations in gonadotropin levels caused by anti-estrogens in cycling animals. Therefore these findings are not considered to be relevant to the use of fulvestrant in postmenopausal women with advanced breast cancer.

6. PHARMACEUTICAL PARTICULARS

6.1 List of excipients

Ethanol (96 per cent)
Benzyl alcohol
Benzyl benzoate
Castor oil

6.2 Incompatibilities

In the absence of compatibility studies, this medicinal product must not be mixed with other medicinal products.

6.3 Shelf life

4 years

6.4 Special precautions for storage

Store and transport in a refrigerator (2°C - 8°C).

Temperature excursions outside 2°C - 8°C should be limited. This includes avoiding storage at temperatures exceeding 30°C, and not exceeding a 28 day period where the average storage temperature for the product is below 25°C (but above 2°C - 8°C). After temperature excursions, the product should be returned immediately to the recommended storage conditions (store and transport in a refrigerator 2°C - 8°C). Temperature excursions have a cumulative effect on the product quality and the 28 day time period must not be exceeded over the duration of the 4-year shelf life of Faslodex (see section 6.3). Exposure to temperatures below 2°C will not damage the product providing it is not stored below – 20°C.

Store the pre-filled syringe in the original package in order to protect from light.

6.5 Nature and contents of container

BD SafetyGlide is a trademark of Becton Dickinson and Company and is CE-marked: CE 0050.

The pre-filled syringe presentation consists of:

One clear type 1 glass pre-filled syringe with polystyrene plunger rod, fitted with a tamper-evident closure, containing 5 ml Faslodex solution for injection.
A safety needle (BD SafetyGlide™) for connection to the barrel is also provided.
Or
Two clear type 1 glass pre-filled syringes with polystyrene plunger rod, fitted with a tamper-evident closure, each containing 5 ml Faslodex solution for injection. Safety needles (BD SafetyGlide™) for connection to each barrel are also provided.

Not all pack sizes may be marketed.

6.6 Special precautions for disposal and other handling

Instructions for administration

Administer the injection according to the local guidelines for performing large volume intramuscular injections.

NOTE: Due to the proximity of the underlying sciatic nerve, caution should be taken if administering Faslodex at the dorsogluteal injection site (see section 4.4).

Warning - Do not autoclave safety needle (BD SafetyGlide™ Shielding Hypodermic Needle) before use. Hands must remain behind the needle at all times during use and disposal.

For each of the two syringes:

- Remove glass syringe barrel from tray and check that it is not damaged.
- Peel open the safety needle (SafetyGlide™) outer packaging.
- Parenteral solutions must be inspected visually for particulate matter and discolouration prior to administration.
- Hold the syringe upright on the ribbed part (C). With the other hand, take hold of the cap (A) and carefully tilt back and forth until the cap disconnects and can be pulled off, do not twist (see Figure 1).

Figure 1

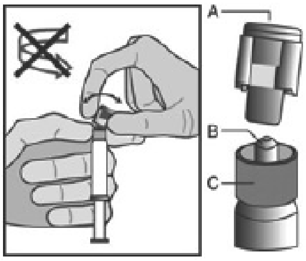

- Remove the cap (A) in a straight upward direction. To maintain sterility do not touch the syringe tip (B) (see Figure 2).

Figure 2

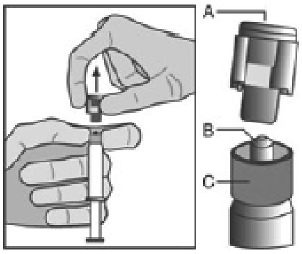

- Attach the safety needle to the Luer-Lok and twist until firmly seated (see Figure 3).
- Check that the needle is locked to the Luer connector before moving out of the vertical plane.
- Pull shield straight off needle to avoid damaging needle point.
- Transport filled syringe to point of administration.
- Remove needle sheath.
- Expel excess gas from the syringe.

Figure 3

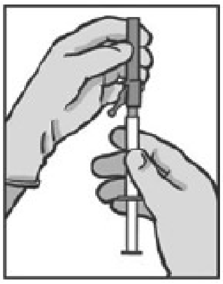

- Administer intramuscularly slowly (1-2 minutes/injection) into the buttock (gluteal area). For user convenience, the needle bevel-up position is oriented to the lever arm (see Figure 4).

Figure 4

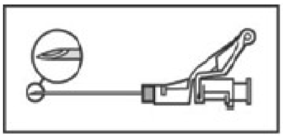

- After injection, immediately apply a single-finger stroke to the activation assisted lever arm to activate the shielding mechanism (see Figure 5).
NOTE: Activate away from self and others. Listen for click and visually confirm needle tip is fully covered.

Figure 5

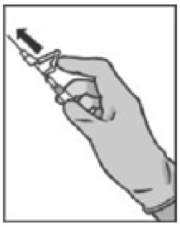

<u>Disposal</u>
Pre-filled syringes are for single use **only**.
Any unused medicinal product or waste material should be disposed of in accordance with local requirements.

7. MARKETING AUTHORISATION HOLDER

AstraZeneca UK Limited
Charter Way,
Macclesfield,
Cheshire
SK10 2NA
United Kingdom

8. MARKETING AUTHORISATION NUMBER(S)

EU/1/03/269/001
EU/1/03/269/002

9. DATE OF FIRST AUTHORISATION/RENEWAL OF THE AUTHORISATION

Date of first authorisation: 10 March 2004

12

Date of latest renewal: 10 March 2009

10. DATE OF REVISION OF THE TEXT

Detailed information on this medicinal product is available on the website of the European Medicines Agency http://www.ema.europa.eu

[redacted]

[redacted]

CT7001_001 MODULE 1B-1 PROTOCOL

PROTOCOL REDACTED TO REMOVE DETAILS OF OTHER MODULES NOT RELEVANT TO THIS MANUSCRIPT

STUDY CT7001_001 VOLUME 3, MODULE 1, PART B-1 (TNBC)

**Title of Core Study CT7001_001:**	A Modular, Multipart, Multiarm, Open-label, Phase I/IIa Study to Evaluate the Safety and Tolerability of CT7001 Alone and in Combination with Anti-cancer Treatments in Patients with Advanced Malignancies
**Title of Volume 3, Module 1 Part B-1 (TNBC):**	Phase 1b Expansion Study of CT7001 as Monotherapy in Patients with Metastatic or Locally Advanced Triple-Negative Breast Cancer.
**Study Number:**	CT7001_001
**EudraCT Number:**	2017-002026-20
**ClinTrials.gov ID:**	NCT03363893
**Study Phase:**	Phase I
**Test Product:**	CT7001 (Samuraciclib)
**Indication in Volume 3, Module 1 Part B-1:**	Treatment of metastatic or locally advanced triple-negative breast cancer in patients who had previously received at least one line of prior chemotherapy for metastatic or locally advanced disease
**Sponsor:**	Carrick Therapeutics<br>NovaUCD,<br>Belfield Innovation Park,<br>University College Dublin,<br>Belfield,<br>Dublin 4, Ireland
**Medical Monitor:**	Dr Nayana Ghodki<br>Bionical Emas<br>63-65 Knowl Piece,<br>Wilbury Way, Hitchin<br>Hertfordshire, SG4 0TY, UK<br>Telephone: +44 (0) 1462 424400<br>E-mail: nayana.ghodki@bionical-emas.com
**Date of Volume 3, Module 1 Part B:**	Version 1.0, 17 September 2018
**Amendment:**	Version 2.0, 26 March 2019<br>*(Version 3.0, 29 November 2019 – Protocol not implemented)*<br>Version 4.0, 30 January 2020<br>Version 5.0, 27 April 2020<br>Version 6.0, 15 July 2020<br>Version 7.0, 23 December 2020

Confidentiality Statement

The information in this document is confidential and will not be disclosed to others without written authorisation from the Sponsor, except to the extent necessary to obtain informed consent from persons receiving the study drug or their legal guardians, or for discussions with Regulatory Authorities, Institutional Review Boards, Ethics Committees, or persons participating in the conduct of the study. Do not copy or distribute without written permission from the Sponsor.

PROTOCOL APPROVAL PAGE

____________________________________<br>Glen Clack<br>Carrick Therapeutics, Senior Medical Director	Date: ________________________
____________________________________<br>Matthew Krebs<br>National Co-ordinator for Overall Modular Study	Date: ________________________
____________________________________<br>Sacha Howell<br>Other Principal Investigator for Module 1 Part B-1	Date: ________________________
____________________________________<br>Kristine Pemberton<br>Carrick Therapeutics, Biostatistician	Date: ________________________

TABLE OF CONTENTS

PROTOCOL APPROVAL PAGE 2
TABLE OF CONTENTS 3
LIST OF TABLES 7
LIST OF FIGURES 7
INVESTIGATOR SIGNATURE PAGE 8
LIST OF ABBREVIATIONS 9
SYNOPSIS OF STUDY CT7001_001, Volume 3, Part B-1 (TNBC) 12
1 INTRODUCTION 21
2 BACKGROUND AND RATIONALE 21
2.1 Triple-Negative Breast Cancer 21
2.2 CDK7 22
2.2.1 CDK7 in TNBC 22
2.3 CT7001 22
2.3.1 Overview of Nonclinical Data 22
2.3.2 Current Clinical Data of CT7001 24
3 OBJECTIVES AND ENDPOINTS 31
3.1 Objectives 31
3.2 Endpoints 31
4 STUDY POPULATION 32
4.1 Inclusion Criteria 32
4.2 Exclusion Criteria 34
5 DESIGN OF MODULE 1B-1 36
6 STUDY TREATMENT 39
6.1 Drug Supply 39
6.1.1 Pharmaceutical Properties 39
6.1.2 Formulation, Packaging and Storage 39
6.2 Dispensing 40
6.3 Dosing Regimen of CT7001 40
6.4 Drug Administration 41
6.5 General Rules 41
6.6 Medication Dosing Errors 41
6.6.1 Overdose 41
6.7 Intra-Subject Dose Modification 42
6.7.1 Types of Dosing Modifications 42
6.8 Inter-Patient Dose Modification 45

6.9 Compliance ... 45
6.10 Drug Storage and Accountability ... 45
6.11 Concomitant Medications ... 46
6.11.1 Prohibited Medications ... 46
6.11.2 Medications Not Recommended ... 47
6.11.3 Permitted and/or Recommended Treatments ... 48
6.12 Contraception ... 50
6.13 Concomitant Radiotherapy or Surgery ... 50
7 STUDY PROCEDURES ... 51
7.1 Screening ... 51
7.1.1 Screen Failure ... 51
7.2 Active Treatment Phase ... 51
7.3 End of Treatment Visit ... 52
7.4 Follow-up Visit ... 52
7.5 Patient Withdrawal ... 52
7.5.1 Active Treatment Phase Discontinuation ... 52
7.5.2 Post-Treatment Study Discontinuation ... 53
7.6 Study Completion ... 54
8 STUDY ASSESSMENTS ... 54
8.1 Safety Assessments ... 54
8.1.1 Laboratory Safety Assessments ... 54
8.1.2 Electrocardiogram (ECG) ... 55
8.1.3 Other Safety Assessments ... 55
8.2 Tumour Assessments ... 56
8.3 Pharmacokinetic Assessments ... 58
8.4 Biomarker Assessments ... 58
9 ADVERSE EVENT REPORTING ... 61
9.1 Reporting Period ... 62
9.2 Definition of Adverse Event ... 63
9.3 Definition of Adverse Event of Special Interest ... 63
9.4 Definition of Serious Adverse Events ... 64
9.4.1 Protocol-Specified Serious Adverse Events ... 65
9.4.2 Potential Cases of Drug-Induced Liver Injury (Hy's Law Cases) ... 65
9.5 Hospitalization ... 66
9.6 Severity Assessment ... 67
9.7 Causality Assessment ... 67
9.8 Exposure During Pregnancy ... 68

9.9 Occupational Exposure 68
9.10 Withdrawal Due to Adverse Events (See Also Section on Patient Withdrawal) 69
9.11 Eliciting Adverse Event Information 69
9.12 Reporting Requirements 69
9.12.1 Serious Adverse Event Reporting Requirements 69
9.12.2 Non-Serious Adverse Event Reporting Requirements 70
9.12.3 Sponsor’s Reporting Requirements to Regulatory Authorities 70
10 DATA ANALYSIS AND STATISTICAL METHODS 71
10.1 Analysis Populations 71
10.1.1 Safety Analysis Population (SA) 71
10.1.2 Recommended Phase 2 Dose Population (RP2D) 71
10.1.3 Objective Response Population (OR) 71
10.1.4 Intent-to-Treat Population (ITT) 71
10.2 Safety Analysis 71
10.2.1 Adverse Events 71
10.2.2 Laboratory Abnormalities 72
10.2.3 Electrocardiogram (ECG) Analysis 72
10.3 Efficacy Analysis 72
10.3.1 Objective Response (OR) 72
10.3.2 Duration of Response (DOR) 72
10.3.3 Disease Control (DC) 72
10.3.4 Waterfall Plot Analysis 73
10.3.5 Progression-Free Survival (PFS) 73
10.3.6 Sample Size and Interim Analysis 73
10.4 Pharmacokinetic Analysis 74
10.5 Biomarker Analysis 74
10.6 Analysis of Other Endpoints 74
11 ETHICS 75
11.1 Institutional Review Board (IRB)/Independent Ethics Committee (IEC) 75
11.2 Ethical Conduct of the Study 76
11.3 Patient Information and Confidentiality 76
11.4 Patient Consent 76
11.5 Potential Risks and Benefits 76
11.5.1 Potential Risks 76
11.5.2 Potential Benefits 78
11.5.3 Overall Risk/Benefit Assessment 78
11.6 Patient Recruitment 78

11.7 Reporting of Safety Issues and Serious Breaches of the Protocol or ICH GCP 79
12 QUALITY CONTROL AND QUALITY ASSURANCE 79
13 DATA HANDLING AND RECORD KEEPING 79
13.1 Case Report Forms/Electronic Data Record 79
13.2 Record Retention 80
14 DEFINITION OF END OF TRIAL 80
14.1 End of Trial in all Participating Countries 80
14.2 End of Trial in a Member State of the European Union 80
15 SPONSOR DISCONTINUATION CRITERIA 80
16 ADMINISTRATIVE PROCEDURES AND CONSIDERATIONS 81
16.1 Safety Review Committee 81
16.2 Protocol Amendments 81
16.3 Clinical Study Report 81
16.4 Financing and Insurance 81
17 PUBLICATION OF STUDY RESULTS 81
17.1 Communication of Results by Carrick 82
17.2 Publications by Investigators 82
18 SCHEDULE OF ASSESSMENTS 84
19 REFERENCES 89
APPENDICES TO VOLUME 3, MODULE 1, PART B-1 (TNBC) 93
APPENDIX A – DRUGS KNOWN TO PREDISPOSE TO TORSADE DE POINTES 93
APPENDIX B - MEDICATIONS, HERBAL SUPPLEMENTS, AND FOODS THAT SIGNIFICANTLY INDUCE OR INHIBIT CYTOCHROME P450 3A4, 2C19 AND/OR 2D6 OR P GLYCOPROTEIN ACTIVITY 95
APPENDIX C - RESPONSE EVALUATION CRITERIA IN SOLID TUMOURS (RECIST) VERSION 1.1 98
APPENDIX D - NEW YORK HEART ASSOCIATION (NYHA) FUNCTIONAL CLASSIFICATION 102
APPENDIX E - ACTIONS REQUIRED IN CASES OF COMBINED INCREASE OF AMINOTRANSFERASE AND TOTAL BILIRUBIN – HY'S LAW 103
APPENDIX F – EASTERN COOPERATIVE ONCOLOGY GROUP (ECOG) PERFORMANCE STATUS 106
APPENDIX G – MANAGEMENT OF THE CT7001_001 STUDY THROUGHOUT COVID-19 PANDEMIC 107
APPENDIX H – CT7001_001 DATA MONITORING COMMITTEE NVD GUIDELINES VERSION 1.0 26-OCT-2020 110

LIST OF TABLES

Table 1: Status of Module 1A, 1B, 2 and 4 as of 16-Nov-2020 25
Table 2: CT7001 Excipients 40
Table 3: Two Dose Reduction Levels for Three Potential Starting Doses 44
Table 4: Number and Strength of Capsules for Each Possible Dose 44
Table 5: Schedule of Events – Mandatory Procedures 84
Table 6: Schedule of Events – Optional Procedures 86
Table 7: Blood Volumes Required for Mandatory Procedures 87
Table 8: Blood Volumes Required for Optional Procedures 88

LIST OF FIGURES

Figure 1: CT7001 decreases pPolII levels in PBMC 29

INVESTIGATOR SIGNATURE PAGE

CT7001_001 Volume 3, Module 1 Part B-1 (TNBC: Phase 1 Expansion Study of CT7001 as Monotherapy in Patients with Metastatic or Locally Advanced Triple-Negative Breast Cancer)

I have read the Core Study Protocol (Volume 1) in association with Volume 3 (the protocol for Module 1B-1 (TNBC)) and agree to conduct the trial in compliance with the International Council for Harmonisation Guideline for Good Clinical Practice and applicable regulatory requirements and to inform all who assist me in the conduct of this trial of their responsibilities and obligations.

Signed: ______________________________ Date: __________________________

Print Name: _________________________

LIST OF ABBREVIATIONS

Abbreviation	Definition
AE	adverse event
AESI	adverse event of special interest
ALP	alkaline phosphatase
ALT	alanine aminotransferase
AST	aspartate aminotransferase
ANC	absolute neutrophil count
AR	androgen receptor
ASCO	American Society of Clinical Oncology
BID	twice a day
CAP	College of American Pathologists
CDK	cyclin-dependent kinase
CNS	central nervous system
CR	complete response
CRO	Contract Research Organisation
CSR	Clinical Study Report
CT	computed tomography
CTC	circulating tumour cells
CTCAE	Common Terminology Criteria for Adverse Events
ctDNA	circulating tumour deoxyribonucleic acid
CYP	cytochrome P450
DC	disease control
DLT	dose-limiting toxicity
DNA	deoxyribonucleic acid
DOR	duration of response
D/N/V	Diarrhoea and/or Nausea and/or Vomiting
ECG	electrocardiogram
ECOG	Eastern Cooperative Oncology Group
eCRF	electronic case report form
ER	oestrogen receptor

Abbreviation	Definition
ESMO	European Society of Medical Oncology
FSH	follicle stimulating hormone
GCP	Good Clinical Practice
G-CSF	granulocyte colony-stimulating factor
HbA1c	Glycated haemoglobin
HER2	human epidermal growth factor receptor 2
hERG	human ether-a-go-go-related gene
HL	Hy’s Law
IB	Investigator’s Brochure
IC_{50}	half maximal inhibitory concentration
ICF	informed consent form
ICH	International Council for Harmonisation
IEC	Independent Ethics Committee
IHC	immunohistochemistry
IMP	Investigational Medicinal Product
INR	international normalised ratio
IRB	Institutional Review Board
ITT	Intent-to-Treat
IV	intravenous
IXRS	Interactive response system
LFT	liver function test
MBAD	minimum biologically active dose
MED1	Mediator of RNA polymerase II transcription subunit 1
MedDRA	Medical Dictionary for Regulatory Activities
MRI	magnetic resonance imaging
NCI	National Cancer Institute
NCCN	National Comprehensive Cancer Network
OR	objective response
ORR	objective response rate
PBMC	peripheral blood mononuclear cell
PDc	pharmacodynamic

**Abbreviation**	**Definition**
PD	progression of disease
PD-L1	programmed death ligand 1
PFS	progression-free survival
PGP	p-glycoprotein
PgR	progesterone receptor
PHL	Potential Hy's Law
PK	pharmacokinetic
pPolII	phosphorylated RNA polymerase II
PR	partial response
QTcF	QT interval corrected for heart rate by the Fridericia formula
RECIST	Response Evaluation Criteria in Solid Tumours
RNA	ribonucleic acid
RP2D	Recommended Phase 2 Dose
SAE	serious adverse event
SD	stable disease
SRC	Safety Review Committee
TBL	total bilirubin
TNBC	triple-negative breast cancer
ULN	upper limit of normal

SYNOPSIS OF STUDY CT7001_001, VOLUME 3, PART B-1 (TNBC)

**Sponsor**: Carrick Therapeutics	
**Study Title**: A Phase 1b Expansion Study of CT7001 as Monotherapy in Patients with Metastatic or Locally Advanced Triple-Negative Breast Cancer	
**Study Number:** CT7001_001	**Study Phase:** Phase Ib/IIa
**EudraCT Number:** 2017-002026-20	**ClinTrials.Gov ID:** NCT03363893
**Volume 3, Module 1, Part B-1 (TNBC) Study Objectives**	
**Primary Objectives:**<br>• To further characterise the safety and tolerability of CT7001 and determine the most appropriate dosing regimen for subsequent Phase 2 testing (definitive recommended Phase 2 dose).<br>**Secondary Objectives:**<br>• To evaluate the activity of CT7001 as monotherapy in patients with metastatic or locally advanced triple-negative breast cancer (TNBC).<br>• To further evaluate CT7001 plasma concentrations.<br>• To evaluate CYP2D6 polymorphisms in this patient population.<br>**Exploratory Objectives:**<br>• To further investigate the presence and identity of metabolites of CT7001 and, if appropriate, characterize their pharmacokinetics (PK).<br>• To further explore the relationship between PK and safety, anti-tumour activity and biological activity and the impact of patient characteristics on PK.<br>• To further explore the effects of CT7001 on PDc gene expression (e.g., c-Myc, MCL-1).<br>• To further explore mutations and expression in genes, proteins and ribonucleic acid (RNAs) relevant to the cell cycle (e.g., phosphorylation of CDK1 and Rb proteins), drug target engagement (e.g., c-Myc, MCL-1) and tumour sensitivity and/or resistance in tumour-derived materials including tumour tissue, circulating tumour DNA and circulating tumour cells (e.g., p53, CDK7, ER, androgen receptor (AR)).	
**Module 1, Part B-1 Study Centres:** Centres will be based in the United Kingdom and the USA.	
**Number of Patients Planned:** 50	
**Eligibility Criteria:**<br>To be eligible for the study patients have to meet **all** of the following criteria:<br>1. Patients at least 18 years of age.	

2. Histologically confirmed carcinoma of the breast not expressing oestrogen receptor (ER) and progesterone receptor (PgR) and negative for human epidermal growth factor receptor 2 (HER2).

    - Assessment of ER, PgR and HER2 in breast carcinoma tissue will be based on results from local pathology laboratories. Independent central review is not intended.
    - Negative assessment for ER, PgR and HER2 by local laboratories should be consistent with the criteria described in the most recent versions of the American Society of Clinical Oncology (ASCO)/College of American Pathologists (CAP) guidelines for testing of ER, PgR and HER2, respectively (Hammond et al, 2010; Wolff et al, 2018):

        - ER- and PgR-negativity is determined as <1% of tumour cells positive by immunohistochemistry (IHC) utilizing an IHC assay consistent with local standards.

        - HER2-negativity is determined as immunohistochemistry score 0/1+ or negative by in situ hybridization (FISH/CISH/SISH) defined as a HER2/CEP17 ratio <2 or for single probe assessment a HER2 copy number <4.

        - Determination of negative ER, PgR and HER2 status should be based on data from the most recent tumour biopsy. In case no tumour biopsy was performed after initial resection of the primary tumour, the original tumour tissue serves as basis for assessment of ER/PgR/HER2 status.

3. Metastatic or locally advanced disease not amenable to resection or radiation therapy with curative intent with documented progressive disease on or within 6 months of most recent prior chemotherapy.

4. Disease must be measurable by Response Evaluation Criteria in Solid Tumours (RECIST, version 1.1), Appendix C.

5. Patients must have received at least one cytotoxic chemotherapy regimen for metastatic/locally advanced disease.

6. Eastern Cooperative Oncology Group (ECOG) performance status 0 or 1 with no deterioration over the previous 2 weeks (Appendix F).

7. Expected life expectancy of greater than 12 weeks.

8. Ability to swallow and retain oral medication.

9. No childbearing potential, defined as women:

    - Who had prior hysterectomy or bilateral surgical oophorectomy or are medically postmenopausal (defined as spontaneous cessation of regular menses for at least 12 consecutive months or follicle stimulating hormone

(FSH) and oestradiol blood levels in the testing laboratory's respective postmenopausal range with no alternative pathological or physiological cause).

10. Women of childbearing potential must be willing to practice effective contraception (defined as abstinence i.e., refraining from heterosexual intercourse during the entire period of risk associated with the study treatment, sex only with person of the same sex, sex only with vasectomized partner, intrauterine device, or double-barrier methods) for the duration of the study and for 6 months after the last dose of CT7001. Single barrier methods (e.g., condom or diaphragm alone) are not considered effective contraception methods.

11. Women of childbearing potential must have a negative serum pregnancy test at baseline (within 7 days prior to first dose of CT7001).

12. Sexually active male subjects must be willing to use condoms with all sexual partners for the duration of the study and for 3 months after the last dose of CT7001. If a female partner is a woman of childbearing potential who is not using effective contraception (as defined above), the subject must use a condom with spermicide during the study and for 6 months after the last dose of CT7001.

13. Patients are willing and able to comply with scheduled visits, treatment plan, laboratory tests, and other study procedures.

14. Personally signed and dated written informed consent indicating that the patient has been informed of all pertinent aspects of the study before any study-specific activity is performed.

Host Genetics Research Study: Pharmacogenomics Samples (Optional)
Patients who meet all of the following criteria may be included in optional genetics substudies:

1. Provision of signed and dated, written informed consent for the genetic research.

To be eligible for the study patients **may not have any** of the following exclusion criteria:

1. More than three lines of cytotoxic chemotherapy for metastatic and/or locally advanced disease.

2. Advanced, symptomatic, visceral metastases if risk of life-threatening complications in the short term (including patients with massive uncontrolled effusions [pleural, pericardial, peritoneal], pulmonary lymphangitis and over 50% liver involvement).

3. Known symptomatic central nervous system (CNS) metastases, carcinomatous meningitis or leptomeningeal disease. Patients with a history of CNS metastases or spinal cord compression due to metastasis are eligible if they have been treated with local therapy (e.g., radiotherapy, stereotactic surgery) and are clinically stable off anticonvulsants and steroids for at least 4 weeks before first dose of CT7001.

4. Inadequate hepatic, renal, bone marrow or cardiac function, specified as follows:
   a) Hepatic:
      i. Aspartate aminotransferase (AST) and alanine aminotransferase (ALT) > 2.5 × the upper limit of normal (ULN) or > 5.0 × ULN for patients with liver metastases.
      ii. Total bilirubin > 1.5 × ULN (>3.0 x ULN if known Gilbert's disease).
      iii. Albumin < 30 g/L.
      iv. Liver function deteriorating in a manner that would likely make the subject not meeting the AST, ALT, bilirubin or albumin levels specified above at the time of the first dose of CT7001.
      v. Other evidence of impaired hepatic synthesis function.
   b) Renal:
      i. Serum creatinine > 1.5 × ULN.
   c) Bone marrow:
      i. Absolute neutrophil count ≤ 1.5 × 10^9/L.
      ii. Platelet count <100 × 10^9/L.
      iii. Haemoglobin < 90 g/L.
   d) Cardiac:
      i. Myocardial infarction within 6 months of study entry, unstable angina, unstable arrhythmia.
      ii. New York Heart Association Class > I heart failure (see Appendix D).
      iii. Mean resting QT interval corrected for heart rate by the Fridericia formula (QTcF) > 470 msec obtained from 3 ECGs obtained within 3-5 minutes apart.
      iv. Clinically important abnormalities in rhythm, conduction, or morphology on resting ECG (e.g., complete left bundle branch block, third degree heart block).
         - Controlled atrial fibrillation is permitted.
      v. Any factor that may increase the risk of QTc prolongation or of arrhythmic events (e.g., hypokalaemia, congenital long QT syndrome, immediate family history of long QT syndrome or unexplained sudden death under 40 years of age).

5. Unresolved toxicity (except alopecia) from prior therapy of ≥ Grade 2 according to CTCAE version 5.0.
6. Refractory nausea and vomiting, chronic gastro-intestinal disease or previous significant bowel resection, with clinically significant sequelae that would preclude adequate absorption of CT7001.
7. Uncontrolled seizures.
8. Active infection requiring systemic antibiotic, antifungal, or antiviral medication within 14 days prior to first dose of CT7001.
9. Severe or uncontrolled medical condition (e.g., severe chronic obstructive pulmonary disease, severe Parkinson’s disease, active inflammatory bowel disease or psychiatric condition).
10. Active bleeding diatheses.
11. History of haemolytic anaemia or marrow aplasia.
12. Renal or other organ transplant.
13. Known hepatitis B, hepatitis C, or human immunodeficiency virus infection.
14. Pregnancy.
15. Breastfeeding.
16. Previous exposure to CT7001
17. Receipt of systemic corticosteroids (at a dose > 10 mg prednisone/day or equivalent) within 14 days before the first dose of IMP.
18. Non-biological anti-cancer medicines within 28 days or ≤ 5 half-lives, whichever is shorter, before the first dose of CT7001.
19. Biological anti-cancer medicines, including IMP (e.g., monoclonal antibodies, antibody-drug-conjugates) within 42 days before the first dose of CT7001.
20. Receipt of St John’s Wort within 21 days before the first dose of CT7001.
21. Concomitant medication, herbal supplement or food that is a strong inhibitor or inducer of CYP2D6, CYP3A4, CYP2C19, or P-glycoprotein activity within 14 days before the first dose of CT7001 (specific examples listed in protocol Appendix B).
22. Receipt of a blood transfusion (blood or blood products) within 14 days before the first dose of CT7001.
23. Known hypersensitivity to CT7001 or any excipient of the product.
24. Diagnosis of any other malignancy within 3 years prior to enrolment, except for adequately treated basal cell or squamous cell skin cancer, or carcinoma in situ of the cervix.

25. In the opinion of the Investigator, unlikely to comply with study procedures.
26. Patients who are investigational site staff members directly involved in the conduct of the trial and their family members, site staff members otherwise supervised by the Investigator, or patients who are Carrick employees directly involved in the conduct of the trial.
27. Has received a live-virus vaccination within 28 days or less of planned treatment start. Note: seasonal flu vaccines that do not contain live virus are permitted.

Pharmacogenomics Samples (Optional)
Patients who meet any of the following criteria will be excluded from optional genetic substudies:

1. Allogenic bone marrow transplant.
2. Non-leucocyte-depleted whole blood transfusion within 120 days of the date of the genetic sample collection.

Test Product: CT7001 (Samuraciclib), oral

Primary Endpoint

- Type, incidence, severity (as graded by CTCAE v5.0), seriousness and relationship to study medications of adverse events (AE) and any laboratory abnormalities.

Secondary Endpoints

- Objective response rate (ORR)
- Duration of response (DOR)
- Disease control (DC: CR or partial response (PR) or stable disease (SD) ≥24 weeks)
- Best percent tumour size change
- Progression-free survival (PFS)
- Trough plasma concentrations of CT7001

Exploratory Endpoints

- Metabolites of CT7001
- Biomarkers in peripheral blood mononuclear cells (PBMC), circulating tumour DNA, circulating tumour cells and tumour tissue, including genes, RNA expression and proteins (e.g., c-Myc, MCL-1, phosphorylated CDK1 and Rb proteins, p53, CDK7, ER, AR)

Analysis Populations

Safety Analysis Population (SA)
The SA population will include all patients who receive at least 1 dose of study treatment. The SA population will be the primary population for evaluation of safety. PK and efficacy endpoints will be also assessed in this population.

Recommended Phase 2 Dose Population (RP2D)
The RP2D population will include all patients who receive at least 1 dose of study treatment at the dosing regimen to be defined as definitive RP2D. This will be the primary population for evaluating efficacy endpoints.

Objective Response Population (OR)
The OR population will include all patients who had their first scheduled post-baseline tumour assessment (approximately 8 weeks from start of study therapy) or objective disease progression before that. Efficacy endpoints will be assessed in this population.

Intent-to-Treat Population (ITT)
The ITT population will include all enrolled patients with designated study drug assignment. The ITT population will be the primary population for describing patient characteristics.

Safety Analyses

Adverse events will be summarized by the frequency of patients experiencing treatment emergent adverse events corresponding to body systems and Medical Dictionary for Regulatory Activities (MedDRA) preferred term. Adverse events will be graded by worst NCI CTCAEv5.0 Grade. Adverse events will be summarized by cycle and by relatedness to study treatment. Detailed information collected for each AE will include a description of the event, duration, whether the AE was serious, intensity, relationship to study drug, action taken, and clinical outcome. Emphasis in the analysis will be placed on AEs classified as treatment emergent.

Adverse events leading to death or discontinuation of trial treatment, events classified as NCI CTCAE v5.0 Grade 3 or higher, trial drug-related events, and serious adverse events will be considered with special attention.

Haematology and chemistry laboratory data will be summarized by cycle. The laboratory results will be graded according to the NCI CTCAEv5.0 severity grade. The frequencies of the worst severity grade observed will be displayed. For parameters for which an NCI CTCAEv5.0 scale does not exist, the frequency of patients with values below, within, and above the normal ranges will be summarized by treatment.

All electrocardiogram (ECGs) obtained during the study will be evaluated for safety. The triplicate data will be averaged and all summary statistics and data presentations will use the triplicate averaged data. Any data obtained from ECGs repeated for safety reasons after the nominal time-points will not be averaged along with the preceding triplicates. For all patients in the SA population, individual change in QTcF will be calculated for each nominal post-baseline time point. These individual changes will be summarized using descriptive statistics.

Efficacy Analysis

The RP2D population will be the primary population for all efficacy analyses. Efficacy endpoints will be assessed as well in the OR and ITT populations. All efficacy endpoints based on radiological (and photographical where applicable) assessments of tumour burden (i.e., OR, DOR, DC, PFS) will be derived using the local radiologist's/investigator's assessment.

Objective response (OR) is defined as a complete response (CR) or partial response (PR) according to the Response Evaluation Criteria in Solid Tumours (RECIST version 1.1; Appendix C).

A patient will be considered to have achieved an OR if the patient has a complete response (CR) or partial response (PR) according to RECIST v.1.1 definitions. Otherwise, the patient will be considered as non-responders in the OR rate analysis. Additionally, patients with inadequate data for tumour assessment (e.g., no baseline or post-baseline assessment) will be considered as non-responders in the OR rate analysis.

The OR rate (ORR) will be estimated by dividing the number of patients with objective response (CR or PR) by the number of patients in a respective analysis population. An exact 95% CI for the response rates will be computed.

Duration of response (DOR) is defined as the time from the first documentation of objective tumour response (CR or PR) to the first documentation of objective tumour progression or to death due to any cause, whichever occurs first. DOR data will be censored on the date of the last tumour assessment on study for patients who do not have objective tumour progression and who do not die due to any cause while on study. DOR will only be calculated for the subgroup of patients with an objective response. The median DOR time estimated from a Kaplan-Meier curve and 95% CI for the median will be computed.

Disease control (DC) is defined as complete response (CR), partial response (PR), or stable disease (SD) ≥24 weeks according to the RECIST version 1.1 (Appendix C) recorded in the time period between enrolment and disease progression or death to any cause. The DC rate (DCR) will be estimated by dividing the number of patients with CR, PR, or SD ≥24 weeks by the number of patients in a particular analysis population. A 95% CI for the DC rate will be computed.

Waterfall Plot Analysis: The best percent change versus baseline in post-baseline aggregate tumour size measurements will be displayed graphically in form of Waterfall plots for the OR population.

Progression-Free Survival (PFS) is defined as the time from the date of enrolment to the date of the first documentation of objective progression of disease (PD) or death due to any cause in the absence of documented PD, whichever occurs first. PFS is difficult to interpret without prospective control and thus specified as an endpoint in Module 1B-1. However, PFS data will be collected and the median PFS time estimated from a Kaplan-Meier curve and 95% CI for the median will be computed.

PFS data will be censored on the date of the last tumour assessment on study for patients who do not have objective tumour progression and who do not die while on study. Patients lacking an assessment of tumour response after enrolment will have their PFS time censored on the date of enrolment with a duration of 1 day. Additionally, patients who start a new anti-cancer therapy prior to documented PD will be censored at the date of the last tumour assessment prior to the start of the new therapy.

Sample Size and Interim Analysis

The planned sample size of the RP2D population is 30 patients. This is considered of sufficient precision in this first estimation of efficacy to properly inform whether and how best to continue the further development of CT7001 as monotherapy in TNBC.

Pharmacokinetic Analysis

Average trough concentrations will be listed by patient. Summary statistics will be provided for trough concentrations by study cycle and for average trough concentrations by patient.

The relationship between trough concentration and potential covariates will be evaluated. All patients treated with CT7001 and for whom drug plasma concentration results (from at least 1 visit) are available will be included in the analysis.

In addition, the relationship between exposure and safety and efficacy endpoints may be explored, based on emerging safety and efficacy data. The results of these modelling analyses may be reported separately from the clinical study report.

Biomarker Analysis
Appropriate statistical methods will be used to investigate any possible relationship of biomarker levels and/or alterations with the recorded efficacy of CT7001.

Analysis of Other Endpoints
Descriptive statistics will be used to summarize all patient characteristics, treatment administration/compliance, safety parameters, and biomarkers. Data will also be displayed graphically, where appropriate.

1 INTRODUCTION

CT7001 (Samuraciclib) is a small molecule, adenosine triphosphate (ATP) competitive, selective oral inhibitor of cyclin-dependent kinase 7 (CDK7). A first-in-human modular Phase 1/2 clinical study was initiated in November 2017 (EudraCT number: 2017-002026-20; ClinicalTrials.gov identifier: NCT03363893). The single- and multiple-ascending dose part of the Phase 1 study (Module 1A) has now completed and has determined 360 mg as a safe and biologically active dose for further testing in Module 1B-1. The Module 1A paired biopsy expansion cohorts in breast cancer is now completed. The planned additional cohort in solid tumour will not initiate. A study of the effect of food on the bioavailability of CT7001 (called Module 4) has completed recruitment and PK data from the randomised, controlled fed-fast period is available. Module 1B-1 (TNBC) and 1B-2 (CRPC) have now completed recruitment and have patients in long term follow up. Module 2 is currently open to recruitment. [redacted] Module 1B-1 represents and additional expansion cohort to part B of the Phase 1 trial and is an open-label, uncontrolled Phase 1 expansion study of CT7001 as monotherapy in patients with metastatic or locally advanced triple-negative breast cancer (TNBC) and documented disease progression who had previously received at least one line of chemotherapy for metastatic disease. Section 2.3.2 will review the status of and data from all modules as per 16-Nov-2020.

2 BACKGROUND AND RATIONALE

The biological and clinical data of CDK7 in TNBC, the nonclinical efficacy of CT7001 in TNBC models, the high unmet medical need of advanced TNBC and the early clinical safety, PK and pharmacodynamic (PDc) data of CT7001 from Module 1 Part A provide a sound rationale for initiating Module 1B-1, a Phase 1 expansion study to investigate CT7001 as monotherapy in women with metastatic or locally advanced TNBC.

2.1 Triple-Negative Breast Cancer

TNBC is an aggressive disease with poor prognosis (Bajaj et al, 2017; Chen et al, 2017; Sharma et al, 2016). Median survival in metastatic TNBC has usually been no more than 12 to 18 months, in patients who had previously received a taxane, an anthracycline and at least one line of chemotherapy for metastatic disease typically <12 months (Bajaj et al, 2017; Baselga et al, 2013; Carey et al, 2012; Chen et al, 2017; Cortes et al, 2011; Dieras et al, 2015; Isakoff et al, 2015; Martin et al, 2018; O'Shaughnessy et al, 2014; Yardley et al, 2018). The standard of care landscape of the treatment of early and first line advanced TNBC is rapidly evolving; although taxanes and anthracyclines may still be used, there are other recognised options for treating oncologists that are now beginning to open (Mehanna et al, 2019; Cardoso et al, 2019). TNBC is now recognised to include various molecular subtypes with different biological behaviour (Bareche et al, 2018; Metzger-Filho et al, 2012; Pareja et al, 2016). With the exception of poly ADP ribose polymerase (PARP) inhibitors for BRCA-mutant TNBC, representing approx. 10% of TNBC, these insights have not yet translated into differential treatment in routine clinical practice (Bajaj et al, 2017; Gadi et al, 2017; Sharma et al, 2016), targeted therapies of proven benefit or drugs which have received regulatory approval specifically for TNBC.

Recently, immunotherapy with programmed death receptor 1 (PD-1)/programmed death ligand 1 (PD-L1) checkpoint inhibitors has shown promise, particularly in the subset of TNBC with expression of PD-L1 (Adams et al, 2017(i), Adams et al, 2017(ii); Dua et al, 2017; Horning et al, 2018). However, chemotherapy continues to serve as standard of care in routine practice, little outcome improvement has been achieved for decades (Gadi et al, 2017; Litton et al, 2018; Sharma et al, 2016; Zeichner et al, 2016) and a significant unmet need remains for new therapies with novel mechanisms of action.

2.2 CDK7

CDK7 has three critical roles in cancer. These are enhanced transcriptional initiation of multiple oncogenes such as c-Myc and upregulation of anti-apoptotic genes such as MCL-1 via phosphorylation of the c-terminal domain of RNA Polymerase II (Chipumuro et al, 2014; Feaver et al, 1994; Fisher et al, 2005; Glover-Cutter et al, 2009; Kwiatkowski et al, 2014), rapid progression through the cell cycle via phosphorylation of other members of the CDK family (such as CDK2, 4 and 6) (Fisher et al, 2005; Fisher and Morgan, 1994; Schachter and Fisher, 2013; Schachter et al, 2013), and loss of sensitivity to hormonal therapy via phosphorylation of ERα (Chen et al, 2000) and the transcriptional coactivator MED1 (Rasool et al, 2019). Please refer to the Investigator's Brochure (IB) for a comprehensive review.

2.2.1 CDK7 in TNBC

CDK7 seems important in TNBC biology (Wang et al, 2015) and high expression of CDK7 has been associated with poor prognosis in patients with TNBC (Li et al, 2017). Thus, targeting CDK7 may be a useful therapeutic strategy for TNBC.

2.3 CT7001

CT7001 (previously also known as ICEC0942) is a small molecule, ATP competitive, selective oral inhibitor of CDK7 which potently inhibits all key biological effects of CDK7 in cancer (Patel et al, 2018).

2.3.1 Overview of Nonclinical Data

The IB provides a comprehensive review and description of all relevant nonclinical study data of CT7001.

Pharmacokinetic studies showed good oral bioavailability in three non-human species (mouse, rat and dog), which predicted good bioavailability in humans. Plasma clearance was high in rats and dogs with a high volume of distribution, resulting in an apparent elimination half-life ($T_{½}$) of 4.8 hours in rats and 9.5 hours in dogs.

Cell growth inhibition studies showed broad activity of CT7001 against a wide range of tumour cell lines, including TNBC (Patel et al, 2018 Ainscow et al, 2018; Clark et al, 2017, Ali et al, 2018). Encouraging activity was also observed in vivo, including in patient-derived xenograft models of TNBC (Bahl et al, 2017). Of note, in sensitive in vivo models CT7001 has shown a cytotoxic phenotype with tumour size reduction.

Western blot analysis showed that CT7001 inhibits the phosphorylation of RNA Pol II, CDK1 and 2 *in vitro* and *in vivo* and this in tumour and in normal cells. This suggested that phosphorylated RNA polymerase II (pPolII) or phosphorylation status of other CDK family members could be a useful pharmacodynamics (PDc) biomarker in early clinical development.

CT7001 was metabolically stable in human microsomes and hepatocytes *in vitro*. Intrinsic clearance of CT7001 was high in rat microsomes, intermediate in mouse and dog microsomes, and low in human liver microsomes. Metabolite profiling following incubation of CT7001 with mouse and human hepatocytes showed that the overall turnover of CT7001 was low, indicating the compound has high stability. No evidence of Phase II metabolism (e.g., glucuronidation) was observed.

In a cytochrome P450 (CYP) study using human microsomes, CT7001 strongly inhibited CYP3A4 and showed weak signals for 2D6 and 2C19, with mean half maximal inhibitory concentration (IC_{50}) values of 5.7, 35.5, and 44.9 µM, respectively. Mean IC_{50} values for CYP1A2, 2C9, 2B6, 2C8 and 3A5 were greater than 50 µM. In a separate assay, the mean IC_{50} of CT7001 for CYP3A4 was 2.9 and 1.8 µM when using testosterone or midazolam as substrates, respectively.

Based on the *in vitro* data, CT7001 is unlikely to cause clinically significant hepatic drug interactions through inhibition of CYP1A2, 2C9, 2B6, 2C8 and 3A5. However, there is a potential for inhibition of intestinal and hepatic CYP3A4 which may be clinically significant for CYP3A4 substrates. CT7001 has shown weak potential to inhibit CYP2D6 and 2C19 which is unlikely to be clinically significant but should be considered when co-medicating with 2D6 and 2C19 substrates.

A phenotyping study investigating the metabolism of CT7001 by eight cytochrome P450 enzymes found that CYP mediated clearance of CT7001 mainly occurs via CYP2D6, followed by 3A4 and a small contribution by 2C19. This suggests that co-medications that modulate the activity of CYP 2D6, 3A4 and 2C19 may affect the exposure of CT7001.

The potential of CT7001 to inhibit transporter proteins has not been studied yet. Plasma protein binding is high (>~95%), with a similar unbound fraction in all 3 species tested (rat, dog and human).

Daily administration of 100 mg/kg/day CT7001 to Han Wistar rats for 7 days was well tolerated. Microscopic findings seen in the GI tract and testes suggest an effect of CT7001 on rapidly dividing cells, likely a result of the pharmacological action of the test article.

The daily administration of 15 or 60 mg/kg/day CT7001 to rats and 20 mg/kg/day CT7001 to beagle dogs for 7 days was also well tolerated with no unscheduled deaths or macroscopic or microscopic changes. However, food consumption was reduced over the dosing period; subsequent moistening of the food showed increased consumption. A significant decrease in reticulocyte numbers was observed. Erythroid enucleation is believed to be dependent on the activity of transcriptionally active CDKs, including CDK7 (Wölwer et al, 2015). Therefore, the reduction in reticulocytes was interpreted as a pharmacological effect of CT7001 and considered as another PDc biomarker of potential utility in early clinical development of CT7001.

The main effects of toxicological significance produced by CT7001 in both rats and dogs are its manifestation in tissues with rapidly dividing cells, namely bone marrow, lymphoid tissue and the gastro-intestinal tract. All effects partially or fully reversed within 4 weeks from cessation of dosing and are consistent with the pharmacological mode-of-action of the drug. CT7001 is not phototoxic.

CT7001 showed no inhibition of human ether-a-go-go-related gene (hERG) channel tail current in a test using the whole-cell patch-clamp technique (human embryonic kidney [HEK] cells transfected with hERG), and the CT7001 IC_{50} in the K^+ channel was determined to be greater than 5 µM.

Cardiovascular toxicity potential was also studied *in vivo* in dogs. Oral administration of 5, 15, and 20 mg/kg CT7001 had no effect on haemodynamic parameters, ECG parameters, or body temperature in the dog, compared with control article (vehicle) administration.

Effects of CT7001 on the central nervous system (CNS [Irwin]) and respiratory systems were assessed in rats. No significant effects were observed in the test article groups.

CT7001 has low mutagenic potential. At concentrations up to the lower limit of toxicity, it induced no mutations in a five strains Ames test. Low potential for genotoxicity was demonstrated in micronucleus tests with human peripheral blood lymphocytes.

2.3.2 Current Clinical Data of CT7001

A first-in-human multi-module Phase I/II clinical study commenced in November 2017 (EudraCT number: 2017-002026-20; ClinicalTrials.gov identifier: NCT03363893). This is the only clinical study conducted to date.

The single- and multiple-ascending dose Phase 1a part of the study (Module 1, Part A) is completed. 33 dosed patients have completed this part of Module 1 (120, 240, 480 mg and a step-down dose level of 360 mg, all taken once daily (OD), and 180 mg given twice a day (BID), all in a fasted state). Module 1A also included separate, additional cohorts with paired biopsies pre-dose and on study, these cohorts have now completed with 11 patients with BC dosed.

Module 4 evaluated the effect of food on CT7001 bioavailability in cancer patients. Two single doses of 120 mg (Cohort 1) or 360 mg (Cohort 2) were given to assess the effect of food on the bioavailability of CT7001. Patients then continued on daily dosing, which originally was 240 mg and changed to 360 mg after determination as recommended Phase 2 dose in Module 1A. Recruitment to Module 4 is complete with 15 patients dosed and 12 patients evaluable for testing of food effect. The results are summarised in Section 2.3.2.2.

Module 1B is a Phase 1b expansion to refine the safety, tolerability, and PK and PDc profiles of CT7001 in patients with advanced solid malignancies. 4 cohorts, each up to 25 patients, may be opened. Cohort 1B-2 (CRPC) has completed recruitment with 11 patients dosed. Other cohorts potentially include SCLC, Ovarian cancer and other appropriate cancer indications.

Module 1B-1 (TNBC) is a larger expansion cohort of CT7001 at 360 mg OD in up to 50 patients with triple negative breast cancer (TNBC). Recruitment to this cohort is now closed with 23 patients dosed. This is an open-label, uncontrolled Phase Ib study to determine the recommended Phase 2 dose of CT7001 as monotherapy, further characterize safety, tolerability and blood concentrations of CT7001, and explore its anti-tumour activity in triple negative breast cancer.

Module 2 is a [redacted] Phase 2 study designed to evaluate the safety, tolerability and efficacy of CT7001 in combination with fulvestrant in patients with HR+ve / HER2-ve breast cancer.
Part A is a single arm open label study in up to 30 patients to establish the recommended

CT7001 dose (240 mg or 360 mg OD) [redacted]

[redacted]

Table 1 provides a summary of the current enrolment and dosing status in Modules 1A, 1B, 2 and 4.

Table 1: Status of Module 1A, 1B, 2 and 4 as of 16-Nov-2020

Module	Cohorts	Dose	Recruited	Dosed	Ongoing
1A	Cohort 1	120 mg OD	8	6	0
1A	Cohort 2	240 mg OD	7	7*	0
1A	Cohort 3	480 mg OD	8	6	0
1A	Cohort 4	360 mg OD	6	6	0
1A	Cohort 5	180 mg BID	9	8	0
1A Paired Biopsy	Breast Cancer	240 mg OD	9	5	0
1A Paired Biopsy	Breast Cancer	360 mg OD	9	6	0
4	Cohort 1	120 $\rightarrow$ 240 mg OD	9	8	0
4	Cohort 2	360 $\rightarrow$ 360 mg OD	9	7	1
1B-1 (TNBC)	N/A	360 mg OD	36	23	3
1B-2 (CRPC)	N/A	360 mg OD	15	11	1
2	Part A	240 mg OD	12	6	0
2	Part A	360 mg OD	25	16	8
	**TOTAL**		**162**	**115**	**13**

* Includes 1 replacement patient from Cohort 3 who had a starting dose of 240 mg

The subsections below provide a synopsis of the clinical data as recorded in the database as of 16-Nov-2020. Please refer to the IB for detailed information. The data reported here and in the IB is to be viewed as preliminary as study CT7001_001 is ongoing, information continues to be rapidly evolving and the database has not been locked and thus most data has not been cleaned yet.

2.3.2.1 Safety

As of 16 November 2020, data is available from 93 patients dosed with CT7001 as monotherapy (M1A, M1B and M4) and 22 patients dosed with CT7001 in combination with Fulvestrant.

In general, CT7001 has shown good safety and acceptable tolerability. Most frequently recorded were diarrhoea, nausea and vomiting, which occurred in more than 75% of patients across dose levels, largely at Grade 1. There was no apparent relationship with dose or with blood concentrations of CT7001 (C_{max}, AUC or trough levels).

Diarrhoea is an expected target-related adverse effect. Nausea and/or vomiting started usually a few hours after administration of CT7001. It cannot be excluded that this is related to C_{max}. This prompted to explore BID dosing in Cohort 5 which, however, did not appear to reduce the incidence of vomiting or of nausea. A current working hypothesis is that a main cause of vomiting as well as nausea may be local chemical irritation of the gastric mucosa and taking the drug after a meal may ameliorate these effects. Therefore, Module 4 (which has evaluated the impact of food on the bioavailability of CT7001) was brought forward in the development program. Food had no clinically significant effect on overall CT7001 exposure at RP2D. Fed dosing is now permitted across the program. The therapeutic or preventive use of common anti-emetics had a positive effect in some patients but little effect in others.

Laboratory abnormalities were uncommon.

- There have been 14 events of increased ALT and 16 events of increased AST reported; the majority of these have been Grade 1
- There have been 16 events of anaemia reported; the majority of these have been Grade 1 or 2
- At 240mg OD and 360mg OD there appears to be a ~20% drop in platelet count in all patients. This appears over the first 15 days on study and then is stable for the duration on treatment; in the majority of patients this is within the normal range of platelet counts. All changes in platelet counts appear fully reversible upon discontinuation of CT7001. There have been 13 platelet related AEs reported:
    - 2 events of Grade 4 thrombocytopenia (1at 180mg BID and 1 at 360mg OD); the event at 180mg BID was associated with minor nose bleeding
    - 1 event of Grade 3 thrombocytopenia (at 360mg OD)
    - 2 events of Grade 2 thrombocytopenia (at 360mg OD)
    - 1 event of Grade 2 platelet count decreased (at 360mg OD)
    - 4 events of Grade 1 thrombocytopenia (at 360mg OD)
    - 3 events of Grade 1 platelet count decreased (1 at 240mg OD and 2 at 360mg)
- Of note, only 2 events of neutropenia/white blood cell count decreased (Grade 1 at 360mg OD and 180mg BID) have been reported.

Ten serious adverse events (excluding those deemed not related and unlikely related to treatment with CT7001 by the investigator) were reported in 115 subjects. The related events concerned:

- Thrombocytopenia: 2 events of Grade 4 were reported in patient M1A01C501, dosed at 180mg BID and in patient M1B04E1C106, dosed at 360mg OD

- Oesophagitis and gastro-oesophageal reflux disease: 2 Grade 3 events in patient M1A01R503, dosed at 180mg BID
- Diarrhoea: Grade 2 in patient M1A02R504, dosed at 180mg BID
- Anaemia (Grade 3), diarrhoea (Grade 3) and dyspnoea (Grade 2) in patient M1B04E1C106 dosed at 360mg OD
- Nausea: Grade 3, in patient M40105, dosed at 360mg OD
- Diarrhoea: Grade 2 in patient M2A04C101 dosed at 240 mg

These cases were reported to regulatory authorities as serious unexpected adverse drug reactions (SUSAR). No death occurred which investigators attributed to study therapy.

Dose-limiting toxicities were only recorded at the non-tolerated dose of 480 mg OD (5 events) and on 180 mg given BID (5 events). These included 8 events which were defined as dose-limiting toxicity (DLT) in the study protocol. These were 1 case each of CTCAE Grade 3 diarrhoea, oral mucositis and vomiting at the non-tolerated dose of 480 mg OD, and 1 case each of Grade 4 thrombocytopenia, Grade 3 weight loss, Grade 3 anorexia, Grade 3 dysphagia/oesophagitis and Grade 3 heartburn at 180 mg BID. The Safety Review Committee judged two additional events to represent a DLT on clinical grounds. One event was Grade 2 nausea and the other Grade 2 vomiting, each recorded at 480 mg OD in the same patient.
All adverse effects which investigators attributed to CT7001 were reversible upon interruption or discontinuation of study therapy.

2.3.2.2 Pharmacokinetics (PK)

Module 1A

Final PK data based on cleaned data and actual sampling time are available from Study CT7001_001 for Cohorts 1 to 5 (120, 240, 360 and 480 mg OD doses and 180 mg BID dose, all fasted). The single- and multiple-dose pharmacokinetics of CT7001 were evaluated using a sample-rich, non-compartmental analysis approach. Initially the single-dose PK samples were taken up until 48 hours (Cohorts 1, 2, and 3). This was found to be insufficient for reliable identification of the terminal elimination phase and derivation of the associated parameters: half-life, lambda_z, $AUC_{0-\infty}$, CL/F, Vz/F, MRT. Thus, further PK sample times points at 72, 120 and 168 hours were introduced for later cohorts to facilitate better characterisation of the single-dose pharmacokinetics. The majority of subjects reported either vomiting or diarrhoea adverse events on PK days that could conceivably have affected exposure, however as this occurred is so many patients none were excluded from the PK population. After oral administration CT7001 was rapidly absorbed with median T_{max} ranging from 1.5 to 4 hours across the cohorts. The absorption phase for CT7001 is characterised by double peaking in some subjects , plasma concentrations then underwent bi-phasic decline. Four of the six subjects dosed 360 mg and all the subjects (8) dosed 180 mg BID experienced the longer sampling regimen to 168 hours. For these subjects the determined half-life ranged (geometric mean) from 53.80 - 101.4 (76.41) hours and 48.68 - 87.45 (74.37) hours for the 360 mg and 180 mg doses respectively. The geometric mean accumulation ratio at steady-state ranged from 2.060 - 3.080 across the dose cohorts. Plasma exposure appeared to increase dose-proportionally after single and multiple dosing. Final multiple dose PK data based on cleaned data and actual sampling time are available from Study CT7001_001 for the Part A Paired Biopsy Breast Cancer Expansion Cohort and are consistent to those observed for cohorts 1 to 5.

Module 4

Preliminary PK data based on uncleaned data and nominal sampling time are available from Study CT7001_001 Module 4 for 120 and 360 mg single dose in the fed (high fat, high calorie meal) and fasted state. PK in the fasted state was similar to that observed in Module 1 with rapid absorption observed with median T_{max} of 1.5 and 4 hours respectively for the 120 mg and 360 mg cohorts and geometric mean half-life of 54.64 and 65.45 hours. After dosing in the fed state at 120 mg dose plasma concentrations appeared to be reduced and PK could not be accurately determined. As there was no safety concern of increased exposure after dosing in the fed state the dose was increased to the RP2D (360 mg) and the impact of dosing in the fed state assessed. After dosing in the fed state T_{max} was delayed by 3 hours with a median T_{max} of 7 hours. C_{max} appeared to be reduced with a ratio of geometric means fed : fasted of 0.84 (90% CI = 0.53 - 1.32) while overall exposure appeared to be unaffected when dosing in the fed state with a ratio of AUC_{0-72} geometric means fed : fasted of 0.96 (90% CI = 0.61- 1.50). Geometric mean half-life in the fed state was 61.24 hours.

Module 2

PK data for the CT7001-Fulvestrant combination is in the early stages of evaluation. Initial interpretation, based on unclean data on nominal times, is that there is no drug-drug interaction, however this remains to be confirmed in a larger data set.

2.3.2.3 Pharmacodynamics (PDc)

PDc data is currently available from surrogate normal cells. Data from paired tumour biopsies is pending. In normal cells, two PDc effects of CT7001 were evaluated, a biochemical and a biomechanical effect of CDK7 inhibition.

Based on the rationale and non-clinical data described in Sections 2.2 and 2.3.1, pPolII in PBMCs was used as biochemical PDc biomarker. Figure 1 illustrates the significant reduction of pPolII signal by ~30% induced by CT7001 in PBMCs across all dose levels tested in Module 1A for patients who have completed 21 days of treatment (i.e., cycle 2 day 1 timepoint). Later timepoints also show inhibition, although this does not reach statistical significance due to the low number of subjects. This should be viewed in context of a maximal 55% reduction in signal observed in PBMCs incubated with CT7001 *ex vivo*.

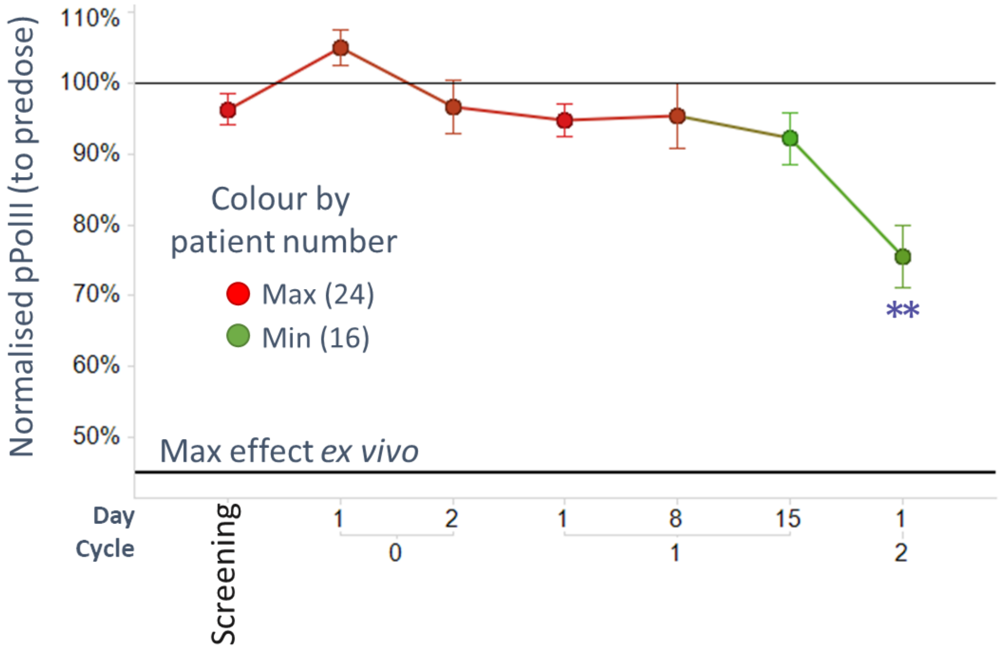

[i]**Figure 1: CT7001 decreases pPolII levels in PBMC**

pPolII was determined by a flow cytometry assay on subject samples of PBMC preparations derived from blood. Note that samples were taken pre-dose at all timepoints. Statistical comparison between pre-treatment samples (Cycle 1 day 1) and subsequent timepoints were done using a 1-tailed Mann-Whitney test. ** data are significantly different to predose levels with p < 1%.

2.3.2.4 Minimum Biologically Active Dose (MBAD)

The study's SRC originally declared 240 mg OD as MBAD based on observed target toxicity (diarrhoea), target-mediated PDc effect on reticulocyte counts and preliminary signals of anti-tumour activity. This prompted the opening of a cohort with paired breast cancer biopsies at 240 mg OD. Later review of the data from cohort 1 at the starting dose of 120 mg OD revealed similar biological activity as observed for 240 mg OD, including PDc and anti-tumour effects. Accordingly, 120 mg OD represents the lowest dose tested to date in humans that has demonstrated biological activity.

2.3.2.5 Non-Tolerated Dose

At 480 mg, 3/6 subjects experienced a protocol defined dose-limiting toxicity (1 case each of Grade 3 diarrhoea, mucositis or vomiting). Furthermore, 3 additional patients experienced G1-2 vomiting with little effect of anti-emetic therapy. As a result, the study's safety review committee (SRC) defined 480 mg as non-tolerated dose when given OD in a fasted state.

2.3.2.6 Maximum Tolerated Dose (MTD) and Preliminary Recommended Phase 2 Dose (RP2D)

Five patients in Cohort 4 (360 mg OD in fasted state) have completed Cycle 1 without recording a DLT or other concerning safety or tolerability findings. Accordingly, this is the dose which has been taken forward to Module 4 Cohort 2, Module 1B-1 (TNBC), Module 1B-2 (CRPC), [redacted] and this is the target dose for use in combination with fulvestrant in Module 2.

2.3.2.7 Anti-Tumour Effects

Preliminary signals of anti-tumour effect have been observed. As of 16-Nov-2020, 3 RECIST partial response have been observed, 2 in patients treated with monotherapy CT7001 and 1 patient treated with CT7001 in combination with Fulvestrant:

- Subject M1A03E102, a 59-year-old female with HR+ breast cancer, with visceral disease; metastases in liver and spleen. This patient had previously been treated with 5 lines of hormonal therapy (exemestane. Letrozole, tamoxifen, anastrozole, fulvestrant) and 4 lines of chemotherapy (paclitaxel, eribulin, capecitabine, 5-FU/epirubicin/cyclophosphamide)
- Subject M1B04E1C107, a 49-year old female with TNBC, with metastases in axilla and lateral node. This patient had previously been treated with 2 lines of chemotherapy (neo adjuvant paclitaxel and carboplatin and palliative gemcitabine and carboplatin)
- Subject M2A02C102, a 53-year old female with HR+ breast cancer, with visceral disease; metastases in liver and lung. This patient had previously been treated with 2 lines of hormonal therapy (tamoxifen and letrozole) and a CDK4/6 inhibitor (palbociclib)

PSA reductions were also observed in all 4 CRPC patients recruited into M1A and M4. This triggered the M1B expansion in patients with CRPC to explore this finding further in patients with RECIST measurable disease.

Across all dose levels explored in Module 1A and 4, the number of patients with disease control (as defined by RECIST SD+PR+CR and/or PSA reduction with no RECIST PD) in those patients evaluable for assessment (as defined by having baseline and at least the first on treatment tumour assessment completed) was as follows:

- All dose levels: 16/46 (36%) at ⩾ 8 wks and 8/46 (17%) at ⩾ 16 wks
- Clinically relevant doses (240mg OD and 360mg OD): 14/31 (45%) at ⩾ 8 wks and 8/31 (26%) at ≥ 16 wks

<u>Module 1, Part B: TNBC patient expansion</u>
In this cohort of 23 patients with advanced TNBC, who had previous received ≤ 3 lines of chemotherapy for their advanced disease, 19 were evaluable for tumour response. 1/19 patients had a PR and 13/19 patients had stable disease as their best response; 5 patients were treated for ≥ 24 weeks and 2 patients for ≥ 1 year.

<u>Module 1, Part B: CRPC patient expansion</u>
In this cohort of 11 CRPC patients, with disease measurable by RECIST, 5/10 patients had stable disease as their best response (1 patient was not evaluable for tumour response); no CRs or PRs were observed. 3 patients were treated for ≥ 24 weeks.

<u>Module 2, Part A: HR+ Breast cancer, in combination with fulvestrant</u>
In this cohort of 22 patients with advanced HR+, HER2- breast cancer, who had previously received ≤ 2 lines of endocrine treatment for their advanced disease, 15 were evaluable for tumour response. 1/15 patients had a PR and 9/15 patients had stable disease as their best response. 6 patients were treated for ≥ 16 weeks.

2.3.2.8 Summary of Current Clinical Experience

The currently clinical experience of CT7001 has shown good safety and PK behaviour, positive PDc effects in surrogate normal cells and preliminary signs of anti-tumour effect; warranting further clinical investigation.

3 OBJECTIVES AND ENDPOINTS

3.1 Objectives

Primary Objectives:

- To further characterize the safety and tolerability of CT7001 and determine the most appropriate dosing regimen for subsequent Phase 2 testing (definitive RP2D)

Secondary Objectives

- To evaluate the activity of CT7001 as monotherapy in patients with metastatic or locally advanced TNBC.
- To further evaluate CT7001 plasma concentrations.
- To evaluate CYP2D6 polymorphisms in this patient population.

Exploratory Objectives

- To further investigate the presence and identity of metabolites of CT7001 and, if appropriate, characterize their PK.
- To further explore the relationship between PK and safety, anti-tumour activity and biological activity and the impact of patient characteristics on PK.
- To further explore the effects of CT7001 on PDc gene expression (e.g., c-Myc, MCL-1).
- To further explore mutations and expression in genes, proteins and RNAs relevant to the cell cycle (e.g., phosphorylation of CDK1 and Rb proteins), drug target engagement (e.g., c-Myc, MCL-1) and tumour sensitivity and/or resistance in tumour-derived materials including tumour tissue, circulating tumour deoxyribonucleic acid (DNA) and circulating tumour cells (e.g., p53, CDK7, ER, AR).

3.2 Endpoints

Primary Endpoints

- Type, incidence, severity (as graded by CTCAE v5.0), seriousness and relationship to study medications of adverse events (AE) and any laboratory abnormalities.

Secondary Endpoints

- Objective response rate (ORR)

- Duration of response (DOR)
- Disease control (DC: CR or PR or SD ≥24 weeks)
- Best percent tumour size change
- Progression-free survival (PFS)
- Trough plasma concentrations of CT7001

Exploratory Endpoints

- Metabolites of CT7001
- Biomarkers in PBMC, circulating tumour DNA, circulating tumour cells and tumour tissue, including genes, RNA expression and proteins (e.g., c-Myc, MCL-1, phosphorylated CDK1 and Rb proteins, p53, CDK7, ER, AR)

4 STUDY POPULATION

Subjects who fails screening may be re-screened only on approval of the SRC and Sponsor. Any subject re-screened will need to provide new informed consent and will be allocated a new study number.

4.1 Inclusion Criteria

To be eligible for the study patients have to meet **all** of the following criteria:

1. Patients at least 18 years of age.
2. Histologically confirmed carcinoma of the breast not expressing oestrogen receptor (ER) and progesterone receptor (PgR) and negative for human epidermal growth factor receptor 2 (HER2).
   - Assessment of ER, PgR and HER2 in breast carcinoma tissue will be based on results from local pathology laboratories. Independent central review is not intended.
   - Negative assessment for ER, PgR and HER2 by local laboratories should be consistent with the criteria described in the most recent versions of the ASCO/CAP guidelines for testing of ER, PgR and HER2, respectively (Hammond et al, 2010; Wolff et al, 2018):
     - ER- and PgR-negativity is determined as <1% of tumour cells positive by IHC utilizing an IHC assay consistent with local standards.
     - HER2-negativity is determined as immunohistochemistry score 0/1+ or negative by in situ hybridization (FISH/CISH/SISH) defined

as a HER2/CEP17 ratio <2 or for single probe assessment a HER2 copy number <4.

- Determination of negative ER, PgR and HER2 status should be based on data from the most recent tumour biopsy. In case no tumour biopsy was performed after initial resection of the primary tumour, the original tumour tissue serves as basis for assessment of ER/PgR/HER2 status.

3. Metastatic or locally advanced disease not amenable to resection or radiation therapy with curative intent with documented progressive disease on or within 6 months of most recent prior chemotherapy.
4. Disease must be measurable by Response Evaluation Criteria in Solid Tumours (RECIST, version 1. 1, Appendix C):
5. Patients must have received at least one cytotoxic chemotherapy regimen for metastatic/locally advanced disease.
6. ECOG performance status 0 or 1 with no deterioration over the previous 2 weeks (Appendix F).
7. Expected life expectancy of greater than 12 weeks.
8. Ability to swallow and retain oral medication.
9. No childbearing potential, defined as women:
   - Who had prior hysterectomy or bilateral surgical oophorectomy or are medically postmenopausal (defined as spontaneous cessation of regular menses for at least 12 consecutive months or follicle-stimulating hormone (FSH) and oestradiol blood levels in the testing laboratory's respective postmenopausal range with no alternative pathological or physiological cause).
10. Women of childbearing potential must be willing to practice effective contraception (defined as abstinence i.e., refraining from heterosexual intercourse during the entire period of risk associated with the study treatment, sex only with person of the same sex, sex only with vasectomized partner, intrauterine device, or double-barrier methods) for the duration of the study and for 6 months after the last dose of CT7001. Single barrier methods (e.g., condom or diaphragm alone) are not considered effective contraception methods.
11. Women of childbearing potential must have a negative serum pregnancy test at baseline (within 7 days prior to first dose of CT7001).
12. Sexually active male subjects must be willing to use condoms with all sexual partners for the duration of the study and for 3 months after the last dose of CT7001. If a female partner is a woman of childbearing potential who is not using effective contraception (as defined above), the subject must use a condom with spermicide during the study and for 6 months after the last dose of CT7001.
13. Patients are willing and able to comply with scheduled visits, treatment plan, laboratory tests, and other study procedures.

14. Personally signed and dated written informed consent indicating that the patient has been informed of all pertinent aspects of the study before any study-specific activity is performed.

Host Genetics Research Study - Pharmacogenomics Samples (Optional)
Patients who meet all of the following criteria may be included in optional genetics substudies:

1. Provision of signed and dated, written informed consent for the genetic research.

4.2 Exclusion Criteria

To be eligible for the study patients **may not have any** of the following exclusion criteria:

1. More than three lines of cytotoxic chemotherapy for metastatic and/or locally advanced disease.
2. Advanced, symptomatic, visceral metastases if risk of life-threatening complications in the short term (including patients with massive uncontrolled effusions [pleural, pericardial, peritoneal], pulmonary lymphangitis and over 50% liver involvement).
3. Known symptomatic CNS metastases, carcinomatous meningitis or leptomeningeal disease. Patients with a history of CNS metastases or spinal cord compression due to metastasis are eligible if they have been treated with local therapy (e.g., radiotherapy, stereotactic surgery) and are clinically stable off anticonvulsants and steroids for at least 4 weeks before first dose of CT7001.
4. Inadequate hepatic, renal, bone marrow or cardiac function, specified as follows:
    a) Hepatic:
        i. Aspartate aminotransferase (AST) and alanine aminotransferase (ALT) > 2.5 × ULN or > 5.0 × ULN for patients with liver metastases.
        ii. Total bilirubin > 1.5 × ULN (>3.0 x ULN if known Gilbert's disease).
        iii. Albumin < 30 g/L.
        iv. Liver function deteriorating in a manner that would likely make the subject not meeting the AST, ALT, bilirubin or albumin levels specified above at the time of the first dose of CT7001.
        v. Other evidence of impaired hepatic synthesis function.
    b) Renal:
        i. Serum creatinine > 1.5 × ULN.
    c) Bone marrow:
        i. Absolute neutrophil count ≤ 1.5 × 10^9/L.

ii. Platelet count <100 × 10^9/L.

iii. Haemoglobin < 90 g/L.

d) Cardiac:

i. Myocardial infarction within 6 months of study entry, unstable angina, unstable arrhythmia.

ii. New York Heart Association Class > I heart failure (see Appendix D).

iii. Mean resting QT interval corrected for heart rate by the Fridericia formula (QTcF) > 470 msec obtained from 3 ECGs obtained within 3-5 minutes apart.

iv. Clinically important abnormalities in rhythm, conduction, or morphology on resting ECG (e.g., complete left bundle branch block, third degree heart block).

- Controlled atrial fibrillation is permitted.

v. Any factor that may increase the risk of QTc prolongation or of arrhythmic events (e.g., hypokalaemia, congenital long QT syndrome, immediate family history of long QT syndrome or unexplained sudden death under 40 years of age).

5. Unresolved toxicity (except alopecia) from prior therapy of ≥ Grade 2 according to CTCAE version 5.0.
6. Refractory nausea and vomiting, chronic gastro-intestinal disease or previous significant bowel resection, with clinically significant sequelae that would preclude adequate absorption of CT7001.
7. Uncontrolled seizures.
8. Active infection requiring systemic antibiotic, antifungal, or antiviral medication within 14 days prior to first dose of CT7001.
9. Severe or uncontrolled medical condition (e.g., severe chronic obstructive pulmonary disease, severe Parkinson’s disease, active inflammatory bowel disease or psychiatric condition).
10. Active bleeding diatheses.
11. History of haemolytic anaemia or marrow aplasia.
12. Renal or other organ transplant.
13. Known hepatitis B, hepatitis C, or human immunodeficiency virus infection.
14. Pregnancy.
15. Breastfeeding.
16. Previous exposure to CT7001.

17. Receipt of systemic corticosteroids (at a dose > 10 mg prednisone/day or equivalent) within 14 days before the first dose of IMP.
18. Non-biological anti-cancer medicines within 28 days or ≤ 5 half-lives, whichever is shorter, before the first dose of CT7001.
19. Biological anti-cancer medicines, including IMP (e.g., monoclonal antibodies, antibody-drug-conjugates) within 42 days before the first dose of CT7001.
20. Receipt of St John's Wort within 21 days before the first dose of CT7001.
21. Concomitant medication, herbal supplement or food that is a strong inhibitor or inducer of CYP2D6, CYP3A4, CYP2C19, or P-glycoprotein activity within 14 days before the first dose of CT7001 (specific examples listed in protocol Appendix B).
22. Receipt of a blood transfusion (blood or blood products) within 14 days before the first dose of CT7001.
23. Known hypersensitivity to CT7001 or any excipient of the product.
24. Diagnosis of any other malignancy within 3 years prior to enrolment, except for adequately treated basal cell or squamous cell skin cancer, or carcinoma in situ of the cervix.
25. In the opinion of the Investigator, unlikely to comply with study procedures.
26. Patients who are investigational site staff members directly involved in the conduct of the trial and their family members, site staff members otherwise supervised by the Investigator, or patients who are Carrick employees directly involved in the conduct of the trial.
27. Has received a live-virus vaccination within 28 days or less of planned treatment start. Note: seasonal flu vaccines that do not contain live virus are permitted.

Host Genetics Research Study - Pharmacogenomics Samples (Optional)
Patients who meet any of the following criteria will be excluded from optional genetic substudies:

1. Allogenic bone marrow transplant.
2. Non-leucocyte-depleted whole blood transfusion within 120 days of the date of the genetic sample collection.

5 DESIGN OF MODULE 1B-1

Module 1B-1 represents part B, Cohort 1 of the ongoing Phase I study of CT7001 in patients with advanced solid tumours and is an open-label, uncontrolled Phase I expansion study in patients with metastatic or locally advanced TNBC who had previously received at least one line of cytotoxic chemotherapy for metastatic and/or locally advanced disease. To be eligible, patients must have documented progressive disease on or within 6 months of the most recent

prior chemotherapy that is measurable by RECIST criteria and may not have received more than three lines of prior cytotoxic chemotherapy for metastatic and/or locally advanced disease.

Module 1A has been recruiting patients with a variety of cancer types, a dosing cohort included usually no more than 6 patients and few patients continued dosing for 4 months or longer. Accordingly, data on the safety of each tested dose level has to be considered as preliminary and evaluation of anti-tumour effects is limited. Therefore, the first dosing regimen of CT7001 to be tested in Module 1B (360 mg once daily) is considered a preliminary RP2D.

Module 1B will involve cohort expansions of specific subject groups which may include, but is not restricted to: triple-negative breast cancer (TNBC), small cell lung cancer (SCLC), ovarian cancer and most recently, prostate cancer (PCA). Cohort expansions will each be up to 25 evaluable subjects.

Module 1B-1 (TNBC) is an expansion cohort of up to 50 subjects and therefore will be conducted in accordance with Volume 3 of the protocol.

A primary objective of Module 1B-1 (TNBC) is to determine a definitive RP2D regimen, taking into account acute, sub-acute and chronic safety and tolerability, efficacy as well as patient convenience. Achieving that objective may require testing of more than one dosing regimen in Module 1B-1 (TNBC). Accordingly, the Module 1B-1 (TNBC) study protocol allows for inter-patient modification of dose and/or schedule.

As in Module 1A, the SRC will determine whether inter-subject modification(s) of the CT7001 dosing regimen appear indicated and which specific regimen to test in a next cohort of patients. The SRC will meet regularly to review all available data on safety and anti-tumour effects from Module 1B-1 but also the data from other study modules which will be open at the same time, specifically the paired breast cancer cohort in Module 1A, Module 1B and Module 2.

In assessing whether and how to modify the CT7001 dosing regimen in a subsequent group of Module 1B-1 patients, the SRC will consider the totality of available data. Modification options include but are not limited to lowering the daily dose, administration of CT7001 twice a day rather than once daily and the introduction of a treatment holiday (e.g., two or three weeks on and one week off). An inter-subject increase in total daily dose beyond the initial dose of 360 mg will only be permissible if that dose was previously evaluated in Module 1A and found to be safe. In case inter-subject modification of the dosing regimen appears indicated, this would prompt a major protocol amendment.

In case the food effect study shows no significant effect on the bioavailability of CT7001, the SRC may allow administration of CT7001 after food. This may apply to new patients but also to patients who are already on study treatment at the time of such a decision. Administration of CT7001 after food will not represent an inter-subject dose modification.

Module 1B-1 will include sparse blood sampling for analysis of CT7001 blood concentrations. Module 1B-1 also includes various optional procedures and analyses which require separate informed consent. These include sequential collection of blood samples for various types of PDc analyses in white blood cells, circulating tumour DNA and/or tumour cells as well as possible future research purposes, sequential biopsies of readily accessible tumour lesions to further explore the effects of CT7001 on PDc biomarkers in tumour tissue, and pre-therapy blood samples for pharmacogenomic testing.

The initial sample size will be a maximum of 50 patients, of whom approximately 30 patients should receive the CT7001 dosing regimen which will be declared as the definitive RP2D. In the event of an ORR of >30%, combined with good durability and acceptable safety and tolerability, Module 1B-1 may get amended to a single arm Phase 2b study with enrolment of up to 130 patients. The primary objective of Phase 2b will be to evaluate the efficacy of CT7001, with objective response rate as the primary endpoint.

Module 1B-1 will include an interim efficacy analysis when 15 patients in the Recommended Phase 2 Dose (RP2D) population are evaluable for efficacy assessment (see section 10.3.6). M1B-1 TNBC expansion phase has been designed using a 2-stage design. The null hypothesis for the ORR is 10% and this will be tested against an alternative hypothesis of ≥ 30%. If 0 or 1 responses are observed in the first 15 evaluable patients, no further patients will be recruited, and the alternative hypothesis will be rejected. Otherwise, recruitment will continue until a total of 30 evaluable patients are recruited and if ≥6/30 (20%) responses are observed, across both stages of the trial, the null hypothesis will be rejected. This design yields a 1-sided type I error rate of <7.5% and power of >90% when the true response rate is 30%. In case the interim analysis suggests a reasonable possibility of observing an ORR >30% at the end of Module 1B-1, work on a possible subsequent amendment to a Phase 2b study may commence.

In accordance with the interim efficacy analysis, CT7001_001 Protocol Volume 3 (Module 1B-1 TNBC) version 4.0 dated 30th January 2020 the timepoint for evaluation was met on 3rd March 2020. At this timepoint the interim efficacy requirements of 2 or more patients achieving a RECIST partial response had not been met.

1 patient in the first 15 evaluable patients with TNBC treated with CT7001, achieved a partial response, defined by RECIST, in their tumour burden. However, in addition 8 out of 15 evaluable patients achieved stable disease. 4 of these evaluable patients were treated for at least 16 weeks, which in this aggressive tumour type is of significant clinical note. 2 evaluable patients have been treated for more than 36 weeks.

Following discussion with the Data Monitoring Committee (DMC), in view of the clinical benefit seen in 8 of the 15 evaluable patients in addition to the partial response seen in 1 evaluable patient enrolled, it has been decided to modify the protocol to allow the full cohort of 30 evaluable patients to be recruited into this expansion.

Patients will undergo regular safety and efficacy assessments as outlined in the Schedule of Events – Mandatory Procedures (Table 5).

Patients will continue to receive study treatment until objective disease progression (Section 8.2), symptomatic deterioration, unacceptable toxicity, death, or withdrawal of consent, whichever occurs first.

Patients who discontinue study treatment for reasons other than documented progressive disease as per RECIST v.1.1 (Section 8.2), will continue to have tumour assessments performed during the follow-up visits every 8 weeks (±7 days) and bone scans (if applicable) every 16 weeks (± 7 days) until RECIST-defined disease progression (Section 8.2), initiation of new anticancer therapy or discontinuation of patient from overall study participation (e.g., death, patient's request, lost to follow-up), whichever occurs first. Efficacy analyses in Module 1B-1 do not include survival.

Efficacy analyses will be performed using the local radiologist's/investigator's tumour assessments as data source. The Sponsor reserves the right to request an anonymised independent review of subject scan data if required.

6 STUDY TREATMENT

6.1 Drug Supply

The investigational product used in this trial is called CT7001 (Samuraciclib), will be supplied by the sponsor.

6.1.1 Pharmaceutical Properties

Chemical (IUPAC) name:

(3R,4R)-4-[[[7-(benzylamino)-3-isopropyl-pyrazolo[1,5-a]pyrimidin-5-yl]amino]methyl]piperidin-3-ol

Laboratory Code: CT7001, ICEC0942

International Non-Proprietary Name: Samuraciclib

Structural formula:

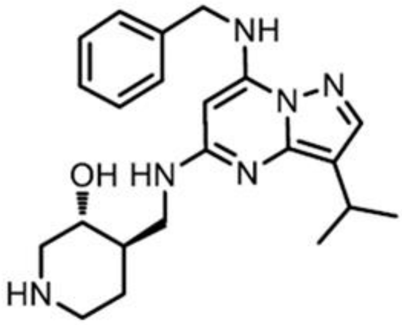

6.1.2 Formulation, Packaging and Storage

CT7001 will be supplied as capsules containing 60 mg or 120 mg equivalents of CT7001 free base and the excipients listed in Table 2.

Table 2: CT7001 Excipients

Material	(%w/w)
CT7001 (drug substance)	30.00
Microcrystalline Cellulose (Avicel PH 102)	64.50
Sodium starch glycolate (Explotab®)	5.00
Magnesium Stearate (Hyqual®)	0.25
Silica Colloidal Anhydrous (Aerosil 200)	0.25
**Total**	100.00

The capsules at each strength are opaque white hydroxypropyl-methylcellulose capsule shells. Capsules with 60 mg are size 1 and capsules with 120 mg size 00, respectively.

The sponsor, through his delegate, will supply the oral drug formulation to sites in high-density polyethylene bottles containing 60 mg or 120 mg capsules. Bottles are secured with a child-resistant and tamper-evident closure.

All Investigational Medicinal Product (IMP) will be kept in a secure place under appropriate storage conditions as specified on the IMP label.

6.2 Dispensing

An Interactive response system (IXRS) will be used to allocate CT7001 medication bottles to the patient, full details can be found in the IXRS Reference Manual.

The patient number should be recorded on the bottle label, in the spaces provided, by site personnel at the time of assignment to patient. Site personnel must ensure that patients clearly understand the directions for self-medication.

Patients should be given a sufficient supply to last until their next study visit. Unused drug and/or empty bottles should be returned to the site at the next study visit.

Patients should be instructed to keep their medication in the bottles provided and not transfer it to any other container.

6.3 Dosing Regimen of CT7001

Module 1A has determined 360 mg given once a day in a fasted state as preliminary RP2D regimen with appropriate safety and tolerability for further investigation. Accordingly, this is the CT7001 dosing regimen used at the start of Module 1B-1. As in Module 1A, a treatment cycle is defined operationally as 21 days.

For dosing regimens with OD dosing, each daily dose should be taken around a similar time of the day. Which time of the day is at the patient's discretion.

For potential dosing regimens with BID dosing, the two doses should be taken 9-12 hours apart.

Module 4 evaluated the effect of food on the bioavailability of CT7001 in cancer patients. On 10th June 2019 the Safety Review Committee reviewed the PK data and determined there was no significant effect observed in AUC in the fed phase of the Module 4, the requirement to fast was removed. CT7001 may be taken orally in either a fasted or fed state, where a patient experiences nausea or vomiting Investigators are recommended to advise their patients to consume CT7001 after a meal.

6.4 Drug Administration

CT7001 may be taken orally in either a fasted or fed state.

Where a patient experiences nausea or vomiting Investigators are recommended to advise their patients to consume CT7001 after a meal.

Site personnel must ensure that patients clearly understand the directions for self-medication. Patients should be instructed to swallow CT7001 capsules whole and not to chew them prior to swallowing. No capsule should be ingested if it is broken, cracked, or otherwise not intact.

Patients will be asked to capture their medication intake, including whether they were fed or fasted, in a medication diary.

6.5 General Rules

Patients who miss a day’s dose must be instructed NOT to ‘make it up’ the next day.

Patients who vomit any time after taking a dose must be instructed NOT to ‘make it up’ but rather resume treatment the next day as prescribed.

6.6 Medication Dosing Errors

Medication dosing errors in Module 1B-1 may mainly result from the administration of CT7001 at the wrong dosage strength. Such medication errors are to be captured on the IP administration electronic case report form (eCRF) which is a specific version of the adverse event (AE) page, and on the serious adverse event (SAE) form when appropriate. In the event of medication dosing error, the sponsor should be notified immediately.

6.6.1 Overdose

There are no clinical data yet on overdose with CT7001 as this has not occurred to date. There is no definition of what constitutes an overdose. There is no known antidote.

Any subject who receives a higher dose than that intended in the module should be monitored closely, managed with appropriate supportive care, and followed-up expectantly. Such incidence should be recorded as an overdose and will be recorded in the eCRF as follows:

- An overdose with associated AEs will be recorded as an AE of the relevant diagnosis/symptoms on the AE eCRF page and in the overdose eCRF page.
- An overdose with no associated symptoms will be reported only on the overdose eCRF page.
- If an overdose occurs, the Investigator or other site personnel must notify the Emas Pharma Ltd trading as Bionical Emas (Bionical Emas) Medical Monitor immediately but no later than by the end of the next business day of first awareness.

The Bionical Emas Medical Monitor will work with the Investigator to ensure that all relevant information is provided to the safety database: drug.safety@bionical-emas.com

For overdoses associated with an SAE, standard reporting timelines apply (see Section 9.12.1**)**. Other overdoses will be reported within 28 days.

6.7 Intra-Subject Dose Modification

Every effort should be made to administer study treatment on the planned dose and schedule. However, in the event of significant treatment-related toxicity, administration of CT7001 may need adjustment as described in the following sections.

Patients are to be instructed to notify Investigators at the first occurrence of any adverse sign or symptom. In the event of significant treatment-related toxicity, CT7001 dosing may be interrupted, delayed and/or reduced as described below. In the event of multiple toxicities, dose modification should be based on the worst toxicity observed.

No specific dose adjustments are recommended for Grade 1-2 treatment-related toxicity. However, investigators should always manage their patients according to their best medical judgment based on the particular clinical circumstances.

6.7.1 Types of Dosing Modifications

Dosing may be stopped, interrupted (within a cycle) or delayed (at start of a next cycle) and the dose may be reduced.

Note to the following Sections 6.7.1.1, 6.7.1.2, 6.7.1.3 and 6.7.1.4:

- MUST = mandatory
- SHOULD = not mandatory but highly recommended
- MAY = per the investigator’s best clinical judgment

6.7.1.1 Mandatory Treatment Discontinuation

- Grade 4 QTc abnormalities (torsades de pointes, polymorphic ventricular tachycardia, signs and/or symptoms of serious arrhythmia)

6.7.1.2 Dosing Interruption or Delay

The first measure of dose modification is interruption (within a cycle) or delay (at start of a next cycle) of dosing.

Patients experiencing the following adverse events MUST have their treatment interrupted or delayed:

- Grade 3 neutropenia (absolute neutrophil count (ANC) < 1.0 × 10^9/L) associated with a documented infection or fever ≥ 38.5°C.
- Grade 4 neutropenia (ANC < 0.5 x 10^9/L).
- Grade ≥ 3 thrombocytopenia (platelet count < 50 x 10^9/L).
- Grade 3 ≥ anaemia (Hb < 80 g/L and transfusion indicated).
- Grade ≥ 3 diarrhoea, oral mucositis, vomiting or nausea if persistent despite optimal medical treatment.

- Grade ≥ 3 other non-haematological toxicity if persistent despite optimal medical treatment.
- Grade 3 average QTc prolongation (QTc ≥ 501 msec or > 60 msec change from baseline) corrected for heart rate by the Fridericia formula

Appropriate follow up assessments should be performed and proper therapy and medical care, as clinically indicated, should be provided. If a treatment delay results from a decline in haematological parameters, the frequency of laboratory assessments should be increased as clinically appropriate.

Following Grade ≥ 3 thrombocytopenia (platelet count < 50 x 10^9/L), minimally weekly haematological monitoring for 4 weeks MUST be performed after resuming treatment with IMP and additionally as clinically indicated. Whilst not mandated, it would be desirable for patients experiencing thrombocytopenia to undertake a bone marrow biopsy to further elucidate any underlying pathology.

6.7.1.3 Restart of Treatment

Restart of treatment within a cycle or start of a next cycle SHOULD occur when the following parameters have been met:

- ANC ≥ 1.0 × 10^9/L, no fever and full resolution of a documented infection
- Platelet count ≥ 100 × 10^9/ L
- Hb ≥ 80 g/L
- Grade ≤ 1 diarrhoea, oral mucositis and vomiting
- Grade ≤ 2 nausea
- QTc < 481 msec corrected for heart rate by the Fridericia formula and potential contributing causes (e.g., electrolyte imbalance, concomitant medications known to prolong QTc) corrected

When treatment is resumed after Grade ≥ 3 thrombocytopenia (platelet count < 50 x 10^9/L), the dose of CT7001[redacted] MUST not exceed 240mg OD.

In case recovery of the other toxicities takes more than 21 days, permanent discontinuation of CT7001 SHOULD be strongly considered. Treatment resumption for patients recovering from treatment-related toxicity after more than 21 days of treatment interruption or cycle delay but deemed to be deriving obvious clinical benefit per the investigator’s best medical judgment is left at the investigator’s discretion.

Depending on when a toxicity resolved, a treatment interruption may lead to the patient missing all subsequent planned doses within that same cycle. Doses omitted for toxicity are not to be replaced within the same cycle.

In the event of a treatment interruption or cycle delay for reasons other than treatment-related toxicity (e.g., non-cancer related surgery) lasting >2 weeks, treatment resumption will be decided in consultation with the sponsor.

6.7.1.4 Dose Reductions

Following dose interruption or cycle delay the dose of CT7001 may need to be reduced when treatment is resumed.

As noted in Section 6.7.1.1, in case of Grade 4 QTc abnormalities study therapy MUST be discontinued permanently.

Prior to concluding that an episode of prolongation of the QTc interval (Grade ≤3) is due to study drug, thorough consideration should be given to potential precipitating factors (e.g., change in patient clinical condition, effect of concurrent medication, electrolyte disturbance) and possible evaluation by specialist. If IMP causality cannot be ruled out, dose reduction as described below should be performed upon restart of therapy.

In case of other non-haematological Grade 4 toxicities, the dose MUST be reduced as per Table 3 when treatment is resumed.

With the exception of thrombocytopenia, when treatment is resumed after sufficient resolution of the other toxicities listed in Section 6.7.1.3, dose reduction SHOULD be considered but is at the investigator’s discretion. Factors to take into account include the time it took to recover from a given toxicity, clinical sequelae associated with a toxicity and overall risk-benefit assessment per the investigator’s best clinical judgment.

In case the investigator considers a dose reduction indicated for other reasons, this needs prior discussion and agreement with the sponsor.

A maximum of two dose reductions of CT7001 will be allowed per patients. Patients requiring more than 2 dose reductions will be discontinued from the study and entered into the follow-up phase.

In case emerging data may prompt SRC to modify the starting dose in the future, Table 3 describes the recommended dose reductions for three different options.

Table 3: Two Dose Reduction Levels for Three Potential Starting Doses

Starting dose	360 mg OD	240 mg OD	180 mg BID
First dose reduction	300 mg OD	180 mg OD	120 mg in the morning **and** 180 mg in the evening
Second dose reduction	240 mg OD	120 mg OD	120 mg BID

OD = once daily; BID = twice a day (9-12 hours apart)

Table 4 describes the number and strength of capsules to be taken for each possible dose.

Table 4: Number and Strength of Capsules for Each Possible Dose

Dose	120 mg Capsules	60 mg Capsules
360 mg	3	0
300 mg	2	1
240 mg	2	0
180 mg	1	1
120 mg	1	0

All dose modifications/adjustments must be clearly documented in the patient's source notes and Investigational product administration eCRF.

6.8 Inter-Patient Dose Modification

Module 1A is complete. Module 4 has completed recruitment and the single dose cross-over bioavailability part with patients currently ongoing in long term dosing. Module 1B-2 (CRPC) has completed recruitment with patients currently ongoing in long term dosing and Module 2 is currently open to recruitment.

The SRC will meet regularly to review all available data on safety and anti-tumour effects from Module 1B-1 but also all the further data emerging from ongoing Modules. Based on the totality of these data, SRC may determine that the starting dose in Module 1B-1 should be modified in subsequent patients. Modification options include but are not exclusive to lowering the daily CT7001 dose, administration of CT7001 twice a day and the introduction of a treatment holiday. An increase in the total daily dose beyond the initial dose of 360 mg will only be permissible if the particular dose had been properly evaluated in Module 1A and found to be safe. In case inter-subject modification of the CT7001 dosing regimen appears indicated, this would prompt a major protocol amendment.

6.9 Compliance

Patients will be required to return all bottles of CT7001 as well as their completed patient diary at the beginning of each cycle for drug accountability. Drug accountability will be performed on Day 1 of every cycle prior to dispensing drug supply for the next cycle. The number of remaining capsules will be documented and recorded.

6.10 Drug Storage and Accountability

Storage conditions stated in the IB may be superseded by the label storage. Investigators and site staff are reminded to continuously monitor room storage temperatures and ensure that thermometers are working correctly as required for proper storage of the investigational product. Temperature excursions must be reported immediately to the sponsor and documented. Once a deviation is identified, the investigational product (CT7001) MUST be quarantined and not used until the sponsor provides documentation of permission to use the investigational product.

At the end of the trial or at the close-out of the site, any unused investigational product will be destroyed. If the destruction occurs at the trial site, the investigator must ensure that the materials are destroyed in compliance with applicable environmental regulations, institutional policy, and any special instructions provided by the sponsor. Destruction must be adequately documented. Alternatively, investigational product maybe shipped to a local depot for destruction.

To ensure adequate records, CT7001 capsules will be accounted for as instructed by the sponsor. Patients are required to return previously dispensed containers as well as their completed patient diary to the clinic at each visit for accountability purposes even if they will not be issued with new medication at that visit.

6.11 Concomitant Medications

If medically reasonable and feasible, subjects taking regular medication (with the exception of strong inhibitors or inducers of CYP3A4, CYP2C19, CYP2D6, or p-glycoprotein (PGP) (see Appendix B) should be maintained on it throughout the study/module.

Patients must be instructed not to take any new medications (over-the-counter or other products) during the study without prior consultation with the investigator. Medications that are considered necessary for the subject's safety and well-being may be given at the discretion of the Investigator.

Any medications including herbal supplements, vitamins or medicines taken by the patient from 28 days prior to the start of study treatment and up to 28 days following the last dose of investigational product and the reason for their administration must be recorded on the eCRF.

Routine postoperative care, such as dressing changes, suture removal, drain removal, or venous access (central or peripheral), does not need to be recorded. Anaesthetics used for any surgical procedures performed during the patient's participation in the study can be recorded as "unspecified anaesthesia" on the concomitant treatment records; it is not necessary to list the specific anaesthetics.

Appropriate palliative and supportive care for cancer-related symptoms will be offered to all patients in this study.

6.11.1 Prohibited Medications

The following treatments are prohibited throughout the duration of the active treatment phase:

- **Anti-cancer agents**: No additional investigational or commercial anti-cancer agents such as chemotherapy, immunotherapy, targeted therapy, biological response modifiers or endocrine therapy will be permitted during the active treatment phase. In general, any drugs containing "for the treatment of breast cancer" on the product insert are not permitted on study.
    - Patients may receive bisphosphonates or denosumab for the treatment of bone metastases during participation in the study.
- **No investigational product other than CT7001**.
- **Blood transfusions** are not allowed within 14 days before the first IMP dose.
- **Live virus or bacterial vaccines** (e.g., yellow fever, measles, influenza, rubella, mumps, typhoid, mycobacterium tuberculosis [BCG], Yersinia pestis [EV] vaccines).
    - An increased risk of infection by the administration of these vaccines has been observed with conventional chemotherapy and the effects with CT7001 are unknown.
        - Live vaccines must not be administered until 3 months after the last dose of IMP.

        - If the live vaccine induces B-cell depletion then they should not be administered until 6 months after the last dose of IMP (Rubin et al, 2014).
    - Administration of the live flu vaccine is allowed if given at least 28 days prior to start of screening.
    - The administration of killed vaccines (e.g., cholera, bubonic plague, non-live influenza, polio, hepatitis A, and rabies vaccine) is allowed.

6.11.2 Medications Not Recommended

The following treatments are not recommended throughout the duration of the active treatment phase. Alternative therapies should be considered whenever possible. If the investigators deemed usage of the following treatments necessary, consultation and agreement with the sponsor is required prior to initiation of treatment.

- Medications, herbal supplements, and foods that are strong inducers or inhibitors of CYP3A4, CYP2C19, CYP2D6 or PGP (see Appendix B for a listing) should be avoided from 14 days before the first dose of CT7001 until 28 days after the last dose of CT7001.
    - **Note:** St. John's Wort should be avoided from 21 days before the first dose of CT7001.
    - Aprepitant (Emend®) is a substrate, moderate inhibitor and inducer of CYP3A4 and an inducer of CYP2C19. Therefore, aprepitant should not be used in this study for the treatment of nausea and vomiting.
    - If the Investigator feels that concomitant administration of such medications, herbal supplements or foods is necessary based upon medical judgment, such products may be administered with caution following discussion between the Investigator and the Carrick Therapeutics Physician or CRO medical monitor.
        - CT7001 is an investigational drug for which no in vivo data on drug interactions are currently available.
        - Patients taking concomitant medications whose disposition is dependent upon CYP3A4 and which have a narrow therapeutic index should be closely monitored for signs of changed tolerability.
        - CT7001 shows a weak potential to inhibit CYP2D6 and 2C19, which should be considered when co-prescribing 2D6 and 2C19 substrates.
- CT7001 exhibits pH-dependent solubility. As such there is a risk that agents that increase gastric pH (such as PPIs, H2 antagonists) may affect the bioavailability of CT7001 and should be avoided in the study if possible. However, if clinically required, they may be prescribed. Such patients should be monitored for signs of changed CT7001 activity.
- Chronic immunosuppressive therapies should be avoided, including systemic corticosteroids.

  - Steroids given for physiological replacement, as anti-emetics or inhaled as well as short course of oral or topical steroids given for allergic reactions or asthma flares are allowed.

- Drugs known to predispose to Torsades de Pointes should be avoided during the active treatment phase. Refer to Appendix A for a list of such drugs.

- The use of any natural or herbal products or other 'folk remedies' should be discouraged. Cannabinoids / CBD oil are discouraged, if these are used they should be clearly captured in the eCRF.

6.11.3 Permitted and/or Recommended Treatments

The following treatments are permitted throughout the duration of the active treatment phase, with all medications and treatments to be recorded in the eCRF:

- Continuation of therapies for pre-existing medical conditions.
  - This includes bisphosphonates and/or receptor activator of nuclear factor kappa-B ligand (RANKL) inhibitors for the treatment of osteoporosis or management of existing bone metastases, provided patients have been receiving them at a stable dose for at least 2 weeks prior to first dose of CT7001.
    - Please note that the need to initiate, or increase the dose of, these therapies during the study will be considered as indicative of disease progression unless disease progression can be fully ruled out and the exact alternative reason for the use of these therapies is clearly documented in the subject's source documentation.

- Treatments of medical and/or surgical complications.

- All study patients should be offered **best supportive care** as per standard institutional practice and/or most recent guidelines by organizations such as ASCO, the National Comprehensive Cancer Network (NCCN) in the US and/or the European Society of Medical Oncology (ESMO). The below, protocol specific, guidance for the management of nausea and/or vomiting may be amended by the SRC based on emerging data. Any change to the guidance will not be considered a substantial amendment to the protocol.
  - **Anti-emetic medication** should be given prophylactically for nausea (N) and/or vomiting (V) as deemed indicated by the investigator and in accordance with the current DMC guidelines (see Appendix H).
  - **Haematopoietic growth factors**:
    - Primary prophylactic use of granulocyte colony stimulating factor (G-CSF) or granulocyte macrophage colony stimulating factor (GM-CSF) is not permitted but non-pegylated G-CSF may be used to treat treatment-emergent neutropenia when clinically indicated as per standard institutional practice and/or most recent ASCO/NCCN/ESMO guidelines.

                - If neutropenic complications occur in a cycle, secondary prophylaxis may be given at the discretion of the investigator, but only if dose reduction or delay are not considered to be a reasonable alternative.

        - Erythropoietin may be used at the investigator's discretion for the supportive treatment of anaemia.

- **Red blood cell transfusions** may be given as clinically indicated for the treatment of anaemia but should be clearly noted as concurrent medications.

- **Diarrhoea**: In the event of diarrhoea, supportive measures should be initiated promptly. These include the following:

    - At the first sign of loose stools, the patient should initiate anti-diarrheal therapy (e.g., loperamide) and notify the investigator/site for further instructions and appropriate follow-up.

    - Patients should also be encouraged to drink plenty of fluids (e.g., 8 to 10 glasses of clear liquids per day).

    - Site personnel should assess response within 24 hours.

    - If diarrhoea does not resolve with anti-diarrheal therapy within 24 hours to at least Grade 1, CT7001 should be suspended until diarrhoea is resolved to at least Grade 1.

    - In case of Grade ≥3 diarrhoea, CT7001 should be interrupted (with a cycle) or delayed (at start of next cycle). See also Sections 6.7.1.2 and 6.7.1.3.

    - In severe cases of diarrhoea, the measuring of neutrophil counts and body temperature and treatment with antidiarrheal agents should be considered.

    - Antidiarrheal agents should be deferred if blood or mucus is present in the stool or if diarrhoea is accompanied by fever. In these circumstances, appropriate diagnostic microbiologic specimens should be obtained to exclude an infectious aetiology.

    - If diarrhoea is severe (requiring intravenous (IV) rehydration) and/or associated with fever or severe neutropenia, broad-spectrum antibiotics such as fluoroquinolones should be prescribed.

    - Patients with severe diarrhoea or any grade of diarrhoea associated with severe nausea or vomiting should be carefully monitored and given intravenous fluid (IV hydration) and electrolyte replacement.

- **Medication and other measures for pain control** should follow standard institutional practice and/or ASCO/NCCN/ESMO guidelines.

- **Other Medications,** in accordance with local standard of care, will be permitted unless specified otherwise (see prohibited medicines and medicines not recommended).

6.12 Contraception

Women of childbearing potential (for definition of no childbearing potential see Section 4.1, Inclusion Criterion #10) must practice effective contraception. This includes:

- Abstinence if consistently employed
- Sex only with person of the same sex or with vasectomised partner
- Intrauterine device (IUD), or barrier method (e.g., condom, diaphragm) for the duration of the study and for 6 months after the last dose of CT7001.
- **Note** that contraceptives that are prone to drug-drug interactions may not be effective because of a potential CYP3A4 interaction with CT7001.

Sexually active male subjects must be willing to use condoms with all sexual partners for the duration of the study and for 3 months after the last dose of CT7001. If a female partner is a woman of childbearing potential who is not using effective contraception (as defined above), the subject must use a condom with spermicide during the study and for 6 months after the last dose of CT7001.

6.13 Concomitant Radiotherapy or Surgery

Concurrent radiotherapy (except palliative radiotherapy as specified below) or cancer-related surgery are prohibited throughout the duration of the active treatment phase of the study. Patients requiring these procedures will be discontinued from the active treatment phase and will enter the follow-up phase.

Palliative radiotherapy is permitted for the treatment of painful bony lesions provided that the lesions were known to be present at the time of study entry and the investigator clearly documents that the need for palliative radiotherapy is not indicative of disease progression.

In view of the current lack of data about the interaction of CT7001 with radiotherapy, study treatment should be interrupted during palliative radiotherapy, stopping at least 1 day before and resuming treatment no earlier than 1 week after.

For patients with bone involvement, it is suggested to institute palliative radiotherapy before study initiation if possible and clinically appropriate (e.g., lesions at risk for spontaneous micro-fractures or painful lesions).

Palliative radiotherapy during the active treatment phase will be considered alternative cancer therapy and will result in censoring of the PFS endpoint. The dates on which palliative radiotherapy is administered should be recorded on the appropriate eCRFs.

Caution is advised on theoretical grounds for any surgical procedures during the study. The appropriate interval of time between surgery and CT7001 required to minimize the risk of impaired wound healing and bleeding has not been determined. Based on the available pharmacokinetic data, stopping of CT7001 is recommended at least 7 days prior to elective

surgery. Postoperatively, the decision to reinitiate CT7001 treatment should be based on a clinical assessment of satisfactory wound healing and recovery from surgery.

7 STUDY PROCEDURES

Prior to undergoing any study-specific procedures (with the exception of certain imaging assessments if meeting the criteria defined in Section 7.1), patients must read and sign the consent form(s). All study procedures and their timing are detailed in the Schedule of Events (Section 18 SCHEDULE OF ASSESSMENTS, Table 5 and Table 6). All data obtained for these assessments must be supported in the patients' source documentation. For the purpose of this trial, a cycle is defined as 21 days. A cycle could be longer than 21 days if persistent toxicity delays the initiation of the subsequent cycle.

7.1 Screening

Voluntary, written, dated, and signed informed consent MUST be obtained before any study specific procedures are performed (with the exception of certain imaging assessments if meeting the criteria defined in this section).

Radiographic tumour assessments that were performed before the signing of the informed consent form as routine procedures (but within 28 days prior to enrolment in the study = allocation to study treatment) do not need to be repeated and may be used as baseline assessments, as long as:

- The tests were performed per the method requirements described in Section 8.2.
- Appropriate documentation indicating that these radiographic tumour assessments were performed as standard of care is available in the patient's source notes.

Bone scans performed as routine procedures within 8 weeks prior to first administration of CT7001 are also accepted as baseline assessment if they meet the two requirements listed above.

Details on screening procedures are provided in the Schedule of Events (Section 18, Table 5 and Table 6).

7.1.1 Screen Failure

Patients who completed the informed consent process but do NOT meet all eligibility criteria and therefore are NOT enrolled in the study will be considered as screen failures.

Subjects who fails screening may be re-screened only on approval of the SRC and Sponsor. Any subject re-screened will need to provide new informed consent and will be allocated a new study number.

7.2 Active Treatment Phase

For details on procedures during the active treatment phase, see the Schedule of Events Tables (Section 18, Table 5 and Table 6). In the event the start of a new cycle is delayed, procedures required on Day 1 of that cycle will be performed when CT7001 is resumed. Day 1 procedures

(i.e., physical examination, ECOG performance status, ECG, blood chemistry and haematology) that were performed prior to knowing the need to delay the start of the cycle do not need to be repeated if:

- Not required to determine whether study drug may be resumed and
- Performed within 7 days prior to restart of study therapy.

7.3 End of Treatment Visit

The end of treatment visit will be performed as soon as possible but no later than 4 weeks from the last dose of CT7001 and prior to initiation of any new anticancer therapy, whichever occurs first. In the event treatment was discontinued due to death or documented disease progression (Section 8.2), the end of treatment visit represents the final visit.

For details on procedures to be performed at the End of Treatment visit, see the Schedule of Event tables, (Section 18, Table 5 and Table 6)

7.4 Follow-up Visit

Unless study treatment was discontinued due to death or documented disease progression, patients should be followed for progression-free survival until documented disease progression, death, start of post-study cancer therapy or lost to follow-up, whichever occurs first. For details on procedures to be performed see the Schedule of Event tables, (Section 18, Table 5 and Table 6).

7.5 Patient Withdrawal

The term "interruption" refers to a patient stopping the investigational product during the course of the study, but then re-starting it at a later time in the study. The reason for dosing interruption will be collected on the appropriate eCRF.

The term "discontinuation" refers to a patient's withdrawal from the study, which may occur during the active treatment phase or post-treatment. The reason for discontinuation must be collected on the appropriate eCRF.

7.5.1 Active Treatment Phase Discontinuation

Patients may be withdrawn from the active treatment phase in case of:

- Disease progression as per RECIST v.1.1 (Section 8.2)
- Symptomatic deterioration (i.e., global deterioration of health status or requiring discontinuation of treatment without objective evidence of disease progression as per RECIST v.1.1)
- Need for additional anti-cancer therapy not specified in the protocol
- Unacceptable toxicities
- Investigator conclusion that it is in the patient’s best interest to discontinue therapy (e.g., poor compliance with either protocol monitoring or with taking the study medications, etc)
- Lost to follow-up

    - If a patient does not return for a scheduled visit, every effort should be made to contact the patient.
    - If 3 attempts to contact the patient were unsuccessful, one of which is by registered letter, the patient should be considered “lost to follow-up”.
    - Steps taken to contact the patient (e.g., dates of telephone calls, registered letters, etc) must be clearly documented in the source documents.
- Patient choice to withdraw from treatment (follow-up permitted by patient)
- Withdrawal of patient consent (cessation of follow-up)
- Death

Patient who discontinue from the active treatment phase must have end of treatment/withdrawal evaluations performed as soon as possible but no later than 28 ± 7 days from the last dose of investigational product and prior to initiation of any new anti-cancer therapy. Data to be collected at the end of study treatment/withdrawal visit are described in the Schedule of Events Table on Mandatory Procedures, (Section 18, Table 5).

If a patient opts to discontinue from the active treatment phase as a result of an unacceptable adverse drug reaction, "withdrawal of consent" should not recorded as the reason for discontinuation. Instead, the reason for discontinuation of active treatment phase must be recorded as "unacceptable toxicity" on the AE eCRF leading to the patient’s withdrawal of consent.

7.5.2 Post-Treatment Study Discontinuation

After discontinuation of study therapy, patients may withdraw from the study at any time at their own request or they may be withdrawn at any time at the discretion of the investigator or sponsor for safety or behavioural reasons, or the inability of the patient to comply with the protocol required schedule of study visits or procedures at a given study site.

Patients will be withdrawn from study in the case of:

- Withdrawal of consent (i.e., refuses tumour assessments)
- Lost to follow-up (see Section 7.5.1) for activities required before declaring a patient as lost to follow-up)
- Death

In any circumstance, every effort should be made to document patient outcome, if possible. The investigator should inquire about the reason for withdrawal, request the patient to return all unused investigational product(s), request the patient to return for a final visit, if applicable, and follow-up with the patient regarding any unresolved adverse events (AEs).

Data to be collected for the end of study treatment/withdrawal are described in the Schedule of Event table on Mandatory Procedures (Section 18, Table 5).

If a patient withdraws from the study and withdraws consent for disclosure of future information, no further evaluations should be performed, and no additional data should be

collected. The sponsor may retain and continue to use any data collected before such withdrawal of consent.

7.6 Study Completion

Study completion is defined as last patient last visit (i.e., when the last patient has progressed or withdrawn).

8 STUDY ASSESSMENTS

The study procedures are described in the Schedule of Events tables and their footnotes. Every effort should be made to ensure that the required tests and procedures are completed as described. However, it is anticipated that from time to time there may be circumstances, outside of the control of the investigator, that may make it unfeasible to perform the test at all or on schedule. In these cases, the investigator will take all steps necessary to ensure the safety and wellbeing of the patient. When a protocol-required test cannot be performed the investigator will document the reason for this and any corrective and preventive actions which he/she has taken to ensure that normal processes are adhered to as soon as possible. The study team will be informed of such incidents in a timely fashion.

8.1 Safety Assessments

Safety assessment will consist of monitoring of all adverse events (AEs), including serious adverse events (SAEs), regular monitoring of haematology, serum chemistry, triplicate 12-lead ECGs, physical examinations, vital signs and ECOG performance status.

Adverse event assessment will include type, incidence, severity (graded by the National Cancer Institute Common Terminology Criteria for Adverse Events [NCI CTCAE], Version 5.0, see Severity Assessment, Section 9.6), timing, seriousness, and relatedness. Baseline tumour-related signs and symptoms will be recorded at the Cycle 1 Day 1 visit and then reported as adverse events during the trial if they worsen in severity or increase in frequency.

8.1.1 Laboratory Safety Assessments

Laboratory tests will include full blood counts, standard serum chemistry and urinalysis.

Full blood counts include: red blood cell count, haematocrit, mean cell volume, reticulocyte count (absolute particle count or relative particle count), white blood cell count with differential (absolute and percent counts of neutrophils, lymphocytes, monocytes, basophils, eosinophils) and platelet count.

Serum chemistry includes: HbA1c, ALT, AST, gamma glutamyl transferase (IU/L), alkaline phosphatase, bilirubin (total), creatine kinase, total protein, albumin, creatinine, urea nitrogen or urea, calcium (total), glucose, sodium, potassium, magnesium, chloride and phosphate.

Urinalysis includes: blood, glucose and protein.

All results will be entered in the eCRF, with Système International (SI) units as standard system of measurements. However, the eCRF will allow reporting of a subset of laboratory tests in conventional units if participating study sites consider this helpful.

They will be assessed during screening within four weeks before enrolment, weekly in the first two cycles and then approximately every three weeks in subsequent cycles, and at the end of treatment and end of study visits. Laboratory tests do not need to be repeated on Day 1 of Cycle 1 if performed within 7 days before the date of treatment assignment. Please also refer to Section 18, Table 5 for Mandatory Procedures. Additional blood tests may be performed at the investigator's discretion as clinically indicated for the purpose of planning treatment administration, dose modification, or following adverse events. If specific tumour markers are followed locally, these will be captured in the eCRF.

8.1.2 Electrocardiogram (ECG)

ECGs will be performed using a12-lead tracing. ECG measurements will include heart rate, PR interval, QRS complex, QT interval, and QTcF. ECG interval readings by the ECG recorder’s algorithm will be read and interpreted at the investigational site for eligibility determination and patient safety monitoring and documentation stored in the source documents.

Triplicate 12-lead ECGs will be performed during screening within four weeks before enrolment, weekly in the first cycle and then approximately every three weeks in all subsequent cycles, and at the end of treatment and end of study visits. Triplicate ECGs do not need to be repeated on Day 1 of Cycle 1 if performed within 7 days of the date of treatment assignment. Additional ECGs may be performed as clinically indicated at any time.

Triplicate 12-lead ECGs will be performed 3-5 minutes apart.

If at any time during treatment the mean QTc is prolonged ≥501 msec on at least two separate ECGs (i.e., CTCAE ≥ Grade 3), then the ECGs should be re-evaluated by a qualified person at the site for confirmation as soon as the finding is made, including verification that the machine reading is accurate. If manual reading confirms a QTc of ≥ 501 msec, an immediate search for reversible causes should be performed (including electrolyte abnormalities, hypoxia and concomitant medications for drugs with the potential to prolong the QTc interval). In addition, repeat ECGs should be performed hourly for at least 3 hours until the QTc interval falls below 501 msec.

Sections 6.7.1.2 and 6.7.1.3 provide instructions on dosing interruptions/delays and restart in the event of QTc prolongation of ≥ 501 msec.

8.1.3 Other Safety Assessments

A full physical examination and assessment of vital signs and performance status will be required at screening, weekly in the first 2 cycles and then on Day 1 in all subsequent cycles, and at the end of treatment and end of study visits.

A full physical examination includes examination of all major body systems, height (at screening only) and weight.

Vital signs include supine blood pressure, pulse rate, temperature and respiratory rate.

Performance status will be assessed according to ECOG performance status scale (see Appendix F).

8.2 Tumour Assessments

Tumour assessments should be performed as scheduled, regardless of treatment interruptions or cycle delays.

Overall tumour response and individual tumour response and will be recorded at each post-baseline visit.

Anti-tumour activity will be assessed locally by the Investigator. The Sponsor reserves the right to request an anonymised independent review of subject scan data if required.

Screening/baseline tumour assessment will be carried out within 28 days before Cycle 1 Day 1. Disease assessment at baseline will include:

- CT or magnetic resonance imaging (MRI) scan of the chest, abdomen, and pelvis.
- CT or MRI scan of any other sites of disease as clinically indicated.
- Clinical assessment of superficial disease which will include photographs of all superficial metastatic lesions.
- Bone scans to detect sites of bone metastasis.
  - Any suspicious abnormalities (i.e., hotspots) identified on the bone scans at baseline must be confirmed by X-ray, CT scan with bone windows or MRI.
- All lesion measurements must be recorded in the eCRF.
- Baseline brain CT or MRI are only required in case signs and symptoms suggest the presence of metastatic brain disease. Refer to Section 18, Table 5 for further details on timing allowance for baseline brain and bone scans.

Post-baseline tumour assessments by CT or MRI scans will be performed every 8 weeks (± 7 days) from Cycle 1 Day 1. Bone scans (as applicable) will be repeated every 16 weeks (±7 days). Tumour assessments will continue until radiographically and/or clinically (i.e., for photographed or palpable lesions) documented PD as per RECIST v.1.1*, death, discontinuation of patient from overall study participation (e.g., patient's request, lost to follow-up) or initiation of new anticancer therapy, whichever occurs first.

*At the time of first documented RECIST disease progression, if the investigator and patient consider the patient is still benefitting from study treatment, dosing may continue until the disease progression is confirmed at the next scheduled imaging evaluation. If at the subsequent scan the progression is confirmed study treatment should end, if not confirmed the patient may remain on study treatment.

Imaging assessments are to be scheduled using Cycle 1 Day 1 as the reference date and are NOT to be scheduled based on the date of the previous imaging time-point. Imaging assessment delay to conform to treatment delay is not permitted.

Every effort should be made to perform a last tumour assessment before starting a new anticancer therapy. Additional unscheduled tumour assessments may be performed as clinically indicated at any time.

<u>Post-baseline tumour assessments will include</u>:

- CT or MRI scan of the chest, abdomen, and pelvis, every 8 weeks (± 7 days) for the first year, and then every 12 weeks (±7 days) in subsequent years (calculated from the date of Cycle 1 Day 1) until documented progression*, death or onset of new anticancer therapy, whichever occurs first.
- CT or MRI scan of any other sites of disease identified at baseline, every 8 weeks (± 7 days) for the first year, and then every 12 weeks (±7 days) in subsequent years (calculated from the date of Cycle 1 Day 1) until documented progression*, death or onset of new anticancer therapy, whichever occurs first.
- Clinical assessment of sites of superficial disease identified at baseline, every 8 weeks (± 7 days) for the first year, and then every 12 weeks (±7 days) in subsequent years (calculated from the date of Cycle 1 Day 1) until documented progression*, death or onset of new anticancer therapy, whichever occurs first.
    - Clinical assessment of superficial disease must coincide with the imaging studies and will include photographs of all superficial metastatic lesions.
- Imaging of bone lesions:
    - If bone lesions were identified at baseline the following assessment must be performed:
        - Bone scans every 16 weeks (± 7 days) from the Cycle 1 Day 1 and to confirm complete response. Abnormalities found on subsequent bone scans must also be confirmed by X-ray, CT scan, or MRI.
        - X-ray/CT scan/MRI every 8 weeks (± 7 days) from Cycle 1 Day 1 using the same modality used to confirm the bone lesions at baseline, for the first year, and then every 12 weeks (±7 days) in subsequent years (calculated from the date of Cycle 1 Day 1) until documented progression*, death or onset of new anticancer therapy, whichever occurs first. Areas that have received palliative radiotherapy on study cannot be used to assess response to study treatment.
    - If no bone lesions were identified at baseline, bone scans should be performed as clinically indicated (i.e., patient describes new or worsening bone pain, or has increasing alkaline phosphatase level or other signs and symptoms of new/progressing bone metastases) but are required to confirm complete response. Abnormalities found on subsequent bone scans must also be confirmed by X-ray, CT scan, or MRI.
- Repeat brain scans will be required only if metastases are suspected.

The CT scans, including brain CT scan if applicable, should be performed with contrast agents unless contraindicated for medical reasons. If IV contrast is medically contraindicated, the imaging modality to be used to follow the disease (either CT without contrast or MRI) should be the modality which best evaluates the disease, and the choice should be determined by the investigator in conjunction with the local radiologist. MRI of the abdomen and pelvis can be

substituted for CT if MRI adequately depicts the disease. However, MRI of the chest should not be substituted for CT of chest even if IV contrast is contraindicated. In such case CT will be performed without contrast. If MRI is used to follow-up bone lesion(s) it must be performed a few days before any treatment that may affect bone-marrow cellularity (e.g., G-CSF).

The same method and technique should be used to characterize each lesion identified and reported at baseline, during the study treatment period and during follow-up. The use of plain-film X-rays (with the exception of bone X-rays as detailed above) is not permitted. The use of positron emission tomography (PET) imaging as the only imaging modality is not permitted.

For patients having effusions or ascites, cases having cytological proof of malignancy should be recorded as non-target lesions on the tumour assessment eCRFs. Effusions that have not been evaluated using cytology or were found to be non-malignant should not be recorded on the non-target lesion eCRF.

Objective tumour response will be measured using the RECIST Version 1.1, (see http://recist.eortc.org/recist-1-1-2/). Please also refer to Appendix C. All measurements should be recorded in metric notation.

8.3 Pharmacokinetic Assessments

Blood samples for PK trough analysis of CT7001 will be collected before dosing on Day 1 and Day 8 of Cycle 1, Day one in all subsequent Cycles and at the end of study visit (see also Section 18, Table 5). All efforts should be made to obtain the pharmacokinetic samples at the scheduled nominal times and within the time window relative to dosing. Additional blood samples may be requested from patients experiencing unexpected or serious adverse events, or adverse events that lead to discontinuation.

The exact time of the sample collection and the most recent dosing time will be recorded on the eCRF. The date of missing doses should also be recorded in the eCRF.

As part of understanding the PK behaviour and profile of CT7001, part of the blood samples may be used for metabolite identification. These data will be used for internal exploratory purposes and will not be included in the Clinical Study Report.

The concentration of CT7001 and metabolites, if applicable, in PK samples will be quantified by liquid chromatography with tandem mass spectrometry.

Refer to the Laboratory Manual for detailed collection, processing and shipping procedures.

8.4 Biomarker Assessments

ER, PgR and HER2

During screening, assessment of negative expression of ER, PgR and HER2 in breast carcinomas from study patients will be based on results from local pathology laboratories. No independent central review is intended. Negative assessment for ER, PgR and HER2 by local laboratories should be consistent with the criteria in the most recent versions of ASCO/CAP guidelines for the hormone receptors and HER2, respectively (Hammond et al, 2010, Wolff et al, 2018). Please see Section 4.1 for definition of specific criteria.

Assessment of CYP and drug transporter genes

CYP2D6 is the main P450 isozyme involved in Phase I metabolism of CT7001 but other members of the CYP family also play a role. Drug transporters may also affect the bioavailability of CT7001. Therefore, a blood sample will be collected before dosing on Day 1 to analyse polymorphisms and copy number variations of CYP and drug transporter genes. The racial and ethnic distribution among study patients will (co-)inform which alleles to be genotyped.

Exploratory Biomarkers

The study includes blood samples for various assessments to further explore the effects of CT7001 on gene expression in normal cells and tumour tissue and the possible effect of mutations and expression in certain genes, RNAs and proteins in tumour-derived materials on sensitivity and/or resistant to CT7001.

Blood samples for PBMC collection and analysis will be used to explore possible changes in RNA expression of a panel of genes which nonclinical data suggest are affected by inhibition of CDK7 (e.g., c-Myc, MCL-1).

Various patient and tumour-derived materials will be collected to further explore the effect of mutations and expression in certain genes, RNAs and proteins on sensitivity and/or resistant to CT7001. The tumour-derived materials include formalin-fixed paraffin embedded (FFPE) and/or fresh frozen tumour tissue obtained through biopsy, circulating tumour DNA (ctDNA) and circulating tumour cells (CTC). A panel of genes, proteins and RNAs relevant to the cell cycle, drug target engagement and/or sensitivity/resistance will be analysed, including phosphorylated CDK1, RNA polymerase II and Rb proteins, c-Myc, MCL-1, p53, CDK7, ER and AR.

With the exception of ER, PgR and HER2 expression in the patient’s breast cancer, all biomarker analyses and related procedures are optional and require specific written informed consent from a patient. Consent will be requested/obtained separately for peripheral blood samples versus tumour biopsies, and for retained samples for pharmacogenomics.

Patients who elect not to allow any or all optional procedures may still participate in the clinical trial.

Blood Samples for RNA-Sequence Analysis are only to be collected from patients who have consented to fresh tumour biopsies. These samples will only be collected on Day 1 of Cycle 1 before and 4 hours after dosing; and before dosing on Day 1 of Cycle 2.

In the event patient consent was obtained, peripheral blood samples for various biomarker assessments will be collected at Screening, on Days 1 of Cycles 1, 2 and 3 and from every subsequent ‘odd’ cycle thereafter and at the time of documented disease progression. Collection times will be entered in the eCRF. This consent is independent of consent for biopsy samples.

Tumour Biopsies

For patients with readily accessible lesions who have provided consent, serial tumour biopsies are encouraged to be obtained at baseline (within one week before first dose), 6 weeks ± 7 days

from start of study therapy and within two weeks after documentation of objective disease progression. Collection times will be entered in the eCRF.

An accessible lesion is defined as a tumour lesion that is amenable to repeat biopsy, unless clinically contraindicated or the patient has withdrawn consent. Failure to obtain sufficient tumour sample after making best efforts to biopsy the tumour will not be considered a protocol deviation.

Where possible, two biopsy cores should be collected per scheduled timepoint. In the obtained biopsy material, first priority will be given to FFPE for immunohistochemistry (CDK7, pPolII, c-Myc, pCDK1 and Ki67/Caspase) and mRNA expression analysis. In case of a second biopsy core being taken, it should be stored in RNA later for ChIP-Sequence analysis. Failure to collect a second biopsy core will not be considered a protocol deviation.

Archival Tumour Tissue

An archival FFPE tumour tissue sample, if available, will be requested for each subject. Even if fresh biopsy samples can be collected, retrieval of the archival diagnostic tumour material is still highly encouraged to provide data on how the tumour has evolved since diagnosis. Archival samples from either primary or metastatic tumour will be accepted, but tissue from the primary tumour is preferred. For a subject who has archival tissue samples from multiple time points tissue from the most recent biopsy is preferred.

Tumour tissue blocks are preferred, but freshly prepared unstained slides (minimum 10, preferably 20) with 5 micron sections from the archival tumour block are accepted if tumour blocks cannot be submitted.

Retained Samples for Future Exploratory Research

CT7001 is early in its clinical development. The science of CDK7 and TNBC is rapidly evolving and so are the data of CT7001. As a result, by the end of the study and/or in the future new biomarker hypotheses might be important to investigate. Provided specific written informed consent was obtained from the patient, blood samples for potential future exploratory research will be collected before dosing on Day 1 of Cycles 1, 2 and 3 and from every subsequent 'odd' cycle thereafter and at the time of documented disease progression.

Retained Sample for Pharmacogenomics

Genomic and metabolomic variation may help to explain some of the variability in response seen with some drugs among different individuals. This is referred to as pharmacogenomics. Comparing the DNA, RNA, protein, and metabolite variation patterns of patients who respond well and those who respond poorly to treatment may help to better define the most appropriate group of patients in which to target a given treatment. Collecting samples for pharmacogenomic analyses and retaining them makes it possible to seek explanations for differences in, for example, exposure, efficacy, tolerability, or safety not anticipated prior to the beginning of the study. For example, putative safety biomarkers, drug metabolizing enzyme genes, drug transport protein genes, or genes thought to be related to the mechanism of drug action may be examined.

Provided specific written informed consent was obtained from the patient and not prohibited by local regulations, a blood sample for potential future pharmacogenomic testing will be taken before dosing on Day 1 of the first Cycle.

Additional Pharmacogenomic Research

Unless prohibited by local regulations, patients will be asked to indicate on the consent form whether they will allow the Retained Pharmacogenomic Sample to also be used for the following research:

- Investigations of the disease under study in the clinical trial, and related conditions.
- Use of sample as control. This includes use in case-control studies of diseases for which Carrick is researching drug therapies; use in characterizing the natural variation amongst people in genes, RNA, proteins, and metabolites; and use in developing new technologies related to pharmacogenomics.
- Patients NEED NOT provide additional samples for the uses described in Additional Pharmacogenomics Research. The retained sample for pharmacogenomics will be used.

The blood sample will be collected before dosing on Day 1 of Cycle 1.

General collection and processing instructions are provided in the Laboratory Manual. Collection times have to be entered in the eCRF.

Please also refer to Table 6 on Schedule of Events for Optional Procedures for the various types of optional procedures and tests, and their sampling schedule.

Biological samples collected for this study will become the property of Carrick Therapeutics and may be used for future research conducted by or on behalf of Carrick or its affiliates, partners, or collaborators. No identifiable personal information will be associated with these blood samples. Any remaining samples will be destroyed no later than 15 years after the end of the study.

9 ADVERSE EVENT REPORTING

All observed adverse events (AEs) regardless of suspected causal relationship to the investigational product will be reported as described in the following sections. For all AEs, the investigator must pursue and obtain information adequate both to determine the outcome of the AE and to assess whether it meets the criteria for classification as an adverse event of special interest (AESI) or SAE requiring immediate notification to the sponsor or its designated representative (’the sponsor’).

For all AEs, sufficient information should be obtained by the investigator to determine the causality of the AE. The investigator is required to assess causality.

Follow-up by the investigator may be required until the event or its sequelae resolve or stabilize at a level acceptable to the investigator, and the sponsor concurs with that assessment.

As part of ongoing safety reviews conducted by the sponsor, any non-serious AE that is determined by the sponsor to be serious will be reported by the sponsor as an SAE. To assist in the determination of case seriousness further information may be requested from the investigator to provide clarity and understanding of the event in the context of the clinical study.

All AEs spontaneously reported by the subject, reported in response to an open question from the study personnel (e.g., ‘Have you had any health problems since the previous visit/you were last asked?’), or revealed by observation will be recorded in the eCRF.

When recording AEs, the diagnosis is preferred (when possible) to a list of signs and symptoms. However, if a diagnosis is known and there are other signs or symptoms that are not generally part of the diagnosis, the diagnosis and each sign or symptom should be recorded separately.

If no diagnosis, disease or syndrome can be recognized, signs or symptoms should be described in the study subject’s own words (verbatim) unless, in the opinion of the investigator, clarification of the subject’s verbatim language is deemed necessary.

The AE term will subsequently be coded using MedDRA.

The AE term, date of AE onset, date of AE resolution (if applicable), seriousness, severity, causality, action taken for the AE, and outcome will be recorded in the eCRF.

Medical conditions that exist before signing the informed consent form will be recorded as part of medical history.

9.1 Reporting Period

For SAEs, the active reporting period to the sponsor begins from the time the patient provides informed consent, which is obtained prior to the patient’s participation in the study, i.e., prior to undergoing any study-related procedure and/or receiving the investigational product, through the end of study visit (28-35 calendar days after the last administration of the investigational product). SAEs occurring to a subject after the active reporting period has ended should be reported to the sponsor if the investigator becomes aware of them; at a minimum, all SAEs that the investigator believes have at least a reasonable possibility of being related to the investigational product are to be reported to the sponsor.

Death must be reported if it occurs during the SAE reporting period after the last dose of investigational product, irrespective of any intervening treatment.

For AESIs, the active reporting period to the sponsor begins from the time the patient receives their first dose of investigational product, through the end of study visit (28-35 calendar days after the last administration of the investigational product). AESIs occurring to a patient after the active reporting period has ended should be reported to the sponsor if the investigator becomes aware of them and if the investigator believes they have at least a reasonable possibility of being related to the investigational product.

AEs (non-serious) should be recorded on the eCRF from the time the patient has taken at least one dose of investigational product through the patient’s last visit. If a patient begins a new anti-cancer therapy, the AE reporting period for non-serious AEs ends at the time the new treatment is started.

9.2 Definition of Adverse Event

For purpose of this study, an AE is any untoward medical occurrence in a patient who participates in this study. The event need not necessarily have a causal relationship with the investigational product or procedure. Examples of AEs include but are not limited to:

- Clinically significant symptoms and signs (including abnormal laboratory findings)
- Changes in physical examination findings
- Hypersensitivity
- Drug abuse
- Drug dependency

Additionally, they may include the signs or symptoms resulting from

- Drug overdose
- Drug withdrawal
- Drug misuse
- Drug interactions
- Exposure during pregnancy
- Exposure via breast feeding
- Medication error
- Occupational exposure.

Worsening of signs and symptoms of the malignancy under study should be reported as AEs in the appropriate section of the eCRF. Disease progression assessed by measurement of malignant lesions on radiographs or other methods should not be reported as AEs.

9.3 Definition of Adverse Event of Special Interest

For purpose of this study, an AESI is any untoward medical occurrence in a patient, serious or non-serious, who participates in this study that the Sponsor has identified to be notified of immediately. The event need not necessarily have a causal relationship with the investigational product.

AESI are required to be reported by the investigator to the Sponsor immediately (i.e., no more than 24 hours after learning of the event; see Section 9.12.1 for reporting instructions).

Adverse events of special interest for this study are the following:

- Thrombocytopenia Grade ≥ 3 (platelet count < 50 x 10^9/L)

9.4 Definition of Serious Adverse Events

An SAE is any untoward medical occurrence at any dose that:

- Results in death
- Is life-threatening (immediate risk of death)
- Requires inpatient hospitalization or prolongation of existing hospitalization
- Results in persistent or significant disability/incapacity (substantial disruption of the ability to conduct normal life functions)
- Results in congenital anomaly/birth defect
- Is an important medical event that may jeopardise the subject or may require medical intervention to prevent one of the outcomes listed above

Medical and scientific judgment should be exercised in determining whether an event is an important medical event. An important medical event may not be immediately life-threatening and/or result in death or hospitalization. However, if it is determined that the event may jeopardize the subject or may require intervention to prevent one of the other AE outcomes, the important medical event should be reported as serious. Examples of such events are intensive treatment in an emergency room or at home for allergic bronchospasm; blood dyscrasias or convulsions that do not result in hospitalization; or development of drug dependency or drug abuse.

Progression of the malignancy under study (including signs and symptoms of progression) should not be reported as an SAE unless the outcome is fatal within the safety reporting period. If the malignancy has a fatal outcome during the study or within the safety reporting period, then the event leading to death must be recorded as an AE and as an SAE with CTCAE Grade 5.

For fatal progression of malignancy cases, please record the progression of the underlying malignancy as an SAE. If a specific event can be attributed to the death, please also include the event term. For example, if the patient has a fatal pneumonia due to progression of rectum cancer, please record events ‘pneumonia’ and ‘progression of rectum cancer’

Hospitalization due to signs and symptoms of disease progression should not be reported as an SAE.

When reporting a SAE, the following questions should be considered and included in the description if applicable:

- Is it of common occurrence in the population under study?
- Was it “treatment-emergent”?
- Did it respond to de-challenge?
- Did it recur on re-challenge?
- Were there concomitant medications?

- Were pertinent laboratory and/or other tests done?
- Was there an obvious alternative cause?

9.4.1 Protocol-Specified Serious Adverse Events

There are no protocol-specified SAEs in this study. All SAEs will be reported by the investigator as described in previous sections and will be handled as SAEs in the safety database.

9.4.2 Potential Cases of Drug-Induced Liver Injury (Hy's Law Cases)

Abnormal values in aspartate aminotransferase (AST) and/or alanine aminotransferase (ALT) levels concurrent with abnormal elevations in total bilirubin level that meet the criteria outlined below in the absence of other causes of liver injury are considered potential cases of drug-induced liver injury (potential Hy's Law cases) and should always be considered important medical events. Please refer to Appendix E for further details of actions required in cases of Hy's Law.

The threshold of laboratory abnormalities for a potential case of drug-induced liver injury depends on the patient's individual baseline values and underlying conditions. Patients who present with the following laboratory abnormalities should be evaluated further to definitively determine the aetiology of the abnormal laboratory values:

- Patients with AST or ALT and total bilirubin baseline values within the normal range who subsequently present with AST or ALT values ≥3 X ULN concurrent with a total bilirubin value ≥2 X ULN with no evidence of haemolysis and an alkaline phosphatase value ≤2 X ULN or not available.
- For patients with pre-existing ALT or AST or total bilirubin values above the upper limit of normal, the following threshold values should be used in the definition mentioned above:
    - For pre-existing AST **or** ALT baseline values above the normal range: AST or ALT values ≥2 times the baseline values and ≥3 X ULN or ≥8 X ULN (whichever is smaller)

      concurrent with

    - For patients with pre-existing values of total bilirubin above the normal range: Total bilirubin level increased from baseline by an amount of at least one time the upper limit of normal **or** if the value reaches ≥3 times the upper limit of normal (whichever is smaller).

The patient should return to the investigational site and be evaluated as soon as possible, preferably within 48 hours from awareness of the abnormal results. This evaluation should include laboratory tests, detailed history and physical assessment, and the possibility of hepatic neoplasia (primary or secondary) should be considered.

In addition to repeating measurements of AST and ALT, laboratory tests should include albumin, creatine kinase, total bilirubin, direct and indirect bilirubin, gamma-glutamyl transferase, prothrombin time (PT)/international normalised ratio (INR), and alkaline phosphatase. A detailed history, including relevant information, such as review of ethanol,

acetaminophen, recreational drug and supplement consumption, family history, occupational exposure, sexual history, travel history, history of contact with a jaundiced person, surgery, blood transfusion, history of liver or allergic disease, and work exposure, should be collected. Testing for acute hepatitis A, B, or C infection and liver imaging (e.g., biliary tract) may be warranted. All cases confirmed on repeat testing as meeting the laboratory criteria defined above, with no other cause for liver function test (LFT) abnormalities identified at the time should be considered potential Hy's Law cases irrespective of availability of all the results of the investigations performed to determine aetiology of the abnormal LFTs. Such potential Hy's Law cases should be reported as SAEs.

9.5 Hospitalization

Hospitalization is defined as any initial admission (even less than 24 hours) in a hospital or equivalent healthcare facility or any prolongation of an existing admission. Admission also includes transfer within the hospital to an acute/intensive care unit (e.g., from the psychiatric wing to a medical floor, or medical floor to a coronary care unit). An emergency room visit does not necessarily constitute a hospitalization; however, the event leading to the emergency room visit should be assessed for medical importance.

Hospitalization does not include the following:

- Rehabilitation facilities
- Hospice facilities
- Respite care (e.g., caregiver relief)
- Skilled nursing facilities
- Nursing homes
- Same day surgeries (as outpatient/same day/ambulatory procedures).

Hospitalization or prolongation of hospitalization in the absence of a precipitating, clinical AE is not in itself an SAE. Examples include:

- Admission for treatment of a pre-existing condition not associated with the development of a new AE or with a worsening of the pre-existing condition (e.g., for work-up of persistent pre-treatment lab abnormality)
- Social admission (e.g., patient has no place to sleep)
- Administrative admission (e.g., for yearly physical examination)
- Protocol-specified admission during a study (e.g., for a procedure required by the study protocol)
- Optional admission not associated with a precipitating clinical AE (e.g., for elective cosmetic surgery)
- Hospitalization for observation without a medical AE
- Pre-planned treatments or surgical procedures.

    - These should be noted in the baseline documentation for the entire protocol and/or for the individual patient.

- Admission exclusively for the administration of blood products.

Diagnostic and therapeutic non-invasive and invasive procedures, such as surgery, should not be reported as AEs. However, the medical condition for which the procedure was performed should be reported if it meets the definition of an AE. For example, an acute appendicitis that begins during the AE reporting period should be reported as the AE, and the resulting appendectomy should be recorded as treatment of the AE.

9.6 Severity Assessment

As required on the AE eCRFs, the investigator will report adverse events using concise medical terminology (verbatim) as well as collect on the eCRF the appropriate Common Terminology Criteria for Adverse Events (CTCAE) (version 5.0, publication date: November 27, 2017; https://ctep.cancer.gov/protocolDevelopment/electronic_applications/docs/CTCAE_v5_Quick_Reference_8.5x11.pdf) and will use the following definitions of severity to describe the maximum intensity of the adverse event.

Grade	Clinical Description of Severity
1	MILD; asymptomatic or mild symptoms; clinical or diagnostic observations only; intervention not indicated.
2	MODERATE; minimal, local or non-invasive intervention indicated; limiting age-appropriate activities of daily living (ADL).
3	SEVERE or medically significant but not immediately life-threatening; hospitalization or prolongation of hospitalization indicated; disabling; limiting self-care ADL.
4	LIFE-THREATENING consequences; urgent intervention indicated
5	DEATH

Note the distinction between the severity and the seriousness of an AE. A severe event is not necessarily an SAE. For example, a headache may be severe (interferes significantly with the patient's usual function) but would not be classified as serious unless it met one of the criteria for SAEs listed above.

9.7 Causality Assessment

The investigator’s assessment of causality must be provided for all AEs (serious and non-serious). The investigator must record the causal relationship in the eCRF, as appropriate, and report such an assessment in accordance with the serious adverse reporting requirements if applicable.

An investigator’s causality assessment is the determination of whether there exists a reasonable possibility that the investigational product caused or contributed to an AE. Generally, the facts (evidence) or arguments to suggest a causal relationship should be provided. If the investigator does not know whether or not the investigational product caused the event, then the event will be handled as “related to investigational product” for reporting purposes. If the investigator's

causality assessment is "unknown but not related to investigational product", this should be clearly documented on study records.

In addition, if the investigator determines an SAE is associated with study procedures, the investigator must record this causal relationship in the source documents and eCRF, as appropriate, and report such an assessment in accordance with the SAE reporting requirements.

9.8 Exposure During Pregnancy

An exposure during pregnancy occurs if:

- A female becomes, or is found to be, pregnant either while receiving or having been exposed (e.g., because of treatment or environmental exposure) to the investigational product; or the female becomes, or is found to be pregnant after discontinuing and/or being exposed to the investigational product
    - An example of environmental exposure would be a case involving direct contact with the investigational product in a pregnant woman (e.g., a nurse reports that she is pregnant and has been exposed to CT7001).

  A male has either received or been exposed (e.g. because of environmental exposure) to the investigational product prior to or around the time of conception and/or is exposed during his partner's pregnancy.

  If a study patient or their partner becomes or is found to be pregnant during the study patient's treatment with the investigational product, the investigator must submit this information to the sponsor, regardless of whether an SAE has occurred. This must be done irrespective of whether an AE has occurred and within 24 hours of awareness of the exposure. The information submitted should include the anticipated date of delivery. Follow-up is conducted to obtain general information on the pregnancy and its outcome for all reports with an unknown outcome. The investigator will follow the pregnancy until completion (or until pregnancy termination) and notify the sponsor of the outcome as a follow up to the initial report. In the case of a live birth, the structural integrity of the neonate can be assessed at the time of birth. In the event of a termination, the reason(s) for termination should be specified and, if clinically possible, the structural integrity of the terminated foetus should be assessed by gross visual inspection (unless pre-procedure test findings are conclusive for a congenital anomaly and the findings are reported). If the outcome of the pregnancy meets the criteria for an SAE (i.e., ectopic pregnancy, spontaneous abortion, intrauterine foetal demise, neonatal death, or congenital anomaly [in a live born baby, a terminated foetus, an intrauterine foetal demise, or a neonatal death]), the investigator should follow the procedures for reporting SAEs.

9.9 Occupational Exposure

An occupational exposure occurs when during the performance of job duties, a person (whether a healthcare professional or otherwise) gets in unplanned direct contact with the investigational product, which may or may not lead to the occurrence of an adverse event. An occupational exposure is reported to the sponsor within 24hours of the investigator's awareness, using the SAE Report form, regardless of whether there is an associated AE/SAE. Since the information

does not pertain to a subject enrolled in the study, the information is not reported on an eCRF. However, a copy of the completed SAE Report form is maintained in the investigator site file.

9.10 Withdrawal Due to Adverse Events (See Also Section on Patient Withdrawal)

Withdrawal due to AEs should be distinguished from withdrawal due to other causes, according to the definition of AE noted earlier, and recorded on the appropriate AE eCRF page. When a patient withdraws because of an AESI or SAE, the AESI or SAE must be reported in accordance with the reporting requirements defined below.

9.11 Eliciting Adverse Event Information

The Investigator is to report all directly observed AEs and all AEs spontaneously reported by the study patient. In addition, each study patient will be questioned about AEs.

9.12 Reporting Requirements

Each AE is to be assessed to determine if it meets the criteria for AESI or SAEs. If an AESI or SAE occurs, expedited reporting will follow local and international regulations, as appropriate.

9.12.1 Serious Adverse Event Reporting Requirements

All SAEs will be reported from informed consent until the End of Study visit. All AESI will be reported from the first date of investigational product. An AESI/SAE that occurs after the End of Study visit and comes to the attention of the Investigator must be reported only if there is (in the opinion of the investigator) a reasonable causal relationship with the study drug.

AESI/SAEs must be reported to the sponsor's representative (Bionical Emas) within 24 hours of becoming aware of the event. If the SAE is fatal or life-threatening, notification to Bionical Emas must be made immediately, irrespective of the extent of available AE information.

- This is achieved by completing the SAE Report form and sending it to Bionical Emas by email or fax with the Bionical Emas Medical monitor in copy:

**SAE CONTACT DETAILS:**	Bionical Emas<br>Fax: +44 (0)1462 600456<br>Email: drug.safety@bionical-emas.com
Bionical Emas Medical Monitor	Nayana Ghodki<br>Email: nayana.ghodki@bionical-emas.com
Bionical Emas Medical Monitor (back-up)	Jonathan Whitton<br>Email: jonathan.whitton@bionical-emas.com

The Bionical Emas pharmacovigilance (PV) department, in close association with the Medical Monitor, will report applicable SAEs to the regulatory authorities within the legally required timeframe.

After review of an AESI/SAE report by the Bionical Emas PV department and/or the Medical Monitor, additional information may be requested (e.g., clinic or hospital records or procedure reports) to complete the report. If at the time the Investigator initially reports an AESI/SAE the event has not resolved, the Investigator must provide a follow-up report to the Bionical Emas PV department as soon as it resolves (or upon receipt of significant information if the event is still ongoing).

The 24 hour's time window for reporting also applies to additional new information (follow-up) on previously forwarded AESI/SAE reports as well as to the initial and follow-up reporting of exposure during pregnancy, exposure via breastfeeding and occupational exposure cases. In the event the investigator does not become aware of the occurrence of an AESI/SAE immediately (e.g., if an outpatient study patient initially seeks treatment elsewhere), the investigator is to report the event within 24 hours after learning of it and document the time of his/her first awareness of the AE.

For all AESI/SAEs, the investigator is obligated to pursue and provide information to Bionical Emas in accordance with the reporting timeframe specified above. In addition, an investigator may be requested by Bionical Emas and/or the sponsor to obtain specific additional follow-up information in an expedited fashion. This information collected for AESI/SAEs is more detailed than that captured on the AE eCRF. In general, this will include a description of the AE in sufficient detail to allow for a complete medical assessment of the case and independent determination of possible causality. Information on other possible causes of the event, such as concomitant medications, vaccines and/or illnesses must be provided. In the case of a patient's death, a summary of available autopsy findings must be submitted as soon as possible to the sponsor or its designated representative.

9.12.2 Non-Serious Adverse Event Reporting Requirements

All AEs will be reported on the AE page(s) of the eCRF. It should be noted that the form for collection of SAE information is not the same as the AE eCRF. Where the same data are collected, the forms must be completed in a consistent manner. For example, the same AE term should be used on both forms. AEs should be reported using concise medical terminology on the eCRFs as well as on the form for collection of SAE information.

9.12.3 Sponsor's Reporting Requirements to Regulatory Authorities

Adverse event reporting, including suspected unexpected serious adverse reactions (SUSARs), will be carried out in accordance with applicable local regulations.

10 DATA ANALYSIS AND STATISTICAL METHODS

10.1 Analysis Populations

10.1.1 Safety Analysis Population (SA)

The SA population will include all patients who receive at least 1 dose of study treatment. The SA population will be the primary population for evaluation of safety. PK and efficacy endpoints will be also assessed in this population.

10.1.2 Recommended Phase 2 Dose Population (RP2D)

The RP2D population will include all patients who receive at least 1 dose of study treatment at the dosing regimen to be defined as definitive RP2D. This will be the primary population for evaluating efficacy endpoints.

10.1.3 Objective Response Population (OR)

The OR population will include all patients who had their first scheduled post-baseline tumour assessment (approximately 8 weeks from start of study therapy) or objective disease progression before that. Efficacy endpoints will be assessed in this population.

10.1.4 Intent-to-Treat Population (ITT)

The ITT population will include all enrolled patients with designated study drug assignment. The ITT population will be the primary population for describing patient characteristics.

10.2 Safety Analysis

The SA population will be the primary population for safety evaluation.

10.2.1 Adverse Events

Adverse events will be classified using the medical dictionary for regulatory activities (MedDRA) classification system. Whenever possible, the severity of the toxicities will be graded according to the NCI CTCAE version 5.0, publication date: November 27, 2017;

https://ctep.cancer.gov/protocolDevelopment/electronic_applications/docs/CTCAE_v5_Quick_Reference_8.5x11.pdf).

Adverse events will be summarized by the frequency of patients experiencing treatment emergent adverse events corresponding to body systems and MedDRA preferred term. Treatment-emergent AEs are defined as AEs which occur from the first dose of CT7001 up to 28 days after the last dose of CT7001. Adverse events will be graded by worst NCI CTCAE v5.0 Grade. Adverse events will be summarized by cycle and by relatedness to study treatment. Detailed information collected for each AE will include a description of the event, duration, whether the AE was serious, intensity, relationship to study drug, action taken, and clinical outcome. Emphasis in the analysis will be placed on AEs classified as treatment emergent.

Adverse events leading to death or discontinuation of trial treatment, events classified as NCI CTCAE v5.0 Grade 3 or higher, trial drug-related events, and serious adverse events will be considered with special attention.

10.2.2 Laboratory Abnormalities

Haematology and chemistry laboratory data will be summarized by cycle. The laboratory results will be graded according to the NCI CTCAE v5.0 severity grade. The frequencies of the worst severity grade observed will be displayed. For parameters for which an NCI CTCAE v5.0 scale does not exist, the frequency of patients with values below, within, and above the normal ranges will be summarized by treatment.

10.2.3 Electrocardiogram (ECG) Analysis

All ECGs obtained during the study will be evaluated for safety. The triplicate data will be averaged and all summary statistics and data presentations will use the triplicate averaged data. Any data obtained from ECGs repeated for safety reasons after the nominal time-points will not be averaged along with the preceding triplicates. For all patients in the SA population, individual change in QTcF will be calculated for each nominal post-baseline time point. These individual changes will be summarized using descriptive statistics.

10.3 Efficacy Analysis

The RP2D population will be the primary population for all efficacy analyses. Efficacy endpoints will be assessed as well in the OR and ITT populations. All efficacy endpoints based on radiological (and photographical where applicable) assessments of tumour burden (i.e., OR, DOR, DC, PFS) will be derived using the local radiologist's/investigator's assessment.

10.3.1 Objective Response (OR)

Objective response is defined as a complete response (CR) or partial response (PR) according to the Response Evaluation Criteria in Solid Tumours (RECIST version 1.1; Appendix C).

A patient will be considered to have achieved an OR if the patient has a complete response (CR) or partial response (PR) according to RECIST v.1.1 definitions. Otherwise, the patient will be considered as non-responders in the OR rate analysis. Additionally, patients with inadequate data for tumour assessment (e.g., no baseline or post-baseline assessment) will be considered as non-responders in the OR rate analysis.

The OR rate (ORR) will be estimated by dividing the number of patients with objective response (CR or PR) by the number of patients in a respective analysis population. An exact 95% CI for the response rates will be computed.

10.3.2 Duration of Response (DOR)

Duration of response (DOR) is defined as the time from the first documentation of objective tumour response (CR or PR) to the first documentation of objective tumour progression or to death due to any cause, whichever occurs first. DOR data will be censored on the date of the last tumour assessment on study for patients who do not have objective tumour progression and who do not die due to any cause while on study. DOR will only be calculated for the subgroup of patients with an objective response. The median DOR time estimated from a Kaplan-Meier curve and 95% CI for the median will be computed.

10.3.3 Disease Control (DC)

Disease control (DC) is defined as complete response (CR), partial response (PR), or stable disease (SD) ≥24 weeks according to the RECIST version 1.1 (Appendix C) recorded in the

time period between enrolment and disease progression or death to any cause. The DC rate (DCR) will be estimated by dividing the number of patients with CR, PR, or SD ≥24 weeks by the number of patients in a particular analysis population. A 95% CI for the DC rate will be computed.

10.3.4 Waterfall Plot Analysis

Percentage change in tumour size will be determined for patients with measurable disease at baseline and is derived at each visit by the percentage change from baseline in the sum of diameters of TLs (Target Lesions). The best percentage change in tumour size is defined as the value representing the largest decrease (or smallest increase) from baseline in tumour size.

The best percent change versus baseline in post-baseline aggregate tumour size measurements will be displayed graphically in form of Waterfall plots for the OR population.

10.3.5 Progression-Free Survival (PFS)

PFS is defined as the time from the date of enrolment to the date of the first documentation of objective progression of disease (PD) or death due to any cause in the absence of documented PD, whichever occurs first. PFS is difficult to interpret without prospective control and thus specified as an endpoint in Module 1B-1. However, PFS data will be collected and the median PFS time estimated from a Kaplan-Meier curve and 95% CI for the median will be computed.

PFS data will be censored on the date of the last tumour assessment on study for patients who do not have objective tumour progression and who do not die while on study. Patients lacking an assessment of tumour response after enrolment will have their PFS time censored on the date of enrolment with a duration of 1 day. Additionally, patients who start a new anti-cancer therapy prior to documented PD will be censored at the date of the last tumour assessment prior to the start of the new therapy.

10.3.6 Sample Size and Interim Analysis

The planned sample size of the RP2D population is 30 patients. This is considered of sufficient precision in this first estimation of efficacy to properly inform whether and how best to continue the further development of CT7001 as monotherapy in TNBC.

The following table displays the width of 90% confidence intervals for three examples of true ORR with a sample size of 30 patients in the RP2D population.

Objective Response Rate	90% Confidence Interval for ORR
10%	(3%, 24%)
20%	(9%, 36%)
30%	(17%, 47%)

In case two or three steps of inter-subject dose modification would be required, it is estimated that the total sample size of Module 1B-1 will be a maximum of 50 patients.

As described in Section 5, Module 1B-1 may get amended to a single arm Phase 2b study in the event of an objective response rate (ORR) of >30%. Module 1B-1 will include an interim efficacy analysis when 15 patients in the RP2D population are evaluable for efficacy

assessment. M1B-1 TNBC expansion phase has been designed using a 2-stage design. The null hypothesis for the ORR is 10% and this will be tested against an alternative hypothesis of ≥ 30%. If 0 or 1 responses are observed in the first 15 evaluable patients, no further patients will be recruited and the alternative hypothesis will be rejected. Otherwise, recruitment will continue until a total of 30 evaluable patients are recruited and if ≥6/30 (20%) responses are observed, across both stages of the trial, the null hypothesis will be rejected. This design yields a 1-sided type I error rate of <7.5% and power of >90% when the true response rate is 30%. In case the interim analysis suggests a reasonable possibility of observing an ORR >30% at the end of Module 1B-1, work on a possible subsequent amendment to a Phase 2b study will commence.

In accordance with the interim efficacy analysis, CT7001_001 Protocol Volume 3 (Module 1B-1 TNBC) version 4.0 dated 30th January 2020 the timepoint for evaluation was met on 3rd March 2020. At this timepoint the interim efficacy requirements of 2 or more patients achieving a RECIST partial response had not been met.

1 patient in the first 15 evaluable patients with TNBC treated with CT7001, achieved a partial response, defined by RECIST, in their tumour burden. However, in addition 8 out of 15 evaluable patients achieved stable disease. 4 of these evaluable patients were treated for at least 16 weeks, which in this aggressive tumour type is of significant clinical note. 2 evaluable patients have been treated for more than 36 weeks.

Following discussion with the Data Monitoring Committee (DMC), in view of the clinical benefit seen in 8 of the 15 evaluable patients in addition to the partial response seen in 1 evaluable patient enrolled, it has been decided to modify the protocol to allow the full cohort of 30 evaluable patients to be recruited into this expansion.

10.4 Pharmacokinetic Analysis

Average trough concentrations will be listed by patient. Summary statistics will be provided for trough concentrations by study cycle and for average trough concentrations by patient. The relationship between trough concentration and potential covariates will be evaluated. All patients treated with CT7001 and for whom drug plasma concentration results (from at least 1 visit) are available will be included in the analysis.

In addition, the relationship between exposure and safety and efficacy endpoints may be explored, based on emerging safety and efficacy data. The results of these modelling analyses may be reported separately from the clinical study report.

10.5 Biomarker Analysis

Appropriate statistical methods will be used to investigate any possible relationship of biomarker levels and/or alterations with the recorded efficacy of CT7001.

10.6 Analysis of Other Endpoints

Descriptive statistics will be used to summarize all patient characteristics, treatment administration/compliance, safety parameters, and biomarkers. Data will also be displayed graphically, where appropriate.

Demographic and Baseline Characteristics
Demographics and baseline characteristics (including medical history and baseline disease characteristics) medications will be summarised descriptively for the Safety Population.

Prior and Concomitant Medications
Prior and concomitant medications will be coded using the most current WHO Drug Dictionary and summarised by anatomical therapeutic chemical level 3 and preferred term.

Exposure
Total exposure (date of last dose minus date of first dose +1) and total time on study (date of discontinuation minus date of first dose +1) will be summarised descriptively.

Physical Examination
Abnormal findings will be listed.

ECOG Performance Status
ECOG performance status will be listed individually by subject and summarised descriptively.

Weight and Vital Signs

Weight and vital sign measurements will be listed individually by subject and summarised descriptively.

Pharmacogenomics and Exploratory Research
The results of exploratory biomarker research may be pooled with biomarker data from other studies with the study drug to generate hypotheses to be tested in future studies.

11 ETHICS

11.1 Institutional Review Board (IRB)/Independent Ethics Committee (IEC)

It is the responsibility of the investigator to have prospective approval of the study protocol, protocol amendments, patient information sheets, informed consent documents, and other relevant documents, e.g., recruitment advertisements, if applicable, from the IRB/IEC. The sponsor or its delegate will supply relevant material for the Investigator to submit to the IRB/IEC for review and approval or may submit the required documents on behalf of the investigator, as per local standard practice, guidelines and regulations.

All correspondence with the IRB/IEC should be retained in the Investigator File. Copies of IRB/IEC approvals should be forwarded to Carrick or its delegate. The only circumstance in which an amendment may be initiated prior to IRB/IEC approval is where the change is necessary to eliminate apparent immediate hazards to the patients. In that event, the investigator must notify the IRB/IEC and Carrick or its delegate in writing immediately after the implementation.

The IRB/IEC will be provided with reports at the interval required (not to exceed 1 year) and a report after the completion or discontinuation of the Investigator's participation in the study.

11.2 Ethical Conduct of the Study

The study will be conducted in accordance with legal and regulatory requirements, as well as the general principles set forth in the International Ethical Guidelines for Biomedical Research Involving Human Patients (Council for International Organizations of Medical Sciences 2002), Guidelines for Good Clinical Practice (ICH 1996), and the Declaration of Helsinki (World Medical Association 1996 & 2008). In addition, the study will be conducted in accordance with the protocol, the International Council for Harmonisation (ICH) guideline on harmonization guideline for Good Clinical Practice (GCP), and applicable local regulatory and Data Protection requirements and laws.

11.3 Patient Information and Confidentiality

All parties will ensure protection of the personal data of study subjects and will not include the names of study subjects on any sponsor forms, reports, publications, or in any other disclosures, except where required by laws. A subject’s name, address, birth date and other identifiable data will be replaced by a numerical code consisting of a numbering system provided by Carrick or its delegate to de-identify the study subject. In case of data transfer, Carrick will maintain high standards of confidentiality and protection of the study subjects’ personal data.

11.4 Patient Consent

The informed consent document must be in compliance with ICH GCP, local regulatory requirements, data protection and legal requirements. The informed consent form(s) used during the informed consent process must be reviewed by the sponsor, approved by the IRB/IEC and available for inspection. The investigator must ensure that each study patient is fully informed about the nature and objectives of the study and possible risks associated with participation. The patients will be informed that participation is voluntary and that they can withdraw from the study at any time.

The investigator, or a person designated by the investigator, will obtain written informed consent from each patient before any study-specific activity is performed. The investigator will retain the original of each patient’s signed consent document(s). The patient will be provided with a copy of the signed informed consent form(s).

A copy of the informed consent form (ICF) will be given to the patient, and the original ICF will be maintained with the patients’ study records.

Separate consent will be obtained for participation in the treatment study and related mandatory procedures, and for optional procedures including but not limited to biomarker analyses, pharmacogenomic analyses and tumour biopsies.

Patients can withdraw their consent at any time, in which case the investigator must notify Carrick or its delegate in writing.

11.5 Potential Risks and Benefits

11.5.1 Potential Risks

The nonclinical and emerging clinical safety profile of CT7001 have not identified risks that would preclude investigation in the advanced breast cancer setting. The currently available clinical safety and tolerability data has to be considered as preliminary. However, the available

data has shown appropriate safety and tolerability for proceeding to investigation in patients with metastatic TNBC and documented disease progression to previous chemotherapy.

At 120 mg, 240 mg OD and 360mg OD CT7001 has been generally well tolerated. Adverse drug reactions of note were G1-2 nausea, vomiting and diarrhoea. At 480 mg OD, 3/6 subjects experienced a DLT (G3 diarrhoea, oral mucositis and vomiting). At 180mg BID, 2/8 patients experienced a DLT (G4 thrombocytopenia, Grade 3 weight loss, Grade 3 anorexia, Grade 3 dysphagia/oesophagitis and Grade 3 heartburn). 360 mg OD has been determined as maximum tolerated dose and preliminary recommended Phase 2 dose.

At 240mg OD and 360mg OD there appears to be a ~20% drop in platelet count in all patients. This appears over the first 15 days on study and then is stable for the duration on treatment; in the majority of patients this is within the normal range of platelet counts. All changes in platelet counts appear fully reversible upon discontinuation of CT7001. There have been 13 platelet related AEs reported:

- 2 events of Grade 4 thrombocytopenia (1 at 180mg BID and 1 at 360mg OD); the event at 180mg BID was associated with minor nose bleeding
- 1 event of Grade 3 thrombocytopenia (at 360mg OD)
- 2 events of Grade 2 thrombocytopenia (at 360mg OD)
- 1 event of Grade 2 platelet count decreased (at 360mg OD)
- 4 events of Grade 1 thrombocytopenia (at 360mg OD)
- 3 events of Grade 1 platelet count decreased (1 at 240mg OD and 2 at 360mg OD)

Other laboratory AEs have been rare and mild. The recorded laboratory abnormalities include increase in liver transaminases, prolongation of QTc, 1st degree AV block and anaemia. Of note, a decline in neutrophil count is not anticipated. Accordingly, fever or infection as a clinical complication of severe neutropenia is not expected.

It has been anecdotally noted, by some Investigators, that patients with well controlled diabetes at the time of entry to the study have struggled to control their blood glucose while being dosed with CT7001. The data collected as part of the study neither support or refute this observation. Moving forward, however an assessment of HbA1c will be made in all patients in the study, and those with diabetes at entry will have assessments of fasting glucose.

Module 1B-1 includes mandatory procedures for safety monitoring. Various sections of the study protocol and appendices provide instructions and/or guidance to mitigate the risk of severe treatment-emergent toxicity and in case adverse effects may occur for their prompt and proper medical management.

Nonclinical data from hERG testing and in vivo safety pharmacology studies suggest a low potential of CT7001 for clinically significant prolongation of QTc, cardiovascular, respiratory or central nervous system toxicity (see IB).

Clinical drug interaction data of CT7001 are currently not available. In vitro cytochrome P450 (CYP) studies suggest that CT7001 is unlikely to cause clinically significant hepatic drug interactions through inhibition of CYP1A2, 2C9, 2B6, 2C8 and 3A5. Co-medication with drugs or food that modulate 2D6 or 2C19 and particularly CYP3A4 may affect the exposure of CT7001, and there is a potential for CT7001 to inhibit intestinal and hepatic CYP3A4 which may be clinically significant for CYP3A4 substrates. The potential of CT7001 to inhibit

transporter proteins has not yet been studied. The risk of clinically significant drug interaction is mitigated by provisions in the study protocol (Sections 4.2, 6.11 and Appendix B) not to take certain drugs or food or doing so with caution.

CT7001 exhibits pH-dependent solubility. Medication which increases gastric pH (such as PPIs, H2 antagonists) may reduce the bioavailability of CT7001 and should be avoided unless clinically required. This is described in Section 6.11.2.

Preliminary in vitro studies have shown low potential of CT7001 for mutagenicity and genotoxicity. Nonetheless, patients should be informed of the potential risk of reproductive toxicity and the study protocol requires women of childbearing potential to agree to use adequate contraception during the study and for 6 months after the final dose of CT7001 (Section 4.1). Patients also must have a negative pregnancy test prior to enrolment (Section 18, Table 5). It is currently unknown whether CT7001 is excretion in human breast milk. Therefore, women who are breastfeeding are excluded from the study (Section 4.2).

11.5.2 Potential Benefits

As discussed in Section 2.1, TNBC is an aggressive disease with poor prognosis, chemotherapy continues to serve as standard of care in routine practice, little outcome improvement has been achieved for decades and a significant unmet need remains for new therapies with novel mechanisms of action.

CDK7 has recently emerged as an attractive gene control target in cancers driven by transcriptional dependencies (Kwiatkowski et al, 2014; Chipumuro et al, 2014; Christensen et al, 2014; Wang et al, 2015). A recent study has found that high CDK7 expression is associated with poor prognosis in TNBC and thus targeting CDK7 may be a useful therapeutic strategy for TNBC (Li et al, 2017). Nonclinical studies of CT7001 have shown encouraging activity in tumour models of TNBC (please refer to IB for details). However, it is currently not known whether CT7001 may be of clinical benefit to patients with metastatic TNBC, including patients with prior treatment as required for participation in Module 1B-1.

11.5.3 Overall Risk/Benefit Assessment

The available nonclinical and early clinical safety data suggest a low probability that patients in Module 1B-1 might get exposed to a safety risk which is higher than current standard of care chemotherapy options. The study protocol and procedures include multiple measures aimed at minimizing potential risks as much as feasible. It is currently unknown whether CT7001 may be of clinical benefit to patients with metastatic TNBC. However, metastatic TNBC is an aggressive disease with modest benefit by current options of standard of care beyond the point at which patients are eligible for this study, and there is a strong scientific, nonclinical and clinical rationale for investigating CT7001 in this population. Taken together, the overall assessment of potential risk vs benefit appears acceptable for investigation of CT7001 in a patient population as defined in the eligibility criteria of the current study (Section 4).

11.6 Patient Recruitment

Investigator databases may be used to aid patient recruitment. In case advertisements are used they must have received prior approval by IRB/IEC.

11.7 Reporting of Safety Issues and Serious Breaches of the Protocol or ICH GCP

In the event of any prohibition or restriction imposed (i.e., clinical hold) by an applicable competent authority in any area of the world, or if the investigator is aware of any new information which might influence the evaluation of the benefits and risks of the investigational product, Carrick or its delegate should be informed immediately. In addition, the investigator will inform Carrick immediately of any urgent safety measures taken by the investigator to protect the study patients against any immediate hazard, and of any serious breaches of this protocol or of ICH GCP that the investigator becomes aware of.

12 QUALITY CONTROL AND QUALITY ASSURANCE

During study conduct, Carrick or its agents will conduct periodic monitoring visits to ensure that the study protocol and Good Clinical Practices (GCPs) are being followed. The monitors may review source documents to confirm that the data recorded on eCRFs is accurate. The investigator and institution will allow Carrick monitors or its agents as well as appropriate regulatory authorities direct access to source documents to perform this verification. The study site may be subject to review by an Institutional Review Board (IRB) or Independent Ethics Committee (IEC) and/or to quality assurance audits performed by Carrick or companies working with or on behalf of Carrick and/or to inspection by appropriate regulatory authorities. It is important that the investigator(s) and their relevant personnel are available during the monitoring visits and possible audits or inspections and that sufficient time is devoted to the process.

13 DATA HANDLING AND RECORD KEEPING

13.1 Case Report Forms/Electronic Data Record

As used in this protocol, the term CRF should be understood to refer to either an electronic data record, a paper form or both, depending on the data collection method used in this study.

A CRF is required and should be completed for each included patient. The completed original CRFs are the sole property of Carrick and should not be made available in any form to third parties, except for authorized representatives of Carrick or appropriate regulatory authorities, without written permission from Carrick.

The investigator has ultimate responsibility for the collection and reporting of all clinical, safety and laboratory data entered on the CRFs and any other data collection forms including source documents and ensuring that they are accurate, authentic / original, attributable, complete, consistent, legible, timely (contemporaneous), enduring and available when required.

The CRFs must be signed by the investigator or by an authorized staff member to attest that the data contained on the CRFs is true. Any corrections to entries made in the CRFs, source documents must be dated, initialled and explained (if necessary) and should not obscure the original entry.

In most cases, the source documents are the hospital's or the physician's patient chart. In these cases, data collected on the CRFs must match the data in those charts. In some cases, the CRF, or part of the CRF, may also serve as source documents. In these cases, a document should be available at the investigator’s site as well as at Carrick and clearly identify those data that will be recorded in the CRF, and for which the CRF will stand as the source document.

13.2 Record Retention

To enable evaluations and/or audits from regulatory authorities or Carrick or its designated agents the investigator agrees to keep records, including the identity of all participating patients (sufficient information to link records, e.g., CRFs and hospital records), all original signed informed consent documents, copies of all CRFs, safety reporting forms, source documents and detailed records of treatment disposition, and adequate documentation of relevant correspondence (e.g., letters, meeting minutes, telephone calls reports). The records should be retained by the investigator as long as is required by International Council for Harmonisation (ICH) guidelines, local regulations, or as specified in the Clinical Study Agreement (CSA), whichever is longer. If the investigator becomes unable for any reason to continue to retain study records for the required period (e.g., retirement, relocation), Carrick should be prospectively notified. The study records must be transferred to a designee acceptable to Carrick, such as another investigator, another institution, or to an independent third party arranged by Carrick. Investigator records must be kept for a minimum of 15 years after completion or discontinuation of the study or for longer if required by applicable local regulations. The investigator must obtain Carrick's written permission before disposing of any records, even if retention requirements have been met.

14 DEFINITION OF END OF TRIAL

14.1 End of Trial in all Participating Countries

End of Trial in all participating countries is defined as Last Patient Last Visit.

14.2 End of Trial in a Member State of the European Union

End of Trial in a Member State of the European Union is defined as the time at which it is deemed that sufficient patients have been recruited and completed the study as stated in the regulatory application (i.e., Clinical Trial Application (CTA)) and ethics application in the Member State. Poor recruitment (recruiting less than the anticipated number in the CTA) by a Member State is not a reason for premature termination but is considered a normal conclusion to the study in that Member State.

15 SPONSOR DISCONTINUATION CRITERIA

Premature termination of this study may occur because of a regulatory authority decision, change in opinion of the IRB/IEC, drug safety problems, or at the discretion of Carrick. In addition, Carrick retains the right to discontinue development of CT7001 at any time. If a study is prematurely terminated or discontinued, Carrick will promptly notify the investigator. After

notification, the investigator must contact all participating patients and the hospital pharmacy (if applicable) within a week of notification. As directed by Carrick, all study materials must be collected and all CRFs completed to the greatest extent possible.

16 ADMINISTRATIVE PROCEDURES AND CONSIDERATIONS

16.1 Safety Review Committee

The study will use a Safety Review Committee (SRC). The SRC membership and governance is outlined in a separate charter. The SRC will consist of Principal Investigator (or delegate) as chair, investigators from a representative subset of study sites, and medical and other scientific personnel from the sponsor and/or its delegate. Additional external subject matter expert consultants may get invited on an ad hoc basis, as appropriate. The SRC Remit document will define membership and decision process.

The SRC will be responsible for ongoing monitoring of the safety data as well as other data from patients in Module 1B-1, but also all data emerging form other study modules, particularly Module 1A and Module 4 (food effects on bioavailability of CT7001 in cancer patients). The SRC will also be responsible to determine whether inter-subject modification of the CT7001 dosing regimen in Module 1B-1 appears indicated and which specific regimen to evaluate in a next cohort of patients. This assessment and decision will consider the totality of available data from Module 1B-1 but also Modules 1A 4 and other 1B Expansion Cohorts.

16.2 Protocol Amendments

Any substantive change in the study requires a protocol amendment. All protocol amendments must be reviewed and agreed to by Carrick and the Principal Investigator(s) and approved by applicable regulatory authorities and IRB/IECs before implementation.

Expansion of Module 1B-1 to a Phase 2B study of approximately 130 patients will require a substantive protocol amendment.

16.3 Clinical Study Report

A final clinical study report (CSR) will be prepared in accordance with ICH guidelines on structure and contents of CSRs and any applicable regulatory and legal requirements and the completed CSR will be submitted to all relevant authorities within required time. Considering the multi-module nature of the current study, separate CSRs may be prepared and submitted for each completed study module.

16.4 Financing and Insurance

Financing and insurance will be addressed in a separate clinical trial agreement.

17 PUBLICATION OF STUDY RESULTS

Publication of study results is discussed in the Clinical Study Agreement.

17.1 Communication of Results by Carrick

Carrick fulfils its commitment to publicly disclose clinical trial results through posting the results of this study on eudract.ema.europa.eu/ (EudraCT) and www.clinicaltrials.gov (ClinicalTrials.gov). Carrick posts the results of all studies that it has registered on EudraCT and/or ClinicalTrials.gov regardless of the reason for registration.

At EudraCT, Carrick posts the results ≤ 12 months after the end of the trial.

For posting of results at ClinicalTrials.gov, the timing depends on the status of the Carrick product:

- For studies involving a Carrick product whose drug development is discontinued before approval, Carrick posts the results within one year of discontinuation of the program (if there are no plans for out-licensing) or within two years (if out-licensing plans have not completed).
- For studies involving products that are not yet approved in any country, Carrick posts the results of completed studies within 30 days of US regulatory approval or one year after the first ex-US regulatory approval of the product (if only submitted for approval ex-US).
- For studies involving products applicable under the US Food and Drug Administration Amendments Act of 2007 (FDAAA), i.e., FDA approved products, Carrick posts results within one year of the primary outcome completion date (PCD).
    - Primary Completion Date is defined as the date that the final patient was examined or received an intervention for the purposes of final collection of data for the primary outcome, whether the clinical trial concluded according to the pre-specified protocol or was terminated.
- For studies involving products approved in any country, but not FDA approved, Carrick posts results one year from last patient, last visit (LPLV).

17.2 Publications by Investigators

Carrick has no objection to publication by Investigators of any information collected or generated by Investigators, whether or not the results are favourable to the Investigational Product. However, to ensure against inadvertent disclosure of Confidential Information or unprotected Inventions, Investigators will provide Carrick an opportunity to review any proposed publication or other type of disclosure before it is submitted or otherwise disclosed.

Investigators will provide manuscripts, abstracts, or the full text of any other intended disclosure (poster presentation, invited speaker or guest lecturer presentation, etc) to Carrick at least 30 days before they are submitted for publication or otherwise disclosed. If any patent action is required to protect intellectual property rights, Investigators agree to delay the disclosure for a period not to exceed an additional 60 days.

Investigators will, on request, remove any previously undisclosed Confidential Information (other than the Study results themselves) before disclosure.

If the Study is part of a multi-centre study, Investigators agree that the first publication is to be a joint publication covering all centres. However, if a joint manuscript has not been submitted

for publication within 12 months of completion or termination of the Study at all participating sites, Investigators are free to publish separately, subject to the other requirements of this Section.

For all publications relating to the study, Institutions will comply with recognized ethical standards concerning publications and authorship, including Section II "Ethical Considerations in the Conduct and Reporting of Research" of the Uniform Requirements for Manuscripts Submitted to Biomedical Journals, http://www.icmje.org/index.html#authorship established by the International Committee of Medical Journal Editors.

Publication of study results is also provided for in the Clinical Study Agreement between Carrick and the Institution. In this section entitled Publications by Investigators, the defined terms shall have the meanings given to them in the Clinical Study Agreement.

18 SCHEDULE OF ASSESSMENTS

Table 5: Schedule of Events – Mandatory Procedures

The investigator may schedule visits (unplanned visits) in addition to those listed on the schedule of activities to conduct evaluations or assessments required to protect the wellbeing of the patient.

**Protocol Activity**	**Screening**	**Active Treatment Phase [a] - One Cycle = 21 days**				**End of Treatment/ Withdrawal [c]**	**Post-Treatment Follow-Up [d]**
		**Cycles 1 and 2**			**Cycles ≥3 [v]**		
**Study Day**	**Within 28 days prior to treatment assignment unless specified otherwise**	**Day 1 [b]**	**Day 8**	**Day 15**	**Day 1**		
**Visit Window**		**±2 days**	**±2 days**	**±2 days**	**±3 days**	**Within 28 days**	
Informed Consent [e]	X						
Medical/Oncological History [f]	X						
Signs/Symptoms [g]		X	X	X	X	X	
Physical Examination/Vital Signs [h]	X	X	X	X	X	X	
ECOG Performance Status	X	X	X	X	X	X	
Laboratory Studies							
Haematology [i]	X	X	X	X	X	X	
Serum chemistry [j]	X	X	X	X	X	X	
Pregnancy test, serum oestradiol and FSH (if applicable) [k]	X						
Urinalysis [l]	X	X	X	X	X	X	
Triplicate 12-Lead ECGs [m]	X	X		X	X	X	
Tumour Assessments							
CT/MRI Scans and clinical evaluation of superficial disease [n]	X	Performed/repeated as described in footnote				X	X
Radionuclide Bone Scan, Whole Body [o]	X	Performed/repeated as described in footnote				X	X
Other Clinical Assessments							
Adverse Event Reporting [p]	X	Performed as described in footnote					
Concomitant Medications/Treatments	Recorded from 28 days prior to the start of study treatment up to 28 days after the last dose of study treatment						
Pharmacokinetics [q]		X	X[r]		X	X	
CYP2D6 Polymorphisms [s]		X					
IP Diary [t]		X			X		
Study Treatment							
CT7001 [u]		Daily Dosing					

a. **Active Treatment Phase**: Assessments should be performed prior to dosing on the visit day unless otherwise indicated. One cycle consists of 21 days. A cycle could be longer than 21 days if persistent toxicity delays initiation of the subsequent cycle. Day 1 of any cycle visit should coincide with the day the CT7001 treatment begins. If there are delays due to toxicity, then the start of the next cycle visit will be delayed until the patient has recovered and can begin study treatment again. The active treatment phase is ongoing as long as the patient is receiving CT7001.

b. **Serum Chemistry, Haematology, Physical Examination and ECG** not required if performed as part of screening within 7 days prior to treatment assignment.

c. **End of Treatment/Withdrawal**: Visit to be performed as soon as possible but no later than 4 weeks from the last dose of investigational products and prior to initiation of any new anticancer therapy. Obtain assessments if not completed during the previous 4 weeks on study (or within the previous 8 weeks or 12 weeks [as applicable] for disease assessments).

d. **Post Treatment Follow-up**: Patients who discontinue study treatment should be contacted 28 calendar days (±7 days) after discontinuation of study treatment) to assess if there have been any new adverse events and/or any change to any previously reported adverse events. Telephone contact is acceptable. Patients who discontinue active study treatment for any reason other than objective disease progression or death will continue to have tumour assessments performed every 8 weeks (±7days) for the first year, and then every 12 weeks (±7 days) in subsequent years (calculated from Cycle 1 Day 1) until documented progression, death or onset of new anticancer therapy, whichever occurs first.

e. **Informed Consent**: Informed consent must be obtained prior to any protocol required assessments being performed (with the exception of certain imaging assessments if meeting the criteria defined in the Section 7.1 (Screening).

f. **Medical/Oncological History**: To include information on prior anticancer treatments, alcohol consumption and tobacco use.

g. **Baseline Signs and Symptoms** (tumour-related or otherwise) will be recorded at the Cycle1 Day1 visit prior to initiating treatment and then reported as adverse events during the trial if they worsen in severity or increase in frequency.

h. **Physical Examination/Vital Signs**: Includes an examination of all major body systems and breasts, height (at screening only), weight, supine blood pressure, pulse rate, respiratory rate and body temperature. May be performed by a physician, registered nurse or other qualified health care provider.

i. **Haematology** includes red blood cell count, haematocrit, mean cell volume, reticulocyte count (absolute particle count or relative particle count), white blood cell count with differential (absolute and percent counts of neutrophils, lymphocytes, monocytes, basophils, eosinophils) and platelet count. Additional haematology tests may be performed if clinically indicated.

j. **Serum Chemistry** includes HbA1c, ALT, AST, gamma glutamyl transferase (IU/L), alkaline phosphatase, bilirubin (total), creatine kinase, total protein, albumin, creatinine, urea nitrogen or urea, calcium (total), glucose, sodium, potassium, magnesium, chloride and phosphate. Additional serum chemistry tests may be performed as clinically indicated, including tumour markers (in accordance with local practice).

k. **Pregnancy Test** (serum or urine) at screening only for women of childbearing potential within 7 days of first dose of CT7001. (Tests may be repeated during the active treatment phase or later if so required by IRB/IECs or by local regulations.) Serum oestradiol and follicle stimulating hormone (FSH) levels are analysed at screening to confirm postmenopausal status of women <60 years old and not amenorrhoeic for at least 12 consecutive months with no alternative pathological or physiological cause.

l. **Urinalysis** includes visual examination and a dipstick test (including blood, glucose and protein). If either is abnormal, a microscopic examination should be performed as well.

m. **Triplicate ECGs** to be taken within 3-5 minutes of each other.

n. **CT or MRI Scans**: The same imaging method (CT or MRI) as used at Screening must be used at each subsequent disease assessment. Scans are performed every 8 weeks ((±7 days) for the first year and then every 12 weeks ((±7 days) from Cycle 1 Day 1 until disease progression (reference section 8.2), death, discontinuation from study participation (e.g., patient's request, lost to follow up) or start of subsequent cancer treatment, whichever occurs first.
Clinical Evaluation of Superficial Disease should be performed during active treatment phase on Day 1 of every treatment Cycle (±2 days in first 2 Cycles and ±3 days in subsequent Cycles), at the end of treatment/withdrawal visit and during follow up at same intervals as described for CT or MRI scans.

o. **Radionuclide Bone Scans, Whole Body** should be performed at Screening. If at Screening bone lesions were identified, scans will be repeated during the active treatment phase and during follow-up visits every 16 weeks (± 1 week) from Cycle 1 Day 1, and at the time of confirmation of CR. If no bone lesions were identified at Screening, bone scans will only be performed when clinically indicated (i.e., patient describes new or worsening bone pain or has increasing alkaline phosphatase level or other signs and symptoms of new/progressing bone metastases) but are required at the time of confirmation of CR. New abnormalities found on subsequent bone scans must be confirmed by X-ray, CT scan with bone windows or MRI.

p. **Adverse Event Reporting**: Serious Adverse Events (SAEs) must be reported from the time the patient provides informed consent through and including 28 calendar days after the last administration of the study drug. SAEs occurring after the active reporting period has ended should be reported if the investigator becomes aware of them. All SAEs that the investigator believes have at least a reasonable possibility of being related to the study drugs are to be reported to the Sponsor. All AEs (serious and non-serious) should be recorded on the eCRF from the first dose of study treatment through last patient visit. It is expected that telephone contact with the patient will be made to assess SAEs and AEs 28 calendar days (±7 days) after the last administration of the study drug.

q. **Pharmacokinetics (PK)**: PK blood samples for through concentrations of CT7001 should be collected pre-dose on Day 1 of all Cycles, Day 8 of Cycle 1 and at the end of treatment/withdrawal visit.

r. **PK Blood Sample on Day 8** needs to be collected only in Cycle 1.

s. **CYP2D6 Polymorphisms**: A blood sample should be collected pre-dose on Day 1 of Cycle 1 for genotyping of CYP2D6 allelic variants and copy number change. As CYP2D6 is the main P450 enzyme involved in hepatic Phase I metabolism of CT7001, this is considered a mandatory study procedure.

t. **IP Diary**: Patients will be asked to complete a diary to document their investigational product intake.
u. **CT7001**: CT7001 will be dispensed by an IXRS system. Patients will be required to return all bottles of CT7001 as well as the completed patient diary on Day1 of each cycle for drug accountability.
v. Following 12 months of treatment, from Cycle 18 onwards, at the discretion of the investigator and patient, even numbered study visits may be omitted. Odd numbered study visits must be performed every 42 days +/- 3 days.

Table 6: Schedule of Events – Optional Procedures

Protocol Activity		Cycle 1	Cycle 2		Cycles ≥3	Disease Progression
Study Day	Within 28 days prior to treatment assignment unless specified otherwise	Day 1	Day 1	Day 15	Day 1	
Visit Window		-1 day	-1 day	±2 days	-3 days	+14 days
Informed Consent [a]	X					
Blood Samples for ctDNA [b]	X	X	X		Pre-dose (every odd Cycle from C3)	X
Blood Samples for RNA Sequence Analysis [c]		X[c]	X			
Blood Samples for Exploratory Research [b]		X	X		Pre-dose (every odd Cycle from C3)	X
Blood Sample for Pharmacogenomics [d]		X				
Tumour Biopsies [e]	X			X		X
Archival Tumour Tissue [f]		X				

a. **Informed Consent**: All optional procedures require specific informed consent and this separately for: (1) ctDNA and WBC isolation (2) RNA-seq analysis, (3) exploratory research; (4) pharmacogenomics; (5) tumour biopsies; (6) collection of archival tumour tissue.
b. **Blood samples for ctDNA and for potential future exploratory research** should be collected at Screening (ctDNA only) and prior to dosing of CT7001 on Day 1 of Cycles 1, 2 and 3 and Day 1 in every subsequent odd cycle and, if feasible, within 14 days after documented disease progression.
c. **For RNA-Sequence analysis**, Predose samples should be taken on Day 1 of Cycles 1 and 2 with a second blood sample to be collected on Day 1 of Cycle 1 approximately 4 hours after dosing. Blood Samples for RNA-Sequence to be collected from patients ONLY who have consented to fresh tumour biopsies.
d. **A Blood Sample for Potential Future Pharmacogenomics Analysis** should be taken prior to dosing on Day 1 of Cycle 1. Due to its importance in CT7001 metabolism, analysis of CYP2D6 gene variations (polymorphisms and/or copy number change) is part of the mandatory procedures in this study.
e. **Tumour Biopsies**: A biopsy of a readily accessible tumour lesion should be obtained within 10 days before first dosing, on Day 15 ± 2 days of Cycle 2 and, if feasible, within two weeks after disease progression. First priority is given to formalin-fixed material for immunohistochemistry (including but not limited to CDK7, pPolII, c-Myc, pCDK1 and Ki67/Caspase). Where a second biopsy core is taken, it will be used for RNA ChIP Sequencing.
f. **Archival Tumour Tissue**: Even in case a fresh tumour biopsy can be obtained, an archival formalin-fixed paraffin-embedded tumour tissue sample may be requested.

Table 7: Blood Volumes Required for Mandatory Procedures

**Protocol Activity**	**Screening**	**Active Treatment Phase - One Cycle = 21 days**				**End of Treatment/ Withdrawal** [c]
		**Cycles 1 and 2**			**Cycles ≥3**	
**Study Day**	**Within 28 days prior to treatment assignment unless specified otherwise**	**Day 1**	**Day 8**	**Day 15**	**Day 1**	
**Visit Window**		**±2 days**	**±2 days**	**±2 days**	**±3 days**	**Within 28 days**
Laboratory Studies						
Haematology [a]	X	X	X	X	X	X
Serum Chemistry [b]	X	X	X	X	X	X
Pregnancy Test, Serum Oestradiol and FSH (if applicable) [c]	X					
Other Clinical Assessments						
Pharmacokinetics [d]		X	X		X	X
CYP2D6 Polymorphisms [e]		X				
Total blood volume	20 ml	23 ml	C1D8: 19 ml<br>C2D8: 15 ml	15 ml	19 ml	19 ml

a. **Haematology**: 5 ml per sample.
b. **Serum Chemistry:** 10 ml per sample.
c. **Pregnancy Test:** 5 ml per sample.
d. **Pharmacokinetics:** 4 ml per sample. Not applicable at C2D8.
e. **CYP2D6 Polymorphisms**: 4 ml per sample.

Table 8: Blood Volumes Required for Optional Procedures

Protocol Activity		Cycle 1	Cycle 2	Cycles ≥3	Disease Progression
**Study Day**	**Within 28 days prior to treatment assignment unless specified otherwise**	**Day 1**	**Day 1**	**Day 1**	
**Visit Window**		**-1 day**	**-1 day**	**±3 days**	**+14 days**
Blood Samples for ctDNA [a]	X	X	X	every odd Cycle from C3	X
Blood Samples for RNA-Sequence Analysis [b]		X[e]	X		
Blood Samples for Exploratory Research [c]		X	X	every odd Cycle from C3	X
Blood Sample for Pharmacogenomics [d]		X			
Total blood volume	10 ml	44 ml	30 ml	Odd cycles: 20 ml<br>Even cycles: 0 ml	20 ml

a. **Blood Samples for ctDNA**: 10 ml per sample.
b. **Blood Samples for RNA-Sequence analysis:** 10 ml per sample.
c. **Blood Samples for Exploratory Research:** 10 ml per sample.
d. **Blood Sample for Pharmacogenomics**: 4 ml per sample.
e. **Blood Samples for RNA-Sequence analysis:** 10 ml per sample. Predose samples should be taken on Day 1 of Cycles 1 and 2 with a second blood sample to be collected on Day 1 of Cycle 1 approximately 4 hours after dosing. Blood Samples for RNA-Sequence to be collected from patients ONLY who have consented to fresh tumour biopsies.

19 REFERENCES

Adams S *et al.* (i) Phase 2 study of pembrolizumab monotherapy for previously treated metastatic triple-negative breast cancer: KEYNOTE-086 cohort A. *ASCO Annual Meeting*, 2017; abstract 1008

Adams S *et al.* (ii) Phase 2 study of pembrolizumab as first-line therapy for PD-L1-positive metastatic triple-negative breast cancer: KEYNOTE-086 cohort B. *ASCO Annual Meeting*, 2017; abstract 1088

Ainscow E *et al*. CT7001: An orally bioavailable CDK7 inhibitor is a potential therapy for breast, small-cell lung and haematological cancers. *American Association of Cancer Research Annual Meeting*, 2018; http://www.abstractsonline.com/pp8/#!/4562/presentation/6442

Ali S *et al*. ICEC0942, a new oral selective inhibitor of the cell cycle and transcriptional regulator CDK7 for the treatment of estrogen receptor positive and negative breast cancer. *Cancer Res*, 2018; 78(4), supplement/P1-10-05 http://cancerres.aacrjournals.org/content/78/4_Supplement/P1-10-05

Bahl A *et al*. Activity of CT7001 an orally bio-available cyclin-dependent kinase 7 selective inhibitor in models of triple negative breast cancer. *San Antonio Breast Cancer Conference*, 2017; https://www.abstracts2view.com/sabcs17/view.php?nu=SABCS17L_109&terms=

Bajaj P *et al*. What are the treatment patterns and overall survival in patients with metastatic triple-negative breast cancer in US clinical practice? *Ann Oncol*, 2017; 28: Suppl. 5, 268P

Bardia A *et al*. Efficacy and safety of anti-trop-2 antibody drug conjugate sacituzumab govitecan (IMMU-132) in heavily pretreated patients with metastatic triple-negative breast cancer. *J Clin Oncol*, 2017; 35:2141-2148

Bareche Y *et al*. Unravelling triple-negative breast cancer molecular heterogeneity using an integrative multiomic analysis. *Ann Oncol*, 2018; 29:895–902

Baselga J *et al*. Randomized phase II study of the anti–epidermal growth factor receptor monoclonal antibody cetuximab with cisplatin versus cisplatin alone in patients with metastatic triple-negative breast cancer. *J Clin Oncol*, 2013; 31:2586-2592

Cardoso *et al*. Early Breast Cancer: ESMO Clinical Practice Guidelines for diagnosis, treatment and follow-up. *Annals of Oncology*, 2019; 30: 1194–1220

Carey LA *et al*. TBCRC 001: randomized phase II study of cetuximab in combination with carboplatin in stage IV triple-negative breast cancer. *J Clin Oncol*, 2012; 30:2615-2623

Chen D *et al*, Activation of estrogen receptor alpha by S118 phosphorylation involves a ligand-dependent interaction with TFIIH and participation of CDK7. *Mol Cell*, 2000; 6:127–37

Chen MT *et al*. Comparison of patterns and prognosis among distant metastatic breast cancer patients by age groups: a SEER population based analysis. *Nature Scientific* Reports, 2017; 7:1-8

Chipumuro E *et al*. CDK7 inhibition suppresses super-enhancer linked oncogenic transcription in MYCN-driven cancer. *Cell*, 2014; 159:1126–39

Christensen CL, Kwiatkowski N, Abraham BJ, Carretero J, Al-Shahrour F, et al. Targeting Transcriptional Addictions in Small Cell Lung Cancer with a Covalent CDK7 Inhibitor. Cancer Cell. 2014: 26 (6); 909–922.

Clark K *et* al. CT7001, a novel orally bio-available CDK7 inhibitor, is highly active in in-vitro and in-vivo models of AML. *American Society of Hematology*, 2017; http://www.bloodjournal.org/content/130/Suppl_1/2645

Cortes J *et al.* Eribulin monotherapy versus treatment of physician's choice in patients with metastatic breast cancer (EMBRACE): a phase 3 open-label randomised study. *Lancet*, 2011; 377:914–23

Dieras V *et al*. Randomized phase II placebo-controlled trial of onartuzumab and/or bevacizumab in combination with weekly paclitaxel in patients with metastatic triple-negative breast cancer. *Ann Oncol*, 2015; 26:1904–1910

Dua I *et al*. Immunotherapy for triple-negative breast cancer: a focus on immune checkpoint inhibitors. *Am J Hematol/Oncol*, 2017; 13:20–27

Feaver WJ *et al*. Relationship of CDK-activating kinase and RNA polymerase II CTD kinase TFIIH/TFIIK. *Cell*, 1994; 79:1103–1109

Fisher RP. Secrets of a double agent: CDK7 in cell-cycle control and transcription. *J Cell Sci*, 2005; 118:5171–80

Fisher RP, Morgan DO. A novel cyclin associates with MO15/CDK7 to form the CDK-activating kinase. *Cell*, 1994; 78:713–724

Gadi VK and Davidson NE. Practical approach to triple-negative breast cancer. *J Oncol Practice*, 2017; 13:293-300

Glover-Cutter K *et al*. TFIIH-associated Cdk7 kinase functions in phosphorylation of C-terminal domain Ser7 residues, promoter-proximal pausing, and termination by RNA polymerase II. *Molecular and cellular biology*, 2009; 29:5455-5464

Hammond MEH *et al*. American Society of Clinical Oncology/College of American Pathologists guideline recommendations for immunohistochemical testing of estrogen and progesterone receptors in breast cancer. *Arch Pathol Lab Med*, 2010; 134:907-922

Horning S. Frontline atezolizumab/nab-paclitaxel improves PFS in phase III TNBC trial. Press release http://www.onclive.com/web-exclusives/frontline-atezolizumabnabpaclitaxel-improves-pfs-in-phase-iii-mbc-trial. 2018

Isakoff J *et al*. TBCRC009: A multicenter phase II clinical trial of platinum monotherapy with biomarker assessment in metastatic triple-negative breast cancer. *J Clin Oncol*, 2015; 33:1902-1909

Kaufmann PA *et al.* Phase III open-label randomized study of eribulin mesylate versus capecitabine in patients with locally advanced or metastatic breast cancer previously treated with an anthracycline and a taxane. *J Clin Oncol*, 2015; 33:594-602

Kim A-B *et al*. Ipatasertib plus paclitaxel versus placebo plus paclitaxel as first-line therapy for metastatic triple-negative breast cancer (LOTUS): a multicentre, randomised, double-blind, placebo-controlled, phase 2 trial. *Lancet Oncol*, 2017; 18:1360–72.

Kwiatkowski N *et al*. Targeting transcription regulation in cancer with a covalent CDK7 inhibitor. *Nature*, 2014; 511:616–620

Li B *et al*. Therapeutic rationale to target highly expressed CDK7 conferring poor outcomes in triple-negative breast cancer. *Cancer Res*, 2017; 77:3834-3845

Litton J *et al*. Talazoparib in patients with advanced breast cancer and a germline BRCA mutation. *N Engl J Med*, 2018, 379:753-763.

Martin M *et al*. Randomised phase III trial of vinflunine plus capecitabine versus capecitabine alone in patients with advanced breast cancer previously treated with an anthracycline and resistant to taxane. *Ann Oncol*, 2018; 29:1195–1202

Mehanna *et al*. Triple-negative breast cancer: current perspective on the evolving therapeutic landscape.,. 2019;11: 431-437

Metzger-Filho O *et al*. Dissecting the heterogeneity of triple-negative breast cancer. *J Clin Oncol*, 2012; 30:1879-1887

O'Shaughnessy J *et al*. Phase III study of iniparib plus gemcitabine and carboplatin versus gemcitabine and carboplatin in patients with metastatic triple-negative breast cancer. *J Clin Oncol*, 2014; 32:3840-3847

Pareja F *et al*. Triple-negative breast cancer: the importance of molecular and histologic subtyping, and recognition of low-grade variants. *npj Breast Cancer*, 2016; 2:16036; doi:10.1038/npjbcancer.2016.36 (published online)

Patel H *et al*. ICEC0942, an Orally Bioavailable Selective Inhibitor of CDK7 for Cancer Treatment. *Mol Cancer Ther.* 2018 Jun;17(6):1156-1166.

Rasool RU, Natesan R, Deng Q, *et al.* CDK7 Inhibition Suppresses Castration-Resistant Prostate Cancer through MED1 Inactivation. Cancer Discov. 2019;9(11):1538-1555. doi:10.1158/2159-8290.CD-19-0189

Rubin LG, Levin MJ, Ljungman P, *et al*. 2013 IDSA Clinical Practice Guideline for Vaccination of the Immunocompromised Host. Clinical Infectious Diseases, 2014; 58(3):e44–100

Schachter MM, Fisher RP. The CDK-activating kinase Cdk7: taking yes for an answer. *Cell Cycle*, 2013; 12:3239–40

Schachter MM *et al*. A Cdk7-Cdk4 T-loop phosphorylation cascade promotes G1 progression. *Mol Cell*, 2013; 50:250–260

Sharma P. Biology and management of patients with triple-negative breast cancer. *The Oncologist*, 2016; 21:1050–106

Tutt A *et al*. Carboplatin in BRCA1/2-mutated and triple-negative breast cancer BRCAness subgroups: the TNT Trial. *Nature Medicine*, 2018; 24: 628–637

Wang Y *et al*. 2015 CDK7-dependent transcriptional addiction in triple-negative breast cancer. *Cell*, Sep 24;163(1):174-186

Wolff AC *et al*. Human epidermal growth factor receptor 2 testing in breast cancer: American Society of Clinical Oncology/College of American Pathologists Clinical practice guideline focused update. *J Clin Oncol*, 2018; 36:2105-2122

Wölwer CB *et al.* A Chemical Screening Approach to Identify Novel Key Mediators of Erythroid Enucleation. *PLoS One*, 2015, Nov 16; 10(11):e0142655.

Yardley DA *et al*. Nab-paclitaxel plus carboplatin or gemcitabine vs gemcitabine plus carboplatin as first-line treatment for patients with triple-negative metastatic breast cancer: Results from the tnAcity trial. *Ann Oncol*, 2018; mdy201, https://doi.org/10.1093/annonc/mdy201 (published online)

Zeichner B *et al*. A review of systemic treatment in metastatic triple-negative breast cancer. *Breast Cancer: Basic and Clin Res*, 2016; 10:25–36.

APPENDICES TO VOLUME 3, MODULE 1, PART B-1 (TNBC)

APPENDIX A – DRUGS KNOWN TO PREDISPOSE TO TORSADE DE POINTES

Generic Name	Brand Name(s)
Amiodarone	Cordarone®, Pacerone®
Arsenic trioxide	Trisenox®
Astemizole	Hismanal®
Azithromycin	Zithromax®
Bepridil	Vascor®
Chloroquine	Aralen®
Chlorpromazine	Thorazine®
Cisapride	Propulsid®
Citalopram	Celexa®
Clarithromycin	Biaxin®
Disopyramide	Norpace®
Dofetilide	Tikosyn®
Domperidone	Motilium®
Droperidol	Inapsine®
Erythromycin	Erythrocin®, E.E.S. ®
Flecainide	Tambocor®
Halofantrine	Halfan®
Haloperidol	Haldol®
Ibutilide	Corvert®
Levomethadyl	Orlaam®
Mesoridazine	Serentil®
Methadone	Dolophine®, Methadose®
Moxifloxacin	Avelox®
Ondansetron*	Zofran®
Pentamidine	Pentam®, NebuPent®
Pimozide	Orap®

Probucol	Lorelco®
Procainamide	Pronestyl®, Procan®
Quinidine	Cardioquin®, Quinaglute®
Sotalol	Betapace®
Sparfloxacin	Zagam®
Terfenadine	Seldane®
Thioridazine	Mellaril®
Vandetanib	Caprelsa®

* when administered intravenously at high dose (32 mg)

Adapted from the University of Arizona Cancer Center for Education and Research on Therapeutics: "Torsades List: Drugs with a Risk of Torsades de Pointes," drugs that are generally accepted by the QTdrugs.org Advisory Board to carry a risk of Torsades de Pointes on the University of Arizona CERT website: http://www.crediblemeds.org/.

This list is not meant to be considered all inclusive. See website for current list.

APPENDIX B - MEDICATIONS, HERBAL SUPPLEMENTS, AND FOODS THAT SIGNIFICANTLY INDUCE OR INHIBIT CYTOCHROME P450 3A4, 2C19 AND/OR 2D6 OR P GLYCOPROTEIN ACTIVITY

Strong CYP3A4, CYP2C19 and/or CYP2D6 Inhibitors

The drugs in Appendix B Table 1 are known to strongly inhibit CYP3A4, CYP2C19 and/or CYP2D6 and should be avoided while administering CT7001 (FDA Drug Development and Drug Interactions: Table of Substrates, Inhibitors and Inducers, and the Flockhart Table, Indiana University School of Medicine). The list is not exhaustive, and a similar restriction will apply to other agents that are known to strongly modulate CYP3A4, CYP2C19 and/or CYP2D6. Medical judgement is required but **please be vigilant for signs and/or changes in tolerability particularly with 3A4 substrates.**

Please contact the Bionical Emas medical monitor with any queries you have on this issue.

Appendix B Table 1: Strong CYP3A4, CYP2C19 and/or CYP2D6 Inhibitors

CYP3A4 Inhibitor	CYP2C19 Inhibitor	CYP2D6 Inhibitor
Clarithromycin,	Fluconazole	Bupropion
Telithromycin,	Fluoxetine	Cinacalcet
Troleandomycin	Fluvoxamine	Fluoxetine
Indinavir,	Ticlopidine	Paroxetine
lopinavir,	Voriconzole	Quinidine
Nelfinavir,		Terbinafine
Ritonavir,		
Saquinavir		
Tipranavir		
Telaprevir		
Itraconazole		
Ketoconazole		
Posaconazole		
Voriconazole		
Suboxone		
Nefadozone		
Boceprivir		
Conivaptan		
Cobicistat		
Danoprevir		
Elvitegravir		
Grapefruit juice		
Paritaprevir		
Idelalisib		
Diltiazem,		

Appendix B Table 1: Strong CYP3A4, CYP2C19 and/or CYP2D6 Inhibitors

CYP3A4 Inhibitor	CYP2C19 Inhibitor	CYP2D6 Inhibitor
Nelfinavir		

Strong CYP3A4, CYP2C19 and/or CYP2D6 Inducers

The drugs in Appendix B Table 2 are known to strongly induce CYP3A4, CYP2C19 and/or CYP2D6 and should be avoided while administering CT7001 (FDA Drug Development and Drug Interactions: Table of Substrates, Inhibitors and Inducers, and the Flockhart Table, Indiana University School of Medicine). The list is not exhaustive, and a similar restriction will apply to other agents that are known to strongly induce CYP3A4, CYP2C19 and/or CYP2D6. Medical judgement is required.

Please contact the Bionical Emas medical monitor with any queries you have on this issue.

Appendix B Table 2: Strong CYP3A4, CYP2C19 and/or CYP2D6 Inducers

CYP3A4 Inducer	CYP2C19 Inducer	CYP2D6 Inducer
Carbamazepine<br>Enzalutamide<br>Mitotane,<br>Phenytoin<br>Rifampin<br>St. John's wort<br>Phenobarbital<br>Rifabutin<br>Nevirapine<br>Troglitazone	Aprepitant<br>Carbamazepine<br>Enzalutamide<br>Rifampin<br>Ritonavir<br>Nevirapine<br>Phentobarbital<br>St John's Wort	None known

Drugs whose clearance is dependent on CYP3A4 and have a narrow therapeutic index

There are currently no data confirming that there is a PK interaction between CT7001 and other drugs. However, in vitro data suggests CT7001 has the potential to cause drug interactions at the intestinal and hepatic level through CYP3A4.

CT7001 shows a weak potential to inhibit CYP2D6 and 2C19 which is unlikely to be clinically significant but should be considered when co-prescribing sensitive 2D6 and 2C19 substrates and substrates with narrow therapeutic index (e.g. S-mephenytoin). The potential for CT7001 to inhibit transporter systems is currently unknown.

If CT7001 is co-administered with CYP3A substrates with narrow therapeutic indices, including but not limited to alfentanil, cisapride, cyclosporine, ergot derivatives, fentanyl, pimozide, quinidine, sirolimus, tacrolimus, atorvastatin, lovastatin and simvastatin the subject should be closely monitored for signs of changed tolerability as a result of increased exposure

of the concomitant medication. This list is not intended to be exhaustive, and similar precautions should be applied to other agents that are known to depend on CYP3A4 for metabolism.

Medical judgement is required. Please contact the Bionical Emas medical monitor with any queries you have on this issue.

P-Glycoprotein (PGP) Inhibitors and Inducers

The drugs in Appendix B Table 3 are known to strongly inhibit or induce PGP and should be avoided while administering CT7001 (FDA Drug Development and Drug Interactions: Table of Substrates, Inhibitors and Inducers, and the Flockhart Table, Indiana University School of Medicine). The list is not exhaustive, and a similar restriction will apply to other agents that are known to strongly induce PGP. Medical judgement is required.

Please contact the Bionical Emas medical monitor with any queries you have on this issue.

Appendix B Table 3: P-Glycoprotein (PGP) Inhibitors and Inducers

PGP Inhibitors	PGP Inducers
Amiodarone	Avasimibe
Carvedilol	Carbamazepine
Clarithromycin	Phenytoin
Dronedarone	Rifampin
Itraconazole	Ritonavir
Lapatinib	St. John’s Wort
Lovinavir	Tipranavir
Ritonavir	
Propafenone	
Quinidine	
Ranolazine	
Ritonavir	
Saquinavir	
Telaprivir	
Tipranavir	
Verapamil	

APPENDIX C - RESPONSE EVALUATION CRITERIA IN SOLID TUMOURS (RECIST) VERSION 1.1

Adapted from E.A. Eisenhauer, et al: New response evaluation criteria in solid tumours: Revised RECIST guideline (version 1.1). European Journal of Cancer 45 (2009) 228–247.

CATEGORIZING LESIONS AT BASELINE

Measurable Lesions

Lesions that can be accurately measured in at least one dimension.

- Lesions with longest diameter twice the slice thickness and at least 10 mm or greater when assessed by CT or MRI (slice thickness 5-8 mm).
- Lesions with longest diameter at least 20 mm when assessed by Chest X-ray.
- Superficial lesions with longest diameter 10 mm or greater when assessed by calliper.
- Malignant lymph nodes with the short axis 15 mm or greater when assessed by CT.

NOTE: The shortest axis is used as the diameter for malignant lymph nodes, longest axis for all other measurable lesions.

Non-Measurable Disease

Non-measurable disease includes lesions too small to be considered measurable (including nodes with short axis between 10 and 14.9 mm) and truly non-measurable disease such as pleural or pericardial effusions, ascites, inflammatory breast disease, leptomeningeal disease, lymphangitic involvement of skin or lung, clinical lesions that cannot be accurately measured with calipers, abdominal masses identified by physical examination that are not measurable by reproducible imaging techniques.

- Bone disease: Bone disease is non-measurable with the exception of soft tissue components that can be evaluated by CT or MRI and meet the definition of measurability at baseline.
- Previous local treatment: A previously irradiated lesion (or lesion subjected to other local treatment) is non-measurable unless it has progressed since completion of treatment.

Normal Sites

- Cystic lesions: Simple cysts should not be considered as malignant lesions and should not be recorded either as target or non-target disease. Cystic lesions thought to represent cystic metastases can be measurable lesions if they meet the specific definition above. If non-cystic lesions are also present, these are preferred as target lesions.
- Normal nodes: Nodes with short axis <10 mm are considered normal and should not be recorded or followed either as measurable or non-measurable disease.

RECORDING TUMOUR ASSESSMENTS

All sites of disease must be assessed at baseline. Baseline assessments should be done as close as possible prior to study start. For an adequate baseline assessment, all required scans must be done within 28 days prior to treatment and all disease must be documented appropriately. If baseline assessment is inadequate, subsequent statuses generally should be indeterminate.

Target Lesions

All measurable lesions up to a maximum of 2 lesions per organ, 5 lesions in total, representative of all involved organs, should be identified as target lesions at baseline. Target lesions should be selected on the basis of size (longest lesions) and suitability for accurate repeated measurements. Record the longest diameter for each lesion, except in the case of pathological lymph nodes for which the short axis should be recorded. The sum of the diameters (longest for non-nodal lesions, short axis for nodal lesions) for all target lesions at baseline will be the basis for comparison to assessments performed on study.

- If two target lesions coalesce the measurement of the coalesced mass is used. If a large target lesion splits, the sum of the parts is used.
- Measurements for target lesions that become small should continue to be recorded. If a target lesion becomes too small to measure, 0 mm should be recorded if the lesion is considered to have disappeared; otherwise a default value of 5 mm should be recorded.

NOTE: When nodal lesions decrease to <10 mm (normal), the actual measurement should still be recorded.

Non-Target Disease

All non-measurable disease is non-target. All measurable lesions not identified as target lesions are also included as non-target disease. Measurements are not required but rather assessments will be expressed as ABSENT, INDETERMINATE, PRESENT/NOT INCREASED, INCREASED. Multiple non-target lesions in one organ may be recorded as a single item on the case report form (e.g. 'multiple enlarged pelvic lymph nodes' or 'multiple liver metastases').

OBJECTIVE RESPONSE STATUS AT EACH EVALUATION

Disease sites must be assessed using the same technique as baseline, including consistent administration of contrast and timing of scanning. If a change needs to be made the case must be discussed with the radiologist to determine if substitution is possible. If not, subsequent objective statuses are indeterminate.

Target Disease

- Complete Response (CR): Complete disappearance of all target lesions with the exception of nodal disease. All target nodes must decrease to normal size (short axis < 10 mm). All target lesions must be assessed.
- Partial Response (PR): Greater than or equal to 30% decrease under baseline of the sum of diameters of all target measurable lesions. The short diameter is used in the sum for target nodes, while the longest diameter is used in the sum for all other target lesions. All target lesions must be assessed.

- Stable Disease (SD): Does not qualify for CR, PR or Progression. All target lesions must be assessed. Stable can follow PR only in the rare case that the sum increases by less than 20% from the nadir, but enough that a previously documented 30% decrease no longer holds.
- Progressive Disease (PD): 20% increase in the sum of diameters of target measurable lesions above the smallest sum observed (over baseline if no decrease in the sum is observed during therapy), with a minimum absolute increase of 5 mm.
- Indeterminate. Progression has not been documented, and:
  - One or more target measurable lesions have not been assessed.
  - Or assessment methods used were inconsistent with those used at baseline.
  - Or one or more target lesions cannot be measured accurately (e.g., poorly visible unless due to being too small to measure).
  - Or one or more target lesions were excised or irradiated and have not reappeared or increased.

Non-Target Disease

- CR: Disappearance of all non-target lesions and normalization of tumour marker levels. All lymph nodes must be ‘normal’ in size (<10 mm short axis).
- Non-CR/Non-PD: Persistence of any non-target lesions and/or tumour marker level above the normal limits.
- PD: Unequivocal progression of pre-existing lesions. Generally, the overall tumour burden must increase sufficiently to merit discontinuation of therapy. In the presence of SD or PR in target disease, progression due to unequivocal increase in non-target disease should be rare.

Indeterminate: Progression has not been determined and one or more non-target sites were not assessed or assessment methods were inconsistent with those used at baseline.

New Lesions

The appearance of any new unequivocal malignant lesion indicates PD. If a new lesion is equivocal, for example due to its small size, continued assessment will clarify the etiology. If repeat assessments confirm the lesion, then progression should be recorded on the date of the initial assessment. A lesion identified in an area not previously scanned will be considered a new lesion.

Supplemental Investigations

- If CR determination depends on a residual lesion that decreased in size but did not disappear completely, it is recommended the residual lesion be investigated with biopsy or fine needle aspirate. If no disease is identified, objective status is CR.
- If progression determination depends on a lesion with an increase possibly due to necrosis, the lesion may be investigated with biopsy or fine needle aspirate to clarify status.

Subjective Progression

Patients requiring discontinuation of treatment without objective evidence of disease progression should not be reported as PD on tumour assessment eCRFs. This should be

indicated on the end of treatment eCRF as off treatment due to Global Deterioration of Health Status. Every effort should be made to document objective progression even after discontinuation of treatment.

Appendix 3 Table 1. Objective Response Status at Each Evaluation

Target Lesions	Non-Target Disease	New Lesions	Objective Status
CR	CR	No	CR
CR	Non-CR/Non-PD, Indeterminate or Missing	No	PR
PR	Non-CR/Non-PD, Indeterminate or Missing	No	PR
SD	Non-CR/Non-PD, Indeterminate or Missing	No	SD
Indeterminate or Missing	Non-PD	No	Indeterminate
PD	Any	Yes or No	PD
Any	PD	Yes or No	PD
Any	Any	Yes	PD

APPENDIX D - NEW YORK HEART ASSOCIATION (NYHA) FUNCTIONAL CLASSIFICATION

NYHA Functional Classification

NYHA Class	Patients with Cardiac Disease (Description of HF Related Symptoms)
Class I (Mild)	Patients with cardiac disease but without resulting in limitation of physical activity. Ordinary physical activity does not cause undue fatigue, palpitation (rapid or pounding heart beat), dyspnoea (shortness of breath), or anginal pain (chest pain).
Class II (Mild)	Patients with cardiac disease resulting in slight limitation of physical activity. They are comfortable at rest. Ordinary physical activity results in fatigue, palpitation, dyspnoea, or anginal pain
Class III (Moderate)	Patients with cardiac disease resulting in marked limitation of physical activity. They are comfortable at rest. Less than ordinary activity causes fatigue, palpitation, dyspnoea, or anginal pain.
Class IV (Severe)	Patients with cardiac disease resulting in the inability to carry on any physical activity without discomfort. Symptoms of heart failure or the anginal syndrome may be present even at rest. If any physical activity is undertaken, discomfort is increased.

The Criteria Committee of the New York Heart Association. *Nomenclature and Criteria for Diagnosis of Diseases of the Heart and Great Vessels*. 9th ed. Boston, Mass: Little, Brown & Co; 1994:253-256.

APPENDIX E - ACTIONS REQUIRED IN CASES OF COMBINED INCREASE OF AMINOTRANSFERASE AND TOTAL BILIRUBIN – HY'S LAW

BACKGROUND

Hy's law is a rule that states that a subject is at high risk of a fatal drug-induced liver injury (DLI) if given a medication that causes hepatocellular injury (not cholestatic injury) with jaundice. The law is based on observations by Hy Zimmerman, a major scholar of drug-induced liver injury.

INVESTIGATOR ACCOUNTABILITIES

Each Investigator, or delegate, will regularly review the subject's laboratory data for increases in liver biochemistry parameters pertaining to cases of Hy's Law (HL). It is the Investigators responsibility to assess whether a subject meets Potential Hy's Law (PHL) criteria at any point during the CT7001_001 Study.

The Investigator is responsible for recording data related PHL/HL cases and for reporting AEs and SAEs as per the processes outlined in Section 9.12 of the CT7001_001 Module 1 Part B-1 Clinical Study Protocol.

The assessment of PHL cases may be carried out in conjunction with representatives from Carrick.

DEFINITIONS

Potential Hy's Law (PHL)

- Aspartate Aminotransferase (AST) or Alanine Aminotransferase (ALT) $\geq 3 \times$ ULN and Total Bilirubin (TBL) $\geq 2 \times$ ULN at any point during the study irrespective of an increase in Alkaline Phosphatase (ALP).

Hy's Law (HL)

- AST or ALT $\geq 3 \times$ ULN and TBL $\geq 2 \times$ ULN, where no other reason, other than the IMP, can be found to explain the combination of increases, e.g., elevated ALP indicating cholestasis, viral hepatitis, another drug.

PHL and HL elevations do not have to occur at the same time or within a specified time frame.

IDENTIFICATION OF POTENTIAL HY'S LAW CASES

Laboratory data from subjects participating in the CT7001_001 study should be assessed to identify occurrence of the following criteria:

- Alanine aminotransferase $\geq 3 \times$ ULN
- Aspartate aminotransferase $\geq 3 \times$ ULN
- Total bilirubin $\geq 2 \times$ ULN

The Investigator, or delegate, will assess follow-up laboratory reports to determine if PHL/HL criteria are met. The Investigator, or delegate, will enter the laboratory data into the CT7001_001 clinical study database to facilitate review by the SRC and / or Sponsor as necessary.

PHL FOLLOW-UP REVIEW AND ASSESSMENT

If PHL criteria <u>are not</u> met:

If the subject does not meet PHL criteria the Investigator will conduct follow-up on subsequent laboratory results as per the Clinical Study Protocol.

If PHL criteria <u>are</u> met:

If the subject meets PHL criteria the Investigator will immediately notify the CT7001_001 Study Team Physician and the Bionical Emas Drug Safety Physician

The Investigator, in conjunction with the CT7001_001 Study Team Physician and the Bionical Emas Drug Safety Physician will decide on treatment options to manage cases PHL, to include:

- Ongoing assessment of LFTs and associated clinical symptoms until values return to normal ranges (as assessed by the Investigator).

If the PHL case meets serious criteria, report the event as an SAE using standard reporting procedures outlined in the CT7001_001 Clinical Study Protocol (assessment of seriousness will be performed in conjunction with the Study Team Physician.

FOLLOW-UP, REVIEW AND ASSESSMENT OF PHL

Cases

- No later than 3 weeks after the first LFT abnormality was detected, the Investigator, in conjunction with the CT7001_001 Study Team Physician will assess the case and decide the case is a drug induced liver injury (DILI) caused by the IMP. The Bionical Emas Drug Safety Physician may also be involved in this review.

Following the review and assessment, the Investigator will follow the instructions below.

- If there is an agreed alternative explanation for the ALT or AST and TBL elevations, a determination of whether the alternative explanation is an AE will be made and subsequently whether the AE meets the criteria for a SAE as defined in Section 9.2.

- If the alternative explanation is not an AE, record the alternative explanation on the appropriate eCRF.

If the alternative explanation is an AE/SAE, record the AE /SAE in the eCRF accordingly, and follow standard reporting processes outlines in the CT7001_001 Clinical Study Protocol. If it is agreed that there is no explanation that would explain the ALT or AST and TBL elevations other than the IMP:

- Report an SAE (report term 'Hy's Law') according to the standard reporting processes outlined in the CT7001_001 Clinical Study Protocol.
- The 'Medically Important' serious criterion should be used if no other serious criteria apply.
- As there is no alternative explanation for the HL case, a causality assessment of 'related' should be assigned.

Actions Required for Repeat Episodes of PHL

When a subject meets PHL criteria on more than one instance, investigator should determine the cause for the previous occurrence of PHL criteria being met and answer the following question:

- Was the alternative cause for the previous occurrence of PHL criteria being metchronic or progressing malignant disease?
- If **'No'**: follow the reporting process described in this Appendix.
- If **'Yes'**: determine if there has been a significant change in the subject's condition compared with when PHL criteria were previously met.
- If there is no significant change no action is required.
  If there is a significant change, then the reporting process described in this Appendix should be followed (a 'significant' change in the subject's condition refers to a clinically relevant change in any of the individual liver biochemistry parameters (ALT, AST or total bilirubin) in isolation or in combination, or a clinically relevant change in associated symptoms. The determination of whether there has been a significant change will be at the discretion of the Investigator; this may be in consultation with the CT7001_001 Study Team Physician.

REFERENCES

FDA Guidance for Industry (issued July 2009) 'Drug-induced liver injury: Premarketing clinical evaluation':

http://www.fda.gov/downloads/Drugs/GuidanceComplianceRegulatoryInformation/Guidances/UCM174090.pdf

APPENDIX F – EASTERN COOPERATIVE ONCOLOGY GROUP (ECOG) PERFORMANCE STATUS

ECOG PERFORMANCE STATUS*	
**Grade**	**ECOG**
0	Fully active, able to carry on all pre-disease performance without restriction
1	Restricted in physically strenuous activity but ambulatory and able to carry out work of a light or sedentary nature, e.g., light house work, office work
2	Ambulatory and capable of all selfcare but unable to carry out any work activities. Up and about more than 50% of waking hours
3	Capable of only limited selfcare, confined to bed or chair more than 50% of waking hours
4	Completely disabled. Cannot carry on any selfcare. Totally confined to bed or chair
5	Dead

* As published in Am. J. Clin. Oncol.:
Oken, M.M., Creech, R.H., Tormey, D.C., Horton, J., Davis, T.E., McFadden, E.T., Carbone, P.P.: Toxicity And Response Criteria Of The Eastern Cooperative Oncology Group. Am J Clin Oncol 5:649-655, 1982.

APPENDIX G – MANAGEMENT OF THE CT7001_001 STUDY THROUGHOUT COVID-19 PANDEMIC.

Introduction

In early 2020 the global pandemic, COVID-19, impacted the conduct of clinical trials throughout the world. Sponsors and Investigators had to evaluate the ongoing risk to patients enrolled on clinical trials and put in place adequate measures to protect patient safety whilst, maintaining compliance with good clinical practice (GCP), and minimising loss of integrity to the clinical trial data. This appendix aims to outline the approach that Carrick Therapeutics undertook throughout this pandemic. These measures were initiated on 18th March 2020 and will continue until the COVID-19 risk subsides. All Regulatory Authority Guidance relating to managing clinical trials during COVID-19 issued by FDA, EMA and MHRA are monitored and followed.

- FDA - Guidance on Conduct of Clinical Trials of Medical Products during the COVID-19 Public Health Emergency Guidance for Industry, Investigators, and Institutional Review Boards; March 2020 and updates

- EMA - GUIDANCE ON THE MANAGEMENT OF CLINICAL TRIALS DURING THE COVID-19 (CORONAVIRUS) PANDEMIC; Version 1, 20 March 2020 and updates

- MHRA - Managing clinical trials during Coronavirus (COVID-19) - How investigators and sponsors should manage clinical trials during COVID-19; 19 March 2020 and updates

Risk of COVID-19 infection whilst on CT7001

The non-clinical and clinical safety profile of CT7001 observed to date does not indicate any substantial risk to patients continuing treatment during the COVID-19 situation. For this reason, it was assessed that all patients currently on study were benefitting from treatment and were permitted to continue receiving CT7001.

Recruitment during COVID-19

At a global level, Carrick Therapeutics assessed that limitations imposed by COVID-19 did not pose any new safety risks to trial participants and authorised all relevant modules to remain open to recruitment. However, in accordance with institutional guidance and restrictions at a local level, some Investigators took the decision not to recruit patients into clinical trials during COVID-19.

Some modules/cohorts were cancelled or delayed due to the impact of COVID-19.

No inclusion/exclusion criteria waivers were permitted and patients needed to meet protocol requirements to enter the study.

Module 1A Dose-Escalation: This cohort was complete and not impacted.

Module 1A Breast Cancer Paired Biopsy: This cohort was fully recruited and therefore recruitment was not impacted, patients continued on study during COVID-19.

Module 1A Solid Tumour Paired Biopsy: This cohort was not initiated and was subsequently cancelled. All Investigators in this module had halted recruitment to new clinical trials.

Module 1B-2 (CRPC): This cohort was fully recruited and therefore recruitment was not impacted, patients continued on study during COVID-19.

Module 1B-1 (TNBC): This cohort remained open to recruitment during the onset of COVID-19 but did not enrol and was then subsequently closed to recruitment due to an unrelated reason (Interim Efficacy analysis), patients continued on study during COVID-19.

Module 2A: This cohort remained open to recruitment during the COVID-19 period.

[redacted]

Module 4: This cohort was fully recruited and therefore recruitment was not impacted, patients continued on study during COVID-19.

Modification in patient management throughout COVID-19

As the COVID-19 situation evolved it became evident that Investigators were required to prioritize clinical resource and reduce or cancel any non-essential patient contact.

Where safe and reasonable to do so, Investigators should continue to follow the protocol schedule of events for the relevant module.

However, in the event modification to the visit schedule became necessary, Carrick Therapeutics identified the minimum patient management requirements in order to ensure patient safety throughout COVID-19.

- Any new patient must continue to undergo all mandatory screening and eligibility tests as identified in the protocol schedule of events, no inclusion/exclusion waivers will be granted.
- All mandatory screening and Cycle 1 procedures must be performed for patients newly enrolling.
- All mandatory procedures at Cycle 1 Day 15 and Cycle 2 Day 1 must be performed.
- From Cycle 3 an AE and ConMed assessment must be performed at every visit. This may be by telephone where applicable.
- From Cycle 3 haematology, biochemistry and urinalysis may be performed external to the hospital site.
- From Cycle 3, CT7001 may be dispensed to cover two cycles provided the patient continues to demonstrate compliance >75%.
- From Cycle 6, haematology, biochemistry and urinalysis may be performed at alternate cycles.
- From Cycle 12, haematology, biochemistry and urinalysis may be performed at every third cycle.

- CT, MRI and Bone Scans should continue to be performed at the protocol specified interval for M1B-1 (TNBC), M1B-2 (CRPC) and Module 2.
- For any patient with cardiac history on enrolment or taking concomitant medication that might increase the risk of cardiac symptoms, e.g. amitriptyline then ECGs must be performed according to schedule of events.

Alternative Healthcare Facilities

Carrick Therapeutics authorized the use of alternative healthcare facilities during COVID-19 to perform routine clinical assessments that may include laboratory tests, imaging scans, ECG's. These may be performed, where applicable, by a primary care physician, a private facility or by a home nursing service. Any costs that the hospital or a patient incurs will be reimbursed by the Sponsor. The impact on study data will be assessed and captured in the Clinical Study Report but it is expected to be minimal.

Home Nursing Service

Wren Healthcare Limited was contracted by Carrick Therapeutics to provide a home nursing service in the UK. The service was optional and was offered as an opt-in service for investigators and patients who were consented. This service was also supported by A4P Bio Logistics who coordinated a courier service for the delivery of temperature-controlled safety and biomarker laboratory samples.

IMP Management

A4P Bio Logistics (UK) and Almac (USA) were contracted to provide a temperature-controlled, chain of custody managed, courier collection from the dispensing pharmacy to the patient's home.

Where sites were unable to use these services due to institutional guidelines, methods of IMP delivery to patients were reviewed on a case by case basis.

Site Monitoring

On-site monitoring was not permitted throughout the peak of the COVID-19 outbreak. Monitoring plans were amended to include remote contacts and, where compliant with local regulations, remote data review.

Protocol Deviations

The COVID-19 situation is likely to increase protocol deviations. All COVID-19 related protocol deviations will be captured in the eCRF. An adaption to the database has been completed that will identify protocol deviations related to COVID-19 and these will be assessed and reported in the clinical study report.

APPENDIX H – CT7001_001 DATA MONITORING COMMITTEE NVD GUIDELINES VERSION 1.0 26-OCT-2020

DATA MONITORING COMMTTEE GUIDANCE: MANAGEMENT OF NAUSEA AND/OR VOMITING AND/OR DIARRHOEA

Introduction

CT7001_001 is a modular protocol enrolling patients with advanced solid tumours onto either monotherapy CT7001 or in combination with standard care, as applicable to the module. The first patient was enrolled onto CT7001_001 on 20-Nov-2017 and at the introduction of this guidance document, in excess of 100 patients have received treatment with CT7001; with recruitment ongoing it is anticipated approximately 250 patients will be exposed to CT7001 throughout the lifecycle of the protocol.

Nausea, vomiting and diarrhoea have been reported at high incidence (70-80%) by patients as adverse events since the outset. Although these symptoms are primarily mild and able to be managed adequately there is a smaller cohort of patients (~6%) who have been unable to tolerate treatment with CT7001. Please refer to the current Investigator Brochure for a breakdown of grades, occurrence and outcomes.

The Data Monitoring Committee (DMC) and Safety Review Committee (SRC) continue to monitor nausea, vomiting and diarrhoea closely in an effort to characterise the symptoms and provide guidance to treating physicians and the patients in relation to best management protocols.

This guidance document, supporting protocol CT7001_001, has been produced following clinical input from the CT7001_001 DMC, following consultation with the Experimental Cancer Medicines Centres (ECMC) Research Nurses Network Group and in accordance with National Comprehensive Cancer Network (NCCN) Guidelines.

In parallel, Carrick Therapeutics is planning to investigate the impact of an enteric capsule formulation on the incidence and severity of nausea, vomiting and diarrhoea in a solid tumour population.

The following mitigation steps are required:

1. Discuss nausea, vomiting and diarrhoea symptoms and management with all newly enrolling patients.

2. A Nausea, Vomiting and Diarrhoea Information Leaflet is currently being produced for submission to IEC/IRBs. Once approved, please issue a copy to all new patients at Cycle 1 Day 1.

3. Patients should be given an adequate supply of both anti-emetics and anti-diarrhoeal medication to take home with them. They should be instructed to take the relevant medication based on their symptoms; there is no need to seek approval from the Investigator.

    a. Anti-emetic therapy: A serotonin (5-HT3) antagonist should be dispensed (NCCN Antiemesis Guidelines v2;2017).
    b. Anti-diarrhoeal therapy: patients should be dispensed loperamide.

4. Anti-emetic therapy will be given prophylactically before all doses, including C1D1.

5. Anti-diarrhoeal therapy will be initiated immediately on first symptoms.

6. Prophylactic therapy can be considered for withdrawal once a patient is established on treatment. Otherwise management is directed by clinical judgement of a patient's symptoms and signs.

7. The following additional steps may also be of benefit:
   - Advise patients to take their CT7001 just prior to bed or rest for approximately 60 minutes after taking CT7001 as this may reduce the severity of nausea.
   - Consumption of a small meal / food approximately 30 minutes prior to taking CT7001 may reduce the severity of nausea.
   - Explore techniques to help patients manage ongoing stress or anxiety as this has been reported to exacerbate gastrointestinal symptoms.

Patients should be instructed to contact the investigator immediately if they experience symptoms of nausea, vomiting or diarrhoea, even if mild.

1. Patients should also be encouraged to drink plenty of fluid

2. Investigators should follow up with the patient by telephone within 24h to assess response. Thereafter regular contact should be made to monitor symptoms and to ensure optimal therapy is being received.

3. If N/V is not optimally controlled on anti-emetics alone then consider use of an oral proton pump inhibitor

4. Dietetic measures to control diarrhoea: stop all lactose containing products, drink 8 to 10 large glasses of clear liquids per day (fluid intake of ~2L per day should be maintained), eat frequent small meals, recommend low fat diet enriched with banana, rice, apple sauce and toast i.e. BRAT diet

5. For diarrhoea it is important that patients take a full dose of loperamide, alternative anti-diarrheals such as Lomotil, codeine and ocreotide can also be considered as secondary therapy

6. If symptoms persist for >5 days at Grade 2 or >2 days at Grade 3+ then the Investigator should contact the study medical monitor.

7. Investigators should consider if patients experiencing anticipatory nausea/psychosomatic symptoms may benefit from short (up to 7 days) treatment breaks as appropriate, especially in the first two cycles, to allow for resolution of symptoms and changes to management protocols prior to reinitiating CT7001 therapy.

8. The protocol also allows for dose adjustments of CT7001 to 300mg daily and then 240mg daily

The above information is provided as a guideline based on the clinical experience with CT7001 to date, at all times the Investigator retains full control of the appropriate medical management of their patient.

It is important to note that Aprepitant (Emend®) is a substrate, moderate inhibitor and inducer of CYP3A4 and an inducer of CYP2C19. Therefore, aprepitant should not be used in this study for the treatment of nausea and vomiting.

CT7001_001 MODULE 2 PROTOCOL

PROTOCOL REDACTED TO REMOVE DETAILS OF OTHER MODULES NOT RELEVANT TO THIS MANUSCRIPT

STUDY CT7001_001 VOLUME 4, MODULE 2

in

HORMONE RECEPTOR-POSITIVE, HUMAN EPIDERMAL GROWTH FACTOR RECEPTOR-NEGATIVE METASTATIC BREAST CANCER

**Title of Core Study CT7001_001:**	A Modular, Multipart, Multi-arm, Open-label, Phase 1/2a Study to Evaluate the Safety and Tolerability of CT7001 Alone and in Combination with Anti-Cancer Treatments in Patients with Advanced Malignancies
**Title of Volume 4, Module 2:**	A Phase 1/2 Study of CT7001 in Combination with Fulvestrant in Patients with Metastatic or Locally Advanced Hormone-Receptor-Positive and Human Epidermal Growth Factor Receptor 2-Negative Breast Cancer
**Study Number:**	CT7001_001
**EudraCT Number:**	2017-002026-20
**ClinTrials.gov ID:**	NCT03363893
**Study Phase:**	Phase 1/2
**Investigational Products:**	CT7001 (Samuraciclib), fulvestrant [redacted]
**Indication:**	Treatment of metastatic or locally advanced hormone-receptor-positive and human epidermal growth factor receptor 2-negative breast cancer in patients who had previously received an aromatase inhibitor and a CDK4/6 inhibitor
**Sponsor:**	Carrick Therapeutics<br>NovaUCD,<br>Belfield Innovation Park,<br>University College Dublin,<br>Belfield,<br>Dublin 4, Ireland
**Medical Monitor:**<br><br>**Module 2 Part A**	[redacted]<br>Bionical Emas<br>63-65 Knowl Piece,<br>Wilbury Way, Hitchin<br>Hertfordshire, SG4 0TY, UK<br>Telephone: [redacted]<br>E-mail: [redacted]
[redacted]<br><br>[redacted]	[redacted]
**Date of Original Protocol**	Version 2.0, 6 March 2019<br>*(Version 1.0, 15 January 2019 – Protocol not implemented)*

**Amendment**	Version 3.0, 26 March 2019<br>*(Version 4.0, 29 November 2019 – Protocol not implemented)*<br>Version 5.0, 30 January 2020<br>Version 6.0, 27 April 2020<br>Version 7.0, 15 July 2020<br>Version 8.0, 23 December 2020<br>Version 9.0, 14 June 2021

Confidentiality Statement

The information in this document is confidential and will not be disclosed to others without written authorisation from the Sponsor, except to the extent necessary to obtain informed consent from persons receiving the study drug or their legal guardians, or for discussions with Regulatory Authorities, Institutional Review Boards, Ethics Committees, or persons participating in the conduct of the study. Do not copy or distribute without written permission from the Sponsor.

PROTOCOL APPROVAL PAGE

DocuSigned by: [redacted] Signing Reason: I approve this document Signing Time: 21-Jun-2021 | 11:42 BST 94C0D49A78C748F09D998E746FA969BE<br>[redacted]<br>Carrick Therapeutics, Senior Medical Director	Date: 21-Jun-2021 | 11:42 BST
DocuSigned by: [redacted] Signing Reason: I approve this document Signing Time: 21-Jun-2021 | 16:35 BST 6768291FC8264A8EA78347C3E990EF4A<br>[redacted]<br>National Co-ordinator for Overall Modular Study	Date: 21-Jun-2021 | 16:35 BST
DocuSigned by: [redacted] Signing Reason: I approve this document Signing Time: 21-Jun-2021 | 04:07 MDT E9BE010FA0D649B891783A4ECE97B3DA<br>[redacted]<br>Principal Investigator for Module 2	Date: 21-Jun-2021 | 04:07 MDT
DocuSigned by: [redacted] Signing Reason: I approve this document Signing Time: 21-Jun-2021 | 10:58 BST D27C397C775641A9A89360FD282B30C0<br>[redacted]<br>Carrick Therapeutics, Biostatistician	Date: 21-Jun-2021 | 10:58 BST

TABLE OF CONTENTS

PROTOCOL APPROVAL PAGE **3**
TABLE OF CONTENTS **4**
INVESTIGATOR SIGNATURE PAGE **10**
LIST OF ABBREVIATIONS **11**
SYNOPSIS OF STUDY CT7001_001, Volume 4, Module 2 .... **14**
STUDY PROTOCOL OF CT7001_001, Module 2 .... **31**
1 INTRODUCTION **31**
2 BACKGROUND AND RATIONALE **31**
2.1 Hormone Receptor-Positive Breast Cancer 31
2.2 CDK7 33
2.3 CT7001 33
2.3.1 Overview of Nonclinical Data 33
2.3.2 Current Clinical Data of CT7001 35
2.4 Fulvestrant 42
2.5 Potential for Drug-Drug Interaction (DDI) Between CT7001 and Fulvestrant 44
3 STUDY OBJECTIVES AND ENDPOINTS **45**
3.1 Objectives 45
3.2 Endpoints 46
4 STUDY POPULATION **47**
4.1 Inclusion Criteria 47
4.2 Exclusion Criteria 49
5 STUDY DESIGN **53**
5.1 Part A 54
5.2 Part B 56
5.2.1 Randomization Procedure 57
5.3 Part C 57
6 STUDY TREATMENT **58**
6.1 Drug Supply 58
6.1.1 Pharmaceutical Properties of CT7001 58
6.1.2 Pharmaceutical Properties of Fulvestrant 58
6.1.3 Formulation, Packaging and Storage of CT7001, [redacted] and Fulvestrant 58
6.2 Dispensing of CT7001 [redacted] 60
6.3 Dosing Regimen of CT7001 [redacted] 60
6.4 Drug Administration of CT7001 [redacted] 60
6.5 General Rules for CT7001 [redacted] 60

6.6 Medication Dosing Errors of CT7001 [redacted] 61
6.7 Dosing Regimen and Drug Administration of Fulvestrant....61
6.8 Drug Diary for LHRH agonist....61
6.9 Overdose of CT7001[redacted] 61
6.10 Overdose of Fulvestrant....61
6.11 Recording of Overdose....62
6.12 Dose Modification....62
6.12.1 Fulvestrant....62
6.12.2 Types of Dosing Modifications of CT7001[redacted] 63
6.13 Compliance....65
6.14 Drug Storage of Investigational Products (IPs) and Accountability....66
6.15 Fulvestrant Monotherapy....66
6.16 Supply of Investigational Product Post Study....66
6.17 Concomitant Medications....67
6.17.1 Prohibited Medications....67
6.17.2 Medications Not Recommended....68
6.17.3 Recommended and Permitted Treatments....69
6.18 Contraception....71
6.19 Concomitant Radiotherapy or Surgery....71
7 STUDY PROCEDURES....72
7.1 Screening....72
7.1.1 Screen Failure....73
7.2 Active Treatment Phase....73
[redacted]
7.4 End of Treatment Visit....73
7.5 Follow-up Phase....74
7.6 Patient Withdrawal....74
7.6.1 Active Treatment Phase Discontinuation....74
7.6.2 Post-Treatment Study Discontinuation....75
7.7 Study Completion....76
8 STUDY ASSESSMENTS....76
8.1 Tumour Assessments....76
8.1.1 Screening/Baseline....77
8.1.2 Post-Baseline....77
8.1.3 Independent Review of Disease Assessments....79
8.2 Safety Assessments....79
8.2.1 Laboratory Safety Assessments....79
8.2.2 Electrocardiogram (ECG)....80
8.2.3 Other Safety Assessments....81

8.3 Pharmacokinetic Assessments 81
8.4 Assessment of CYP and drug transporter genes 81
8.5 Biomarker Assessments 82
8.5.1 ER, PgR and HER2 82
8.5.2 Exploratory Biomarkers 82
8.5.3 Tumour Biopsies 83
8.5.4 Archival Tumour Tissue 83
8.5.5 Retained Samples for Future Exploratory Research 83
8.5.6 Retained Sample for Potential Future Pharmacogenomic Research 84
9 ADVERSE EVENT REPORTING 84
9.1 Reporting Period 85
9.2 Definition of Adverse Event 85
9.3 Definition of Adverse Event of Special Interest 86
9.4 Definition of Serious Adverse Events 86
9.4.1 Protocol-Specified Serious Adverse Events 87
9.4.2 Potential Cases of Drug-Induced Liver Injury (Hy's Law Cases) 88
9.5 Hospitalisation 89
9.6 Severity Assessment 90
9.7 Causality Assessment 90
9.8 Exposure During Pregnancy 91
9.9 Occupational Exposure 91
9.10 Withdrawal Due to Adverse Events (See Also Section on Patient Withdrawal) .. 92
9.11 Eliciting Adverse Event Information 92
9.12 Reporting Requirements 92
9.12.1 Serious Adverse Event Reporting Requirements 92
9.12.2 Non-Serious Adverse Event Reporting Requirements 93
9.12.3 Sponsor's Reporting Requirements to Regulatory Authorities 94
10 DATA ANALYSIS AND STATISTICAL METHODS 94
10.1 Analysis Populations 94
10.1.1 Intent-to-Treat (ITT) Population 94
10.1.2 As-Treated (AT) Population 94
10.1.3 Evaluable for Response Population 94
10.1.4 Pharmacokinetics (PK) Population 94
10.2 Efficacy Analysis 94
10.2.1 Analysis of Progression-Free Survival 95
10.2.2 Objective Response 96
10.2.3 Duration of Response 97
10.2.4 Clinical Benefit Response 97
10.2.5 Percent Change in Tumour Size 97
10.2.6 Waterfall Plot Analysis 97
10.3 Safety Analysis 98

10.3.1 Adverse Events (AEs) 98
10.3.2 Laboratory Abnormalities 98
10.3.3 Electrocardiogram (ECG) Analysis 98
10.4 Pharmacokinetic Analysis 98
10.5 Biomarker Analysis 99
10.6 Analysis of Other Endpoints....99
11 ETHICS 99
11.1 Institutional Review Board (IRB)/Independent Ethics Committee (IEC)....99
11.2 Ethical Conduct of the Study....99
11.3 Patient Information and Confidentiality....100
11.4 Patient Consent....100
11.5 Potential Risks and Benefits....100
11.5.1 Potential Risks....100
11.5.2 Potential Benefits 102
11.5.3 Overall Risk/Benefit Assessment....103
11.6 Patient Recruitment 104
11.7 Reporting of Safety Issues and Serious Breaches of the Protocol or ICH GCP..104
12 QUALITY CONTROL AND QUALITY ASSURANCE 104
13 DATA HANDLING AND RECORD KEEPING....104
13.1 Case Report Forms/Electronic Data Record....104
13.2 Record Retention 105
14 DEFINITION OF END OF TRIAL 106
14.1 End of Trial in all Participating Countries....106
14.2 End of Trial in a Member State of the European Union....106
15 EARLY DISCONTINUATION OF TRIAL....106
16 ADMINISTRATIVE PROCEDURES AND CONSIDERATIONS....106
16.1 Data Monitoring Committee....106
16.2 Protocol Amendments 107
16.3 Clinical Study Report 107
16.4 Financing and Insurance....107
17 PUBLICATION OF STUDY RESULTS 107
17.1 Communication of Results by Carrick 107
17.2 Publications by Investigators....108
18 SCHEDULE OF ASSESSMENTS 109
REFERENCES....115
APPENDIX A – DRUGS KNOWN TO PREDISPOSE TO TORSADE DE POINTES 120

APPENDIX B - MEDICATIONS, HERBAL SUPPLEMENTS, AND FOODS THAT SIGNIFICANTLY INDUCE OR INHIBIT CYTOCHROME P450 3A4, 2C19 AND/OR 2D6 OR P GLYCOPROTEIN ACTIVITY 121

APPENDIX C – RESPONSE EVALUATION CRITERIA IN SOLID TUMOURS (RECIST) VERSION 1.1 124

APPENDIX D – NEW YORK HEART ASSOCIATION (NYHA) FUNCTIONAL CLASSIFICATION 129

APPENDIX E – US FDA LABEL, EU SPC AND EPAR ASSESSMENT REPORT OF FULVESTRANT 130

APPENDIX F – EASTERN COOPERATIVE ONCOLOGY GROUP (ECOG) PERFORMANCE STATUS 131

APPENDIX G – MANAGEMENT OF THE CT7001_001 STUDY THROUGHOUT COVID-19 PANDEMIC. 132

APPENDIX H – CT7001_001 DATA MONITORING COMMITTEE NVD GUIDELINES VERSION 2.0 08-JUN-2021 135

LIST OF TABLES

Table 1: Status of Modules 1A, 1B, 2 and 4 as of 07-May-2021 37
Table 2: Content of CT7001 Capsules 59
[redacted]
Table 4: Two Dose Reduction Levels for Two Potential Starting Doses of CT7001 [redacted] 65
Table 5: Number and Strength of Capsules for Each Possible Dose 65
Table 6: Schedule of Events 109
Table 7: Blood Volumes 114

LIST OF FIGURES

Figure 1: CT7001 decreases pPolII levels in PBMC. 40

INVESTIGATOR SIGNATURE PAGE

CT7001_001 Volume 4, Module 2 (A Phase 1/2 Study of CT7001 in Combination with Fulvestrant in Patients with Metastatic or Locally Advanced Hormone-Receptor-Positive and Human Epidermal Growth Factor Receptor 2-Negative Breast Cancer)

I have read the Core Study Protocol (Volume 1) and Volume 4 (the protocol for Module 2) and agree to conduct the trial in compliance with the International Council for Harmonisation Guideline for Good Clinical Practice and applicable regulatory requirements and to inform all who assist me in the conduct of this trial of their responsibilities and obligations.

Signed: ____________________________ Date: _________________________

Print Name: ________________________

LIST OF ABBREVIATIONS

Abbreviation	Definition
AE	Adverse event
AESI	Adverse Event of Special Interest
AI	Aromatase inhibitors
ALT	Alanine aminotransferase
AR	Androgen receptor
ASCO	American Society of Clinical Oncology
AST	Aspartate aminotransferase
AT	As-treated
BC	Breast cancer
BICR	Blinded independent central review
BID	Twice a day
CBR	Clinical benefit response
CI	Confidence interval
CISH	Chromogenic in situ hybridization
CMH	Cochran-Mantel-Haenszel test
c-myc	Proto-oncogene
CNS	Central nervous system
CR	Complete response
CRO	Contract Research Organisation
CT	Computed tomography
CTCAE	Common Terminology Criteria for Adverse Events
CYP	Cytochrome P450
DDI	Drug-drug interaction
DLT	Dose-limiting toxicities
DMC	Data Monitoring Committee
DNA	Deoxyribonucleic acid
DOR	Duration of response
D/N/V	Diarrhoea and/or Nausea and/or Vomiting
ECG	Electrocardiogram
ECOG	Eastern Cooperative Oncology Group
eCRF	Electronic Case Report Form
EPAR	European Public Assessment Report

ER	Oestrogen receptor
ESMO	European Society of Medical Oncology
ESR1	Oestrogen receptor 1
FDA	Food and Drug Administration
FFPE	Formalin-fixed paraffin embedded
FISH	Fluorescent in situ hybridization
FSH	Follicle stimulating hormone
G-CSF	Granulocyte colony stimulating factor
HbA1c	Glycated haemoglobin
HER2	Human epidermal growth factor receptor 2
hERG	Human ether-a-go-go-related gene
HR	Hazard ratio
HR	Hormone receptor
IB	Investigators Brochure
IC_{50}	Half maximal inhibitory concentration
ICH	International Council for Harmonization
IDMC	Independent Data Monitoring Committee
IEC	Independent Ethics Committee
IM	Intramuscularly
INR	International normalized ratio
IP	Investigational product
IRB	Institutional Review Board
ITT	Intent-to-treat
IV	Intravenous
IXRS	Interactive response system
LHRH	Luteinizing hormone-releasing hormone
MCL1	Induced myeloid leukemia cell differentiation protein
MED1	Mediator of RNA polymerase II transcription subunit 1
MedDRA	Medical Dictionary for Regulatory Activities
MMRM	Mixed model repeated measures
MRI	Magnetic resonance imaging
MTD	Maximum tolerated dose
mTOR	Mammalian target of rapamycin
NCCN	National Comprehensive Cancer Network

NCI	National Cancer Institute
OD	Once daily
OR	Objective response
ORR	Objective response rate
PBMCs	Peripheral blood mononuclear cells
PD	Progression of disease
PDc	Pharmacodynamics
PFS	Progression-free survival
PGP	P-glycoprotein
PgR	Progesterone receptor
PIK3CA	Phosphatidylinositol 4,5-bisphosphate 3-kinase catalytic subunit alpha
PK	Pharmacokinetics
PolII	RNA Polymerase II
pPolII	Phosphorylated RNA Polymerase II
PR	Partial response
p53	Tumour suppressor p53
QTcF	Fridericia formula
Rb	Retinoblastoma
RECIST	Response Evaluation Criteria In Solid Tumours
RNA	Ribonucleic acid
RP2D	Recommended Phase 2 dose
SAE	Serious adverse event
SAP	Statistical Analysis Plan
SD	Stable Disease
SISH	Silver-enhanced in situ hybridization
TNBC	Triple-negative breast cancer
ULN	Upper limit of normal

SYNOPSIS OF STUDY CT7001_001, VOLUME 4, MODULE 2

**Sponsor**: Carrick Therapeutics	
**Study Title**: A Phase 1/2 Study of CT7001 in Combination with Fulvestrant in Patients with Metastatic or Locally Advanced Hormone-Receptor-Positive and Human Epidermal Growth Factor Receptor 2-Negative Breast Cancer	
**Study Number:** CT7001_001	**Study Phase:** Phase 1b/2
**EudraCT Number:** 2017-002026-20	**ClinTrials.Gov ID:** NCT03363893
**Volume 4, Module 2 Study Objectives**	

<u>Study Design</u>

The present study is an international, multicentre Phase 1/2 study in patients with metastatic or locally advanced hormone receptor (HR)-positive and human epidermal growth factor receptor 2 (HER2)-negative breast cancer (BC). The study will have three parts. In each part, patients must meet all the eligibility criteria described in Section 4 and will receive study treatment until objective disease progression (Section 8.1.2), symptomatic deterioration, unacceptable toxicity, death, withdrawal of consent or completion of Module 2 primary endpoint, whichever occurs first.

The overall study design is illustrated in the following schema.

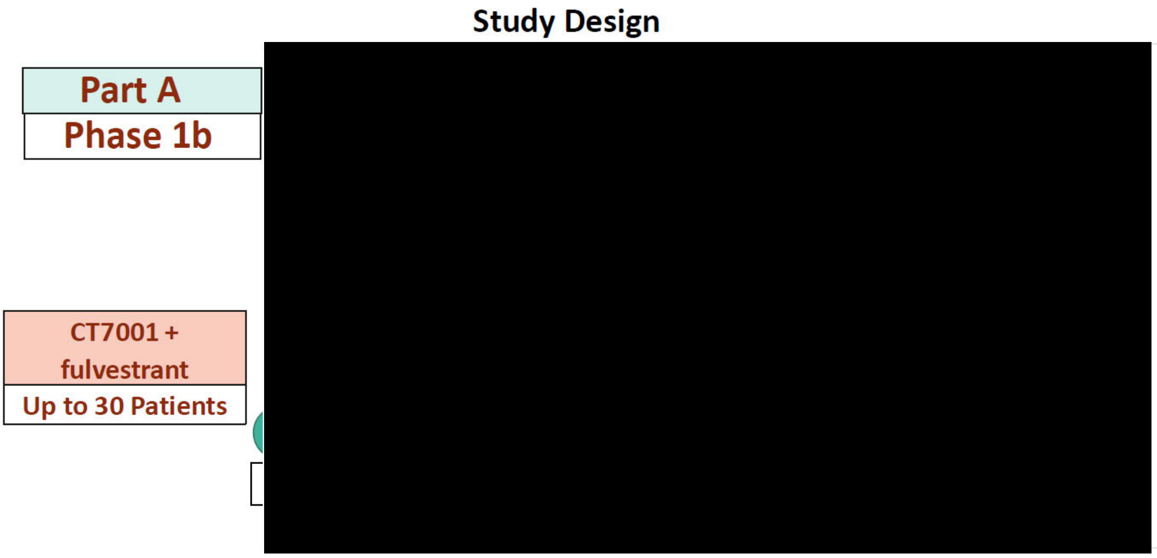

In each part of the study a treatment cycle will be defined as 28 days. CT7001 [redacted] will be administered orally once a day (OD). Module 4 evaluated the effect of food on the bioavailability of CT7001 in cancer patients. On 10th June 2019 the Safety Review Committee reviewed the PK data and determined there was no significant effect observed in AUC in the fed phase of the Module 4, the requirement to fast was removed.

CT7001 may be taken orally in either a fasted or fed state, where a patient experiences nausea or vomiting Investigators are recommended to advise their patients to consume CT7001 after a meal.

Throughout the study the dose of fulvestrant will be fixed at the standard dose of 500 mg administered as intramuscularly (IM) injections at intervals of 28 ± 2 days with an additional 500 mg dose given 14 ± 2 days after the first dose. Pre-and peri-menopausal women must have commenced treatment with a luteinizing hormone-releasing hormone (LHRH) agonist at least 4 weeks prior to first dose of CT7001[redacted]. Every effort should be made to administer an LHRH agonist on site at the time of fulvestrant administration. LHRH agonist is not considered as an investigational product in this study but rather a mandatory auxiliary treatment in the subgroup of patients who are pre- or peri-menopausal.

The DMC will monitor the safety and efficacy data on a periodic basis. The DMC will make recommendation as to whether the trial should continue based on ongoing reviews of safety data. In Part A, the DMC approved second CT7001 dosing cohort on 07 February 2020 as no Dose Limiting Toxicities were observed in the first cohort. [redacted]

Patients will undergo regular safety and efficacy assessments as outlined in the Schedule of Events. Primary efficacy analyses will be performed based on the local radiologist's/investigator's tumour assessments, using Response Evaluation Criteria in Solid Tumours (RECIST) version 1.1. If required, supportive efficacy analyses may be performed by blinded independent central review (BICR). Efficacy analyses in this study will not include survival.

Tumour assessments will continue until radiographically and/or clinically (i.e., for photographed or palpable lesions) documented progressive disease as per RECIST version 1.1 (Section 8.1.2), death, discontinuation of patient from overall study participation (e.g., patient's request, lost to follow-up), initiation of new anticancer therapy (Part A [redacted]) or Module 2 LPLV is met, whichever occurs first. [redacted]

All parts of the study will include sparse blood sampling for analysis of CT7001 and fulvestrant trough concentrations. Various members of the cytochrome P450 (CYP) family play a role in the hepatic metabolism of CT7001, and drug transporters may affect bioavailability. Accordingly, genotyping of CYP and drug transporter genes will be considered a mandatory study procedure.

All parts of the study will include a set of optional procedures and analyses which require separate informed consent. These include baseline biopsies of readily accessible tumour lesions to analyse various gene mutations, ribonucleic acid (RNA) expression profiles and expression of various proteins and their potential impact on efficacy. [redacted] In all parts of the study baseline blood samples will be collected for various molecular analysis, some to be retained for potential future research and pharmacogenomic testing, respectively (unless prohibited by local laws or regulations).

Patients will be given the opportunity to provide feedback in relation to their clinical trial experience at the end of their study participation.

Part A

Part A is an open-label, single-arm, ascending dose Phase 1b study to determine the dosing regimen of CT7001 and fulvestrant to be taken to subsequent randomized Phase 2 testing in Part B. Before taking a particular dosing regimen to Part B, at least 6 patients in Part A should have received that regimen and at least 3 patients should have completed ≥ 2 cycles. The decision algorithm described below is based on dose-limiting toxicities (DLTs) recorded in the first cycle. Patients are considered evaluable, if they completed the first cycle or discontinued therapy in the first cycle due to DLT. Patients in a cohort will be replaced if they cannot complete the first cycle unless due to a DLT.

Part A is planned to have two dose cohorts with up to 6 evaluable patients to be enrolled per cohort to determine the dose to take to part B and/or expand the cohort. In each cohort, the dose of fulvestrant will be fixed at the standard dose of 500 mg given at intervals of 28 ± 2 days with an additional 500 mg dose given 14 ± 2 days after the first dose. Fulvestrant will be administered as two consecutive slow IM injections (1-2 minutes) of 250 mg in 5 mL, one in each buttock (gluteal area).

Cohort 1 will test CT7001 at 240 mg OD, which is approximately 33% lower than the preliminary recommended Phase 2 dose as monotherapy (360 mg). In case no DLT is recorded in Cycle 1 in the first 3 evaluable patients, cohort 2 will commence enrolment at 360 mg of CT7001 OD. In case of 1/3 patients have a DLT in cohort 1, a further 3 evaluable patients will be assessed. If < 2/6 patients will have a DLT, cohort 2 will start enrolment.

In case ≥ 2/3 or ≥ 2/6 patients in cohort 1 experience a DLT, dose escalation MUST be stopped. The DMC will then determine if patients in Part A and in Part B can only be administered a lower dose, or a different dosing regimen. A new, lower dose, or a justification for a different dosing regimen at the same dose MUST require approval of a substantial amendment.

In case 0/3 or 1/3 evaluable patients in cohort 2 experience a DLT, the cohort will expand to six patients. In case ≥ 2/3 or ≥ 2/6 patients experience a DLT, the cohort 1 dosing regimen (CT7001 at 240 mg and fulvestrant at 500 mg) will be determined as the preliminary Phase 2 dosing regimen. However, before taking to Part B, at least 6 patients should be treated with that regimen in Part A and at least 3 should have completed at least 2 cycles.

In case < 2/6 evaluable patients in cohort 2 have a DLT and at least 3 patients have completed at least 2 cycles, this will be considered the recommended Phase 2 regimen and taken to Part B testing.

The DMC will determine whether and when to open the second CT7001 dosing cohort and which dosing regimen to advance to randomized Phase 2 testing in Part B. In case the DMC may consider safety and/or tolerability data as being borderline for taking a dosing regimen to Part B, additional patients in sets of 3 may get enrolled in Part A for further assessment.

Part A is estimated to require approximately 12 patients who completed Cycle 1 to confirm the recommended phase 2 dose (RP2D) to progress to Part B. Following RP2D selection by the Data Monitoring Committee (DMC) additional patients will be enrolled into Module 2A

which is anticipated to yield 20 evaluable patients at the RP2D. It is anticipated that 25-30 patients overall will be enrolled into Module 2A.

Module 2 Part A completed enrolment on 23 March 2021 with 31 patients dosed. [redacted]

[redacted]

[redacted]

[redacted]

[redacted]

[redacted]

[redacted]

[redacted]

- [redacted]
- [redacted]
  - [redacted]

[redacted]

- [redacted]
- [redacted]
- [redacted]
- [redacted]
- [redacted]

[redacted]

Primary Objective, Part A

- To determine the recommended Phase 2 dose of CT7001 given in combination with fulvestrant at 500 mg.

[redacted]

Secondary Objectives

- To evaluate safety and tolerability (all study parts).
- To evaluate the trough concentrations of CT7001 when used in combination with fulvestrant compared to historical CT7001 data (all study parts).
- To evaluate correlations between CT7001 exposures and efficacy/safety findings in this patient population (all study parts).
- [redacted]
- To evaluate the incidence and type of genotypes and copy number variation of CYP2D6 and other CYP genes and of drug transporter genes that may be involved in the metabolism of CT7001 (all study parts).

Exploratory Objectives (all study parts)

- To further investigate the presence and identity of metabolites of CT7001 and, if appropriate, characterize their pharmacokinetics (PK).

- To further evaluate the impact of patient characteristics (including but not limited to race, ethnicity, age, CYP and drug transporter polymorphisms) on trough concentrations of CT7001 (all study parts).
- To evaluate correlations between CT7001 exposures and efficacy/safety findings in this patient population (all study parts).
- To further explore mutations and expression in genes, proteins and RNAs relevant to the cell cycle (e.g., phosphorylation of CDK1 and retinoblastoma (Rb) proteins), drug target engagement (e.g., c-Myc, MCL-1) and tumour sensitivity and/or resistance in tumour-derived materials including circulating tumour DNA and tumour tissue (e.g., p53, CDK7, ER, ESR1, AR, PIK3CA) and their potential impact on efficacy.

Module 2 Study Centres: Centres will be located, in the United Kingdom, the USA, [redacted]

Number of Patients Planned: [redacted]

Inclusion Criteria

To be eligible for the study patients must meet **all** the following criteria:

1. Women 18 years of age or older who are either:
   - o Postmenopausal, as defined by one of the following criteria:
     - Age ≥60 years;
     - Age 50-59 years of age amenorrhoeic for at least 12 months after cessation of all exogenous hormonal treatments, with no alternative pathological or physiological cause <u>or</u> have serum oestradiol and follicle stimulating hormone (FSH) level within the laboratory's reference range for postmenopausal females;
     - Age <50 years of age amenorrhoeic for at least 12 months following cessation of exogenous hormonal treatments, with no alternative pathological or physiological cause <u>and</u> serum oestradiol and follicle stimulating hormone (FSH) level within the laboratory's reference range for postmenopausal females;
     - Documented bilateral oophorectomy;
     - Medically confirmed ovarian failure.

     OR
   - o Pre/peri-menopausal, i.e., not meeting the criteria for being postmenopausal, if amenable to treatment with an LHRH agonist.
     - LHRH agonist is considered a mandatory auxiliary treatment in this sub-population and is to be given in accordance with the locally approved label of the drug and routine institutional practice. Patients must have commenced treatment with an LHRH agonist at least 4 weeks prior to first dose of CT7001 [redacted]

2. No childbearing potential, defined as women:
    - Who had prior hysterectomy or bilateral surgical oophorectomy or are medically postmenopausal (as defined in inclusion criteria 1).
3. Women of childbearing potential must be willing to practice effective contraception (defined as abstinence, sex only with person of the same sex, sex only with vasectomized partner, intrauterine device, or barrier method [e.g., condom, diaphragm] for the duration of the study and for 24 months after the last study dose.
    - The drug-drug interactions of CT7001 are not fully characterized so hormonal contraceptives that are prone to drug-drug interactions may not be effective because of a potential CYP3A4 interaction with CT7001.
4. Women of childbearing potential must have a negative serum pregnancy test at baseline (within 7 days prior to first dose of CT7001[redacted]).
5. Histologically confirmed diagnosis of carcinoma of the breast with evidence of metastatic or locally advanced disease, not amenable to resection or radiation therapy with curative intent.
6. Documentation of oestrogen receptor (ER)-positive and/or progesterone receptor (PgR)-positive tumour based on most recent tumour biopsy utilizing an assay consistent with local standards.
    - ER- and PgR-positivity is defined as ≥1% positive stained cells (American Society of Clinical Oncology/College of American Pathologists (ASCO/CAP) guidelines, Hammond et al., 2010).
7. Documentation of HER2 negativity based on local testing on most recent tumour biopsy.
    - HER2-negativity is defined as immunohistochemistry score 0/1+ or negative by in situ hybridization (FISH/CISH/SISH) defined as a HER2/CEP17 ratio <2 or for single probe assessment a HER2 copy number <4 (ASCO/CAP guidelines, Wolff et al., 2018).
    - In case no tumour biopsy was performed after initial resection of the primary tumour, the original tumour tissue serves as basis for assessment of ER/PgR and HER2 status.
    - Assessment of ER, PgR and HER2 status will be based on results from local pathology laboratories. Independent central review is not intended.
8. **Part A Only:** Measurable disease as defined by RECIST version 1.1 (Appendix C).
9. Patients must have documented objective disease progression while on or within 6 months after the end of the most recent therapy.
    - [redacted]

10. Patients must have received an aromatase inhibitor together with a CDK4/6 inhibitor in the same line of therapy for the treatment of:

    - locally advanced or metastatic disease or
    - early breast cancer, if the disease-free interval, between initiation of adjuvant therapy and first line treatment of locally advanced or metastatic disease was < 12 months.

    In addition, the following prior therapies for locally advanced or metastatic disease are allowed:

    - Everolimus (Part A Only)
    - No more than two lines of endocrine treatment (prior fulvestrant is not allowed)
    - No more than one line of prior chemotherapy for locally advanced or metastatic disease (Part A Only).

11. [redacted]

12. Eastern Cooperative Oncology Group (ECOG) performance status 0 or 1 with no deterioration over the previous 2 weeks (see Appendix F).
13. Expected life expectancy of greater than 12 weeks.
14. Ability to swallow and retain oral medication and receive intramuscular injections.
15. Patients are willing and able to comply with scheduled visits, treatment plan, laboratory tests, and other study procedures.
16. Evidence of personally signed and dated written informed consent indicating that the patient has been informed of all pertinent aspects of the study before any study-specific activity is performed.

Host Genetics Research Study - Pharmacogenomics Samples (Optional)

Patients who meet all of the following criteria may be included in optional genetics sub-studies:

1. Provision of signed and dated, written informed consent for the genetic research.

Exclusion Criteria

To be eligible for the study patients **may not have any** of the following exclusion criteria:

1. Prior therapy with fulvestrant.

- [redacted]

2. More than 2 lines of endocrine treatment for locally advanced or metastatic disease.
3. [redacted]

[redacted]

5. Patients with liver metastasis may only be enrolled upon approval by the medical monitor:
    - **Part A:** On 11th September 2020 the DMC approved the request to not enrol further patients with liver metastasis into Part A
    - [redacted]
6. Inadequate hepatic, renal, bone marrow or cardiac function, specified as follows:
    - Hepatic (any of below):
        - Aspartate aminotransferase (AST) and/or alanine aminotransferase (ALT) > 2.5 × the upper limit of normal (ULN) or > 5.0 × ULN for patients with liver metastases.
        - Alkaline phosphatase > 2.5 x ULN or > 5 x ULN if bone or liver metastases present.
        - Total bilirubin > 1.5 × ULN (> 3.0 x ULN if known Gilbert's disease).
        - Albumin < 30 g/L.
        - Liver function deteriorating at a speed that would likely make the subject not meeting the AST, ALT, bilirubin or albumin levels specified above at the time of the first study dose.
        - Other evidence of impaired hepatic synthesis function.
    - Renal:
        - Serum creatinine > 1.5 × ULN.
    - Bone marrow (any of below):
        - Absolute neutrophil count ≤ 1.5 × 10^9/L (≤ 1.500/mm^3).
        - Platelet count <100 × 10^9/L (< 100,00/mm^3).
        - Haemoglobin < 90 g/L (< 9 g/dL).
    - Cardiac (any of below):
        - Myocardial infarction within 6 months of study entry, unstable angina, unstable arrhythmia.
        - New York Heart Association Class > I heart failure (see Appendix D).

  - Mean resting QT interval corrected for heart rate by the Fridericia formula (QTcF) > 470 msec obtained from 3 electrocardiograms (ECGs) obtained within 3-5 minutes apart.
  - Clinically important abnormalities in rhythm, conduction, or morphology on resting ECG (e.g., complete left bundle branch block, third degree heart block).
    - Controlled atrial fibrillation is permitted.
  - Any factor that may increase the risk of QTc prolongation or of arrhythmic events (e.g., hypokalaemia, congenital long QT syndrome, immediate family history of long QT syndrome or unexplained sudden death under 40 years of age).

7. Unresolved toxicity (except alopecia) from prior therapy of ≥ Grade 2 according to Common Terminology Criteria for Adverse Events (CTCAE) version 5.0.
8. Advanced visceral metastases if risk of life-threatening complications in the short term (including patients with massive uncontrolled effusions [pleural, pericardial, peritoneal], pulmonary lymphangitis and over 50% liver involvement).
9. Known symptomatic central nervous system (CNS) metastases, carcinomatous meningitis or leptomeningeal disease.
   - Patients with a history of CNS metastases or spinal cord compression due to metastasis are eligible if they have been treated with local therapy (e.g., radiotherapy, stereotactic surgery) and are clinically stable off anticonvulsants and steroids for at least 4 weeks before first dose of CT7001[redacted]
10. Refractory nausea and vomiting, chronic gastro-intestinal disease or previous significant bowel resection, with clinically significant sequelae that would preclude adequate absorption of CT7001.
11. Uncontrolled seizures.
12. Active infection requiring systemic antibiotic, antifungal, or antiviral medication within 14 days prior to allocation to study therapy.
13. Receipt of systemic corticosteroids (at a dose > 10 mg prednisone/day or equivalent) within 14 days before the first dose of IMP.
14. Has received a live-virus vaccination within 28 days or less of planned treatment start. Note: seasonal flu vaccines that do not contain live virus are permitted.
15. Severe or uncontrolled medical condition (e.g., severe chronic obstructive pulmonary disease, severe Parkinson’s disease, active inflammatory bowel disease or psychiatric condition).
16. Active bleeding diatheses.
17. History of haemolytic anaemia or marrow aplasia.

18. Renal or other organ transplant.
19. Known hepatitis B, hepatitis C, or human immunodeficiency virus infection.
20. Pregnancy.
21. Breastfeeding.
22. Non-biological anti-cancer medicines within 28 days or ≤ 5 half-lives, whichever is shorter, before the first study dose.
23. Biological anti-cancer medicines (e.g., monoclonal antibodies, antibody-drug-conjugates) within 42 days before the first study dose.
24. Receipt of St John's Wort within 21 days before the first study dose.
25. Concomitant medication, herbal supplement or food that is a strong inhibitor or inducer of CYP3A4, CYP2C19, CYP2D6, or P-glycoprotein activity within 14 days before the first dose of CT7001 (specific examples listed in protocol Appendix B).
26. Receipt of a blood transfusion (blood or blood products) within 14 days before the first study dose of IMP.
27. Known hypersensitivity to CT7001, fulvestrant or any excipient of the investigational products.
28. Diagnosis of any other malignancy within 3 years prior to enrolment, except for adequately treated basal cell or squamous cell skin cancer, or carcinoma in situ of the cervix.
29. In the opinion of the Investigator, unlikely to comply with study procedures.
30. Patients who are investigational site staff members directly involved in the conduct of the trial and their family members, site staff members otherwise supervised by the Investigator, or patients who are Carrick employees directly involved in the conduct of the trial.

Host Genetics Research Study - Pharmacogenomics Samples (Optional)

Patients who meet any of the following criteria will be excluded from optional genetic sub-studies:

1. Allogenic bone marrow transplant.
2. Non-leucocyte-depleted whole blood transfusion within 120 days of the date of the genetic sample collection.

Investigational Products: CT7001 (Samuraciclib), oral; [redacted] Fulvestrant; i.m. injection

<u>Primary Endpoint, study Part A</u>

- Dose-limiting toxicities and type, incidence, severity (as graded by CTCAE v5.0), seriousness and relationship to study medications of adverse events (AEs) and any laboratory abnormalities.

[redacted]

- [redacted]

[redacted]

- [redacted]

Secondary Endpoints

- Objective Response [redacted]
- Duration of Response [redacted]
- Clinical Benefit Response (complete or partial response, or stable disease ≥ 24 weeks; all study parts).
- Best percent change in tumour size.
- [redacted]
- Trough plasma concentrations of CT7001 (all study parts).
- Trough plasma concentrations of fulvestrant [redacted]
- Incidence and type of genotypes and copy number variation of CYP2D6, other CYP genes and drug transporter genes that may be involved in the metabolism of CT7001 (all study parts).

Exploratory Endpoints (all study parts)

- Metabolites of CT7001.
- Biomarkers in peripheral blood mononuclear cells (PBMCs), circulating tumour DNA and tumour tissue, including genes, RNA expression and proteins (e.g., c-Myc, MCL-1, phosphorylated CDK1 and Rb proteins, p53, CDK7, ER, ESR1, AR, PIK3CA).
- Effect of patient characteristics (including but not limited to race, ethnicity, age, CYP and drug transporter polymorphisms) on trough concentrations of CT7001 (all study parts).
- Correlations between CT7001 exposures and efficacy/safety findings in this patient population (all study parts).

Analysis Populations

Intent-to-Treat (ITT) Population

The ITT population will include all enrolled patients with designated study drug assignment.

As-Treated (AT) Population

The AT population will include all patients who receive at least 1 dose of study treatment, with treatment assignments designated according to actual study treatment received.

- In each study part, the AT population will be the primary population for evaluating safety and treatment administration/compliance.

Evaluable for Response Population

Evaluable for Response Population: All subjects who received at least 1 dose of CT7001 and had measurable disease at baseline.

Pharmacokinetics (PK) Population

The PK population will include all patients who received at least one dose of CT7001 and fulvestrant, had at least one plasma concentration of CT7001 or fulvestrant above the lower limit of quantification and had no protocol deviations or other events that may impact PK analysis. The PK population will serve as the PK analysis set in all parts of the study.

Efficacy Analysis

Analyses of PFS will be based on the ITT population. Analyses of ORR, CBR, tumour size will be performed on the evaluable for response population.

All analyses will be performed by using SAS® Version 9.1.3 or higher.

The primary analyses of efficacy endpoints will be based on local radiologist's/investigator's assessments. If required, supportive analyses may be performed based on BICR of radiographic images and clinical information through a third-party core imaging laboratory.

Subgroup analyses may be performed. Of special interest will be the subgroups defined by the stratification factors:

- Patient has only non-measurable disease at baseline in accordance with RECIST v1.1, yes versus no.
- Presence of liver metastases, yes versus no.

Analysis of Progression-Free Survival

[redacted] PFS is defined as the time from the date of randomization to the date of the first documentation of objective progression of disease (PD) or death due to any cause, whichever occurs first. PFS data will be censored on the date of the last tumour assessment on study for patients who do not have objective tumour progression and who do not die while on study. Patients lacking an evaluation of tumour response after randomization will have their PFS time censored on the date of randomization with a duration of 1 day. Patients who start a new anti-cancer therapy prior to

documented PD will be censored at the date of the last tumour assessment prior to the start of the new therapy. Further details related to the definition of censoring will be included in the SAP.

The primary analyses of PFS will be performed in the ITT population, based on local assessment of objective PD. A stratified log-rank test will be used to compare PFS time between the 2 treatment arms with a one-sided significance level of 0.1. The stratification factor(s) are specified in Section 5. If the 1-sided p-value is <0.1 this would indicate there is a less than 10% probability that the observed result could have occurred due to chance. This size of alpha error is consistent with the primary aim of Phase 2 studies which is to identify new agents or therapies that are sufficiently promising for advancing to subsequent Phase 3 development (Rubinstein et al., 2005).

The PFS time associated with each treatment arm will be summarized for the ITT population using the Kaplan-Meier method and displayed graphically where appropriate. Confidence intervals (CIs) for the 25th, 50th and 75th percentiles of the event-free time will be reported. The Cox Proportional hazards model will be fitted to compute the treatment hazard ratio and the corresponding 2-sided 95% as well as upper 1-sided 90% CI.

If required, additional supportive PFS analyses for both populations may be conducted based on BICR of objective PD.

[redacted]

[redacted]

[redacted]

[redacted]

[redacted]

Objective Response

Objective response (OR) is defined as a complete response (CR) or partial response (PR) according to RECIST version 1.1 (Appendix C).

A patient will be considered to have achieved an OR if the patient has a CR or PR according to RECIST v.1.1 definitions. Otherwise, the patient will be considered as non-responders in the OR rate analysis. Additionally, patients with inadequate data for tumour assessment (e.g., no baseline or post-baseline assessment) will be considered as non-responders in the OR rate analysis.

The ORR will be estimated by dividing the number of patients with objective response (CR or PR) by the number of patients with measurable disease allocated to study treatment. An exact binomial 95% CI will be computed.

[redacted]

Primary ORR analyses will be based on the investigator's assessment. If required, additional analyses may be conducted based on BICR. The best overall response for each patient will be summarized by treatment arm.

The evaluable for response population will serve as primary population for ORR analysis.

Duration of Response

Duration of response (DOR) is defined as the time from the first documentation of objective tumour response (CR or PR) to the first documentation of objective tumour progression or to death due to any cause, whichever occurs first. DOR data will be censored on the date of the last tumour assessment on study for patients who do not have objective tumour progression and who do not die due to any cause while on study. DOR will only be calculated for the subgroup of patients with an objective response.

[redacted]

Clinical Benefit Response

Clinical benefit response (CBR) is defined as CR, PR, or stable disease (SD) lasting ≥24 weeks recorded in the time period between enrolment and disease progression or death to any cause. The CBR rate will be estimated by dividing the number of patients with CR, PR, or SD ≥24 weeks by the number of patients in the particular analysis population. A 95% CI for the CBR rate will be provided.

[redacted]

The evaluable for response population will serve as primary population for CBR analysis.

[redacted]

Waterfall Plot Analysis

In Part A [redacted] the best percent change versus baseline in post-baseline aggregate tumour size measurements will be displayed graphically in form of Waterfall plots for the evaluable for response population. [redacted]

The evaluable for response population will serve as primary population for tumour size analysis.

Safety Analysis

The AT population will be the primary population for safety evaluation.

Adverse Events (AEs)

AEs will be classified using the Medical Dictionary for Regulatory Activities (MedDRA) classification system. Whenever possible, the severity of the toxicities will be graded according to CTCAE version 5.0, publication date: November 27, 2017; https://ctep.cancer.gov/protocolDevelopment/electronic_applications/docs/CTCAE_v5_Quick_Reference_8.5x11.pdf).

AEs will be summarized by the frequency of patients experiencing treatment emergent adverse events corresponding to body systems and MedDRA preferred term. AEs will be graded by worst NCI CTCAEv5.0 Grade. AEs will be summarized by cycle and by relatedness to study treatment. The frequencies of the worst severity grade observed will be displayed by study treatment. Detailed information collected for each AE will include a description of the event, duration, whether the AE was serious, intensity, relationship to study drug, action taken, and clinical outcome. Emphasis in the analysis will be placed on AEs classified as treatment emergent.

AEs leading to death or discontinuation of trial treatment, events classified as NCI CTCAE v5.0 Grade 3 or higher, trial drug-related events, and serious adverse events will be considered with special attention.

Laboratory Abnormalities

Haematology, serum chemistry and urinalysis data will be summarized by cycle. The laboratory results will be graded according to the NCI CTCAEv5.0 severity grade. The frequencies of the worst severity grade observed will be displayed by study treatment. For parameters for which an NCI CTCAEv5.0 scale does not exist, the frequency of patients with values below, within, and above the normal ranges will be summarized by treatment.

Electrocardiogram (ECG) Analysis

All ECGs obtained during the study will be evaluated for safety. The triplicate data will be averaged, and all summary statistics and data presentations will use the triplicate averaged data. Any data obtained from ECGs repeated for safety reasons after the nominal time-points will not be averaged along with the preceding triplicates. For all patients in the AT population, individual change in QTcF will be calculated for each nominal post-baseline time point. These individual changes will be summarized using descriptive statistics.

Pharmacokinetic Analysis

Summary statistics for the PK analysis set will be provided for trough concentrations of CT7001 and fulvestrant in Part A [redacted]. All patients treated with CT7001 and fulvestrant for whom drug plasma concentration results (from at least 1 visit) are available will be included in the analysis. Incidence and type of genotypes and copy number variation of CYP2D6, other CYP genes and drug transporters genes will be summarised (all study parts).

In addition, the relationship between exposure and safety and efficacy endpoints and/or the relationship between trough concentration and potential covariates (e.g., CYP2D6 polymorphisms) will be explored, based on emerging safety and efficacy data. The effect of patient characteristics (including but not limited to race, ethnicity, age, CYP and drug transporter gene polymorphisms) on trough concentrations of CT7001 (all study parts) may be investigated. These data may be combined with data from other CT7001 studies. The results of these modelling analyses may be reported separately from the clinical study report.

Biomarker Analysis

Appropriate statistical methods will be used to investigate any possible relationship of candidate biomarkers with the recorded efficacy outcomes.

Analysis of Other Endpoints

Descriptive statistics will be used to summarize patient characteristics, treatment administration/compliance and biomarkers. Data will be displayed graphically, where appropriate.

STUDY PROTOCOL OF CT7001_001, MODULE 2

A Phase 1/2 Study of CT7001 in Combination with Fulvestrant in Patients with Metastatic or Locally Advanced Hormone-Receptor-Positive and Human Epidermal Growth Factor Receptor 2-Negative Breast Cancer

1 INTRODUCTION

CT7001 (Samuraciclib) is a small molecule, adenosine triphosphate (ATP) competitive, selective oral inhibitor of cyclin-dependent-kinase 7 (CDK7). A first-in-human modular Phase 1/2 clinical study was initiated in November 2017 (EudraCT number: 2017-002026-20; ClinicalTrials.gov identifier: NCT03363893). The single and multiple-ascending dose part of the Phase 1 study (Module 1A) has now completed and has determined 360 mg given OD as the preliminary recommended Phase 2 dose (RP2D) for further testing as monotherapy in Module 1B. The Module 1A paired biopsy expansion cohorts in breast cancer is completed. The planned additional cohort in solid tumour planned will not initiate. A study of the effect of food on the bioavailability of CT7001 (called Module 4) has completed recruitment and PK data from the randomised, controlled fed-fast period is available. Module 1B-1 (TNBC), an expansion cohort in women with metastatic or recurrent triple-negative breast cancer (TNBC), has received approval by the US Food and Drug Administration (FDA) and UK Medicines and Healthcare Products Regulatory Agency (MHRA) and has completed. Module 1B-2 (CRPC) has completed. Module 6 is a study to investigate an enteric capsule formulation of CT7001 on PK and safety profile and is planned to open in 2021. The present study (Module 2) is a first effort to evaluate CT7001 in combination with fulvestrant in patients with locally advanced or metastatic hormone receptor-positive (HR-positive) and human epidermal growth factor receptor 2-negative (HER2-negative) breast cancer (BC).

2 BACKGROUND AND RATIONALE

The potential role of CDK7 in cancer in general and in HR-positive BC in particular, the nonclinical efficacy observed of CT7001 in combination with endocrine therapies in models of HR-positive BC, the clinical safety, PK and PDc data of CT7001 in Module 1A and the continuing need of enhancing/ restoring clinical sensitivity to established endocrine treatments through rationale combination with agents which have a novel mechanism of action provide a sound rationale for the present study.

2.1 Hormone Receptor-Positive Breast Cancer

HR-positive BC accounts for approximately 80% of all BC and represents the largest BC subgroup (Russnes et al., 2017). Approximately 10% of HR+ BC are also positive for HER2.

For decades, endocrine therapy has been the mainstay of systemic treatment of HR+ BC. This included surgical or medical castration, the anti-oestrogen tamoxifen, the selective oestrogen receptor degrader (SERD) fulvestrant and AIs such as letrozole, anastrozole and exemestane. Endocrine therapies have significantly improved disease-free and overall survival when used as adjuvant therapy in early breast cancer (Burstein et al., 2016) and progression-free and overall survival when used for treatment of metastatic disease (Giordano et al., 2018). The mTOR inhibitor everolimus has achieved significant improvement in PFS when combined with exemestane in postmenopausal patients who had previously failed a non-steroidal AI (Baselga et al., 2012; Piccart et al., 2014), which led to its approval by US FDA and EMA in this indication. Of note, despite the promising efficacy demonstrated in this study, a high percentage of patients discontinued everolimus due to a challenging tolerability profile. A recent randomized Phase 2 study reported significant PFS improvement when everolimus was added to fulvestrant (Kornblum et al., 2018). Recently, CDK4/6 inhibitors such as palbociclib, ribociclib and abemaciclib, have received approval by US FDA and EMA for the treatment of metastatic HR-positive BC as they have produced substantial extension of PFS when combined with AIs (Finn et al., 2016; Hortobagyi et al., 2016; Goetz et al., 2017; Tripathy et al., 2018; Turner et al., 2015) or fulvestrant (Turner et al., 2018; Slamon et al., 2018; Sledge et al., 2017; Cristofanilli et al., 2016). When used as monotherapy in patients who had failed endocrine therapies and chemotherapy, the CDK4/6 inhibitor abemaciclib has achieved an objective response rate of approximately 20% with good durability (Dickler et al., 2017), which led to approval by the US FDA for that indication. Approximately 40% of patients with HR-positive breast cancer harbour PIK3CA mutations (Loi et al., 2010). Two recently reported Phase 3 studies of PI3K inhibitors in combination with fulvestrant showed some improvement in PFS but significant toxicity (Baselga et al., 2018; Andre et al., 2018). The use of cytotoxic chemotherapy in HR-positive metastatic BC is limited to patients present with aggressive clinical disease or have exhausted endocrine treatment options. In patients with HR- and HER2-positive BC, the addition of a HER2 inhibitor to endocrine therapy has improved outcome significantly over endocrine therapy alone (Johnston et al., 2009; Kaufman et al., 2009). Combination of chemotherapy or endocrine therapy with a HER2 inhibitor has become the standard of care in this subgroup (Giordano et al., 2018) which thus will not be part of the present study.

Fulvestrant is a standard treatment option in patients with progressive disease after previous endocrine treatment including an AI (Rugo et al., 2016; Cardoso et al., 2018). The combination of fulvestrant and various CDK4/6 inhibitors has shown significant efficacy improvement in such patients when compared to fulvestrant plus placebo (Turner et al., 2018; Slamon et al., 2018; Sledge et al., 2017; Cristofanilli et al., 2016). The combination of an AI with a CDK4/6 inhibitor has become a standard treatment option for patients with HR-positive metastatic BC in routine clinical practice (Rugo et al., 2016; Cardoso et al., 2018). There is very little data of fulvestrant monotherapy reported to date in a patient population which had previously received a CDK4/6 inhibitor. A subgroup analysis from the phase 3 study SOLAR-1 study, that has led to the approval of the PI3K inhibitor alpelisib for patients with HR+ breast cancer, in which the median PFS of fulvestrant monotherapy was 1.8 months for patients who had received prior therapy with a CDK4/6 inhibitor (Juric et al 2019, Andre et al 2020). The present study is an effort to evaluate the safety and efficacy of fulvestrant in combination with the CDK7 inhibitor CT7001 in this patient population.

It has been reported that patients with breast cancer who have liver metastases experience significantly worse PFS than those without liver involvement when treated with fulvestrant (He et al 2019). A review of the emerging data from Module 2A in September 2020 found that 10/15 (67%) patients recruited had liver metastases, whereas the literature would suggest that the actual prevalence is around 30% (Andre et al 2020). In view of this finding, on 11th September 2020 the DMC approved the request to not enrol further patients with liver metastasis into Part A to rebalance this cohort of patients.

2.2 CDK7

CDK7 has three critical roles in cancer. These are enhanced transcriptional initiation of multiple oncogenes such as c-Myc and upregulation of anti-apoptotic genes such as MCL-1 via phosphorylation of the c-terminal domain of RNA Polymerase II (Chipumuro et al., 2014; Feaver et al., 1994; Fisher., 2005; Glover-Cutter et al., 2009; Kwiatkowski et al,, 2014), acceleration of progression through the cell cycle via phosphorylation of other members of the CDK family (such as CDK2, 4 and 6) (Fisher., 2005; Fisher and Morgan, 1994; Schachter and Fisher, 2013; Schachter et al., 2013), and loss of sensitivity to hormonal therapy via phosphorylation of ERα (Chen et al., 2000) and the transcriptional coactivator MED1 (Rasool et al, 2019). The latter two mechanisms seem of particular relevance in HR-positive BC. Of note, inhibition of CDK7 was recently reported to overcome resistance to CDK4/6 inhibitors in HR-positive BC cells, CDK7 was identified as a top ranked essential gene in these cells and CDK7 inhibition showed synergistic activity when combined with fulvestrant (Guarducci et al., 2018). Please refer to the Investigators Brochure (IB) for a more comprehensive review.

2.3 CT7001

CT7001 (previously also known as ICEC0942) is a small molecule, ATP competitive, selective oral inhibitor of CDK7 which potently inhibits all key biological effects of CDK7 in cancer (Patel et al., 2018).

2.3.1 Overview of Nonclinical Data

The IB provides a comprehensive review and description of the relevant nonclinical study data of CT7001.

Pharmacokinetic studies showed good oral bioavailability in three non-human species (mouse, rat and dog), which predicted good bioavailability in humans. Plasma clearance was high in rats and dogs with a high volume of distribution, resulting in an apparent elimination half-life ($T_{½}$) of 4.8 hours in rats and 9.5 hours in dogs.

Cell growth inhibition studies showed broad activity of CT7001 against a wide range of tumour cell lines, including the HR-positive, oestrogen-sensitive MCF-7 cell line (Patel et al., 2018; Ainscow et al., 2018; Clark et al., 2017; Ali et al., 2018). CT7001 also exhibited encouraging *in vivo* activity in MCF-7 xenograft studies as a single agent but most notably a strong combinatorial effect with endocrine therapy (Patel et al., 2018; Ali et al., 2018). CT7001 inhibited CDK7-mediated phosphorylation of RNA PolII (pPolII) and retinoblastoma (RB) phosphorylation in MCF7 cells in a time- and dose-dependent manner, the RB effect produced via inhibition of CDK2/4/6 activity. CT7001 inhibited phosphorylation of serine 118, the ER

site targeted by CDK7, in culture and in tumour xenografts of MCF-7 cells, indicating that CT7001 inhibits ER activity (Patel et al., 2018; Ali et al., 2018). Of note, recent studies in cell lines with acquired resistance to CDK4/6 inhibitors have shown similar sensitivity to CT7001 in the parental and resistant lines (unpublished data).

Western blot analysis showed that CT7001 inhibits the phosphorylation of RNA Pol II, CDK1 and 2, *in vitro* and *in vivo* and this in tumour and in normal cells. This suggested that pPolII or phosphorylation status of other CDK family members could be useful pharmacodynamics (PDc) biomarker in early clinical development.

CT7001 was metabolically stable in human microsomes and hepatocytes *in vitro*. Intrinsic clearance of CT7001 was high in rat microsomes, intermediate in mouse and dog microsomes, and low in human liver microsomes. Metabolite profiling following incubation of CT7001 with mouse and human hepatocytes showed that the overall turnover of CT7001 was low, indicating the compound has high stability. No evidence of Phase II metabolism (e.g., glucuronidation) was observed.

In a cytochrome P450 (CYP) study using human microsomes, CT7001 strongly inhibited CYP3A4 and showed weak signals for 2D6 and 2C19, with mean half-maximal inhibitory concentration (IC_{50}) values of 5.7, 35.5, and 44.9 µM, respectively. Mean IC_{50} values for CYP1A2, 2C9, 2B6, 2C8 and 3A5 were greater than 50 µM. In a separate assay, the mean IC_{50} of CT7001 for CYP3A4 was 2.9 and 1.8 µM when using testosterone or midazolam as substrates, respectively.

Based on the *in vitro* data, CT7001 is unlikely to cause clinically significant hepatic drug interactions through inhibition of CYP1A2, 2C9, 2B6, 2C8 and 3A5. However, there is a potential for inhibition of intestinal and hepatic CYP3A4 which may be clinically significant for CYP3A4 substrates. CT7001 has shown weak potential to inhibit CYP2D6 and 2C19 which is unlikely to be clinically significant but should be considered when co-medicating with 2D6 and 2C19 substrates.

A phenotyping study investigating the metabolism of CT7001 by eight cytochrome P450 enzymes found that CYP mediated clearance of CT7001 mainly occurs via CYP2D6, followed by 3A4 and a small contribution by 2C19. This suggests that co-medications that modulate the activity of CYP 2D6, 3A4 and 2C19 may affect the exposure of CT7001. However, the proportion of metabolized CT7001 was small.

The potential of CT7001 to inhibit transporter proteins has not been studied yet. Plasma protein binding is high (>~95%), with a similar unbound fraction in all 3-species tested (rat, dog and human).

Daily administration of 100 mg/kg/day CT7001 to Han Wistar rats for 7 days was well tolerated. Microscopic findings seen in the gastrointestinal tract and testes suggest an effect of CT7001 on rapidly dividing cells, likely a result of the pharmacological action of the test article.

The daily administration of 15 or 60 mg/kg/day CT7001 to rats and 20 mg/kg/day CT7001 to beagle dogs for 7 days was also well tolerated with no unscheduled deaths or macroscopic or microscopic changes. However, food consumption was reduced over the dosing period; subsequent moistening of the food showed increased consumption. A significant decrease in

reticulocyte numbers was observed. Erythroid enucleation is believed to be dependent on the activity of transcriptionally active CDKs, including CDK7 (Wölwer et al., 2015). Therefore, the reduction in reticulocytes was interpreted as a pharmacological effect of CT7001 and considered as another PDc biomarker of potential utility in early clinical development of CT7001.

The main effects of toxicological significance produced by CT7001 in both rats and dogs are its manifestation in tissues with rapidly dividing cells, namely bone marrow, lymphoid tissue and the gastrointestinal tract. All effects reversed partially or fully within 4 weeks from cessation of dosing and are consistent with the pharmacological mode-of-action of the drug. CT7001 is not phototoxic.

CT7001 showed no inhibition of human ether-a-go-go-related gene (hERG) channel tail current in a test using the whole-cell patch-clamp technique (human embryonic kidney [HEK] cells transfected with hERG), and the CT7001 IC_{50} in the K^+ channel was determined to be greater than 5 μM.

Cardiovascular toxicity potential was also studied *in vivo* in dogs. Oral administration of 5, 15, and 20 mg/kg CT7001 had no effect on haemodynamic parameters, ECG parameters, or body temperature in the dog, compared with control article (vehicle) administration.

Effects of CT7001 on the central nervous system (CNS [Irwin]) and respiratory systems were assessed in rats. No significant effects were observed in the test article groups.

CT7001 has low mutagenic potential. At concentrations up to the lower limit of toxicity, it induced no mutations in a five strain Ames Test. Low potential for genotoxicity was demonstrated in micronucleus tests with human peripheral blood lymphocytes.

2.3.2 Current Clinical Data of CT7001

A first-in-human multi-module Phase I/II clinical study commenced in November 2017 (EudraCT number: 2017-002026-20; ClinicalTrials.gov identifier: NCT03363893). This is the only clinical study conducted to date.

The single- and multiple-ascending dose Phase 1a part of the study (Module 1, Part A) is completed. 33 dosed patients have completed this part of Module 1 (120, 240, 480 mg and a step-down dose level of 360 mg, all taken once daily (OD), and 180 mg given twice a day (BID), all in a fasted state). Module 1A also included separate, additional cohorts with paired biopsies pre-dose and on study, these cohorts have now completed with 11 patients with BC dosed.

Module 4 evaluated the effect of food on CT7001 bioavailability in cancer patients. Two single doses of 120 mg (Cohort 1) or 360 mg (Cohort 2) were given to assess the effect of food on the bioavailability of CT7001. Patients then continued on daily dosing, which originally was 240 mg and changed to 360 mg after determination as recommended Phase 2 dose in Module 1A. Recruitment to Module 4 is complete with 15 patients dosed and 12 patients evaluable for testing of food effect. The results are summarised in Section 2.3.2.2.

Module 1B is a Phase 1b expansion to refine the safety, tolerability, and PK and PDc profiles of CT7001 in patients with advanced solid malignancies. 4 cohorts, each up to 25 patients, may

be opened. Cohort 1B-2 (CRPC) has completed with 11 patients dosed. Other cohorts potentially include SCLC, Ovarian cancer and other appropriate cancer indications.

Module 1B-1 (TNBC) is a larger expansion cohort of CT7001 at 360 mg OD in up to 50 patients with triple negative breast cancer (TNBC). Module 1B-1 (TNBC) completed with 23 patients dosed. This is an open-label, uncontrolled Phase Ib study to determine the recommended Phase 2 dose of CT7001 as monotherapy, further characterize safety, tolerability and blood concentrations of CT7001, and explore its anti-tumour activity in triple negative breast cancer.

Module 2 is a 3-part Phase 2 study designed to evaluate the safety, tolerability and efficacy of CT7001 in combination with fulvestrant in patients with HR+ve / HER2-ve breast cancer. Part A is a single arm open label study to establish the recommended CT7001 dose (240 mg or 360 mg OD) to take forward into Part B and to further refine tolerability. Recruitment to Part A is complete with 6 patients dosed at 240 mg OD and 25 patients dosed at 360 mg OD. [redacted]

Module 6 is planned to open in 2021. This is a Phase 1 study designed to evaluate the PK and tolerability of a new enteric capsule forumulation of CT7001 at 360 mg OD in 36 patients with advanced solid tumours.

Table 1 provides a summary of the current enrolment and dosing status in Modules 1A, 1B, 2 and 4.

Table 1: Status of Modules 1A, 1B, 2 and 4 as of 07-May-2021

Module	Cohorts	Dose	Recruited	Dosed	Ongoing
1A	Cohort 1	120 mg OD	8	6	0
1A	Cohort 2	240 mg OD	7	7*	0
1A	Cohort 3	480 mg OD	8	6	0
1A	Cohort 4	360 mg OD	6	6	0
1A	Cohort 5	180 mg BID	9	8	0
1A Paired Biopsy	Breast Cancer	240 mg OD	9	5	0
1A Paired Biopsy	Breast Cancer	360 mg OD	9	6	0
4	Cohort 1	120→ 240 mg OD	9	8	0
4	Cohort 2	360→ 360 mg OD	9	7	1
1B-1 (TNBC)	N/A	360 mg OD	36	23	0
1B-2 (CRPC)	N/A	360 mg OD	15	11	0
2	Part A	240 mg OD	12	6	0
2	Part A	360 mg OD	37	25	9
	**TOTAL**		**174**	**124**	**10**

* Includes 1 replacement subject from Cohort 3 who had a starting dose of 240 mg

The subsections below provide a synopsis of the clinical data as recorded in the database as of 12-Apr-2021. Please refer to the IB for detailed information. The data reported here and in the IB is to be viewed as preliminary as study CT7001_001 is ongoing, information continues to be rapidly evolving and the database has not been locked and thus most data has not been cleaned yet.

2.3.2.1 Safety

As of 12 April 2021, data is available from 93 patients dosed with CT7001 as monotherapy (M1A, M1B and M4) and 31 patients dosed with CT7001 in combination with Fulvestrant.

In general, CT7001 has shown good safety and acceptable tolerability. Most frequently recorded were diarrhoea, nausea and vomiting, which occurred in more than 75% of patients across dose levels, largely at Grade 1. There was no apparent relationship with dose or with blood concentrations of CT7001 (C_{max}, AUC or trough levels).

Diarrhoea is an expected target-related adverse effect. Nausea and/or vomiting started usually a few hours after administration of CT7001. It cannot be excluded that this is related to C_{max}. This prompted to explore BID dosing in Cohort 5 which, however, did not appear to reduce the incidence of vomiting or of nausea. A current working hypothesis is that a main cause of vomiting as well as nausea may be local chemical irritation of the gastric mucosa and taking the drug after a meal may ameliorate these effects. Therefore, Module 4 (which has evaluated

the impact of food on the bioavailability of CT7001) was brought forward in the development program. Food had no clinically significant effect on overall CT7001 exposure at RP2D. Fed dosing is now permitted across the program. The therapeutic or preventive use of common anti-emetics had a positive effect in some patients but little effect in others.

Laboratory abnormalities were uncommon.

- There have been 17 events of increased ALT and 17 events of increased AST reported; the majority of these have been Grade 1
- There have been 18 events of anaemia reported; the majority of these have been Grade 1 or 2
- At 240mg OD and 360mg OD there appears to be a ~20% drop in platelet count in all patients. This appears over the first 15 days on study and then is stable for the duration on treatment; in the majority of patients this is within the normal range of platelet counts. All changes in platelet counts appear fully reversible upon discontinuation of CT7001. There have been 16 platelet related AEs reported:
    - 2 events of Grade 4 thrombocytopenia (1 at 180mg BID and 1 at 360mg OD); the event at 180mg BID was associated with minor nose bleeding
    - 1 event of Grade 3 thrombocytopenia (at 360mg OD)
    - 2 events of Grade 2 thrombocytopenia (at 360mg OD)
    - 1 event of Grade 2 platelet count decreased (at 360mg OD)
    - 6 events of Grade 1 thrombocytopenia (at 360mg OD)
    - 4 events of Grade 1 platelet count decreased (1 at 240mg OD and 3 at 360mg OD)
- Of note, only 3 events of Grade 1 neutropenia/white blood cell count decreased (2 at 360mg OD and 1 at 180mg BID) have been reported.

Ten serious adverse events (excluding those deemed not related and unlikely related to treatment with CT7001 by the investigator) were reported in 124 subjects. The related events concerned:

- Thrombocytopenia: 2 events of Grade 4 were reported in patient M1A01C501, dosed at 180mg BID and in patient M1B04E1C106, dosed at 360mg OD
- Oesophagitis and gastro-oesophageal reflux disease: 2 Grade 3 events in patient M1A01R503, dosed at 180mg BID
- Diarrhoea: Grade 2 in patient M1A02R504, dosed at 180mg BID
- Anaemia (Grade 3), diarrhoea (Grade 3) and dyspnoea (Grade 2) in patient M1B04E1C106 dosed at 360mg OD
- Nausea: Grade 3, in patient M40104, dosed at 360mg OD
- Diarrhoea: Grade 2 in patient M2A04C101 dosed at 240mg

These cases were reported to regulatory authorities as serious unexpected adverse drug reactions (SUSAR). No death occurred which investigators attributed to study therapy.

Dose-limiting toxicities were only recorded at the non-tolerated dose of 480 mg OD (5 events) and on 180 mg given BID (5 events). These included 8 events which were defined as dose-limiting toxicity (DLT) in the study protocol. These were 1 case each of CTCAE Grade 3 diarrhoea, oral mucositis and vomiting at the non-tolerated dose of 480 mg OD, and 1 case each of Grade 4 thrombocytopenia, Grade 3 weight loss, Grade 3 anorexia, Grade 3 dysphagia/oesophagitis and Grade 3 heartburn at 180 mg BID. The Safety Review

Committee judged two additional events to represent a DLT on clinical grounds. One event was Grade 2 nausea and the other Grade 2 vomiting, each recorded at 480 mg OD in the same patient.

All adverse effects which investigators attributed to CT7001 were reversible upon interruption or discontinuation of study therapy.

2.3.2.2 Pharmacokinetics (PK)

Module 1A

Final PK data based on cleaned data and actual sampling time are available from Study CT7001_001 for Cohorts 1 to 5 (120, 240, 360 and 480 mg OD doses and 180 mg BID dose, all fasted). The single- and multiple-dose pharmacokinetics of CT7001 were evaluated using a sample-rich, non-compartmental analysis approach. Initially the single-dose PK samples were taken up until 48 hours (Cohorts 1, 2, and 3). This was found to be insufficient for reliable identification of the terminal elimination phase and derivation of the associated parameters: half-life, lambda_z, $AUC0_{-\infty}$, CL/F, Vz/F, MRT. Thus, further PK sample times points at 72, 120 and 168 hours were introduced for later cohorts to facilitate better characterisation of the single-dose pharmacokinetics. The majority of subjects reported either vomiting or diarrhoea adverse events on PK days that could conceivably have affected exposure, however as this occurred in so many patients none were excluded from the PK population. After oral administration CT7001 was rapidly absorbed with median T_{max} ranging from 1.5 to 4 hours across the cohorts. The absorption phase for CT7001 is characterised by double peaking in some subjects, plasma concentrations then underwent bi-phasic decline. Four of the six subjects dosed 360 mg and all the subjects (8) dosed 180 mg BID experienced the longer sampling regimen to 168 hours. For these subjects the determined half-life ranged (geometric mean) from 53.80 - 101.4 (76.41) hours and 48.68 - 87.45 (74.37) hours for the 360 mg and 180 mg doses respectively. The geometric mean accumulation ratio at steady-state ranged from 2.060 - 3.080 across the dose cohorts. Plasma exposure appeared to increase dose-proportionally after single and multiple dosing. Final multiple dose PK data based on cleaned data and actual sampling time are available from Study CT7001_001 for the Part A Paired Biopsy Breast Cancer Expansion Cohort and are consistent to those observed for cohorts 1 to 5.

Module 4

Preliminary PK data based on uncleaned data and nominal sampling time are available from Study CT7001_001 Module 4 for 120 and 360 mg single dose in the fed (high fat, high calorie meal) and fasted state. PK in the fasted state was similar to that observed in Module 1 with rapid absorption observed with median T_{max} of 1.5 and 4 hours respectively for the 120 mg and 360 mg cohorts and geometric mean half-life of 54.64 and 65.45 hours. After dosing in the fed state at 120 mg dose plasma concentrations appeared to be reduced and PK could not be accurately determined. As there was no safety concern of increased exposure after dosing in the fed state the dose was increased to the RP2D (360 mg) and the impact of dosing in the fed state assessed. After dosing in the fed state T_{max} was delayed by 3 hours with a median T_{max} of 7 hours. C_{max} appeared to be reduced with a ratio of geometric means fed : fasted of 0.84 (90% CI = 0.53 - 1.32) while overall exposure appeared to be unaffected when dosing in the fed state with a ratio of AUC_{0-72} geometric means fed : fasted of 0.96 (90% CI = 0.61- 1.50). Geometric mean half-life in the fed state was 61.24 hours.

Module 2

PK data for the CT7001-Fulvestrant combination is in the early stages of evaluation. Initial interpretation, based on unclean data on nominal times, is that there is no drug-drug interaction, however this remains to be confirmed in a larger data set.

2.3.2.3 Pharmacodynamics (PDc)

PDc data is currently available from surrogate normal cells. Data from paired tumour biopsies is pending. In normal cells, two PDc effects of CT7001 were evaluated, a biochemical and a biomechanical readout of CDK7 inhibition.

Based on the rationale and non-clinical data described in Sections 2.2 and 2.3.1, phosphorylated RNA polymerase II (pPolII) in PBMCs was used as biochemical PDc biomarker. Figure 1 illustrates the significant reduction of pPolII signal by ~30% induced by CT7001 in PBMC across all dose levels tested in Module 1A for subjects who have completed 21 days of treatment (i.e., cycle 2 day 1 timepoint). Later timepoints also show inhibition, although not reaching statistical significance due to the low number of subjects. This should be viewed in context of a maximal 55% reduction in signal observed in PBMCs incubated with CT7001 *ex vivo*.

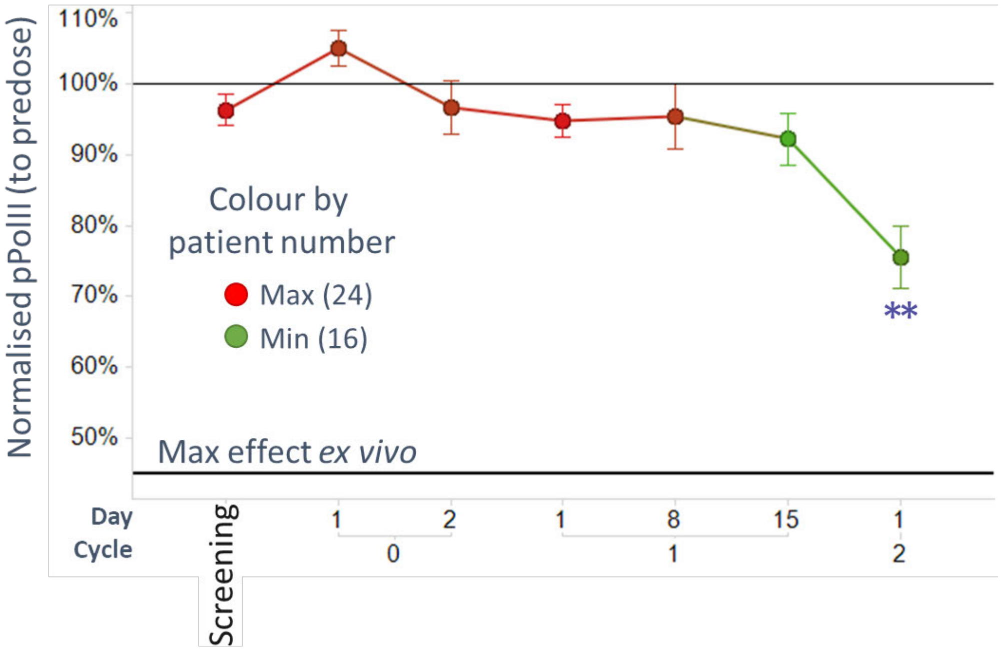

Figure 1: CT7001 decreases pPolII levels in PBMC.

pPolII was determined by a flow cytometry assay on subject samples of PBMC preparations derived from blood. The data show box plots for each sampling timepoint normalised to the screening sample for that subject. Note that samples were taken pre-dose at all timepoints. Statistical comparison between pre-treatment samples (Cycle 1 day 1) and subsequent timepoints were done using a 1-tailed Mann-Whitney test . ** data are significantly different to predose levels with $p < 1\%$.

2.3.2.4 Minimal Biologically Active Dose (MBAD)

The study's SRC originally declared 240 mg OD as MBAD based on observed target toxicity (diarrhoea), target-mediated PDc effect on reticulocyte counts and preliminary signals of anti-tumour activity. This prompted the opening of a cohort with paired breast cancer biopsies at 240 mg OD. Later review of the data from cohort 1 at the starting dose of 120 mg OD revealed similar biological activity as observed for 240 mg OD, including PDc and anti-tumour effects. Accordingly, 120 mg OD represents the lowest dose tested to date in humans that has demonstrated biological activity.

2.3.2.5 Non-Tolerated Dose

At 480 mg, 3/6 subjects experienced a protocol-defined DLT (1 case each of Grade 3 diarrhoea, mucositis or vomiting). Furthermore, 3 additional patients experienced G1-2 vomiting with little effect of anti-emetic therapy. In one of these patients, the SRC judged this to amount to a DLT, on clinical grounds. As a result, 480 mg was defined as non-tolerated dose when given OD in a fasted state.

2.3.2.6 Maximum Tolerated Dose (MTD) and Preliminary Recommended Phase 2 Dose (RP2D)

Six subjects in Cohort 4 (360 mg OD in fasted state) have completed Cycle 1 without recording a DLT or other concerning safety or tolerability findings. Accordingly, a dose of 360 mg OD given in a fasted state has been determined as the MTD and the preliminary RP2D for further characterization and investigation of CT7001 as monotherapy. Accordingly, this is the dose which has been taken forward to Module 4 Cohort 2, Module 1B-1, (TNBC), Module 1B-2 (CRPC), Module 6 and this is the confirmed starting dose for use in combination with fulvestrant in Module 2B.

2.3.2.7 Anti-Tumour Effects

Preliminary signals of anti-tumour effect have been observed. As of 12-Apr-2021, 4 RECIST partial response have been observed, 2 in patients treated with monotherapy CT7001 and 2 in patients treated with CT7001 in combination with fulvestrant:

- Subject M1A03E102, a 59-year old female with HR+ breast cancer, with visceral disease; metastases in liver and spleen. This patient had previously been treated with 5 lines of hormonal therapy (exemestane, letrozole, tamoxifen, anastrozole, fulvestrant) and 4 lines of chemotherapy (paclitaxel, eribulin, capecitabine, 5-FU/epirubicin/cyclophosphamide)
- Subject M1B04E1C107, a 49-year old female with TNBC, with metastases in axilla and lateral node. This patient had previously been treated with 2 lines of chemotherapy (neo adjuvant paclitaxel and carboplatin and palliative gemcitabine and carboplatin)
- Subject M2A02C102, a 53-year old female with HR+ breast cancer, with visceral disease; metastases in liver and lung. This patient had previously been treated with 2 lines of hormonal therapy (tamoxifen and letrozole) and a CDK4/6 inhibitor (palbociclib)
- Subject M2A33C201, a 58-year old female with HR+ breast cancer, with metastases in pleural cavity and lymph nodes. This patient had previously treated with 2 lines of hormonal therapy (tamoxifen and arimidex) and chemotherapy (adriamycin, cytoxan, and taxol) and a CDK4/6 inhibitor (palbociclib)

PSA reductions were also observed in all 4 CRPC patients recruited into M1A and M4. This triggered the M1B expansion in patients with CRPC to explore this finding further in patients with RECIST measurable disease.

Across all dose levels explored in Module 1A and 4, the number of patients with disease control (as defined by RECIST SD+PR+CR and/or PSA reduction with no RECIST PD) in those patients evaluable for assessment (as defined by having baseline and at least the first on treatment tumour assessment completed) was as follows:

- All dose levels: 16/46 (35%) at ≥ 8 wks and 8/46 (17%) at ≥ 16 wks
- Clinically relevant doses (240 mg OD and 360 mg OD): 14/31 (45%) at ≥ 8 wks and 8/31 (26%) at ≥ 16 wks

Module 1, Part B: TNBC patient expansion
In this cohort of 23 patients with advanced TNBC, who had previous received ≤ 3 lines of chemotherapy for their advanced disease, 20 were evaluable for tumour response. 1/20 patients had a PR and 12/20 patients had stable disease as their best response; 5 patients were treated for ≥ 24 weeks and 3 patients for ≥ 1 year.

Module 1, Part B: CRPC patient expansion
In this cohort of 11 CRPC patients, with disease measurable by RECIST, 5/10 patients had stable disease as their best response (1 patient was not evaluable for tumour response); no CRs or PRs were observed. 3 patients were treated for ≥ 24 weeks.

Module 2, Part A: HR+ Breast cancer, in combination with fulvestrant
In this cohort of 31 patients with advanced HR+, HER2- breast cancer, who had previously received ≤ 2 lines of endocrine treatment for their advanced disease, 22 were evaluable for tumour response. 2/22 patients had a PR and 14/22 patients had stable disease as their best response. 12 patients were treated for ≥ 16 weeks.

2.3.2.8 Summary of Current Clinical Experience

The current clinical experience with CT7001 has shown good safety and PK behaviour, positive PDc effects in surrogate normal cells and preliminary signs of anti-tumour effect, warranting further clinical investigation.

2.4 Fulvestrant

Fulvestrant is a potent selective oestrogen receptor degrader (SERD) which is commonly used in routine clinical care of patients with HR-positive BC. Fulvestrant has currently US FDA and EMA approval for the treatment of:

- HR-positive, HER2-negative advanced BC in postmenopausal women not previously treated with endocrine therapy.
- HR-positive advanced BC in postmenopausal women with disease progression following endocrine therapy.

- Treatment of HR-positive, HER2-negative advanced or metastatic BC in combination with palbociclib or abemaciclib in women with disease progression after endocrine therapy.

Initial approval of fulvestrant was limited to postmenopausal women. However, the recent Phase 3 combination studies with the CDK4/6 inhibitors palbociclib and abemaciclib included pre/perimenopausal women who received an LHRH agonist. This has extended approval to that population.

Two initial Phase 3 trials versus anastrozole used fulvestrant at 250 mg showed no difference in time to death (HR (hazard ratio) 1.01, 95% CI 0.86-1.19) between the two treatment groups (Osborne et al., 2002; Howell et al., 2002). A subsequent Phase 3 trial (CONFIRM) in postmenopausal women with advanced breast cancer who had disease progression on or after anti-oestrogen therapy (423 patients) or AI therapy (313 patients) compared fulvestrant at 500 mg (n=362) versus 250 mg (n=374) (Di Leo et al., 2010). Median PFS for fulvestrant 500 mg was 6.5 months compared to 5.5 months for fulvestrant 250 mg, with a corresponding median overall survival of 26.4 months versus 22.3 months (HR 0.81, 95% CI 0.69-0.96, p-value 0.016). A neoadjuvant study compared the two dose levels of fulvestrant and found significantly greater reduction in Ki67 and ER for 500 mg (Kuter et al., 2008). A randomized, double-blind Phase 3 trial (FALCON) compared fulvestrant 500 mg to anastrozole 1 mg in patients with HR-positive BC who had not received prior endocrine therapy for metastatic disease (Robertson et al., 2016). PFS was significantly longer in the fulvestrant group than in the anastrozole group (HR 0.797, 95% CI 0.637–0.999, p=0·0486). Median PFS was 16.6 months (95% CI 13.83–20.99) in the fulvestrant group versus 13.8 months (11.99–16.59) in the anastrozole group. These data have led to regulatory approval of fulvestrant at 500 mg and subsequent use of the 500 mg dose in routine clinical care and in clinical trials.

Fulvestrant at 500 mg was used as endocrine treatment standard in various recently reported randomized controlled trials of novel agents. This included the PALOMA-3 (Cristofanelli et al., 2016), MONALEESA-3 (Slamon et al., 2018) and MONARCH-2 (Sledge et al., 2017) Phase 3 trials of fulvestrant with various CDK4/6 inhibitors, the BELLE-2 (Baselga et al., 2017), FERGI (Krop et al., 2016) and SOLAR-1 (Andre et al., 2018) Phase 3 studies of fulvestrant with various PI3K inhibitors, and a randomized Phase 2 study in combination with the mTOR inhibitor everolimus (Kornblum et al., 2018).

Among these studies, BELLE-2, FERGI, SOLAR-1 and PrE0102 required prior use of an AI and thus the PFS data from these four studies were used for sample size estimation in Part B of the current trial (see Section 10.2.1.1).

Please refer to the most recent versions of the US FDA label and EMA SPC and European Public Assessment Report (EPAR) in Appendix E for detailed information on fulvestrant.

Fulvestrant at 500 mg can be considered a standard treatment option in patients with HR-positive, HER2-negative metastatic breast cancer whose disease has progressed after prior endocrine therapy, including AIs. The present study is an effort to evaluate the safety and efficacy of fulvestrant in combination with the CDK7 inhibitor CT7001 in patients with HR-positive/HER2-negative advanced breast cancer whose disease has progressed after prior therapy with an AI and a CDK4/6 inhibitor.

2.5 Potential for Drug-Drug Interaction (DDI) Between CT7001 and Fulvestrant

The potential for clinically significant DDI between CT7001 and fulvestrant appears to be very low.

As described in Section 2.3.1, CT7001 has shown no evidence of Phase II drug metabolism (e.g., glucuronidation). The CYP mediated clearance of CT7001 mainly occurs via CYP2D6, followed by 3A4 and 2C19. CT7001 is a strong inhibitor of CYP3A4 and has weak potential to inhibit CYP2D6 and 2C19.

As described in the attached EU and FDA product information of Faslodex®, there are no known DDIs of fulvestrant.

Fulvestrant does not significantly inhibit any of the major CYP isoenzymes *in vitro*, including CYP1A2, 2C9, 2C19, 2D6, and 3A4, and studies of co-administration of fulvestrant with midazolam indicate that therapeutic doses of fulvestrant have no inhibitory effects on CYP 3A4 or alter blood levels of drug metabolized by that enzyme. Although fulvestrant is partly metabolized by CYP3A4, a clinical study with rifampin, a potent inducer of CYP3A4, showed no effect on the PK of fulvestrant. Also results from a healthy volunteer study with ketoconazole, a potent inhibitor of CYP3A4, indicated that ketoconazole had no effect on the PK of fulvestrant and dosage adjustment is not necessary in patients co-prescribed CYP3A4 inhibitors or inducers. Since CT7001 is not expected to affect the PK of fulvestrant, the standard dosing regimen of fulvestrant (i.e., 500 mg, intramuscularly on Days 1 and 15 of Cycle 1 and then on Day 1 of each subsequent 28-days Cycle) will be used in the present study, be it in combination with [redacted] CT7001.

Nonetheless, the current study will start with a Phase 1b part (called Part A) [redacted]. The starting dose of CT7001 in Part A will be 240 mg, which is approximately 33% lower than the current recommended preliminary Phase 2 dose of 360 mg OD. Fulvestrant will be administered at full standard dose of 500 mg given intramuscularly (IM) every four weeks, with an additional 500 mg dose given two weeks after the first dose. Throughout the study blood samples will be collected to analyse trough concentrations of CT7001 and fulvestrant and compare CT7001 concentrations with data from monotherapy studies and fulvestrant concentrations in the patient groups receiving fulvestrant plus CT7001 [redacted].

Pre/peri-menopausal women in this study must receive treatment with an LHRH agonist as auxiliary therapy, to be given as per standard institutional practice in this patient population and starting at least 4 weeks prior to first dose of CT7001 [redacted] (see Section 4.1). There are no known DDIs of fulvestrant and LHRH agonists and when combined the two drugs are generally being given at full individual doses.

Likewise, the sponsor considers the potential for a clinically significant DDI between CT7001 and an LHRH agonist to be very low. LHRH agonists are synthetic decapeptide analogue of gonadotropin releasing hormone whose primary route of elimination is the cleavage of C-terminal amino acids followed by renal excretion. No formal drug-drug interaction studies have been performed and no confirmed interactions have been reported between LHRH agonists and other drugs (prescribing information Zoladex®, Lupron® , Eliguard®, Trelstar®, Vantas® Supprelin® , Decapeptyl® , Gonapeptyl®).

3 STUDY OBJECTIVES AND ENDPOINTS

3.1 Objectives

Primary Objective, study Part A

- To determine the recommended Phase 2 dose of CT7001 given in combination with fulvestrant at 500 mg.

[redacted]

Secondary Objectives

- To evaluate safety and tolerability (all study parts).
- To evaluate the trough concentrations of CT7001 when used in combination with fulvestrant compared to historical CT7001 data (all study parts).
- To evaluate correlations between CT7001 exposures and efficacy/safety findings in this patient population (all study parts).
- [redacted]
- To evaluate the incidence and type of genotypes and copy number variation of CYP2D6 and other CYP genes and of drug transporter genes that may be involved in the metabolism of CT7001 (all study parts).

Exploratory Objectives (all study parts)

- To further investigate the presence and identity of metabolites of CT7001 and, if appropriate, characterize their PK.
- To further evaluate the impact of patient characteristics (including but not limited to race, ethnicity, age, CYP and drug transporter polymorphisms) on trough concentrations of CT7001 (all study parts).
- To evaluate correlations between CT7001 exposures and efficacy/safety findings in this patient population (all study parts).
- To further explore mutations and expression in genes, proteins and RNAs relevant to the cell cycle (e.g., phosphorylation of CDK1 and Rb proteins), drug target engagement (e.g., c-Myc, MCL-1) and tumour sensitivity and/or resistance in tumour-derived materials including circulating tumour DNA and tumour tissue (e.g., p53, CDK7, ER, ESR1, AR, PIK3CA) and their potential impact on efficacy.

3.2 Endpoints

Primary Endpoint, study Part A

- Dose-limiting toxicities and type, incidence, severity (as graded by CTCAE v5.0), seriousness and relationship to study medications of adverse events and any laboratory abnormalities.

[redacted]

Secondary Endpoints

- Objective Response (study Part A [redacted]).
- Duration of Response (study Part A [redacted]).
- Clinical Benefit Response (complete or partial response, or stable disease ≥24 weeks; all study parts).
- Best percent change in tumour size.
- [redacted]
- Trough plasma concentrations of CT7001 (all study parts).
- Trough plasma concentrations of fulvestrant (study Part A[redacted]).
- Incidence and type of genotypes and copy number variation of CYP2D6, other CYP genes and drug transporter genes that may be involved in the metabolism of CT7001 (all study parts).

Exploratory Endpoints (all study parts)

- Metabolites of CT7001.
- Biomarkers in PBMC, circulating tumour DNA and tumour tissue, including genes, RNA expression and proteins (e.g., c-Myc, MCL-1, phosphorylated CDK1 and Rb proteins, p53, CDK7, ER, ESR1, AR, PIK3CA).
- Effect of patient characteristics (including but not limited to race, ethnicity, age, CYP and drug transporter polymorphisms) on trough concentrations of CT7001 (all study parts).
- Correlations between CT7001 exposures and efficacy/safety findings in this patient population (all study parts).

4 STUDY POPULATION

Subjects who fail screening may be re-screened only on approval of the SRC and Sponsor. Any subject re-screened will need to provide new informed consent and will be allocated a new subject number.

4.1 Inclusion Criteria

To be eligible for the study patients must meet **all** the following criteria:

1. Women 18 years of age or older who are either:
   - Postmenopausal, as defined by one of the following criteria:
     - Age ≥60 years;
     - Age 50-59 years of age amenorrhoeic for at least 12 months after cessation of all exogenous hormonal treatments, with no alternative pathological or physiological cause <u>or</u> have serum oestradiol and FSH level within the laboratory's reference range for postmenopausal females;
     - Age <50 years of age amenorrhoeic for at least 12 months following cessation of exogenous hormonal treatments, with no alternative pathological or physiological cause <u>and</u> serum oestradiol and FSH level within the laboratory's reference range for postmenopausal females;
     - Documented bilateral oophorectomy;
     - Medically confirmed ovarian failure.

     OR
   - Pre/peri-menopausal, i.e., not meeting the criteria for being postmenopausal, if amenable to treatment with an LHRH agonist.
     - LHRH agonists are considered a mandatory auxiliary treatment in this sub-population and is to be given in accordance with the locally approved label of the drug and routine institutional practice. Patients must have commenced treatment with an LHRH agonist at least 4 weeks prior to first dose of CT7001[redacted].
2. No childbearing potential, defined as women:
   - Who had prior hysterectomy or bilateral surgical oophorectomy or are medically postmenopausal (defined as in inclusion criteria 1).
3. Women of childbearing potential must be willing to practice effective contraception (defined as abstinence, sex only with person of the same sex, sex only with vasectomized partner, intrauterine device, or barrier method [e.g., condom, diaphragm] for the duration of the study and for 24 months after the last study dose.

- The drug-drug interactions of CT7001 are not fully characterized so hormonal contraceptives that are prone to drug-drug interactions may not be effective because of a potential CYP3A4 interaction with CT7001.

4. Women of childbearing potential must have a negative serum pregnancy test at baseline (within 7 days prior to first dose of CT7001 [redacted]).

5. Histologically confirmed diagnosis of carcinoma of the breast with evidence of metastatic or locally advanced disease, not amenable to resection or radiation therapy with curative intent.

6. Documentation of ER-positive and/or PgR-positive tumour based on most recent tumour biopsy utilizing an assay consistent with local standards.

   - ER- and PgR-positivity is defined as ≥1% positive stained cells (ASCO/CAP guidelines, Hammond et al, 2010).

7. Documentation of HER2 negativity based on local testing on most recent tumour biopsy.

   - HER2-negativity is defined as immunohistochemistry score 0/1+ or negative by in situ hybridization (FISH/CISH/SISH) defined as a HER2/CEP17 ratio <2 or for single probe assessment a HER2 copy number <4 (ASCO/CAP guidelines, Wolff et al., 2018).

   - In case no tumour biopsy was performed after initial resection of the primary tumour, the original tumour tissue serves as basis for assessment of ER/PgR and HER2 status.

   - Assessment of ER, PgR and HER2 status will be based on results from local pathology laboratories. Independent central review is not intended.

8. **Part A Only:** Measurable disease as defined by Response Evaluation Criteria in Solid Tumours (RECIST) version 1.1 (Appendix C).

9. Patients must have documented objective disease progression while on or within 6 months after the end of the most recent therapy.

[redacted]

10. Patients must have received an aromatase inhibitor together with a CDK4/6 inhibitor in the same line of therapy for the treatment of:

    - locally advanced or metastatic disease or
    - early breast cancer, if the disease-free interval, between initiation of adjuvant therapy and first line treatment of locally advanced or metastatic disease was < 12 months.

    In addition, the following prior therapies for locally advanced or metastatic disease are allowed:

    - Everolimus (Part A Only)

- No more than two lines of endocrine treatment (prior fulvestrant is not allowed)
- No more than one line of prior chemotherapy for locally advanced or metastatic disease (Part A Only).

[redacted]

12. ECOG performance status 0 or 1 with no deterioration over the previous 2 weeks.
13. Expected life expectancy of greater than 12 weeks.
14. Ability to swallow and retain oral medication and receive intramuscular injections.
15. Patients are willing and able to comply with scheduled visits, treatment plan, laboratory tests, and other study procedures.
16. Evidence of personally signed and dated written informed consent indicating that the patient has been informed of all pertinent aspects of the study before any study-specific activity is performed.

Host Genetics Research Study: Pharmacogenomics Samples (Optional)

Patients who meet all of the following criteria may be included in optional genetics sub-studies:

1. Provision of signed and dated, written informed consent for the genetic research.

4.2 Exclusion Criteria

To be eligible for the study patients **may not have any** of the following exclusion criteria:

1. Prior therapy with fulvestrant.

[redacted]

2. More than 2 lines of endocrine treatment for locally advanced or metastatic disease.

[redacted]

5. Patients with liver metastasis may only be enrolled upon approval by the medical monitor:

- **Part A:** On 11th September 2020 the DMC approved the request to not enrol further patients with liver metastasis into Part A
- [redacted]

6. Inadequate hepatic, renal, bone marrow or cardiac function, specified as follows:
   - Hepatic (any of below):
     - AST and/or ALT > 2.5 × the ULN or > 5.0 × ULN for patients with liver metastases.
     - Alkaline phosphatase > 2.5 x ULN or > 5 x ULN if bone or liver metastases present.
     - Total bilirubin > 1.5 × ULN (>3.0 x ULN if known Gilbert's disease).
     - Albumin < 30 g/L.
     - Liver function deteriorating at a speed that would likely make the subject not meeting the AST, ALT, bilirubin or albumin levels specified above at the time of the first study dose.
     - Other evidence of impaired hepatic synthesis function.
   - Renal:
     - Serum creatinine > 1.5 × ULN.
   - Bone marrow (any of below):
     - Absolute neutrophil count ≤ 1.5 × 10^9/L (≤ 1.500/mm^3).
     - Platelet count <100 × 10^9/L (< 100,00/mm^3).
     - Haemoglobin < 90 g/L (< 9 g/dL).
   - Cardiac (any of below):
     - Myocardial infarction within 6 months of study entry, unstable angina, unstable arrhythmia.
     - New York Heart Association Class > I heart failure (see Appendix D).
     - Mean resting QT interval corrected for heart rate by the Fridericia formula (QTcF) > 470 msec obtained from 3 ECGs obtained within 3-5 minutes apart.
     - Clinically important abnormalities in rhythm, conduction, or morphology on resting ECG (e.g., complete left bundle branch block, third degree heart block).
       - Controlled atrial fibrillation is permitted.
     - Any factor that may increase the risk of QTc prolongation or of arrhythmic events (e.g., hypokalaemia, congenital long QT syndrome,

immediate family history of long QT syndrome or unexplained sudden death under 40 years of age).

7. Unresolved toxicity (except alopecia) from prior therapy of ≥ Grade 2 according to Common Terminology Criteria for Adverse Events (CTCAE) version 5.0.
8. Advanced visceral metastases if risk of life-threatening complications in the short term (including patients with massive uncontrolled effusions [pleural, pericardial, peritoneal], pulmonary lymphangitis and over 50% liver involvement).
9. Known symptomatic CNS metastases, carcinomatous meningitis or leptomeningeal disease.
   - Patients with a history of CNS metastases or spinal cord compression due to metastasis are eligible if they have been treated with local therapy (e.g., radiotherapy, stereotactic surgery) and are clinically stable off anticonvulsants and steroids for at least 4 weeks before first dose of CT7001 [redacted].
10. Refractory nausea and vomiting, chronic gastro-intestinal disease or previous significant bowel resection, with clinically significant sequelae that would preclude adequate absorption of CT7001.
11. Uncontrolled seizures.
12. Active infection requiring systemic antibiotic, antifungal, or antiviral medication within14 days prior to allocation to study therapy.
13. Receipt of systemic corticosteroids (at a dose > 10 mg prednisone/day or equivalent) within 14 days before the first dose of IMP.
14. Has received a live-virus vaccination within 28 days or less of planned treatment start. Note: seasonal flu vaccines that do not contain live virus are permitted.
15. Severe or uncontrolled medical condition (e.g., severe chronic obstructive pulmonary disease, severe Parkinson's disease, active inflammatory bowel disease or psychiatric condition).
16. Active bleeding diatheses.
17. History of haemolytic anaemia or marrow aplasia.
18. Renal or other organ transplant.
19. Known hepatitis B, hepatitis C, or human immunodeficiency virus infection.
20. Pregnancy.
21. Breastfeeding.
22. Non-biological anti-cancer medicines within 28 days or ≤ 5 half-lives, whichever is shorter, before the first study dose.
23. Biological anti-cancer medicines (e.g., monoclonal antibodies, antibody-drug conjugates) within 42 days before the first study dose.

24. Receipt of St John's Wort within 21 days before the first study dose.
25. Concomitant medication, herbal supplement or food that is a strong inhibitor or inducer of CYP3A4, CYP2C19, CYP2D6, or P-glycoprotein activity within 14 days before the first dose of CT7001 (specific examples listed in protocol Appendix B).
26. Receipt of a blood transfusion (blood or blood products) within 14 days before the first study dose of IMP.
27. Known hypersensitivity to CT7001, fulvestrant or any excipient of the investigational products.
28. Diagnosis of any other malignancy within 3 years prior to enrolment, except for adequately treated basal cell or squamous cell skin cancer, or carcinoma in situ of the cervix.
29. In the opinion of the Investigator, unlikely to comply with study procedures.
30. Patients who are investigational site staff members directly involved in the conduct of the trial and their family members, site staff members otherwise supervised by the Investigator, or patients who are Carrick employees directly involved in the conduct of the trial.

Host Genetics Research Study - Pharmacogenomics Samples (Optional)

Patients who meet any of the following criteria will be excluded from optional genetic sub-studies:

1. Allogenic bone marrow transplant.
2. Non-leucocyte-depleted whole blood transfusion within 120 days of the date of the genetic sample collection.

5 STUDY DESIGN

The present study is an international, multicentre Phase 1/2 study in patients with metastatic or locally advanced HR-positive and HER2-negative BC. The study will have three parts. In each part, patients must meet all the eligibility criteria described in Section 4 and will receive study treatment until objective disease progression (Section 8.1.2), symptomatic deterioration, unacceptable toxicity, death, withdrawal of consent or completion of Module 2 primary endpoint, whichever occurs first.

The overall study design is illustrated in the following schema.

Study Design

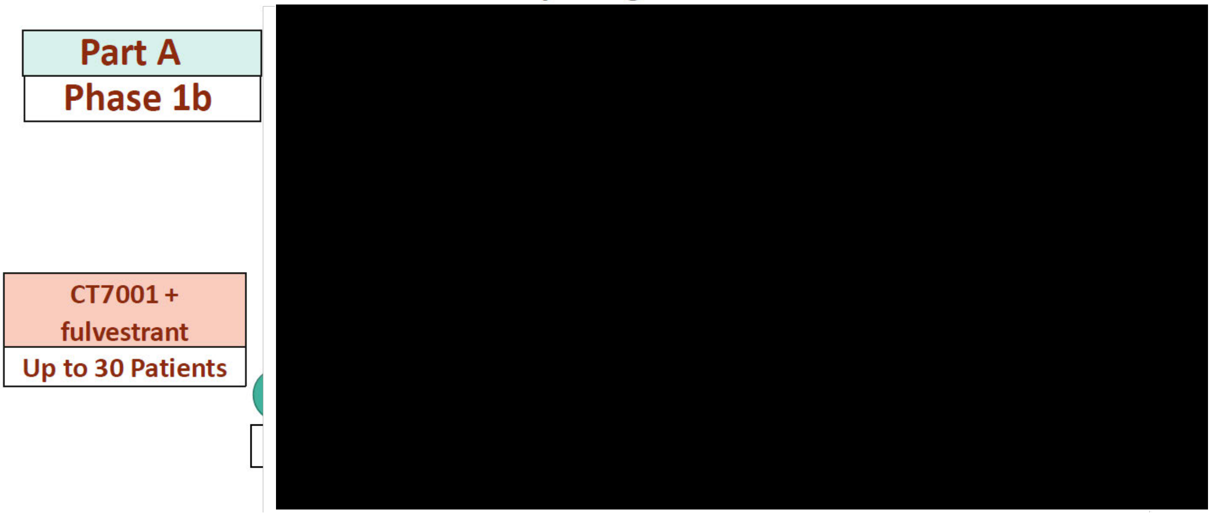

[redacted]

In each part of the study a treatment cycle will be defined as 28 days. CT7001 [redacted] will be administered orally OD.

Module 4 evaluated the effect of food on the bioavailability of CT7001 in cancer patients. On 10th June 2019 the Safety Review Committee reviewed the PK data and determined there was no significant effect observed in AUC in the fed phase of the Module 4, the requirement to fast was removed. CT7001 may be taken orally in either a fasted or fed state, where a patient experiences nausea or vomiting Investigators are recommended to advise their patients to consume CT7001 after a meal.

Throughout the study the dose of fulvestrant will be fixed at the standard dose of 500 mg administered IM at intervals of 28 ± 2 days with an additional 500 mg dose given 14 ± 2 days after the first dose. Pre-and peri-menopausal women must have commenced treatment with an LHRH agonist at least 4 weeks prior to first dose of CT7001 [redacted]. Every effort should be made to administer the LHRH agonist on site at the time of fulvestrant administration.

The DMC will monitor the safety data on a periodic basis. The DMC will make recommendation as to whether the trial should continue based on ongoing reviews of safety data. In Part A (Section 5.1), the DMC approved second CT7001 dosing cohort on 07 February 2020 as no Dose Limiting Toxicities were observed in the first cohort. DMC will determine which dosing regimen to advance to Part B and will also review the efficacy data on a periodic basis.

Module 2 Part A completed enrolment on 23 March 2021 with 31 patients dosed. [redacted]

The study will continue while these periodic analyses and reviews are ongoing.

Patients will undergo regular safety and efficacy assessments as outlined in the Schedule of Events. Primary efficacy analyses will be performed based on the local radiologist's/investigator's tumour assessments, using RECIST version 1.1 (Appendix C). If required, supportive efficacy analyses may be performed by BICR. Efficacy analyses in this study will not include survival.

Tumour assessments will continue until radiographically and/or clinically (i.e., for photographed or palpable lesions) documented progressive disease as per RECIST version 1.1 (Section 8.1.2), death, discontinuation of patient from overall study participation (e.g., patient's request, lost to follow-up), initiation of new anticancer therapy (Parts A [redacted]) or Module 2 LPLV is met, whichever occurs first. [redacted]

All parts of the study will include sparse blood sampling for analysis of CT7001 and fulvestrant trough concentrations. Various members of the CYP family play a role in the hepatic metabolism of CT7001, and drug transporters may affect bioavailability. Accordingly, genotyping of CYP and drug transporter genes will be considered a mandatory study procedure.

All parts of the study will include a set of optional procedures and analyses which require separate informed consent. These include baseline biopsies of readily accessible tumour lesions to analyse various gene mutations, RNA expression profiles and expression of various proteins and their potential impact on efficacy. [redacted] In all parts of the study baseline blood samples will be collected for various molecular analysis, some to be retained for potential future research and pharmacogenomic testing, respectively (unless prohibited by local laws or regulations).

Patients will be given the opportunity to provide feedback in relation to their clinical trial experience at the end of their study participation.

5.1 Part A

Part A is an open-label, single-arm, ascending dose Phase 1b study to determine the dosing regimen of CT7001 and fulvestrant to be taken to subsequent randomized Phase 2 testing in Part B. Before taking a particular dosing regimen to Part B, at least 6 patients in Part A should have received that regimen and at least 3 patients should have completed ≥ 2 cycles. The decision algorithm described below is based on DLTs recorded in the first cycle. Patients are considered evaluable if they completed the first cycle or discontinued therapy in the first cycle due to DLT. Patients in a cohort will be replaced if they cannot complete the first cycle unless due to a DLT.

Part A is planned to have two dose cohorts with up to 6 evaluable patients to be enrolled per cohort to determine the dose to take to part B and/or expand the cohort. In each cohort, the dose

of fulvestrant will be fixed at the standard dose of 500 mg given at intervals of 28 ± 2 days with an additional 500 mg dose given 14 ± 2 days after the first dose. Fulvestrant will be administered as two consecutive slow IM injections (1-2 minutes) of 250 mg in 5 mL, one in each buttock (gluteal area).

Cohort 1 will test CT7001 at 240 mg OD, which is approximately 33% lower than the preliminary recommended Phase 2 dose as monotherapy (360 mg). In case no DLT is recorded in Cycle 1 in the first 3 evaluable patients, cohort 2 will commence enrolment at 360 mg of CT7001 OD. In case of 1/3 patients have a DLT in cohort 1, a further 3 evaluable patients will be assessed. If < 2/6 patients will have a DLT, cohort 2 will start enrolment.

In case ≥ 2/3 or ≥ 2/6 patients in cohort 1 experience a DLT, dose escalation MUST be stopped. The DMC will then determine if patients in Part A and in Part B can only be administered a lower dose, or a different dosing regimen. A new, lower dose, or a justification for a different dosing regimen at the same dose MUST require approval of a substantial amendment.

In case 0/3 or 1/3 evaluable patients in cohort 2 experience a DLT, the cohort will expand to six patients. In case ≥ 2/3 or ≥ 2/6 patients experience a DLT, the cohort 1 dosing regimen (CT7001 at 240 mg and fulvestrant at 500 mg) will be determined as the preliminary Phase 2 dosing regimen. However, before taking to Part B, at least 6 patients should be treated with that regimen in Part A and at least 3 should have completed at least 2 cycles.

In case <2/6 evaluable patients in cohort 2 have a DLT and at least 3 patients have completed at least 2 cycles, this will be considered the recommended Phase 2 regimen and taken to Part B testing.

The DMC will determine whether and when to open the second CT7001 dosing cohort and which dosing regimen to advance to randomized Phase 2 testing in Part B. In case the DMC may consider safety and/or tolerability data as being borderline for taking a dosing regimen to Part B, additional patients in sets of 3 may get enrolled in Part A for further assessment.

Part A is estimated to require approximately 12 patients who completed Cycle 1 to confirm the recommended phase 2 dose (RP2D) to progress to Part B. Following RP2D selection by the Data Monitoring Committee (DMC) additional patients will be enrolled into Module 2A which is anticipated to yield 20 evaluable patients at the RP2D.

Module 2 Part A completed enrolment on 23 March 2021 with 31 patients dosed. [redacted]

Definition of Dose-limiting Toxicity

A DLT is defined as any toxicity not attributable to the disease or disease-related processes under investigation, which occurs before the end of Cycle 1 and which includes:

- Haematological toxicities:
  - Grade 4 neutropenia (ANC < 500 cells/mm^3) lasting longer than 4 consecutive days
  - Grade 3 neutropenia (ANC ≥500 to <1000 cells/mm^3) of any duration accompanied by fever ≥ 38.5°C or systemic infection

  - Grade 3 thrombocytopenia (25,000 to <50,000 cells/mm^3) with bleeding
  - Any other confirmed haematological toxicity ≥ CTCAE Grade 4 (a repeat may be required for confirmation of an isolated abnormality in the absence of clinical signs, symptoms or other abnormal investigations, i.e., a suspected spurious value).

- Non-haematological toxicity ≥ CTCAE Grade 3 including:
  - Laboratory abnormalities (a repeat may be required for confirmation of an isolated abnormality in the absence of clinical signs, symptoms or other abnormal investigations, i.e., a suspected spurious value)
  - QTcF prolongation (> 500 msec and /or + 60 ms)
  - Any other toxicity that is greater than that at Baseline and is clinically significant or unacceptable or does not respond to supportive care
  - Any event, including significant dose reductions or omissions, judged to be a DLT by the SRC.

The definition of a DLT excludes:

- Alopecia of any grade.
- Inadequately treated Grade 3 nausea, vomiting, or diarrhoea (all patients should receive optimal antiemetic or antidiarrhoeal prophylaxis or treatment).
- Any toxicity clearly unrelated to CT7001 treatment, e.g., solely related to the disease or disease-related process under investigation.
- Isolated laboratory changes of any grade without clinical sequelae or clinical significance.

[redacted]

[redacted]

6 STUDY TREATMENT

In each part of the study, study treatment will continue until any of the events described in Section 7.6.1 occurs.

6.1 Drug Supply

The investigational products (IPs) used in this trial are CT7001 (Samuraciclib)[redacted] and fulvestrant, all of which will be supplied by the sponsor.

6.1.1 Pharmaceutical Properties of CT7001

Chemical (IUPAC) name:

(3R,4R)-4-[[[7-(benzylamino)-3-isopropyl-pyrazolo[1,5-a]pyrimidin-5-yl]amino]methyl]piperidin-3-ol

Laboratory Code: CT7001, ICEC0942

International Non-Proprietary Name: Samuraciclib

Structural formula:

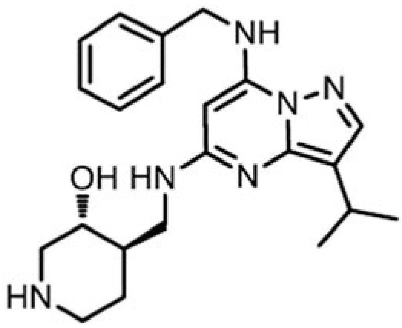

6.1.2 Pharmaceutical Properties of Fulvestrant

Please refer to the SPC of Faslodex® attached in Appendix E.

6.1.3 Formulation, Packaging and Storage of CT7001[redacted] and Fulvestrant

CT7001 will be supplied as capsules containing 60 mg or 120 mg equivalents of CT7001 free base and the excipients listed in Table 2.

Table 2: Content of CT7001 Capsules

Material	(%w/w)
CT7001 (drug substance)	30.00
Microcrystalline Cellulose (Avicel PH 102)	64.50
Sodium starch glycolate (Explotab®)	5.00
Magnesium Stearate (Hyqual®)	0.25
Silica Colloidal Anhydrous (Aerosil 200)	0.25
**Total**	100.00

[redacted]

[redacted]

The capsules at each strength are opaque white hydroxypropyl-methylcellulose capsule shells. Capsules with 60 mg are size 1 and capsules with 120 mg size 00, respectively.

The sponsor, through its delegate will supply the oral drug formulations to sites in high-density polyethylene bottles containing 60 mg or 120 mg capsules. Bottles are secured with a child-resistant and tamper-evident closure.

Commercially available fulvestrant will be provided in its commercially approved primary packaging, relabelled as IP and supplied to sites by the sponsor. Fulvestrant is supplied as two 5-mL clear neutral glass (Type 1) barrels, each containing 250 mg/5 mL of fulvestrant solution for intramuscular injection and fitted with a tamper evident closure. The syringes are presented in a tray with polystyrene plunger rod and safety needles (SafetyGlideTM) for connection to

the barrel. Complete information about the fulvestrant formulation can be found in the SPC for Faslodex® attached in Appendix E.

All IPs will be kept in a secure place under appropriate storage conditions as specified on the IP labels.

6.2 Dispensing of CT7001 [redacted]

An Interactive response system (IXRS) will be used to allocate CT7001[redacted] and Fulvestrant medication kits to the patient, full details can be found in the IXRS Reference Manual.

Site personnel must ensure that patients clearly understand the directions for self-medication. Patients should be given a sufficient supply to last until their next study visit. Patients should be instructed to finish one bottle of study drug before opening a new bottle. Unused drug and/or empty bottles should be returned to the site at the next study visit.

Patients should be instructed to keep their medication in the provided bottles and not transfer it to any other container.

6.3 Dosing Regimen of CT7001 [redacted]

In this study, a treatment cycle is defined operationally as 28 days. Each daily dose should be taken around a similar time of the day. The time of the day is at the patient's discretion.

As described in Section 5, the starting dose of CT7001 in Part A will be 240 mg OD. Due to lack of DDI and overlapping toxicity, it is considered likely that the CT7001 dose can be escalated to 360 mg and that this will be the dose to be used in combination with fulvestrant in Part B and C. Module 2 Part A completed enrolment on 23 March 2021 with 31 patients dosed. [redacted]

6.4 Drug Administration of CT7001 [redacted]

CT7001 may be taken orally in either a fasted or fed state.

Where a patient experiences nausea or vomiting Investigators are recommended to advise their patients to consume CT7001 after a meal.

Site personnel must ensure that patients clearly understand the directions for self-medication. Patients should be instructed to swallow CT7001 [redacted] capsules whole and not to chew them prior to swallowing. No capsule should be ingested if it is broken, cracked, or otherwise not intact.

Patients will be asked to capture their medication intake, including whether they were fed or fasted, in a medication diary.

6.5 General Rules for CT7001 [redacted]

Patients who miss a day's dose must be instructed NOT to 'make it up' the next day.

Patients who vomit any time after taking a dose must be instructed NOT to 'make it up' but rather resume treatment the next day as prescribed.

6.6 Medication Dosing Errors of CT7001 [redacted]

Medication dosing errors may mainly result from the administration of CT7001 [redacted] at the wrong dosage strength. Such medication errors are to be captured on the IP Administration electronic case report form (eCRF) which is a specific version of the adverse event (AE) page, and on the SAE form when appropriate. In the event of medication dosing error, the sponsor should be notified immediately.

6.7 Dosing Regimen and Drug Administration of Fulvestrant

Fulvestrant will be dosed at 500 mg given at intervals of 28 ± 2 days with an additional 500 mg dose given 14 ± 2 days after the first dose. A treatment cycle is defined as 28 days.

Fulvestrant is supplied as two 5-mL clear neutral glass (Type 1) barrels, each containing 250 mg/5 mL of fulvestrant solution for intramuscular injection and fitted with a tamper evident closure. The syringes are presented in a tray with polystyrene plunger rod and safety needles (SafetyGlideTM) for connection to the barrel.

Fulvestrant will be administered as two consecutive slow intramuscular (IM) injections (1-2 minutes) of 250 mg in 5 mL, one in each buttock (gluteal area). Drug preparation and administration will be performed at the study site by a physician, registered nurse or other qualified health care provider.

Refer to the SPC for Faslodex® attached in Appendix E for instructions and steps necessary for drug preparation and administration.

6.8 Drug Diary for LHRH agonist

In order to reduce, the number of clinic visits for study patients, every effort should be made to administer the LHRH agonist at the study site at the time of the fulvestrant administration. In case the LHRH agonist will not be administered at the study site, the patient will receive a second drug diary to document the LHRH agonist injection. The completed diary must be returned to the site at the next study visit.

6.9 Overdose of CT7001[redacted]

There are no clinical data yet on overdose with CT7001 as this has not occurred to date. There is no definition of what constitutes an overdose. There is no known antidote.

Any patient who receives a higher dose of CT7001[redacted] than intended should be monitored closely, managed with appropriate supportive care, and followed-up expectantly. Such incidence should be recorded as an overdose as described in Section 6.11.

6.10 Overdose of Fulvestrant

As per Faslodex® label, human experience of overdose with Faslodex® is limited. No adverse reactions were seen in healthy male and female volunteers who received intravenous fulvestrant, which resulted in peak plasma concentrations at the end of the infusion that were approximately 10 to 15 times those seen after intramuscular injection. The potential toxicity of fulvestrant at these or higher concentrations in cancer patients who may have additional

comorbidities is unknown. There is no specific treatment in the event of fulvestrant overdose, and symptoms of overdose are not established.

Any patient who receives a higher dose of fulvestrant than intended should be monitored closely, managed with appropriate supportive care, and followed-up expectantly.

6.11 Recording of Overdose

Overdoses will be recorded in the eCRF as follows:

- An overdose with associated AEs will be recorded as an AE of the relevant diagnosis/symptoms on the AE eCRF page and in the overdose eCRF page.
- An overdose with no associated symptoms will be reported only on the overdose eCRF page.
- If an overdose occurs, the Investigator or other site personnel must notify the relevant CRO Medical Monitor immediately but no later than by the end of the next business day of first awareness.

The Medical Monitor will work with the Investigator to ensure that all relevant information is provided to the safety database: drug.safety@bionical-emas.com

For overdoses associated with an SAE, standard reporting timelines apply (see Section 9.12.1). Other overdoses will be reported within 28 days.

6.12 Dose Modification

Every effort should be made to administer study treatment on the planned dose and schedule. However, in the event of significant treatment-related toxicity, administration of CT7001 [redacted] may need adjustment as described in the following sections.

Dose adjustment is permitted only for CT7001[redacted]. The fulvestrant dose cannot be adjusted, its dosing can only be delayed or interrupted (see Section 6.12.1).

Patients are to be instructed to notify Investigators at the first occurrence of any adverse sign or symptom. In the event of significant treatment-related toxicity, CT7001[redacted] dosing may be interrupted, delayed and/or reduced as described below. In the event of multiple toxicities, dose modification should be based on the worst toxicity observed.

No specific dose adjustments are recommended for Grade 1-2 treatment-related toxicity. However, investigators should always manage their patients according to their best medical judgment based on the specific clinical circumstances.

6.12.1 Fulvestrant

The fulvestrant dose cannot be adjusted, dosing can only be delayed or interrupted. Delay or interruption of fulvestrant dosing will be determined by the investigator in accordance with the label. A fulvestrant injection can be skipped in case of a fulvestrant-related toxicity, or dosing can be delayed. Treatment delay for fulvestrant-related toxicities will be performed as per the investigator's best medical judgement. If delay of longer than 7 days is required, then the dose

should be skipped. In the event of a toxicity requiring dosing delay of CT7001[redacted], fulvestrant can also be delayed as the investigator may consider necessary for satisfactory recovery of toxicity.

Fulvestrant should not be administered if the platelet count is <50 × 10^9/L (<50,000/mm^3).

6.12.2 Types of Dosing Modifications of CT7001[redacted]

Dosing may be stopped, interrupted (within a cycle) or delayed (at start of a next cycle) and the dose may be reduced.

Note to the following Sections 6.12.2.1, 6.12.2.2, 6.12.2.3 and 6.12.2.4:

- MUST = mandatory
- SHOULD = not mandatory but highly recommended
- MAY = per the investigator's best clinical judgment

6.12.2.1 Mandatory Treatment Discontinuation

- Grade 4 QTc abnormalities (torsades de pointes, polymorphic ventricular tachycardia, signs and/or symptoms of serious arrhythmia).

6.12.2.2 Dosing Interruption or Delay

The first measure of dose modification is interruption (within a cycle) or delay (at start of a next cycle) of dosing.

Patients experiencing the following adverse events MUST have their treatment interrupted or delayed:

- Grade 3 neutropenia (ANC < 1.0 × 10^9/L) associated with a documented infection or fever ≥ 38.5°C.
- Grade 4 neutropenia (ANC < 0.5 x 10^9/L).
- Grade ≥ 3 thrombocytopenia (platelet count < 50 x 10^9/L).
- Grade ≥ 3 anaemia (Hb < 80 g/L and transfusion indicated).
- Grade ≥ 3 diarrhoea, oral mucositis, vomiting or nausea if persistent despite optimal medical treatment.
- Grade ≥ 3 other non-haematological toxicity if persistent despite optimal medical treatment.
- Grade 3 average QTc prolongation (QTc ≥ 501 msec or > 60 msec change from baseline) corrected for heart rate by the Fridericia formula.

Appropriate follow up assessments should be performed and proper therapy and medical care, as clinically indicated, should be provided. If a treatment delay results from a decline in haematological parameters, the frequency of laboratory assessments should be increased as clinically appropriate.

Following Grade ≥ 3 thrombocytopenia (platelet count < 50 x 10^9/L), minimally weekly haematological monitoring for 4 weeks MUST be performed after resuming treatment with IMP and additionally as clinically indicated. Whilst not mandated, it would be desirable for

patients experiencing thrombocytopenia to have a peripheral blood smear performed and undertake a bone marrow biopsy to further elucidate any underlying pathology.

6.12.2.3 Restart of Treatment

Restart of treatment within a cycle or start of a next cycle SHOULD occur when the following parameters have been met:

- ANC ≥ 1.0 × 10^9/L, no fever and full resolution of a documented infection.
- Platelet count ≥ 100 × 10^9/L.
- Hb ≥ 80 g/L.
- Grade ≤ 1 diarrhoea, oral mucositis and vomiting.
- Grade ≤ 2 nausea.
- QTc < 481 msec corrected for heart rate by the Fridericia formula and potential contributing causes (e.g., electrolyte imbalance, concomitant medications known to prolong QTc) corrected.

When treatment is resumed after Grade ≥ 3 thrombocytopenia (platelet count < 50 x 10^9/L), the dose of CT7001 [redacted] MUST not exceed 240mg OD.

In case recovery of the other toxicities takes more than 21 days, permanent discontinuation of CT7001 SHOULD be strongly considered. Treatment resumption for patients recovering from treatment-related toxicity after more than 21 days of treatment interruption or cycle delay but deemed to be deriving obvious clinical benefit per the investigator's best medical judgment is left at the investigator's discretion.

Depending on when a toxicity resolved, a treatment interruption may lead to the patient missing all subsequent planned doses within that same cycle. Doses omitted for toxicity are not to be replaced within the same cycle.

In the event of a treatment interruption or cycle delay for reasons other than treatment-related toxicity (e.g., non-cancer related surgery) lasting >2 weeks, treatment resumption will be decided in consultation with the sponsor.

6.12.2.4 Dose Reductions

Following dose interruption or cycle delay the dose of CT7001 [redacted] may need to be reduced when treatment is resumed.

As noted in Section 6.12.2.1, in case of Grade 4 QTc abnormalities study therapy MUST be discontinued permanently.

Prior to concluding that an episode of prolongation of the QTc interval (Grade ≤3) is due to study drug, thorough consideration should be given to potential precipitating factors (e.g., change in patient clinical condition, effect of concurrent medication, electrolyte disturbance) and possible evaluation by specialist. If IP causality cannot be ruled out, dose reduction as described below should be performed upon restart of therapy.

With the exception of thrombocytopenia, when treatment is resumed after sufficient resolution of the other toxicities listed in Section 6.12.2.2, dose reduction SHOULD be considered but is at the investigator's discretion. Factors to take into account include, the time it took to recover from a given toxicity, clinical sequelae associated with a toxicity and overall risk-benefit assessment per the investigator's best clinical judgment.

In case the investigator considers a dose reduction, indicated for other reasons this requires prior discussion and agreement with the sponsor.

A maximum of two dose reductions of CT7001 will be allowed per subject. Subjects requiring more than 2 dose reductions will be discontinued from the study and entered, into the follow-up phase unless they meet the criteria for use of Fulvestrant monotherapy (Section 6.15).

Following the second dose reduction, and at the investigator's discretion, the patient may be re-challenged at the first dose reduction strength at a later date.

Table 4 describes the recommended dose reductions for two different dose options of CT7001[redacted].

Table 4: Two Dose Reduction Levels for Two Potential Starting Doses of CT7001[redacted]

Starting dose	360 mg OD	240 mg OD
First dose reduction	240 mg OD	120 mg OD
Second dose reduction	120 mg OD	Not Applicable

OD = once daily

Table 5 describes the number and strength of capsules to be taken for each possible dose.

Table 5: Number and Strength of Capsules for Each Possible Dose

Dose	120 mg Capsules	60 mg Capsules*
360 mg	3	6
240 mg	2	4
120 mg	1	2

* All patients should be prescribed 120 mg capsules in the first instance. In the event a patient experiences difficulty in swallowing 120 mg capsules then 60 mg capsules will be made available. Please refer to the Pharmacy Manual for further information.

All dose modifications/adjustments must be clearly documented in the patient's source notes and IP administration eCRF.

6.13 Compliance

Patients will be required to return all bottles of CT7001[redacted] as well as their completed patient diary at the beginning of each cycle for drug accountability. Drug accountability will be performed on Day 1 of every cycle prior to dispensing drug supply for the next cycle. The

number of remaining capsules will be documented and recorded. Patients who miss doses will be counselled on the importance of compliance.

6.14 Drug Storage of Investigational Products (IPs) and Accountability

Storage conditions as stated in the IB are superseded by the instructions on the label. Investigators and site staff are reminded to continuously monitor room storage temperatures and ensure that thermometers are working correctly as required for proper storage of the IPs. Temperature excursions must be reported immediately to the sponsor and documented. Once a deviation is identified, the IPs MUST be quarantined and not used until the sponsor provides documentation of permission to use the IP.

At the end of the trial or at the close-out of the site, any unused IPs will be destroyed. If the destruction occurs at the trial site, the investigator must ensure that the materials are destroyed in compliance with applicable environmental regulations, institutional policy, and any special instructions provided by the sponsor. Destruction must be adequately documented. Alternatively, IP maybe shipped to a local depot for destruction.

To ensure adequate records, CT7001[redacted] capsules will be accounted for as instructed by the sponsor. Patients are required to return previously dispensed containers as well as their completed patient diary to the clinic at each visit for accountability purposes even if they will not be issued with new medication at that visit.

6.15 Fulvestrant Monotherapy

Under specific circumstances the Investigator may apply to the Sponsor to keep a patient on-study and receiving monotherapy Fulvestrant. The following conditions will need to be met:

- Patient should meet study discontinuation criteria due to tolerability of CT7001
- Patient is continuing to receive clinical benefit from Fulvestrant
- Fulvestrant is not reimbursed locally
- Discontinuation of Fulvestrant is considered unethical

Following Sponsor approval, patients will remain on-study until such time they meet discontinuation criteria (Section 7.6.1). During this Fulvestrant monotherapy period a reduced schedule of events will be followed (Table 6).

6.16 Supply of Investigational Product Post Study

Under specific circumstances, a patient may meet study discontinuation criteria (Section 7.6.1) but continue to receive clinical benefit from the Investigational Product. For the specific instance of patients receiving ongoing clinical benefit at the time of the primary endpoint analysis, then at the discretion of the Investigator, with approval of the Sponsor, and in compliance with national regulations the Investigator may initiate completion of study participation and apply for Managed Access of Investigational Product. It is anticipated that for this to apply the final analysis would have demonstrated a statistically significant clinically relevant benefit.

6.17 Concomitant Medications

If medically reasonable and feasible, subjects taking regular medication (with the exception of, strong inhibitors or inducers of CYP3A4, CYP2C19, CYP2D6, or P-glycoprotein (PGP) (see Appendix B) should be maintained on it throughout the study/module.

Patients must be instructed not to take any new medications (over-the-counter or other products) during the study without prior consultation with the investigator. Medications that are considered necessary for the subject's safety and well-being may be given at the discretion of the Investigator.

Any medications including herbal supplements, vitamins or medicines taken by the patient from 28 days prior to the start of study treatment and up to 28 days following the last dose of investigational product and the reason for their administration, must be recorded on the eCRF.

Routine postoperative care, such as dressing changes, suture removal, drain removal, or venous access (central or peripheral), does not need to be recorded. Anaesthetics used for any surgical procedures performed during the patient's participation in the study can be recorded as "unspecified anaesthesia" on the concomitant treatment records; it is not necessary to list the specific anaesthetics.

Appropriate palliative and supportive care for cancer-related symptoms must be offered to all patients in this study.

6.17.1 Prohibited Medications

The following treatments are prohibited throughout the duration of the active treatment phase:

- **Anti-cancer agents**: No additional investigational or commercial anti-cancer agents such as chemotherapy, immunotherapy, targeted therapy, biological response modifiers or endocrine therapy will be permitted during the active treatment phase. In general, any drugs containing "for the treatment of breast cancer" on the product insert are not permitted on study.
    - Please note that this does not apply to the LHRH agonists. In pre- or peri-menopausal women, treatment with an LHRH agonist not only is permitted but is mandatory in this study, as described in Sections 4 and 5.
- **No investigational product other than CT7001, [redacted] and fulvestrant**.
- **Blood transfusions** are not allowed within 14 days before the first IP dose.
- **Live (where the vaccine is of a 'live-attenuated' virus/bacteria rather than a viral/bacterial-vector approach) virus or bacterial vaccines** (e.g., yellow fever, measles, influenza, rubella, mumps, typhoid, mycobacterium tuberculosis [BCG], Yersinia pestis [EV] vaccines).
    - An increased risk of infection by the administration of these vaccines has been observed with conventional chemotherapy and the effects with CT7001 are unknown.

      - Live vaccines must not be administered until 3 months after the last dose of IP.
      - If the live vaccine induces B-cell depletion then they should not be administered until 6 months after the last dose of IP (Rubin et al., 2014).
   - Administration of the live flu vaccine is allowed if given at least 28 days prior to start of screening.
   - The administration of killed vaccines (e.g., cholera, bubonic plague, non-live influenza, polio, hepatitis A, and rabies vaccine) is allowed.

6.17.2 Medications Not Recommended

The following treatments are not recommended throughout the duration of the active treatment phase. Alternative therapies should be considered whenever possible. If the investigators deemed usage of the following treatments necessary, consultation and agreement with the sponsor is required prior to initiation of treatment.

- Medications, herbal supplements, and foods that are strong inducers or inhibitors of CYP3A4, CYP2C19, CYP2D6 or PGP (see Appendix B for a listing) should be avoided from 14 days before the first dose of CT7001[redacted] until 28 days after the last dose of CT7001[redacted].
   - **Note:** St. John's Wort should be avoided from 21 days before the first dose of CT7001[redacted].
   - Aprepitant (Emend®) is a substrate, moderate inhibitor and inducer of CYP3A4 and an inducer of CYP2C19. Therefore, aprepitant should not be used in this study for the treatment of nausea and vomiting.
   - If the Investigator feels that concomitant administration of such medications, herbal supplements or foods is necessary based upon medical judgment, such products may be administered with caution following discussion between the Investigator and the Carrick Therapeutics Physician or clinical research organisation (CRO) medical monitor.
      - CT7001 is an investigational drug for which no *in vivo* data on drug interactions are currently available.
      - Patients taking concomitant medications whose disposition is dependent upon CYP3A4 and which have a narrow therapeutic index should be closely monitored for signs of changed tolerability.
      - CT7001 shows a weak potential to inhibit CYP2D6 and 2C19, which should be considered when co-prescribing 2D6 and 2C19 substrates.
- CT7001 exhibits pH-dependent solubility. As such there is a risk that agents that increase gastric pH (such as PPIs (proton pump inhibitors), H2 antagonists) may affect the bioavailability of CT7001 and should be avoided in the study if possible. However, if clinically required, they may be prescribed. Such patients should be monitored for signs of changed CT7001[redacted] effects.

- Chronic immunosuppressive therapies should be avoided, including systemic corticosteroids.
    - Steroids given for physiological replacement, as anti-emetics or inhaled as well as short course of oral or topical steroids given for allergic reactions or asthma flares are allowed.
- Drugs known to predispose to Torsades de Pointes should be avoided during the active treatment phase. Refer to Appendix A for a list of such drugs.
- The use of any natural or herbal products or other 'folk remedies' should be discouraged. Cannabinoids / CBD oil are discouraged, if these are used they should be clearly captured in the eCRF.

6.17.3 Recommended and Permitted Treatments

The following treatments are permitted throughout the duration of the active treatment phase, with all medications and treatments to be recorded in the eCRF:

- As described in Sections 4 and 5, pre- and peri-menopausal patients must receive an LHRH agonist, given and dosed as per locally approved label and standard institutional practice. In such patients, treatment with the LHRH agonist must have started a minimum of 4 weeks before assignment to therapy in the present study. LHRH agonists are considered an auxiliary medicinal product in this study.
- Continuation of therapies for pre-existing medical conditions.
    - This includes bisphosphonates and/or receptor activator of nuclear factor kappa-B ligand (RANKL) inhibitors for the treatment of osteoporosis or management of existing bone metastases, provided patients have been receiving them at a stable dose for at least 2 weeks prior to first dose of CT7001[redacted].
        - Please note that the need to initiate, or increase the dose of, these therapies during the study will be considered as indicative of disease progression unless disease progression can be fully ruled out and the exact alternative reason for the use of these therapies is clearly documented in the subject's source documentation.
- Treatments of medical and/or surgical complications.
- All study patients must be offered **best supportive care** as per standard institutional practice and/or most recent guidelines by organizations such as ASCO, the National Comprehensive Cancer Network (NCCN) in the US and/or the European Society of Medical Oncology (ESMO). The below, protocol specific, management of nausea and/or vomiting may be amended by the Safety Review Committee based on emerging data. Any change to the NVD management instructions will not be considered a substantial amendment to the protocol.
    - **Anti-emetic medication**, specifically a 5-HT3 antagonist in the first instance, must be given prophylactically for nausea (N) and/or vomiting (V) prior to the first dose and during Cycle 1 (Day 1 to 15) in accordance with the current DMC

NVD Management Instructions (see Appendix H). Patients must be given an adequate supply of an anti-emetic to take home with them. Thereafter, withdrawal of the anti-emetic may be commenced at the Investigator's discretion.

- **Haematopoietic growth factors**:
  - Primary prophylactic use of granulocyte colony stimulating factor (G-CSF) or granulocyte macrophage colony stimulating factor (GM-CSF) is not permitted but non-pegylated G-CSF may be used to treat treatment-emergent neutropenia when clinically indicated as per standard institutional practice and/or most recent ASCO/NCCN/ESMO guidelines.
    - If neutropenic complications occur in a cycle, secondary prophylaxis may be given at the discretion of the investigator, but only if dose reduction or delay are not considered to be a reasonable alternative.
  - Erythropoietin may be used at the investigator's discretion for the supportive treatment of anaemia.
- **Red blood cell transfusions** may be given as clinically indicated for the treatment of anaemia but should be clearly noted as concurrent medications.
- **Platelet transfusions** may be given as clinically indicated for the treatment of bleeding associated with thrombocytopenia but should be clearly noted as concurrent medications.
- **Diarrhoea**: Patients must have available an adequate supply of an anti-diarrhoeal medication to take home with them. In the event of diarrhoea, supportive measures must be initiated promptly. These include the following:
  - At the first sign of loose stools, the patient must initiate anti-diarrheal therapy (e.g., loperamide) and notify the investigator/site for further instructions and appropriate follow-up.
  - Patients should also be encouraged to drink plenty of fluids (e.g., 8 to 10 glasses of clear liquids per day).
  - Site personnel should assess response within 24 hours.
  - If diarrhoea does not resolve with anti-diarrheal therapy within 24 hours to at least Grade 1, CT7001 should be suspended until diarrhoea is resolved to at least Grade 1.
  - In case of Grade ≥3 diarrhoea, CT7001 should be interrupted (with a cycle) or delayed (at start of next cycle). See also Sections 6.12.2.2 and 6.12.2.3.

        - In severe cases of diarrhoea, the measuring of neutrophil counts and body temperature and treatment with antidiarrheal agents should be considered.
            - Antidiarrheal agents should be deferred if blood or mucus is present in the stool or if diarrhoea is accompanied by fever. In these circumstances, appropriate diagnostic microbiologic specimens should be obtained to exclude an infectious aetiology.
        - If diarrhoea is severe (requiring intravenous (IV) rehydration) and/or associated with fever or severe neutropenia, broad-spectrum antibiotics such as fluoroquinolones should be prescribed.
        - Patients with severe diarrhoea or any grade of diarrhoea associated with severe nausea or vomiting should be carefully monitored and if needed given intravenous fluid (IV hydration) and electrolyte replacement.
    - **Medication and other measures for pain control** should follow standard institutional practice and/or ASCO/NCCN/ESMO guidelines.
    - **Other Medications,** in accordance with local standard of care, will be permitted unless specified otherwise (see prohibited medicines and medicines not recommended).

6.18 Contraception

Women of childbearing potential (for definition of no childbearing potential see Section 4.1) must practice effective contraception during treatment and for 2 years after the last dose of study drug and/or fulvestrant. This includes:

- Abstinence if consistently employed.
- Sex only with person of the same sex or with vasectomised partner.
- Intrauterine device (IUD), or barrier method (e.g., condom, diaphragm)
- **Note** that contraceptives that are prone to drug-drug interactions may not be effective because of a potential CYP3A4 interaction with CT7001.

6.19 Concomitant Radiotherapy or Surgery

Concurrent radiotherapy (except palliative radiotherapy as specified below) or cancer-related surgery are prohibited throughout the duration of the active treatment phase of the study. Patients requiring these procedures will be discontinued from the active treatment phase and will enter the follow-up phase.

Palliative radiotherapy is permitted for the treatment of painful bony lesions provided that the lesions were known to be present at the time of study entry and the investigator clearly documents that the need for palliative radiotherapy is not indicative of disease progression.

In view of the current lack of data about the interaction of CT7001 with radiotherapy, study treatment with CT7001[redacted] should be interrupted during palliative radiotherapy, stopping at least 1 day before and resuming treatment no earlier than 1 week after.

For patients with bone involvement, it is suggested to institute palliative radiotherapy before study initiation if possible and clinically appropriate (e.g., lesions at risk for spontaneous micro-fractures or painful lesions).

Palliative radiotherapy during the active treatment phase will be considered alternative cancer therapy and will result in censoring of the PFS endpoint. The dates on which palliative radiotherapy is administered should be recorded on the appropriate eCRFs.

Caution is advised on theoretical grounds for any surgical procedures during the study. The appropriate interval of time between surgery and CT7001 required to minimize the risk of impaired wound healing and bleeding has not been determined. Based on the available pharmacokinetic data, stopping of CT7001[redacted] is recommended at least 7 days prior to elective surgery. Postoperatively, the decision to reinitiate CT7001[redacted] treatment should be based on a clinical assessment of satisfactory wound healing and recovery from surgery.

7 STUDY PROCEDURES

Prior to undergoing any study-specific procedures (with the exception of certain imaging assessments, if meeting the criteria defined in Section 7.1), patients must read and sign the consent form(s). All study procedures and their timing are detailed in the Schedule of Events (Section 18, Table 6). All data obtained for these assessments must be supported in the patients' source documentation. For the purposes of this trial, a cycle is defined as 28 days. A cycle could be longer than 28 days if persistent toxicity delays the initiation of the subsequent cycle.

7.1 Screening

Voluntary, written, dated, and signed informed consent MUST be obtained before any study specific procedures are performed (with the exception of certain imaging assessments, if meeting the criteria defined in this section).

Radiographic tumour assessments that were performed before the signing of the informed consent form as routine procedures (but within 28 days prior to allocation to study treatment) do not need to be repeated and may be used as baseline assessments if:

- The tests were performed per the method requirements described in the Schedule of Events (Section 18, Table 6 and Section 8.1).
- Appropriate documentation indicating that these radiographic tumour assessments were performed as standard of care is available in the patient's source notes.

Bone scans performed as routine procedures within 12 weeks prior to allocation to study treatment are also accepted as baseline assessment if they meet the two requirements listed above.

Details on screening procedures are provided in the Schedule of Events (Section 18, Table 6).

7.1.1 Screen Failure

Patients who completed the informed consent process but do NOT meet all eligibility criteria and therefore are NOT enrolled in the study will be considered as screen failures.

Subjects who fail screening may be re-screened only on approval of the SRC and Sponsor. Any subject re-screened will need to provide new informed consent and will be allocated a new subject number.

7.2 Active Treatment Phase

For details on procedures during the active treatment phase, see the Schedule of Events (Section 18, Table 6). In the event the start of a new cycle is delayed, procedures required on Day 1 of that cycle will be performed when study treatment is resumed. Day 1 procedures (i.e., physical examination, ECOG performance status, ECG, blood chemistry and haematology) that were performed prior to knowing the need to delay the start of the cycle do not need to be repeated if:

- Not required to determine whether study drug may be resumed and
- Performed within 7 days prior to restart of study therapy.

[redacted]

7.4 End of Treatment Visit

The end of treatment visit will be performed 28 days from last dose of IP and prior to the initiation of any new anti-cancer therapy, whichever occurs first. In the event treatment was discontinued due to death or documented disease progression (reference section 8.1.2), the end of treatment visit represents the final visit.

For details on procedures to be performed at the End of Treatment visit, see the Schedule of Events (Section 18, Table 6).

7.5 Follow-up Phase

Unless study treatment was discontinued due to death or documented disease progression, patients will be followed for progression-free survival until documented disease progression, death, start of post-study cancer therapy (Part A only), lost to follow-up or Module LPLV, whichever occurs first. For details on procedures to be performed see the Schedule of Events (Section 18, Table 6).

7.6 Patient Withdrawal

The term "interruption" refers to a patient stopping the investigational product during the course of the study, but then re-starting it at a later time in the study. The reason for dosing interruption will be collected on the appropriate eCRF.

The term "discontinuation" refers to a patient's withdrawal from the study, which may occur during the active treatment phase or post-treatment. The reason for discontinuation must be collected on the appropriate eCRF.

7.6.1 Active Treatment Phase Discontinuation

Patients must be withdrawn from the active treatment phase in case of:

- Disease progression as per RECIST v.1.1 (Appendix C) (reference section 8.1.2).
- Symptomatic deterioration (i.e., global deterioration of health status or requiring discontinuation of treatment without objective evidence of disease progression as per RECIST v.1.1).
- Need for additional anti-cancer therapy not specified in the protocol.
- Unacceptable toxicities.
- Investigator conclusion that it is in the patient’s best interest to discontinue therapy (e.g., poor compliance with either protocol monitoring or with taking the study medications, etc).
- Lost to follow-up.
    - If a patient does not return for a scheduled visit, every effort should be made to contact the patient.
    - If 3 attempts to contact the patient were unsuccessful, one of which is by registered letter, the patient should be considered “lost to follow-up”.
    - Steps taken to contact the patient (e.g., dates of telephone calls, registered letters, etc) must be clearly documented in the source documents.
- Patient choice to withdraw from treatment (follow-up permitted by patient).
- Withdrawal of patient consent (cessation of follow-up).
- Death.

[redacted]

Patients who discontinue from the active treatment phase must have end of treatment/withdrawal evaluations performed as soon as possible but no later than 28 days from the last dose of investigational product and prior to initiation of any new anti-cancer therapy. Data to be collected at the end of study treatment/withdrawal visit are described in the Schedule of Events (Section 18, Table 6).

If a patient opts to discontinue from the active treatment phase as a result of an unacceptable adverse drug reaction, "withdrawal of consent" should not be recorded as the reason for discontinuation. Instead, the reason for discontinuation of active treatment phase must be recorded as "unacceptable toxicity" on the AE eCRF leading to the patient's withdrawal of consent.

7.6.2 Post-Treatment Study Discontinuation

After discontinuation of study therapy, patients may withdraw from the study at any time at their own request or they may be withdrawn at any time at the discretion of the investigator or sponsor for safety or behavioural reasons, or the inability of the patient to comply with the protocol required schedule of study visits or procedures at a given study site.

Patients will be withdrawn from study in the case of:

- Withdrawal of consent (i.e., refuses tumour assessments).
- Lost to follow-up (see Section 7.6.1 for activities required before declaring a patient as lost to follow-up).
- Module 2 Last Patient Last Visit achieved
- Death.

In any circumstance, every effort should be made to document patient outcome, if possible. The investigator should inquire about the reason for withdrawal, request the patient to return all unused investigational product(s), request the patient to return for a final visit, if applicable, and follow-up with the patient regarding any unresolved AEs.

Data to be collected for the end of study treatment/withdrawal are described in the Schedule of Events (Section 18, Table 6).

If a patient withdraws from the study and withdraws consent for disclosure of future information, no further evaluations should be performed, and no additional data should be collected. The sponsor may retain and continue to use any data collected before such withdrawal of consent.

7.7 Study Completion

Study completion is defined as last patient last visit i.e.:

- Objective disease progression
- Symptomatic deterioration
- Unacceptable Toxicity
- Death
- Withdrawal of consent
- [redacted]

8 STUDY ASSESSMENTS

The study procedures are described in the Schedule of Events (Section 18, Table 6) and its footnotes. Every effort should be made to ensure that the required tests and procedures are completed as described. However, it is anticipated that from time to time there may be circumstances, outside of the control of the investigator, that may make it unfeasible to perform the test at all or on schedule. In these cases, the investigator will take all steps necessary to ensure the safety and wellbeing of the patient. When a protocol-required test cannot be performed the investigator will document the reason for this and any corrective and preventive actions which he/she has taken to ensure that normal processes are adhered to as soon as possible. The study team will be informed of such incidents in a timely fashion.

8.1 Tumour Assessments

Tumour assessments should be performed as scheduled, regardless of treatment interruptions or cycle delays.

CT scans, including brain CT scan if applicable, should be performed with an IV contrast agent unless contraindicated for medical reasons.

If IV contrast is medically contraindicated, the imaging method to be used to follow the disease (i.e., CT without contrast or MRI) should be the method which best evaluates the particular disease location. This decision should be made by the investigator in conjunction with the local radiologist.

For abdomen, pelvis and the brain, MRI can be substituted for CT. For the chest, however, MRI may not be substituted for CT even if IV contrast is contraindicated. In such case CT without contrast must be used.

If MRI is used to follow-up bone lesion(s) it must be performed a few days before any treatment that may affect bone-marrow cellularity (e.g., G-CSF).

The same imaging method(s) as used at screening must be used for subsequent tumour assessments throughout the study.

8.1.1 Screening/Baseline

Screening tumour assessment by CT, MRI and clinical examination must be carried out within 28 days before allocation to study therapy. For bone scans which were performed as routine procedure, a period of 12 weeks prior to allocation to study therapy is accepted provided the requirements described in Section 7.1 are met.

Disease assessment at baseline will include:

- CT scan of the chest.
- CT scan or MRI of the abdomen and pelvis.
- CT scan or MRI of any other sites of disease as clinically indicated.
- Clinical assessment of superficial disease which will include photographs of all superficial tumour lesions.
- Radionuclide bone scan to detect sites of bone metastasis.
    - Any suspicious abnormalities (i.e., hotspots) identified on the bone scans at baseline must be confirmed by CT scan with bone windows or MRI.
- All lesion measurements must be recorded in the eCRF.
- Baseline brain CT or MRI are only required in case clinical signs or symptoms suggest the presence of metastatic brain disease.

The use of plain-film X-rays or positron emission tomography (PET) is not permitted for purpose of recording and following tumour lesions.

For patients with effusions such as pleural effusion or ascites, cases having cytological proof of malignancy should be recorded as non-target lesions on the tumour assessment eCRFs. Effusions that have not been evaluated using cytology or were found to be non-malignant should not be recorded on the non-target lesion eCRF.

8.1.2 Post-Baseline

During the study treatment period and follow-up, the same method must be used for assessment of a given region (e.g. chest, abdomen, pelvis) as was used at baseline for tumour assessment in that location.

Post-baseline tumour assessments by CT or MRI will be performed every 8 weeks (± 7 days) for the first year and then every 12 weeks (± 7 days) in subsequent years (calculated from the date of Cycle 1 Day 1).

Bone scans (as applicable) will be repeated every 16 weeks (±7 days) from Cycle 1 Day 1.

If measurable/evaluable bone lesions were identified at baseline by CT scan or MRI, CT scan or MRI, will be repeated every 8 weeks (± 7 days), using the same modality used to confirm the bone lesions at baseline, for the first year, and then every 12 weeks (±7 days) in subsequent years (calculated from the date of Cycle 1 Day 1).

Additional unscheduled tumour assessments may be performed at any time as clinically indicated.

Tumour assessments will continue until radiographically and/or clinically (i.e., for photographed or palpable lesions) documented PD as per RECISTv.1.1* (Appendix C), death, discontinuation of patient from overall study participation (e.g., patient's request, lost to follow-up), initiation of new anticancer therapy (Parts A [redacted] or Module 2 LPLV is met, whichever occurs first. [redacted]

*At the time of first documented RECIST disease progression, if the investigator and patient consider the patient is still benefitting from study treatment, dosing may continue until the disease progression is confirmed at the next scheduled imaging evaluation. If at the subsequent scan the progression is confirmed study treatment should end, if not confirmed the patient may remain on study treatment. Every effort should be made to perform a last tumour assessment before starting a new anticancer therapy.

Patients who discontinue CT7001 but remain on study (Fulvestrant monotherapy) should have a last tumour assessment prior to their next administration of Fulvestrant where possible.

Please note: Imaging assessments must be scheduled using Cycle 1 Day 1 as the reference date and NOT based on the date of the previous imaging time-point. Imaging assessment delay to conform to treatment delay is not permitted.

Post-baseline tumour assessments will include:

- CT scan of the chest.
- CT scan or MRI of the abdomen and pelvis.
- CT scan or MRI of other sites of disease identified at baseline.
- Clinical assessment of sites of superficial disease identified at baseline.
    - Clinical assessment of superficial disease must coincide with the imaging studies and will include photographs of all superficial metastatic lesions.
- If measurable/evaluable bone lesions were identified at baseline by CT scan or MRI:
    - CT scan with bone window or MRI of those lesions, using the same modality used to confirm the bone lesions at baseline.
    - Areas that have received palliative radiotherapy on study cannot be used to assess response to study treatment.
- If no measurable/evaluable bone lesions were confirmed at baseline by CT scan or MRI but the baseline bone scan showed hot spots:
    - Bone scans every 16 weeks (± 7 days) from the date of Cycle 1 Day 1 and to confirm complete response.
- If no bone lesions were identified at baseline:

  - Bone scans should be performed as clinically indicated (i.e., patient describes new or worsening bone pain, or has increasing alkaline phosphatase level or other signs and symptoms suspicious of bone metastases) but are required to confirm complete response.

- New abnormalities found on bone scans must be confirmed by CT scan with bone window or MRI.

- Repeat brain scans are required only if metastases are clinically suspected.

Objective tumour response will be measured using RECIST Version 1.1, (see Appendix C). All measurements should be recorded in metric notation using a ruler or calliper.

8.1.3 Independent Review of Disease Assessments

For Parts A [redacted] anti-tumour activity will be assessed locally by the Investigator. The Sponsor reserves the right to request an anonymised independent review of subject scan data if required.

[redacted]

Further information on images and media to be forwarded for independent review, correct procedures for the coding/blinding of the patient's name/identity and the return of the source data/documents to the site is provided in the Imaging Site Manual.

8.2 Safety Assessments

Safety assessment will consist of monitoring of all AEs, including serious adverse events (SAEs), regular monitoring of haematology, serum chemistry, triplicate 12-lead ECGs, physical examinations, vital signs and ECOG performance status.

Adverse event assessment will include type, incidence, severity (graded by the CTCAE, Version 5.0, see Section 9.6 Severity Assessment), timing, seriousness, and relatedness. Baseline tumour-related signs and symptoms will be recorded at the Cycle 1 Day 1 visit and then reported as adverse events during the trial if they worsen in severity or increase in frequency.

All protocol required safety assessments should be performed before administration of fulvestrant.

8.2.1 Laboratory Safety Assessments

Laboratory tests will include full blood counts, standard serum chemistry and urinalysis.

Full blood counts include: red blood cell count, haematocrit, mean cell volume, reticulocyte count (absolute particle count), white blood cell count with differential (absolute and percent counts of neutrophils, lymphocytes, monocytes, basophils, eosinophils) and platelet count. [redacted]

Serum chemistry includes: HbA1c, ALT, AST, gamma glutamyl transferase, alkaline phosphatase, bilirubin (total), creatine kinase, total protein, albumin, creatinine, urea nitrogen or urea, calcium (total), glucose, sodium, potassium, magnesium, chloride and phosphate. For patients entering the study with a medical history of diabetes, a fasted glucose test will be required pre-dose at C1D1 and C1D15 (mandatory) and at the remaining cycles at the discretion of the investigator.

Urinalysis includes: blood, glucose and protein.

All laboratory tests will be performed during screening within 28 days before allocation to study therapy, on Days 1 and 15 in the first cycle (day 15 only in Part A [redacted]), on Day 1 in subsequent cycles, and at the end of treatment and end of study visits. In Part A, an additional assessment is required on Day 15 of the second cycle. Please also refer to the Schedule of Events (Section 18, Table 6)).

Laboratory tests do not need to be repeated on Day 1 of Cycle 1 if performed within 7 days prior to allocation to study therapy.

All results will be entered in the eCRF, with Système International (SI) units as standard system of measurements. However, the eCRF will allow reporting of a subset of laboratory tests in conventional units if participating study sites consider this helpful.

Additional blood tests may be performed at the investigator's discretion as clinically indicated for purpose of planning treatment administration, dose modification, or following adverse events. If specific tumour markers are followed locally, these will be captured in the eCRF.

8.2.2 Electrocardiogram (ECG)

ECGs will be performed using a12-lead tracing. ECG measurements will include heart rate, PR interval, QRS complex, QT interval, and QTcF. ECG interval readings by the ECG recorder’s algorithm will be read and interpreted at the investigational site for eligibility determination and patient safety monitoring and documentation stored in the source documents.

Triplicate 12-lead ECGs will be performed during screening within four weeks before allocation to study therapy, approximately every four weeks in all cycles, and at the end of treatment and end of study visits. Triplicate ECGs do not need to be repeated on Day 1 of Cycle 1 if performed within 7 days prior to allocation to study therapy. Triplicate ECGs are not required during Fulvestrant monotherapy.

Additional ECGs may be performed as clinically indicated at any time.

Triplicate 12-lead ECGs will be performed 3-5 minutes apart, after the patient had been resting for at least 10 minutes.

If at any time during treatment the mean QTc is prolonged ≥501 msec on at least two separate ECGs (i.e., CTCAE ≥ Grade 3), then the ECGs should be re-evaluated by a qualified person at the site for confirmation as soon as the finding is made, including verification that the machine reading is accurate. If manual reading confirms a QTc of ≥ 501 msec, an immediate search for reversible causes should be performed (including electrolyte abnormalities, hypoxia and concomitant medications for drugs with the potential to prolong the QTc interval). In addition,

repeat ECGs should be performed hourly for at least 3 hours or until the QTc interval falls below 501 msec, whichever occurs first.

8.2.3 Other Safety Assessments

A full physical examination and assessment of vital signs and performance status is required at screening, on Day 1 in all cycles, and at the end of treatment and end of study visits. Examination and assessments do not need to be repeated on Day 1 of Cycle 1 if performed within 7 days prior to allocation to study therapy. In Part A, an additional examination and assessment is required on Day 15 of the first two cycles.

A full physical examination includes examination of all major body systems, height (at screening only) and weight. A physical examination is not required during Fulvestrant monotherapy.

Vital signs include supine blood pressure, pulse rate, temperature and respiratory rate.

Performance status will be assessed according to The Eastern Cooperative Oncology Group (ECOG) performance status scale (see Appendix F).

8.3 Pharmacokinetic Assessments

Blood samples for CT7001[redacted]trough concentrations will be collected before dosing on Day 1 and Day 15 of Cycle 1, Day 1 in Cycles 2 and 3, Day 1 in every other odd subsequent Cycle and at the end of treatment visit (see also the Schedule of Events, Section 18, Table 6).

Blood samples for fulvestrant trough concentrations will be collected before injection on Day 1 and Day 15 of Cycle 1, Day 1 in Cycles 2 and 3, Day 1 in every other odd subsequent Cycle and at the end of treatment visit. PK samples are not required during Fulvestrant monotherapy.

All efforts should be made to obtain the PK samples at the scheduled nominal times and within the time window relative to dosing. Additional blood samples may be requested from patients experiencing unexpected or serious adverse events, or adverse events that lead to discontinuation.

The exact time of the sample collection and the most recent dosing time will be recorded on the eCRF. The date of missing doses should also be recorded in the eCRF.

As part of understanding the PK behaviour and profile of CT7001, part of the blood samples may be used for metabolite identification. These data will be used for internal exploratory purposes and will not be included in the Clinical Study Report.

The concentration of CT7001 and metabolites, if applicable, in PK samples will be quantified by liquid chromatography with tandem mass spectrometry.

Refer to the Laboratory Manual for detailed collection, processing and shipping procedures.

8.4 Assessment of CYP and drug transporter genes

CYP2D6 is the main P450 isozyme involved in Phase I metabolism of CT7001 but other members of the CYP family also play a role. Drug transporters may also affect the bioavailability of CT7001. Therefore, a blood sample will be collected before dosing on Day

1 to analyse polymorphisms and copy number variations of CYP and drug transporter genes. The racial and ethnic distribution among study patients will (co-)inform which alleles to be genotyped.

8.5 Biomarker Assessments

Unless prohibited by local laws and regulations, biological samples collected in this study will become the property of Carrick Therapeutics and may be used for future research conducted by or on behalf of Carrick or its affiliates, partners, or collaborators. No identifiable personal information will be associated with these blood samples. Any remaining samples will be destroyed no later than 15 years after the end of the study.

General collection and processing instructions are provided in the Laboratory Manual. Collection times must be entered in the eCRF. Please also refer to the Schedule of Events (Section 18, Table 6) for the various types of procedures and tests, and their sampling schedule.

8.5.1 ER, PgR and HER2

Assessment of expression of ER, PgR and of HER2 status in breast carcinomas from patients in screening will be based on results from local pathology laboratories. No independent central review is intended.

Assessment of ER, PgR and HER2 by local laboratories must be consistent with the criteria in the most recent versions of ASCO/CAP guidelines for the hormone receptors and HER2, respectively (Hammond et al., 2010; Wolff et al., 2018).

- ER- and PgR-positivity is defined as ≥1% positive stained cells (ASCO/CAP guidelines, Hammond et al., 2010).
- HER2-negativity is defined as immunohistochemistry score 0/1+ or negative by in situ hybridization (FISH/CISH/SISH) defined as a HER2/CEP17 ratio < 2 or for single probe assessment a HER2 copy number < 4 (ASCO/CAP guidelines, Wolff et al., 2018).

Assessment of ER, PgR and HER2 status should be based on the most recent tumour biopsy utilizing an assay consistent with local standards. In case no tumour biopsy was performed after initial resection of the primary tumour, the original tumour tissue serves as basis for assessment of ER/PgR and HER2 status.

8.5.2 Exploratory Biomarkers

Various tumour-derived materials will be collected to further explore the effect of mutations and expression in certain genes, RNAs and proteins on sensitivity and/or resistance to CT7001. The tumour-derived materials include formalin-fixed paraffin embedded (FFPE) and/or fresh frozen tumour tissue obtained through tumour biopsy and circulating tumour DNA (ctDNA). A panel of genes, proteins and RNAs relevant to the cell cycle, drug target engagement and/or sensitivity/resistance will be analysed, such as phosphorylated CDK1, RNA polymerase II, MED1, Rb proteins, c-Myc, MCL-1, p53, CDK7, ER, ESR1, AR and PIK3CA.

Except for ER, PgR and HER2 status as part of screening, all biomarker analyses and related procedures are considered optional. Patient consent will be requested/obtained separately for peripheral blood samples versus tumour biopsies, and for retained samples for pharmacogenomics.

Patients who elect not to allow any or all optional procedures may still participate in the clinical trial.

In the event patient consent was obtained, peripheral blood samples for various biomarker assessments will be collected at Screening, on Day 1 of Cycles 1, 2 and each 'odd numbered' treatment cycle and at the time of documented disease progression. Collection times will be entered in the eCRF. Biomarker samples are not required during Fulvestrant monotherapy.

8.5.3 Tumour Biopsies

For subjects with readily accessible lesions who have provided consent, tumour biopsies are encouraged to be obtained at baseline (within 10 days before allocation to study therapy), on Day 15 ± 7 days in Cycle 2 and within two weeks after documentation of objective disease progression. Collection times will be entered in the eCRF.

An accessible lesion is defined as a tumour lesion that is amenable to repeat biopsy, in the same lesion, unless clinically contraindicated or the subject has withdrawn consent. Failure to obtain a sufficient tumour sample after making best efforts to biopsy the tumour will not be considered a protocol deviation.

Where possible, two biopsy cores should be collected per scheduled timepoint. In the obtained biopsy material, first priority will be given to FFPE for immunohistochemistry (CDK7, pPolII, c-Myc, pMED1, pCDK1 and Ki67/Caspase) and mRNA expression analysis. In case of a second biopsy core being taken, it should be stored in RNA later for ChIP-Sequence analysis. Failure to collect a second biopsy core <u>will not</u> be considered a protocol deviation.

8.5.4 Archival Tumour Tissue

An archival FFPE tumour tissue sample, if available, will be requested for each subject. Even if fresh biopsy samples can be collected, retrieval of the archival diagnostic tumour material is highly important in order to provide data on how the tumour has evolved since diagnosis. Archival samples from either primary or metastatic tumour will be accepted, but tissue from the primary tumour is preferred. For a subject who has archival tissue samples from multiple time points, tissue from the most recent biopsy is preferred.

Tumour tissue blocks are preferred, but freshly prepared unstained slides (minimum 10, preferably 20) with 5-micron sections from the archival tumour block are accepted if tumour blocks cannot be submitted.

8.5.5 Retained Samples for Future Exploratory Research

CT7001 is early in its clinical development. The science of CDK7 is rapidly evolving and so are the data of CT7001. As a result, by the end of the study and/or in the future new biomarker hypotheses might be important to investigate. Provided specific written informed consent was obtained from the patient and not prohibited by local laws and regulations, blood samples for

potential future exploratory research will be collected before dosing on Day 1 of Cycles 1, 2 and 3, Day 1 in every other ‘odd numbered’ treatment Cycle and within 2 weeks after disease progression. Biomarker samples are not required during Fulvestrant monotherapy.

8.5.6 Retained Sample for Potential Future Pharmacogenomic Research

Genotyping of CYP and drug transporter genes (Section 8.4) is a mandatory element of this study. Unless prohibited by local laws and regulations, patients will be asked to indicate on the consent form whether they will allow retention of remaining material from the blood sample collected and prepared for that analysis and potential use for additional future pharmacogenomic research, which may include but is not limited to:

- Investigations of the disease under study in the clinical trial, and related conditions.
- Use of sample as control. This includes use in case-control studies of diseases for which Carrick is researching drug therapies; use in characterizing the natural variation amongst people in genes, RNA, proteins, and metabolites; and use in developing new technologies related to pharmacogenomics.

Details on the long-term storage of these samples are provided in the Laboratory Manual.

9 ADVERSE EVENT REPORTING

All observed adverse events (AEs) regardless of suspected causal relationship to the investigational product will be reported as described in the following sections. For all AEs, the investigator must pursue and obtain information adequate both to determine the outcome of the AE and to assess whether it meets the criteria for classification as an adverse event of special interest (AESI) or SAE requiring immediate notification to the sponsor or its designated representative (’the sponsor’).

For all AEs, sufficient information should be obtained by the investigator to determine the causality of the AE. The investigator is required to assess causality.

Follow-up by the investigator may be required until the event or its sequelae resolve or stabilize at a level acceptable to the investigator, and the sponsor concurs with that assessment.

As part of ongoing safety reviews conducted by the sponsor, any non-serious AE that is determined by the sponsor to be serious will be reported by the sponsor as an SAE. To assist in the determination of case seriousness further information may be requested from the investigator to provide clarity and understanding of the event in the context of the clinical study.

All AEs spontaneously reported by the subject, reported in response to an open question from the study personnel (e.g., ‘Have you had any health problems since the previous visit/you were last asked?’), or revealed by observation will be recorded in the eCRF.

When recording AEs, the diagnosis is preferred (when possible) to a list of signs and symptoms. However, if a diagnosis is known and there are other signs or symptoms that are not generally part of the diagnosis, the diagnosis and each sign or symptom should be recorded separately.

If no diagnosis, disease or syndrome can be recognized, signs or symptoms should be described in the study subject's own words (verbatim) unless, in the opinion of the investigator, clarification of the subject's verbatim language is deemed necessary.

The AE term will subsequently be coded using MedDRA.

The AE term, date of AE onset, date of AE resolution (if applicable), seriousness, severity, causality, action taken for the AE, and outcome will be recorded in the eCRF.

Medical conditions that exist before signing the informed consent form will be recorded as part of medical history.

9.1 Reporting Period

For SAEs, the active reporting period to the sponsor begins from the time the patient provides informed consent, which is obtained prior to the patient's participation in the study, i.e., prior to undergoing any study-related procedure and/or receiving the investigational product, through the end of study visit (28-35 calendar days after the last administration of the investigational product). SAEs occurring to a subject after the active reporting period has ended should be reported to the sponsor if the investigator becomes aware of them; at a minimum, all SAEs that the investigator believes have at least a reasonable possibility of being related to the investigational product are to be reported to the sponsor.

Death must be reported if it occurs during the SAE reporting period after the last dose of investigational product, irrespective of any intervening treatment.

For AESIs, the active reporting period to the sponsor begins from the time the patient receives their first dose of investigational product, through the end of study visit (28-35 calendar days after the last administration of the investigational product). AESIs occurring to a patient after the active reporting period has ended should be reported to the sponsor if the investigator becomes aware of them and if the investigator believes they have at least a reasonable possibility of being related to the investigational product.

AEs (non-serious) should be recorded on the eCRF from the time the patient has taken at least one dose of investigational product through the patient's last visit. If a patient begins a new anti-cancer therapy, the AE reporting period for non-serious AEs ends at the time the new treatment is started.

9.2 Definition of Adverse Event

For purpose of this study, an AE is any untoward medical occurrence in a patient who participates in this study. The event need not necessarily have a causal relationship with the investigational product or procedure. Examples of AEs include but are not limited to:

- Clinically significant symptoms and signs (including abnormal laboratory findings)
- Changes in physical examination findings

- Hypersensitivity
- Drug abuse
- Drug dependency

Additionally, they may include the signs or symptoms resulting from

- Drug overdose
- Drug withdrawal
- Drug misuse
- Drug interactions
- Exposure during pregnancy
- Exposure via breast feeding
- Medication error
- Occupational exposure

Worsening of signs and symptoms of the malignancy under study should be reported as AEs in the appropriate section of the eCRF. Disease progression assessed by measurement of malignant lesions on radiographs or other methods should not be reported as AEs.

9.3 Definition of Adverse Event of Special Interest

For purpose of this study, an AESI is any untoward medical occurrence in a patient, serious or non-serious, who participates in this study that the Sponsor has identified to be notified of immediately. The event need not necessarily have a causal relationship with the investigational product.

AESI are required to be reported by the investigator to the Sponsor immediately (i.e., no more than 24 hours after learning of the event; see Section 9.12.1 for reporting instructions).

Adverse events of special interest for this study are the following:

- Thrombocytopenia Grade ≥ 3 (platelet count < 50 x 10^9/L)

9.4 Definition of Serious Adverse Events

An SAE is any untoward medical occurrence at any dose that:

- Results in death
- Is life-threatening (immediate risk of death)
- Requires inpatient hospitalisation or prolongation of existing hospitalisation
- Results in persistent or significant disability/incapacity (substantial disruption of the ability to conduct normal life functions)

- Results in congenital anomaly/birth defect
- Is an important medical event that may jeopardise the subject or may require medical intervention to prevent one of the outcomes listed above

Medical and scientific judgment should be exercised in determining whether an event is an important medical event. An important medical event may not be immediately life-threatening and/or result in death or hospitalisation. However, if it is determined that the event may jeopardize the subject or may require intervention to prevent one of the other AE outcomes, the important medical event should be reported as serious. Examples of such events are intensive treatment in an emergency room or at home for allergic bronchospasm; blood dyscrasias or convulsions that do not result in hospitalisation; or development of drug dependency or drug abuse.

Progression of the malignancy under study (including signs and symptoms of progression) should not be reported as an SAE unless the outcome is fatal within the safety reporting period. If the malignancy has a fatal outcome during the study or within the safety reporting period, then the event leading to death must be recorded as an AE and as an SAE with CTCAE Grade 5.

For fatal progression of malignancy cases, please record the progression of the underlying malignancy as an SAE. If a specific event can be attributed to the death, please also include the event term. For example, if the patient has a fatal pneumonia due to progression of rectum cancer, please record events ‘pneumonia’ and ‘progression of rectum cancer’.

Hospitalisation due to signs and symptoms of disease progression should not be reported as an SAE.

When reporting a SAE, the following questions should be considered and included in the description if applicable:

- Is it a common occurrence in the population under study?
- Was it “treatment-emergent”?
- Did it respond to de-challenge?
- Did it recur on re-challenge?
- Were there concomitant medications?
- Were pertinent laboratory and/or other tests done?
- Was there an obvious alternative cause?

9.4.1 Protocol-Specified Serious Adverse Events

There are no protocol-specified SAEs in this study. All SAEs will be reported by the investigator as described in previous sections and will be handled as SAEs in the safety database.

9.4.2 Potential Cases of Drug-Induced Liver Injury (Hy's Law Cases)

Abnormal values in aspartate aminotransferase (AST) and/or alanine aminotransferase (ALT) levels concurrent with abnormal elevations in total bilirubin level that meet the criteria outlined below in the absence of other causes of liver injury are considered potential cases of drug-induced liver injury (potential Hy's Law cases) and should always be considered important medical events.

The threshold of laboratory abnormalities for a potential case of drug-induced liver injury depends on the patient's individual baseline values and underlying conditions. Patients who present with the following laboratory abnormalities should be evaluated further to definitively determine the aetiology of the abnormal laboratory values:

- Patients with AST or ALT and total bilirubin baseline values within the normal range who subsequently present with AST or ALT values ≥ 3 times the upper limit of normal (X ULN) concurrent with a total bilirubin value ≥ 2 X ULN with no evidence of haemolysis and an alkaline phosphatase value ≤ 2 X ULN or not available.

- For patients with pre-existing ALT or AST or total bilirubin values above the upper limit of normal, the following threshold values should be used in the definition mentioned above:

    - For pre-existing AST **or** ALT baseline values above the normal range: AST or ALT values ≥ 2 times the baseline values and ≥ 3 X ULN or ≥ 8 X ULN (whichever is smaller)

      concurrent with

    - For patients with pre-existing values of total bilirubin above the normal range: Total bilirubin level increased from baseline by an amount of at least one time the upper limit of normal **or** if the value reaches ≥ 3 times the upper limit of normal (whichever is smaller).

The patient should return to the investigational site and be evaluated as soon as possible, preferably within 48 hours from awareness of the abnormal results. This evaluation should include laboratory tests, detailed history and physical assessment, and the possibility of hepatic neoplasia (primary or secondary) should be considered.

In addition to repeating measurements of AST and ALT, laboratory tests should include albumin, creatine kinase, total bilirubin, direct and indirect bilirubin, gamma-glutamyl transferase, prothrombin time (PT)/ international normalized ratio (INR), and alkaline phosphatase. A detailed history, including relevant information, such as review of ethanol, acetaminophen, recreational drug and supplement consumption, family history, occupational exposure, sexual history, travel history, history of contact with a jaundiced person, surgery, blood transfusion, history of liver or allergic disease, and work exposure, should be collected. Testing for acute hepatitis A, B, or C infection and liver imaging (e.g., biliary tract) may be warranted. All cases confirmed on repeat testing as meeting the laboratory criteria defined above, with no other cause for liver function test (LFT) abnormalities identified at the time should be considered potential Hy's Law cases irrespective of availability of all the results of

the investigations performed to determine aetiology of the abnormal LFTs. Such potential Hy's Law cases should be reported as SAEs.

9.5 Hospitalisation

Hospitalisation is defined as any initial admission (even less than 24 hours) in a hospital or equivalent healthcare facility or any prolongation of an existing admission. Admission also includes transfer within the hospital to an acute/intensive care unit (e.g., from the psychiatric wing to a medical floor, or medical floor to a coronary care unit). An emergency room visit does not necessarily constitute a hospitalisation; however, the event leading to the emergency room visit should be assessed for medical importance.

Hospitalisation does not include the following:

- Rehabilitation facilities
- Hospice facilities
- Respite care (e.g., caregiver relief)
- Skilled nursing facilities
- Nursing homes
- Same day surgeries (as outpatient/same day/ambulatory procedures)

Hospitalisation or prolongation of hospitalisation in the absence of a precipitating, clinical AE is not in itself an SAE. Examples include:

- Admission for treatment of a pre-existing condition not associated with the development of a new AE or with a worsening of the pre-existing condition (e.g., for work-up of persistent pre-treatment lab abnormality).
- Social admission (e.g., patient has no place to sleep).
- Administrative admission (e.g., for yearly physical examination).
- Protocol-specified admission during a study (e.g., for a procedure required by the study protocol).
- Optional admission not associated with a precipitating clinical AE (e.g., for elective cosmetic surgery).
- Hospitalisation for observation without a medical AE.
- Pre-planned treatments or surgical procedures.
  - These should be noted in the baseline documentation for the entire protocol and/or for the individual patient.
- Admission exclusively for the administration of blood products.

Diagnostic and therapeutic non-invasive and invasive procedures, such as surgery, should not be reported as AEs. However, the medical condition for which the procedure was performed

should be reported if it meets the definition of an AE. For example, an acute appendicitis that begins during the AE reporting period should be reported as the AE, and the resulting appendectomy should be recorded as treatment of the AE.

9.6 Severity Assessment

As required on the AE eCRFs, the investigator will report adverse events using concise medical terminology (verbatim) as well as collect on the eCRF the appropriate Common Terminology Criteria for Adverse Events (CTCAE) (version 5.0, publication date: November 27, 2017; (https://ctep.cancer.gov/protocolDevelopment/electronic_applications/docs/CTCAE_v5_Quick_Reference_8.5x11.pdf)

The following definitions of severity should be used to describe the maximum intensity of the adverse event.

Grade	Clinical Description of Severity
1	MILD; asymptomatic or mild symptoms; clinical or diagnostic observations only; intervention not indicated.
2	MODERATE; minimal, local or non-invasive intervention indicated; limiting age-appropriate activities of daily living (ADL).
3	SEVERE or medically significant but not immediately life-threatening; hospitalisation or prolongation of hospitalisation indicated; disabling; limiting self-care ADL.
4	LIFE-THREATENING consequences; urgent intervention indicated
5	DEATH

Note the distinction between the severity and the seriousness of an AE. A severe event is not necessarily an SAE. For example, a headache may be severe (interferes significantly with the patient's usual function) but would not be classified as serious unless it met one of the criteria for SAEs listed above.

9.7 Causality Assessment

The investigator's assessment of causality must be provided for all AEs (serious and non-serious). The investigator must record the causal relationship in the eCRF, as appropriate, and report such an assessment in accordance with the serious adverse reporting requirements if applicable.

An investigator's causality assessment is the determination of whether there exists a reasonable possibility that the investigational product(s) caused or contributed to an AE. Generally, the facts (evidence) or arguments to suggest a causal relationship should be provided. If the investigator does not know whether the investigational product caused the event, then the event will be treated as "related to investigational product" for reporting purposes. If the

investigator's causality assessment is "unknown but not related to investigational product", this should be clearly documented on study records.

In addition, if the investigator determines an SAE is associated with study procedures, the investigator must record this causal relationship in the source documents and eCRF, as appropriate, and report such an assessment in accordance with the SAE reporting requirements.

9.8 Exposure During Pregnancy

An exposure during pregnancy occurs if:

1. A female becomes, or is found to be, pregnant either while receiving or having been exposed (e.g., because of treatment or environmental exposure) to the investigational product(s); or the female becomes, or is found to be pregnant after discontinuing and/or being exposed to the investigational product(s)
    - An example of environmental exposure would be a case involving direct contact with the investigational product(s) in a pregnant woman (e.g., a nurse reports that she is pregnant and has been exposed to the investigational product(s)).
2. A male has been exposed (e.g., because of environmental exposure) to the investigational product(s) prior to or around the time of conception and/or is exposed during his partner's pregnancy.

If a study patient becomes or is found to be pregnant during the study patient's treatment with the investigational product(s), the investigator must submit this information to the sponsor, regardless of whether an SAE has occurred. This must be done irrespective of whether an AE has occurred and within 24 hours of awareness of the exposure. The information submitted should include the anticipated date of delivery. Follow-up is conducted to obtain general information on the pregnancy and its outcome for all reports with an unknown outcome. The investigator will follow the pregnancy until completion (or until pregnancy termination) and notify the sponsor of the outcome as a follow up to the initial report. In the case of a live birth, the structural integrity of the neonate can be assessed at the time of birth. In the event of a termination, the reason(s) for termination should be specified and, if clinically possible, the structural integrity of the terminated foetus should be assessed by gross visual inspection (unless pre-procedure test findings are conclusive for a congenital anomaly and the findings are reported). If the outcome of the pregnancy meets the criteria for an SAE (i.e., ectopic pregnancy, spontaneous abortion, intrauterine foetal demise, neonatal death, or congenital anomaly [in a live born baby, a terminated foetus, an intrauterine foetal demise, or a neonatal death]), the investigator should follow the procedures for reporting SAEs.

9.9 Occupational Exposure

An occupational exposure occurs when during the performance of job duties, a person (whether a healthcare professional or otherwise) gets in unplanned direct contact with the investigational product(s), which may or may not lead to the occurrence of an adverse event. An occupational exposure must be reported to the sponsor within 24 hours of the investigator's awareness, using the SAE Report form, regardless of whether there is an associated AE/SAE. Since the information does not pertain to a subject enrolled in the study, the information is not reported

on an eCRF. However, a copy of the completed SAE Report Form is maintained in the investigator site file.

9.10 Withdrawal Due to Adverse Events (See Also Section on Patient Withdrawal)

Withdrawal due to AEs should be distinguished from withdrawal due to other causes, according to the definition of AE noted earlier, and recorded on the appropriate AE eCRF page. When a patient withdraws because of an AESI or SAE, the AESI or SAE must be reported in accordance with the reporting requirements defined below.

9.11 Eliciting Adverse Event Information

The Investigator is to report all directly observed AEs and all AEs spontaneously reported by the study patient. In addition, each study patient will be questioned about AEs.

9.12 Reporting Requirements

Each AE is to be assessed to determine if it meets the criteria for AESI or SAEs. If an AESI or SAE occurs, expedited reporting will follow local and international regulations, as appropriate.

9.12.1 Serious Adverse Event Reporting Requirements

All SAEs will be reported from informed consent until the End of Study visit. All AESI will be reported from the first date of investigational product. An AESI/SAE that occurs after the End of Study visit and comes to the attention of the Investigator must be reported only if there is (in the opinion of the investigator) a reasonable causal relationship with the study drug.

AESI/SAEs must be reported to the sponsor or its representative within 24 hours of becoming aware of the event. If the SAE is fatal or life-threatening, notification to the sponsor or it’s representative must be made immediately, irrespective of the extent of available AE information.

- This is achieved by completing the SAE Report form and sending it to Bionical Emas by email or fax (all modules) with the relevant CRO Medical Monitor in copy:

**SAE CONTACT DETAILS:**	Bionical Emas<br>Fax: +44 (0)1462 600456<br>Email: drug.safety@bionical-emas.com
Module 2A	
Bionical Emas Medical Monitor	Jonathan Whitton<br>Email: jonathan.whitton@bionical-emas.com
Bionical Emas Medical Monitor (back-up)	Terence Goh<br>Email: terence.goh@bionical-emas.com

[redacted]

The Bionical Emas pharmacovigilance (PV) department, in close association with the Medical Monitor, will report applicable SAEs to the regulatory authorities within the legally required timeframe.

After review of an AESI/SAE report by the Bionical Emas PV department and/or the Medical Monitor, additional information may be requested (e.g., clinic or hospital records or procedure reports) to complete the report. If at the time the Investigator initially reports an AESI/SAE the event has not resolved, the Investigator must provide a follow-up report to the Bionical Emas Pharmacovigilance (PV) department as soon as it resolves (or upon receipt of significant information if the event is still ongoing).

The 24 hours' time window for reporting also applies to additional new information (follow-up) on previously forwarded AESI/SAE reports as well as to the initial and follow-up reporting of exposure during pregnancy, exposure via breastfeeding and occupational exposure cases. In the event the investigator does not become aware of the occurrence of an AESI/SAE immediately (e.g., if an outpatient study patient initially seeks treatment elsewhere), the investigator is to report the event within 24 hours after learning of it and document the time of his/her first awareness of the AE.

For all AESI/SAEs, the investigator is obligated to pursue and provide information to Bionical Emas in accordance with the reporting timeframe specified above. In addition, an investigator may be requested by Bionical Emas and/or the sponsor to obtain specific additional follow-up information in an expedited fashion. This information collected for AESI/SAEs is more detailed than that captured on the AE eCRF. In general, this will include a description of the AE in sufficient detail to allow for a complete medical assessment of the case and independent determination of possible causality. Information on other possible causes of the event, such as concomitant medications, vaccines and/or illnesses must be provided. In the case of a patient's death, a summary of available autopsy findings must be submitted as soon as possible to the sponsor or its designated representative.

9.12.2 Non-Serious Adverse Event Reporting Requirements

All AEs will be reported on the AE page(s) of the eCRF. It should be noted that the form for collection of SAE information is not the same as the AE eCRF. Where the same data are collected, the forms must be completed in a consistent manner. For example, the same AE term should be used on both forms. AEs should be reported using concise medical terminology on the eCRFs as well as on the form for collection of SAE information.

9.12.3 Sponsor's Reporting Requirements to Regulatory Authorities

Adverse event reporting, including SUSARs, will be carried out in accordance with applicable local regulations.

10 DATA ANALYSIS AND STATISTICAL METHODS

10.1 Analysis Populations

10.1.1 Intent-to-Treat (ITT) Population

The ITT population will include all enrolled patients with designated study drug assignment.

10.1.2 As-Treated (AT) Population

The AT population will include all patients who receive at least 1 dose of study treatment, with treatment assignments designated according to actual study treatment received.

- In each study part, the AT population will be the primary population for evaluating safety and treatment administration/compliance.

10.1.3 Evaluable for Response Population

Evaluable for Response Population: All subjects who received at least 1 dose of CT7001 and had measurable disease at baseline.

10.1.4 Pharmacokinetics (PK) Population

The PK population will include all patients who received at least one dose of CT7001 and fulvestrant, had at least one plasma concentration of CT7001 or fulvestrant above the lower limit of quantification and had no protocol deviations or other events that may impact PK analysis. The PK population will serve as the PK analysis set in all parts of the study.

10.2 Efficacy Analysis

Analyses of PFS will be based on the ITT population. Analyses of ORR, CBR and tumour size will be performed on the evaluable for response population.

All analyses will be performed by using SAS® Version 9.1.3 or higher. The Protocol outlines the main statistical methodology for this study, further details will be provided in the Statistical Analysis Plan (SAP).

The primary analyses of efficacy endpoints will be based on local radiologist's/investigator's assessments, using RECIST version 1.1. If required supportive analyses may be performed based on BICR of radiographic images and clinical information through a third-party core imaging laboratory.

Subgroup analyses may be performed. Of special interest will be the subgroups defined by the stratification factors:

- Patient has only non-measurable disease at baseline in accordance with RECIST v1.1, yes versus no.
- Presence of liver metastases, yes versus no.

10.2.1 Analysis of Progression-Free Survival

[redacted] PFS is defined as the time from the date of randomization to the date of the first documentation of objective progression of disease (PD) or death due to any cause, whichever occurs first. PFS data will be censored on the date of the last tumour assessment on study for patients who do not have objective tumour progression and who do not die while on study. Patients lacking an evaluation of tumour response after randomization will have their PFS time censored on the date of randomization with a duration of 1 day. Patients who start a new anti-cancer therapy prior to documented PD will be censored at the date of the last tumour assessment prior to the start of the new therapy. Further details related to the definition of censoring will be included in the SAP.

The primary analyses of PFS will be performed in the ITT population, based on local assessment of objective PD. A stratified log-rank test will be used to compare PFS time between the 2 treatment arms with a one-sided significance level of 0.1. The stratification factor(s) are specified in Section 5. If the 1-sided p-value is <0.1 this would indicate there is a less than 10% probability that the observed result could have occurred due to chance. This size of alpha error is consistent with the primary aim of Phase 2 studies which is to identify new agents or therapies that are sufficiently promising for advancing to subsequent Phase 3 development (Rubinstein et al., 2005).

The PFS time associated with each treatment arm will be summarized for the ITT population using the Kaplan-Meier method and displayed graphically where appropriate. Confidence intervals (CIs) for the 25th, 50th and 75th percentiles of the event-free time will be reported. The Cox Proportional hazards model will be fitted to compute the treatment hazard ratio and the corresponding 2-sided 95% as well as upper 1-sided 90% CI.

If required additional supportive PFS analyses for both populations may be conducted based on BICR of objective PD.

[redacted]

[redacted]

10.2.2 Objective Response

Objective response (OR) is defined as a CR or PR according to RECIST version 1.1 (Appendix C).

A patient will be considered to have achieved an OR if the patient has a CR or PR according to RECIST v.1.1 definitions. Otherwise, the patient will be considered as non-responders in the OR rate analysis. Additionally, patients with inadequate data for tumour assessment (e.g., no baseline or post-baseline assessment) will be considered as non-responders in the OR rate analysis.

The OR rate (ORR) will be estimated by dividing the number of patients with objective response (CR or PR) by the number of patients with measurable disease allocated to study treatment. An exact binomial 95% CI will be computed.

[redacted]

Primary ORR analyses will be based on the investigator's assessment. If required, additional analyses may be conducted based on BICR. The best overall response for each patient will be summarized by treatment arm.

The evaluable for response population will serve as primary population for ORR analysis

10.2.3 Duration of Response

Duration of response (DOR) is defined as the time from the first documentation of objective tumour response (CR or PR) to the first documentation of objective tumour progression or to death due to any cause, whichever occurs first. DOR data will be censored on the date of the last tumour assessment on study for patients who do not have objective tumour progression and who do not die due to any cause while on study. DOR will only be calculated for the subgroup of patients with an objective response.

[redacted]

10.2.4 Clinical Benefit Response

Clinical benefit response (CBR) is defined as CR, PR, or SD lasting ≥24 weeks recorded in the time period between enrolment and disease progression or death to any cause. The CBR rate will be estimated by dividing the number of patients with CR, PR, or SD ≥24 weeks by the number of patients in the particular analysis population. A 95% CI for the CBR rate will be provided.

[redacted]

The evaluable for response population will serve as primary population for CBR analysis.

10.2.5 Percent Change in Tumour Size

[redacted]

The evaluable for response population will serve as primary population for tumour size analysis.

10.2.6 Waterfall Plot Analysis

In Part A [redacted] the best percent change versus baseline in post-baseline aggregate tumour size measurements will be displayed graphically in form of Waterfall plots for the evaluable for response population. [redacted]

The evaluable for response population will serve as primary population for tumour size analysis.

10.3 Safety Analysis

The AT population will be the primary population for safety evaluation.

10.3.1 Adverse Events (AEs)

AEs will be classified using the medical dictionary for regulatory activities (MedDRA) classification system. Whenever possible, the severity of the toxicities will be graded according to the NCI CTCAE version 5.0, publication date: November 27, 2017; https://ctep.cancer.gov/protocolDevelopment/electronic_applications/docs/CTCAE_v5_Quick_Reference_8.5x11.pdf.

AEs will be summarized by the frequency of patients experiencing treatment emergent adverse events corresponding to body systems and MedDRA preferred term. AEs will be graded by worst NCI CTCAE v5.0 Grade. AEs will be summarized by cycle and by relatedness to study treatment. The frequencies of the worst severity grade observed will be displayed by study treatment. Detailed information collected for each AE will include a description of the event, duration, whether the AE was serious, intensity, relationship to study drug, action taken, and clinical outcome. Emphasis in the analysis will be placed on AEs classified as treatment emergent.

AEs leading to death or discontinuation of trial treatment, events classified as NCI CTCAE v5.0 Grade 3 or higher, trial drug-related events, and serious adverse events will be considered with special attention.

10.3.2 Laboratory Abnormalities

Haematology, serum chemistry and urinalysis data will be summarized by cycle. The laboratory results will be graded according to the NCI CTCAE v5.0 severity grade. The frequencies of the worst severity grade observed will be displayed by study treatment. For parameters for which an NCI CTCAE v5.0 scale does not exist, the frequency of patients with values below, within, and above the normal ranges will be summarized by treatment.

10.3.3 Electrocardiogram (ECG) Analysis

All ECGs obtained during the study will be evaluated for safety. The triplicate data will be averaged, and all summary statistics and data presentations will use the triplicate averaged data. Any data obtained from ECGs repeated for safety reasons after the nominal time-points will not be averaged along with the preceding triplicates. For all patients in the AT population, individual change in QTcF will be calculated for each nominal post-baseline time point. These individual changes will be summarized using descriptive statistics.

10.4 Pharmacokinetic Analysis

Summary statistics for the PK analysis set will be provided for trough concentrations of CT7001 and fulvestrant in Part A [redacted] All patients treated with CT7001 and fulvestrant for whom drug plasma concentration results (from at least 1 visit) are available will be included in the analysis. Incidence and type of genotypes and copy number

variation of CYP2D6, other CYP genes and drug transporters genes will be summarised (all study parts).

In addition, the relationship between exposure and safety and efficacy endpoints and/or the relationship between trough concentration and potential covariates (e.g., CYP2D6 polymorphisms) will be explored, based on emerging safety and efficacy data. The effect of patient characteristics (including but not limited to race, ethnicity, age, CYP and drug transporter gene polymorphisms) on trough concentrations of CT7001 (all study parts) may be investigated. These data may be combined with data from other CT7001 studies. The results of these modelling analyses may be reported separately from the clinical study report.

10.5 Biomarker Analysis

Appropriate statistical methods will be used to investigate any possible relationship of candidate biomarkers with the recorded efficacy outcomes.

10.6 Analysis of Other Endpoints

Descriptive statistics will be used to summarise patient characteristics, treatment administration/compliance and biomarkers. Data will be displayed graphically, where appropriate.

11 ETHICS

11.1 Institutional Review Board (IRB)/Independent Ethics Committee (IEC)

It is the responsibility of the investigator to have prospective approval of the study protocol, protocol amendments, patient information sheets, informed consent documents, and other relevant documents, e.g., recruitment advertisements, if applicable, from the IRB/IEC. The sponsor or its delegate will supply relevant material for the Investigator to submit to the IRB/IEC for review and approval.

All correspondence with the IRB/IEC should be retained in the Investigator File. Copies of IRB/IEC approvals should be forwarded to the sponsor or its delegate. The only circumstance in which an amendment may be initiated prior to IRB/IEC approval is where the change is necessary to eliminate apparent immediate hazards to the patients. In that event, the investigator must notify the IRB/IEC and the sponsor or its delegate in writing immediately after the implementation.

The IRB/IEC will be provided with reports at the interval required (not to exceed 1 year) and a report after the completion or discontinuation of the Investigator's participation in the study.

11.2 Ethical Conduct of the Study

The study will be conducted in accordance with legal and regulatory requirements, as well as the general principles set forth in the International Ethical Guidelines for Biomedical Research

Involving Human Patients (Council for International Organizations of Medical Sciences 2002), Guidelines for Good Clinical Practice (International Council for Harmonization (ICH) 1996), and the Declaration of Helsinki (World Medical Association 1996 & 2008). In addition, the study will be conducted in accordance with the protocol, the ICH guideline on harmonization guideline for Good Clinical Practice (GCP), and applicable local regulatory and data protection requirements and laws.

11.3 Patient Information and Confidentiality

All parties will ensure protection of the personal data of study subjects and will not include the names of study subjects on any sponsor forms, reports, publications, or in any other disclosures, except where required by laws. A subject's name, address, birth date and other identifiable data will be replaced by a numerical code consisting of a numbering system provided by Carrick or its delegate to de-identify the study subject. In case of data transfer, Carrick will maintain high standards of confidentiality and protection of the study subjects' personal data.

11.4 Patient Consent

The informed consent document must comply with ICH GCP, local regulatory requirements, and legal requirements. The informed consent form(s) used during the informed consent process must be reviewed by the sponsor, approved by the IRB/IEC and available for inspection. The investigator must ensure that each study subject is fully informed about the nature and objectives of the study and possible risks associated with participation. The subjects will be informed that participation is voluntary and that they can withdraw from the study at any time.

The investigator, or a person designated by the investigator, will obtain written informed consent from each subject before any study-specific activity is performed. The investigator will retain the original of each subject's signed consent document(s). The study subject will be provided with a copy of the signed informed consent form(s).

A copy of the ICF will be given to the subject, and the original ICF will be maintained with the subject's study records.

Separate consent will be obtained for participation in the treatment study and related mandatory procedures, and for optional procedures including but not limited to biomarker analyses, pharmacogenomic analyses and tumour biopsies.

Study subjects can withdraw their consent at any time, in which case the investigator must notify the sponsor or its delegate in writing.

11.5 Potential Risks and Benefits

11.5.1 Potential Risks

The nonclinical and emerging clinical safety profile of CT7001 have not identified risks that would preclude investigation in the advanced breast cancer setting. The currently available clinical safety and tolerability data must be considered as preliminary. However, the available data has shown appropriate safety and tolerability for proceeding to investigation in patients

with locally advanced or metastatic HR+ BC and documented disease progression to previous standard of care treatment, including an aromatase inhibitor, a CDK/4/6 inhibitor, and optionally tamoxifen, everolimus and one line of prior chemotherapy.

At 120 mg, 240 mg OD and 360 mg OD CT7001 has been generally well tolerated. Adverse drug reactions of note were G1-2 nausea, vomiting and diarrhoea. At 480 mg OD, 3/6 subjects experienced a DLT (G3 diarrhoea, oral mucositis and vomiting). At 180 mg BID, 2/8 patients experienced DLTs (Grade 4 thrombocytopenia, Grade 3 weight loss, Grade 3 anorexia, Grade 3 dysphagia/oesophagitis and Grade 3 heartburn). 360 mg OD has been determined as maximum tolerated dose and preliminary recommended Phase 2 dose.

At 240 mg OD and 360 mg OD there appears to be a ~20% drop in platelet count in all patients. This appears over the first 15 days on study and then is stable for the duration on treatment; in the majority of patients this is within the normal range of platelet counts. All changes in platelet counts appear fully reversible upon discontinuation of CT7001. There have been 16 platelet related AEs reported:

- 2 events of Grade 4 thrombocytopenia (1 at 180mg BID and 1 at 360mg OD); the event at 180mg BID was associated with minor nose bleeding
- 1 event of Grade 3 thrombocytopenia (at 360mg OD)
- 2 events of Grade 2 thrombocytopenia (at 360mg OD)
- 1 event of Grade 2 platelet count decreased (at 360mg OD)
- 6 events of Grade 1 thrombocytopenia (at 360mg OD)
- 4 events of Grade 1 platelet count decreased (1 at 240mg OD and 3 at 360mg OD)

Other laboratory AEs have been rare and mild. The recorded laboratory abnormalities include increase in liver transaminases, prolongation of QTc, 1st degree AV block and anaemia. Of note, a decline in neutrophil count is not anticipated. Accordingly, fever or infection as a clinical complication of severe neutropenia is not expected.

It has been anecdotally noted, by some Investigators, that patients with well controlled diabetes at the time of entry to the study have struggled to control their blood glucose while being dosed with CT7001. The data collected as part of the study neither support or refute this observation. Moving forward, however an assessment of HbA1c will be made in all patients in the study, and those with diabetes at entry will have assessments of fasting glucose.

The current study includes mandatory procedures for safety monitoring. Various sections of the study protocol and appendices provide instructions and/or guidance to mitigate the risk of severe treatment-emergent toxicity and in case adverse effects may occur for their prompt and proper medical management.

Non-clinical data from hERG testing and *in vivo* safety pharmacology studies suggest a low potential of CT7001 for clinically significant prolongation of QTc, cardiovascular, respiratory or central nervous system toxicity (see the IB).

Clinical drug interaction data of CT7001 are currently not available. In vitro cytochrome P450 (CYP) studies suggest that CT7001 is unlikely to cause clinically significant hepatic drug interactions through inhibition of CYP1A2, 2C9, 2B6, 2C8 and 3A5. Co-medication with drugs or food that modulate 2D6 or 2C19 and particularly CYP3A4 may affect the exposure of CT7001, and there is a potential for CT7001 to inhibit intestinal and hepatic CYP3A4 which

may be clinically significant for CYP3A4 substrates. The potential of CT7001 to inhibit transporter proteins has not yet been studied. The risk of clinically significant drug interaction is mitigated by provisions in the study protocol (Sections 4.2, 6.17.2 and Appendix B) not to take certain drugs or food or doing so with caution. Importantly, there is a low likelihood of clinically relevant drug interaction with fulvestrant (see Section 2.5).

CT7001 exhibits pH-dependent solubility. Medication which increases gastric pH (such as PPIs, H2 antagonists) may reduce the bioavailability of CT7001 and should be avoided unless clinically required. This is described in Section 6.17.2.

Preliminary *in vitro* studies have shown low potential of CT7001 for mutagenicity and genotoxicity. However, pregnancy is listed as a contra-indication for the use of fulvestrant in the Faslodex® SPC and under warnings and precautions in the FDA label (Appendix E). Accordingly, patients must be informed of the potential risk of reproductive toxicity and the study protocol requires women with childbearing potential to agree to use adequate contraception during the study and for 24 months after the final study dose (Section 4.1). Female patients also must have a negative pregnancy test prior to enrolment (Section 18, Table 6).

It is currently unknown whether CT7001 is excreted in human breast milk, and lactation is listed as a contra-indication for the use of fulvestrant in the Faslodex® SPC (Appendix E). Accordingly, women who are breastfeeding are excluded from the study (Section 4.2).

The effects of CT7001 and of fulvestrant on fertility in humans have not been studied.

Fulvestrant has no or negligible influence on the ability to drive or use machines (see Faslodex® SPC and FDA label in Appendix E). The effects of CT7001 on the ability to drive or operate machinery are unknown. Accordingly, caution should be observed by patients when driving or operating machinery.

Please refer to the Faslodex® SPC and FDA label in Appendix E for information on undesirable effects and adverse drug reactions which have been reported for fulvestrant. The most common adverse reactions occurring in ≥5% of patients receiving Faslodex® 500 mg were injection site pain, nausea, bone pain, arthralgia, headache, back pain, fatigue, pain in extremity, hot flash, vomiting, anorexia, asthenia, musculoskeletal pain, cough, dyspnoea, and constipation. Increased hepatic enzymes (ALT, AST, ALP) occurred in >15% of Faslodex® patients and were not dose-dependent.

11.5.2 Potential Benefits

All patients in this study will receive fulvestrant at standard current dosing of 500 mg. In patients who had previously received an AI, fulvestrant can be considered a commonly used standard of care treatment option in routine clinical practice.

It is currently not known whether CT7001 may be of additional clinical benefit to patients with advanced HR-positive BC when combined with fulvestrant in patients who had previously received an AI and a CDK4/6 inhibitor. However, the available scientific, biological and non-clinical efficacy data provide a strong rationale for clinical investigation of this combination.

CDK7 has three critical roles in cancer (Section 2.2 and IB). Two of these mechanisms appear of particular relevance in HR-positive BC, which are loss of sensitivity to hormonal therapy via phosphorylation of ERα (Chen et al., 2000) and the transcriptional coactivator MED1 (Rasool et al, 2019) and acceleration of progression through the cell cycle via phosphorylation of other members of the CDK family (such as CDK2, 4 and 6) (Fisher, 2005; Fisher and Morgan, 1994; Schachter and Fisher, 2013; Schachter et al., 2013).

CT7001 inhibited CDK7-mediated phosphorylation of RNA PolII and Rb phosphorylation in the oestrogen-sensitive MCF7 cell line in a time- and dose-dependent manner, the Rb effect produced via inhibition of CDK2/4/6 activity (Patel et al., 2018). CT7001 inhibited phosphorylation of serine 118, the ER site targeted by CDK7, in culture and in tumour xenografts of MCF-7 cells, indicating that CT7001 inhibits ER activity (Patel et al., 2018; Ali et al, 2018). Cell growth inhibition studies showed broad activity of CT7001 against a wide range of tumour cell lines, including the HR-positive, oestrogen-sensitive MCF-7 cell line (Patel et al., 2018; Ali et al., 2018; Ainscow et al., 2018; Clark et al., 2017). CT7001 also exhibited encouraging *in vivo* activity in MCF-7 xenograft studies as a single agent but most notably a strong combinatorial effect with anti-hormonal drugs (Patel et al., 2018; Ali et al., 2018). Of note, recent studies in cell lines with acquired resistance to CDK4/6 inhibitors have shown similar sensitivity to CT7001 in the parental and resistant lines (unpublished data). This has been independently corroborated by similar findings for other CDK7 inhibitors (Guarducci et al., 2018). Furthermore, in cells resistant to CDK4/6 inhibition CDK7 was identified as a top ranked essential gene and inhibition of CDK7 in combination with fulvestrant showed synergistic activity (Guarducci et al., 2018).

When evaluated as a single agent in the first-in-human ascending dose study (Module 1A), CT7001 has shown preliminary signals of anti-tumour effect in various types of solid tumours (Section 2.3.2.7). Three patients had stable disease for ≥18 weeks, including 1 patient with a best change in lesion size of -21% on CT scan. Five patients had stable disease for ≥12+ weeks, including 1 patient HR-positive BC. This patient also had a decline by 30% in the blood biomarker CA-153.

11.5.3 Overall Risk/Benefit Assessment

Fulvestrant at the dosing regimen to be used in the present study is an approved standard treatment option in patients with HR-positive advanced BC and has a well-established risk/benefit profile. As discussed in Section 2.5, the risk of clinically relevant DDI between fulvestrant and CT7001 is low. The available nonclinical and clinical safety data of CT7001 suggest a low probability that patients in the present study might get exposed to a major or unacceptable safety risk. The study protocol and procedures include multiple measures aimed at minimizing potential risks as much as feasible. It is currently not known whether CT7001 may be of additional clinical benefit to patients with advanced HR-positive BC when combined with fulvestrant in patients who had previously received an AI and a CDK4/6 inhibitor. However, the available scientific, biological and non-clinical efficacy data, the clinical safety, PK, PDc and preliminary efficacy data of CT7001 as monotherapy in Module 1A, and the continuing need of enhancing/restoring clinical sensitivity to established endocrine treatments through rationale combination with agents which have a novel mechanism of action, provide a strong scientific, mechanistic and clinical rationale for investigation of the combination of CT7001 with fulvestrant in that patient population.

Each part of the current study is carefully designed to ensure a proper balance of risk versus benefit for participating patients. Based on all considerations, the overall assessment of potential risk vs benefit appears to support the investigation of CT7001 in combination with fulvestrant as intended in the present study.

11.6 Patient Recruitment

Investigator databases may be used to aid patient recruitment. In case advertisements are used they must have received prior approval by IRB/IEC.

11.7 Reporting of Safety Issues and Serious Breaches of the Protocol or ICH GCP

In the event of any prohibition or restriction imposed (i.e., clinical hold) by an applicable competent authority in any area of the world, or if the investigator is aware of any new information which might influence the evaluation of the benefits and risks of the investigational product, Carrick or its delegate should be informed immediately. In addition, the investigator will inform Carrick immediately of any urgent safety measures taken by the investigator to protect the study patients against any immediate hazard, and of any serious breaches of this protocol or of ICH GCP that the investigator becomes aware of.

12 QUALITY CONTROL AND QUALITY ASSURANCE

During study conduct, Carrick or its agents will conduct periodic monitoring visits to ensure that the study protocol and Good Clinical Practices (GCPs) are being followed. The monitors may review source documents to confirm that the data recorded on eCRFs is accurate. The investigator and institution will allow Carrick monitors or its agents as well as appropriate regulatory authorities direct access to source documents to perform this verification. The study site may be subject to review by an Institutional Review Board (IRB) or Independent Ethics Committee (IEC) and/or to quality assurance audits performed by Carrick or companies working with or on behalf of Carrick and/or to inspection by appropriate regulatory authorities. It is important that the investigator(s) and their relevant personnel are available during the monitoring visits and possible audits or inspections and that adequate time is devoted to the process.

13 DATA HANDLING AND RECORD KEEPING

13.1 Case Report Forms/Electronic Data Record

As used in this protocol, the term CRF should be understood to refer to either an electronic data record, a paper form or both, depending on the data collection method used in this study.

A CRF is required and should be completed for each included patient. The completed original CRFs are the sole property of Carrick and should not be made available in any form to third parties, except for authorized representatives of Carrick or appropriate regulatory authorities, without written permission from Carrick.

The investigator has ultimate responsibility for the collection and reporting of all clinical, safety and laboratory data entered on the CRFs and any other data collection forms including source documents and ensuring that they are accurate, authentic / original, attributable, complete, consistent, legible, timely (contemporaneous), enduring and available when required.

The CRFs must be signed by the investigator or by an authorized staff member to attest that the data contained on the CRFs is true. Any corrections to entries made in the CRFs, source documents must be dated, initialled and explained (if necessary) and should not obscure the original entry.

In most cases, the source documents are the hospital's or the physician's patient chart. In these cases, data collected on the CRFs must match the data in those charts. In some cases, the CRF, or part of the CRF, may also serve as source documents. In these cases, a document should be available at the investigator's site as well as at Carrick and clearly identify those data that will be recorded in the CRF, and for which the CRF will stand as the source document.

13.2 Record Retention

To enable evaluations and/or audits from regulatory authorities or Carrick or its designated agents the investigator agrees to keep records, including the identity of all participating patients (sufficient information to link records, e.g., CRFs and hospital records), all original signed informed consent documents, copies of all CRFs, safety reporting forms, source documents and detailed records of treatment disposition, and adequate documentation of relevant correspondence (e.g., letters, meeting minutes, telephone calls reports). The records should be retained by the investigator as long as is required by International Council for Harmonisation (ICH) guidelines, local regulations, or as specified in the Clinical Study Agreement (CSA), whichever is longer. If the investigator becomes unable for any reason to continue to retain study records for the required period (e.g., retirement, relocation), Carrick should be prospectively notified. The study records must be transferred to a designee acceptable to Carrick, such as another investigator, another institution, or to an independent third party arranged by Carrick. Investigator records must be kept for a minimum of 15 years after completion or discontinuation of the study or for longer if required by applicable local regulations. The investigator must obtain Carrick's written permission before disposing of any records, even if retention requirements have been met.

14 DEFINITION OF END OF TRIAL

14.1 End of Trial in all Participating Countries

End of Trial in all participating countries is defined as Last Patient Last Visit in the final module.

14.2 End of Trial in a Member State of the European Union

In a Member State of the European Union, end of trial is defined as the time at which it is deemed that sufficient patients have been recruited and completed the study as stated in the regulatory application (i.e., Clinical Trial Application (CTA)) and ethics application in the Member State. Poor recruitment (recruiting less than the anticipated number in the CTA) by a Member State is not a reason for premature termination but is considered a normal conclusion to the study in that Member State.

15 EARLY DISCONTINUATION OF TRIAL

Premature termination of this study may occur because of a regulatory authority decision, change in opinion of the IRB/IEC, drug safety problems, or at the discretion of Carrick. In addition, Carrick retains the right to discontinue development of CT7001 at any time. If a study is prematurely terminated or discontinued, Carrick will promptly notify the investigators and regulatory authorities. After notification, each investigator must contact all participating patients and the hospital pharmacy (if applicable) within a week of notification. Investigators also must communicate the decision and reason(s) for discontinuation to the IEB/IEC. As directed by Carrick, all study materials must be collected and all eCRFs completed to the greatest extent possible.

16 ADMINISTRATIVE PROCEDURES AND CONSIDERATIONS

16.1 Data Monitoring Committee

A blinded DMC will monitor the safety data on a periodic basis. The DMC will make recommendation as to whether the trial should continue based on ongoing reviews of safety and efficacy data. In Part A, the DMC will determine whether and when to open cohort 2 and which dosing regimen to advance to Part B. [redacted]

Membership and governance of the DMC are outlined in a separate charter.

16.2 Protocol Amendments

Any substantive change in the study requires a protocol amendment. As described in Section 5, it will not require a protocol amendment if DMC were to permit administration of CT7001 after a meal based on no clinically significant effect on bioavailability demonstrated in the food effect study (Module 4). Protocol amendments must be reviewed and agreed to by Carrick and the Principal Investigator(s) and approved by applicable regulatory authorities and IRB/IECs before implementation.

16.3 Clinical Study Report

A final clinical study report (CSR) will be prepared in accordance with ICH guidelines on structure and contents of CSRs and any applicable regulatory and legal requirements and the completed CSR will be submitted to all relevant authorities within required time. Considering the multi-module nature of the current study, separate CSRs may be prepared and submitted for each completed study module.

16.4 Financing and Insurance

Financing and insurance will be addressed in a separate clinical study agreement.

17 PUBLICATION OF STUDY RESULTS

Publication of study results is discussed in the Clinical Study Agreement.

17.1 Communication of Results by Carrick

Carrick fulfils its commitment to publicly disclose clinical trial results through posting the results of this study on eudract.ema.europa.eu/ (EudraCT) and www.clinicaltrials.gov (ClinicalTrials.gov). Carrick posts the results of all studies that it has registered on EudraCT and/or ClinicalTrials.gov regardless of the reason for registration.

At EudraCT, Carrick posts the results ≤ 12 months after the end of the trial.

For posting of results at ClinicalTrials.gov, the timing depends on the status of the Carrick product:

- For studies involving a Carrick product whose drug development is discontinued before approval, Carrick posts the results within one year of discontinuation of the program (if there are no plans for out-licensing) or within two years (if out-licensing plans have not completed).
- For studies involving products that are not yet approved in any country, Carrick posts the results of completed studies within 30 days of US regulatory approval or one year after the first ex-US regulatory approval of the product (if only submitted for approval ex-US).

- For studies involving products applicable under the US Food and Drug Administration Amendments Act of 2007 (FDAAA), i.e., FDA approved products, Carrick posts results within one year of the primary outcome completion date (PCD).
    - Primary Completion Date is defined as the date that the final patient was examined or received an intervention for the purposes of final collection of data for the primary outcome, whether the clinical trial concluded according to the pre-specified protocol or was terminated.
- For studies involving products approved in any country, but not FDA approved, Carrick posts results one year from last patient, last visit (LPLV).

17.2 Publications by Investigators

Publication of study results is also provided for in the Clinical Study Agreement between Carrick and the Institution. In this section entitled Publications by Investigators, the defined terms shall have the meanings given to them in the Clinical Study Agreement.

For all publications relating to the study, Institutions will comply with recognized ethical standards concerning publications and authorship, including Section II "Ethical Considerations in the Conduct and Reporting of Research" of the Uniform Requirements for Manuscripts Submitted to Biomedical Journals, http://www.icmje.org/index.html#authorship, established by the International Committee of Medical Journal Editors.

Carrick has no objection to publication by Investigators of any information collected or generated by Investigators, whether or not the results are favourable to the Investigational Product. However, to ensure against inadvertent disclosure of Confidential Information or unprotected Inventions, Investigators will provide Carrick an opportunity to review any proposed publication or other type of disclosure before it is submitted or otherwise disclosed.

Investigators will provide manuscripts, abstracts, or the full text of any other intended disclosure (poster presentation, invited speaker or guest lecturer presentation, etc) to Carrick at least 30 days before they are submitted for publication or otherwise disclosed. If any patent action is required to protect intellectual property rights, Investigators agree to delay the disclosure for a period not to exceed an additional 60 days.

Investigators will, on request, remove any previously undisclosed Confidential Information (other than the Study results themselves) before disclosure.

If the Study is part of a multi-centre study, Investigators agree that the first publication is to be a joint publication covering all centres. However, if a joint manuscript has not been submitted for publication within 12 months of completion or termination of the Study at all participating sites, Investigators are free to publish separately, subject to the other requirements of this Section.

18 SCHEDULE OF ASSESSMENTS

Table 6: Schedule of Events

The investigator may schedule visits (unplanned visits) in addition to those listed on the schedule of activities to conduct evaluations or assessments required to protect the wellbeing of the patient.

**Protocol Activity**	**Screening** [a]	**Active Treatment Phase** [b] **- One Cycle = 28 days**			**End of Treatment/ Withdrawal** [d, cc]	**Post-Treatment Follow-Up** [e]
		**Cycles 1 and 2**		**Cycles ≥3** [dd]		
**Study Day**	**Within 28 days prior to allocation to study therapy unless specified otherwise**	**Day 1** [c]	**Day 15**	**Day 1**		
**Visit Window**		**±2 days**	**±2 days**	**±7 days**	**Within 28 days**	
Informed Consent [f]	X					
Medical/Oncological History [g]	X					
Signs/Symptoms [h, cc]	X	Pre-dose	Pre-dose	Pre-dose	X	
Physical Examination/Vital Signs [i, cc]	X	Pre-dose	Pre-dose	Pre-dose	X	
ECOG Performance Status	X	Pre-dose	Pre-dose	Pre-dose	X	
Laboratory Studies						
Haematology [j]	X	Pre-dose	Pre-dose	Pre-dose	X	
Serum Chemistry [k]	X	Pre-dose	Pre-dose	Pre-dose	X	
Fasted Glucose [ee]		Pre-dose	Pre-dose	Pre-dose	Pre-dose	
Pregnancy Test, Serum Oestradiol and FSH (if applicable) [l]	X					
Urinalysis [m]	X	Pre-dose	Pre-dose	Pre-dose	X	
Triplicate 12-Lead ECGs [cc]	X	Pre-dose		Pre-dose	X	
Tumour Assessments						
CT/MRI Scans and Clinical Evaluation of Superficial Disease [n, cc]	X	Performed/repeated as described in footnote [n]			X	X
Radionuclide Bone Scan, Whole Body [o, cc]	X	Performed/repeated as described in footnote [o]			X	X
Other Clinical Assessments						
Adverse Event Reporting [p]	Recorded/reported from 28 days prior to the start of study treatment up to 28 days after the last dose of study treatment					
Concomitant Medications/Treatments						
Patient Feedback [q]					X	
Pharmacokinetics [r, cc]		Pre-dose [r]	Pre-dose [r] (C1 only)	Pre-dose (every other Cycle)	X [r]	
CYP2D6 Polymorphisms [s]		Pre-dose (C1 only)				
IP Diary [t, cc]		X	X	X		
Study Treatment						
[redacted]		[redacted]				

Protocol Activity	Screening	Active Treatment Phase - One Cycle = 28 days				End of Treatment/ Withdrawal	Post-Treatment Follow-Up
CT7001 [redacted] u, cc		Daily Dosing					
Fulvestrant [redacted] v		X	X (C1 only)	X			
		Cycle 1	Cycle 1	Cycle 2	Cycles ≥3		
Study Day	Within 28 days prior to allocation to study therapy unless specified otherwise	Day 1	Day 15	Day 1	Day 1		
Visit Window		Within 48 hours before first drug administration	Before drug administration	Before drug administration	Before drug administration		
Optional Procedures and Assessments [w]							
Informed Consent [w]	X						
ctDNA [x, cc]	X	Pre-dose		Pre-dose	Pre-dose (every other Cycle)	X (within 2 weeks after disease progression)	
Pharmacogenomics [y]		Pre-dose					
Exploratory Research [z, cc]		Pre-dose		Pre-dose	Pre-dose (every other Cycle)	X (within 2 weeks after disease progression)	
Tumour Biopsy [aa, cc]	X (within 10 days prior to allocation to study therapy)			X (Cycle 2 Day 15 ± 7 days)		X (within 2 weeks after disease progression)	
Archival Tumour Material [bb]		To be collected at any time during active treatment phase					

a. **Screening**:
- Prior to any protocol required assessments being performed informed consent must be obtained from the patient.
- In case tumour assessments by CT or MRI were performed as part of routine procedures before the signing of the informed consent but within 28 days prior to allocation to study therapy, those assessments do not need to be repeated and can be used as baseline assessments as long as:
  - The tests were performed per the method requirements described in Section 8.1 and in footnote n.
  - Proper documentation is available in the patient's source notes that the radiographic procedures were performed as part of standard of care.
- Radionuclide bone scans performed as routine procedure within 12 weeks prior to allocation to study therapy are also accepted as baseline assessment if they meet the two requirements described above.

b. **Active Treatment Phase**:
- The active treatment phase is ongoing as long as the patient is receiving both study drugs (i.e., CT7001 [redacted] and fulvestrant).
- Assessments should be performed prior to dosing on the visit day unless otherwise indicated.
- A treatment cycle consists of 28 days but could be longer if persistent toxicity delays initiation of the subsequent cycle. In that case, Day 1 assessments of the subsequent cycle will be performed when study treatment is resumed, coinciding with the day the CT7001 [redacted] treatment begins.
- Day 1 procedures which were performed prior to knowing that the start of the cycle needs delay do not need to be repeated if:
  - Not required to determine whether toxicity has sufficiently resolved to resume study therapy.
  - Performed within 7 days prior to restart of study therapy.

- Fulvestrant injections will be given every 28 days (±7 days) except Cycle 1 during which it will be administered on Days 1 and 15 (±2 days). In case toxicity requires delay of the subsequent cycle, fulvestrant injections will be postponed accordingly.

c. **Serum chemistry, haematology, physical examination and ECG** are not required on Day 1 of Cycle 1 if performed as part of screening within 7 days prior to allocation to study therapy.

d. **End of Treatment/Withdrawal**: Visit and required tests to be performed as soon as possible but no later than 28 days from the last dose of investigational products on study and prior to initiation of any new anticancer therapy.

- In the event study therapy was discontinued due to death or documented disease progression (Section 8.1.2), the end of treatment visit represents the final visit.
- Obtain tumour assessment by:
  - CT or MRI, as applicable, unless performed within the previous 8 weeks.
  - Bone scan unless performed within the previous 12 weeks.
- Patients who continue on-study Fulvestrant monotherapy: The end of treatment schedule of events should be completed at the time of CT7001 discontinuation or prior to the administration of next dose of fulvestrant. The modified end of treatment visit will be completed at the discontinuation of Fulvestrant.
- For patients who continue to receive clinical benefit despite the individual patient or the module meeting the primary endpoint, then at the discretion of the Investigator, with approval of the Sponsor, and in compliance with national regulations the Investigator may initiate completion of study participation and apply for Managed Access of Investigational Product.

e. **Post Treatment Follow-up**: Patients who discontinue study treatment for any reason other than objective disease progression or death will continue to have tumour assessments performed by CT or MRI, as applicable, and clinical evaluation in case of superficial disease every 8 weeks (±7days) for the first year, and then every 12 weeks (±7 days) in subsequent years (calculated from the date of cycle 1 day 1) until documented progression, death, onset of new anticancer therapy (Part A [redacted]) or Module 2 LPLV, whichever occurs first. [redacted]

- With regard to bone scans, please refer to footnote o.

f. **Informed Consent**: Informed consent must be obtained prior to any protocol required assessments being performed (with the exception of certain imaging assessments if meeting the criteria defined in the Section 7.1 (Screening) and footnote a.

g. **Medical/Oncological History**: To include information on prior anticancer treatments, alcohol consumption and tobacco use.

h. **Signs and Symptoms** (tumour-related or otherwise) at baseline will be recorded at the Cycle1 Day1 visit prior to initiating treatment and then reported as adverse events during the trial if they worsen in severity or increase in frequency.

i. **Physical Examination/Vital Signs**: Includes an examination of all major body systems and breasts, height (at screening only), weight, supine blood pressure, pulse rate, respiratory rate and body temperature. May be performed by a physician, registered nurse or other qualified health care provider.

j. **Haematology** includes haemoglobin, WBC, absolute neutrophil count, platelet count and reticulocytes. Additional haematology tests may be performed if clinically indicated. [redacted]

k. **Serum Chemistry** includes HbA1c, AST, ALT, alkaline phosphatase, creatine kinase, glucose, sodium, potassium, magnesium, total calcium, total bilirubin, blood urea nitrogen (BUN), serum creatinine and albumin. Additional serum chemistries tests may be performed as clinically indicated, including tumour markers (in accordance with local practice).

l. **Pregnancy Test** (serum) at screening, within 7 days prior to first dose of CT7001 or [redacted] only for women of childbearing potential. (Tests may be repeated during the active treatment phase or later if so required by IRB/IECs or by local regulations.)

- Serum oestradiol and follicle stimulating hormone (FSH) levels are analysed at screening to confirm postmenopausal status of women <50 years old or <60 years old and not amenorrhoeic for at least 12 consecutive months with no alternative pathological or physiological cause.

m. **Urinalysis** includes visual examination and a dipstick test for blood, glucose and protein. If either is abnormal, a microscopic examination should be performed as well.

n. **CT or MRI Scans**: Please refer to Section 8.1 for instructions regarding use of CT scan or MRI for tumour assessment in chest versus abdomen, pelvis and other areas.

- The same imaging method (CT or MRI) as used at Screening must be used at each subsequent disease assessment.
- Scans are performed at screening (see also footnote a), every 8 weeks (±7 days) for the first year and then every 12 weeks (±7 days) in subsequent years, from Cycle 1 Day 1 until disease progression (Section 8.1.2), death, discontinuation from study participation (e.g., patient's request, lost to follow up) or start of subsequent cancer treatment, whichever occurs first.

- **Clinical Evaluation of Superficial Disease** should include photographs of all superficial tumour lesions and should be performed at same intervals as described for CT or MRI scans and timing of clinical evaluations should coincide with the scans (+/-7 days).

o. **Radionuclide Bone Scans, Whole Body** should be performed at Screening. See also footnote a.
- If at Screening bone lesions were identified, scans will be repeated during the active treatment phase and during follow-up visits every 16 weeks (± 7 days) from Cycle 1 Day 1, and at the time of confirmation of CR.
- If no bone lesions were identified at Screening, bone scans will only be performed when clinically indicated (i.e., patient describes new or worsening bone pain or has increasing alkaline phosphatase level or other signs and symptoms of new/progressing bone metastases) but are required at the time of confirmation of CR. New abnormalities found on subsequent bone scans must be confirmed by CT scan with bone windows or MRI.

p. **Adverse Event Reporting**: Serious Adverse Events (SAEs) must be reported from the time the patient provides informed consent through and including 28 calendar days after the last administration of the study drug. SAEs occurring after the active reporting period has ended should be reported if the investigator becomes aware of them. All SAEs that the investigator believes have at least a reasonable possibility of being related to the study drugs are to be reported to the Sponsor. All AEs (serious and non-serious) should be recorded on the eCRF from the first dose of study treatment through last patient visit. It is expected that telephone contact with the patient will be made to assess SAEs and AEs 28 calendar days (±7 days) after the last administration of the study drug.

q. **Patient Feedback**: All patients at the time of discontinuing from the study will be given the opportunity to complete a brief questionnaire to provide feedback relating to their trial participation. Completion of this questionnaire is optional. A sub-set of patients who express interest may be given the opportunity to participate in a face-to-face interview.

r. **Pharmacokinetics (PK)**:
- Blood samples for trough concentrations of CT7001[redacted] will be collected before dosing on Day 1 and 15 of Cycles 1, Day 1 in Cycles 2 and 3, Day 1 in every subsequent odd Cycle and at the end of treatment visit.
- Blood samples for trough concentrations of fulvestrant will be collected before injection of fulvestrant on Days 1 and Day 15 of Cycle 1, Day 1 in Cycles 2 and 3, Day 1 in every subsequent odd Cycle and at the end of treatment visit.

s. **CYP2D6 Polymorphisms**: A blood sample should be collected pre-dose on Day 1 of Cycle 1 for genotyping of CYP2D6 allelic variants and copy number change. As CYP2D6 is the main P450 enzyme involved in hepatic Phase I metabolism of CT7001, this is considered a mandatory study procedure.

t. [redacted]

u. **CT7001**[redacted]: CT7001[redacted] will be dispensed by an IXRS system. Patients will be required to return all bottles of CT7001[redacted] as well as the completed patient diary on Day1 of each cycle for drug accountability.

v. **Fulvestrant**: Fulvestrant will be dispensed by an IXRS system. To be administered on-site as two consecutive slow intramuscular (IM) injections (1-2 minutes) of 250 mg in 5 mL, one in each buttock (gluteal area). Fulvestrant will be dosed at 500 mg given at intervals of 28 ± 2 days, with an additional 500 mg given 14 ± 2 days after the first dose.

w. **Optional Procedures and Assessments** require specific **Informed Consent**, and this separately for (1) ctDNA, WBC isolation, (2) retained sample for exploratory research, (3) tumour biopsies, (4) archival tumour material, and (5) retention of remaining material from the blood sample collected and prepared for genotyping of CYP and drug transporter genes and potential use for additional future pharmacogenomic research .

x. **ctDNA**: A blood sample for ctDNA should be collected at screening, pre-dose on Day 1 of Cycles 1, 2 and 3, Day 1 in every other subsequent odd Cycle and within 2 weeks after disease progression.

y. **Pharmacogenomics**: A blood sample for pharmacogenomic analysis should be collected pre-dose on Day 1 Cycle 1.

z. **Exploratory Research:** A blood sample for potential future exploratory research should be collected pre-dose on Day 1 of Cycles 1, 2 and 3, Day 1 in every other subsequent odd Cycle and within 2 weeks after disease progression.

aa. **Tumour Biopsy**: In patients with readily accessible lesions who have provided consent, a biopsy should be obtained within 10 days before allocation to study therapy and as much as feasible on Day 15 ± 7 days of Cycle 2 and within two weeks after disease progression. Priority is given to formalin-fixed material for immunohistochemistry (including but not limited to CDK7, pPolII, c-Myc, pCDK1 and Ki67/Caspase). In case the biopsy is of sufficient size, remaining material should be fresh-frozen for RNA signature and ChIP-Seq analyses.

bb. **Archival Tumour Material**: Even in case a fresh tumour biopsy can be obtained, an archival formalin-fixed paraffin-embedded tumour tissue sample will be requested (see also Section 8.5.4). It can be collected any time during the active treatment phase but ideally should be obtained during the first 2 cycles.

cc. **Fulvestrant Monotherapy**: These procedures may be omitted for patients who have Sponsor Approval to continue on-study receiving monotherapy Fulvestrant.

dd. Following 12 months of treatment, from Cycle 12 onwards, at the discretion of the investigator and patient, even numbered study visits may be omitted with the exception of fulvestrant administration. Odd numbered study visits must be performed every 56 days +/- 7 days. Where local regulations and procedures allow, Fulvestrant may be administered by a qualified individual at the patients home or by their primary care physician.

ee. **Fasted Glucose**: For patients entering the study with a medical history of diabetes, a fasted glucose test will be required pre-dose at C1D1 and C1D15 (mandatory) and at the remaining cycles at the discretion of the investigator.

[redacted] [redacted]

Table 7: Blood Volumes

Protocol Activity	Screening	Active Treatment Phase - One Cycle = 28 days			End of Treatment/ Withdrawal	Post-Treatment Follow-Up
		Cycles 1 and 2		Cycles ≥3		
Study Day	Within 28 days prior to allocation to study therapy unless specified otherwise	Day 1	Day 15	Day 1		
Visit Window		±2 days	±2 days	±7 days	Within 28 days	
Laboratory Studies						
Haematology [a]	X	Pre-dose	Pre-dose	Pre-dose	X	
Serum Chemistry [b]	X	Pre-dose	Pre-dose	Pre-dose	X	
Pregnancy Test, Serum Oestradiol and FSH (if Applicable) [c]	X					
Other Clinical Assessments						
Pharmacokinetics [d]		Pre-dose	Pre-dose (C1 only)	Pre-dose (every other Cycle)	X	
CYP2D6 Polymorphisms [e]		Pre-dose (C1 only)				
Optional Procedures and Assessments						
ctDNA [f]	X	Pre-dose		Pre-dose (every other Cycle)	X (within 2 weeks after disease progression)	
Pharmacogenomics [g]		Pre-dose (C1 only)				
Exploratory Research [h]		Pre-dose		Pre-dose (every other Cycle)	X (within 2 weeks after disease progression)	
Total Volumes	30 mL	47 mL	19 mL	39 mL	39 mL	

a. **Haematology**: 5 ml per sample.

b. **Serum Chemistry:** 10 ml per sample.

c. **Pregnancy Test:** 5 ml per sample.

d. **Pharmacokinetics:** 4 ml per sample.

e. **CYP2D6 Polymorphisms:** 4 ml per sample.

f. **ctDNA**: 10 ml per sample.

g. **Pharmacogenomics**: 4 ml per sample.

h. **Exploratory Research:** 10 ml per sample.

REFERENCES

Ali S *et al*. ICEC0942, a new oral selective inhibitor of the cell cycle and transcriptional regulator CDK7 for the treatment of estrogen receptor positive and negative breast cancer. *Cancer Res*, 2018; 78(4), supplement/P1-10-05 http://cancerres.aacrjournals.org/content/78/4_Supplement/P1-10-05

Amit O *et* al. Blinded independent central review of progression in cancer clinical trials: Results from a meta-analysis. *Eur J Cancer*, 2011; 47:1772–1778

Andre F *et al*. Alpelisib (ALP) + fulvestrant (FUL) for advanced breast cancer (ABC): results of the Phase 3 SOLAR-1 trial. *Ann Oncol*, 2018, 29 suppl 8:LBA3-PR, http://doi.org/10.1093/annonc/mdy424.010

André, F *et al.* LBA18 Overall survival (os) results from SOLAR-1, a phase III study of alpelisib (ALP) + fulvestrant (FUL) for hormone receptor-positive (HR+), human epidermal growth factor receptor 2-negative (HER2–) advanced breast cancer (ABC). *Annals of Oncology,* 2020; 31, S1150-S1151.

Ainscow E *et al*. CT7001: An orally bioavailable CDK7 inhibitor is a potential therapy for breast, small-cell lung and haematological cancers. *American Association of Cancer Research Annual Meeting*, 2018; http://www.abstractsonline.com/pp8/#!/4562/presentation/6442

Bahl A *et al*. Activity of CT7001 an orally bio-available cyclin-dependent kinase 7 selective inhibitor in models of triple negative breast cancer. *San Antonio Breast Cancer Conference*, 2017; https://www.abstracts2view.com/sabcs17/view.php?nu=SABCS17L_109&terms=

Baselga J *et al*. Phase III study of taselisib (GDC-0032) plus fulvestrant versus fulvestrant in patients with estrogen receptor-positive, PIK3CA-mutant, locally advanced or metastatic breast cancer: Primary analysis from SANDPIPER. *ASCO Annual Meeting,* 2018, abstract LBA1006, http://meetinglibrary.asco.org/record/158535/abstract

Baselga J *et al*. Buparlisib plus fulvestrant versus placebo plus fulvestrant in postmenopausal, hormone receptor-positive, HER2-negative, advanced breast cancer (BELLE-2): a randomised, double-blind, placebo-controlled, phase 3 trial, *Lancet Oncol*, 2017; 18(7):904-916. https://doi.org/10.1016/S1470-2045(17)30376-5

Baselga J *et al*. Everolimus in postmenopausal hormone-receptor positive advanced breast cancer. *N Engl J Med*, 2012; 366:520-529

Burstein HJ *et al*. Adjuvant endocrine therapy for women with hormone receptor–positive breast cancer: ASCO Clinical Practice Guideline Focused Update. *J Clin Oncol*, 2016, 34:1689-1701

Cardoso F *et al*. 4th ESO–ESMO International Consensus Guidelines for advanced breast cancer (ABC 4). *Ann Oncol*, 2018; 29:1634–1657

Chen D *et al*, Activation of estrogen receptor alpha by S118 phosphorylation involves a ligand-dependent interaction with TFIIH and participation of CDK7. *Mol Cell*, 2000; 6:127–37

Chen MT *et al*. Comparison of patterns and prognosis among distant metastatic breast cancer patients by age groups: a SEER population based analysis. *Nature Scientific* Reports, 2017; 7:1-8

Chipumuro E *et al*. CDK7 inhibition suppresses super-enhancer linked oncogenic transcription in MYCN-driven cancer. *Cell*, 2014; 159:1126–39

Clark K *et* al. CT7001, a novel orally bio-available CDK7 inhibitor, is highly active in in-vitro and in-vivo models of AML. *American Society of Hematology*, 2017; http://www.bloodjournal.org/content/130/Suppl_1/2645

Cristofanilli M *et al*. Fulvestrant plus palbociclib versus fulvestrant plus placebo for treatment of hormone-receptor-positive, HER2-negative metastatic breast cancer that progressed on previous endocrine therapy (PALOMA-3): final analysis of the multicentre, double-blind, phase 3 randomised controlled trial. *Lancet Oncol*, 2016; 17:425–39

Dickler *et al*. MONARCH 1, a phase II study of abemaciclib, a CDK4 and CDK6 inhibitor, as a single agent, in patients with refractory HR+/HER2- metastatic breast cancer. *Clin Cancer Res*, 2017, 23:5218-5224

Di Leo A *et* al. Results of the CONFIRM Phase III trial comparing fulvestrant 250 mg with fulvestrant 500 mg in postmenopausal women with estrogen receptor–positive advanced breast cancer. *J Clin Oncol*, 2010; 28:4594-4600

Feaver WJ *et al*. Relationship of CDK-activating kinase and RNA polymerase II CTD kinase TFIIH/TFIIK. *Cell*, 1994; 79:1103–1109

Finn RS *et al*. Palbociclib and letrozole in advanced breast cancer. *N Engl J Med*, 2016; 375:1925-1936

Fisher RP. Secrets of a double agent: CDK7 in cell-cycle control and transcription. *J Cell Sci*, 2005; 118:5171–80

Fisher RP, Morgan DO. A novel cyclin associates with MO15/CDK7 to form the CDK-activating kinase. *Cell*, 1994; 78:713–724

Giordano SH *et al*. Systemic therapy for patients with advanced human epidermal growth factor receptor 2–positive breast cancer: ASCO Clinical Practice Guideline Update. *J Clin Oncol*, 2018; 36:2736-2740

Glover-Cutter K *et al*. TFIIH-associated Cdk7 kinase functions in phosphorylation of C-terminal domain Ser7 residues, promoter-proximal pausing, and termination by RNA polymerase II. *Molecular and cellular biology*, 2009; 29:5455-5464

Goetz MP *et al*. MONARCH 3: Abemaciclib as initial therapy for advanced breast cancer. *J Clin Oncol*, 2017; 35:3638-3646

Guarducci *et al*. Inhibition of CDK7 overcomes resistance to CDK4/6 inhibitors in hormone receptor positive breast cancer cells. *San Antonio Breast Cancer Symposium*, 2018; PD7-12, https://d1io3yog0oux5.cloudfront.net/_8c6f8d424146f31610528aede7b2f364/syros/db/353/2109/pdf/SABCS+2018+Final+Poster.pdf

Hammond MEH *et al.* American Society of Clinical Oncology/College of American Pathologists guideline recommendations for immunohistochemical testing of estrogen and progesterone receptors in breast cancer. *Arch Pathol Lab Med*, 2010; 134:907-922

He, M. *et al.* Metastatic breast cancer patients with lung or liver metastases should be distinguished before being treated with fulvestrant. *Cancer Medicine*, 2019; 8(14), 6212-6220.

Hortobagyi GN *et al*. Ribociclib as rirst-line therapy for HR-positive, advanced breast cancer. *N Engl J Med*, 2016; 375:1738-1748

Howell A *et al*. Fulvestrant, formerly ICI 182,780, is as effective as anastrozole in postmenopausal women with advanced breast cancer progressing after prior endocrine treatment. *J Clin Oncol*, 2002; 20:3396-3403

Johnston S *et al*. Lapatinib combined with letrozole versus letrozole and placebo as first-line therapy for postmenopausal hormone receptor–positive metastatic breast cancer. *J Clin Oncol*, 2009; 27:5538-5546

Juric, D., *et al.* Abstract GS3-08: Alpelisib + fulvestrant for advanced breast cancer: Subgroup analyses from the phase III SOLAR-1 trial. *General Session Abstracts,* 2019; 79(4), GS3-08Kaufman B *et al*. Trastuzumab plus anastrozole versus anastrozole alone for the treatment of postmenopausal women with human epidermal growth factor receptor 2–positive, hormone receptor–positive metastatic breast cancer: Results from the randomized phase III TAnDEM study. *J Clin Oncol*, 2009; 27:5529-5537

Kornblum N *et al*. Randomized Phase II trial of fulvestrant plus everolimus or placebo in postmenopausal women with hormone receptor–positive, human epidermal growth factor receptor 2–negative metastatic breast cancer resistant to aromatase inhibitor therapy: Results of PrE0102. *J Clin Oncol*, 2018; 36:1556-156

Krop IE *et al*. Pictilisib for oestrogen receptor-positive, aromatase inhibitor-resistant, advanced or metastatic breast cancer (FERGI): a randomized, double-blind, placebo-controlled, phase 2 trial. *Lancet Oncol*, 2016; 17:811-821

Kuter *et al*. Pharmacokinetic profile of fulvestrant 500 mg vs 250 mg: Results from the NEWEST study. *J Clin* Oncol, 2008; 26: abstr 579

Kwiatkowski N *et al*. Targeting transcription regulation in cancer with a covalent CDK7 inhibitor. *Nature*, 2014; 511:616–620

Li B *et al*. Therapeutic rationale to target highly expressed CDK7 conferring poor outcomes in triple-negative breast cancer. *Cancer Res*, 2017; 77:3834-3845

Loi S *et al*. PIK3CA mutations associated with gene signature of low mTORC1 signaling and better outcomes in estrogen receptor-positive breast cancer. *Proc Natl Acad Sci USA*, 2010; 107:10208–10213

Osborne CK *et al*. Double-blind, randomized trial comparing the efficacy and tolerability of fulvestrant versus anastrazole in postmenopausal women with advanced breast cancer progressing on prior endocrine therapy: results of a North American trial. *J Clin Oncol*, 2002; 20:3386-3395

Patel H *et al*. ICEC0942, an orally bioavailable selective inhibitor of CDK7 for cancer treatment. *Mol Cancer Ther.* 2018 Jun;17(6):1156-1166

Piccart M *et al*. Everolimus plus exemestane for hormonereceptor-positive, human epidermal growth factor receptor-2-negative advanced breast cancer: overall survival results from BOLERO-2. *Ann Oncol*, 2014; 25:2357–2362

Prescribing information:

Goserelin (Zoladex®): http://www1.astrazeneca-us.com/pi/zoladex3_6.pdf

Lupron®
https://www.accessdata.fda.gov/drugsatfda_docs/label/2014/020517s036_019732s041lbl.pdf

Eliguard®
https://www.accessdata.fda.gov/drugsatfda_docs/label/2011/021343s019,021379s015,021488s016,021731s012lbl.pdf

Trelstar®
https://www.accessdata.fda.gov/drugsatfda_docs/label/2011/020715s024,021288s021,022437s002lbl.pdf

Vantas®http://www.endo.com/File%20Library/Products/Prescribing%20Information/Vantas_prescribing_information.html

Supprelin® https://www.accessdata.fda.gov/drugsatfda_docs/label/2011/022058s006lbl.pdf

https://www.supprelinla.com/

Decapeptyl® : https://www.medicines.org.uk/emc/search?q="Decapeptyl"

Gonapeptyl®: https://www.medicines.org.uk/emc/product/2229

Rasool RU, Natesan R, Deng Q, *et al.* CDK7 Inhibition Suppresses Castration-Resistant Prostate Cancer through MED1 Inactivation. Cancer Discov. 2019;9(11):1538-1555. doi:10.1158/2159-8290.CD-19-0189

Robertson JFR *et* al. Fulvestrant 500 mg versus anastrozole 1 mg for hormone receptor-positive advanced breast cancer (FALCON): an international, randomised, double-blind, phase 3 trial. *Lancet*, 2016; 388:2997–3005

Rubin LG *et al*. IDSA Clinical practice guideline for vaccination of the immunocompromised host. *Clinical Infectious Diseases*, 2014; 58(3):e44–100

Rubinstein LV *et al*. Design issues of randomized Phase II trials and a proposal for Phase II screening trials. J Clin Oncol 2005; 23(28):7199-7206

Rugo HS *et* al. Endocrine therapy for hormone receptor–positive metastatic breast cancer: American Society of Clinical Oncology Guideline. *J Clin Oncol*, 2016; 34:3069-3103

Russnes *et al*. Breast cancer molecular stratification. From intrinsic subtypes to integrative clusters. *Am J Pathol*, 2017; 187:2152-2162

Schachter MM, Fisher RP. The CDK-activating kinase Cdk7: taking yes for an answer. *Cell Cycle*, 2013; 12:3239–3240

Schachter MM *et al*. A Cdk7-Cdk4 T-loop phosphorylation cascade promotes G1 progression. *Mol Cell*, 2013; 50:250–260

Slamon DJ *et al*. Phase III randomized study of ribociclib and fulvestrant in hormone receptor–positive, human epidermal growth factor receptor 2–negative advanced breast cancer: MONALEESA-3. *J Clin Oncol*, 2018; 36:2465-2472

Sledge GW *et al*. MONARCH 2: Abemaciclib in combination with fulvestrant in women with HR+/HER2− advanced breast cancer who had progressed while receiving endocrine therapy. *J Clin Oncol*, 2017; 35:2875-2884

Tripathy D *et al*. Ribociclib plus endocrine therapy for premenopausal women with hormone-receptor-positive, advanced breast cancer (MONALEESA-7): a randomised phase 3 trial. *Lancet*, 2018; 19:904-915

Turner NC *et al*. Palbociclib in hormone-receptor–positive advanced breast cancer. *N Engl J Med*, 2015; 373:209-219

Turner NC *et al*. Overall survival with palbociclib and fulvestrant in advanced breast cancer. *N Engl J Med*, 2018; 379:1926-1936

Wang Y *et al*. 2015 CDK7-dependent transcriptional addiction in triple-negative breast cancer. *Cell,* 2015, Sep 24;163(1):174-186

Wolff AC *et* al. Human epidermal growth factor receptor 2 testing in breast cancer: American Society of Clinical Oncology/College of American Pathologists Clinical practice guideline focused update. *J Clin Oncol*, 2018; 36:2105-2122

Wölwer CB *et al.* A Chemical Screening Approach to Identify Novel Key Mediators of Erythroid Enucleation. *PLoS One*, 2015, Nov 16; 10(11):e0142655

Zhang J *et al*. Systematic bias between blinded independent central review and local assessment: literature review and analyses of 76 phase III randomised controlled trials in 45 688 patients with advanced solid tumour. *BMJ Open*, 2018; 8:e017240. doi:10.1136/ bmjopen-2017-017240

APPENDIX A – DRUGS KNOWN TO PREDISPOSE TO TORSADE DE POINTES

Generic Name	Brand Name(s)
Amiodarone	Cordarone®, Pacerone®
Arsenic trioxide	Trisenox®
Astemizole	Hismanal®
Azithromycin	Zithromax®
Bepridil	Vascor®
Chloroquine	Aralen®
Chlorpromazine	Thorazine®
Cisapride	Propulsid®
Citalopram	Celexa®
Clarithromycin	Biaxin®
Disopyramide	Norpace®
Dofetilide	Tikosyn®
Domperidone	Motilium®
Droperidol	Inapsine®
Erythromycin	Erythrocin®, E.E.S. ®
Flecainide	Tambocor®
Halofantrine	Halfan®
Haloperidol	Haldol®
Ibutilide	Corvert®
Levomethadyl	Orlaam®
Mesoridazine	Serentil®
Methadone	Dolophine®, Methadose®
Moxifloxacin	Avelox®
Ondansetron*	Zofran®
Pentamidine	Pentam®, NebuPent®
Pimozide	Orap®
Probucol	Lorelco®
Procainamide	Pronestyl®, Procan®
Quinidine	Cardioquin®, Quinaglute®
Sotalol	Betapace®
Sparfloxacin	Zagam®
Terfenadine	Seldane®
Thioridazine	Mellaril®
Vandetanib	Caprelsa®

* when administered intravenously at high dose (32 mg)

Adapted from the University of Arizona Cancer Center for Education and Research on Therapeutics: "Torsades List: Drugs with a Risk of Torsades de Pointes," drugs that are generally accepted by the QTdrugs.org Advisory Board to carry a risk of Torsades de Pointes on the University of Arizona CERT website: http://www.crediblemeds.org/.

This list is not meant to be considered all inclusive. See website for current list.

APPENDIX B - MEDICATIONS, HERBAL SUPPLEMENTS, AND FOODS THAT SIGNIFICANTLY INDUCE OR INHIBIT CYTOCHROME P450 3A4, 2C19 AND/OR 2D6 OR P GLYCOPROTEIN ACTIVITY

Strong CYP3A4, CYP2C19 and/or CYP2D6 Inhibitors

The drugs in Table B-1 are known to strongly inhibit CYP3A4, CYP2C19 and/or CYP2D6 and should be avoided while administering CT7001 (FDA Drug Development and Drug Interactions: Table of Substrates, Inhibitors and Inducers, and the Flockhart Table, Indiana University School of Medicine). The list is not exhaustive, and a similar restriction will apply to other agents that are known to strongly modulate CYP3A4, CYP2C19 and/or CYP2D6. Medical judgement is required but **please be vigilant for signs and/or changes in tolerability particularly with 3A4 substrates.**

Please contact the relevant CRO medical monitor with any queries you have on this issue.

Table B-1: Strong CYP3A4, CYP2C19 and/or CYP2D6 Inhibitors

CYP3A4 Inhibitor	CYP2C19 Inhibitor	CYP2D6 Inhibitor
Clarithromycin,	Fluconazole	Bupropion
Telithromycin,	Fluoxetine	Cinacalcet
Troleandomycin	Fluvoxamine	Fluoxetine
Indinavir,	Ticlopidine	Paroxetine
lopinavir,	Voriconzole	Quinidine
Nelfinavir,		Terbinafine
Ritonavir,		
Saquinavir		
Tipranavir		
Telaprevir		
Itraconazole		
Ketoconazole		
Posaconazole		
Voriconazole		
Suboxone		
Nefadozone		
Boceprivir		
Conivaptan		
Cobicistat		
Danoprevir		
Elvitegravir		
Grapefruit juice		

Table B-1: Strong CYP3A4, CYP2C19 and/or CYP2D6 Inhibitors

CYP3A4 Inhibitor	CYP2C19 Inhibitor	CYP2D6 Inhibitor
Paritaprevir		
Idelalisib		
Diltiazem,		
Nelfinavir		

Strong CYP3A4, CYP2C19 and/or CYP2D6 Inducers

The drugs in Table B-2 are known to strongly induce CYP3A4, CYP2C19 and/or CYP2D6 and should be avoided while administering CT7001 (FDA Drug Development and Drug Interactions: Table of Substrates, Inhibitors and Inducers, and the Flockhart Table, Indiana University School of Medicine). The list is not exhaustive, and a similar restriction will apply to other agents that are known to strongly induce CYP3A4, CYP2C19 and/or CYP2D6. Medical judgement is required.

Please contact the relevant CRO medical monitor with any queries you have on this issue.

Table B-2: Strong CYP3A4, CYP2C19 and/or CYP2D6 Inducers

CYP3A4 Inducer	CYP2C19 Inducer	CYP2D6 Inducer
Carbamazepine	Aprepitant	None known
Enzalutamide	Carbamazepine	
Mitotane,	Enzalutamide	
Phenytoin	Rifampin	
Rifampin	Ritonavir	
St. John's wort	Nevirapine	
Phenobarbital	Phentobarbital	
Rifabutin	St John's Wort	
Nevirapine		
Troglitazone		

Drugs whose clearance is dependent on CYP3A4 and have a narrow therapeutic index

There are currently no data confirming that there is a PK interaction between CT7001 and other drugs. However, *in vitro* data suggests CT7001 has the potential to cause drug interactions at the intestinal and hepatic level through CYP3A4.

CT7001 shows a weak potential to inhibit CYP2D6 and 2C19 which is unlikely to be clinically significant but should be considered when co-prescribing sensitive 2D6 and 2C19 substrates and substrates with narrow therapeutic index (e.g. S-mephenytoin). The potential for CT7001 to inhibit transporter systems is currently unknown.

If CT7001 is co-administered with CYP3A substrates with narrow therapeutic indices, including but not limited to alfentanil, cisapride, cyclosporine, ergot derivatives, fentanyl, pimozide, quinidine, sirolimus, tacrolimus, atorvastatin, lovastatin and simvastatin the subject should be closely monitored for signs of changed tolerability as a result of increased exposure of the concomitant medication. This list is not intended to be exhaustive, and similar precautions should be applied to other agents that are known to depend on CYP3A4 for metabolism.

Medical judgement is required. Please contact the relevant CRO medical monitor with any queries you have on this issue.

P-Glycoprotein (PGP) Inhibitors and Inducers

The drugs in Table B-3 are known to strongly inhibit or induce P-Glycoprotein (PGP) and should be avoided while administering CT7001 (FDA Drug Development and Drug Interactions: Table of Substrates, Inhibitors and Inducers, and the Flockhart Table, Indiana University School of Medicine). The list is not exhaustive, and a similar restriction will apply to other agents that are known to strongly induce PGP. Medical judgement is required.

Please contact the relevant CRO medical monitor with any queries you have on this issue.

Table B-3: P-Glycoprotein (PGP) Inhibitors and Inducers

PGP Inhibitors	PGP Inducers
Amiodarone	Avasimibe
Carvedilol	Carbamazepine
Clarithromycin	Phenytoin
Dronedarone	Rifampin
Itraconazole	Ritonavir
Lapatinib	St. John's Wort
Lovinavir	Tipranavir
Ritonavir	
Propafenone	
Quinidine	
Ranolazine	
Ritonavir	
Saquinavir	
Telaprivir	
Tipranavir	
Verapamil	

APPENDIX C – RESPONSE EVALUATION CRITERIA IN SOLID TUMOURS (RECIST) VERSION 1.1

Adapted from E.A. Eisenhauer, et al: New response evaluation criteria in solid tumours: Revised RECIST guideline (version 1.1). European Journal of Cancer 45 (2009) 228–247

CATEGORIZING LESIONS AT BASELINE

Measurable Lesions

Lesions that can be accurately measured in at least one dimension.

- Lesions with longest diameter twice the slice thickness and at least 10 mm or greater when assessed by CT or MRI (slice thickness 5-8 mm).
- Lesions with longest diameter at least 20 mm when assessed by Chest X-ray.
- Superficial lesions with longest diameter 10 mm or greater when assessed by calliper.
- Malignant lymph nodes with the short axis 15 mm or greater when assessed by CT.

NOTE: The shortest axis is used as the diameter for malignant lymph nodes, longest axis for all other measurable lesions.

Non-Measurable Disease

Non-measurable disease includes lesions too small to be considered measurable (including nodes with short axis between 10 and 14.9 mm) and truly non-measurable disease such as pleural or pericardial effusions, ascites, inflammatory breast disease, leptomeningeal disease, lymphangitic involvement of skin or lung, clinical lesions that cannot be accurately measured with callipers, abdominal masses identified by physical examination that are not measurable by reproducible imaging techniques.

- Bone disease: Bone disease is non-measurable with the exception of soft tissue components that can be evaluated by CT or MRI and meet the definition of measurability at baseline.
- Previous local treatment: A previously irradiated lesion (or lesion subjected to other local treatment) is non-measurable unless it has progressed since completion of treatment.

Normal Sites

- Cystic lesions: Simple cysts should not be considered as malignant lesions and should not be recorded either as target or non-target disease. Cystic lesions thought to represent cystic metastases can be measurable lesions if they meet the specific definition above. If non-cystic lesions are also present, these are preferred as target lesions.
- Normal nodes: Nodes with short axis <10 mm are considered normal and should not be recorded or followed either as measurable or non-measurable disease.

RECORDING TUMOUR ASSESSMENTS

All sites of disease must be assessed at baseline. Baseline assessments should be done as close as possible prior to study start. For an adequate baseline assessment, all required scans must be

done within 28 days prior to treatment and all disease must be documented appropriately. If baseline assessment is inadequate, subsequent statuses generally should be indeterminate.

Target Lesions

All measurable lesions up to a maximum of 2 lesions per organ, 5 lesions in total, representative of all involved organs, should be identified as target lesions at baseline. Target lesions should be selected on the basis of size (longest lesions) and suitability for accurate repeated measurements. Record the longest diameter for each lesion, except in the case of pathological lymph nodes for which the short axis should be recorded. The sum of the diameters (longest for non-nodal lesions, short axis for nodal lesions) for all target lesions at baseline will be the basis for comparison to assessments performed on study.

- If two target lesions coalesce the measurement of the coalesced mass is used. If a large target lesion splits, the sum of the parts is used.
- Measurements for target lesions that become small should continue to be recorded. If a target lesion becomes too small to measure, 0 mm should be recorded if the lesion is considered to have disappeared; otherwise a default value of 5 mm should be recorded.

NOTE: When nodal lesions decrease to <10 mm (normal), the actual measurement should still be recorded.

Non-Target Disease

All non-measurable disease is non-target. All measurable lesions not identified as target lesions are also included as non-target disease. Measurements are not required but rather assessments will be expressed as ABSENT, INDETERMINATE, PRESENT/NOT INCREASED, INCREASED. Multiple non-target lesions in one organ may be recorded as a single item on the case report form (e.g. ‘multiple enlarged pelvic lymph nodes’ or ‘multiple liver metastases’).

OBJECTIVE RESPONSE STATUS AT EACH EVALUATION

Disease sites must be assessed using the same technique as baseline, including consistent administration of contrast and timing of scanning. If a change needs to be made the case must be discussed with the radiologist to determine if substitution is possible. If not, subsequent objective statuses are indeterminate.

Target Disease

- Complete Response (CR): Complete disappearance of all target lesions with the exception of nodal disease. All target nodes must decrease to normal size (short axis < 10 mm). All target lesions must be assessed.
- Partial Response (PR): Greater than or equal to 30% decrease under baseline of the sum of diameters of all target measurable lesions. The short diameter is used in the sum for target nodes, while the longest diameter is used in the sum for all other target lesions. All target lesions must be assessed.
- Stable Disease (SD): Does not qualify for CR, PR or Progression. All target lesions must be assessed. Stable can follow PR only in the rare case that the sum increases by

less than 20% from the nadir, but enough that a previously documented 30% decrease no longer holds.

- Progressive Disease (PD): 20% increase in the sum of diameters of target measurable lesions above the smallest sum observed (over baseline if no decrease in the sum is observed during therapy), with a minimum absolute increase of 5 mm.
- Indeterminate. Progression has not been documented, and:
  - One or more target measurable lesions have not been assessed.
  - Or assessment methods used were inconsistent with those used at baseline.
  - Or one or more target lesions cannot be measured accurately (e.g., poorly visible unless due to being too small to measure).
  - Or one or more target lesions were excised or irradiated and have not reappeared or increased.

Non-Target Disease

- CR: Disappearance of all non-target lesions and normalization of tumour marker levels. All lymph nodes must be 'normal' in size (<10 mm short axis).
- Non-CR/Non-PD: Persistence of any non-target lesions and/or tumour marker level above the normal limits.
- PD: Unequivocal progression of pre-existing lesions. Generally, the overall tumour burden must increase sufficiently to merit discontinuation of therapy. In the presence of SD or PR in target disease, progression due to unequivocal increase in non-target disease should be rare.
- Indeterminate: Progression has not been determined and one or more non-target sites were not assessed, or assessment methods were inconsistent with those used at baseline.

New Lesions

The appearance of any new unequivocal malignant lesion indicates PD. If a new lesion is equivocal, for example due to its small size, continued assessment will clarify the aetiology. If repeat assessments confirm the lesion, then progression should be recorded on the date of the initial assessment. A lesion identified in an area not previously scanned will be considered a new lesion.

Supplemental Investigations

- If CR determination depends on a residual lesion that decreased in size but did not disappear completely, it is recommended the residual lesion be investigated with biopsy or fine needle aspirate. If no disease is identified, objective status is CR.
- If progression determination depends on a lesion with an increase possibly due to necrosis, the lesion may be investigated with biopsy or fine needle aspirate to clarify status.

Subjective Progression

Patients requiring discontinuation of treatment without objective evidence of disease progression should not be reported as PD on tumour assessment eCRFs. This should be indicated on the end of treatment eCRF as off treatment due to Global Deterioration of Health

Status. Every effort should be made to document objective progression even after discontinuation of treatment.

Table C-1. Objective Response Status at Each Evaluation

Target Lesions	Non-Target Disease	New Lesions	Objective Status
CR	CR	No	CR
CR	Non-CR/Non-PD, Indeterminate or Missing	No	PR
PR	Non-CR/Non-PD, Indeterminate or Missing	No	PR
SD	Non-CR/Non-PD, Indeterminate or Missing	No	SD
Indeterminate or Missing	Non-PD	No	Indeterminate
PD	Any	Yes or No	PD
Any	PD	Yes or No	PD
Any	Any	Yes	PD

If the protocol allows enrolment of patients with only non-target disease, the following table will be used:

Table C-2. Objective Response Status at Each Evaluation for Patients with Non-Target Disease Only

Non-Target Disease	New Lesions	Objective Status
CR	No	CR
Non-CR/Non-PD	No	Non-CR/Non-PD
Indeterminate	No	Indeterminate
Unequivocal progression	Yes or No	PD
Any	Yes	PD

The best overall response (BOR) is the best response recorded from the randomization until disease progression or death due to any cause. This is derived from the sequence of objective statuses. Objective statuses are not considered after objective progression is documented or after start of the first anticancer treatment post discontinuation of protocol treatment. BOR for each patient will be derived as one of the following categories:

- **Complete response (CR)**: At least one objective status of CR documented before progression.
- **Partial response (PR)**: At least one objective status of PR documented before progression.

- **Stable disease (SD)**: At least one objective status of stable documented at least 8 weeks after randomization date and before progression but not qualifying as CR, PR.
- **Progressive Disease (PD)**: Objective status of progression within 16 weeks of randomization, not qualifying as CR, PR or SD.
- **Indeterminate (IND)**: Progression not documented within 16weeks after randomization and no other response category applies.

APPENDIX D – NEW YORK HEART ASSOCIATION (NYHA) FUNCTIONAL CLASSIFICATION

classification_nyha_n
ew_york_heart_associ

APPENDIX E – US FDA LABEL, EU SPC AND EPAR ASSESSMENT REPORT OF FULVESTRANT

Faslodex Label FDA.pdf

Faslodex 250 mg solution for injection -

faslodex-epar-assessment-report-variation

APPENDIX F – EASTERN COOPERATIVE ONCOLOGY GROUP (ECOG) PERFORMANCE STATUS

ECOG PERFORMANCE STATUS*	
**Grade**	**ECOG**
0	Fully active, able to carry on all pre-disease performance without restriction
1	Restricted in physically strenuous activity but ambulatory and able to carry out work of a light or sedentary nature, e.g., light house work, office work
2	Ambulatory and capable of all selfcare but unable to carry out any work activities. Up and about more than 50% of waking hours
3	Capable of only limited selfcare, confined to bed or chair more than 50% of waking hours
4	Completely disabled. Cannot carry on any selfcare. Totally confined to bed or chair
5	Dead

* As published in Am. J. Clin. Oncol.:
Oken, M.M., Creech, R.H., Tormey, D.C., Horton, J., Davis, T.E., McFadden, E.T., Carbone, P.P.: Toxicity And Response Criteria Of The Eastern Cooperative Oncology Group. Am J Clin Oncol 5:649-655, 1982.

APPENDIX G – MANAGEMENT OF THE CT7001_001 STUDY THROUGHOUT COVID-19 PANDEMIC.

Introduction

In early 2020 the global pandemic, COVID-19, impacted the conduct of clinical trials throughout the world. Sponsors and Investigators had to evaluate the ongoing risk to patients enrolled on clinical trials and put in place adequate measures to protect patient safety whilst, maintaining compliance with good clinical practice (GCP), and minimising loss of integrity to the clinical trial data. This appendix aims to outline the approach that Carrick Therapeutics undertook throughout this pandemic. These measures were initiated on 18th March 2020 and will continue until the COVID-19 risk subsides. All Regulatory Authority Guidance relating to managing clinical trials during COVID-19 issued by FDA, EMA and MHRA are monitored and followed.

- FDA - Guidance on Conduct of Clinical Trials of Medical Products during the COVID-19 Public Health Emergency Guidance for Industry, Investigators, and Institutional Review Boards; March 2020 and updates

- EMA - GUIDANCE ON THE MANAGEMENT OF CLINICAL TRIALS DURING THE COVID-19 (CORONAVIRUS) PANDEMIC; Version 1, 20 March 2020 and updates

- MHRA - Managing clinical trials during Coronavirus (COVID-19) - How investigators and sponsors should manage clinical trials during COVID-19; 19 March 2020 and updates

Risk of COVID-19 infection whilst on CT7001

The non-clinical and clinical safety profile of CT7001 observed to date does not indicate any substantial risk to patients continuing treatment during the COVID-19 situation. For this reason, it was assessed that all patients currently on study were benefitting from treatment and were permitted to continue receiving CT7001.

Recruitment during COVID-19

At a global level, Carrick Therapeutics assessed that limitations imposed by COVID-19 did not pose any new safety risks to trial participants and authorised all relevant modules to remain open to recruitment. However, in accordance with institutional guidance and restrictions at a local level, some Investigators took the decision not to recruit patients into clinical trials during COVID-19.

Some modules/cohorts were cancelled or delayed due to the impact of COVID-19.

No inclusion/exclusion criteria waivers were permitted and patients needed to meet protocol requirements to enter the study.

Module 1A Dose-Escalation: This cohort was complete and not impacted.

Module 1A Breast Cancer Paired Biopsy: This cohort was fully recruited and therefore recruitment was not impacted, patients continued on study during COVID-19.

Module 1A Solid Tumour Paired Biopsy: This cohort was not initiated and was subsequently cancelled. All Investigators in this module had halted recruitment to new clinical trials.

Module 1B-2 (CRPC): This cohort was fully recruited and therefore recruitment was not impacted, patients continued on study during COVID-19.

Module 1B-1 (TNBC): This cohort remained open to recruitment during the onset of COVID-19 but did not enrol and was then subsequently closed to recruitment due to an unrelated reason (Interim Efficacy analysis), patients continued on study during COVID-19.

Module 2A: This cohort remained open to recruitment during the COVID-19 period.

Module 2B/C: This cohort was not initiated during the COVID-19 period and the opening to recruitment has been delayed.

Module 4: This cohort was fully recruited and therefore recruitment was not impacted, patients continued on study during COVID-19.

Modification in patient management throughout COVID-19

As the COVID-19 situation evolved it became evident that Investigators were required to prioritize clinical resource and reduce or cancel any non-essential patient contact.

Where safe and reasonable to do so, Investigators should continue to follow the protocol schedule of events for the relevant module.

However, in the event modification to the visit schedule became necessary, Carrick Therapeutics identified the minimum patient management requirements in order to ensure patient safety throughout COVID-19.

- Any new patient must continue to undergo all mandatory screening and eligibility tests as identified in the protocol schedule of events, no inclusion/exclusion waivers will be granted.
- All mandatory screening and Cycle 1 procedures must be performed for patients newly enrolling.
- All mandatory procedures at Cycle 1 Day 15 and Cycle 2 Day 1 must be performed.
- From Cycle 3 an AE and ConMed assessment must be performed at every visit. This may be by telephone where applicable.
- From Cycle 3 haematology, biochemistry and urinalysis may be performed external to the hospital site.
- From Cycle 3, CT7001 may be dispensed to cover two cycles provided the patient continues to demonstrate compliance >75%.
- From Cycle 6, haematology, biochemistry and urinalysis may be performed at alternate cycles.
- From Cycle 12, haematology, biochemistry and urinalysis may be performed at every third cycle.

- CT, MRI and Bone Scans should continue to be performed at the protocol specified interval for M1B-1 (TNBC), M1B-2 (CRPC) and Module 2.
- For any patient with cardiac history on enrolment or taking concomitant medication that might increase the risk of cardiac symptoms, e.g. amitriptyline then ECGs must be performed according to schedule of events.

Alternative Healthcare Facilities

Carrick Therapeutics authorized the use of alternative healthcare facilities during COVID-19 to perform routine clinical assessments that may include laboratory tests, imaging scans, ECG's. These may be performed, where applicable, by a primary care physician, a private facility or by a home nursing service. Any costs that the hospital or a patient incurs will be reimbursed by the Sponsor. The impact on study data will be assessed and captured in the Clinical Study Report but it is expected to be minimal.

Home Nursing Service

Wren Healthcare Limited was contracted by Carrick Therapeutics to provide a home nursing service in the UK. The service was optional and was offered as an opt-in service for investigators and patients who were consented. This service was also supported by A4P Bio Logistics who coordinated a courier service for the delivery of temperature-controlled safety and biomarker laboratory samples.

IMP Management

A4P Bio Logistics (UK) and Almac (USA) were contracted to provide a temperature-controlled, chain of custody managed, courier collection from the dispensing pharmacy to the patient's home.

Where sites were unable to use these services due to institutional guidelines, methods of IMP delivery to patients were reviewed on a case by case basis.

Site Monitoring

On-site monitoring was not permitted throughout the peak of the COVID-19 outbreak. Monitoring plans were amended to include remote contacts and, where compliant with local regulations, remote data review.

Protocol Deviations

The COVID-19 situation is likely to increase protocol deviations. All COVID-19 related protocol deviations will be captured in the eCRF. An adaption to the database has been completed that will identify protocol deviations related to COVID-19 and these will be assessed and reported in the clinical study report.

APPENDIX H – CT7001_001 DATA MONITORING COMMITTEE NVD GUIDELINES VERSION 2.0 08-JUN-2021

CT7001_001 Data Monitoring Committee NVD Management Instructions
Version 2.0 08-JUN-2021

DATA MONITORING COMMTTEE: MANAGEMENT OF NAUSEA AND/OR VOMITING AND/OR DIARRHOEA

Introduction

CT7001_001 is a modular protocol enrolling patients with advanced solid tumours onto either monotherapy CT7001 or in combination with standard care, as applicable to the module. The first patient was enrolled onto CT7001_001 on 20-Nov-2017 and at the introduction of this guidance document, in excess of 100 patients had received treatment with CT7001; with recruitment ongoing it is anticipated approximately 250 patients will be exposed to CT7001 throughout the lifecycle of the protocol.

Nausea, vomiting and diarrhoea have been very commonly reported by patients as adverse events since the outset. Although these symptoms are primarily mild and able to be managed adequately there is a smaller cohort of patients who have been unable to tolerate treatment with CT7001. Please refer to the current Investigator Brochure for a breakdown of grades, occurrence and outcomes.

The Data Monitoring Committee (DMC) and Safety Review Committee (SRC) continue to monitor nausea, vomiting and diarrhoea closely in an effort to characterise the symptoms and provide guidance to treating physicians and the patients in relation to best management protocols.

This guidance document, supporting protocol CT7001_001, has been produced following clinical input from the CT7001_001 DMC, following consultation with the Experimental Cancer Medicines Centres (ECMC) Research Nurses Network Group and in accordance with National Comprehensive Cancer Network (NCCN) Guidelines.

In parallel, Carrick Therapeutics is planning to investigate the impact of an enteric capsule formulation on the incidence and severity of nausea, vomiting and diarrhoea in a solid tumour population.

The following mitigation steps are required:

1. A full discussion will be held with all newly enrolling patients regarding nausea, vomiting and diarrhoea symptoms and appropriate management.

2. A Nausea, Vomiting and Diarrhoea Information Leaflet is available. A copy will be provided to all new patients at Cycle 1 Day 1.

3. Patients must be given an adequate supply of anti-emetics and anti-diarrhoeal medication to take home with them. They must be instructed to take anti-emetic therapy prophylactically prior to their first dose of CT7001. The patient must also be instructed to initiate anti-diarrhoeal therapy at the first sign of symptoms; there is no need to seek approval from the Investigator. The following are recommended:

    a. Anti-emetic therapy: A serotonin (5-HT3) antagonist (NCCN Antiemesis Guidelines v2;2017).
        i. At the discretion of the Investigator additional anti-emetic e.g. metoclopramide may be given PRN in combination with serotonin (5-HT3) antagonist.

Page **1** of **3**

ii. In the event a patient is unable to tolerate a serotonin (5-HT3) agonist then an alternative anti-emetic may be prescribed e.g. prochlorperazine.

b. Anti-diarrhoeal therapy: loperamide.

4. Anti-emetic therapy must be given prophylactically before all doses, including C1D1, up to first clinic review visit on Day 15 and then continued at the discretion of the Investigator.

5. Anti-diarrhoeal therapy must be initiated immediately on first symptoms.

6. Prophylactic therapy can be considered for withdrawal once a patient is established on treatment. Longer term management is directed by clinical judgement of a patient's symptoms and signs.

7. The following additional steps are also advised:
   - Advise patients to take their CT7001 just prior to bed or rest for approximately 60 minutes after taking CT7001 as this may reduce the severity of nausea.
   - Consumption of a small meal / food approximately 30 minutes prior to taking CT7001 may reduce the severity of nausea.
   - Discuss techniques to help patients manage ongoing stress or anxiety as this has been reported to exacerbate gastrointestinal symptoms.

Patients should be instructed to contact the investigator at the earliest opportunity if they experience symptoms of nausea, vomiting or diarrhoea, even if mild.

1. Patients should also be encouraged to drink plenty of fluid.

2. Investigators should follow up with the patient by telephone to assess response and maintain regular contact to monitor symptoms and to ensure optimal supportive therapy is being received.

3. If N/V is not optimally controlled on anti-emetics alone then consider use of an oral proton pump inhibitor.

4. Dietetic measures to control diarrhoea can be considered: stop all lactose containing products, drink 8 to 10 large glasses of clear liquids per day (fluid intake of ~2L per day should be maintained), eat frequent small meals, recommend low fat diet enriched with banana, rice, apple sauce and toast i.e. BRAT diet.

5. For diarrhoea it is important that patients take a full dose of loperamide; 4mg on first episode of diarrhoea followed by 2mg on each further episode of diarrhoea with a maximum of 16mg over a 24 hour period. Alternative anti-diarrhoeals such as Lomotil, codeine, opium tincture, octreotide etc. can also be considered as secondary therapy if the patient has not responded to loperamide within ~ 48 hours.

6. If symptoms persist for >5 days at Grade 2 or >2 days at Grade 3+ then the Investigator should contact the study medical monitor.

CT7001_001 Data Monitoring Committee NVD Management Instructions
Version 2.0 08-JUN-2021

7. Investigators should consider if patients experiencing anticipatory nausea/psychosomatic symptoms may benefit from short (up to 7 days) treatment breaks as appropriate, especially in the first two cycles, to allow for resolution of symptoms and changes to management protocols prior to reinitiating CT7001 therapy.

8. The protocol also allows for dose reductions of CT7001 to 240mg daily and then 120mg daily

The above information is provided based on the clinical experience with CT7001 to date, at all times the Investigator retains full control of the appropriate medical management of their patient.

It is important to note that Aprepitant (Emend®) is a substrate, moderate inhibitor and inducer of CYP3A4 and an inducer of CYP2C19. Therefore, aprepitant should not be used in this study for the treatment of nausea and vomiting.

DocuSign

Certificate Of Completion

Envelope Id: 31190CC7C56F4F71A7B69671A5529E1B

Status: Completed

Subject: Please DocuSign: CT7001_001_Module 2_Protocol_v9.0_14Jun21_Final_Clean.pdf

Source Envelope:

Document Pages: 137

Signatures: 4

Envelope Originator:

Certificate Pages: 5

Initials: 0

[redacted]

AutoNav: Enabled

63-65 Knowl Piece

EnvelopeId Stamping: Disabled

Wilbury Way

Time Zone: (UTC) Dublin, Edinburgh, Lisbon, London

Hitchin, Hertfordshire SG4 0TY

[redacted]

Record Tracking

Status: Original

18-Jun-21 | 16:02

Holder: [redacted]

Location: DocuSign

Signer Events | Signature | Timestamp

[redacted]

Security Level: Email, Account Authentication (Required)

DocuSigned by:

[redacted]

Signing Reason: I approve this document

Signing Time: 21-Jun-2021 | 04:07 MDT

E9BE010FA0D649B891783A4ECE97B3DA

Signature Adoption: Pre-selected Style

Signature ID:

E9BE010F-A0D6-49B8-9178-3A4ECE97B3DA

[redacted]

With Signing Authentication via DocuSign password

With Signing Reasons (on each tab):

I approve this document

Sent: 18-Jun-21 | 16:08

Viewed: 21-Jun-21 | 08:30

Signed: 21-Jun-21 | 11:07

Electronic Record and Signature Disclosure:

Accepted: 21-Jun-21 | 08:30

ID: f0da07de-75ca-4fed-b8f3-8eb52d626936

[redacted]

Senior Medical Director

Security Level: Email, Account Authentication (Required)

[redacted]

Signature Adoption: Pre-selected Style

Signature ID:

94C0D49A-78C7-48F0-9D99-8E746FA969BE

[redacted]

With Signing Authentication via DocuSign password

With Signing Reasons (on each tab):

I approve this document

Sent: 18-Jun-21 | 16:08

Viewed: 21-Jun-21 | 11:41

Signed: 21-Jun-21 | 11:42

Electronic Record and Signature Disclosure:

Accepted: 01-Oct-20 | 12:09

ID: 726a003c-f209-4c5d-91c4-e40caf870c0e

Signer Events	Signature	Timestamp
[redacted]<br>Statistician<br>Security Level: Email, Account Authentication (Required)	[redacted]<br><br>Signature Adoption: Pre-selected Style<br>Signature ID:<br>D27C397C-7756-41A9-A893-60FD282B30C0<br>[redacted]<br><br>With Signing Authentication via DocuSign password<br>With Signing Reasons (on each tab):<br>I approve this document	Sent: 18-Jun-21 | 16:08<br>Viewed: 21-Jun-21 | 10:57<br>Signed: 21-Jun-21 | 10:58

Electronic Record and Signature Disclosure:
Accepted: 06-Jan-21 | 11:53
ID: a1056371-266a-4016-9d5c-cc3242b460d9

[redacted]<br>The Christie NHS Foundation Trust<br>Security Level: Email, Account Authentication (Required)	[redacted]<br><br>Signature Adoption: Pre-selected Style<br>Signature ID:<br>6768291F-C826-4A8E-A783-47C3E990EF4A<br>[redacted]<br><br>With Signing Authentication via DocuSign password<br>With Signing Reasons (on each tab):<br>I approve this document	Sent: 18-Jun-21 | 16:08<br>Resent: 21-Jun-21 | 12:48<br>Resent: 21-Jun-21 | 16:16<br>Viewed: 21-Jun-21 | 16:35<br>Signed: 21-Jun-21 | 16:35

Electronic Record and Signature Disclosure:
Accepted: 21-Jun-21 | 16:35
ID: 8890c413-02d8-47b6-91bc-880ff6a8525d

In Person Signer Events	Signature	Timestamp

Editor Delivery Events	Status	Timestamp

Agent Delivery Events	Status	Timestamp

Intermediary Delivery Events	Status	Timestamp

Certified Delivery Events	Status	Timestamp

Carbon Copy Events	Status	Timestamp

Witness Events	Signature	Timestamp

Notary Events	Signature	Timestamp

Envelope Summary Events	Status	Timestamps
Envelope Sent	Hashed/Encrypted	18-Jun-21 | 16:08
Certified Delivered	Security Checked	21-Jun-21 | 16:35
Signing Complete	Security Checked	21-Jun-21 | 16:35
Completed	Security Checked	21-Jun-21 | 16:35

Payment Events	Status	Timestamps

Electronic Record and Signature Disclosure

ELECTRONIC RECORD AND SIGNATURE DISCLOSURE

From time to time, Bionical Emas (we, us or Company) may be required by law to provide to you certain written notices or disclosures. Described below are the terms and conditions for providing to you such notices and disclosures electronically through the DocuSign system. Please read the information below carefully and thoroughly, and if you can access this information electronically to your satisfaction and agree to this Electronic Record and Signature Disclosure (ERSD), please confirm your agreement by selecting the check-box next to 'I agree to use electronic records and signatures' before clicking 'CONTINUE' within the DocuSign system.

Getting paper copies

At any time, you may request from us a paper copy of any record provided or made available electronically to you by us. You will have the ability to download and print documents we send to you through the DocuSign system during and immediately after the signing session and, if you elect to create a DocuSign account, you may access the documents for a limited period of time (usually 30 days) after such documents are first sent to you. After such time, if you wish for us to send you paper copies of any such documents from our office to you, you will be charged a $0.00 per-page fee. You may request delivery of such paper copies from us by following the procedure described below.

Withdrawing your consent

If you decide to receive notices and disclosures from us electronically, you may at any time change your mind and tell us that thereafter you want to receive required notices and disclosures only in paper format. How you must inform us of your decision to receive future notices and disclosure in paper format and withdraw your consent to receive notices and disclosures electronically is described below.

Consequences of changing your mind

If you elect to receive required notices and disclosures only in paper format, it will slow the speed at which we can complete certain steps in transactions with you and delivering services to you because we will need first to send the required notices or disclosures to you in paper format, and then wait until we receive back from you your acknowledgment of your receipt of such paper notices or disclosures. Further, you will no longer be able to use the DocuSign system to receive required notices and consents electronically from us or to sign electronically documents from us.

All notices and disclosures will be sent to you electronically

Unless you tell us otherwise in accordance with the procedures described herein, we will provide electronically to you through the DocuSign system all required notices, disclosures, authorizations, acknowledgements, and other documents that are required to be provided or made available to you during the course of our relationship with you. To reduce the chance of you inadvertently not receiving any notice or disclosure, we prefer to provide all of the required notices and disclosures to you by the same method and to the same address that you have given us. Thus, you can receive all the disclosures and notices electronically or in paper format through the paper mail delivery system. If you do not agree with this process, please let us know as described below. Please also see the paragraph immediately above that describes the consequences of your electing not to receive delivery of the notices and disclosures electronically from us.

How to contact Bionical Emas:

You may contact us to let us know of your changes as to how we may contact you electronically, to request paper copies of certain information from us, and to withdraw your prior consent to receive notices and disclosures electronically as follows:
To contact us by email send messages to: DISTDPO@Bionical-Emas.com
To contact us by paper mail, please send correspondence to:
Bionical Emas
63-65 Knowl Piece
Wilbury Way
Hitchin, Hertfordshire, United Kingdom, SG4 0TY

To advise Bionical Emas of your new email address

To let us know of a change in your email address where we should send notices and disclosures electronically to you, you must send an email message to us at DISTDPO@Bionical-Emas.com and in the body of such request you must state: your previous email address, your new email address. We do not require any other information from you to change your email address.

If you created a DocuSign account, you may update it with your new email address through your account preferences.

To request paper copies from Bionical Emas

To request delivery from us of paper copies of the notices and disclosures previously provided by us to you electronically, you must send us an email to DISPDPO@Bionical-emas.com and in the body of such request you must state your email address, full name, mailing address, and telephone number. We will discuss any anticipated fees and how these are collected with you.

To withdraw your consent with Bionical Emas

To inform us that you no longer wish to receive future notices and disclosures in electronic format you may:

i. decline to sign a document from within your signing session, and on the subsequent page, select the check-box indicating you wish to withdraw your consent, or you may;

ii. send us an email to DISTDPO@Bionical-Emas.com and in the body of such request you must state your email, full name, mailing address, and telephone number. We do not need any other information from you to withdraw consent.. The consequences of your withdrawing consent will be discussed with you..

Required hardware and software

The minimum system requirements for using the DocuSign system may change over time. The current system requirements are found here: https://support.docusign.com/guides/signer-guide-signing-system-requirements.

Acknowledging your access and consent to receive and sign documents electronically

To confirm to us that you can access this information electronically, which will be similar to other electronic notices and disclosures that we will provide to you, please confirm that you have read this ERSD, and (i) that you are able to print on paper or electronically save this ERSD for your future reference and access; or (ii) that you are able to email this ERSD to an email address where you will be able to print on paper or save it for your future reference and access. Further, if you consent to receiving notices and disclosures exclusively in electronic format as described herein, then select the check-box next to 'I agree to use electronic records and signatures' before clicking 'CONTINUE' within the DocuSign system.

By selecting the check-box next to 'I agree to use electronic records and signatures', you confirm that:

- You can access and read this Electronic Record and Signature Disclosure; and
- You can print on paper this Electronic Record and Signature Disclosure, or save or send this Electronic Record and Disclosure to a location where you can print it, for future reference and access; and
- Until or unless you notify Bionical Emas as described above, you consent to receive exclusively through electronic means all notices, disclosures, authorizations, acknowledgements, and other documents that are required to be provided or made available to you by Bionical Emas during the course of your relationship with Bionical Emas.

CT7001_001 MODULE 1A STATISTICAL ANALYSIS PLAN

Title: Statistical Analysis Plan for CT7001_001 Module 1 Part A

Compound Name/Number: CT7001

Effective Date: 08-Oct-2020

Description: Initial evaluation of monotherapy (single and multiple dose) to identify the CT7001 minimally biologically active dose (MBAD) and maximum tolerated dose (MTD) in patients with advanced solid malignancies.

Authors' Names, Titles and Functional Areas: [redacted] *Project Statistician, Biostatistics*
[redacted] *Project Statistician, Biostatistics*

Copyright © 2017 PHASTAR. All rights reserved.
Unauthorised copying or use of this information is prohibited.

SIGNATURE PAGES

The signatories on the following pages have all read and approved this present version of the document.

Approved by:

Name:	[redacted]
Title:	Dr
Function:	Senior Medical Director

I certify that I have read this version of the Statistical Analysis Plan and approve its contents.

Signed:	DocuSigned by: [redacted] Signing Time: 23-Oct-2020 | 03:55 PDT 94C0D49A78C748F09D998E746FA969BE	Date:	23-Oct-2020 | 03:55 PDT

Approved by:

Name:	[redacted]
Title:	Mr
Function:	Project Statistician

I certify that I have read this version of the Statistical Analysis Plan and approve its contents.

Signed:	DocuSigned by: [redacted] Signing Time: 22-Oct-2020 | 02:14 PDT 3042C6FEF19F4AA7B5609885D9BFC35E	Date:	22-Oct-2020 | 02:14 PDT

SIGNATURE PAGES 2
ABBREVIATIONS 8
TRADEMARK INFORMATION 9
REVISION HISTORY 10
1. INTRODUCTION 12
2. STUDY OBJECTIVES AND ENDPOINTS 12
2.1 STUDY OBJECTIVES 12
2.1.1 PRIMARY OBJECTIVES 12
2.1.2 SECONDARY OBJECTIVES 13
2.1.3 SAFETY OBJECTIVES 13
2.2 STUDY ENDPOINTS 13
2.2.1 PRIMARY ENDPOINTS 13
2.2.2 SECONDARY ENDPOINTS 13
2.2.3 SAFETY ENDPOINTS 15
2.3 STATISTICAL HYPOTHESES 15
2.4 EXPLORATORY OBJECTIVES, ENDPOINTS AND HYPOTHESES 15
2.4.1 EXPLORATORY OBJECTIVES 15
2.4.2 EXPLORATORY ENDPOINTS 16
3. STUDY DESIGN 16
4. TIMING OF PLANNED ANALYSES 19
5. SAMPLE SIZE CONSIDERATIONS 19
6. ANALYSIS POPULATIONS 19
6.1 SAFETY POPULATION 19

6.2 PK POPULATION ... 19
6.3 BIOMARKER POPULATION ... 20
6.4 EVALUABLE FOR ANTI-TUMOUR ACTIVITY POPULATION ... 20
7. PROTOCOL DEVIATIONS ... 20
8. GENERAL CONSIDERATIONS FOR DATA ANALYSES ... 20
8.1 STANDARD SUMMARY STATISTICS ... 20
8.2 BASELINE DEFINITION ... 21
9. DATA HANDLING CONVENTIONS ... 21
9.1 PREMATURE WITHDRAWAL AND MISSING DATA ... 21
9.2 DERIVED AND TRANSFORMED DATA ... 21
9.2.1 STUDY POPULATION ... 21
9.2.1.1 DEMOGRAPHICS AND BASELINE CHARACTERISTICS ... 21
9.2.1.2 CONCOMITANT MEDICATIONS ... 21
9.2.1.3 DURATION OF EXPOSURE ... 22
9.2.1.4 DOSE INTENSITY ... 22
9.2.2 EFFICACY DERIVATIONS ... 22
9.2.3 SAFETY DERIVATIONS ... 23
9.2.3.1 ADVERSE EVENTS ... 23
9.2.3.2 SERIOUS ADVERSE EVENTS ... 23
9.2.3.3 ADVERSE EVENTS OF SPECIAL INTEREST ... 23
9.2.3.4 CLINICAL LABORATORY EVALUATIONS ... 24
9.2.3.5 VITAL SIGNS ... 24
9.2.3.6 ELECTROCARDIOGRAM (ECG) ... 24

9.2.4 ANTI-TUMOUR ACTIVITY VARIABLES.....24
9.2.4.1 TUMOUR RESPONSE.....24
9.2.4.2 BEST OBJECTIVE RESPONSE.....25
9.2.4.3 OBJECTIVE RESPONSE RATE.....25
9.2.4.4 CLINICAL BENEFIT RATE.....25
9.2.4.5 DURABLE RESPONSE RATE.....25
9.2.4.6 DURABILITY OF RESPONSE.....25
9.2.4.7 NON-PROGRESSIVE DISEASE.....26
9.2.4.8 PERCENTAGE CHANGE IN TUMOUR SIZE.....26
9.2.4.9 PROGRESSION-FREE SURVIVAL.....26
9.2.4.10 OVERALL SURVIVAL.....27
9.2.5 SERIAL TUMOUR SAMPLES - OPTIONAL.....27
9.2.6 BIOMARKERS.....27
9.2.7 PHARMACOKINETICS.....27
9.3 ASSESSMENT TIME WINDOWS.....28
10. STATISTICAL ANALYSES AND METHODOLOGY.....28
10.1 STUDY POPULATION.....28
10.1.1 DISPOSITION OF SUBJECTS.....28
10.1.2 PROTOCOL DEVIATIONS.....28
10.1.3 DEMOGRAPHIC AND BASELINE CHARACTERISTICS.....28
10.1.4 TREATMENT COMPLIANCE.....28
10.1.5 EXTENT OF EXPOSURE.....29
10.2 EFFICACY ANALYSES.....29

**10.3**	**SAFETY ANALYSES**	**29**
**10.3.1**	**ADVERSE EVENTS**	**29**
**10.3.2**	**CLINICAL LABORATORY EVALUATIONS**	**30**
**10.3.3**	**VITAL SIGNS**	**30**
**10.3.4**	**ELECTROCARDIOGRAM (ECG)**	**30**
**10.3.4.1**	**ECG PARAMETERS**	**30**
**10.3.5**	**OTHER SAFETY VARIABLES**	**31**
**10.3.6**	**ANTI-TUMOUR ACTIVITY**	**31**
**10.3.6.1**	**BEST OBJECTIVE RESPONSE**	**31**
**10.3.6.2**	**OBJECTIVE RESPONSE RATE**	**31**
**10.3.6.3**	**CLINICAL BENEFIT RATE**	**31**
**10.3.6.4**	**DURABLE RESPONSE RATE**	**31**
**10.3.6.5**	**DURABILITY OF RESPONSE**	**32**
**10.3.6.6**	**NON-PROGRESSIVE DISEASE**	**32**
**10.3.6.7**	**PERCENTAGE CHANGE IN TUMOUR SIZE**	**32**
**10.3.6.8**	**PROGRESSION-FREE SURVIVAL**	**32**
**10.3.6.9**	**OVERALL SURVIVAL**	**32**
**10.3.7**	**SERIAL TUMOUR SAMPLES - OPTIONAL**	**33**
**10.3.8**	**BIOMARKERS**	**33**
**10.4**	**CLINICAL PHARMACOLOGY**	**33**
**10.4.1**	**PHARMACOKINETICS**	**33**
**10.4.2**	**PHARMACODYNAMICS**	**35**
**10.4.3**	**PHARMACOKINETICS/PHARMACODYNAMICS**	**35**

10.5 PHARMACOGENETICS AND EXPLORATORY RESEARCH....................35

11. CHANGES FROM THE PROTOCOL-SPECIFIED ANALYSIS...................36

12. REFERENCES...36

13. APPENDIX A – LIST OF TABLES, LISTINGS AND FIGURES..................36

ABBREVIATIONS

AE	Adverse Event
AESI	Adverse Event of Special Interest
ATC	Anatomical Therapeutic Chemical
BMI	Body Mass Index
BLQ	Below Limit of Quantification
BOR	Best objective response
CBR	Clinical Benefit Rate
CR	Complete Response
CSP	Clinical Study Protocol
CTCAE	Common Terminology Criteria for Adverse Events
DLT	Dose-Limiting Toxicity
DNA	Deoxyribonucleic acid
ECG	Electrocardiogram
ECOG	Eastern Cooperative Oncology Group
ICF	Informed Consent Form
IP	Investigational Product
LLOQ	Lower Limit of Quantification
MAD	Multiple Ascending Dose
MBAD	Minimally Biologically Active Dose
MTD	Maximum Tolerated Dose
n	Frequency Count
N	Sample Size
NCA	Non-Compartmental Analysis
NE	Not Evaluable

NPD	Non-progressive disease
NTD	Non-tolerated Dose
OD	Once Daily
PD	Progressive Disease
PID	Percentage intended dose
PFS	Progression free survival
PK	Pharmacokinetics
PR	Partial Response
pRP2D	Possible Recommended Phase 2 Dose
PT	Preferred Term
RDI	Relative dose intensity
SAD	Single Ascending Dose
SAE	Serious Adverse Event
SD	Stable Disease
SOC	System Organ Class
SRC	Safety Review Committee
SAP	Statistical Analysis Plan
SI	International System of Units
TEAE	Treatment Emergent AEs
TL	Target Lesions

TRADEMARK INFORMATION

BAST	BAST Inc Ltd.
Phoenix WinNonlin	Phoenix WinNonlin is a registered trademark of Certara USA Inc.
SAS	SAS (Statistical Analysis Software) is a registered trademark of SAS Institute Inc.

Revision history

Version	Date	Summary of revisions
1.0	21-Nov-2017	Initial version
1.1	18-May2018	Section 3 - Added details of dose level selected for breast cancer cohort<br>Section 8.2 – Added definition of Baseline<br>Section 9.2.3.1 – Added details of how DLTs will be identified<br>Section 9.2.3.3 – Added details of how AEs of Special Interest will be identified<br>Section 9.2.4.1 – Added clarification that tumour response is by investigator assessment using RECIST<br>Section 10.3.5.8 and 10.3.5.9 - Removed Kaplan-Meier analysis from Part A as data not likely to be sufficient or mature enough for meaningful analysis<br>Section 9.2.7 and 10.4.1 - Removed PK analysis details as this will be performed by BAST<br>Section 9.3 – Added details of assessment time windows<br>Section 10.3.2 – Added paragraph to confirm conversion of lab parameters to SI units<br>Section 10.3.4.1 – Added details of calculation of mean values for triplicate ECG assessments<br>Referred to 'Patients' as opposed to 'Subjects' consistently in the SAP<br>Minor updates to text to clarify wording as appropriate
1.2	16-Jul-2018	Referred to 'Subjects' as opposed to 'Patients'<br>Section 9.2.3.1 Clarification of DLT identification process<br>Section 9.2.3.3 Clarification of AESI identification process<br>Section 10.1.1 Added definition of Study completion<br>Section 10.3.6 Added text to indicate this section is out of scope<br>Section 9.2.1.4 Added text to define cumulative dose calculation

		Sections 9.2.7 and 10.4 Substantial amendments to text as a result of PHASTAR performing final PK analysis.
2.0	22-Aug-2018	Updated Clinical Study Protocol version.<br>Section 6 Added details of dosing cohort for replacement subject<br>Section 6.2 Added text to clarify how PK Population will be determined.<br>Section 10.4.1 Added rules for treatment of BLQs in Pharmacokinetic concentration summaries and other minor clarifications for PK analysis.
2.1	03-Apr-2019	Section 3: Added details of SRC decision to dose final subject in breast cancer expansion cohort at 360 mg OD.<br>Section 6.4: Updated definition of Evaluable for Response Population to include subjects without measurable disease at baseline (see Appendix 11).<br>Section 9.2.4.9: Added text to clarify how a missed visit is assessed and to clarify that only subjects with post-baseline assessments are included in analysis.<br>Section 9.2.7: Added some text to describe the change in PK sampling times following Amendment 4.0 to the CSP.<br>Section 10.3.5 Added new section ‘Other Safety Variables’ and moved ECOG and Physical examination to this section. More appropriate in Safety section than Baseline section.<br>Updated Clinical Study Protocol version.
2.2	26-Jun-2019	Section 10.3.1: Summary of AEs by cycle to be included. Text to clarify that BC expansion patients will be excluded from DLT summary.<br>Section 10.3.6.2: Details of swimmer plots included.<br>Section 10.3.6.3: Percentage of subjects with stable disease for >=8 weeks, >=12 weeks, >=16 weeks to be included in summaries.<br>Section 10.3.6.7: Details of waterfall and spider plots included.
2.3	20-Sep-2019	Minor updates to CBR and NPD to improve/clarify derivations description.

2.4	08-Oct-2020	Updated Clinical Study Protocol version.<br>Section 3: Additional details of paired biopsy expansion dosing groups and updated diagram to match how the study was executed.<br>Section 4: Clarified inclusion of expansion cohort in subsequent analyses.<br>Section 7: Detailed information regarding COVID-19 related deviations and alternate procedures.<br>Update Evaluable for Response population to Evaluable for Anti-tumour Activity population and updated derivation to include baseline RECIST assessment.<br>Appendix A: Removed tables 8.3, 8.4 and 11.1<br>Section 9.2.3: Updated text to explain adverse events expected to be observed and only include thrombocytopenia in adverse events of special interest.

1. INTRODUCTION

CT7001_001 will explore the potential of CT7001 alone and in combination with other anti-cancer agents in the treatment of patients with advanced malignancies. It is the first in human study conducted using CT7001. The study is modular in design, with Module 1 designed to investigate the safety and tolerability of CT7001 in solid malignancies with the aim of identifying the both the minimally biologically active dose (MBAD) and maximum tolerated dose (MTD) of CT7001. Part A of Module 1 will investigate the safety and tolerability of CT7001 with the aim of identifying both the MBAD and MTD dose of CT7001. Part A will also include a sequential tumour biopsy expansion cohort, for evaluation of PK/PD and tumour responses once MBAD is defined.

This Statistical Analysis Plan (SAP) provides details of the summaries and analyses to be performed to report the findings of the study, specifically for Module 1, Part A. It should be read in conjunction with the Clinical Study Protocol; (CSP) CT7001_001, v13.0, (15 Jul 2020).

2. STUDY OBJECTIVES AND ENDPOINTS

2.1 STUDY OBJECTIVES

2.1.1 PRIMARY OBJECTIVES

- To investigate the safety and tolerability of CT7001 given alone or in combination with anti-cancer treatments.

- To select the CT7001 dose(s) and schedule(s) for further clinical evaluation in subjects with advanced solid malignancies.

2.1.2 SECONDARY OBJECTIVES

- To characterise the Pharmacokinetics (PK) of CT7001 after a single dose and at steady state after multiple dosing.
- To assess the biological and anti-tumour activity of CT7001.

2.1.3 SAFETY OBJECTIVES

- To investigate the safety and tolerability of CT7001 given alone or in combination with anti-cancer treatments.

2.2 STUDY ENDPOINTS

2.2.1 PRIMARY ENDPOINTS

Primary endpoints common to all modules of this study are as follows:

- Adverse Events (AEs)
- Clinical laboratory results (haematology, serum chemistry, coagulation, urinalysis)
- Physical examination findings
- Eastern Cooperative Oncology Group (ECOG) performance status
- Electrocardiogram (ECG) parameters (heart rate, PR interval, QRS complex, QT interval, and QTcF)
- Weight
- Vital signs (supine systolic and diastolic blood pressure and pulse, temperature respiratory rate, oxygen saturation)
- There are no additional primary endpoints specific to Module 1, Part A.

2.2.2 SECONDARY ENDPOINTS

Secondary endpoints for this study are as follows:

- PK Parameters for CT7001 (see below for full description)
- Biological Activity Parameters (Biomarkers)

- Anti-tumour Activity

Specific PK parameters for Module 1, Part A are:

Cycle 0 Day 1 (single dose)

- Plasma concentrations
- C_{max}: maximum observed plasma concentration
- C_{24}: plasma concentration at 24 hours
- T_{max}: time to maximum observed plasma concentration
- $T_{½}$: apparent terminal half-life
- λ_z: terminal rate constant
- λ_z span ratio: multiple of terminal half-life that the optimal regression is derived from
- AUC_{0-12}: area under the plasma concentration-time curve from Time 0 to 12 hours
- AUC_{0-24}: area under the plasma concentration-time curve from Time 0 to 24 hours
- AUC_{0-48}: area under the plasma concentration-time curve from Time 0 to 48 hours
- AUC_{0-t}: area under the plasma concentration-time curve from Time 0 to the time of the last measurable concentration
- $AUC_{0-\infty}$: area under the plasma concentration-time curve from Time 0 extrapolated to infinity
- CL/F: apparent plasma clearance
- V_z/F: apparent volume of distribution
- MRT: mean residence time

Cycle 2 Day 1

- Plasma Concentrations
- $C_{ss,max}$: C_{max} at steady state
- $C_{ss,min}$: minimum observed plasma concentration at steady state
- $T_{ss,max}$: T_{max} at steady state
- AUC_{tau}: area under the plasma concentration-time curve in the dosing interval
- CL_{ss}/F: CL/F at steady state
- MRT_{ss}: MRT at steady state

Cycle 0 Day 1, Cycle 1* and Cycle 2 Day 1

- Dose proportionality
- TCP: temporal change parameter
- R_{ac}: accumulation ratio
- Dose-normalised C_{max}
- Dose-normalised AUCs
- C_{min}:C_{max} ratio
- Trough concentrations
- Time to steady-state

*Cycle 1 trough and steady state only

Cycle 3,5,6 (multiple dose)

- Plasma concentrations
- Trough concentrations

2.2.3 SAFETY ENDPOINTS

The safety endpoints are described above in section 2.2.1.

2.3 STATISTICAL HYPOTHESES

Due to the exploratory nature of this module of the study, and the small numbers of subjects involved, no formal statistical testing will be performed.

2.4 EXPLORATORY OBJECTIVES, ENDPOINTS AND HYPOTHESES

2.4.1 EXPLORATORY OBJECTIVES

- To investigate the presence and identity of metabolites of CT7001 and, if appropriate, characterise their PK.
- To explore the relationship between PK and safety, anti-tumour activity, and biological activity and the impact of subject characteristics on PK.

- To collect and store Deoxyribonucleic acid (DNA) for future exploratory research into genes and genetic variation that may influence response to CT7001 (i.e., distribution, safety, tolerability, and efficacy).
- To collect and store pre-dose plasma and serum samples and archival tumour tissue, if available, for potential future exploratory research into factors that may influence the development of agents to treat human disease or response to CT7001 (i.e., distribution, safety, tolerability. and efficacy).
- To investigate predictive markers and acquired resistance to CT7001 that may be observed in circulating tumour DNA.
- To investigate predictive markers and acquired resistance to CT7001 that may be observed in tumour tissue.

2.4.2 EXPLORATORY ENDPOINTS

PK Parameters for Major Metabolites

- Metabolite identification
- Metabolite: CT7001 ratio.
- Predictive Markers and Acquired Resistance to CT7001

3. STUDY DESIGN

Module 1 Part A is an initial evaluation of monotherapy in subjects with advanced solid malignancies, which will investigate the safety and tolerability of CT7001 with the aim of identifying both the MBAD and MTD of CT7001.

Part A of Module 1 will commence by enrolling subjects with advanced solid tumours into a monotherapy dose escalation arm. Eligible participants will be enrolled in sequential cohorts treated with CT7001 given as an oral capsule dose while being monitored for safety and dose-limiting toxicity (DLT). Subjects will be enrolled to ensure a minimum of 3 and a maximum of 6 evaluable subjects per dose cohort, in a rolling 6 design (evaluable subjects are defined as those that receive CT7001 and have either completed minimum safety evaluation requirements and have received at least 75% of the specified dose during the first 21-day cycle or experienced a DLT during Cycle 0 or Cycle 1). Dose escalation and de-escalation will proceed as follows:

- If no dose-limiting toxicity (DLT) is observed in a cohort of 3 to 6 evaluable subjects, then dose escalation may occur. Dose increases will be permitted after review of data from a minimum of 3 evaluable subjects has been performed.

- If one subject experiences a DLT in a group of 3 or more evaluable subjects, then the cohort will be expanded to include 6 evaluable subjects. If only one DLT is observed in the complete cohort of 6 evaluable subjects, then dose escalation may occur.
- If at least 2 subjects experience a DLT in a group of up to 6 subjects, irrespective of the number of subjects enrolled, the dose will be considered not tolerated, and recruitment to the cohort and dose escalation will cease.
- A lower intermediary dose (de-escalation) may be considered to better define the MTD.

Within each cohort, subjects will receive a single dose of CT7001 on Day 1 of Cycle 0 (C0D1) followed by a 2-day washout period (C0D2), before receiving cycles of 21 continuous days dosing of CT7001 capsules.

Dose increments will be guided by safety data observed during Cycle 1, as well as on-going assessment of safety beyond Cycle 1 in earlier cohorts, plus PK and Pharmacodynamic data as available. Every new dose cohort will be evaluated for the occurrence of a DLT during Cycle 0 or Cycle 1 of treatment.

The dose for subsequent cohorts or a decision to stop recruitment to Part A will be agreed by the Safety Review Committee (SRC) after review of the data from each cohort.

Part A will also include a sequential tumour biopsy expansion cohort in breast cancer patients (n=6 evaluable subjects), for evaluation of PK and Pharmacodynamic data and tumour responses once MBAD is defined and agreed by the SRC. Evaluable subjects will have 2 evaluable samples from 2 different timepoints, for example, a baseline sample at study entry and a further sample while on treatment. At the SRC meeting on Monday 12th March 2019 it was agreed that the dose level for the breast cancer expansion cohort would be 240mg OD. At the SRC meeting on 10th September 2019, following the completion of module 1A, 360mg OD was determined a safe, tolerable and biologically active dose. This dose was therefore taken forward as the second dose level for the paired biopsy expansion cohort. The 11 dosed subjects in the paired biopsy expansion were therefore split between 240mg OD [N=5] and 360mg OD [N=6]. The subjects in this expansion cohort will be included in all safety and efficacy analyses (except DLT summaries) alongside subjects previously analysed in the MAD cohort. In addition, disposition, protocol deviation, demographic and cancer history will be presented for the paired biopsy cohort alone.

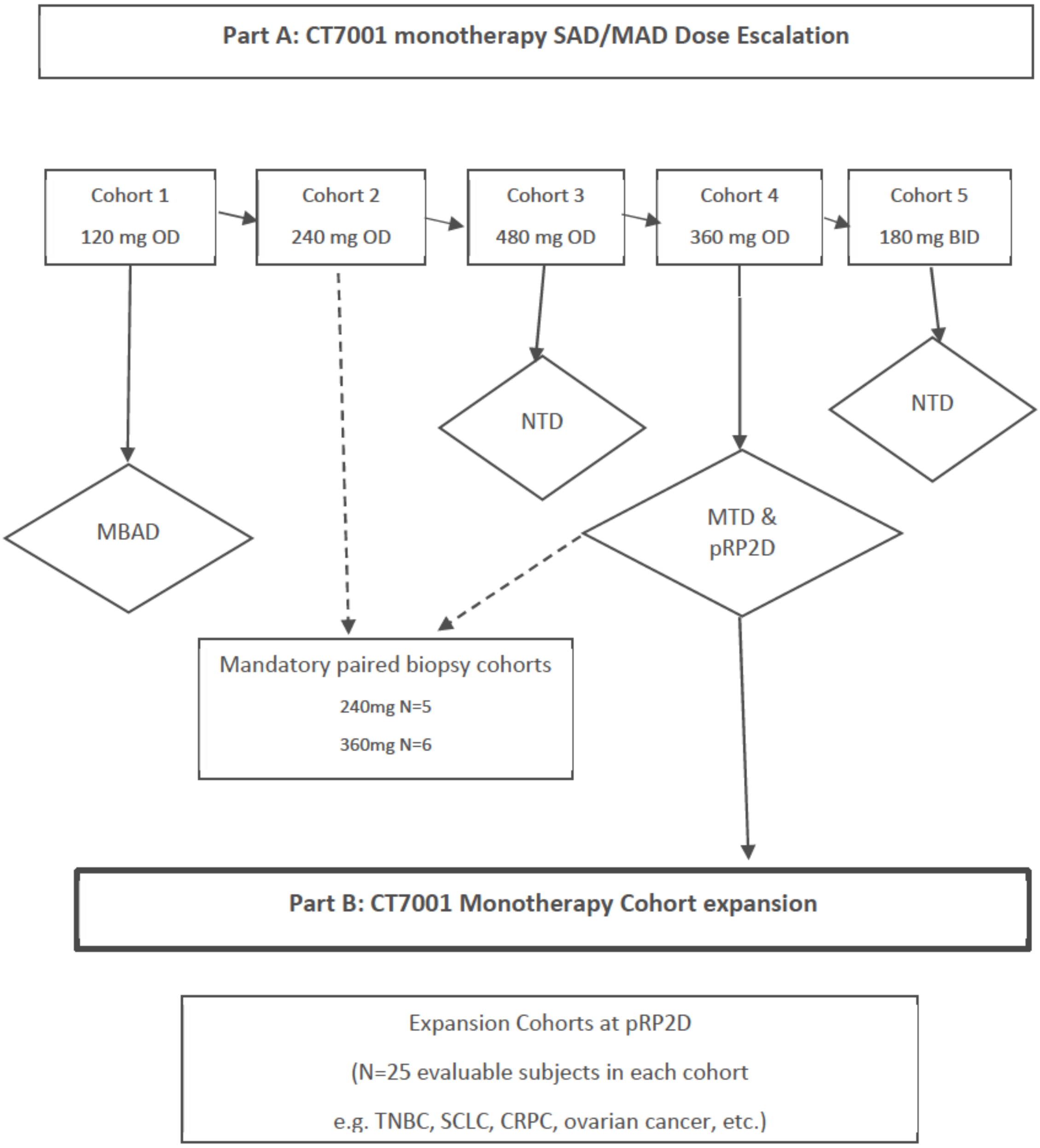
Part A: CT7001 monotherapy SAD/MAD Dose Escalation
Cohort 1
120 mg OD
Cohort 2
240 mg OD
Cohort 3
480 mg OD
Cohort 4
360 mg OD
Cohort 5
180 mg BID
MBAD
NTD
MTD &
pRP2D
NTD
Mandatory paired biopsy cohorts
240mg N=5
360mg N=6
Part B: CT7001 Monotherapy Cohort expansion
Expansion Cohorts at pRP2D
(N=25 evaluable subjects in each cohort
e.g. TNBC, SCLC, CRPC, ovarian cancer, etc.)

4. Timing of planned analyses

Formal statistical analysis will not be performed for this study. Safety and tolerability will be assessed throughout the study by recording of AEs and concomitant medications, clinical laboratory evaluations, physical examinations, ECOG performance status, weight, ECG and vital signs. Data from each cohort will be reviewed by the SRC to determine the dose for subsequent cohorts, or a decision to stop recruitment to Part A.

Subjects from the paired biopsy expansion cohort will be included in the CSR alongside all subjects from the dose escalation study.

5. Sample size considerations

The primary objective of the dose-escalation phase (Part A) of Module 1 is to investigate the safety and tolerability of CT7001 to enable determination of both the MBAD and MTD of CT7001 and of the dose(s) and schedule(s) for evaluation in the expansion phase (Part B) of this module.

The cohort size of all study modules is based upon accepted methodology for Phase I oncology studies. The sample size of cohorts of 3 to 6 subjects was based on the requirement for adequate safety, PK, and Pharmacodynamic data balanced with exposure of as few subjects as possible to the investigational product (IP) and study procedures. These cohorts may be expanded by up to 12 additional evaluable subjects at doses at or above the minimally biological active dose. The total number of subjects in Module 1 Part A will depend on this potential expansion of cohorts and the number of dose escalations conducted.

6. Analysis populations

In all analysis populations, subjects will be analysed according to the dose they actually received. Subject M1A03R303 was originally recruited as a replacement for the 480 mg OD cohort, but was subsequently assigned to receive 240 mg OD following the SRC decision that 480 mg OD was not tolerated. This subject will be summarised in the 240 mg OD cohort.

6.1 Safety Population

The Safety population is defined as all subjects who received at least 1 dose of CT7001.

6.2 PK Population

The PK population is defined as all subjects who received at least 1 dose of CT7001 and who have at least 1 CT7001 plasma concentration above the lower limit of quantification and no important AEs or

protocol deviations or other event that may impact PK analysis. Subjects included in the PK population will be determined by Carrick prior to database lock.

6.3 Biomarker Population

The Biomarker population is defined as all subjects who received at least 1 dose of CT7001 and provided at least 1 biomarker sample.

6.4 Evaluable for Anti-tumour Activity Population

The Evaluable for Anti-tumour Activity Population is defined as all subjects who received at least 1 dose of CT7001, have a baseline RECIST assessment and have at least one other RECIST assessment.

7. Protocol Deviations

Major protocol deviations will be captured and provided to summarise at the end of the study. A major protocol deviation is defined as a significant deviation i.e. one that may affect to a significant degree the safety or physical or mental integrity of the subjects of the study or the scientific value of the study or one which reflects a significant deviation from the standards of Good Clinical Practice (e.g. accidental release of confidential subject information). In the biopsy expansion cohort, failure to obtain sufficient tumour sample after making best efforts to biopsy the tumour will be considered a protocol deviation.

In line with FDA guidance, the document "CT70001_001 Protocol Deviation Guidance During COVID-19 v2.0 29Jul2020.docx" details the process for recording and assessing COVID-19 related protocol deviations and alternate procedures during the pandemic. Any procedure that is conducted by an alternate location, process, method or schedule is added to the Alternate Procedure Tracker and will be provided for the CSR.

8. General considerations for data analyses

8.1 Standard summary statistics

Data analyses will be descriptive in nature. Continuous variables will be summarised using n, mean, standard deviation, median, minimum value, and maximum value. Categorical variables will be summarised using the sample size (N), frequency count (n), and percentage, calculated relative to the total number of subjects in the relevant analysis population. Hypothesis testing will not be performed. All analyses and listings will use the observed data. Missing values will not be imputed.

Summary statistics will be presented to the following level of precision (unless otherwise specified): mean and median 1 more decimal place than raw data, standard deviation 2 more decimal places than

raw data, and, minimum and maximum same precision as raw data. Percentages will be displayed to 1 decimal place.

All tables and listings of the data will be generated using the SAS System®, Version 9.1 or later.

8.2 Baseline definition

Unless otherwise specified, baseline will be defined as the last result prior to first dose on Cycle 0 Day 1, based on the date and time of the assessment. In cases where the time of the assessment is not collected it will be assumed that it was performed prior to dosing on Cycle 0 Day 1 and will therefore be considered as Baseline.

9. Data handling conventions

9.1 Premature withdrawal and missing data

Only data recorded will be included in any tables, figures or listings.

9.2 Derived and transformed data

9.2.1 Study Population

9.2.1.1 Demographics and Baseline Characteristics

Age, in whole years, will be calculated with respect to the subject's Screening visit (calculated in the eCRF).

Body Mass Index (BMI) will be calculated as Weight (kg) / Height (m)^2

9.2.1.2 Concomitant medications

Medications will be classified as prior, concomitant, or, post-treatment based on start and stop dates in relation to study treatment dates.

- Prior medications: Those ending before the start date of study treatment.
- Concomitant medications: Those taken at any time between the start date and stop date of study treatment, inclusive. Medications that started before study treatment but continued during this period are also considered as concomitant medications.
- Post treatment medications: Those started after the stop date of study treatment.

It will be assumed that medication has been taken on the date which it is reported as started or stopped. For any medication starting on the same date as study treatment and where time medication is taken is missing, it will be assumed that the medication was taken after the subject started taking study treatment (i.e. concomitant). Any other situation where it cannot be determined whether medication was stopped prior to first dose of IP, i.e. missing month or year in a partial date, the medication will be assumed to be concomitant.

9.2.1.3 DURATION OF EXPOSURE

Number of days of exposure to study drug will be calculated as:

Duration of Exposure in Days = (Treatment Stop Date – Treatment Start Date) + 1

9.2.1.4 DOSE INTENSITY

Relative dose intensity (RDI) is the percentage of actual dose received relative to the intended dose through to treatment discontinuation and percentage intended dose (PID) is the percentage of the actual dose received relative to the intended dose through to progression. RDI and PID will be defined as follows:

- RDI = 100% * d/D, where d is the actual cumulative dose delivered up to the actual last day of dosing and D is the intended cumulative dose up to the actual last day of dosing. D is the total dose that would be delivered, if there were no modification to dose or schedule.

- PID = 100% * d/D, where d is the actual cumulative dose delivered up to progression (or a censoring event) and D is the intended cumulative dose up to progression (or a censoring event, as defined in section 9.2.4.9). D is the total dose that would be delivered, if there were no modification to dose or schedule.

The entire intended treatment period will be used in the derivation of RDI and PID. RDI and PID will be the same for any subject where the last day of dosing is the same as the date of progression. The actual cumulative dose will be calculated based on subject dosing information as recorded in the eCRF.

9.2.2 EFFICACY DERIVATIONS

Anti-tumour activity derivations are described in section 9.2.4

9.2.3 SAFETY DERIVATIONS

9.2.3.1 ADVERSE EVENTS

All clinical AEs occurring after the subject signs the informed consent form (ICF) and until the End of Study visit of each module will be recorded on the AE eCRF. Medical conditions that exist before signing the ICF will be recorded as part of medical history. The Investigator will assess the relationship between the IP and each AE to determine whether the AE is considered to be causally related to IP. Causally related AEs are those with a relationship of 'Possibly Related', 'Probably Related' and 'Related'.

AEs will also be classified as DLTs through medical review of all AE terms, identified by Data Management during the query review process. AEs considered to be DLTs will be flagged in the AEs listings, which will be reviewed during the dry run/final analysis.

AEs that have been observed in conjunction with CT7001 can be defined by three categories; AEs that offer the most commercial interest (neutropenia and febrile neutropenia), AEs that offer the best insight into patients' quality of life (nausea, vomiting and diarrhoea) and events that are of most interest to regulators (thrombocytopenia and low platelet counts).

9.2.3.2 SERIOUS ADVERSE EVENTS

A serious adverse event (SAE) is an AE occurring during any study that fulfils one or more of the following criteria:

- Results in death
- Is immediately life-threatening
- Requires inpatient hospitalisation or prolongation of existing hospitalisation
- Results in persistent or significant disability/ incapacity or substantial disruption of the ability to conduct normal life functions
- Is or results in a congenital abnormality or birth defect
- Is an important medical event that may jeopardise the subject or may require medical intervention to prevent one of the outcomes listed above.

Life-threatening means that the subject was at immediate risk of death at the time of the SAE; it does not refer to a serious AE that hypothetically might have caused death if it were more severe.

All SAEs will be reported from consent until the End of Study visit. An SAE that occurs after the End of Study visit and comes to the attention of the Investigator will be reported only if there is (in the opinion of the Investigator) reasonable causal relationship with the study drug.

9.2.3.3 ADVERSE EVENTS OF SPECIAL INTEREST

For the purpose of this module, thrombocytopenia and low platelet counts will be the only adverse events of special interest analysed as per regulatory recommendation.

9.2.3.4 Clinical laboratory evaluations

Haematology (including reticulocyte count), serum chemistry (including CA125 / tumour specific biomarkers), urinalysis and coagulation samples will be assessed as specified in the protocol. Reticulocyte decrease is expected based on the target engagement of CDK7 inhibition.

9.2.3.5 Vital signs

Systolic and diastolic blood pressure and pulse will be measured as specified in the protocol. Measurements will be taken after the subject has been resting semi supine for at least 10 minutes. Oral temperature, respiratory rate, and oxygen saturation will also be measured.

9.2.3.6 Electrocardiogram (ECG)

When specified in the protocol, triplicate 12-lead ECG will be performed 3 to 5 minutes apart after the subject has been resting semi supine for at least 10 minutes. Heart rate, RR interval, PR interval, QRS complex, QT interval, and QTcF will be recorded. QTc will be calculated using Fridericia and Framingham correction formulae. This method shows the best rate correction and significantly improved prediction of 30-day and 1-year mortality. In general, Bazett’s correction overcorrects at elevated heart rates and under corrects at heart rates below 60 bpm and hence is not an ideal correction. Fridericia’s correction is more accurate than Bazett’s correction in subjects with such altered heart rates.

9.2.4 Anti-tumour Activity variables

Anti-tumour activity will be assessed as defined in the protocol.

9.2.4.1 Tumour Response

At each tumour assessment visit in each module subjects will be assigned tumour response of Complete Response (CR), Partial Response (PR), Stable Disease (SD) or Progressive Disease (PD) by the investigator according to Response Evaluation Criteria in Solid Tumours (RECIST) version 1.1. Assessments will be based on the status of their disease compared with baseline and previous visit assessments.

In the absence of progression, tumour response (CR, PR, SD) will be calculated in comparison to the baseline tumour measurements obtained before starting treatment.

If a subject has had a tumour assessment which cannot be evaluated, then the subject will be assigned a visit response of Not Evaluable (NE) unless there is evidence of progression in which case the response will be assigned as PD.

The investigator assessed RECIST response will be used for the analysis.

9.2.4.2 Best Objective Response

Best objective response (BOR) will be determined for each subject based on the best response recorded from the start of study treatment to the end of treatment, including any assessments for confirmation after the end of treatment in that module.

9.2.4.3 Objective Response Rate

Objective response rate is defined as the percentage of subjects who have at least one response of CR or PR prior to any evidence of progression.

9.2.4.4 Clinical Benefit Rate

The Clinical Benefit Rate (CBR) is defined as the percentage of subjects with a confirmed reduction in tumour burden, CR or PR, or stabilisation of disease for at least 24 weeks.

9.2.4.5 Durable Response Rate

The Durable Response Rate is defined as the percentage of subjects who have a confirmed response (CR or PR) with a duration of at least 3 months.

Duration of response will be defined as the time from the date of first documented response until date of documented progression or death in the absence of disease progression, the end of response should coincide with the date of progression or death from any cause used for the Progression free survival (PFS) endpoint. The time of the initial response will be defined as the date of the first assessment with a response of PR or CR.

If a subject does not progress following a response, then their duration of response will use the PFS censoring time, defined in Section 9.2.4.9.

9.2.4.6 Durability of Response

Durability of Response is defined as the time (in days) from documentation of tumour response to disease progression (i.e., date of PFS event or censoring – date of first response + 1).

The time of the initial response will be defined as the date of the first assessment with a response of CR or PR. The end of response should coincide with the date of progression or death from any cause used for the PFS endpoint. If a subject does not progress following a response, then their durability of response will use the PFS censoring time, defined in Section 9.2.4.9.

9.2.4.7 Non-progressive Disease

Non-progressive disease (NPD) will be assessed at specified timepoints after the start of therapy. NPD is defined as the proportion of all subjects dosed that have a visit response of SD, PR or CR at the specified timepoint.

If a patient has a response of CR or PR at an earlier visit that becomes PD or NE at the specified timepoint, this would not be classified as NPD at the specified timepoint.

A time window of 1 week around the specified timepoint will be applied and it is recommended that any visits occurring within this window after dosing are acceptable; however, if an earlier visit is defined as PD then the visit response at the specified timepoint would also be defined as PD.

If the response at the specified timepoint is missing or NE but the next evaluable response is SD or better, then the subject will be defined as having NPD at the specified time.

9.2.4.8 Percentage Change in Tumour Size

Percentage change in TL tumour size will be determined at each visit for subjects in a module with measurable disease at baseline and is derived at each visit by the percentage change from baseline in the sum of the diameters of TLs.

The best percentage change in TL tumour size from baseline, defined as either the maximum reduction from baseline or the minimum increase from baseline in the absence of a reduction, at any visit, will be determined for every subject with measurable disease at baseline.

9.2.4.9 Progression-free Survival

Progression free survival (PFS) is defined as the time from start of treatment until the date of objective disease progression or death (by any cause in the absence of progression) regardless of whether the subject withdraws from therapy or receives another anti-cancer therapy prior to progression. Subjects who have not progressed or died at the time of analysis will be censored at the time of the latest date of assessment from their last evaluable RECIST assessment. However, if the subject progresses or dies after two or more missed visits, the subject will be censored at the time of the latest evaluable RECIST assessment. Given that RECIST response is assessed every other cycle and a cycle is 21 days, 1 missed visit would equate to 42 days (2*21) plus a 7 day window, a total of 49 days. Therefore 2 missed visits would equate to a total of 98 days. If the subject has no evaluable visits or does not have baseline data they will be censored at 0 days unless they die within two visits of baseline (in which case PFS is defined as the time from start of treatment until the date of death, by any cause).

The PFS time for each module will always be derived based on scan/assessment dates not visit dates. Only subjects with evaluable baseline RECIST assessment and at least one post-baseline RECIST assessment will be included in the analysis of PFS.

9.2.4.10 Overall Survival

Overall Survival is defined as the time from start of treatment until death from any cause. Any subject not known to have died at the time of analysis will be censored based on the last recorded date on which the subject was known to be alive.

9.2.5 Serial Tumour Samples - Optional

For subjects with accessible lesions and who have provided consent, collection of fresh serial tumour biopsies is encouraged at the timepoints specified in the protocol. Timing of these samples may be changed based on emerging PK or Pharmacodynamic data available during the trial. An accessible lesion is defined as a tumour lesion that is amenable to repeat biopsy, unless clinically contraindicated or the subject has withdrawn consent.

These samples will be analysed for CDK7 pathway biomarkers that may include but are not limited to phospho-Pol II, Ki67 and Cleaved Caspase.

9.2.6 Biomarkers

Blood samples for peripheral blood mononuclear cell and plasma for circulating tumour DNA analysis will be used for biomarker and genetic analyses, respectively, and will be collected as specified in the protocol.

9.2.7 Pharmacokinetics

PK analyses will be performed by BAST Inc. Ltd using the PK population, where possible the PK Parameters defined in Section 2.2.2 will be determined. The actual sampling times for each module will be used and PK parameters, as listed in Section 2.2.2, will be derived by BAST using standard non-compartmental methods using R Statistical Package or Phoenix WinNonLin. Time to steady-state will be determined through comparison of C2D1 trough concentrations with C1D1, C1D8 and C1D15 trough concentrations and potentially C3, C5 and C7 trough concentrations.

The procedures used to perform the PK analyses will follow BAST Best Practice for Non-Compartmental Analysis of PK Data, version 2, 16th August 2017 or later.

Note that following substantial amendment 4.0 to the CT7001_001 clinical protocol, the PK sampling times for Module 1 Part A were changed. These revised sampling times apply to Cohort 4 onwards of Module 1 Part A. Cohorts 1 – 3 sampling times were as per the original sample analysis phase plan excluding 2 subjects in Cohort 4 that were incorrectly sampled according to the earlier version of the schedule.

9.3 ASSESSMENT TIME WINDOWS

Only data from nominal protocol-scheduled visits/time points will be included in the summary statistics, i.e. unscheduled and repeated tests will not be included. All data collected will be presented in the listings.

10. STATISTICAL ANALYSES AND METHODOLOGY

10.1 STUDY POPULATION

10.1.1 DISPOSITION OF SUBJECTS

Subject disposition will be summarised by CT7001 treatment group for all subjects who provide informed consent. The number and percentage of subjects enrolled in the study module, completing the study module, and discontinuing the study module will be presented in a tabular format. The primary reasons for discontinuation will also be summarised by CT7001 treatment group. A subject is determined to have completed the module if they have received at least 75% of the specified dose during the first 21 day cycle, or experienced a DLT during C0 or C1, but has withdrawn after this due to disease progression (including no clinical benefit) or poor tolerability, and will be recorded on the Study Completion/Withdrawal page of the eCRF.

A summary of analysis populations will be tabulated.

10.1.2 PROTOCOL DEVIATIONS

Important protocol deviations will be listed by subject. Listing will also show whether protocol deviations were related to COVID-19 or not.

10.1.3 DEMOGRAPHIC AND BASELINE CHARACTERISTICS

Demographics and baseline characteristics (including medical history and baseline disease characteristics) will be summarised descriptively by CT7001 treatment group for the Safety Population in each study module.

Prior and concomitant medications will be coded using the most current World Health Organisation Drug Dictionary and summarised by CT7001 treatment group, Anatomical Therapeutic Chemical (ATC) level 3 and generic term. All prior, concomitant and post-treatment medications will be listed.

10.1.4 TREATMENT COMPLIANCE

Not applicable.

10.1.5 Extent of exposure

Total exposure (date of last dose minus date of first dose+1) and total time on study (date of discontinuation minus date of first dose+1) will be summarised descriptively by CT7001 treatment group. Mean actual cumulative dose and mean dose per day, calculated per subject as cumulative dose over treatment period divided by total exposure, will also be summarised by CT7001 treatment group.

In addition, the number and percentage of subjects with at least 1 dose interruption/dose delay (i.e. subjects with an adverse event with action of 'Drug interrupted' as recorded in the eCRF) and at least 1 dose reduction (i.e. subjects with an adverse event with action of 'Dose Reduced' as recorded in the eCRF) will be presented separately for the initial period of evaluability defined in each module and for any time in the study after this initial period.

RDI and PID will be summarised descriptively by CT7001 treatment group. In addition, the number and percentage of subjects will be summarised categorically by CT7001 treatment group for RDI and PID.

10.2 Efficacy analyses

Anti-tumour activity endpoints will be analysed in each module using the Evaluable for Anti-tumour Activity Population. These are all detailed in Section 10.3.6.

10.3 Safety analyses

Safety analyses will be performed using the Safety Population. All summaries by visit will use the visit recorded. Safety data from all cycles of treatment within a module will be combined in the presentation.

10.3.1 Adverse events

AEs will be listed individually by subject, cohort, and CT7001 dose. For subjects who have a dose modification, all AEs (regardless of relationship to CT7001) will be assigned to the initial CT7001 dose.

AE summary tables will include only treatment emergent AEs (TEAEs), defined as those AEs which occur from Cycle 0 Day 1 of the study module to 28 days after the last dose in a module. Any AE that occurs before the first dose of CT7001 (i.e., before Cycle 0 Day 1) or after the 28-day follow-up period will be listed only and not included in the summaries.

A subject level summary of TEAEs will be presented including the number and percentage of subjects in the following categories: any AE, AEs of CTCAE grade 3 or higher, AEs with outcome of death, SAEs, AEs leading to discontinuation of IP, AEs causally related to CT7001 and AEs classified as DLTs. Note

that subjects in the breast cancer expansion cohort will not be included in the summary of AEs classified as DLTs as these subjects were not part of the dose escalation procedure.

The number of subjects experiencing each TEAE will be summarised by CT7001 treatment group, MedDRA System Organ Class (SOC) and MedDRA Preferred Term (PT). In addition, the event rate per 100 subject years will be presented, calculated as the number of subjects with AEs divided by the total duration of treatment across all subjects in a given group, multiplied by 100. TEAEs will also be summarised by cycle, SOC and PT. Cycle will be based on the reported start date of the TEAE, using treatment start dates for each cycle to determine in which cycle the TEAE falls. For instance, a TEAE starting after the dose on Cycle 1 Day 1 but before Cycle 2 Day 1 would be assigned to Cycle 1 and so on.

The number of subjects experiencing each TEAE will also be summarised by CT7001 treatment group, MedDRA SOC, MedDRA PT, and Common Terminology Criteria for Adverse Events (CTCAE) grade.

In addition, the number of subjects experiencing each TEAE will be summarised by CT7001 treatment group, MedDRA SOC and MedDRA PT, separately for the following categories: AEs classified as DLTs (excluding subjects in the breast cancer expansion cohort), AEs causally related to CT7001, AEs of special interest, AEs with CTCAE grade >=3 and SAEs.

Details of any deaths, SAEs, AEs with outcome of death, AEs of special interest and AEs leading to study discontinuation will be listed separately.

10.3.2 CLINICAL LABORATORY EVALUATIONS

All laboratory results will be listed individually by subject and summarised descriptively by visit and CT7001 treatment group.

Haematology and biochemistry values will be converted into SI units. Conversions for all parameter units will be approved by medic review.

10.3.3 VITAL SIGNS

Weight will be listed individually by subject and summarised by visit and CT7001 treatment group descriptively.

10.3.4 ELECTROCARDIOGRAM (ECG)

10.3.4.1 ECG PARAMETERS

ECG parameters will be listed individually by subject and the mean of triplicates will be summarised descriptively by visit and CT7001 treatment group. If an individual has more than three assessments at any one visit/time point, then the latest three readings will be used to calculate the mean of the triplicate.

10.3.5 Other Safety variables

ECOG performance status will be listed individually by subject.

Abnormal physical examination findings will be listed.

10.3.6 Anti-tumour activity

Anti-tumour activity endpoints will be analysed in each module using the Evaluable for Anti-tumour Activity Population. Details of Lesion Measurements, including RECIST assessments, will be provided in a listing.

10.3.6.1 Best Objective Response

BOR will be summarised by CT7001 treatment group for the number and percentage of subjects in each category of response (CR, PR, SD, PD, NE). Percentage of subjects with each response calculated will be based on the total number of subjects with a baseline RECIST assessment.

10.3.6.2 Objective Response Rate

The number and percentage of subjects with Objective Response will be summarised by CT7001 treatment group. Objective response rate will be calculated separately for all subjects in the Evaluable for Anti-tumour Activity Population and subjects this population who have measurable disease at baseline.

The response profile for each subject's tumour response while on treatment will be presented in a swimmer plot, which will display a bar representing time from start of treatment until discontinuation with PD/Death and responses of CR and PR indicated at the day they occur. One dose level will be presented per page and different fill pattern used depending on the location of the subject's primary malignancy according to the categories "Breast", "Prostate" and "Other".

10.3.6.3 Clinical Benefit Rate

The number and percentage of subjects with CBR (stable disease >= 24 weeks) will be summarised by CT7001 treatment group. The number and percentage of subjects with stable disease >= 8 weeks, >= 12 weeks, >= 16 weeks will also be summarised.

10.3.6.4 Durable Response Rate

The number and percentage of subjects with a durable response, i.e. response for at least 3 months, will be summarised by CT7001 treatment group.

10.3.6.5 DURABILITY OF RESPONSE

Durability of Response, defined as the time from documentation of tumour response to disease progression or death, will be summarised by CT7001 treatment group using standard summary statistics.

10.3.6.6 NON-PROGRESSIVE DISEASE

The number and percentage of subjects with NPD at each visit will be summarised by CT7001 treatment group.

10.3.6.7 PERCENTAGE CHANGE IN TUMOUR SIZE

Percentage change in TL tumour size for subjects with measurable disease at baseline will be descriptively summarised by CT7001 treatment group and visit. The best percentage change in TL tumour size from baseline, as defined in section 9.2.4.8, will also be summarised by CT7001 treatment group and displayed graphically in the form of a waterfall plot. Subjects with primary malignancy in breast or prostate will be indicated on the waterfall plot using different pattern/colour.

Changes in tumour size will also be displayed graphically by means of a spider plot showing individual subject's percentage change in tumour size over time, with reference lines included at +20% and -30%, corresponding with the definitions of progression and partial response according to RECIST v1.1 guidelines.

10.3.6.8 PROGRESSION-FREE SURVIVAL

Progression status at the time of the analysis will be summarised descriptively by CT7001 treatment group for the Evaluable for Anti-tumour Activity Analysis Population using the investigator's assessment of overall tumour response (RECIST v1.1.). The summary will include total number and percentage of subjects with an event (RECIST progression or death in the absence of progression), number and percentage of censored subjects (no RECIST progression or death at time of analysis, RECIST progression or death after 2 or more missed visits).

10.3.6.9 OVERALL SURVIVAL

Overall Survival at the time of analysis will be summarised by CT7001 treatment group for the Evaluable for Anti-tumour Activity Analysis Population. The number and percentage of subjects in each survival category (Death and Censored category) will be presented.

10.3.7 Serial Tumour Samples - Optional

CDK7 pathway biomarkers, e.g. phospho-Pol II, Ki67 and Cleaved Caspase will be summarised by visit and CT7001 treatment group using standard summary statistics and changes over time will be plotted. These analyses fall outside the scope of this SAP.

10.3.8 Biomarkers

Blood samples for peripheral blood mononuclear cell and plasma for circulating tumour DNA analysis will be collected. Analysis of these biomarkers is outside the scope of this SAP.

10.4 Clinical Pharmacology

10.4.1 Pharmacokinetics

All pharmacokinetic concentrations of CT7001 will be listed individually by subject and any subjects not included in the analysis population will be flagged. All PK parameters will be listed for any subject with a measurable plasma concentration.

Plasma concentrations of CT7001 obtained will be summarised by nominal sample time and CT7001 treatment group for the PK analysis set. Derived PK parameters, as provided by BAST, will be summarised by CT7001 treatment group. Parameters following single and multiple dosing will be summarised separately. Non-compartmental analysis (NCA), performed by BAST, will include data from Cycle 0 Day 1 for the single dose, and Cycle 2 Day 1 for multiple dosing. Plasma concentrations at each time point and PK parameters will be summarised by CT7001 treatment group using appropriate summary statistics for the PK analysis set as listed below.

The following summary statistics will be presented for plasma concentrations and all PK parameters except for T_{max} and $T_{ss,max}$:

- The geometric mean (gmean, calculated as exp[μ], where μ is the mean of the data on a logarithmic scale)
- Geometric coefficient of variation (GCV, calculated as 100 √ [exp(s^2)-1], where s is the standard deviation of the data on the logarithmic scale)
- Geometric standard deviation (Geo_{SD}) calculated as exp(s)
- Geometric mean ±SD (calculated as exp[μ±s]) (plasma concentration only)
- Arithmetic mean calculated using untransformed data
- Coefficient of variation (CV%) using untransformed data
- Standard Deviation calculated using untransformed data
- Minimum
- Median
- Maximum
- Number of observation (n)

The following summary statistics will be presented for T_{max} and $T_{ss,max}$:

- Median
- Minimum
- Maximum
- Number of observations (n)

In the listings and summary statistics of the PK parameters, reporting precision and parameter symbols will follow Appendix 6 of BAST Best Practice for Non-Compartmental Analysis of PK Data, version 2 or later. For PK concentrations, listings will report concentrations to the same level of precision as the raw data and in the summaries, minimum and maximum will follow the raw data and all other summaries will be reported to 4 significant figures (as specified in BAST Best Practice for Non-Compartmental Analysis of PK data, Appendix 6).

The rules for treatment of samples below the limit of quantification (BLQ), used by BAST in the non-compartmental analysis, are as follows:

1. For analysis of data from the first dose, all BLQ observations prior to the first measurable concentration will be included in the non-compartmental analysis (NCA) calculations with a concentration set to zero. These samples will contribute to the listings and tables summary statistics as NQ (not quantifiable).
2. Any other time points (in data from both first dose and after multiple doses) with concentration BLQ will be excluded from the NCA calculations. These samples will contribute to listings and tables summary statistics calculations as NQ.
3. If two or more consecutive BLQ concentrations at times after T_{max} are followed by quantifiable concentration(s), then these quantifiable values are excluded from the NCA calculations (unless there is scientific rationale not to do so), and this is documented. These samples will not contribute to listings and tables summary statistic calculations.

Reporting of descriptive statistics of the PK concentration data in the presence of BLQ observations will follow the rules below, as per BAST Best Practice for Non-Compartmental Analysis of PK Data, version 2, 16th August 2017 or later:

1. If, at a given nominal time point, 50% or fewer of the plasma concentrations are NQ, the geometric mean, geometric SD, geometric CV, arithmetic mean and arithmetic SD are calculated by substituting the lower limit of quantification (LLOQ) for values which are NQ.
2. If more than 50%, but not all, of the concentrations are NQ, the geometric mean, geometric SD, geometric CV, arithmetic mean and arithmetic SD are reported as not calculable (NC). The maximum concentration will be reported from the individual data, and the minimum and median will be set as NQ.
3. If all the concentrations are NQ, then all properties are reported as NQ.
4. The number of observations above LLOQ are reported for each time point along with the total number of observations.
5. Three observations > LLOQ are required as a minimum for a plasma concentration to be summarised.

Pharmacokinetic graphs

The pharmacokinetic concentration data will be displayed graphically. Individual plasma PK concentration-time profiles will be plotted by treatment using the actual sample time. Individual profiles will be overlaid on the same plot and one figure will be produced per treatment group on both the linear/linear and log/linear scale. Geometric mean (x/÷ geometric SD) concentration-time profiles will be produced and presented on the linear/linear and log/linear scale using nominal sampling time. All treatments will be overlaid on the same plot and a different line style and colour used for each treatment, clearly indicated in a figure legend. Plots of trough geometric mean concentration (x/÷ geometric SD) will also be produced on the linear/linear and log/linear scale, all treatment groups overlaid on the same figure. Plots will be stratified by single and multiple dose. For consistency the same plasma concentration values are used in the building of any mean data figures as those given in the descriptive statistics summary table for each time point.

For the individual figures, values reported as below the assay lower limit of quantification (LLOQ) will be set to LLOQ and for the mean figures time points where all concentrations are < LLOQ will be plotted at the assay LLOQ.

Dose proportionality analysis

Plots of individual and gmean AUC values and C_{max} after single and multiple dosing versus dose, or log-dose will be produced. Dose normalising will be carried out relative to the starting dose and box-plots of the dose normalised AUC and Cmax values at each dose level will be produced.

10.4.2 PHARMACODYNAMICS

Not Applicable.

10.4.3 PHARMACOKINETICS/PHARMACODYNAMICS

Not Applicable.

10.5 PHARMACOGENETICS AND EXPLORATORY RESEARCH

The results of exploratory biomarker research may be pooled with biomarker data from other studies with the study drug to generate hypotheses to be tested in future studies. The plasma concentration data for CT7001 will be analysed using a population PK approach, which may include investigating the influence of covariates on PK. A population pharmacodynamic approach may be used to investigate the relationship between dose, PK and selected primary, secondary and/or exploratory endpoints. Results will be reported separately from the Clinical Study Report. These data may also be combined with similar data from other studies and explored using population PK and/or PK-pharmacodynamic methods and will be reported separately from the Study Report. Metabolite ID may be performed on pooled samples and will be reported separately.

11. CHANGES FROM THE PROTOCOL-SPECIFIED ANALYSIS

Section 6.4: Definition of the Evaluable for Response Anti-tumour Activity Population altered to include subjects who did not have measurable disease at baseline owing to the fact that the Module 1A inclusion criteria does not have this stipulation.

12. REFERENCES

Not Applicable

13. APPENDIX A – LIST OF TABLES, LISTINGS AND FIGURES

Tables

<u>Demographic, Baseline, Concomitant Medication and Other Subject-Specific Characteristics</u>

Table 1.1.1 Subject disposition (Multiple ascending dose cohort)

Table 1.1.2 Subject disposition (Paired biopsy cohort)

Table 1.2 Analysis populations (All subjects)

Table 2.1.1 Demographic characteristics (Safety Analysis Population - Multiple ascending dose cohort)

Table 2.1.2 Demographic characteristics (Safety Analysis Population - Paired biopsy cohort)

Table 3.1 Medical history (Safety Analysis Population)

Table 3.2.1 Cancer history – Prior cancer treatment (Safety Analysis Population - Multiple ascending dose cohort)

Table 3.2.2 Cancer history – Prior cancer treatment (Safety Analysis Population - Paired biopsy cohort)

Table 3.3.1 Cancer history at screening (Safety Analysis Population - Multiple ascending dose cohort)

Table 3.3.2 Cancer history at screening (Safety Analysis Population - Paired biopsy cohort)

Table 4.1 Prior medications (Safety Analysis Population)

Table 4.2 Concomitant medications (Safety Analysis Population)

Table 5.1 Nicotine use, categorised (Safety Analysis Population)

<u>Safety Data</u>

Table 6.1 Duration of exposure and cumulative exposure over time (Safety Analysis Population)

Table 6.2 Dose intensity of CT7001 (Safety Analysis Population)

Table 7.1.1 Adverse events in any category – subject level (Safety Analysis Population)

Table 7.1.2 Adverse events in any category – event level (Safety Analysis Population)

Table 7.2 Adverse events and event rate by system organ class and preferred term (Safety Analysis Population)

Table 7.3 Adverse events by cycle, system organ class and preferred term (Safety Analysis Population)

Table 7.4 Adverse events by system organ class, preferred term and maximum CTCAE grade (Safety Analysis Population)

Table 7.5 Adverse events classifies a Dose Limiting Toxicity by system organ class and preferred term (Safety Analysis Population - Multiple ascending dose cohort)

Table 7.6 Adverse events causally related to CT7001, by system organ class and preferred term (Safety Analysis Population)

Table 7.7 Adverse events of special interest (Safety Analysis Population)

Table 7.8 Adverse events with CTCAE grade >=3, by system organ class and preferred term (Safety Analysis Population)

Table 7.9 Serious adverse events by system organ class and preferred term (Safety Analysis Population)

Table 8.1 Haematology laboratory variables over time (Safety Analysis Population)

Table 8.2 Clinical chemistry laboratory variables over time (Safety Analysis Population)

Table 9.1 Weight and vital signs variables over time (Safety Analysis Population)

Table 10.1 Electrocardiogram variables over time (Safety Analysis Population)

Anti-tumour activity

Table 12.1 Best objective response and objective response rate by CT7001 dose group (Evaluable for Anti-tumour Activity Analysis Population)

Table 12.2 Clinical benefit rate and stable disease by CT7001 dose group (Evaluable for Anti-tumour Activity Analysis Population)

Table 12.3 Durable response rate and durability of response by CT7001 dose group (Evaluable for Anti-tumour Activity Analysis Population)

Table 12.4 Non-progressive disease summary by CT7001 dose group (Evaluable for Anti-tumour Activity Analysis Population)

Table 12.5 Percentage change in tumour size summary by CT7001 dose group and visit (Evaluable for Anti-tumour Activity Analysis Population)

Table 12.6 Best percentage change in tumour size by CT7001 dose group (Evaluable for Anti-tumour Activity Analysis Population)

Table 12.7 Progression-free survival by CT7001 dose group (Evaluable for Anti-tumour Activity Analysis Population)

Table 12.8 Overall survival summary by CT7001 dose group (Evaluable for Anti-tumour Activity Analysis Population)

Pharmacokinetics

Table 13.1 Single dosing – plasma concentrations, summary by CT7001 dose group (PK Analysis Population)

Table 13.2 Multiple dosing – plasma concentrations, summary by CT7001 dose group (PK Analysis Population)

Table 13.3 Multiple dosing – trough plasma concentrations of CT7001 (ng/mL), summary by CT7001 dose group (PK Analysis Population)

Table 14.1 Single dosing – PK Parameters, summary by CT7001 dose group (PK Analysis Population)

Table 14.2 Multiple dosing – PK Parameters, summary by CT7001 dose group (PK Analysis Population)

Listings

Baseline and Other Subject-Specific Characteristics

Listing 1.1 Subject disposition (All subjects)

Listing 1.2 Protocol deviations (All subjects)

Listing 2.1 Prior, concomitant and post-treatment medications (Safety Analysis Population)

Safety Data

Listing 3.1 Study drug administration (Safety Analysis Population)

Listing 3.2 Study drug accountability (Safety Analysis Population)

Listing 4.1 Adverse events (Safety Analysis Population)

Listing 4.2 Adverse events leading to death (Safety Analysis Population)

Listing 4.3 Serious adverse events (Safety Analysis Population)

Listing 4.4 Adverse events of special interest (Safety Analysis Population)

Listing 4.5 Adverse events – Dose Limiting Toxicities (Safety Analysis Population - Multiple ascending dose cohort)

Listing 4.6 Adverse events leading to discontinuation of IP (Safety Analysis Population)

Listing 5.1 Haematology (Safety Analysis Population)

Listing 5.2 Clinical chemistry (Safety Analysis Population)

Listing 5.3 Urinalysis (Safety Analysis Population)

Listing 6.1 Weight and vital signs (Safety Analysis Population)

Listing 7.1 Electrocardiogram – individual results (Safety Analysis Population)

Listing 7.2 Electrocardiogram – finding/interpretation (Safety Analysis Population)

Listing 8.1 ECOG status (Safety Analysis Population)

Listing 9.1 Abnormal physical examination (Safety Analysis Population)

<u>Anti-tumour Activity</u>

Listing 10.1 Tumour lesion details and measurements (Evaluable for Anti-tumour Activity Analysis Population)

Listing 10.2 Individual efficacy response data (Evaluable for Anti-tumour Activity Analysis Population)

Listing 10.3 Duration of response and best objective response (Evaluable for Anti-tumour Activity Analysis Population)

Listing 10.4 Progression-free survival and overall survival (Evaluable for Anti-tumour Activity Analysis Population)

<u>Pharmacokinetics</u>

Listing 11.1 Single dosing – individual subject plasma concentrations of CT7001 (PK Analysis Population)

Listing 11.2 Multiple dosing – individual subject plasma concentrations of CT7001 (PK Analysis Population)

Listing 12.1 Single dosing – Individual PK parameters (PK Analysis Population)

Listing 12.2 Multiple dosing – Individual PK parameters (PK Analysis Population)

Figures

<u>Pharmacokinetics</u>

Figure 1.1.1 Single dosing – Individual plasma pharmacokinetic concentrations up to 48H or 168H by actual time and dose group, Linear/Linear scale (PK Analysis Population)

Figure 1.1.2 Single dosing - Individual plasma pharmacokinetic concentrations up to 48H or 168H by actual time and dose group, Log/Linear scale (PK Analysis Population)

Figure 1.2.1 Multiple dosing – Individual plasma pharmacokinetic concentrations up to 24H (Cycle 2 Day 1) by actual time and dose group, Linear/Linear scale (PK Analysis Population)

Figure 1.2.2 Multiple dosing – Individual plasma pharmacokinetic concentrations up to 24H (Cycle 2 Day 1) by actual time and dose group, Log/Linear scale (PK Analysis Population)

Figure 2.1.1 Single dosing – Geometric Mean plasma pharmacokinetic concentrations up to 168H (Cycle 0 Day 1) by nominal time and dose group, Linear/Linear Scale, Geometric mean ±SD (PK Analysis Population)

Figure 2.1.2 Single dosing – Geometric Mean plasma pharmacokinetic concentrations up to 168H (Cycle 0 Day 1) by nominal time and dose group, Log/Linear Scale, Geometric mean ±SD (PK Analysis Population)

Figure 2.1.3 Single dosing – Geometric mean plasma pharmacokinetic concentrations up to 168H (Cycle 0 Day 1) by nominal time and dose group, Linear/Linear Scale (PK Analysis Population)

Figure 2.1.4 Single dosing – Geometric mean plasma pharmacokinetic concentrations up to 168H (Cycle 0 Day 1) by nominal time and dose group, Log/Linear Scale (PK Analysis Population)

Figure 2.2.1 Multiple dosing – Geometric Mean plasma pharmacokinetic concentrations up to 24H (Cycle 2 Day 1) by nominal time and dose group, Linear/Linear Scale, Geometric mean ±SD (PK Analysis Population)

Figure 2.2.2 Multiple dosing – Geometric Mean plasma pharmacokinetic concentrations up to 24H (Cycle 2 Day 1) by nominal time and dose group, Log/Linear Scale, Geometric mean ±SD (PK Analysis Population)

Figure 2.2.3 Multiple dosing - Geometric Mean plasma pharmacokinetic concentrations up to 24H (Cycle 2 Day 1) by nominal time and dose group, Linear/Linear Scale

Figure 2.3.4 Multiple dosing - Geometric Mean plasma pharmacokinetic concentrations up to 24H (Cycle 2 Day 1) by nominal time and dose group, Log/Linear Scale

Figure 3.1 Mean trough plasma pharmacokinetic concentrations by nominal time and dose group, Geometric mean ±SD, Linear/Linear Scale (PK Analysis Population)

Figure 3.2 Mean trough plasma pharmacokinetic concentrations by nominal time and dose group, Geometric mean ±SD, Log/Linear Scale (PK Analysis Population)

Figure 4.1.1 Single dosing AUC_{0-24}(unit) – Individual data and geometric mean, versus CT7001 dose (PK Analysis Population)

Figure 4.1.2 Single dosing Cmax (unit) – Individual data and geometric mean, versus CT7001 dose (PK Analysis Population)

Figure 4.2.1 Multiple dosing AUC_{tau} (unit) – Individual data and geometric mean, versus CT7001 dose (PK Analysis Population)

Figure 4.2.2 Multiple dosing Css,max (unit) – Individual data and geometric mean, versus CT7001 dose (PK Analysis Population)

Figure 5.1.1 Single dosing - box-plot of dose normalised AUC_{0-24} versus CT7001 dose (PK Analysis Population)

Figure 5.1.2 Single dosing – box-plot of dose normalised Cmax versus CT7001 dose (PK Analysis Population)

Figure 5.2.1 Multiple dosing – box-plot of dose normalised AUC_{tau} versus CT7001 dose (PK Analysis Population)

Figure 5.2.2 Multiple dosing – box-plot of dose normalised Css, max versus CT7001 dose (PK Analysis Population)

Anti-tumour Activity

Figure 6.1 Response profile for each subject – Swimmer plot (Evaluable for Anti-tumour Activity Analysis Population)

Figure 6.2 Best percentage change from baseline in tumour size – Waterfall plot (Evaluable for Anti-tumour Activity Analysis Population)

Figure 6.3 Percentage change from baseline in tumour size, individual subject data over time (Evaluable for Anti-tumour Activity Analysis Population)

Certificate Of Completion

Envelope Id: CAC02CD506334DB5A9EC9867EB9BF7E9
Subject: M1A SAP: Please DocuSign: CT7001-001 SAP Module1_PartA Final v2.4.pdf
Source Envelope:
Document Pages: 41
Certificate Pages: 5
AutoNav: Enabled
EnvelopeId Stamping: Disabled
Time Zone: (UTC) Dublin, Edinburgh, Lisbon, London

Signatures: 2
Initials: 0

Status: Completed

Envelope Originator:
[redacted]
65 Knowl Piece
Wi bury Way
Hitchin, Hertfordshire SG4 0TY
[redacted]

Record Tracking

Status: Original<br>22-Oct-20 | 09:13	Holder: [redacted]	Location: DocuSign

Signer Events	Signature	Timestamp
[redacted]<br>Senior Medical Director<br>Security Level: Email, Account Authentication (Required), Access Code<br><br>**Electronic Record and Signature Disclosure:**<br>Accepted: 01-Oct-20 | 12:09<br>ID: 726a003c-f209-4c5d-91c4-e40caf870c0e	[redacted]<br>Signature Adoption: Pre-selected Style<br>Signature ID:<br>94C0D49A-78C7-48F0-9D99-8E746FA969BE<br>[redacted]<br>With Signing Authentication via DocuSign password<br>With Signing Reasons (on each tab):<br>I approve this document	Sent: 22-Oct-20 | 09:16<br>Viewed: 23-Oct-20 | 11:53<br>Signed: 23-Oct-20 | 11:55
[redacted]<br>Security Level: Email, Account Authentication (Required), Access Code<br><br>**Electronic Record and Signature Disclosure:**<br>Accepted: 09-Oct-20 | 16:08<br>ID: 43020772-c0bb-4a89-b8e2-35ed29368e28	[redacted]<br>Signature Adoption: Pre-selected Style<br>Signature ID:<br>3042C6FE-F19F-4AA7-B560-9885D9BFC35E<br>[redacted]<br>With Signing Authentication via DocuSign password<br>With Signing Reasons (on each tab):<br>I am the author of this document	Sent: 22-Oct-20 | 09:16<br>Viewed: 22-Oct-20 | 10:13<br>Signed: 22-Oct-20 | 10:14

In Person Signer Events	Signature	Timestamp

Editor Delivery Events	Status	Timestamp

Agent Delivery Events	Status	Timestamp

Intermediary Delivery Events	Status	Timestamp

Certified Delivery Events	Status	Timestamp

Carbon Copy Events	Status	Timestamp
[redacted]<br>Clinical Project Associate<br>Bionical Emas<br>Security Level: Email, Account Authentication (Required)<br>**Electronic Record and Signature Disclosure:**<br>Not Offered via DocuSign	COPIED	Sent: 22-Oct-20 | 14:07

Witness Events	Signature	Timestamp

Notary Events	Signature	Timestamp

Envelope Summary Events	Status	Timestamps
Envelope Sent	Hashed/Encrypted	22-Oct-20 | 14:07
Certified Delivered	Security Checked	23-Oct-20 | 11:53
Signing Complete	Security Checked	23-Oct-20 | 11:55
Completed	Security Checked	23-Oct-20 | 11:55

Payment Events	Status	Timestamps

Electronic Record and Signature Disclosure

ELECTRONIC RECORD AND SIGNATURE DISCLOSURE

From time to time, Bionical Emas (we, us or Company) may be required by law to provide to you certain written notices or disclosures. Described below are the terms and conditions for providing to you such notices and disclosures electronically through the DocuSign system. Please read the information below carefully and thoroughly, and if you can access this information electronically to your satisfaction and agree to this Electronic Record and Signature Disclosure (ERSD), please confirm your agreement by selecting the check-box next to ‘I agree to use electronic records and signatures’ before clicking ‘CONTINUE’ within the DocuSign system.

Getting paper copies

At any time, you may request from us a paper copy of any record provided or made available electronically to you by us. You will have the ability to download and print documents we send to you through the DocuSign system during and immediately after the signing session and, if you elect to create a DocuSign account, you may access the documents for a limited period of time (usually 30 days) after such documents are first sent to you. After such time, if you wish for us to send you paper copies of any such documents from our office to you, you will be charged a $0.00 per-page fee. You may request delivery of such paper copies from us by following the procedure described below.

Withdrawing your consent

If you decide to receive notices and disclosures from us electronically, you may at any time change your mind and tell us that thereafter you want to receive required notices and disclosures only in paper format. How you must inform us of your decision to receive future notices and disclosure in paper format and withdraw your consent to receive notices and disclosures electronically is described below.

Consequences of changing your mind

If you elect to receive required notices and disclosures only in paper format, it will slow the speed at which we can complete certain steps in transactions with you and delivering services to you because we will need first to send the required notices or disclosures to you in paper format, and then wait until we receive back from you your acknowledgment of your receipt of such paper notices or disclosures. Further, you will no longer be able to use the DocuSign system to receive required notices and consents electronically from us or to sign electronically documents from us.

All notices and disclosures will be sent to you electronically

Unless you tell us otherwise in accordance with the procedures described herein, we will provide electronically to you through the DocuSign system all required notices, disclosures, authorizations, acknowledgements, and other documents that are required to be provided or made available to you during the course of our relationship with you. To reduce the chance of you inadvertently not receiving any notice or disclosure, we prefer to provide all of the required notices and disclosures to you by the same method and to the same address that you have given us. Thus, you can receive all the disclosures and notices electronically or in paper format through the paper mail delivery system. If you do not agree with this process, please let us know as described below. Please also see the paragraph immediately above that describes the consequences of your electing not to receive delivery of the notices and disclosures electronically from us.

How to contact Bionical Emas:

You may contact us to let us know of your changes as to how we may contact you electronically, to request paper copies of certain information from us, and to withdraw your prior consent to receive notices and disclosures electronically as follows:
To contact us by email send messages to: DISTDPO@Bionical-Emas.com
To contact us by paper mail, please send correspondence to:
Bionical Emas
63-65 Knowl Piece
Wilbury Way
Hitchin, Hertfordshire, United Kingdom, SG4 0TY

To advise Bionical Emas of your new email address

To let us know of a change in your email address where we should send notices and disclosures electronically to you, you must send an email message to us at DISTDPO@Bionical-Emas.com and in the body of such request you must state: your previous email address, your new email address. We do not require any other information from you to change your email address.

If you created a DocuSign account, you may update it with your new email address through your account preferences.

To request paper copies from Bionical Emas

To request delivery from us of paper copies of the notices and disclosures previously provided by us to you electronically, you must send us an email to DISPDPO@Bionical-emas.com and in the body of such request you must state your email address, full name, mailing address, and telephone number. We will discuss any anticipated fees and how these are collected with you.

To withdraw your consent with Bionical Emas

To inform us that you no longer wish to receive future notices and disclosures in electronic format you may:

i. decline to sign a document from within your signing session, and on the subsequent page, select the check-box indicating you wish to withdraw your consent, or you may;

ii. send us an email to DISTDPO@Bionical-Emas.com and in the body of such request you must state your email, full name, mailing address, and telephone number. We do not need any other information from you to withdraw consent.. The consequences of your withdrawing consent will be discussed with you..

Required hardware and software

The minimum system requirements for using the DocuSign system may change over time. The current system requirements are found here: https://support.docusign.com/guides/signer-guide-signing-system-requirements.

Acknowledging your access and consent to receive and sign documents electronically

To confirm to us that you can access this information electronically, which will be similar to other electronic notices and disclosures that we will provide to you, please confirm that you have read this ERSD, and (i) that you are able to print on paper or electronically save this ERSD for your future reference and access; or (ii) that you are able to email this ERSD to an email address where you will be able to print on paper or save it for your future reference and access. Further, if you consent to receiving notices and disclosures exclusively in electronic format as described herein, then select the check-box next to 'I agree to use electronic records and signatures' before clicking 'CONTINUE' within the DocuSign system.

By selecting the check-box next to 'I agree to use electronic records and signatures', you confirm that:

- You can access and read this Electronic Record and Signature Disclosure; and
- You can print on paper this Electronic Record and Signature Disclosure, or save or send this Electronic Record and Disclosure to a location where you can print it, for future reference and access; and
- Until or unless you notify Bionical Emas as described above, you consent to receive exclusively through electronic means all notices, disclosures, authorizations, acknowledgements, and other documents that are required to be provided or made available to you by Bionical Emas during the course of your relationship with Bionical Emas.

MODULE 2A STATISTICAL ANALYSIS PLAN

Title: Statistical Analysis Plan for CT7001_001 Module 2A

Compound Name/Number: Samuraciclib/CT7001

Effective Date: 10-Aug-2022

Description: A Phase 1/2 study of CT7001 in Combination with Fulvestrant in Patients with Metastatic or Locally Advanced Hormone-Receptor-Positive and Human Epidermal Growth Factor Receptor 2-Negative Breast Cancer.

Subject: Oncology

Authors' Names, Titles and Functional Areas: [redacted], *Project Statistician, Biostatistics*
[redacted], *Project Statistician, Biostatistics*
[redacted], *Project Statistician, Biostatistics*

Copyright © 2022 PHASTAR. All rights reserved.

Unauthorised copying or use of this information is prohibited.

SIGNATURE PAGES

The signatories on the following pages have all read and approved this present version of the document.

Approved by:

Name:	[redacted]
Title:	Dr
Function:	Senior Medical Director

I certify that I have read this version of the Statistical Analysis Plan and approve its contents.

Signed:	DocuSigned by: [redacted] Signing Reason: I approve this document Signing Time: 10-Aug-2022 | 17:07 BST 94C0D49A78C748F09D998E746FA969BE	Date:	10-Aug-2022 | 17:07 BST

Name:	[redacted]
Title:	Mr
Function:	Project Statistician

I certify that I have read this version of the Statistical Analysis Plan and approve its contents.

Signed:	DocuSigned by: [redacted] Signing Reason: I am the author of this document Signing Time: 10-Aug-2022 | 14:06 BST 2746D8C16A104C14BA656CAB6108B8E2	Date:	10-Aug-2022 | 14:06 BST

SIGNATURE PAGES 2

ABBREVIATIONS 6

REVISION HISTORY 9

1. INTRODUCTION 10

2. STUDY OBJECTIVES AND ENDPOINTS 10

2.1 Study objectives 10

2.1.1 Primary objectives 10

2.1.2 Secondary objectives 10

2.2 Study endpoints 10

2.2.1 Primary endpoints 10

2.2.2 Secondary endpoints 11

2.3 Statistical hypotheses 11

2.4 Exploratory objectives, endpoints and hypotheses 11

2.4.1 Exploratory objectives 11

3. STUDY DESIGN 12

4. TIMING OF PLANNED ANALYSES 14

5. SAMPLE SIZE CONSIDERATIONS 14

6. ANALYSIS POPULATIONS 14

6.1 Intent-to-Treat (ITT) Population 14

6.2 As-Treated (AT) Population 14

6.3 Evaluable for Response Population 15

6.4 Pharmacokinetics (PK) Population 15

7. PROTOCOL DEVIATIONS 15

8. GENERAL CONSIDERATIONS FOR DATA ANALYSES 15

8.1 Standard summary statistics 15

8.2 Baseline definition 16

9. DATA HANDLING CONVENTIONS 16
9.1 Premature withdrawal and missing data 16
9.2 Derived and transformed data 16
9.2.1 Study Population 16
9.2.2 Safety Derivations 17
9.2.3 Efficacy Derivations 19
9.2.4 Pharmacokinetics 22
9.2.5 Biomarkers 22
9.3 Assessment time windows 22
10. STATISTICAL ANALYSES AND METHODOLOGY 22
10.1 Study population 22
10.1.1 Disposition of subjects 22
10.1.2 Protocol deviations 22
10.1.3 Demographic and baseline characteristics 23
10.1.4 Treatment compliance 23
10.1.5 Extent of exposure 23
10.2 Safety analyses 24
10.2.1 Adverse events 24
10.2.2 Clinical laboratory evaluations 25
10.2.3 Electrocardiogram (ECG) 25
10.2.4 Other safety evaluations 25
10.3 Efficacy analyses 25
10.3.1 Objective response (OR) 25
10.3.2 Duration of response (DOR) 26
10.3.3 Clinical benefit rate (CBR) 26
10.3.4 Percentage change in tumour size 26
10.3.5 Progression-free survival 26
10.4 Clinical Pharmacology 26
10.4.1 Pharmacokinetics 26
11. CHANGES FROM THE PROTOCOL-SPECIFIED ANALYSIS 27

12. REFERENCES 28

13. APPENDIX A – LIST OF TABLES, LISTINGS AND FIGURES.. 28

ABBREVIATIONS

AE	Adverse Event
ALT	Alanine aminotransferase
AST	Aspartate aminotransferase
AT	As-Treated
ATP	Adenosine triphosphate
BC	Breast cancer
BLQ	Below limit of quantification
BMI	Body Mass Index
CBR	Clinical benefit rate
CDK7	Cyclin-dependent-kinase 7
CI	Confidence interval
CMH	Cohran-Mantel-Haenszel
c-myc	Proto-oncogene
CR	Complete Response
CSP	Clinical Study Protocol
CTCAE	Common Terminology Criteria for Adverse Events
CV	Coefficient of variation
CYP	Cytochrome P450
DLT	Dose-limiting toxicity
DMC	Data Monitoring Committee
DOR	Duration of response
ECG	Electrocardiogram
ECOG	Eastern Cooperative Oncology Group
eCRF	Electronic Case Report Form
EORTC QLQ-C30	European Organisation for Research and Treatment of Cancer Quality of Life Questionnaire – Core 30 items

EORTC QLQ-BR23	European Organisation for Research and Treatment of Cancer Quality of Life Questionnaire – Breast cancer module
EQ-5D-5L	European Quality of Life – 5 Dimensions – 5 Levels
ER	Estrogen receptor
EuroQoL	European Quality of Life
GCV	Geometric coefficient of variation
HER2	Human epidermal growth factor receptor 2
HR	Hazard ratio
ICF	Informed Consent Form
IDMC	Independent Data Monitoring Committee
IM	Intramuscularly
ITT	Intent-to-Treat
KM	Kaplan-Meier
LHRH	Luteinizing hormone-releasing hormone
LLOQ	Lower limit of quantification
MedDRA	Medical Dictionary for Regulatory Activities
NE	Not Evaluable
NQ	Non-quantifiable
OD	Once daily
OR	Objective response
ORR	Objective response rate
PBMC	Peripheral blood mononuclear cells
PID	Percentage intended dose
PD	Progression of Disease
PFS	Progression-Free Survival
PgR	Progesterone receptor
PK	Pharmacokinetics

PR	Partial Response
PR2D	Preliminary recommended Phase 2 dose
PRO	Patient Reported Outcomes
PT	Preferred Term
QoL	Quality of life
QTcF	Fridericia formula
Rb	Retinoblastoma
RDI	Relative dose intensity
RECIST	Response Evaluation Criteria in Solid Tumours
RNA	Ribonucleic acid
RP2D	Recommended Phase 2 Dose
SAE	Serious adverse event
SAP	Statistical analysis plan
SD	Stable Disease
SOC	System Organ Class
TEAE	Treatment-emergent adverse event
TL	Target Lesion
TNBC	Triple-negative breast cancer

Trademark Information

SAS	SAS (Statistical Analysis Software) is a registered trademark of SAS Institute Inc.

REVISION HISTORY

Version	Date	Summary of revisions
1.0	18-Mar-2019	Initial version
2.0	22-Jun-2021	1. Updated CSP version throughout to v15.0 (14-Jun-2021) and removed references to parts B and C.<br>2. Section 9.2.3.3 Progression-free survival: Updated missed visit rule based on changes to schedule of assessments outlined in Section 9.2.3.1.<br>3. Appendix A: Added additional Adverse Events tables to include event level summaries of AEs.
3.0	10-Aug-2022	1. Updated authors<br>2. Included references to Table 3.5, Listing 2.3 & 2.4 in appendix as well as changing hormonal therapy to endocrine therapy, along with references to all therapies (endocrine neoadjuvant/adjuvant/metastatic, chemotherapy neoadjuvant/adjuvant/metastatic, other novel therapy) within Section 10.1.3.

1. INTRODUCTION

CT7001_001 is a modular, multipart, multi-arm, open-label, Phase 1/2 study to evaluate the safety and tolerability of CT7001 alone and in combination with anti-cancer treatments in subjects with advanced malignancies. CT7001 is a small molecule, adenosine triphosphate (ATP) competitive, selective oral inhibitor of cyclin-dependent-kinase 7 (CDK7). A first-in-human modular Phase 1/2 clinical study was initiated in November 2017 (EudraCT number: 2017-002026-20; ClinicalTrials.gov identifier: NCT03363893). The single and multiple-ascending dose 360 mg given once daily (OD) as the preliminary recommended Phase 2 dose (PR2D) for further testing as monotherapy. Accordingly, this is the dose which has been taken to investigation in Part 1B of the Phase I study; Module 1B-1, an expansion cohort in patients with metastatic or recurrent triple-negative breast cancer (TNBC) and Module 1B-2, an expansion cohort in patients with castrate resistant prostate cancer (CRPC). A study of the effect of food on the bioavailability of CT7001 (Module 4) has completed recruitment and the primary endpoint analysis indicated no significant effect of fed versus fasted on the PK AUC.

The present study (Module 2) is the first effort to evaluate CT7001 in combination with fulvestrant in subjects with locally advanced or metastatic hormone receptor-positive (HR-positive) and human epidermal growth factor receptor 2-negative (HER2-negative) breast cancer (BC). Module 2 consists of 3 study parts.

This statistical analysis plan (SAP) provides details of the summaries and analyses to be performed to report the findings of the study, specifically for Module 2, Part A. It should be read in conjunction with the Clinical Study Protocol; (CSP) CT7001_001, v15.0 (14-Jun-2021).

2. STUDY OBJECTIVES AND ENDPOINTS

2.1 STUDY OBJECTIVES

2.1.1 PRIMARY OBJECTIVES

- To determine the recommended Phase 2 dose of CT7001 given in combination with fulvestrant at 500 mg.

2.1.2 SECONDARY OBJECTIVES

- To evaluate safety and tolerability.
- To evaluate the trough concentrations of CT7001 when used in combination with fulvestrant compared to historical CT7001 data.
- To evaluate correlations between CT7001 exposures and efficacy/safety findings in the subject population.
- To evaluate the incidence and type of genotypes and haplotype variation of CYP2D6 and other CYP genes and of drug transporter genes that may be involved in the metabolism of CT7001.

2.2 STUDY ENDPOINTS

2.2.1 PRIMARY ENDPOINTS

Primary endpoints specific to Module 2 Part A are as follows:

- Dose-limiting toxicities and type, incidence, severity (as graded by CTCAE v5.0), seriousness and relationship to study medications of adverse events and any laboratory abnormalities.

Primary endpoints common to all CT7001_001 study modules:

- Adverse events (AEs)
- Clinical laboratory results (haematology, serum chemistry, coagulation, urinalysis)
- Physical examination findings
- Eastern Cooperative Oncology Group (ECOG) performance status
- Electrocardiogram (ECG) parameters (heart rate, PR interval, QRS complex, QT interval and QTcF)
- Weight
- Vital signs (supine systolic and diastolic blood pressure and pulse, temperature, respiratory rate, oxygen saturation)

2.2.2 SECONDARY ENDPOINTS

- Objective Response
- Duration of Response
- Clinical Benefit Rate (complete or partial response, or stable disease ≥ 24 weeks)
- Best percent change in tumour size
- Progression free survival
- Trough plasma concentration of CT7001
- Trough plasma concentrations of fulvestrant
- Incidence and type of genotypes and haplotype variation of CYP2D6, other CYP genes and drug transporter genes that may be involved in the metabolism of CT7001

2.3 STATISTICAL HYPOTHESES

No formal statistical hypothesis testing will be undertaken for Module 2, Part A.

2.4 EXPLORATORY OBJECTIVES, ENDPOINTS AND HYPOTHESES

2.4.1 EXPLORATORY OBJECTIVES

Exploratory objectives are listed below. Any analyses relating to these is outside the scope of this SAP, unless otherwise stated.

- To further investigate the presence and identity of metabolites of CT7001 and, if appropriate, characterise their PK.
- To further evaluate the impact of subject characteristics (including but not limited to race, ethnicity, age, CYP and drug transporter polymorphisms) on trough concentrations of CT7001.

- To evaluate correlations between CT7001 exposures and efficacy/safety finding in this patient population.
- To further explore mutations and expression in genes, proteins and RNAs relevant to the cell cycle (e.g. phosphorylation of CDK1 and Rb proteins), drug target engagement (e.g. c-Myc, MCL-1) and tumour sensitivity and/or resistance in tumour-derived materials including circulating tumour DNA and tumour tissue (e.g. p53, CDK7, ER, ESR1, AR, PIk3CA) and their potential impact on efficacy.

3. STUDY DESIGN

The present study is an international, multi-centre Phase 1/2 study in subjects with metastatic or locally advanced HR-positive and HER2-negative BC. The study will have three parts. In each part, subjects must meet all the eligibility criteria described in Volume 4 Section 4 of the Clinical Study Protocol (CSP) CT7001_001, v15.0 (14-Jun-2021) and will receive study treatment until objective disease progression, symptomatic deterioration, unacceptable toxicity, death, withdrawal of consent or completion of Module 2 endpoint, whichever occurs first.

The overall study design is illustrated in the following schema.

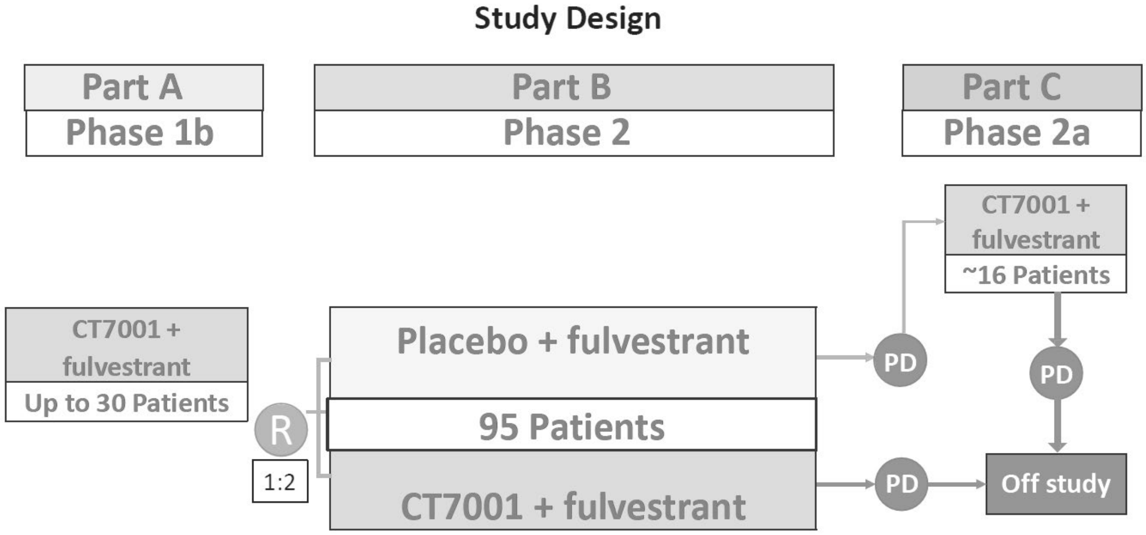

In each part of the study a treatment cycle will be defined as 28 days. CT7001 (or placebo) will be administered orally OD.

Module 4 evaluated the effect of food on the bioavailability of CT7001 in cancer patients. On 10th June 2019 the Safety Review Committee (SRC) reviewed the PK data and determined there was no significant effect observed in AUC in the fed phase of the Module 4, therefore the requirement to fast was removed. CT7001 may be taken orally in either a fed or fasted state. Where a patient

experiences nausea or vomiting investigators are recommended to advise their patients to consume CT7001 after a meal.

Throughout the study the dose of fulvestrant will be fixed at the standard dose of 500 mg administered IM at intervals of 28 ± 2 days with an additional 500 mg given 14 ± 2 days after the first dose. Pre- and peri-menopausal women must have commenced treatment with an LHRH agonist at least 4 weeks prior to first dose of CT7001.

The DMC will monitor safety data on a periodic basis. The DMC will also review the efficacy data on a periodic basis.

Subjects will undergo regular safety and efficacy assessments as outlined in the Schedule of Events. Primary efficacy analyses will be performed based on the local radiologist's/investigator's tumour assessments, using Response Evaluation Criteria in Solid Tumours (RECIST) version 1.1.

Tumour assessments will continue until radiographically and/or clinically (i.e. for photographed or palpable lesions) documented progressive disease as per RECIST version 1.1, death, discontinuation of subject from overall study participation (e.g. subject's request, lost to follow-up) or initiation of new anticancer therapy, whichever occurs first.

All parts of the study include sparse blood sampling for analysis of CT7001 and fulvestrant trough concentrations. Genotyping of CYP and drug transporter genes will also be considered a mandatory study procedure.

All parts of the study will include a set of optional procedures and analyses which require separate informed consent, details of which can be found in the Clinical Study Protocol (CSP) CT7001_001, v15.0 (14-Jun-2021).

Part A is an open-label, single-arm, ascending dose Phase 1b study to determine the dosing regimen of CT7001 and fulvestrant to be taken to subsequent randomised Phase 2 testing in Part B. Before taking a particular dosing regimen to Part B, at least 6 subjects in Part A should have received that regimen and at least 3 subjects should have completed ≥ 2 cycles.

Part A is planned to have 2 dose cohorts with up to 6 evaluable subjects to be enrolled per cohort. In each cohort, the dose of fulvestrant will be fixed at the standard dose of 500 mg given at intervals of 28 days ± 2 days with an additional 500 mg dose given 14 days ± 2 days after the first dose.

Cohort 1 will test CT7001 at 240 mg OD, which is approximately 33% lower than the preliminary recommended Phase 2 dose as monotherapy (360 mg). Cohort 2 is planned to test CT7001 at 360 mg OD. The decision algorithm below is based on dose-limiting toxicities (DLTs) recorded in the first cycle. Subjects are considered evaluable if they completed the first cycle or discontinued the first cycle due to DLT. Subjects in a cohort will be replaced if they cannot complete the first cycle unless due to a DLT.

1. If 1/3 subjects have a DLT in cohort 1, a further 3 evaluable subjects will be assessed.

2. If < 2/6 subjects have a DLT in cohort 1, cohort 2 (CT7001 at 360 mg and fulvestrant at 500 mg) will start enrolment.
3. If ≥ 2/3 or ≥ 2/6 evaluable subjects in cohort 1 experience a DLT, dose escalation must be stopped. The DMC will determine if subjects in Part A and in Part B can be administered a lower dose, or different dosing regimen. This would require approval of a substantial protocol amendment.
4. If 0/3 or 1/3 evaluable subjects in cohort 2 experience a DLT, the cohort will expand to 6 subjects.
5. If ≥ 2/3 or ≥ 2/6 subjects experience a DLT, the cohort 1 dosing regimen (CT7001 at 240 mg and fulvestrant at 500 mg) will be considered as the preliminary Phase 2 dosing regimen. Before taking to Part B, at least 6 subjects should be treated with that regimen in Part A and at least 3 should have completed 2 cycles.
6. If < 2/6 evaluable subjects in cohort 2 have a DLT and at least 3 subjects have completed at least 2 cycles, this will be considered the recommended Phase 2 regimen taken to Part B testing.

Part A completed enrolment on 23 March 2021 with 31 patients dosed. On 11 April 2021 the DMC confirmed that 360 mg OD is the Module 2 Part B starting dose.

4. TIMING OF PLANNED ANALYSES

Formal statistical analysis will not be performed. The DMC will monitor safety and efficacy data on a periodic basis. Final analysis by PHASTAR will occur post-database lock.

5. SAMPLE SIZE CONSIDERATIONS

The primary objective of Part A is to determine the recommended Phase 2 dose of CT7001 given in combination with fulvestrant. Module 2 Part A completed enrolment on 23 March 2021 with 6 and 25 subjects dosed in the 240mg OD and 360mg OD cohorts respectively.

6. ANALYSIS POPULATIONS

6.1 INTENT-TO-TREAT (ITT) POPULATION

The ITT Population will include all enrolled subjects with designated study drug assignment. The ITT population will be used for the baseline and demographic summaries and will be the primary population for PFS analyses. For all analyses using the ITT population, planned treatment will be used.

6.2 AS-TREATED (AT) POPULATION

The AT Population will include all subjects who received at least 1 dose of study treatment, with treatment assignments according to actual study treatment received.

- The AT Population will be the primary population for evaluating safety and treatment administration/compliance.

6.3 Evaluable for Response Population

The Evaluable for Response Population will include all subjects who received at least 1 dose of CT7001 and had measurable disease at baseline and had a post-baseline tumour assessment. The Evaluable Response Population population will be used for the OR and CBR analyses.

6.4 Pharmacokinetics (PK) Population

The PK Population will include all subjects who received at least one dose of CT7001 and fulvestrant, had at least one plasma concentration of CT7001 or fulvestrant above the lower limit of quantification and had no protocol deviations or other events that may impact PK analysis. The PK Population will serve as the PK analysis set and will be determined by Carrick prior to the final analysis.

7. PROTOCOL DEVIATIONS

Major protocol deviations will be captured and provided to summarise at the end of the study. A major protocol deviation is defined as a significant deviation i.e. one that may affect to a significant degree the safety or physical or mental integrity of the subjects of the study or the scientific value of the study or one which reflects a significant deviation from the standards of Good Clinical Practice (e.g. accidental release of confidential subject information). Protocol deviations will be recorded in the electronic case report form (eCRF).

In line with FDA guidance, the document “CT70001_001 Protocol Deviation Guidance During COVID-19 v2.0 29Jul2020.docx” details the process for recording and assessing COVID-19 related protocol deviations and alternate procedures during the pandemic. Any procedure that is conducted by an alternate location, process, method or schedule is added to the Alternate Procedure Tracker and will be provided for the CSR.

8. GENERAL CONSIDERATIONS FOR DATA ANALYSES

8.1 Standard summary statistics

Data analyses will be descriptive in nature. Unless otherwise specified, continuous variables will be summarised using n (number of non-missing observations), mean, standard deviation (SD), median minimum and maximum. Additionally, for the analyses of plasma concentrations, coefficient of variation (CV%), geometric mean, geometric SD and geometric CV% will be reported where applicable. Categorical variables will be summarised using the sample size (N), frequency count (n), and percentage, calculated relative to the total number of subjects in the relevant analysis population. All analyses and listings will use the observed data and missing values will not be imputed.

Summary statistics will be presented to the following level of precision (unless otherwise specified): mean and median to 1 more decimal place than raw data, standard deviation to 2 more decimal

places than raw data, and minimum and maximum to same precision as raw data. Percentages will be displayed to 1 decimal place.

All tables and listings of the data will be generated using the SAS System®, version 9.1 or later.

8.2 BASELINE DEFINITION

Unless otherwise specified, baseline will be defined as the last result prior to first dose of study treatment on Cycle 1 Day 1, based on the date and time of the assessment. In cases where the time of the assessment is not collected it will be assumed that it was performed prior to dosing on Cycle 1 Day 1 and will therefore not be considered as Baseline.

9. DATA HANDLING CONVENTIONS

9.1 PREMATURE WITHDRAWAL AND MISSING DATA

Only data recorded will be included in any tables, figures or listings. For subjects who withdraw from the study, all data compiled up to the point of discontinuation will be used for the analysis.

9.2 DERIVED AND TRANSFORMED DATA

9.2.1 STUDY POPULATION

9.2.1.1 DEMOGRAPHICS AND BASELINE CHARACTERISTICS

Age, in whole years, will be calculated with respect to the subject's screening visit (calculated in the eCRF).

Body Mass Index (BMI) will be calculated as Weight (kg) / Height (m)^2

9.2.1.2 CONCOMITANT MEDICATIONS

Medications will be classified as prior, concomitant, or post-treatment based on start and stop dates in relation to study treatment dates.

- Prior medications: those ending before the start date of study treatment.
- Concomitant medications: those taken at any time between the start date and stop date of study treatment, inclusive. Medications that started before study treatment but continued during this period are also considered as concomitant medications.
- Post-treatment medications: those started after the stop date of study treatment.

It will be assumed that medication has been taken on the date which is reported as started or stopped. For any medication starting on the same date as study treatment and where time medication is taken is missing, it will be assumed that the medication was taken after the subject started taking study treatment (i.e. concomitant). Any other situation where it cannot be determined whether medication was stopped prior to first dose of IP, i.e. missing month or year in a partial date, the medication will be assumed to be concomitant.

9.2.1.3 Duration of exposure

Number of days of exposure to study drug will be calculated as:

Duration of exposure in days = (Treatment stop date – Treatment start date) + 1

Total time on study will be calculated as:

Time on study in days = (Date of discontinuation – Treatment start date) + 1

9.2.1.4 Dose intensity

Relative dose intensity (RDI) is the percentage of actual dose received relative to the intended dose through to treatment discontinuation and percentage intended dose (PID) is the percentage of the actual dose received relative to the intended dose through to progression. RDI and PID will be defined as follows:

- RDI = 100% * d/D, where d is the actual cumulative dose delivered up to the actual last day of dosing and D is the intended cumulative dose up to the actual last day of dosing. D is the total dose that would be delivered, if there were no modification to dose or schedule.
- PID = 100% * d/D, where d is the actual cumulative dose delivered up to progression (or a censoring event) and D is the intended cumulative dose up to progression (or a censoring event, as defined in section 9.2.4.9). D is the total dose that would be delivered, if there were no modification to dose or schedule.

The entire intended treatment period will be used in the derivation of RDI and PID. RDI and PID will be the same for any subject where the last day of dosing is the same as the date of progression. The actual cumulative dose will be calculated based on subject dosing information as recorded in the eCRF. RDI and PID will be derived for CT7001 only.

9.2.2 Safety Derivations

9.2.2.1 Adverse events

All clinical AEs (serious and non-serious) occurring from the time the subject has taken at least one dose of investigational product through to the End of Study visit will be recorded on the eCRF. For serious adverse events the reporting period begins from the time the subject signs the informed consent form (ICF). If a subject begins a new anti-cancer therapy, the AE reporting period for non-serious AEs ends at the time the new treatment is started. Medical conditions that exist before signing the ICF will be recorded as part of medical history.

Adverse event assessment will include type, incidence, severity (graded by the National Cancer Institute Common Terminology Criteria for Adverse Events [NCI CTCAE], Version 5.0, timing, seriousness, and relatedness.

The Investigator will assess the relationship between the IP and each AE to determine whether the AE is considered to be causally related to IP. Causally related AEs are those with a relationship of 'Possibly Related', 'Probably Related' and 'Related'.

9.2.2.2 Serious adverse events

A serious adverse event (SAE) is an AE occurring during any study that fulfils one or more of the following criteria:

- Results in death
- Is immediately life-threatening
- Requires inpatient hospitalisation or prolongation of existing hospitalisation
- Results in persistent or significant disability/ incapacity or substantial disruption of the ability to conduct normal life functions
- Is or results in a congenital abnormality or birth defect
- Is an important medical event that may jeopardise the subject or may require medical intervention to prevent one of the outcomes listed above.

Life-threatening means that the subject was at immediate risk of death at the time of the SAE; it does not refer to a serious AE that hypothetically might have caused death if it were more severe.

All SAEs will be reported from consent until the End of Study visit (28-35 days after the last administration of the investigational product). An SAE that occurs after the End of Study visit and comes to the attention of the Investigator will be reported only if there is (in the opinion of the Investigator) reasonable causal relationship with the study drug.

9.2.2.3 Adverse events of special interest

For the purpose of this module, thrombocytopenia and low platelet counts will be the only adverse events of special interest analysed as per regulatory recommendation.

9.2.2.4 Clinical laboratory evaluations

Laboratory tests will include full blood counts, standard serum chemistry and urinalysis.

Haematology includes: haemoglobin, red blood cell count, haematocrit, mean cell volume, reticulocyte count (absolute particle count or relative particle count), white blood cell count with differential (absolute and percent counts of neutrophils, lymphocytes, monocytes, basophils, eosinophils) and platelet count.

Serum chemistry includes: HbA1c, alanine aminotransferase (ALT), aspartate aminotransferase (AST), gamma glutamyl transferase, alkaline phosphatase, bilirubin (total), creatine kinase, total protein, albumin, creatinine, urea nitrogen or urea, calcium (total), glucose, sodium, potassium, magnesium, chloride and phosphate.

Urinalysis includes: blood, glucose and protein.

All laboratory tests will be performed during screening within 28 days before allocation to study therapy, on Days 1 and 15 in the first cycle, on Day 1 in subsequent cycle, and at the end of treatment and end of study visits. An additional assessment is required on Day 15 of the second cycle. Laboratory tests do not need to be repeated on Day 1 Cycle 1 if performed within 7 days prior to allocation to study therapy.

All results will be entered into the eCRF, with Système International (SI) units as standard system of measurements. However, the eCRF will allow for reporting of a subset of laboratory tests in conventional units if participating study sites consider this helpful. All analyses of laboratory results will use SI units where applicable.

Laboratory results will be graded according to the NCI CTCAEv5.0 severity grade, where possible.

9.2.2.5 Electrocardiogram (ECG)

ECGs will be performed using a 12-lead tracing. ECG measurements will include heart rate, PR interval, QRS complex, QT interval and QTcF. Triplicate 12-lead ECGs will be performed during screening within four weeks before allocation to study therapy, approximately every four weeks in all cycles, and at the end of treatment and end of study visits. Triplicate ECGs do not need to be repeated on Day 1 Cycle 1 if performed within 7 days prior to allocation to study therapy.

The mean of the triplicates will be calculated at each visit. If an individual has more than three assessments at any one visit, then unless a reading should be discarded for technical reasons, the latest three readings will be used to calculate the mean of the triplicate.

9.2.2.6 Other safety assessments

A full physical examination and assessment of vital signs and performance status will be required at screening, on Day 1 in all cycles, and at the end of treatment and end of study visits. Examination and assessments do not need to be repeated on Day 1 of Cycle 1 if performed within 7 days prior to allocation to study therapy. An additional examination and assessment are required on Day 15 of the first two cycles.

A full physical examination includes examination of all major body systems, height (at screening only) and weight.

Vital signs include supine blood pressure, pulse rate, temperature and respiratory rate.

Performance status will be assessed according to The Eastern Cooperative Oncology Group (ECOG) performance status scale.

9.2.3 Efficacy Derivations

9.2.3.1 Tumour assessments

Tumour assessments will be performed as scheduled, regardless of treatment interruptions or cycle delays.

Screening/baseline tumour assessment by CT, MRI and clinical examination must be carried out within 28 days before allocation to study therapy. For bone scans which were performed as routine procedure, a period of 12 weeks prior to allocation to study therapy is accepted provided the requirements described in the protocol are met.

During the study treatment period and follow-up, the same method must be used for assessment of a given region (e.g. chest, abdomen, pelvis) as was used at baseline for tumour assessment in that location. Post-baseline tumour assessments will be performed every 8 weeks ± 7 days for the first year and then every 12 weeks ± 7 days from Cycle 1 Day 1. Bone scans (as applicable) will be repeated every 16 weeks ± 7 days from Cycle 1 Day 1. If measurable/evaluable bone lesions are identified at baseline by CT scan or MRI, CT scan or MRI will be repeated every 8 weeks (±7 days), using the same modality used to confirm the bone lesions at baseline, for the first year and then every 12 weeks (±7 days) in subsequent years (calculated from Cycle 1 Day 1).

Tumour assessments will continue until radiographically and/or clinically (i.e. for photographed or palpable lesions) documented PD as per RECIST v1.1, death, discontinuation of subject from overall study participation (e.g. subject's request, lost to follow-up) or initiation of new anti-cancer therapy, whichever occurs first.

Objective tumour response will be measured using RECIST Version 1.1. At each post-baseline assessment individual tumour response and overall visit response will be recorded as either complete response (CR), partial response (PR), stable disease (SD), progression of disease (PD) or not evaluable (NE).

All efficacy endpoints based on the radiological (and photographical where applicable) assessments of tumour burden (i.e. OR, DOR, CBR, PFS) will be derived using the local radiologist's/investigator's assessment.

9.2.3.2 Independent review of disease assessments

Given the high degree of historical correlation in determining PFS treatment effect by investigator assessment versus independent radiologic review, investigator assessment will be used for the primary determination and analysis of efficacy parameters in this study. Further details on the information to be forwarded for independent review can be found in the CSP.

9.2.3.3 Objective response (OR)

Objective response (OR) is defined as a CR or PR according to RECIST Version 1.1. A subject will be considered to have achieved an OR if the subject has a CR or PR according to RECIST v1.1 definitions. Otherwise, the subject will be considered as a non-responder in the OR rate analysis. Additionally, subjects with inadequate data for tumour response (e.g. no baseline or post-baseline assessment) will be considered as non-responders in the OR rate analysis. Confirmed and unconfirmed responses will be presented.

9.2.3.4 Duration of response (DOR)

Duration of response (DOR) is defined as the time from first documentation of objective tumour response (CR or PR) to the first documentation of objective tumour progression or to death due to any cause, whichever occurs first. DOR data will be censored on the date of last tumour assessment on study for subjects who do not have objective tumour progression and who do not die due to any cause while on study. DOR will only be calculated for the subgroup of subjects with an objective response.

9.2.3.5 Clinical benefit rate(CBR)

Clinical benefit rate (CBR) is defined as CR, PR, or SD lasting ≥ 24 weeks recorded in the time period between first dose and disease progression or death due to any cause. To include allowance for visit windows if an assessment confirms SD at 21 weeks or later then this will be treated as Clinical benefit. This allows for each of the 3 assessments at 8, 16 and 24 weeks being a week early.

9.2.3.6 Percentage change in tumour size

Percentage change in tumour size will be determined for subjects in the evaluable for response population and is derived at each timepoint by the percentage change from baseline in the sum of diameters of target lesions (TLs). The best percentage change in tumour size is defined as the value representing the largest decrease (or smallest increase) from baseline in tumour size. Baseline is defined as the lesion measurements recorded at screening. Timepoints will be derived from the dates of disease assessments and categorised according to the planned schedule.

9.2.3.7 Progression-free survival (PFS)

PFS is defined as the time from date of first dose to the date of the first documentation of objective progression of disease (PD) or death due to any cause, whichever occurs first. PFS data will be censored on the date of last evaluable tumour assessment on study for subjects who do not have objective tumour progression and who do not die while on study. Subjects lacking an evaluation of tumour response after first dose will have their PFS time censored on the date of first dose with a duration of 1 day.

However, if the subject progresses or dies after two or more missed visits, the subject will be censored at the date of the latest evaluable RECIST assessment. Given that assessments are scheduled every 8 weeks (± 7 days) in the first year and 12 weeks (± 7 days) thereafter, two missed visits would equate to 16 weeks (± 2 weeks), a total of 18 weeks in the first year and 24 weeks (± 2 weeks), a total of 26 weeks, for assessments occurring after more than 1 year on study. If the two missed visits occur over the period when the scheduled frequency of RECIST assessments changes from eight-weekly to twelve-weekly this will equate to 22 weeks (i.e. take the average of 8 and 12 weeks which gives 10 weeks and then apply the same rationale, hence 2 x 10 weeks + 1 week for an early assessment + 1 week for a late assessment = 22 weeks).

Additionally, subjects who start a new anti-cancer therapy prior to documented PD will be censored at the date of the last evaluable tumour assessment prior to the start of the new therapy.

The PFS time will always be derived based on scan/assessment dates not visit dates.

9.2.4 PHARMACOKINETICS

Blood samples for CT7001/placebo trough concentrations will be collected before dosing on Day 1 and Day 15 of Cycle 1, Day 1 in all subsequent cycles and at the end of treatment visit.

Blood samples for fulvestrant trough concentrations will be collected before injection on Day 1 and Day 15 of Cycle 1, on Day 1 in every other subsequent cycle and at the end of treatment visit.

As part of the understanding of PK behaviour and profile of CT7001, part of the blood samples may be used for metabolite identification. These data will be used for internal exploratory purposes only.

9.2.5 BIOMARKERS

Various tumour-derived materials will be collected to further explore the effect of mutations and expressions in certain genes, RNAs and proteins on sensitivity and/or resistance to CT7001.

Except for ER, PgR and HER2 status as part of screening, all biomarkers analyses and related procedures are considered optional. These analyses will be considered exploratory and fall outside the scope of this SAP.

9.3 ASSESSMENT TIME WINDOWS

Only data from nominal protocol-scheduled visits/time points will be included in the analyses, i.e. unscheduled and repeated tests will not be included. However, all data collected will be presented in the listings.

10. STATISTICAL ANALYSES AND METHODOLOGY

10.1 STUDY POPULATION

10.1.1 DISPOSITION OF SUBJECTS

Subject disposition will be summarised by cohort or arm for all subjects in the ITT Population. The number and percentage of subjects enrolled in the module part, subjects who received treatment, subjects ongoing or discontinuing treatment, and subjects ongoing or discontinuing the study module at the time of analysis will be presented in tabular format. The primary reasons for discontinuation will also be summarised.

A summary of analysis populations will be tabulated.

10.1.2 PROTOCOL DEVIATIONS

All important protocol deviations will be listed by subject for all subjects in the ITT Population. Listing will also show whether protocol deviations were related to COVID-19 or not.

10.1.3 DEMOGRAPHIC AND BASELINE CHARACTERISTICS

Demographic and baseline subject characteristics will be summarised for the ITT Population. Standard descriptive statistics will be presented for the continuous variables of age (years), weight at screening (kg), height (cm) and body mass index (kg/m^2) [calculated as weight (kg)/height (m)^2]. Categories of sex, race and ethnicity will be summarised.

Medical history will be coded using MedDRA and summarised for ITT Population by cohort/arm, system organ class and preferred term.

Breast cancer history at screening and prior chemotherapy and endocrine treatment details will be listed and summarised descriptively. Number and percentage of subjects receiving other prior treatments (endocrine therapy, radiotherapy, surgery, biological/immunological/other, novel therapies) will also be summarised by the following: endocrine neoadjuvant, chemotherapy neoadjuvant, endocrine adjuvant, chemotherapy adjuvant, endocrine metastatic, and chemotherapy metastatic.

Prior and concomitant medications will be coded using the most current World Health Organisation Drug Dictionary and summarised by cohort/arm, Anatomical Therapeutic Chemical level 3 and generic term. All prior, concomitant and post-treatment medications will be listed.

Additionally, incidence and type of genotypes and haplotype variation of CYP2D6 will be summarised. A list of the polymorphisms present for each subject will be listed.

Nicotine use will be summarised.

10.1.4 TREATMENT COMPLIANCE

Subjects will be required to return all bottles of CT7001/placebo as well as their completed patient diary at the beginning of each cycle for drug accountability. Drug accountability will be performed on Day 1 of every cycle prior to dispensing drug supply for the next cycle. The number of remaining capsules will be documented and recorded. This information will be listed for each subject in the AT Population.

10.1.5 EXTENT OF EXPOSURE

Exposure will be listed and summarised for the AT Population.

Total exposure (date of last dose minus date of first dose+1) and total time on study (date of discontinuation minus date of first dose+1) will be summarised descriptively by cohort/arm.

In addition, the number and percentage of subjects with at least 1 dose interruption/dose delay and at least 1 dose reduction of CT7001 will be presented. Number and percentage of subjects with at least 1 dose interruptions/delay of fulvestrant will also be summarised. Note that the fulvestrant dose cannot be adjusted.

RDI and PID of CT7001 will be summarised descriptively. In addition, the number and percentage of subjects will be summarised categorically (>= 80%, < 80%) for RDI and PID.

Cumulative exposure over time (days) and number of cycles completed will be summarised descriptively. A treatment cycle is defined as 28 days.

10.2 SAFETY ANALYSES

Safety analyses will be performed using the AT Population. All summaries by visit will use the visit recorded.

10.2.1 ADVERSE EVENTS

AEs will be listed individually by subject, including details of MedDRA system organ class, MedDRA preferred term, reported term, study day, start date/time, end date/time, duration, severity, outcome, relationship to study drug, and any action taken.

AE summary tables will include only treatment emergent AEs (TEAEs), defined as those AEs which occur from first dose of study treatment up to 28 days after the last dose of study treatment. Any AE that occurs before the first dose of study treatment or after the 28-day follow-up period will be listed only and not included in the summaries.

A subject level summary of TEAEs will be presented including the number and percentage of subjects in the following categories: any AE, AEs of CTCAE grade 3 or higher, AEs with outcome of death, SAEs, AEs leading to discontinuation of IP, dose-limiting toxicities (DLTs) and AEs causally related to study treatment.

The number of subjects experiencing each TEAE will be summarised by cohort/arm, MedDRA System Organ Class (SOC) and MedDRA Preferred Term (PT). In addition, the incidence rate per 100 subject years will be presented, calculated as the number of subjects with AEs divided by the total duration of treatment across all subjects in a given group, multiplied by 100.

The number and percentage of subjects experiencing each TEAE will also be summarised by cohort/arm, MedDRA SOC, MedDRA PT, and Common Terminology Criteria for Adverse Events (CTCAE) grade.

In addition, the number and percentage of subjects experiencing each TEAE will be summarised by cohort/arm, MedDRA SOC and MedDRA PT, separately for the following categories: AEs causally related to CT7001, AEs of special interest, DLTs, AEs with CTCAE grade >=3 and SAEs.

TEAEs will also be summarised by cycle, SOC and PT. Cycle will be assigned based on the reported start date of the AE.

Details of any deaths, SAEs, AEs with outcome of death, AEs of special interest, DLTs and AEs leading to study discontinuation will be listed separately.

10.2.2 Clinical laboratory evaluations

Haematology and serum chemistry data will be summarised by cycle and cohort/arm. Actual results and changes from baseline will be summarised descriptively for haematology and clinical chemistry, and frequencies of positive/negative urinalysis results will be tabulated. The laboratory results will be graded according to the NCI CTCAE v5.0 severity grade. Frequencies of the worst severity grade observed will be displayed in a shift from baseline table by study treatment. For parameters for which a NCI CTCAE v5.0 scale does not exist, the frequency of subjects with values below, within, and above the normal ranges and shift from baseline will be summarised by cohort/arm.

All laboratory results will be listed.

10.2.3 Electrocardiogram (ECG)

ECG parameters, including finding/interpretation, will be listed individually by subject and the mean of the triplicates will be summarised descriptively by cycle and cohort/arm. Actual results and changes from baseline will be presented.

10.2.4 Other safety evaluations

Weight and vital signs will be listed individually by subjects. Weight will be summarised descriptively by cycle and cohort/arm. Actual results and changes from baseline will be presented.

All abnormal physical examination results will be listed by subject.

ECOG performance status will be listed individually by subject.

10.3 Efficacy analyses

Analyses of PFS will be based on the ITT population. Analyses of ORR, CBR, tumour size will be performed on the evaluable for response population.

10.3.1 Objective response (OR)

The OR rate (ORR) will be estimated by dividing the number of subjects with objective response (CR or PR) by the number of subjects in the evaluable for response population. An exact 95% CI will be computed using the Clopper-Pearson method.

Best objective response (BOR) will be determined for each subject based on the best response recorded from the start of study treatment to the end of treatment. BOR will be summarised for the number and percentage of subjects in each category of response (CR, PR, SD, PD, NE).

Swimmer plots which show the profile of each of the subject’s tumour response while on treatment will also be produced.

BOR and ORR will be calculated in the evaluable for response population using the investigator’s assessment.

10.3.2 Duration of response (DOR)

DOR will be summarised descriptively for subjects with an OR.

10.3.3 Clinical benefit rate (CBR)

The CBR will be estimated by dividing the number of subjects with CR, PR, or SD ≥ 24 weeks by the number of subjects in the evaluable for response population. An exact 95% CI for the CBR rate will be estimated using the Clopper-Pearson method.

10.3.4 Percentage change in tumour size

Percentage change in tumour size will be summarised by time since first dose. The best percent change versus baseline in post-baseline aggregate tumour size measurements will be summarised by treatment group and displayed graphically in the form of waterfall plots for the evaluable for response population.

10.3.5 Progression-free survival

Progression status at the time of analysis will be summarised descriptively for the ITT Population using the investigator assessment. Summary will include total number and percentage of subjects with an event (RECIST progression or death in the absence of progression), number and percentage of censored subjects (no RECIST progression or death, started a new-anti-cancer therapy, RECIST progression or death after 2 or more missed visits).

10.4 Clinical Pharmacology

10.4.1 Pharmacokinetics

Summary statistics for the PK analysis population will be provided for trough concentrations of CT7001 and fulvestrant. All subjects dosed will be listed with annotation detailing whether they are included or excluded from the PK analysis population individual plasma concentrations will also listed with annotation detailing whether they are included or excluded from the PK analysis. All subjects treated with CT7001 and fulvestrant for whom drug plasma concentration results (from at least 1 visit) are available will be summarised using appropriate statistics as listed below:

- The geometric mean (gmean, calculated as exp[μ], where μ is the mean of the data on a logarithmic scale)
- Geometric coefficient of variation (GCV, calculated as 100 √ [exp(s^2)-1], where s is the standard deviation of the data on the logarithmic scale)
- Geometric standard deviation (Geo_{SD}) calculated as exp(s)
- Geometric mean ±SD (calculated as exp[μ±s]) (plasma concentration only)
- Arithmetic mean calculated using untransformed data
- Coefficient of variation (CV%) using untransformed data
- Standard Deviation calculated using untransformed data

- Minimum
- Median
- Maximum
- Number of observation (n)

Summaries will be provided by cycle using the last dose prior to the PK blood sample to determine the dose level.

For plasma concentrations, listings will report concentrations to the same level of precision as the raw data and in the summaries, minimum and maximum will follow the raw data and all other summaries will be reported to 4 significant figures.

Reporting of descriptive statistics of the PK concentration data in the presence of below the limit of quantification (BLQ) observations will follow the rules below:

1. If, at a given nominal time point, 50% or fewer of the plasma concentrations are non-quantifiable (NQ), the geometric mean, geometric SD, geometric CV, arithmetic mean and arithmetic SD are calculated by substituting the lower limit of quantification (LLOQ) for values which are NQ.
2. If more than 50%, but not all, of the concentrations are NQ, the geometric mean, geometric SD, geometric CV, arithmetic mean and arithmetic SD are reported as not calculable (NC). The maximum concentration will be reported from the individual data, and the minimum and median will be set as NQ.
3. If all the concentrations are NQ, then all properties are reported as NQ.
4. The number of observations above LLOQ are reported for each time point along with the total number of observations.
5. Three observations > LLOQ are required as a minimum for a plasma concentration to be summarised.

Pharmacokinetic graphs

Plots of trough geometric mean concentration (x/÷ geometric SD) over time will be produced on the linear/linear and log/linear scale, cohort/arm overlaid on the same figure. For consistency the same plasma concentration values are used in the building of any mean data figures as those given in the descriptive statistics summary table for each cycle.

One figure per treatment group with all individual profiles overlaid on the same plot, as well as individual figures for each subject's concentration-time profile will also be produced.

For the individual figures, values reported as below the assay lower limit of quantification (LLOQ) will be set to LLOQ and for the mean figures time points where all concentrations are < LLOQ will be plotted at the assay LLOQ.

11. CHANGES FROM THE PROTOCOL-SPECIFIED ANALYSIS

Sections 2.2.2: Altered to include Progression Free Survival as a secondary endpoint.

12. REFERENCES

Clinical Study Protocol (CSP) CT7001_001, v15.0 (14-Jun-2021).

Osoba et al 1998
Osoba D, Rodrigues G, Myles J, Zee B, Pater J. Interpreting the significance of changes in health-related quality-of-life scores. J Clin Oncol. 1998 Jan;16(1):139-44.

13. APPENDIX A – LIST OF TABLES, LISTINGS AND FIGURES

Tables

Demographic, Baseline, Concomitant Medication and Other Subject-Specific Characteristics

Table 1.1 Subject disposition (All subjects)
Table 1.2 Analysis populations (All subjects)
Table 2.1 Demographic characteristics (ITT Population)
Table 3.1 Medical history (ITT Population)
Table 3.2 Breast cancer history at screening (ITT Population)
Table 3.3 Prior cancer treatment - Chemotherapy (ITT Population)
Table 3.4 Other prior cancer treatment (ITT Population)
Table 3.5 Other prior anti-cancer therapies (ITT Population)
Table 4.1 Prior medications (ITT Population)
Table 4.2 Concomitant medications (ITT Population)
Table 5.1 Summary of CYP2D6 genotypes (ITT Population)
Table 6.1 Nicotine use, categorised (ITT Population)

Safety Data

Table 7.1 Duration of exposure and cumulative exposure over time (AT Population)
Table 7.4 Dose intensity of CT7001 (AT Population)
Table 8.1.1 Adverse events in any category – subject level (AT Population)
Table 8.1.2 Adverse events in any category – event level (AT Population)
Table 8.2 Adverse events and incidence rate by system organ class and preferred term (AT Population)
Table 8.3 Adverse events by cycle, system organ class and preferred term (AT Population)
Table 8.4 Adverse events by system organ class, preferred term and maximum reported CTCAE grade (AT Population)
Table 8.5.1 Adverse events causally related to CT7001, by system organ class and preferred term (AT Population)
Table 8.5.2 Adverse events causally related to Fulvestrant, by system organ class and preferred term (AT Population)
Table 8.6 Adverse events of special interest, by system organ class and preferred term (AT Population)

Table 8.7 Adverse events classified as Dose Limiting Toxicities (DLT), by system organ class and preferred term (AT Population)
Table 8.8 Adverse events with CTCAE grade >= 3, by system organ class and preferred term (AT Population)
Table 8.9 Serious adverse events, by system organ class and preferred term (AT Population)
Table 9.1 Haematology laboratory variables over time (AT Population)
Table 9.2 Haematology, CTCAE grade change from baseline to maximum on treatment (AT Population)
Table 9.3 Haematology, normal ranges shift from baseline to post-baseline assessments (AT Population)
Table 9.4 Clinical chemistry laboratory variables over time (AT Population)
Table 9.5 Clinical chemistry, CTCAE grade change from baseline to maximum on treatment (AT Population)
Table 9.6 Clinical chemistry, normal ranges shift from baseline to post-baseline assessments (AT Population)

Table 10.1 Weight over time (AT Population)
Table 11.1 Electrocardiogram variables over time (AT Population)

Efficacy

Table 13.1 Best objective response and objective response rate (Evaluable for Response Population)
Table 13.2 Clinical benefit rate (Evaluable for Response Population)
Table 13.3 Duration of response (Evaluable for Response Population)
Table 13.4 Percentage change in tumour size over time (Evaluable for Response Population)
Table 13.5 Best percentage change in tumour size (Evaluable for Response Population)
Table 13.6 Progression-free survival (ITT Population)

Pharmacokinetics

Table 14.1 CT7001 trough plasma concentrations (unit) (PK Population)
Table 14.2 Mean individual CT7001 trough plasma concentration (ng/mL) (PK Population)
Table 14.3 Fulvestrant trough plasma concentrations (unit) (PK Population)
Table 14.4 Mean individual Fulvestrant trough plasma concentration (ng/mL) (PK Population)

Listings

Demographic, Baseline, Concomitant Medication and Other Subject-Specific Characteristics

Listing 1.1 Subject disposition (All subjects)
Listing 1.2 Analysis populations and reasons for exclusion (All subjects)
Listing 1.3 Protocol deviations (ITT Population)
Listing 2.1 Breast cancer history (ITT Population)

Listing 2.2 Prior cancer treatments (ITT Population)
Listing 2.3 Prior endocrine therapy (ITT Population)
Listing 2.4 Prior chemotherapy (ITT Population)
Listing 3.1 Prior, concomitant and post-treatment medications (ITT Population)
Listing 4.1 Polymorphisms of Dug Metabolising Enzymes (ITT Population)

Safety Data

Listing 5.1 Study drug administration of CT7001 (AT Population)
Listing 5.2 Study drug administration of Fulvestrant (AT Population)
Listing 5.3 Duration of exposure of CT7001 (AT Population)
Listing 5.4 Study drug accountability of CT7001 (AT Population)
Listing 6.1 Adverse events (AT Population)
Listing 6.2 Adverse events leading to death (AT Population)
Listing 6.3 Serious adverse events (AT Population)
Listing 6.4 Adverse events of special interest (AT Population)
Listing 6.5 Adverse events leading to discontinuation (AT Population)
Listing 6.6 Adverse events classified as Dose Limiting Toxicities (DLT) (AT Population)
Listing 7.1 Summary of deaths (AT Population)
Listing 8.1 Haematology (AT Population)
Listing 8.2 Clinical chemistry (AT Population)
Listing 8.3 Urinalysis (AT Population)
Listing 9.1 Weight and vital signs (AT Population)
Listing 10.1 Electrocardiogram – individual results (AT Population)
Listing 10.2 Electrocardiogram – finding/interpretation (AT Population)
Listing 11.1 Abnormal physical examination (AT Population)
Listing 12.1 ECOG performance status (AT Population)

Efficacy

Listing 13.1 Tumour lesion details and measurements (Evaluable for Response Population)
Listing 13.2 Individual efficacy response data (Evaluable for Response Population)
Listing 13.3 Duration of response and best objective response (Evaluable for Response Population)
Listing 13.4 Progression-free survival (ITT Population)

Pharmacokinetics

Listing 14.1 CT7001 individual trough plasma concentrations (All Subjects Dosed)
Listing 14.2 Mean plasma trough concentrations of CT7001 over treatment period (All Subjects Dosed)
Listing 14.3 Fulvestrant individual trough plasma concentrations (All Subjects Dosed)
Listing 14.4 Listing 14.4 Mean plasma trough concentrations of Fulvestrant over treatment period (All Subjects Dosed)

Pharmacogenomics

Listing 15.1 Pharmacogenomic results

Figures

Efficacy

Figure 1.1 Best percentage change from baseline in tumour size – Waterfall plot (Evaluable for Response population)

Figure 1.2 Percentage change from baseline in tumour size, individual subject data over time (Evaluable for Response Population)

Figure 1.3 Response profile for each subject – Swimmer plot (Evaluable for Response Population)

Figure 1.4 Kaplan-Meier plot of Duration of Response (DoR) (Evaluable for Response Population)

Figure 1.5 Kaplan-Meier plot of Progression-free Survival (PFS) (ITT Population)

Pharmacokinetics

Figure 2.1.1 CT7001 mean trough plasma concentrations, Linear/Linear scale, Geometric mean +/- SD (PK Population)
Figure 2.1.2 CT7001 mean trough plasma concentrations, Log/Linear scale, Geometric mean +/- SD (PK Population)
Figure 2.2.1 Fulvestrant mean trough plasma concentrations, Linear/Linear scale, Geometric mean +/- SD (PK Population)
Figure 2.2.2 Fulvestrant mean trough plasma concentrations, Log/Linear scale, Geometric mean +/- SD (PK Population)
Figure 3.1.1 CT7001 individual trough plasma concentrations, Linear/Linear scale (PK Population)
Figure 3.1.2 CT7001 individual trough plasma concentrations, Log/Linear scale (PK Population)
Figure 3.2.1 Fulvestrant individual trough plasma concentrations, Linear/Linear scale (PK Population)
Figure 3.2.2 Fulvestrant individual trough plasma concentrations, Log/Linear scale (PK Population)

DocuSign

Certificate Of Completion

Envelope Id: DF6E8E8EDB6B4D1BACF95AF2FA8879C6		Status: Completed
Subject: Please DocuSign: CT7001-001 SAP Module 2A FINAL v3.0.pdf		
Source Envelope:		
Document Pages: 31	Signatures: 2	Envelope Originator:
Certificate Pages: 5	Initials: 0	[redacted]
AutoNav: Enabled		Bionical Emas Group
EnvelopeId Stamping: Disabled		Spirella Building Suite 209
Time Zone: (UTC) Dublin, Edinburgh, Lisbon, London		Letchworth Garden City, Hertfordshire SG6 4ET
		[redacted]

Record Tracking

Status: Original<br>10-Aug-22 | 13:58	Holder: [redacted]	Location: DocuSign

Signer Events

Signer Events	Signature	Timestamp
[redacted]<br>Security Level: Email, Account Authentication (Required)	[redacted]<br>Signature Adoption: Pre-selected Style<br>Signature ID:<br>2746D8C1-6A10-4C14-BA65-6CAB6108B8E2<br>[redacted]<br>With Signing Authentication via DocuSign password<br>With Signing Reasons (on each tab):<br>I am the author of this document	Sent: 10-Aug-22 | 13:59<br>Viewed: 10-Aug-22 | 14:06<br>Signed: 10-Aug-22 | 14:06
**Electronic Record and Signature Disclosure:**<br>Accepted: 11-Feb-22 | 14:04<br>ID: fbddb06f-37cb-41d9-8443-e41e7aedbc51		
[redacted]<br>Senior Medical Director<br>Security Level: Email, Account Authentication (Required)	[redacted]<br>Signature Adoption: Pre-selected Style<br>Signature ID:<br>94C0D49A-78C7-48F0-9D99-8E746FA969BE<br>[redacted]<br>With Signing Authentication via DocuSign password<br>With Signing Reasons (on each tab):<br>I approve this document	Sent: 10-Aug-22 | 13:59<br>Viewed: 10-Aug-22 | 17:06<br>Signed: 10-Aug-22 | 17:07
**Electronic Record and Signature Disclosure:**<br>Accepted: 01-Oct-20 | 12:09<br>ID: 726a003c-f209-4c5d-91c4-e40caf870c0e		

In Person Signer Events	Signature	Timestamp

Editor Delivery Events	Status	Timestamp

Agent Delivery Events	Status	Timestamp

Intermediary Delivery Events	Status	Timestamp

Certified Delivery Events	Status	Timestamp

Carbon Copy Events	Status	Timestamp
[redacted]<br>Senior Clinical Operations&QA Specialist<br>Carrick Therapeutics Ltd.<br>Security Level: Email, Account Authentication (Required)<br>**Electronic Record and Signature Disclosure:**<br>Accepted: 30-Sep-20 | 14:34<br>ID: a1a88c10-8f87-452b-a745-f1cb8e68431b	COPIED	Sent: 10-Aug-22 | 13:59<br>Viewed: 10-Aug-22 | 14:34

Witness Events	Signature	Timestamp

Notary Events	Signature	Timestamp

Envelope Summary Events	Status	Timestamps
Envelope Sent	Hashed/Encrypted	10-Aug-22 | 13:59
Certified Delivered	Security Checked	10-Aug-22 | 17:06
Signing Complete	Security Checked	10-Aug-22 | 17:07
Completed	Security Checked	10-Aug-22 | 17:07

Payment Events	Status	Timestamps

Electronic Record and Signature Disclosure

ELECTRONIC RECORD AND SIGNATURE DISCLOSURE

From time to time, Bionical Emas (we, us or Company) may be required by law to provide to you certain written notices or disclosures. Described below are the terms and conditions for providing to you such notices and disclosures electronically through the DocuSign system. Please read the information below carefully and thoroughly, and if you can access this information electronically to your satisfaction and agree to this Electronic Record and Signature Disclosure (ERSD), please confirm your agreement by selecting the check-box next to 'I agree to use electronic records and signatures' before clicking 'CONTINUE' within the DocuSign system.

Getting paper copies

At any time, you may request from us a paper copy of any record provided or made available electronically to you by us. You will have the ability to download and print documents we send to you through the DocuSign system during and immediately after the signing session and, if you elect to create a DocuSign account, you may access the documents for a limited period of time (usually 30 days) after such documents are first sent to you. After such time, if you wish for us to send you paper copies of any such documents from our office to you, you will be charged a $0.00 per-page fee. You may request delivery of such paper copies from us by following the procedure described below.

Withdrawing your consent

If you decide to receive notices and disclosures from us electronically, you may at any time change your mind and tell us that thereafter you want to receive required notices and disclosures only in paper format. How you must inform us of your decision to receive future notices and disclosure in paper format and withdraw your consent to receive notices and disclosures electronically is described below.

Consequences of changing your mind

If you elect to receive required notices and disclosures only in paper format, it will slow the speed at which we can complete certain steps in transactions with you and delivering services to you because we will need first to send the required notices or disclosures to you in paper format, and then wait until we receive back from you your acknowledgment of your receipt of such paper notices or disclosures. Further, you will no longer be able to use the DocuSign system to receive required notices and consents electronically from us or to sign electronically documents from us.

All notices and disclosures will be sent to you electronically

Unless you tell us otherwise in accordance with the procedures described herein, we will provide electronically to you through the DocuSign system all required notices, disclosures, authorizations, acknowledgements, and other documents that are required to be provided or made available to you during the course of our relationship with you. To reduce the chance of you inadvertently not receiving any notice or disclosure, we prefer to provide all of the required notices and disclosures to you by the same method and to the same address that you have given us. Thus, you can receive all the disclosures and notices electronically or in paper format through the paper mail delivery system. If you do not agree with this process, please let us know as described below. Please also see the paragraph immediately above that describes the consequences of your electing not to receive delivery of the notices and disclosures electronically from us.

How to contact Bionical Emas:

You may contact us to let us know of your changes as to how we may contact you electronically, to request paper copies of certain information from us, and to withdraw your prior consent to receive notices and disclosures electronically as follows:
To contact us by email send messages to: DISTDPO@Bionical-Emas.com
To contact us by paper mail, please send correspondence to:
Bionical Emas
63-65 Knowl Piece
Wilbury Way
Hitchin, Hertfordshire, United Kingdom, SG4 0TY

To advise Bionical Emas of your new email address

To let us know of a change in your email address where we should send notices and disclosures electronically to you, you must send an email message to us at DISTDPO@Bionical-Emas.com and in the body of such request you must state: your previous email address, your new email address. We do not require any other information from you to change your email address.

If you created a DocuSign account, you may update it with your new email address through your account preferences.

To request paper copies from Bionical Emas

To request delivery from us of paper copies of the notices and disclosures previously provided by us to you electronically, you must send us an email to DISPDPO@Bionical-emas.com and in the body of such request you must state your email address, full name, mailing address, and telephone number. We will discuss any anticipated fees and how these are collected with you.

To withdraw your consent with Bionical Emas

To inform us that you no longer wish to receive future notices and disclosures in electronic format you may:

i. decline to sign a document from within your signing session, and on the subsequent page, select the check-box indicating you wish to withdraw your consent, or you may;

ii. send us an email to DISTDPO@Bionical-Emas.com and in the body of such request you must state your email, full name, mailing address, and telephone number. We do not need any other information from you to withdraw consent.. The consequences of your withdrawing consent will be discussed with you..

Required hardware and software

The minimum system requirements for using the DocuSign system may change over time. The current system requirements are found here: https://support.docusign.com/guides/signer-guide-signing-system-requirements.

Acknowledging your access and consent to receive and sign documents electronically

To confirm to us that you can access this information electronically, which will be similar to other electronic notices and disclosures that we will provide to you, please confirm that you have read this ERSD, and (i) that you are able to print on paper or electronically save this ERSD for your future reference and access; or (ii) that you are able to email this ERSD to an email address where you will be able to print on paper or save it for your future reference and access. Further, if you consent to receiving notices and disclosures exclusively in electronic format as described herein, then select the check-box next to 'I agree to use electronic records and signatures' before clicking 'CONTINUE' within the DocuSign system.

By selecting the check-box next to 'I agree to use electronic records and signatures', you confirm that:

- You can access and read this Electronic Record and Signature Disclosure; and
- You can print on paper this Electronic Record and Signature Disclosure, or save or send this Electronic Record and Disclosure to a location where you can print it, for future reference and access; and
- Until or unless you notify Bionical Emas as described above, you consent to receive exclusively through electronic means all notices, disclosures, authorizations, acknowledgements, and other documents that are required to be provided or made available to you by Bionical Emas during the course of your relationship with Bionical Emas.
