# Supplementary material for: Dose escalation and expansion cohorts in patients with advanced breast cancer in a Phase I study of the CDK7-inhibitor samuraciclib
Source: Nat Commun. 2023 Jul 24;14:4444. doi: 10.1038/s41467-023-40061-y (PMC10366102; doi:10.1038/s41467-023-40061-y)
Supplement: Supplementary file 3 — Reporting Summary [file 41467_2023_40061_MOESM3_ESM.pdf]

nature portfolio

Corresponding author(s): Dr Matthew Krebs

Last updated by author(s): 26/05/2023

Reporting Summary

Nature Portfolio wishes to improve the reproducibility of the work that we publish. This form provides structure for consistency and transparency in reporting. For further information on Nature Portfolio policies, see our Editorial Policies and the Editorial Policy Checklist.

Statistics

For all statistical analyses, confirm that the following items are present in the figure legend, table legend, main text, or Methods section.

n/a	Confirmed	
☐	☒	The exact sample size (n) for each experimental group/condition, given as a discrete number and unit of measurement
☐	☒	A statement on whether measurements were taken from distinct samples or whether the same sample was measured repeatedly
☐	☒	The statistical test(s) used AND whether they are one- or two-sided<br>*Only common tests should be described solely by name; describe more complex techniques in the Methods section.*
☐	☒	A description of all covariates tested
☐	☒	A description of any assumptions or corrections, such as tests of normality and adjustment for multiple comparisons
☐	☒	A full description of the statistical parameters including central tendency (e.g. means) or other basic estimates (e.g. regression coefficient) AND variation (e.g. standard deviation) or associated estimates of uncertainty (e.g. confidence intervals)
☐	☒	For null hypothesis testing, the test statistic (e.g. F, t, r) with confidence intervals, effect sizes, degrees of freedom and P value noted<br>*Give P values as exact values whenever suitable.*
☒	☐	For Bayesian analysis, information on the choice of priors and Markov chain Monte Carlo settings
☒	☐	For hierarchical and complex designs, identification of the appropriate level for tests and full reporting of outcomes
☒	☐	Estimates of effect sizes (e.g. Cohen's d, Pearson's r), indicating how they were calculated

Our web collection on statistics for biologists contains articles on many of the points above.

Software and code

Policy information about availability of computer code

Data collection	IBM Clinical Development Version 2019.3.0.1
Data analysis	SAS System version 9.1 for clinical data, stained biopsy data scanned using Hamamtsu Nanozoomer 2.0 HT and analyzed using Indica Labs HALO software version 3.0, and biomarker data exported and analysed using FlowJo (Treestar) version 10.4.2.

For manuscripts utilizing custom algorithms or software that are central to the research but not yet described in published literature, software must be made available to editors and reviewers. We strongly encourage code deposition in a community repository (e.g. GitHub). See the Nature Portfolio guidelines for submitting code & software for further information.

Data

Policy information about availability of data

All manuscripts must include a data availability statement. This statement should provide the following information, where applicable:

- Accession codes, unique identifiers, or web links for publicly available datasets
- A description of any restrictions on data availability
- For clinical datasets or third party data, please ensure that the statement adheres to our policy

The data from this study cannot be made publicly available due to the sponsor’s contractual obligations. Data may be requested after the product and indication has been approved by major health authorities and/or 24 months after completion of all the arms of the NCT03363893 trial. Qualified researchers should submit a proposal to the corresponding author (matthew.krebs@manchester.ac.uk) outlining the reasons for requiring the data. Applications should specifically outline the data the parties are interested in receiving and how the data will be used; the use of the data must also comply with the country- or region-specific regulations. A

signed data access agreement with the sponsor is required before accessing the shared data. The study protocol is included in the Supplementary Information published online with this article.

Human research participants

Policy information about studies involving human research participants and Sex and Gender in Research.

Reporting on sex and gender	Both sexes were recruited into Module 1A.<br>Although studying patients with triple negative breast cancer, Module1B-1 was open to both sexes.<br>Module 2A, studying patients with hormone receptor positive and HER-2 negative breast cancer, was open only to women.
Population characteristics	In Module 1A, mean age was 59.6 years and 64% of patients were female (Table 1). Primary malignancies were predominantly breast (30%) or colorectal (24%). 87.9% of patients had received prior chemotherapy and 36.4% had undergone hormone therapy. In the paired biopsy cohort, mean age was 56.5 years, all patients had undergone prior chemotherapy and 90.9% had received prior hormone therapy.<br>In Module 1B-1, the mean age was 53.6 years, and all patients were female. Patients had received a median of 2 (range 1-3) lines of prior chemotherapy in the advanced TNBC setting.<br>In Module 2A, mean age was 60.4 years. All patients had received prior AI in combination with CDK4/6. Six of 31 patients were pre menopausal.
Recruitment	All patients were recruited in a non-randomized open label fashion by the investigator at each site based on the investigator's pool of potential patients. Patients were then screened against the inclusion/exclusion criteria and included if eligible.
Ethics oversight	The study was approved by the regulatory authorities, and the Yorkshire & The Humber - Leeds West Research Ethics Committee, Jarrow, UK and the local ethics committees for each site. The study was conducted in accordance with ICH-GCP guidelines and all legal, regulatory, ethical, and data protection requirements. All patients provided written informed consent prior to participation.

Note that full information on the approval of the study protocol must also be provided in the manuscript.

Field-specific reporting

Please select the one below that is the best fit for your research. If you are not sure, read the appropriate sections before making your selection.

☒ Life sciences ☐ Behavioural & social sciences ☐ Ecological, evolutionary & environmental sciences

For a reference copy of the document with all sections, see nature.com/documents/nr-reporting-summary-flat.pdf

Life sciences study design

All studies must disclose on these points even when the disclosure is negative.

Sample size	The sample size of cohorts for each study module were based on the requirement for adequate safety, PK, and PDc data, balanced with exposure of as few subjects as possible to the IMP and study procedures. The cohorts could be expanded by additional evaluable subjects as specified within the respective modules, and at doses at or above the established minimally biological active dose.<br><br>This was a Phase I/IIa study with no formal hypothesis to confirm; therefore, no formal sample size calculation was performed. The group sizes were considered sufficient for preliminary exploration of the specified objectives of the study.
Data exclusions	No data were excluded.
Replication	Oncology phase 1/2 single arm open label design, therefore not applicable.
Randomization	No randomisation, as oncology phase 1/2 single arm, open label design.
Blinding	No blinding, as oncology phase 1/2 single arm, open label design.

Reporting for specific materials, systems and methods

We require information from authors about some types of materials, experimental systems and methods used in many studies. Here, indicate whether each material, system or method listed is relevant to your study. If you are not sure if a list item applies to your research, read the appropriate section before selecting a response.

Materials & experimental systems

n/a	Involved in the study
[ ]	[x] Antibodies
[x]	[ ] Eukaryotic cell lines
[x]	[ ] Palaeontology and archaeology
[x]	[ ] Animals and other organisms
[ ]	[x] Clinical data
[x]	[ ] Dual use research of concern

Methods

n/a	Involved in the study
[x]	[ ] ChIP-seq
[ ]	[x] Flow cytometry
[x]	[ ] MRI-based neuroimaging

Antibodies

Antibodies used: See Supplementary Table 12

Validation: See Supplementary Table 12

Clinical data

Policy information about clinical studies

All manuscripts should comply with the ICMJEguidelines for publication of clinical research and a completedCONSORT checklist must be included with all submissions.

Clinical trial registration: NCT03363893

Study protocol: The relevant redacted protocols have been supplied in the Supplementary Information file published online with this article.

Data collection: Clinical data was collected at recruiting sites in the UK and the USA by qualified investigators with experience in oncology clinical trials. All investigators who recruited patients are listed as authors on this manuscript.
In Module 1A, patients participated from November 2017 until the last patient completed treatment in May 2020.
In Module 1B-1, patients participated from January 2019 until the last patient completed treatment in May 2021.
In Module 2A, patients were recruited from November 2019 to March 2021

Outcomes: In Module 1A initial efficacy was evaluated using the disease control rate (DCR) defined as percentage of participants with a complete response (CR) or partial response (PR) or stabilization of disease at first on treatment RECIST assessment. In Modules 1B-1 and Module 2A the Objective Response Rate (ORR) was defined as the percentage of participants who had at least 1 objective response (CR or PR) prior to any evidence of progression and the Clinical benefit rate (CBR) was defined as the percentage of patients with CR or PR or stabilization of disease for at least 24 weeks between enrolment and disease progression or death due to any cause. RECIST V1.1 endpoints were assessed in the response evaluable population - defined as all patients who received ≥1 dose of samuraciclib and had a post-baseline tumor assessment. Statistical analyses of progression-free survival (PFS) using the Kaplan-Meier method were performed on the intent-to-treat (ITT) population - defined as all enrolled patients. AEs were summarized from the Safety Population - defined as all patients who received at least 1 dose of samuraciclib - using the MedDRA system organ class (SOC), preferred term (PT), and graded according to CTCAE V5.0

Flow Cytometry

Plots

Confirm that:

- [x] The axis labels state the marker and fluorochrome used (e.g. CD4-FITC).
- [x] The axis scales are clearly visible. Include numbers along axes only for bottom left plot of group (a 'group' is an analysis of identical markers).
- [x] All plots are contour plots with outliers or pseudocolor plots.
- [x] A numerical value for number of cells or percentage (with statistics) is provided.

Methodology

Sample preparation: 1) Cells were pelleted and resuspended in 2 mL 70% Ethanol and incubated on ice for 1 hour.
2) Cells were then pelleted and then washed and resuspended in ice-cold PBS.
3) Cells were then pelleted and resuspended in 200 µL of FACS buffer (PBS +0.5% BSA) containing primary antibody at a 1:100 dilution.
4) Cells then incubated for 1 hour on ice.
5) Wash cells with 1 mL ice-cold PBS and re-suspend in 200 µL of FACS buffer (PBS +0.5% BSA) containing the secondary antibody (Goat anti-rabbit AF488, 1:1000 dilution).
6) Place the tubes on ice for 1 hour
7) Wash cells with 1 mL ice-cold PBS and re-suspend in 500 µL ice cold PBS. Samples were stored at 4oC prior to flow cytometry analysis.
Cells were analysed cells on a BD Fortessa X20, acquiring 100,000 events. Cells are gated by side scatter area (SSC-A) versus

	forward scatter area (FSC-A) to separate the three populations of cells: lymphocytes, monocytes and granulocytes (see Supplementary Figure 11). Data were exported and analysed using FlowJo (Treestar) version 10.4.2 to report the median fluorescence intensity of staining in the cell populations.
Instrument	BD Fortessa X20
Software	FlowJo (Treestar) version 10.4.2
Cell population abundance	Lymphocytes typically represented 25-30% of patient peripheral blood mononucleate cell preparations.
Gating strategy	See Supplementary Figure 11. Gating was performed based on forward and side scatter for lymphocytes.

☒ Tick this box to confirm that a figure exemplifying the gating strategy is provided in the Supplementary Information.
